# Electroreductive Radical Olefin Difunctionalization with Fluorinated Gases Enabled by Dosage Delivery from a Metal–Organic Framework

**DOI:** 10.1021/jacs.6c01583

**Published:** 2026-04-22

**Authors:** Yihuan Lai, Jiachen He, Oliver P. Lambert, Joharimanitra Randrianandraina, Jung-Hoon Lee, Phillip J. Milner

**Affiliations:** a Department of Chemistry and Chemical Biology, 5922 Cornell University, Ithaca, New York 14853, United States; b Computational Science Research Center, Korea Institute of Science and Technology (KIST), Seoul 02792, Republic of Korea; c KU-KIST Graduate School of Converging Science and Technology, Korea University, Seoul 02841, Republic of Korea

## Abstract

Fluoroalkyl groups are crucial for tuning the pharmacokinetic properties of drug-like molecules, motivating the development of practical methods for their late-stage installation. Despite extensive progress, most contemporary fluoroalkylation strategies rely on impractical reagents or harsh reaction conditions, limiting their sustainability and scalability. Electrochemistry offers a compelling alternative by enabling controlled and tunable radical generation under mild conditions; however, electrochemical fluoroalkylation reactions, particularly reductive transformations, remain significantly underdeveloped. Herein, we report a general platform for electroreductive olefin difunctionalization that employs simple fluoroalkyl iodide gases (CF_3_I, CF_3_CF_2_I, and CF_2_HI) handled safely using the robust, inexpensive, recyclable, and redox-innocent metal–organic framework (MOF) Al–fum. Our method delivers streamlined access to fluoroalkylated products and enables electroreductive olefin difunctionalization for installing the medicinally important CF_2_H group. All reactions proceed with broad functional group tolerance (including for other alkyl halides) and are suitable for follow-on diversification. Together, our findings expand the scope of electroreductive fluoroalkylation chemistry and establish gas–MOF reagents as powerful tools to deliver fluoroalkyl iodide gases for electroorganic synthesis.

## Graphical abstract

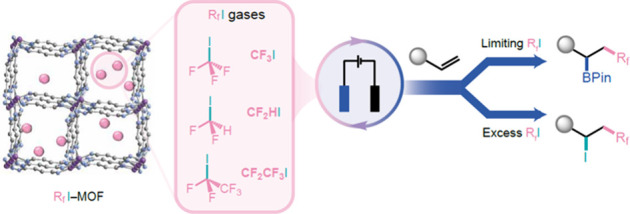

## Introduction

Fluorine plays an essential role in organic chemistry, underpinning a substantial portion of contemporary pharmaceuticals and agrochemicals (Figure 1a).^1–5^ This is because fluorination often leads to marked improvements in biological activity, metabolic stability, and physicochemical properties. Fluoroalkyl groups such as trifluoromethyl (CF_3_−), pentafluoroethyl (CF_3_CF_2_−), difluoromethyl (CF_2_H−), and higher perfluoroalkyl units function as bioisosteres for common functional groups and enable precise modulation of electronic, steric, and lipophilic parameters.^6–11^ Accordingly, the development of efficient fluoroalkylation methods remains a central goal of synthetic chemistry. However, many fluoroalkylation reactions rely on costly, high-molecular-weight reagents or require multistep preparations of specialized precursors,^12–14^ highlighting the need for efficient, atom-economical, and broadly applicable methods that employ simple fluorinated building blocks.

**Figure 1:**
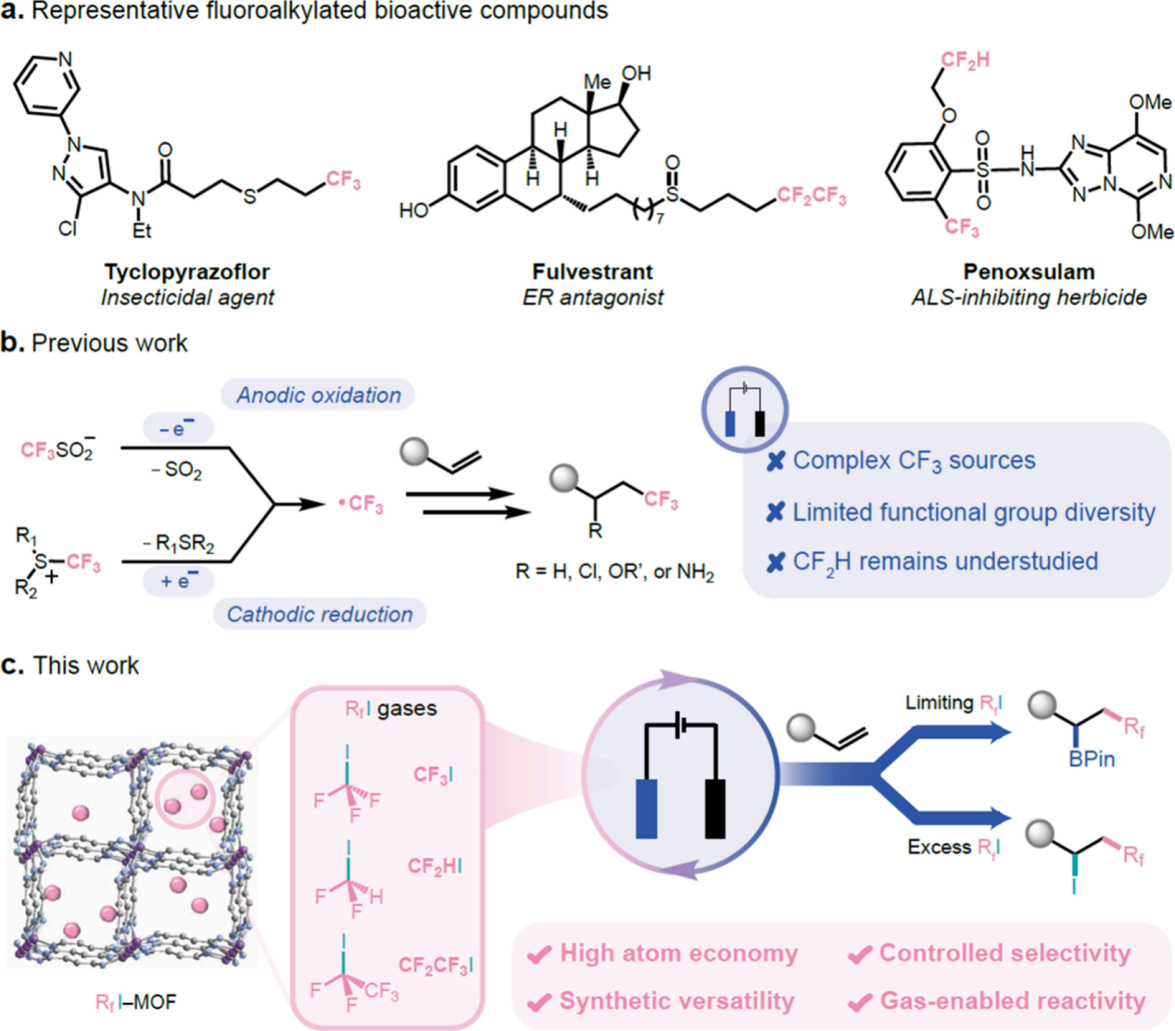
(a) Examples of fluoroalkylated bioactive compounds. (b) Previous work on electrochemical generation of CF_3_· through anodic oxidation or cathodic reduction. (c) MOF-enabled delivery of fluorinated gases for electroreductive olefin difunctionalization (this work).

Electrochemistry has recently re-emerged as a powerful tool for organic synthesis, offering inherent sustainability and complementary selectivity compared to conventional methods.^15–17^ In this context, electrochemical radical olefin difunctionalization represents a promising platform for constructing complex molecules.^18–22^ Because alkenes are among the most abundant and readily accessible functional groups in feedstock chemicals, pharmaceuticals, and natural products, their difunctionalization provides a versatile entry point for molecular diversification.^23–25^ These features make radical olefin difunctionalization a highly attractive strategy for the selective introduction of fluoroalkyl groups (Figure 1b). However, progress in electrochemical fluoroalkyl radical generation has been uneven: while CF_3_ radicals (CF_3_·) are commonly accessed via anodic oxidation,^26–30^ cathodic or reductive electrogeneration of CF_3_· remains comparatively underexplored,^31^ despite its potential advantages such as tolerance of oxidatively sensitive functional groups and electron-rich heterocycles.

Thus, far, electroreductive radical olefin difunctionalization has been limited to anti-Markovnikov hydrotrifluoromethylation,^31^ underscoring the need for reductive strategies that combine CF_3_ installation with more synthetically versatile functional groups. Relatedly, previously reported oxidative systems also pair CF_3_ incorporation with a limited set of second functional groups (e.g., NH_2_,^32^ Cl,^33^ or OR^34,35^ ), constraining their synthetic utility. Moreover, the CF_2_H group has recently gained prominence because it combines strong inductive effects with the capacity to serve as a hydrogen-bond donor,^7,10,36,37^ yet electroreductive difunctionalization methods compatible with difluoromethylation remain underdeveloped.^38–40^ Analogous to CF_3_, previous electroreductive methods for CF_2_H incorporation have been largely limited to hydrodifluoromethylation, thereby restricting their utility for late-stage functionalization. Further, these strategies are generally effective only for activated alkenes and exhibit narrow substrate scopes.^39^ Last, existing electrochemical difunctionalization methods typically rely upon complex fluoroalkylating agents, restricting their atom economy and practical simplicity.^31–35,41^ Together, these gaps highlight the need for efficient and sustainable platforms for transforming readily available fluorinated precursors into structurally diverse fluoroalkylated products.

Due to their low molecular weights and high fluorine contents, hydrofluorocarbon gases represent highly atom-economical fluoroalkyl sources, yet volatility and handling challenges limit their practical use.^42,43^ Batch-handling methods using balloons, *in situ* generation, and stock solutions suffer from poor reproducibility and safety risks, while continuous flow-based approaches, though effective, require specialized equipment.^44–48^ These limitations highlight the need for broadly accessible strategies that can engage simple fluorinated gases with precise stoichiometric control. To address these challenges, we^49–51^ and others^52,53^ recently introduced the use of metal–organic frameworks (MOFs) to adsorb and deliver (fluorinated) gases in a solid, easy-to-handle form.^54^ Although these gas–MOF reagents have proven valuable for the development of novel thermal^49^ and photochemical^51^ transformations, their compatibility with electrochemistry remains unproven. In particular, it is important to determine whether the MOF scaffold can withstand electrochemical conditions and whether MOF-bound gases can effectively reach electrode interfaces, undergo efficient single-electron activation, and participate in radical pathways.

Here, we report a general electroreductive radical olefin difunctionalization platform that employs simple fluoroalkyl iodide gases, including CF_3_I, CF_3_CF_2_I, and CF_2_HI, stored within the inexpensive MOF Al­(OH)­fum (fum^2–^ = fumarate) or Al–fum^55^ to access a diverse set of fluoroalkylated products (Figure 1c). For CF_3_I, solid-state gas delivery provides exceptional control over reagent stoichiometry, enabling tunable chemoselectivity between CF_3_-borylated or CF_3_-iodinated products simply by adjusting the equivalents of the CF_3_I–Al–fum reagent. This product-level control is difficult to achieve with gaseous CF_3_I (boiling point = –22.5 °C) alone. Additionally, we demonstrate that CF_2_HI–Al–fum enables direct cathodic generation of the CF_2_H radical (CF_2_H·), providing an efficient route to CF_2_H-iodinated products from unactivated alkenes. Together with the successful engagement of CF_3_CF_2_I–Al–fum, these results establish that the combination of solid-state gas reagents and controlled electrochemical activation enables an atom-economical approach to CF_3_-, C_2_F_5_-, and CF_2_H-containing products. MOF-mediated delivery facilitates safe and precise handling of gaseous reagents, as control experiments indicate that the MOF does not significantly alter reaction efficiency, serving primarily as a convenient delivery vehicle for rapid reaction optimization. Overall, our results broaden the synthetic scope of reductive radical fluoroalkylation and establish the first example of electrochemical activation of MOF-delivered fluorinated gases to enable synthetically valuable transformations.

## Results and Discussion

To evaluate the feasibility of electroreductive radical olefin difunctionalization with fluorinated gases, we selected the CF_3_-borylation of 1-decene (**2**) as an ideal starting point (Figure 2),^56^ as CF_3_ installation is both important in medicinal chemistry and synthetically demanding.^6,8,11^ In addition, the obtained borylated products offer medicinally valuable handles and represent versatile linchpins for late-stage diversification.^57–60^ Al–fum was chosen as the initial nanovessel of choice due to its low cost, scalability, and excellent compatibility with radical generation via photocatalysis (see SI Section 2 for synthetic details).^51^ The solid reagent CF_3_I–Al–fum could be readily prepared from a balloon of CF_3_I and activated Al–fum using a simple gas-loading procedure (see SI Section 3 for details).

**Figure 2:**
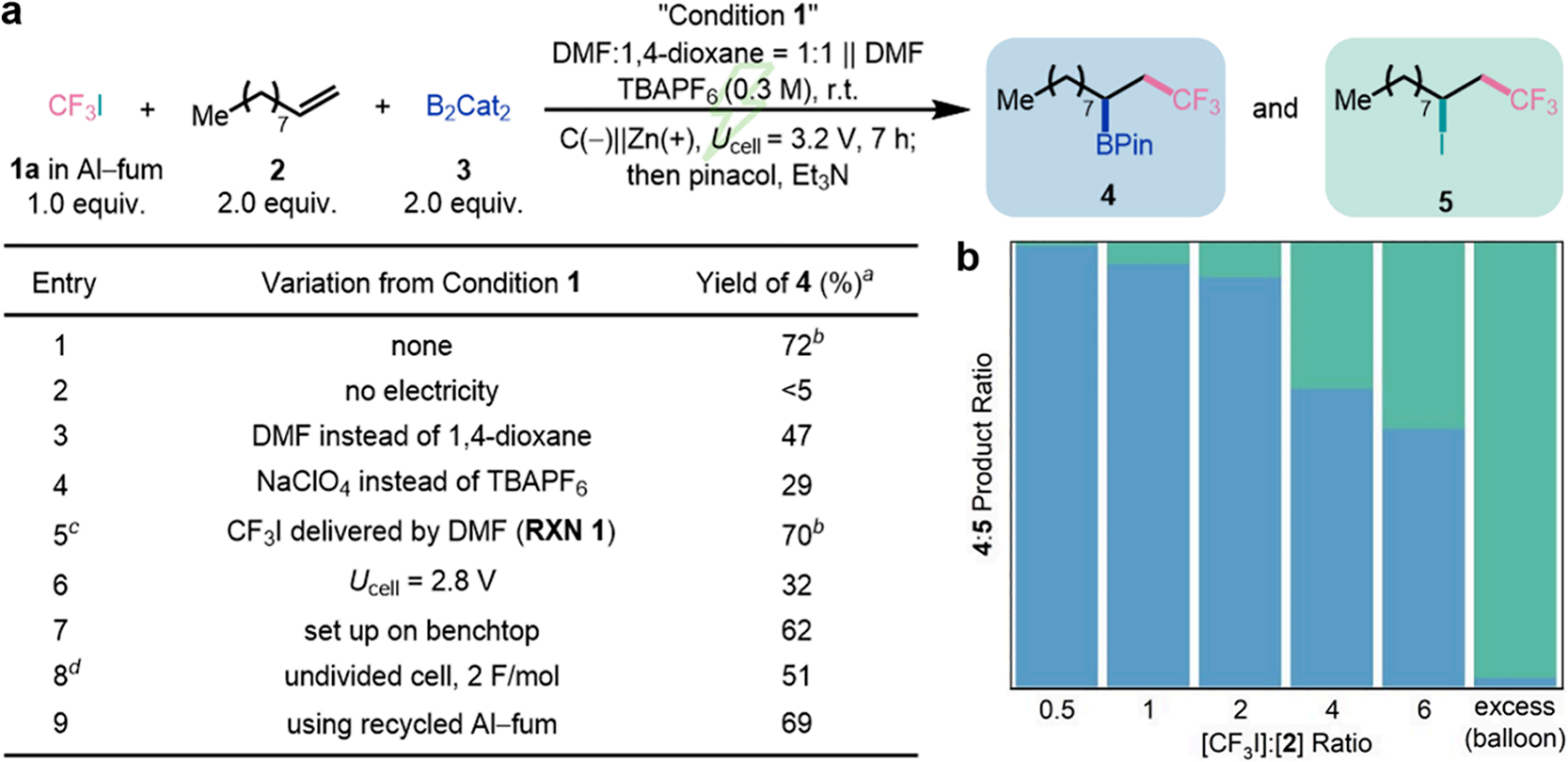
(a) Optimization of the electroreductive CF_3_-borylation of 1-decene (**2**). “Condition **1**”: cathodic chamber: **1a** (in Al*–*fum, 0.4 mmol, 1.0 equiv), **2** (2.0 equiv), **3** (2.0 equiv), TBAPF_6_ (0.3 M), DMF (1.0 mL), 1,4-dioxane (1.0 mL); anodic chamber: TBAPF_6_ (0.3 M), DMF (2.0 mL). After completion of the reaction, Et_3_N (4.0 equiv) and pinacol (4.0 equiv) were added to the cathodic chamber, and the mixture was stirred for another 1 h. ^*a*^Yields obtained by *^19^* F NMR (CF_3_Ph as internal standard). ^*b*^Average of three independent trials. ^*c*^CF_3_I delivered as a stock solution in DMF (0.75 M) instead of using CF_3_I–Al–fum. ^*d*^C­(−)­C­(+), DIPEA (3.0 equiv), in DMF (2 mL), I_c_ = 5 mA. (b) Tunable chemoselectivity between borylation (blue) and iodination (green) enabled by dosage CF_3_I delivery from CF_3_I–Al–fum.

After optimization, we found that the use of 1.0 equiv of CF_3_I–Al–fum, 2.0 equiv of 1-decene (**2**), and 2.0 equiv of bis­(catecholato)­diboron (B_2_Cat_2_, **3**), along with tetrabutylammonium hexafluorophosphate (TBAPF_6_) as the electrolyte, porous carbon foam as the cathode, DMF/1,4-dioxane (1:1 v/v) as the cathodic chamber solvent, Zn as the sacrificial anode, DMF as the anodic chamber solvent, and an applied cell potential of 3.2 V at room temperature (r.t.) for 7 h (≈ 1.5 F/mol) in a divided cell, followed by treatment with Et_3_N and pinacol, affords the desired product **4** in 72% yield (Entry 1, Figure 2a). Independent gas-loading experiments demonstrated highly reproducible CF_3_I uptake capacities, and reactions conducted with different batches of loaded material provided comparable yields, confirming that reaction reproducibility is not affected by loading percentage (SI Table S5). The reaction is proposed to proceed via cathodic generation of CF_3_· from CF_3_I, followed by radical addition to the alkene and boron capture of the resulting alkyl radical (SI Figure S47, see discussion below for mechanistic details). Precise control over the CF_3_I/alkene ratio using CF_3_I–Al–fum reveals a tunable switch between iodination and borylation pathways (Figure 2b). Specifically, under excess CF_3_I (including from a balloon), the reaction proceeds largely through an atom-transfer radical addition (ATRA) pathway to afford iodinated product **5**, whereas limiting CF_3_I selectively yields the borylated product **4**. Thus, the use of CF_3_I as the limiting reagent (easily achieved using CF_3_I–Al–fum) affords the highest selectivity (99:1) for **4**. The selected stoichiometry for “condition 1” reflects a balance between radical generation and pathway selectivity. Limiting CF_3_I suppresses competing ATRA pathways, while excess alkene and B_2_Cat_2_ ensure efficient interception of CF_3_· and subsequent trapping of the alkyl radical, thereby favoring borylation over iodination.

Control experiments confirm that electrical input is essential for product formation, as no reaction occurred without an applied cell potential (Entry 2, Figure 2a). Removing 1,4-dioxane from the cathodic solvent mixture leads to a diminished yield (47%), which is consistent with its proposed role to improve the solubility of hydrophobic CF_3_I (Entry 3, Figure 2a).^61^ TBAPF_6_ was also found to be a superior electrolyte compared to NaClO_4_ (Entry 4, Figure 2a). When CF_3_I was delivered as a stock solution in DMF,^62^ a comparable yield (three trials, 70% in average) was obtained, indicating that Al–fum does not negatively or positively influence the reaction yield (Entry 5, Figure 2a; see further discussion below). This observation further supports that the primary role of the MOF is to regulate reagent delivery and stoichiometry, rather than to influence the intrinsic reaction kinetics or mechanism. Lowering the applied cell potential to 2.8 V resulted in a substantial drop in yield, underscoring the need for a sufficient electrochemical driving force to reduce CF_3_I (Entry 6, Figure 2a). When the reaction was set up on the benchtop under N_2_, the yield was similar to that obtained for a reaction set up in an N_2_-filled glovebox (62%, Entry 7, Figure 2a), highlighting the practical utility of this protocol. Replacement of the sacrificial Zn anode with *N*,*N*-diisopropylethylamine (DIPEA) as a sacrificial reductant in conjunction with a carbon anode enabled the reaction to be performed in an undivided cell under constant-current conditions, albeit with a slight decrease in yield (51%, Entry 8, Figure 2a). Finally, the use of recycled Al–fum, regenerated by rinsing, reactivating, and redosing, provided **4** in similar yield as that obtained with fresh material (69% vs 72%, Entry 9, Figure 2a). The recyclability of the MOF makes it a sustainable alternative to traditional CF_3_ reagents. Together, these findings highlight the unique advantages offered by employing CF_3_I as the limiting reagent in electroreductive radical CF_3_-difunctionalization, which is challenging to achieve with the gas itself but straightforward with CF_3_I–Al–fum.

With the optimized reaction conditions in hand, we next investigated the scope of the developed electroreductive alkene radical CF_3_-borylation (Table 1). A wide range of unactivated alkenes bearing pharmaceutically relevant functional groups proved compatible, including boronate (**7**), ester (**9**, **12**, **13**, **16**, **18**, **19**, **22**), organohalide (**9**, **10**, **13**, **15**, **26**), silane (**20**), ether (**15**, **21, 24**, **26**, **27**, **28**), acetal (**24**), cyclopropane (**16**, **26**), nitrile (**27**), and ketone (**28**) groups. Notably, both alkyl (**9**, **10**, **26**) and aryl (**13**, **15**) halides remain intact, despite their comparable half-peak reduction potentials (*E*_p/2_) to CF_3_I (−1.36 V vs Fc/Fc^+^, SI Figure S28),^63^ demonstrating the high chemoselectivity of this method. A variety of oxidatively sensitive, electron-rich heterocycles, including thiophene (**12**), carbazole (**17**), thiazole (**18**, **27**), benzofuran (**19**), and oxazole (**23**), were also well-tolerated. Notably, for a substrate bearing both a terminal and an internal olefin (**22**), the reaction exhibited exclusive regiocontrol, functionalizing only the more-reactive terminal alkene. The broad functional group compatibility of this radical difunctionalization protocol encouraged us to also investigate the late-stage functionalization of complex, biologically active molecules. Consistently, alkenes derived from oxaprozin (**23**), diacetone-d-glucose (**24**), ibuprofen (**25**), ciprofibrate (**26**), febuxostat (**27**), and estrone (**28**) all reacted smoothly.

**Table 1:**
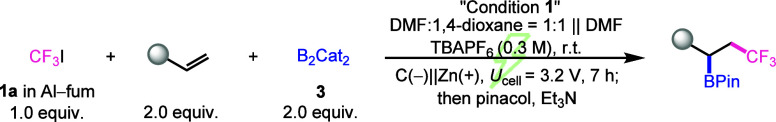

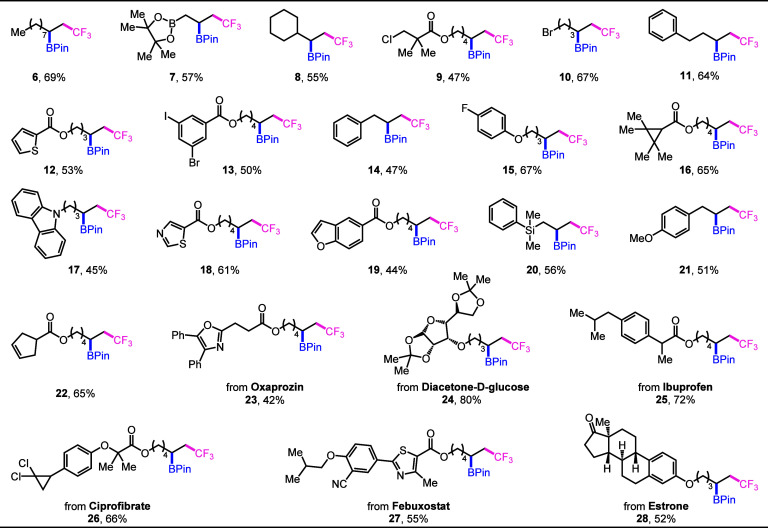
Scope of the Electroreductive Radical Difunctionalization of Unactivated Olefins by Using CF_3_I–Al–fum^a^

a “Condition **1**”: cathodic chamber: **1a** (in Al–fum, 0.4 mmol, 1.0 equiv), alkene (2.0 equiv), **3** (2.0 equiv), TBAPF_6_ (0.3 M), DMF (1.0 mL), 1,4-dioxane (1.0 mL); anodic chamber: TBAPF_6_ (0.3 M), DMF (2.0 mL). After completion of the reaction, Et_3_N (4.0 equiv) and pinacol (4.0 equiv) were added to the cathodic chamber, and the mixture was stirred for another 1 h. Isolated yields are shown.

Motivated by the successful functionalization of CF_3_I, we next extended this electroreductive radical difunctionalization platform to other fluoroalkyl iodides (Table 2), thereby accessing a broader fluoroalkyl design space while enabling systematic modulation of steric, electronic, and lipophilic properties relevant to medicinal chemistry.^3,6,48,56^ For the first time, we found that the gaseous reagent CF_3_CF_2_I (boiling point = 12–13 °C) can be readily adsorbed into Al–fum with a high gravimetric loading capacity (67 wt %, see SI Section 3 for details). Density functional theory (DFT) calculations were conducted to elucidate its preferred binding mode within the MOF (Figure 3a, see SI Section 6 for details). Analysis of the optimized CF_3_CF_2_I–Al–fum structure revealed multiple strong hydrogen-bonding interactions (with distances ranging from 2.40 to 3.15 Å), including C=C–H···F–C, μ–OH···I–C and C=C–H···I–C interactions. Many additional weaker interactions are also predicted (SI Figure S25), contributing to the highest adsorption enthalpy (−Δ*H*_ads_ = 57 kJ/mol) reported for a hydrofluorocarbon gas in Al–fum to date.^51^ To demonstrate the practical utility of CF_3_CF_2_I–Al–fum reagent for routine synthetic applications, we assessed its long-term stability under conditions commonly encountered during laboratory storage. Gas-loaded samples (30–50 mg) were kept in a glovebox freezer (−30 °C), a standard laboratory freezer (−25 °C), or a benchtop desiccator at room temperature for up to three months. Gas retention was evaluated by quantifying the amount of CF_3_CF_2_I released upon submerging the reagent in solvent using ^19^F NMR. Remarkably, CF_3_CF_2_I–Al–fum retained more than 80% of its initial loading capacity after three months under each storage condition (Figure 3b), confirming the long-term storability of this new gas–MOF reagent.

**Figure 3:**
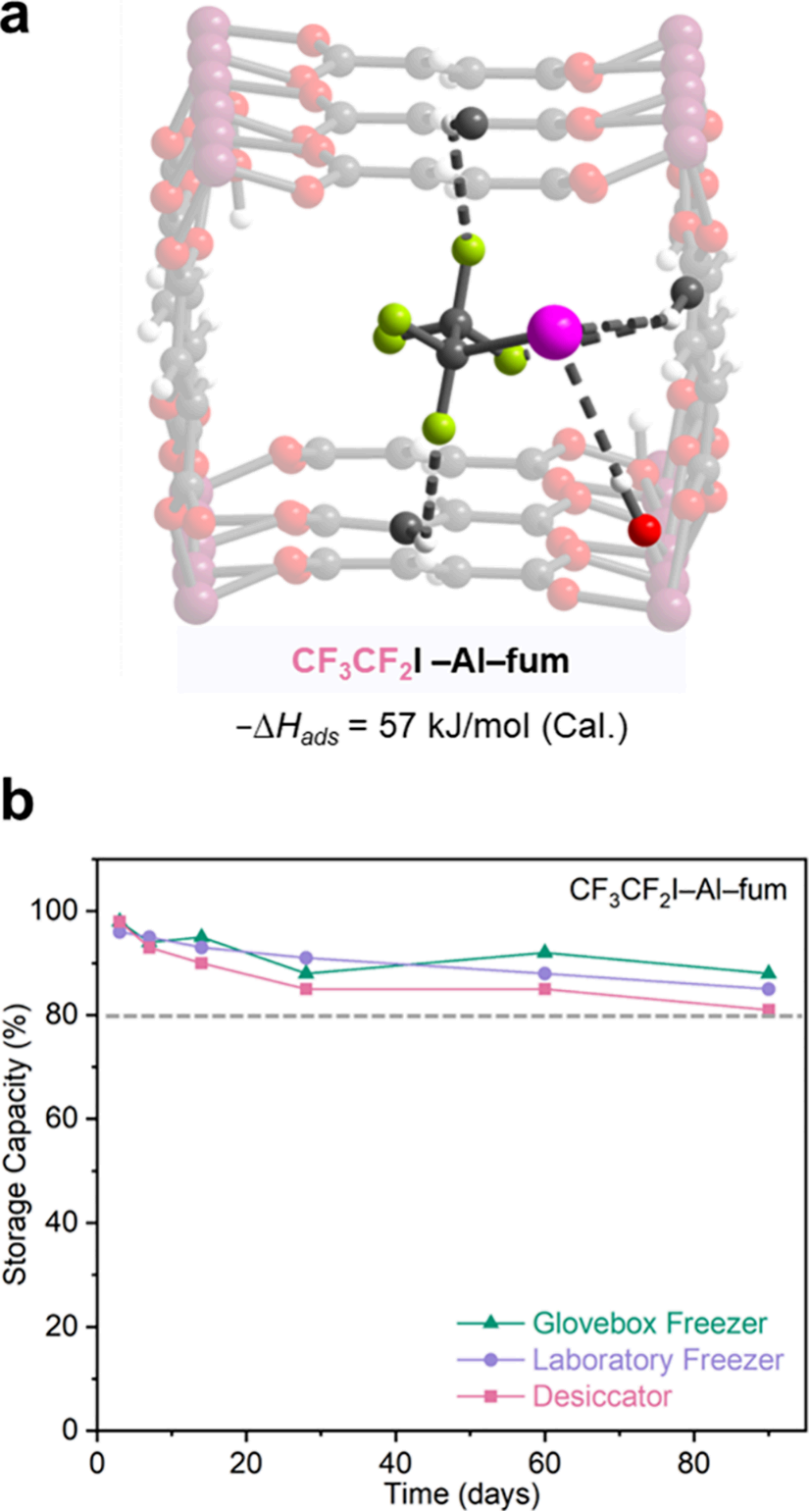
(a) DFT-optimized configuration of CF_3_CF_2_I bound in Al–fum, with calculated – Δ*H*_ads_ indicated. Gray, red, white, plum, green, and magenta spheres correspond to carbon, oxygen, hydrogen, aluminum, fluorine, and iodine, respectively. (b) Storage stability of CF_3_CF_2_I–Al–fum under different conditions. Gas-loaded samples (30–50 mg) were kept in a glovebox freezer (−30 °C) (green), a standard laboratory freezer (−25 °C) (purple), or a benchtop desiccator at r.t. (pink).

**Table 2:**
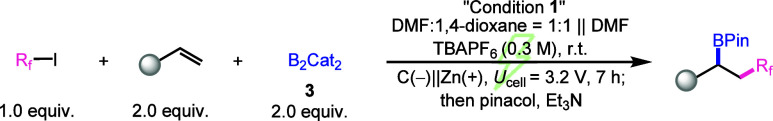

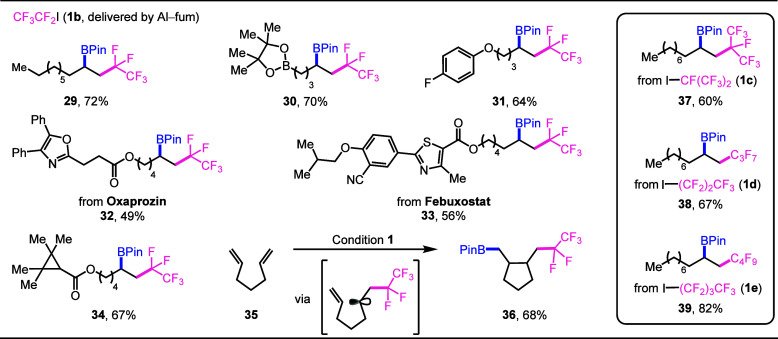
Scope of Electroreductive Radical Difunctionalization of Unactivated Olefins by Using CF_3_CF_2_I–Al–fum and Other Perfluoroalkyl Iodides^a^

a “Condition **1**”: cathodic chamber: R_f_–I (0.4 mmol, 1.0 equiv), alkene (2.0 equiv), **3** (2.0 equiv), TBAPF_6_ (0.3 M), DMF (1.0 mL), 1,4-dioxane (1.0 mL); anodic chamber: TBAPF_6_ (0.3 M), DMF (2.0 mL). After completion of the reaction, Et_3_N (4.0 equiv) and pinacol (4.0 equiv) were added to the cathodic chamber, and the mixture was stirred for another 1 h. Isolated yields are shown.

Introducing the CF_3_CF_2_ group remains synthetically challenging, yet it provides unique steric and physicochemical features that make it an attractive target for electroreductive difunctionalization.^2,6,48^ Under the same electroreductive conditions optimized for CF_3_I (Figure 2), CF_3_CF_2_I–Al–fum enabled the efficient CF_3_CF_2_-borylation of unactivated alkenes bearing boronate (**30**), fluorine (**31**), ester (**32**–**34**), nitrile (**33**), and cyclopropane (**34**) groups (Table 2). Drug-like substrates containing sensitive heterocycles also performed smoothly, delivering the desired products in excellent yields (**32**–**33**). Support for a radical pathway for this transformation was obtained using 1,6-heptadiene as a substrate (**35**), which furnished **36** in 68% yield via an intramolecular radical cyclization pathway. To the best of our knowledge, this work represents the first example of electroreductive CF_3_CF_2_-difunctionalization of alkenes, providing a versatile platform to structurally diverse molecules bearing this synthetically valuable motif.

Beyond gaseous perfluoroalkyl iodide (R_f_–I) reagents, several liquid R_f_–I reagents, including perfluoro-isopropyl (**1c**), perfluoro-*n*-propyl (**1d**), and perfluoro-*n*-butyl (**1e**) iodides, were also compatible with the optimized reaction conditions (Table 2, right). Notably, these transformations proceeded efficiently without the need for MOF-based gas delivery, indicating that this electroreductive platform is not restricted to gaseous fluoroalkyl iodides.

The CF_2_H group has emerged as a particularly attractive motif in medicinal chemistry due to its ability to serve as a lipophilic hydrogen-bond donor, modulate metabolic stability, and fine-tune physicochemical properties.^7,10,36,37^ Despite its importance, no electroreductive strategy has yet enabled the direct installation of CF_2_H groups onto unactivated alkenes. To probe whether our platform could overcome this limitation, we next turned our attention to CF_2_HI, a synthetically valuable yet electrochemically challenging reagent due to its more-negative redox potential (*E*_p/2_ = –2.05 V vs Fc/Fc^+^, SI Figure S33) compared to, for example, CF_3_I (−1.36 V vs Fc/Fc^+^, SI Figure S28). Prior studies, as well as our own observations, indicate that CF_2_H· is not compatible with electrophilic/radical boron reagents due to its nucleophilicity.^64,65^ Thus, we focused on accessing the corresponding CF_2_H-iodination product via an ATRA pathway (similar to CF_3_I, Figure 2b), as this would still provide a useful alkyl iodide handle for subsequent derivatization (Table 3). To achieve this, CF_2_HI–Al–fum was prepared using our previously reported strategy (SI Section 3).^51^

**Table 3:**
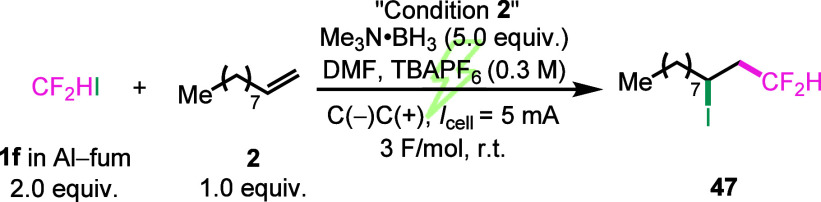
Optimization of the CF_2_H-Iodination of 1-Decene (**2**)­^a^

entry	variation from condition **2**	yield of **47** (%)^b^
1	none	52
2	no electricity	<5
3	DIPEA instead of Me_3_N·BH_3_	33
4	NaCIO_4_ instead of TBAPF_6_	16
5^c^	CF_2_Hl delivered by DMF (**RXN 2**)	49
6	no Me_3_N·BH_3_	31
7^d^	divided cell,C(−)||Zn(+), *U*_cell_ = 3.2 V	<5
8	set up on benchtop	50
9	recycled Al-fum	51

a “Condition **2**”: **1f** (in Al–fum, 0.8 mmol, 2.0 equiv), **2** (1.0 equiv), Me_3_N·BH_3_ (5.0 equiv) TBAPF_6_ (0.3 M) and DMF (4.0 mL).

b Yields obtained by ^19^F NMR (CF_3_Ph as internal standard).

c CF_2_HI delivered as a stock solution in DMF (0.55 M) instead of using CF_2_HI–Al–fum.

d Cathodic chamber: **1f** (0.8 mmol, 2.0 equiv), **2** (1.0 equiv), Me_3_N·BH_3_ (5.0 equiv), TBAPF_6_ (0.3 M), DMF (4.0 mL); anodic chamber: TBAPF_6_ (0.3 M), DMF (4.0 mL).

After optimization, the use of 2.0 equiv of CF_2_HI delivered from Al–fum, Me_3_N·BH_3_ as the sacrificial reductant,^66,67^ TBAPF_6_ as the electrolyte, DMF as the solvent, and carbon felt as both electrodes in an undivided cell provided the desired product **47** in 52% yield (Entry 1, Table 3). The requirement for excess CF_2_HI and a sacrificial reductant likely reflects the more negative reduction potential of CF_2_HI and the need to sustain efficient radical generation under these conditions. Control experiments confirm that electrical input is essential for product formation (Entry 2, Table 3). Substituting Me_3_N·BH_3_ with DIPEA diminished the yield (33%, Entry 3, Table 3), and omission of the sacrificial reductant still permitted product formation, albeit with reduced yield of 31% (Entry 6, Table 3). This result suggests that, in this system, an alternative anodic oxidation pathway involving a species generated *in situ* can sustain electrolysis in the absence of a sacrificial reductant. Consistently, the reaction does not proceed in a divided cell, suggesting that species generated at both electrodes are required for product formation. The CF_2_H-iodinated product **47** must not form readily via I-transfer from CF_2_HI itself, as otherwise the reaction should still proceed in a divided cell (as is the case for CF_3_I, Figure 2b). Indeed, reported gas-phase bond dissociation energies indicate that the C–I bond in CF_2_HI (≈58 kcal mol^–1^) is significantly stronger than in CF_3_I (≈54 kcal mol^–1^),^68^ making iodine atom transfer thermodynamically less favorable and disfavoring a classical ATRA-type radical chain pathway. Thus, we hypothesize instead that this paired electrolysis involves the generation of I_3_^–^ from I^–^ at the anode (*E*_p/2_ = +0.12 V vs Fc/Fc^+^, SI Figure S35),^69^ which enables ATRA to furnish the desired product (SI Figure S48). Consistently, UV–Vis monitoring under modified electrolysis conditions showed the formation of I_3_^–^ (λ_max_ ≈ 290 and 360 nm) only when current was applied (SI Figure S46). These results are consistent with I_3_^–^ generation at the anode and support its role as the iodine source in the ATRA product.

Similar to CF_3_I, delivering CF_2_HI using a stock solution in DMF resulted in a comparable yield (49%, Entry 5, Table 3), which indicates that the presence of Al–fum does not influence the reaction efficiency. To evaluate the practicality of the method, the reaction was also conducted on the benchtop under an N_2_ atmosphere; the yield was similar to that obtained in an N_2_ glovebox (50% vs 52%, Entry 8, Table 3). In addition, recycled Al–fum performed equivalently to the fresh material, providing **47** in 51% yield and demonstrating the robustness and reusability of the MOF delivery platform (Entry 9, Table 3). Together, these findings highlight that the presented method represents a sustainable and efficient means to install CF_2_H groups within organic scaffolds.

With the optimized reaction conditions in hand, we next explored the scope of the CF_2_H-iodination reaction (Table 4). A variety of unactivated alkenes bearing representative functional groups performed well, including organohalide (**40**, **42**, **43**), hydroxyl (**41**), sulfonate (**44**), phthalimide (**46**), and boronate ester (**48**) groups. The reaction displays exceptionally high chemoselectivity, as primary alkyl iodides (**42**) remain intact despite having reduction potentials that are only slightly lower in magnitude than those of the secondary alkyl iodide products and CF_2_HI itself.^70^ Furthermore, alkenes derived from drug molecules, such as ibuprofen (**49**) and indomethacin (**50**), were successfully difunctionalized, delivering the corresponding CF_2_H-iodinated products in good yields. Together, these results establish the CF_2_HI-based radical electroreductive platform as a powerful method for the late-stage installation of CF_2_H groups.

**Table 4:**
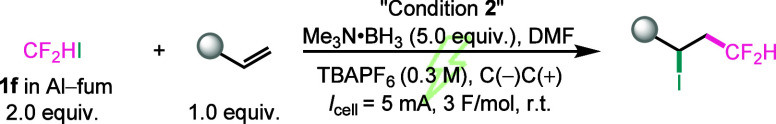

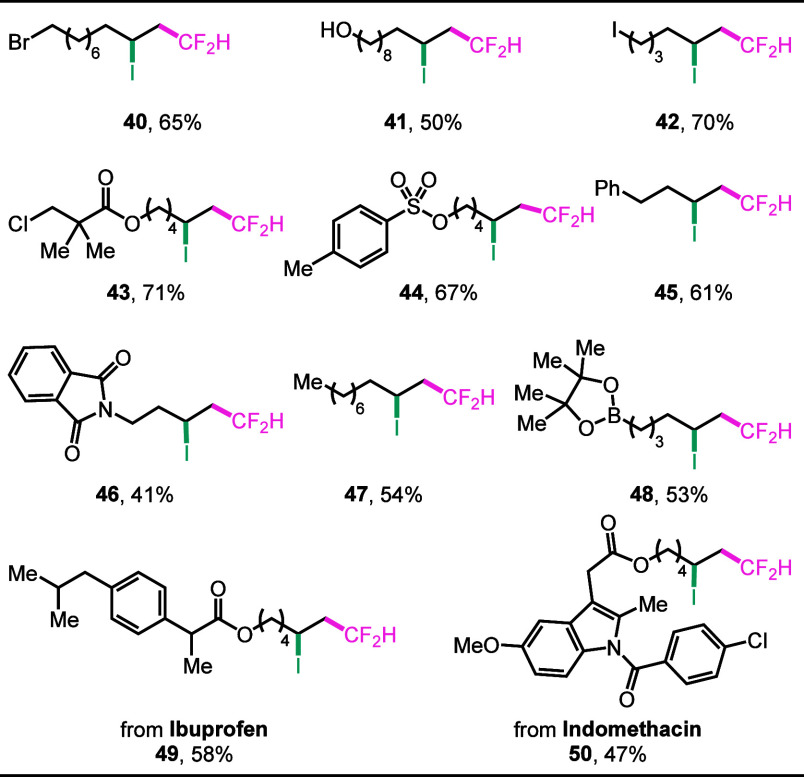
Scope of Electroreductive Radical Difunctionalization of Unactivated Olefins by Using CF_2_HI–Al–fum^a^

a “Condition **2**”: **1f** (in Al–fum, 0.8 mmol, 2.0 equiv), alkene (1.0 equiv), Me_3_N·BH_3_ (5.0 equiv), TBAPF_6_ (0.3 M), and DMF (4.0 mL). Isolated yields are shown.

We hypothesize that all transformations reported herein proceed via initial reduction of R_f_–I reagent at the cathode, which, after mesolytic cleavage, generates R_f_· and I^–^; the electrogenerated R_f_· then undergoes radical addition to the alkene to form a secondary radical, followed by subsequent borylation^56^ or iodination^71^ to furnish the respective products (see SI Section 14 for proposed mechanisms). The radical nature of these reactions is reinforced by the observation of cyclized product **36** from diene **35** (Table 2). To further support this general mechanistic paradigm, we conducted cyclic voltammetry (CV) studies (Figure 4a, SI Section 10). Notably, Al–fum itself does not display significant redox activity within the solvent window, indicating that it is electrochemically inert (SI Figure S36), consistent with its role as a delivery vehicle rather than a contributor to the reaction mechanism. In contrast, CF_3_I, C_2_F_5_I, *i*-C_3_F_7_I, *n*-C_3_F_7_I, and *n*-C_4_F_9_I all undergo irreversible reduction within the range of –1.3 to –1.7 V vs Fc/Fc^+^, consistent with facile electroreduction and subsequent generation of R_f_· (Figure 4a, SI Figures S28–S32). For *n*-C_4_F_9_I, although its reduction potential is more negative (*E*_p/2_ = 1.67 V vs FC/FC^+^), it affords a higher yield (82%) than the other fluoroalkyl iodides. We attribute this to the weaker C–I bond (≈ 49 kcal mol^–1^), which facilitates homolytic cleavage and promotes product formation.^68^ Addition of B_2_Cat_2_ has no observable effect on these reduction events, suggesting that radical generation occurs independently of the boron reagent in all cases (SI Figures S28–S32).

**Figure 4:**
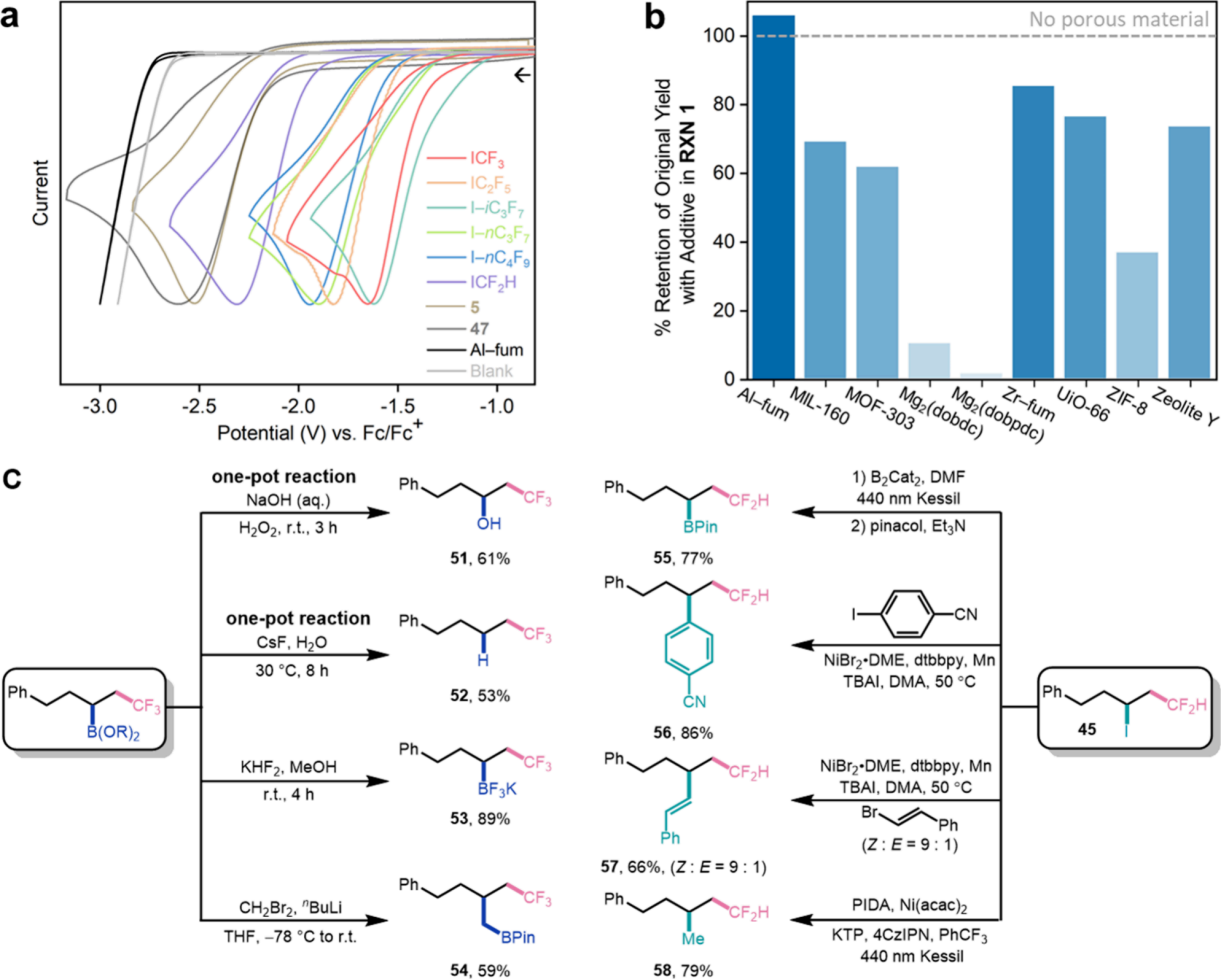
(a) CVs of fluoroalkyl iodides, products **5** and **47**, and Al–fum. 5 mM substrate concentration in DMF containing 0.1 M TBAPF_6_ as electrolyte under an N_2_ atmosphere. Working electrode: glassy carbon; counter electrode: platinum wire, reference electrode: Ag/Ag^+^ (0.01 M AgNO_3_ in 0.1 M TBAPF_6_/DMF). Scan rate: 100 mV/s. *E*_p/2_ of CF_3_I = – 1.36 V vs Fc/Fc^+^; *E*_p/2_ of C_2_F_5_I = – 1.61 V vs Fc/Fc^+^; *E*_p/2_ of *i*-C_3_F_7_I = – 1.29 V vs Fc/Fc^+^; *E*_p/2_ of *n*-C_3_F_7_I = – 1.59 V vs Fc/Fc^+^; *E*_p/2_ of *n*-C_4_F_9_I = – 1.67 V vs Fc/Fc^+^; *E*_p/2_ of CF_2_HI = – 2.05 V vs Fc/Fc^+^; *E*_p/2_ of **5** = – 2.23 V vs Fc/Fc^+^; *E*_p/2_ of **47** = – 2.30 V vs Fc/Fc^+^. (b) Evaluation of porous materials as additives in **RXN 1**. (c) Derivatization reactions from CF_3_-borylated (left) and CF_2_H-iodinated products (right).

To further confirm that the R_f_–I reagent is reduced during the reaction, we monitored the cathode potential of the CF_3_-borylation reaction using an internal reference electrode. Comparison of the average electrode potentials measured during electrolysis (−1.50 V vs Fc/Fc^+^, SI Figure S41) with the CV data supports that the cathodic process corresponds to reduction of CF_3_I (*E*_p_/_2_ = –1.36 V vs Fc/Fc^+^), generating CF_3_· for subsequent addition to the alkene substrate. The secondary alkyl iodide **5** (Figure 2) generated via CF_3_-iodination exhibits a substantially more negative reduction potential (−2.23 V vs Fc/Fc^+^, Figure 4a) compared to any of these R_f_–I reagents. This finding explains why this product can be formed selectively under the reaction conditions when using excess CF_3_I (Figure 2), with CF_3_I likely serving as the I source via an ATRA pathway.^72^ Nonetheless, kinetic analysis of the CF_3_-borylation reaction to produce **4** reveals that a small amount of **5** (<10%) forms during the reaction (SI Figures S37 and S38). We propose that any **5** generated in the presence of B_2_Cat_2_ can undergo a second electroreduction event at the cathode, followed by capture by B_2_Cat_2_.^73^ This hypothesis is supported by the observation that **5** converts to **4** under the standard reaction conditions, albeit slowly (see SI Section 11 for details). Overall, this analysis reveals that R_f_-borylation and R_f_-iodination are likely competing pathways under the reaction conditions, but any trace iodinated product formed *in situ* can be converted into the borylated product, leading to high selectivity for this product in the presence of B_2_Cat_2_ and limiting CF_3_I.

Compared to these perfluoroalkyl iodides, the partially fluorinated CF_2_HI requires a more negative potential for reduction (*E*_p/2_ = – 2.05 V, Figure 4a), yet the corresponding product **47** exhibits even lower propensity for reduction (*E*_p/2_ = – 2.30 V, Figure 4a), explaining the exceptional chemoselectivity for the desired CF_2_H-iodination product. We propose that cathodic reduction of CF_2_HI generates CF_2_H· along with I^–^. The CF_2_H radical then adds to the alkene to form an alkyl radical, while the I^–^ is oxidized at the anode to I_3_^–^, furnishing an I· donor that traps the alkyl radical to give the iodinated product. This mechanistic scenario is consistent with the observation that the reaction fails in a divided cell (Entry 7, Table 3) and still proceeds—albeit with reduced efficiency—in the absence of a sacrificial reductant (Entry 6, Table 3).

The lack of accessible redox features upon reduction of Al–fum (Figure 4a) explain why it is compatible with the electroreductive transformations developed herein. The superiority of Al–fum as a gas-delivery platform compared to other porous solids was validated using a robustness screen (Figure 4b; see SI Sections 2 and 16 for details). In each case, 100 mg of the material was added directly to electroreductive reactions using CF_3_I (**RXN 1**, Figure 2) and CF_2_HI (**RXN 2**, Table 3). In contrast to Al–fum (107% yield retention), all eight tested materials showed diminished yields for **RXN 1**. In particular, the redox-active salicylate MOFs Mg_2_(dobdc) (dobdc^4–^ = 2,5-dioxidobenzene-1,4-dicarboxylate) and Mg_2_(dobpdc) (dobpdc^4–^ = 3,3′-dioxido-1,1′-biphenyl-4,4′-dicarboxylate) led to significant inhibition.^74^ Zr-based frameworks, including Zr–fum and UiO-66 (UiO = Universitetet I Oslo), exhibited better compatibility (85% and 76% retention of yield). A similar trend was observed for **RXN 2** (SI Figure S50), with Al–fum uniquely retaining the yield of the model reaction. These results highlight the unique inertness of Al–fum under electrochemical conditions and underscore its potential suitability as a general carrier for gases in radical transformations.

Finally, to showcase the synthetic value of the difunctionalized products prepared using the methods developed herein, we explored a series of downstream transformations (Figure 4c). The *in situ*-generated CF_3_-borylated intermediate derived from but-3-en-1-ylbenzene could be converted directly in one pot to the hydroxylated product **51** (61% overall yield)^75^ or the corresponding reduced product **52** (53% overall yield),^76^ highlighting the compatibility of the crude product mixtures with sequential operations. The isolated alkylboronate **11** could also be smoothly functionalized: treatment with aqueous KHF_2_ delivered the trifluoroborate salt **53** in 89% yield, and homologation with CH_2_Br_2_/*n*-BuLi at –78 °C afforded **54** in 59% yield.^77^ The CF_2_H-iodinated product **45** similarly proved to be a versatile intermediate for subsequent functionalization. Under 440 nm light irradiation, borylation proceeded efficiently to furnish **55** in 77% yield,^56^ providing access to CF_2_H-borylated products that cannot be produced directly under purely electrochemical conditions. In addition, Ni-catalyzed radical cross-couplings of **45** enabled net CF_2_H-arylation, -vinylation, and -alkylation, delivering products **56**–**58** in good yields.^51,78^ Collectively, these examples demonstrate that the obtained difunctionalized products serve as highly versatile intermediates, offering access to a plethora of products that are challenging to access directly under electrochemical or photochemical conditions.

## Conclusion

In summary, we have developed a general electrochemical platform that enables the radical difunctionalization of alkenes with a broad range of gaseous R_f_–I reagents, including CF_3_I, CF_3_CF_2_I, and CF_2_HI. This work represents the first demonstration that gas–MOF reagents can be used to control product selectivity for organic electrosynthesis and the first electroreductive method to introduce CF_3_CF_2_ and CF_2_H groups across unactivated olefins. By leveraging Al–fum as a tunable gas carrier, we achieved precise delivery of gaseous R_f_–I reagents, enabling switchable formation of borylated and iodinated products. The method exhibits broad substrate generality and chemoselectivity even in the presence of redox-sensitive motifs and other alkyl halides. Notably, mechanistic studies confirm that the MOF acts as a redox-innocent gas reservoir rather than a catalytic or reactive component, enabling controlled delivery of gaseous reagents without directly participating in the reaction pathway. Together, these results establish gas–MOF-mediated electrochemistry as a powerful and general strategy for accessing valuable fluoroalkylated products, including those derived from bioactive alkene starting materials. While the solid-state handling of gaseous reagents enhances operational practicality, we recognize that the current use of sacrificial anodes and polar aprotic solvents limits the overall sustainability profile of these transformations. Future work will focus on expanding the lexicon of both fluoroalkyl gases and radical difunctionalization reactions that can be achieved under mild and sustainable electroreductive conditions.

## Supplementary Material



## Notes

Experimental procedures and analytical data for all compounds (PDF)
