# Supplementary material for: Electroreductive Radical Olefin Difunctionalization with Fluorinated Gases Enabled by Dosage Delivery from a Metal–Organic Framework
Source: J Am Chem Soc. 2026 Apr 22;148(17):17993–8003. doi: 10.1021/jacs.6c01583 (PMC13154208; doi:10.1021/jacs.6c01583)
Supplement: Supplementary file 1 [file ja6c01583_si_001.pdf]

Electroreductive Radical Olefin Difunctionalization with Fluorinated Gases Enabled by Dosage Delivery from a Metal-Organic Framework

Yihuan Lai[a], Jiachen He[a], Oliver P. Lambert[a], Joharimanitra Randrianandraina[b], Jung-Hoon Lee[b,c], Phillip J. Milner[a,*]

[a]Department of Chemistry and Chemical Biology, Cornell University, Ithaca, NY, 14853, United States
[b]Computational Science Research Center, Korea Institute of Science and Technology (KIST), Seoul 02792, Republic of Korea
[c]KU-KIST Graduate School of Converging Science and Technology, Korea University, Seoul 02841, Republic of Korea

*pjm347@cornell.edu

Supporting Information

Table of Contents

1. **General Information.** 3
2. **Synthesis and Characterization of Porous Materials.** 4
3. **General Procedures for Preparing Gas–MOFs.** 17
4. **Solvent-Independent Delivery by using Gas–Al–fum** 22
5. **Gas Release Analysis of Gas–MOF Reagents.** 23
6. **Density Functional Theory (DFT) Calculated Structure of CF_3CF_2I–Al–fum.** 24
7. **Synthesis of Starting Materials.** 26
8. **General Electrolysis Procedures.** 28
9. **Reaction Optimization.** 32
10. **CV Studies.** 38
11. **Kinetic Studies** 46
12. **Three-Electrode Measurement to Determine Working Electrode Potentials** 49
13. **UV–Vis Analysis of Anodic Iodide Oxidation.** 55
14. **Proposed Mechanisms.** 56
15. **Substrates with Diminished Reactivity in CF_2H–Iodination** 58
16. **Porous Materials as Additives in Reactions** 59

17. Procedures for Derivation Reactions. 65
18. Preparation and Characterization of Products. 73
19. Copies of NMR Spectra. 113
20. References. 220

1. General Information.

Unless otherwise noted, all chemicals were purchased from commercial vendors (Sigma Aldrich, Matrix Chemical, Ambeed, Alfa Aesar, Oakwood Chemical, or TCI) and used without additional purification unless noted otherwise. Anhydrous *N,N*-dimethylformamide (DMF) was obtained by vigorously sparging with Ar for 30 min, followed by passage through two columns of activated alumina using a Phoenix SDS JC Meyer Solvent System. The solvent was stored over activated 4 Å molecular sieves in a N_2-filled glovebox when not in use. Carbon felt was purchased from Fuel Cell Store.

Electrolysis experiments were performed using a Gamry Interface 1010E potentiostat or a Tacklife DC power supply with carbon felt electrodes. To prepare the electrodes, a septum was pierced with a needle, and a 2B pencil lead (2 mm in diameter) was inserted through the hole. The carbon felt was cut into a small (4 mm × 4 mm) square and impaled onto the pencil lead. Ag/$AgNO_3$ reference electrodes were obtained from CH Instruments and stored in an acetonitrile solution with 0.01 M $AgNO_3$ and 0.1 M $TBAClO_4$ when not in use. Cyclic voltammograms (CVs) were measured at 22 ^{o}C on a Gamry Interface 1010E potentiostat using a glassy carbon working electrode, a platinum wire counter electrode, and a Ag/$AgNO_3$ reference electrode in a N_2-filled glovebox. In general, two scans were collected: one with ferrocene (Fc) as an internal reference, and one without Fc.

^{1}H NMR, ^{13}C NMR, ^{19}F NMR and ^{11}B NMR spectra data were collected on a Bruker INOVA 400 MHz or a Bruker INOVA 500 MHz spectrometer and are referenced to residual solvent. Chemical shifts are reported in ppm with reference to solvent signals [^{1}H-NMR: $CDCl_3$ (7.26 ppm); ^{13}C-NMR: $CDCl_3$ (77.16 ppm)]. Signal patterns are indicated as s, singlet; d, doublet; t, triplet; q, quartet; and m, multiplet. High-resolution mass spectrometry (MS) data were obtained on a Thermo Fisher Scientific Exactive series DART Mass Spectrometer. Reactions were monitored by thin-layer chromatography (TLC) carried out on commercial silica gel plates using UV light as a visualizing agent. Powder X-ray diffraction (PXRD) patterns were collected on a Rigaku Ultima IV diffractometer or Bruker D8 Advance ECO powder diffractometer equipped with Cu Kα sources (λ = 1.5406 Å). Langmuir surface areas were determined by linear least squares regression analysis using the linearized form of the Langmuir equation. Langmuir surface area measurements were chosen for MOF characterization for expediency because all materials prepared herein have been previously reported.

2. Synthesis and Characterization of Porous Materials.

Al(OH)(fum) or Al–fum. Al–fum was prepared following a modified literature procedure.[1] A 500 mL glass beaker equipped with a stir bar was charged with $Al_2(SO_4)_3\cdot 18H_2O$ (35.0 g, 52.5 mmol, 1.0 equiv.) and H_2O (150 mL) at 60 °C. In a separate beaker, fumaric acid (H_2fum, 12.1 g, 105.0 mmol, 2.0 equiv.) and NaOH (12.6 g, 315.0 mmol, 6.0 equiv.) were dissolved in H_2O (180 mL) at 60 °C, and the resulting solution was added to the Al-containing mixture. The combined solution was stirred for an additional 2 h at 60 °C. The heterogeneous suspension was then cooled to room temperature and filtered. The solid product was rinsed extensively with H_2O (10 × 400 mL). The collected solid was transferred to a Schlenk flask and activated under high vacuum (<100 mTorr) at 150 °C for 24 h. The flask was subsequently transferred into an N_2-filled glovebox, affording activated Al–fum as a white solid (15.3 g, 92%).

A portion of the product (~100 mg) was loaded into a glass adsorption tube equipped with a Micromeritics *TransSeal* and further activated under high vacuum (<10 μbar) at 150 °C for 24 h. Langmuir surface area determined from the 77 K N_2 adsorption isotherm: 1746 ± 193 m^2/g (Lit. 1462 m^2/g).[1]

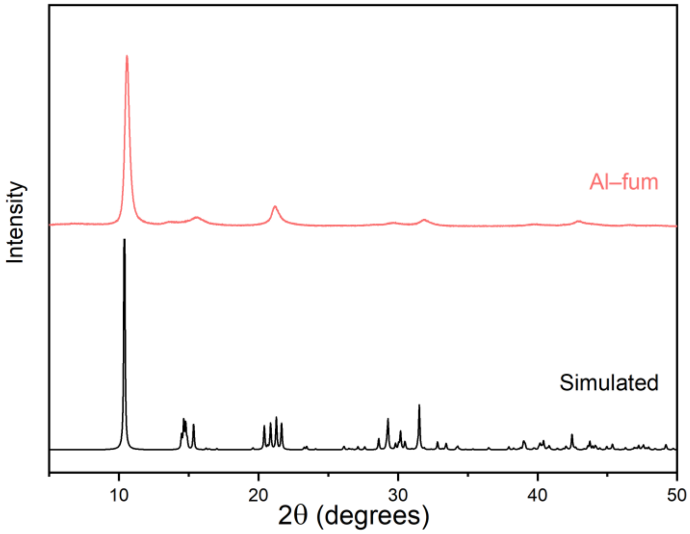

Figure S1. PXRD pattern ($\lambda = 1.5406$ Å) of Al–fum. The simulated pattern based on the previously reported PXRD structure of Al–fum is included for reference.[2] The broadness of the reflections is due to the modest crystallinity of Al–fum.

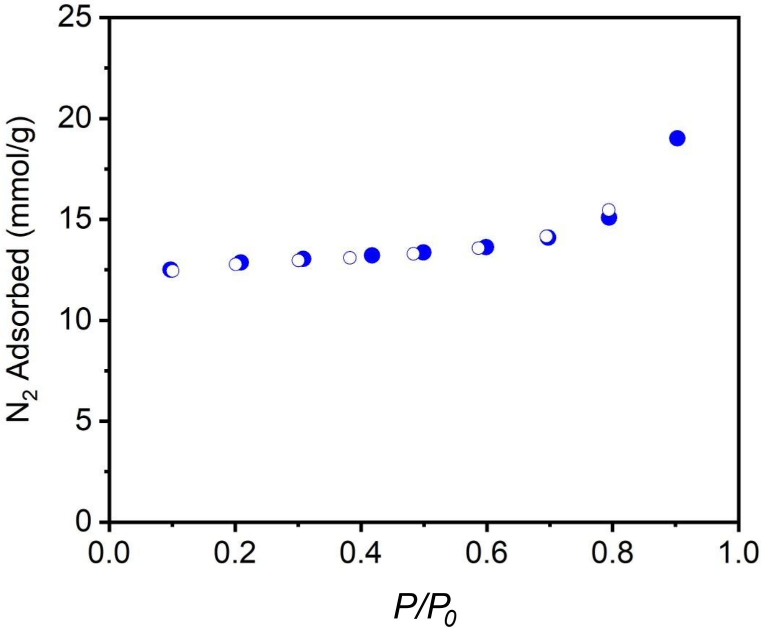

Figure S2. N_2 adsorption (solid circles) and desorption (open circles) isotherms at 77 K of activated Al–fum. The Langmuir surface area was determined to be 1746 ± 193 m^2/g (Lit. 1462 m^2/g).[1]

Mg_2(dobdc) or MOF-74 (Mg). Mg_2(dobdc) was prepared following a modified literature procedure.[3] NaOH (1.6 g, 4.0 mmol, 4.0 equiv.) was dissolved in H_2O (5 mL) in a 25 mL round-bottom flask. 2,5-hydroxybenzene-1,4-dicarboxylic acid (H_4dobdc, 2.0 g, 1.0 mmol, 1.0 equiv.) was added to give a dark heterogeneous mixture. In a separate beaker, $Mg(NO_3)_2 \cdot 6H_2O$ (6.4 g, 25.0 mmol, 2.5 equiv.) was dissolved in H_2O (5 mL), and the solution was transferred to the reaction flask, producing a green suspension. The mixture was stirred at ambient temperature for 1 h and then filtered. The solid was transferred to a 100 mL Pyrex jar containing MeOH (20 mL) and heated at 60 °C for 24 h. The suspension was cooled to room temperature, and the solvent was decanted and replaced with fresh MeOH (20 mL). This solvent exchange procedure was repeated twenty times. The solid was collected by filtration and transferred to a Schlenk flask. Activation was performed under flowing N_2 at room temperature for 6 h, followed by high vacuum (<100 mTorr) with a gradual temperature ramp (0.2 °C/min) to 300 °C in a sand bath. The sample was held at 300 °C under high vacuum for 24 h and then transferred into an N_2-filled glovebox to afford activated Mg_2(dobdc) (1.9 g, 88% yield).

A portion of the product (~100 mg) was loaded into a glass adsorption tube equipped with a Micromeritics *TransSeal* and further activated under high vacuum (<10 μbar) at 150 °C for 24 h. Langmuir surface area determined from the 77 K N_2 adsorption isotherm: 1870 ± 86 m^2/g (Lit. 1957 m^2/g).[3]

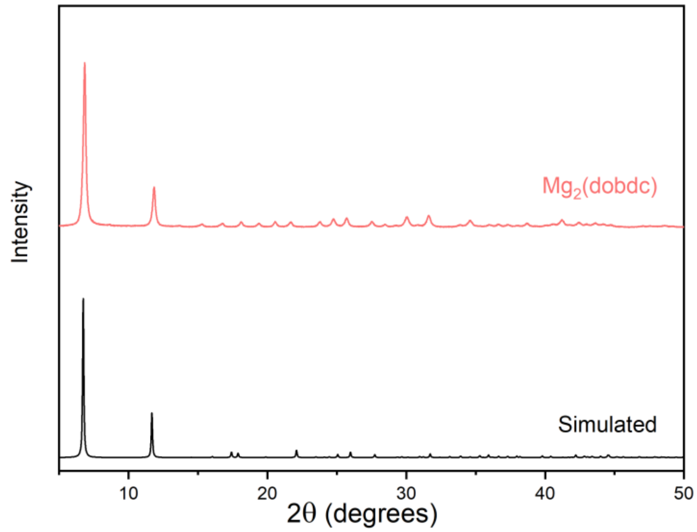

Figure S3. PXRD pattern (λ = 1.5406 Å) of Mg_2(dobdc). The simulated pattern based on the previously reported single-crystal X-ray diffraction (SCXRD) structure of Zn_2(dobdc) is included for reference.[4]

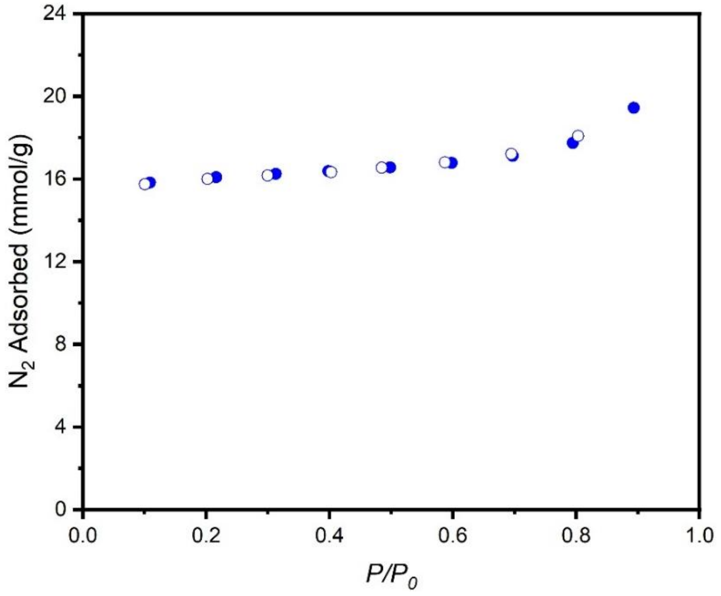

Figure S4. N_2 adsorption (solid circles) and desorption (open circles) isotherms of activated Mg_2(dobdc) at 77 K. The Langmuir surface area was determined to be 1870 ± 86 m^2/g (Lit. 1957 m^2/g).[3]

Mg_2(dobpdc). Mg_2(dobpdc) was prepared following a modified literature procedure.[5] In a 20 mL scintillation vial, $Mg(NO_3)_2 \cdot 6H_2O$ (321 mg, 1.25 mmol, 2.5 equiv.) was dissolved in H_2O (2.5 mL). In a separate 20 mL scintillation vial equipped with a stir bar, NaOH (80.0 mg, 2.0 mmol, 4.0 equiv.), 4,4′-dihydoxy-1,1′-biphenyl-3,3′-dicarboxylic acid (H_4dobpdc 137 mg, 0.5 mmol, 1.0 equiv.), and H_2O (2.5 mL) were combined. The $Mg(NO_3)_2$ solution was added in one portion to the H_4dobpdc solution via a plastic pipette, and the mixture was stirred at room temperature for 1 h. The resulting suspension was filtered under vacuum, and the solid was rinsed with MeOH (15 mL). The crude yellow-white solid was transferred to a clean scintillation vial containing MeOH (15 mL) and heated at 60 °C in an aluminum block for 12 h. After cooling to room temperature, the solvent was decanted and replaced with fresh MeOH (15 mL). This solvent-exchange process was repeated five times (six MeOH soaks total). After the final soak, the solid was collected by filtration, rinsing with acetone (100 mL), air-dried, and transferred to a Schlenk flask. Activation was performed under high vacuum (<100 mbar) at 120 °C for 24 h, after which the flask was brought into an N_2-filled glovebox to afford activated Mg_2(dobpdc) as a white solid (2.03 g, 49% yield).

A portion of the product (~100 mg) was loaded into a glass adsorption tube equipped with a Micromeritics *TransSeal* and further activated under high vacuum (<10 μbar) at 150 °C for 24 h. Langmuir surface area determined from the 77 K N_2 adsorption isotherm: 3533 ± 199 m^2/g (Lit. 3710 m^2/g).[5]

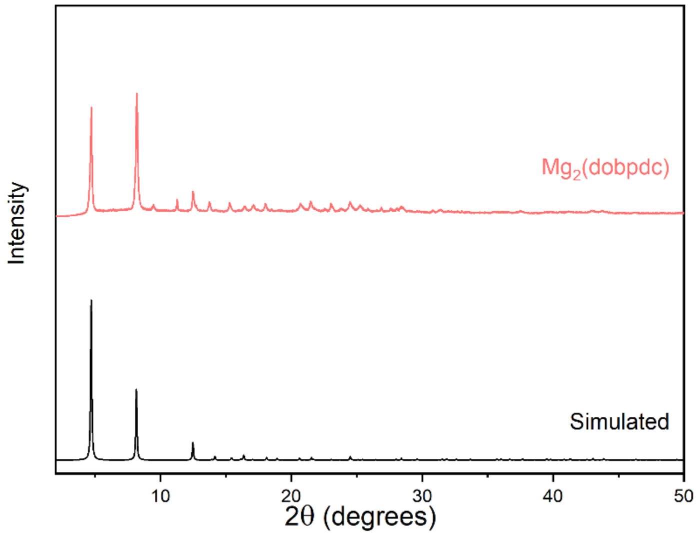

Figure S5. PXRD pattern (λ = 1.5406 Å) of Mg_2(dobpdc). The predicted pattern from the SCXRD structure of the isostructural framework Zn_2(dobpdc) is included for reference.[6]

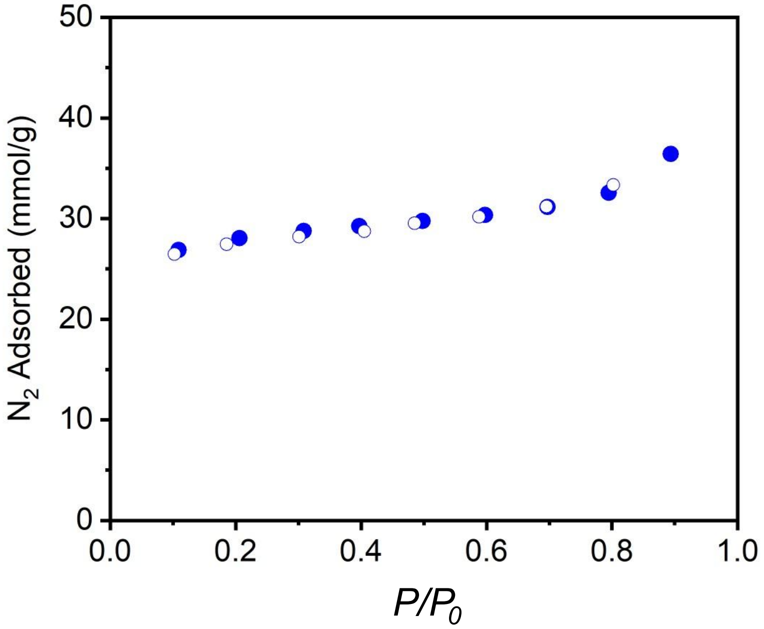

Figure S6. N_2 adsorption (solid circles) and desorption (open circles) isotherms of activated Mg_2(dobpdc) at 77 K. The Langmuir surface area was determined to be 3533 ± 199 m^2/g (Lit. 3710 m^2/g).[5]

Zeolite Y, Sodium. Zeolite Y (sodium form) was obtained from Alfa Aesar and used without further purification. The material was transferred to a Schlenk flask and activated under high vacuum (<100 mTorr) at 180 °C for 24 h. The flask was then brought into an N_2-filled glovebox, and the solid was loaded into a glass adsorption tube fitted with a Micromeritics *TransSeal*. The tube was removed from the glovebox and further activated under high vacuum (<10 μbar) at 180 °C for 24 h. The Langmuir surface area determined from the 77 K N_2 adsorption isotherm was 909 ± 2 m^2/g (900 m^2/g, Alfa Aesar).

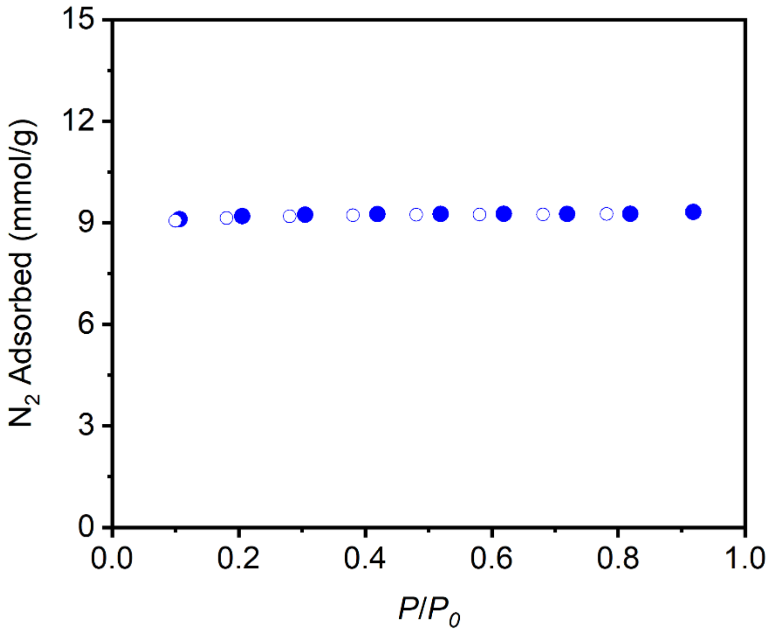

Figure S7. N_2 adsorption (closed circle) and desorption (open circle) isotherms of activated Zeolite Y, Sodium at 77 K. The Langmuir surface area of this material was determined to be 909 ± 2 m^2/g (Alfa Aesar: 900 m^2/g).

Zn(2-MIm) or ZIF-8. ZIF-8 was prepared following a modified literature procedure.[5] $Zn(NO_3)_2 \cdot 6H_2O$ (297.5 mg, 1.0 mmol, 1.0 equiv.) in H_2O (20 mL) was added to a solution of 2-methylimidazole (2-MIm, 335.0 mg, 1.0 mmol, 1.0 equiv.) in H_2O (20 mL) in a 100 mL round-bottom flask. The mixture became milky immediately after mixing. After stirring for 10 min, the solid product was collected by centrifugation (4000 rpm, 30 min) and rinsed with H_2O (5 × 50 mL). The sample was held at 150 °C under high vacuum for 24 h and then transferred into an N_2-filled glovebox to afford activated ZIF-8 (460.5 mg, 77% yield) as a white solid.

A portion of the product (~100 mg) was loaded into a glass adsorption tube equipped with a Micromeritics *TransSeal* and further activated under high vacuum (<10 μbar) at 150 °C for 24 h. Langmuir surface area determined from the 77 K N_2 adsorption isotherm: 1829 ± 6 m^2/g (Lit. 1609 m^2/g).[5]

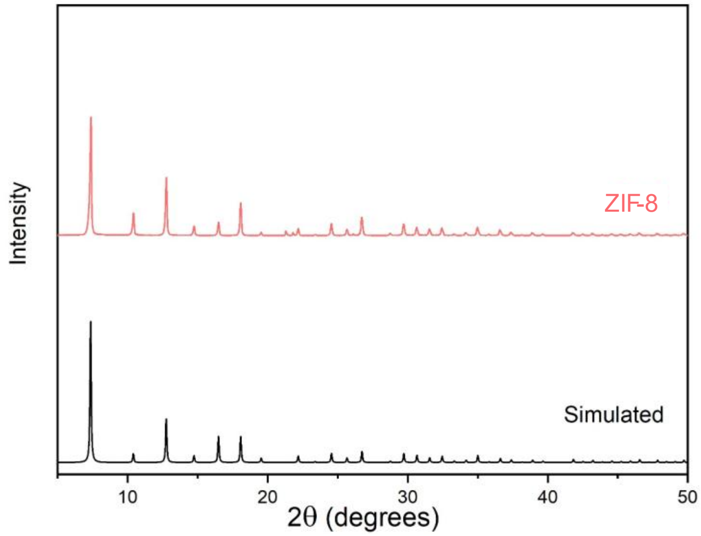

Figure S8. PXRD pattern (λ = 1.5406 Å) of ZIF-8. The simulated pattern based on the previously reported SCXRD structure of ZIF-8 is included for reference.[7]

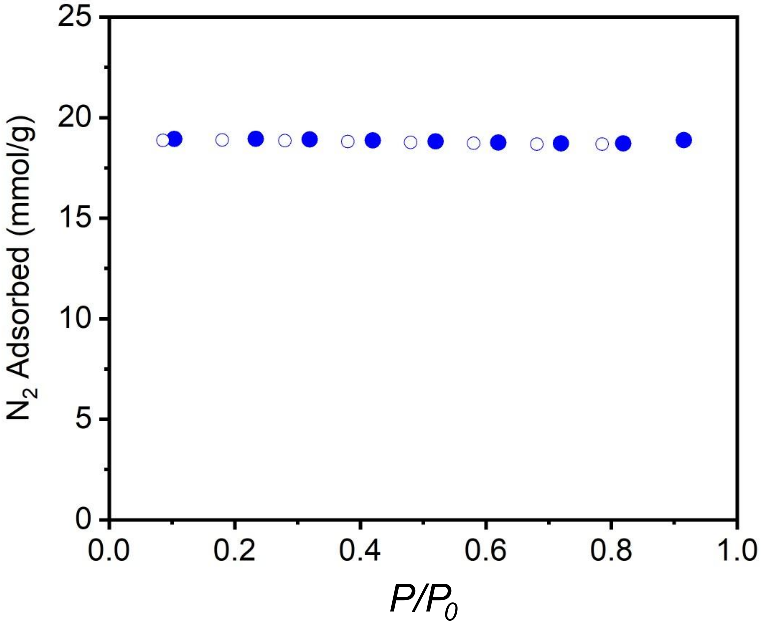

Figure S9. N_2 adsorption (closed circle) and desorption (open circle) isotherms of activated ZIF-8 at 77 K. The Langmuir surface area of this material was determined to be 1829 ± 6 m^2/g (Lit. 1609 m^2/g).[5]

$Zr_6(OH)_4O_4(fum)_6$, Zr–fum, or MOF-801. Zr–fum was prepared following a modified literature procedure.[8] A 100 mL round-bottom flask equipped with a stir bar and reflux condenser was charged with $ZrOCl_2 \cdot 8H_2O$ (6.5 g, 20.0 mmol, 1.0 equiv.), H_2fum (2.3 g, 20.0 mmol, 1.0 equiv.), formic acid (23 mL, 0.60 mol, 30 equiv.), and H_2O (80 mL). The mixture was heated at 95 °C in an oil bath for 24 h. After cooling to room temperature, the suspension was centrifuged (4000 rpm, 10 min), and the supernatant was decanted. The resulting solid was transferred to a 250 mL Pyrex jar containing EtOH (100 mL) and soaked for 24 h at room temperature. The solid was collected by centrifugation, the solvent decanted, and the soaking procedure was repeated twice more (three EtOH soaks in total). The same procedure was then performed with acetone (100 mL per soak, three soaks total, each 24 h). After the final acetone soak, the material was centrifuged, decanted, and air-dried. The solid was transferred to a Schlenk flask and activated under high vacuum (<100 mbar) at 100 °C for 24 h, then brought into an N_2-filled glovebox to afford activated Zr–fum as a white solid (3.5 g, 78% yield).

A portion of the product (~100 mg) was loaded into a glass adsorption tube equipped with a Micromeritics *TransSeal* and further activated under high vacuum (<10 µbar) at 150 °C for 24 h. Langmuir surface area determined from the 77 K N_2 adsorption isotherm: 1078 ± 31 m^2/g (Lit. 1070 m^2/g).[8]

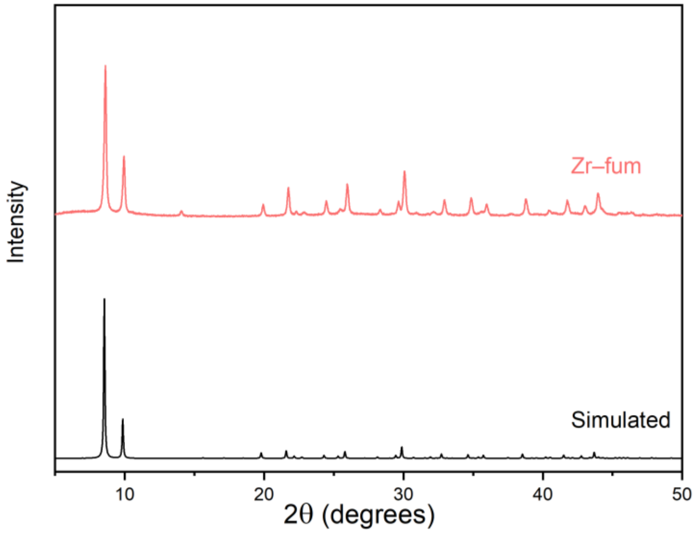

Figure S10. PXRD pattern (λ = 1.5406 Å) of Zr–fum. The simulated pattern based on the previously reported SCXRD structure of Zr–fum is included for reference.[8]

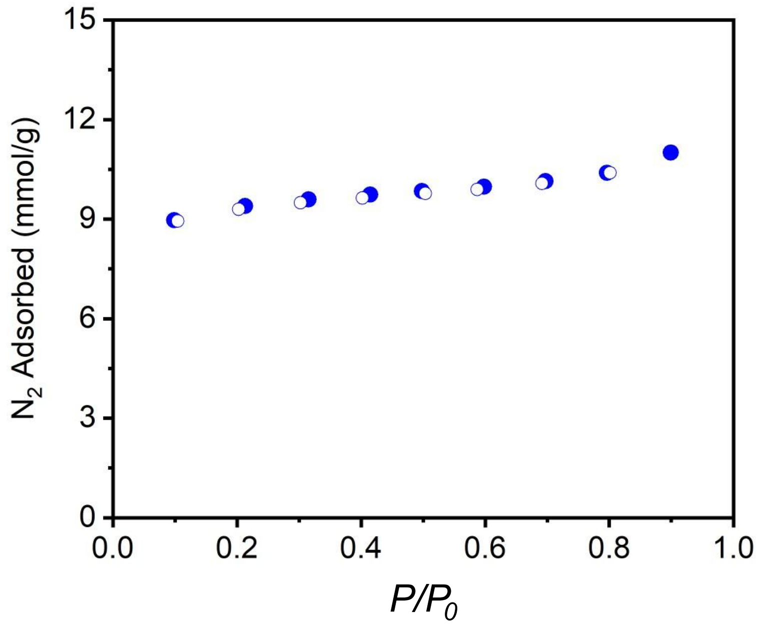

Figure S11. N_2 adsorption (closed circle) and desorption (open circle) isotherms of activated Zr–fum at 77 K. The Langmuir surface area of this material was determined to be 1078 ± 31 m^2/g (Lit. 1070 m^2/g).[8]

$Zr_6(OH)_4O_4(bdc)_6$ or UiO-66. UiO-66 was prepared following a modified literature procedure.[5] A 350 mL screw-cap high-pressure flask equipped with a stir bar was charged with $ZrCl_4$ (1.75 g, 7.5 mmol, 1.0 equiv.), terephthalic acid (H_2bdc, 1.23 g, 7.5 mmol, 1.0 equiv.), DMF (150 mL), and H_2O (0.41 mL, 22.5 mmol, 3.0 equiv.). The mixture was sonicated for 5 min, sealed, and heated at 120 °C in a silicone oil bath with gentle stirring (300 rpm) for 24 h. After cooling to room temperature, the solid was isolated by centrifugation (4000 rpm, 5 min), and the supernatant was decanted. The solid was transferred to a 250 mL Pyrex jar containing fresh DMF (150 mL) and soaked at room temperature for 24 h. This DMF soaking procedure was repeated two additional times (three soaks total). The same procedure was then carried out with MeOH (150 mL per soak, three soaks total). After the final soak, the solid was collected by centrifugation, air-dried, and transferred to a Schlenk flask. Activation was performed under high vacuum (<100 mbar) at 150 °C for 24 h. The flask was transferred into an N_2-filled glovebox to afford UiO-66 as a white solid (1.55 g, 74% yield).

A portion of the product (~100 mg) was loaded into a glass adsorption tube equipped with a Micromeritics *TransSeal* and further activated under high vacuum (<10 μbar) at 150 °C for 24 h. Langmuir surface area determined from the 77 K N_2 adsorption isotherm: 1300 ± 3 m^2/g (Lit. 1615 m^2/g).[5]

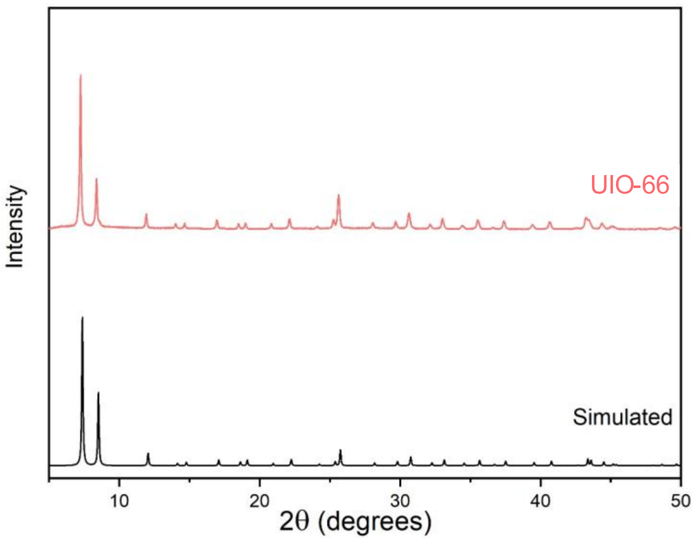

Figure S12. PXRD pattern (λ = 1.5406 Å) of UiO-66. The simulated pattern based on the previously reported SCXRD structure of UiO-66 is included for reference.[9]

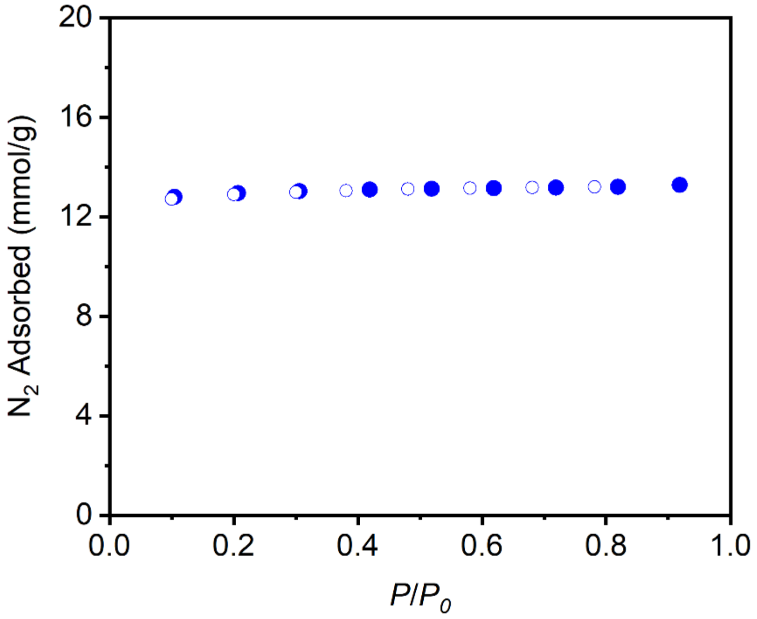

Figure S13. N_2 adsorption (closed circle) and desorption (open circle) isotherms of activated UiO-66 at 77 K. The Langmuir surface area of this material was determined to be 1300 ± 3 m²/g (Lit. 1615 m^2/g).[5]

Al(OH)(fdc) or MIL-160. MIL-160 was prepared following a modified literature procedure.[10] A 50 mL round-bottom flask equipped with a stir bar was charged with NaOH (80.0 mg, 2.0 mmol, 2.0 equiv.), furan-2,5-dicarboxylic acid (H_2fdc, 0.15 g, 1.0 mmol, 1.0 equiv.), $AlCl_3{\cdot}6H_2O$ (0.24 g, 1.0 mmol, 1.0 equiv.), and H_2O (15 mL). The mixture was stirred at reflux for 12 h. After cooling to room temperature, the resulting solid was collected by filtration and rinsed thoroughly with DMF (50 mL) followed by H_2O (100 mL). The material was then transferred to a clean vessel and soaked in acetone (20 mL), with the solvent replaced every 24 h (three acetone soaks total). After the final soak, the solid was collected by filtration, air-dried, and transferred to a Schlenk flask. Activation was performed under high vacuum (<100 mbar) at 120 °C for 24 h, after which the flask was brought into an N_2-filled glovebox to afford activated MIL-160 as a white solid (0.31 g, 79% yield).

A portion of the product (~100 mg) was loaded into a glass adsorption tube equipped with a Micromeritics *TransSeal* and further activated under high vacuum (<10 μbar) at 150 °C for 24 h. Langmuir surface area determined from the 77 K N_2 adsorption isotherm: 1142 ± 0 m^2/g (Lit. 1000 m^2/g).[10]

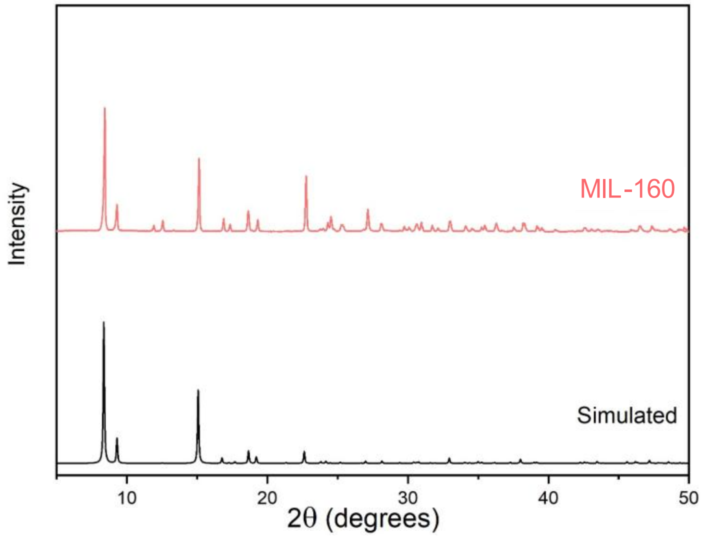

Figure S14. PXRD pattern (λ = 1.5406 Å) of MIL-160. The simulated pattern based on the previously reported PXRD structure of MIL-160 is included for reference.[10]

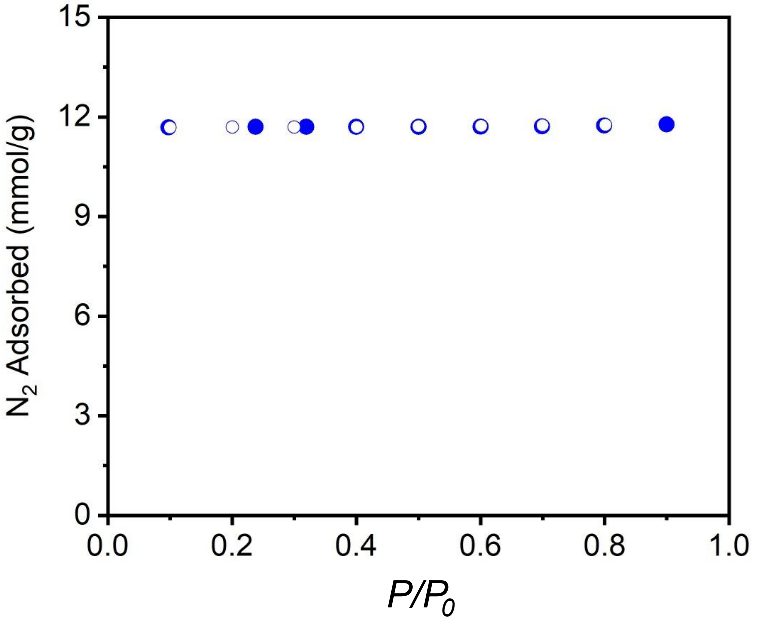

Figure S15. N_2 adsorption (closed circle) and desorption (open circle) isotherms of activated MIL–160 at 77 K. The Langmuir surface area of this material was determined to be 1142 ± 0 m^2/g (Lit. 1000 m^2/g).[10]

Al(OH)(PZDC) or MOF-303. MOF-303 was prepared following a modified literature procedure.[11] NaOH (0.60 g, 15 mmol, 1.5 equiv.) and 1*H*-pyrazole-3,5-dicarboxylic acid hydrate ($H_2PZDC \cdot H_2O$, 1.74 g, 10 mmol, 1.0 equiv.) were added to a 250 mL screw-cap high-pressure flask equipped with a stir bar, followed by H_2O (90 mL). The mixture was stirred until homogeneous. In a separate vial, $AlCl_3 \cdot 6H_2O$ (2.41 g, 10 mmol, 1.0 equiv.) was dissolved in deionized H_2O (10 mL). The $AlCl_3 \cdot 6H_2O$ solution was added dropwise to the reaction mixture through an addition funnel with stirring (100 rpm). A white precipitate formed during the addition, and the mixture became a milky suspension. After removing the stir bar, the reaction mixture was transferred to a 500 mL Pyrex jar. The jar was sealed and heated in oven at 120 °C for 24 h. After cooling to room temperature, the solid product was collected by centrifugation (4000 rpm, 10 min), and the supernatant was decanted. The solid was collected by filtration, rinsed with acetone (100 mL), air-dried, and transferred to a Schlenk flask. Activation was performed under high vacuum (<100 mbar) at 120 °C for 24 h, after which the flask was brought into an N_2-filled glovebox to afford activated MOF-303 as a white solid (2.03 g, 49% yield).

A portion of the product (~100 mg) was loaded into a glass adsorption tube equipped with a Micromeritics *TransSeal* and further activated under high vacuum (<10 μbar) at 150 °C for 24 h. Langmuir surface area determined from the 77 K N_2 adsorption isotherm: 1603 ± 2 m^2/g (Lit. 1342 m^2/g).[11]

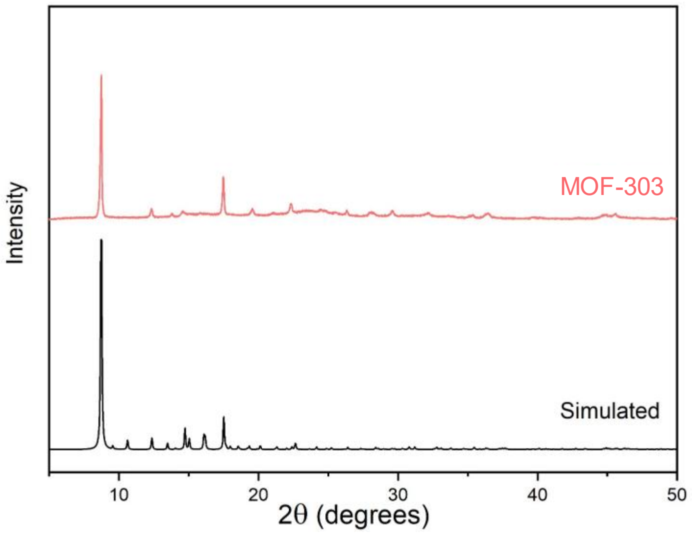

Figure S16. PXRD pattern (λ = 1.5406 Å) of MOF-303. The simulated pattern based on the previously reported PXRD structure of MOF-303 is included for reference.[11]

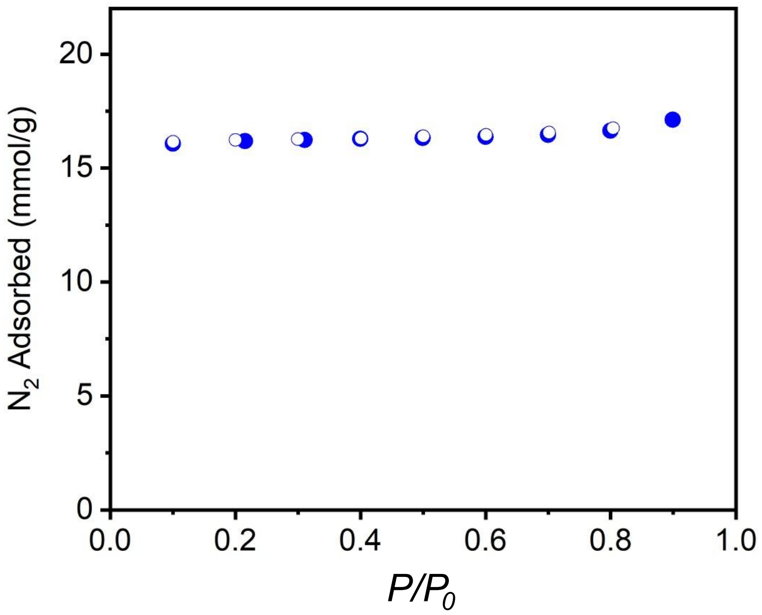

Figure S17. N_2 adsorption (closed circle) and desorption (open circle) isotherms of activated MOF-303 at 77 K. The Langmuir surface area of this material was determined to be 1603 ± 2 m^2/g (Lit. 1342 m^2/g).[11]

3. General Procedures for Preparing Gas–MOFs.

Dosing Gas with Balloon for Preparing CF_3I–Al–fum and CF_3CF_2I–Al–fum. Inside a N_2-filled glovebox, activated Al–fum was weighed and loaded into an oven-dried 20 mL screw-cap vial equipped with a septum. The vial was sealed with PTFE tape, removed from the glovebox, and cooled to −78 °C using an acetone/dry ice bath. A gas balloon (CF_3I or CF_3CF_2I) was then attached via needle (Figure S18), and the Al–fum was dosed with the fluorinated gas at −78 °C for 1 h. A noticeable decrease in the balloon volume was observed (Figure S18, right). After dosing, the vial was warmed to 0 °C and promptly capped.

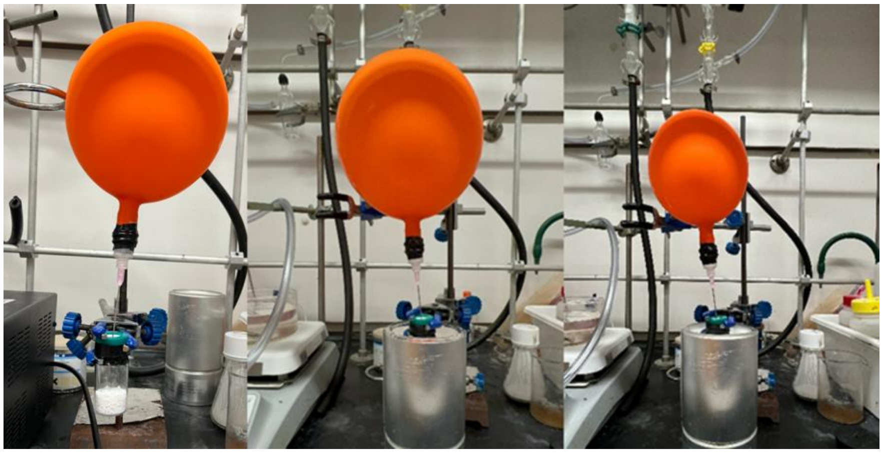

Figure S18. Set-up of dosing gas with a balloon.

The gas delivery was quantified by ^{19}F NMR ($PhCF_3$ as internal standard). Freshly prepared gas–Al–fum (20.0–30.0 mg) was weighed into an 8 mL vial. THF (1 mL) was then added, and the mixture was allowed to stand for 5 minutes before adding $PhCF_3$ (12.0 µL, 0.10 mmol). Based on ^{19}F NMR analysis, freshly prepared CF_3I–Al–fum typically contains 42 wt% CF_3I, while CF_3CF_2I–Al–fum contains 67 wt% CF_3CF_2I.

CF_3I–Al–fum:

^{19}F NMR (376 MHz, THF): δ −10.36 (s) ppm. Spectral data is consistent with those reported in literature.[1]

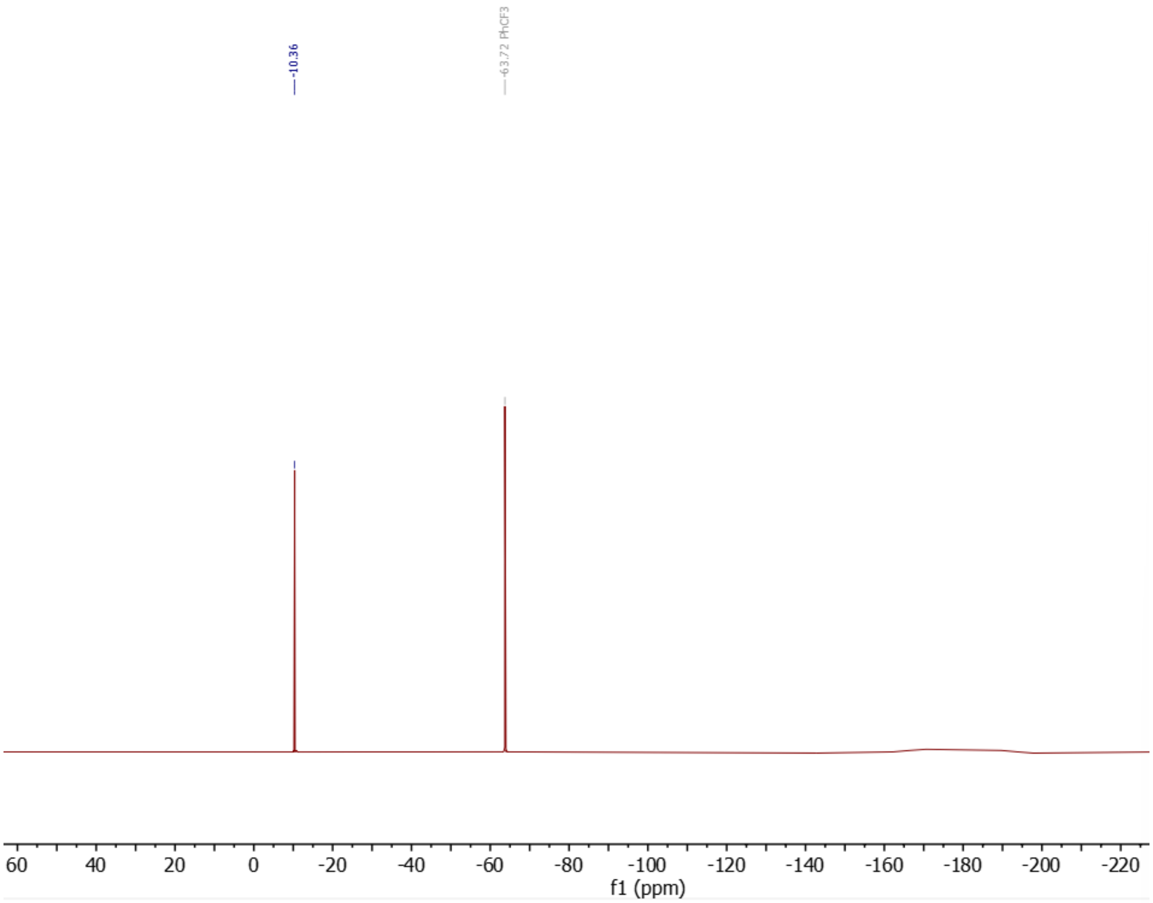

Figure S19. ^{19}F NMR spectrum of CF_3I (THF) delivered from Al–fum.

CF_3CF_2I–Al–fum:

^{19}F NMR (376 MHz, THF): δ −72.08 (q, J = 5.0 Hz, 2F), −85.83 (t, J = 4.0 Hz, 3F) ppm. Spectral data is consistent with those reported in literature.[13]

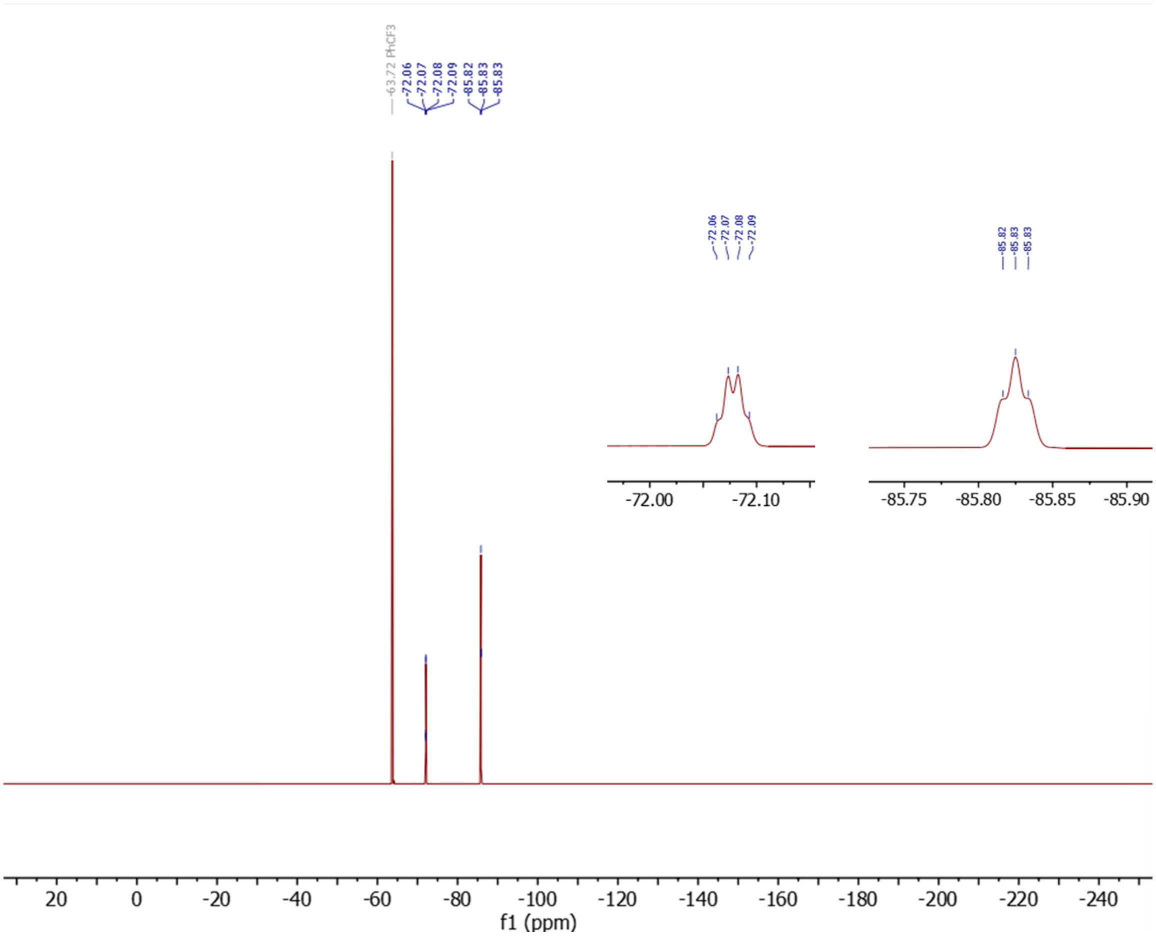

Figure S20. ^{19}F NMR spectrum of CF_3CF_2I (THF) delivered from Al–fum.

***Ex-situ* Gas Generation and Capture for Preparing CF_2HI–Al–fum.**

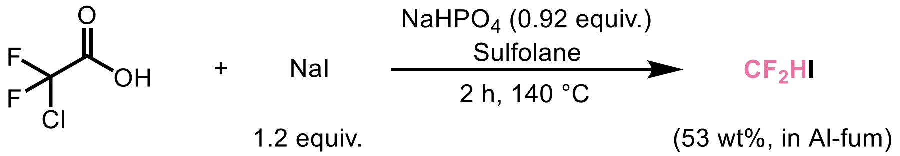

Difluoromethyl iodide (CF_2HI) was generated by a modified literature procedure.[1] A three-neck 100 mL round-bottom flask equipped with a large stir bar was charged with NaI (9.0 g, 60.0 mmol, 1.2 equiv.), Na_2HPO_4 (6.6 g, 46.0 mmol, 0.92 equiv.), and sulfolane (20.0 mL). Then, the flask was connected to a distillation condenser connected to a receiving flask containing activated Al–fum (3.0 g) cooled to −78 °C (acetone/dry ice). The vacuum port was sealed with a rubber stopper fitted with a needle to relieve pressure. After purging with N_2 for 5 min, the reaction mixture was heated to 140 °C in an oil bath. Chlorodifluoroacetic acid (6.5 g, 50.0 mmol, 1.0 equiv.) was added dropwise over 1 h. CF_2HI was generated and distilled into the receiving flask, where it was captured by Al–fum. The mixture was stirred for an additional 1 h at 140 °C, then

cooled and removed from the heat. The resulting CF_2HI–Al–fum was transferred to an oven-dried 20 mL vial, gently warmed to 0 °C, weighed, and sealed.

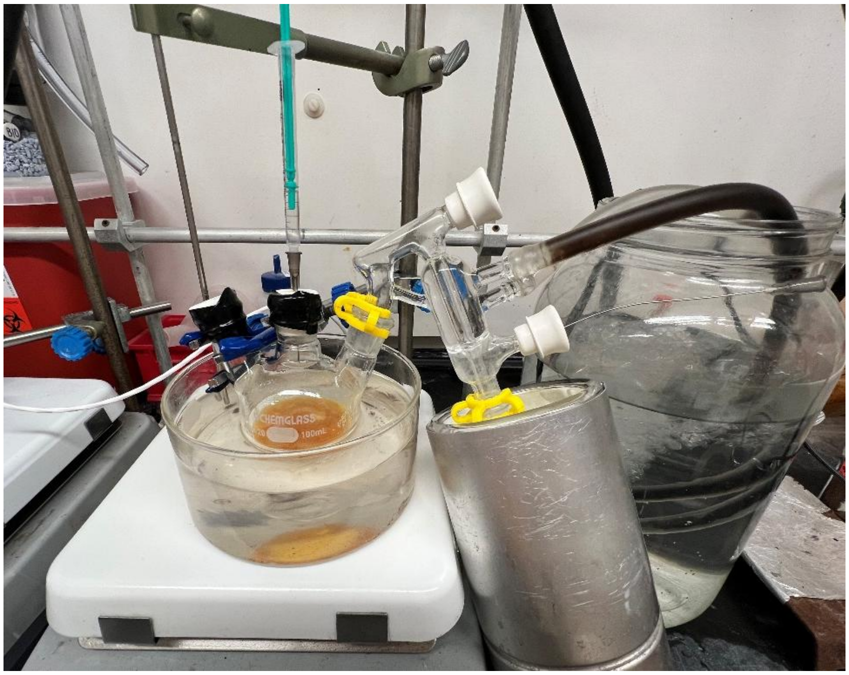

Figure S21. Set-up of *ex-situ* gas generation and capture.

The gas delivery was quantified by ^{19}F NMR ($PhCF_3$ as internal standard). Freshly prepared CF_2HI–Al–fum (20.0–30.0 mg) was weighed into an 8 mL vial. THF (1 mL) was then added, and the mixture was allowed to stand for 5 minutes before adding $PhCF_3$ (12.0 µL, 0.10 mmol). Based on ^{19}F NMR analysis, freshly prepared CF_2HI–Al–fum typically contains 53 wt% CF_2HI.

^{19}F NMR (376 MHz, THF): δ −68.58 (d, *J* = 55.7 Hz, 2F) ppm. Spectral data is consistent with those reported in literature.[1]

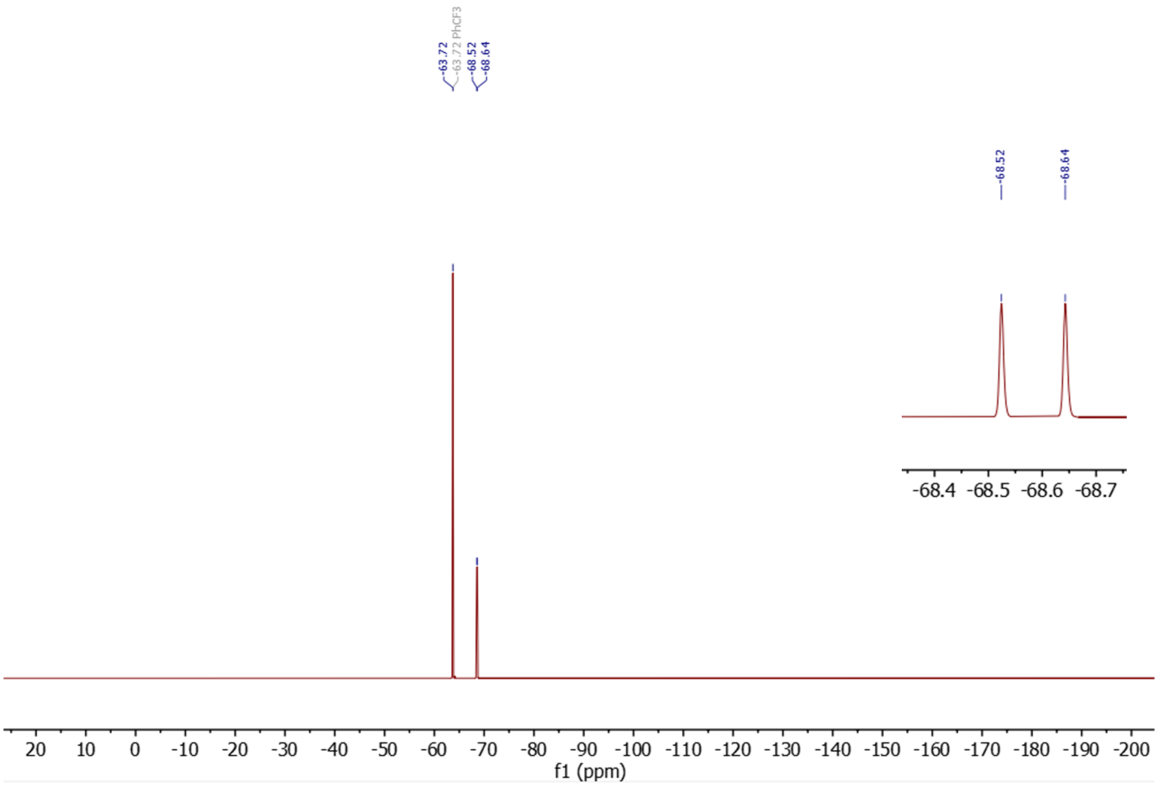

Figure S22. ^{19}F NMR spectrum of CF_2HI (THF) delivered from Al–fum.

4. Solvent-Independent Delivery using Gas–Al–fum.

To further evaluate the practical advantage of gas–Al–fum over conventional stock-solution delivery methods, we examined the ability of the MOF reagent to release CF_3I, CF_3CF_2I, and CF_2HI directly into a range of commonly used organic solvents (THF, hexanes, 1,4-dioxane, CH_2Cl_2, MeCN, and DMF). Unlike DMF-based stock solutions, which must be freshly prepared and are solvent-specific, all the three gas–Al–fum reagents can be added as a solid to reaction vessels containing different solvents. Upon contact with the solvent, rapid gas release occurs, affording homogeneous solutions within minutes under ambient conditions.

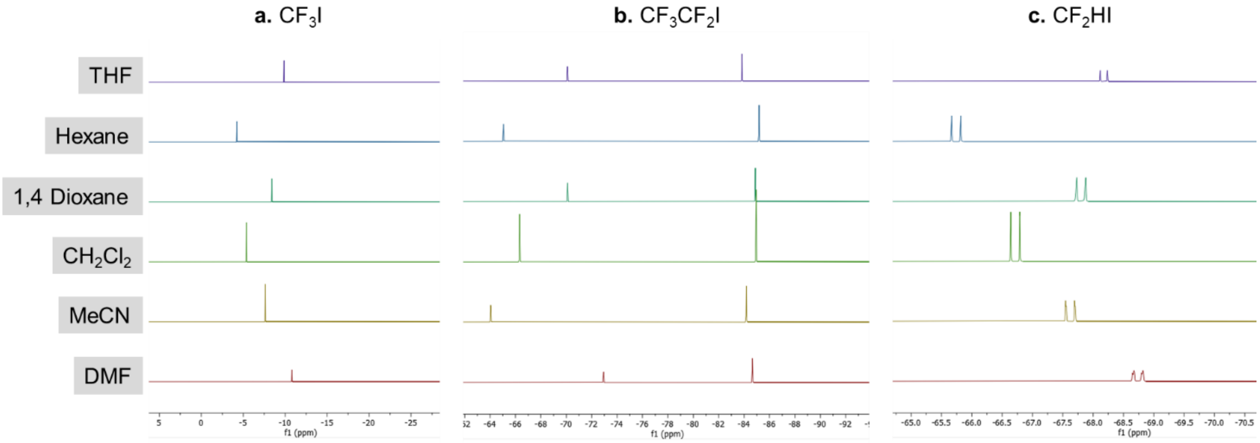

Figure S23. ^{19}F NMR analysis of gas delivery using gas–Al–fum in various solvents. a) CF_3I delivered by CF_3I–Al–fum. b) CF_3CF_2I delivered by CF_3CF_2I–Al–fum. c) CF_2HI delivered by CF_2HI–Al–fum.

5. Gas Release Analysis of Gas–MOF Reagents.

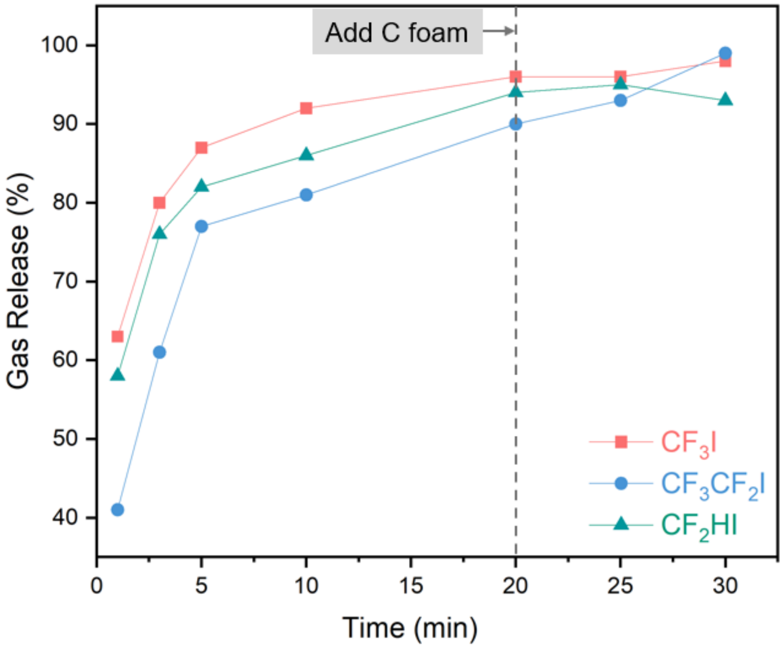

Figure S24. Gas release kinetics in DMF. Red: CF_3I; blue: CF_3CF_2I; green: CF_2HI.

To evaluate whether Al–fum modulates the release rate of fluoroalkyl iodides or alters their local concentration near the electrode surface, we performed gas release studies in DMF under reaction-relevant conditions. Pre-weighed samples of CF_3I–Al–fum, CF_3CF_2I–Al–fum, and CF_2HI–Al–fum were suspended in DMF at room temperature, and the concentration of released gas was quantified over time by ^{19}F NMR. For all three gas–MOF reagents, >90% of the encapsulated fluoroalkyl iodide was released into solution within 20 min.

To determine whether the presence of porous carbon foam influences gas distribution or promotes localized concentration effects at the electrode interface, carbon foam was added to the solution, and the concentration of fluoroalkyl iodides was continuously monitored. No measurable change in the dissolved gas concentration was observed upon addition of the carbon foam. These results indicate that Al–fum does not measurably regulate fluoroalkyl iodide concentration at the electrode surface under the reaction conditions.

6. Density Functional Theory (DFT) Calculated Structure of CF_3CF_2I–Al–fum.

DFT calculations were conducted to compute binding energies of halogenated compounds in Al–fum. The crystal structures were optimized using the Vienna *Ab Initio* Simulation Package (VASP) software[14–17] with the Perdew Burke–Ernzerhof (PBE) exchange–correlation functional[18,19] and Grimme's D3 dispersion correction to take into account van der Waals interactions[20]. The pseudopotentials were described by the Projector–Augmented Wave (PAW) method[21,22]. We used (i) a 500 eV plane–wave cutoff energy and (ii) a 2×2×2 k–point grid. Since the Al–fum framework is highly flexible, the shape and volume of the structure were fixed during relaxation to avoid significant contraction of the pore window[23–25]. Structural optimization was performed until Hellmann–Feynman force values reached the 0.01 eV/Å criterion, and the electronic convergence was achieved at 10^{-7} eV. The adsorption energy of the halogenated compounds (ΔE_B) was calculated using equation:

$$\Delta E_B = E_{adsorbate+MOF} - (E_{MOF} + E_{adsorbate})$$

where $E_{adsorbate+MOF}$ is the total energy of the Al–fum containing the adsorbate while E_{MOF} is the total energy of the empty MOF. The calculation of the adsorption energy was done in the 2×1×1 supercell for both containing adsorbates and empty MOF. $E_{adsorbate}$ is the total energy of the isolated molecule in a 15 Å × 15 Å × 15 Å cubic supercell.

Table S1. Computed binding energies (ΔE_B), zero-point energy (ZPE) and thermal energy (TE) corrections, and binding enthalpies (ΔH_B) (in kJ/mol) of CF_3CF_2I in Al–fum.

Gas	ΔE_B	ZPE	TE	ΔH_B
CF_3CF_2I	−63.99	1.35	5.45	−57.19

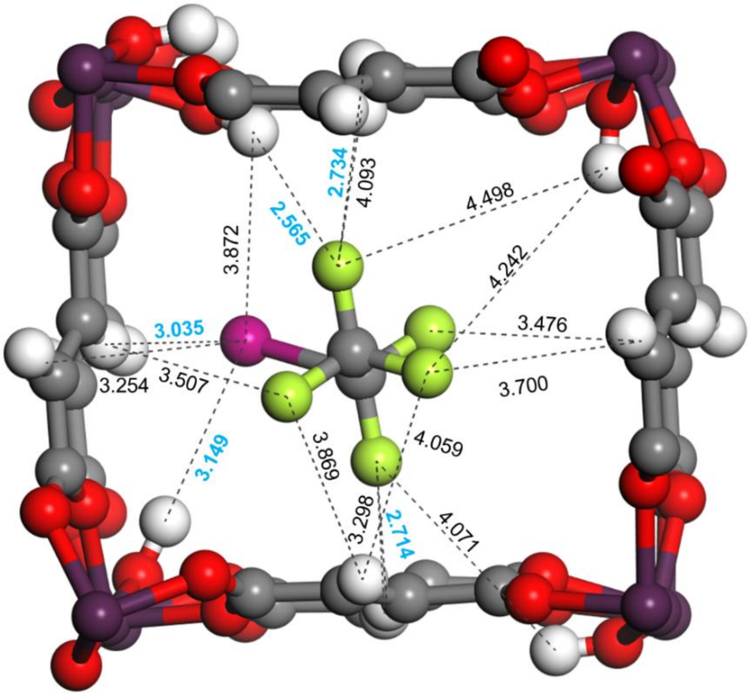

Figure S25. DFT-optimized configuration of CF_3CF_2I bound in Al–fum. Gray, red, white, plum, green, and magenta spheres correspond to carbon, oxygen, hydrogen, aluminum, fluorine, and iodine, respectively. Stronger hydrogen-bonding interactions with distances ranging from 2.40 to 3.15 Å are shown in blue.

7. Synthesis of Starting Materials.

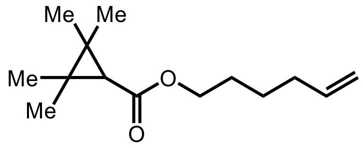

hex-5-en-1-yl 2,2,3,3-tetramethylcyclopropane-1-carboxylate (S1)

Adapted from a literature procedure.[26] A 100 mL round-bottom flask equipped with a stir bar was charged with *N*,*N'*-dicyclohexylcarbodiimide (DCC, 1.80 g, 8.80 mmol, 2.20 equiv.), 2,2,3,3-tetramethylcyclopropane-1-carboxylic acid (568.4 mg, 4.00 mmol, 1.00 equiv.), and 4-dimethylaminopyridine (DMAP, 48.8 mg, 0.40 mmol, 0.10 equiv.). Dry CH_2Cl_2 (20 mL) was added via syringe, and the resulting suspension was cooled to 0 °C in an ice-water bath. Hex-5-en-1-ol (864.1 µL, 7.20 mmol, 1.80 equiv.) was added dropwise. The cooling bath was then removed, and the reaction mixture was allowed to warm to room temperature and stirred for 12 h. The reaction mixture was then diluted with water, and the layers were separated. The aqueous layer was extracted with EtOAc (3 × 20 mL). The combined organic layers were concentrated under reduced pressure, and the crude product was purified by flash chromatography (SiO_2, gradient of 0% → 5% EtOAc in hexanes) to afford the desired compound **S1** (645.6 mg, 72%) as a colorless oil.

^{1}H NMR (500 MHz, $CDCl_3$): δ 5.85–5.75 (m, 1H), 5.03–4.94 (m, 2H), 4.02 (t, *J* = 6.8 Hz, 2H), 2.08 (q, *J* = 7.3 Hz, 2H), 1.63 (t, *J* = 7.5 Hz, 2H), 1.45 (p, *J* = 7.6 Hz, 2H), 1.23 (s, 6H), 1.17 (s, 7H) ppm.
^{13}C NMR (126 MHz, $CDCl_3$): δ 172.42, 138.61, 114.85, 63.70, 35.93, 33.47, 30.03, 28.38, 25.45, 23.68, 16.73 ppm.
MS (DART) m/z: $[M+H]^+$ Calcd. for $C_{14}H_{25}O_2^+$: 225.1855; Found: 225.1867.

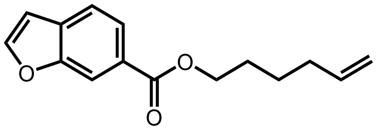

hex-5-en-1-yl benzofuran-6-carboxylate (S2)

Adapted from a literature procedure.[26] A 100 mL round-bottom flask equipped with a stir bar was charged with DCC (1.80 g, 8.80 mmol, 2.20 equiv.), benzofuran-6-carboxylic acid (648.1 mg, 4.00 mmol, 1.00 equiv.), and DMAP (48.8 mg, 0.40 mmol, 0.10 equiv.). Dry CH_2Cl_2 (20 mL) was added via syringe, and the resulting suspension was cooled to 0 °C in an ice-water bath. Hex-5-en-1-ol (864.1 µL, 7.20 mmol, 1.80 equiv.) was added dropwise. The cooling bath was then removed, and the reaction mixture was allowed to warm to room temperature and stirred for 12 h. The reaction mixture was then diluted with water, and the layers were separated. The aqueous layer was extracted with EtOAc (3 × 20 mL). The combined organic layers were concentrated under

reduced pressure, and the crude product was purified by flash chromatography (SiO_2, gradient of 0% → 15% EtOAc in hexanes) to afford the desired compound **S2** (595.6 mg, 61%) as a colorless oil.

^{1}H NMR (500 MHz, $CDCl_3$): δ 8.34 (s, 1H), 8.03 (d, J = 8.7 Hz, 1H), 7.67 (s, 1H), 7.52 (d, J = 8.7 Hz, 1H), 6.84 (s, 1H), 5.83 (ddt, J = 16.9, 10.1, 6.6 Hz, 1H), 5.07–4.97 (m, 2H), 4.35 (t, J = 6.5 Hz, 2H), 2.14 (q, J = 7.1 Hz, 2H), 1.81 (p, J = 7.0 Hz, 2H), 1.57 (p, J = 7.6 Hz, 2H) ppm.
^{13}C NMR (126 MHz, $CDCl_3$): δ 166.92, 157.56, 146.31, 138.49, 127.52, 126.13, 125.60, 123.78, 114.99, 111.33, 107.23, 64.99, 33.46, 28.36, 25.47 ppm.
MS (DART) m/z: $[M+H]^+$ Calcd. for $C_{15}H_{17}O_3^+$: 245.1178; Found: 245.1150.

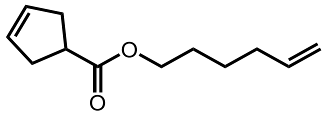

hex-5-en-1-yl cyclopent-3-ene-1-carboxylate (S3)

Adapted from a literature procedure.[26] A 100 mL round-bottom flask equipped with a stir bar was charged with DCC (1.80 g, 8.80 mmol, 2.20 equiv.), cyclopent-3-ene-1-carboxylic acid (413.8 µL, 4.00 mmol, 1.00 equiv.), and DMAP (48.8 mg, 0.40 mmol, 0.10 equiv.). Dry CH_2Cl_2 (20 mL) was added via syringe, and the resulting suspension was cooled to 0 °C in an ice-water bath. Hex-5-en-1-ol (864.1 µL, 7.20 mmol, 1.80 equiv.) was added dropwise. The cooling bath was then removed, and the reaction mixture was allowed to warm to room temperature and stirred for 12 h. The reaction mixture was then diluted with water, and the layers were separated. The aqueous layer was extracted with EtOAc (3 × 20 mL). The combined organic layers were concentrated under reduced pressure, and the crude product was purified by flash chromatography (SiO_2, gradient of 0% → 5% EtOAc in hexanes) to afford the desired compound **S3** (504.4 mg, 65%) as a pale-yellow oil.

^{1}H NMR (500 MHz, $CDCl_3$): δ 5.84–5.73 (m, 1H), 5.65 (s, 2H), 4.98 (dd, J = 20.7, 13.7 Hz, 2H), 4.08 (t, J = 6.5 Hz, 2H), 3.14–3.06 (m, 1H), 2.63 (d, J = 8.1 Hz, 4H), 2.07 (q, J = 7.3 Hz, 2H), 1.68–1.60 (m, 2H), 1.45 (p, J = 7.4 Hz, 2H) ppm.
^{13}C NMR (126 MHz, $CDCl_3$): δ 176.35, 176.33, 138.44, 129.07, 114.91, 64.52, 41.72, 36.41, 33.38, 28.20, 25.30 ppm.
MS (DART) m/z: $[M+H]^+$ Calcd. for $C_{12}H_{19}O_2^+$: 195.1385; Found: 195.1401.

8. General Electrolysis Procedures.

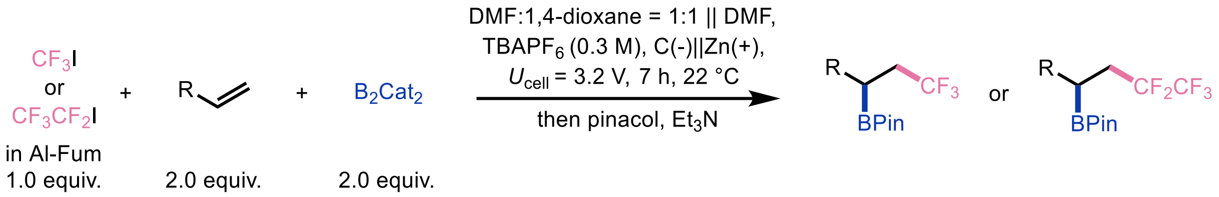

General Procedure A (RXN 1): In a N_2-filled glovebox, an oven-dried custom-made H-type divided cell was equipped with a stir bar on each side, a carbon felt cathode, and a Zn plate anode (Figure S26). The cathodic chamber was charged with bis(catecholato)diboron (B_2Cat_2; 190.2 mg, 0.80 mmol, 2.00 equiv.) and tetrabutylammonium hexafluorophosphate ($TBAPF_6$; 232.5 mg, 0.60 mmol, 1.50 equiv.), while the anodic chamber was charged with $TBAPF_6$ (232.5 mg, 0.60 mmol, 1.50 equiv.). Then, anhydrous degassed 1,4-dioxane (1.0 mL) and DMF (1.0 mL) were added to the cathodic chamber, while the anodic chamber was charged with DMF (2.0 mL). Subsequently, the alkene substrate (0.80 mmol, 2.00 equiv.) and gas–Al–fum (CF_3I–Al–fum, 179.4 mg, 42 wt% CF_3I, or CF_3CF_2I–Al–fum, 220.0 mg, 67 wt% CF_3CF_2I, 0.40 mmol, 1.0 equiv.) were added to the cathodic chamber. The cell was sealed, transferred out from the glovebox, and stirred at a constant cell potential of 3.2 V at 22 ºC for 7 h. After completion of the reaction, Et_3N (223 μL, 1.60 mmol, 4.00 equiv.) and pinacol (189.0 mg, 1.60 mmol, 4.00 equiv.) were added to the cathodic chamber, and the mixture was stirred for another 1 h. Then, the reaction mixture of each chamber was collected, and the chambers were rinsed with additional EtOAc (3 × 5 mL). After concentration under reduced pressure, the product was purified by flash column chromatography on silica gel.

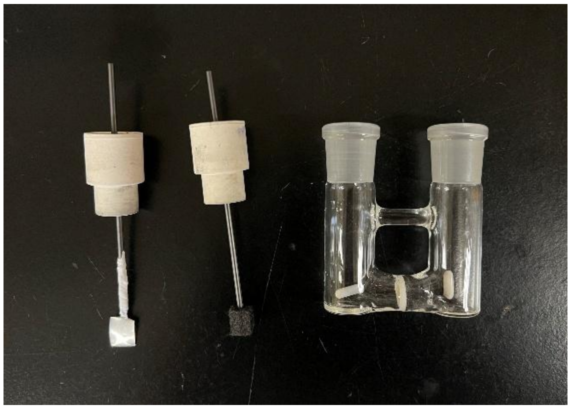

Figure S26. Preparation of electrodes and H-type divided cell.

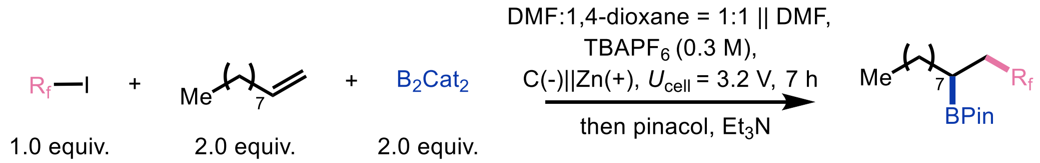

General Procedure B: In a N_2-filled glovebox, an oven-dried custom-made H-type divided cell was equipped with a stir bar on each side, a carbon felt cathode, and a Zn plate anode (Figure S26). The cathodic chamber was charged with B_2Cat_2 (190.2 mg, 0.80 mmol, 2.00 equiv.) and $TBAPF_6$

(232.5 mg, 0.60 mmol, 1.50 equiv.), while the anodic chamber was charged with $TBAPF_6$ (232.5 mg, 0.60 mmol, 1.50 equiv.). Then, anhydrous degassed 1,4-dioxane (1.0 mL) and DMF (1.0 mL) were added to the cathodic chamber, while the anodic chamber was charged with DMF (2.0 mL). Subsequently, 1-decene (152 µL, 0.80 mmol, 2.00 equiv.) and the R_fI substrate (0.40 mmol, 1.00 equiv.) were added to the cathodic chamber. The cell was sealed, transferred out from the glovebox, and stirred at a constant cell potential of 3.2 V at 22 °C for 7 h. After completion of the reaction, Et_3N (223 µL, 1.60 mmol, 4.00 equiv.) and pinacol (189.0 mg, 1.60 mmol, 4.00 equiv.) were added to the cathodic chamber, and the mixture was stirred for another 1 h. Then, the reaction mixture of each chamber was collected, and the chambers were rinsed with additional EtOAc (3 × 5 mL). After concentration under reduced pressure, the product was purified by flash column chromatography on silica gel.

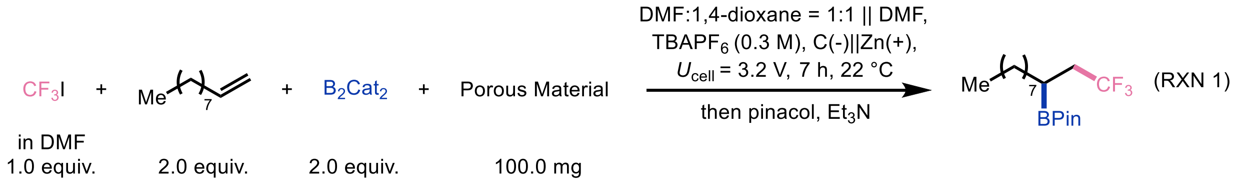

General Procedure C: Porous materials as additives in electrochemical reactions (RXN 1). In a N_2-filled glovebox, an oven-dried custom-made H-type divided cell was equipped with a stir bar on each side, a carbon felt cathode, and a Zn plate anode (Figure S26). The cathodic chamber was charged with B_2Cat_2 (190.2 mg, 0.80 mmol, 2.00 equiv.), $TBAPF_6$ (232.5 mg, 0.60 mmol, 1.50 equiv.) and the porous material additive (100.0 mg), while the anodic chamber was charged with $TBAPF_6$ (232.5 mg, 0.60 mmol, 1.50 equiv.). Then, anhydrous degassed 1,4-dioxane (1.0 mL) and DMF (0.5 mL) were added to the cathodic chamber, while the anodic chamber was charged with DMF (2.0 mL). Subsequently, 1-decene (152 µL, 0.80 mmol, 2.00 equiv.) and CF_3I (in DMF; 533 µL, 0.75 M CF_3I, 0.40 mmol, 1.00 equiv.) were added to the cathodic chamber. The cell was sealed, transferred out from the glovebox, and stirred at a constant cell potential of 3.2 V at 22 °C for 7 h. After completion of the reaction, Et_3N (223 µL, 1.60 mmol, 4.00 equiv.) and pinacol (189.0 mg, 1.60 mmol, 4.00 equiv.) were added to the cathodic chamber, and the mixture was stirred for another 1 h. Then, the reaction mixture was vacuum-filtered to remove the porous material. The NMR yield of the reaction was determined by ^{19}F NMR ($PhCF_3$ as internal reference).

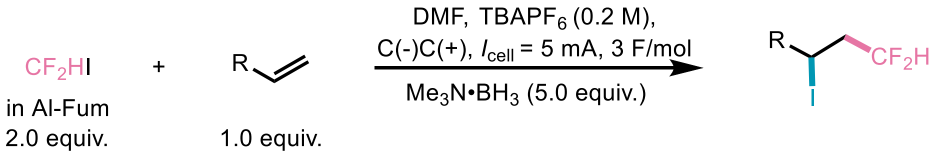

General Procedure D (RXN 2): In a N_2-filled glovebox, an oven-dried 20 mL glass vial was equipped with a stir bar and two carbon felt electrodes (Figure S26). The flask was charged with $TBAPF_6$ (309.8 mg, 0.80 mmol, 2.00 equiv.), borane trimethylamine complex ($Me_3N\cdot BH_3$; 145.9 mg, 2.00 mmol, 5.00 equiv.) and anhydrous degassed DMF (4.0 mL). Then, the alkene substrate (0.80 mmol, 1.00 equiv.) and CF_2HI–Al–fum (134.8 mg, 53 wt% CF_2HI, 0.80 mmol, 2.00 equiv.)

were added sequentially. The vial was sealed, transferred out of the glovebox, and stirred at a constant cell current of 5 mA at 22 °C for 13 h. When the reaction was finished, the reaction mixture was collected, and the vial was rinsed with additional EtOAc (3 × 5 mL). After concentration under reduced pressure, the product was purified by flash column chromatography on silica gel.

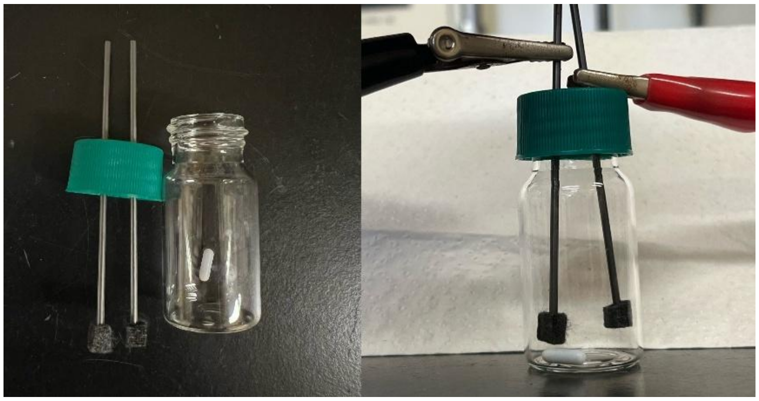

Figure S27. Preparation of electrodes and undivided cell.

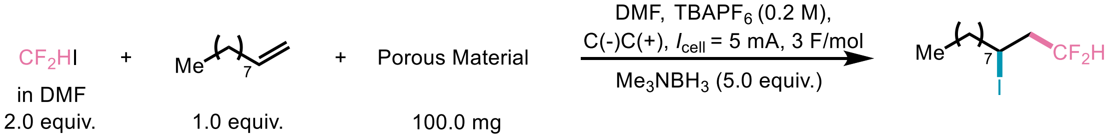

(RXN 2)

General Procedure E: Porous materials as additives in electrochemical reactions (RXN 2). In a N_2-filled glovebox, an oven-dried 20 mL glass vial was equipped with a stir bar and two carbon felt electrodes (Figure S27). The flask was charged with $TBAPF_6$ (309.8 mg, 0.80 mmol, 2.00 equiv.), $Me_3N{\cdot}BH_3$ (145.9 mg, 2.00 mmol, 5.00 equiv.), the porous material additive (100.0 mg), and anhydrous degassed DMF (4.0 mL). Then, 1-decene (76.0 µL, 0.40 mmol, 1.00 equiv.) and CF_2HI (0.55 M in DMF, 0.80 mmol, 2.00 equiv.) were added sequentially. The vial was sealed, transferred out of the glovebox, and stirred at a constant cell current of 5 mA at 22 °C for 13 h. Upon completion of the reaction, the reaction mixture was vacuum-filtered to remove the porous material. The NMR yield of the reaction was determined by ^{19}F NMR ($PhCF_3$ as internal reference).

General procedure for the recycling experiments: After completion of the reaction, Al–fum was recovered by filtration, washed with acetone (3 × 20 mL), and dried in air. The material was then transferred to an oven-dried 8 mL screw-cap septum vial and activated under high vacuum (<100 mTorr) at room temperature for 2 h, followed by heating at 150 °C under high vacuum for an additional 3 h. After cooling the vial to −78 °C (acetone/dry ice bath), the MOF was recharged with the corresponding gas according to the general procedures for gas–MOF preparation (Section 3). The regenerated gas–MOF was then subjected to subsequent reactions following General Procedure A or D.

Table S2. Results for the capacity of the gas–MOFs by using recycled Al–fum and the corresponding ^{19}F NMR yield of for each reaction ($PhCF_3$ as internal reference).

Gas–MOF	Capacity using recycled Al–fum	^{19}F NMR yield
CF_3I–Al–fum	30 wt%	69% (RXN 1)
CF_2HI–Al–fum	36 wt%	51% (RXN 2)

Compared to freshly prepared materials, the gas uptake capacities of recycled MOFs were modestly reduced (30 wt% vs 42 wt% for CF_3I–Al–fum; 36 wt% vs 53 wt% for CF_2HI–Al–fum). Notably, this decrease in gas loading had no measurable impact on reaction efficiency, as comparable yields were obtained for both reactions (69% vs 71% for RXN 1; 51% vs 52% for RXN 2).

9. Reaction Optimization.

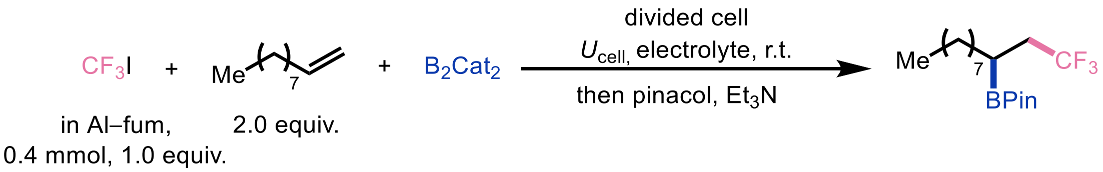

Table S3. Optimization of the electroreductive CF_3-borylation of 1-decene in a divided cell.

Entry	CF_3I	U_{cell} (V)	Electrodes	Solvent	Electrolyte	B_2Cat_2	Time	NMR Yield
**1**	0.1 M	2.5	C foam(−)‖Zn(+)	DMF	$TBAPF_6$ (0.3 M)	2.0 equiv.	7 h	16%
**2**	0.1 M	3.0	C foam(−)‖Zn(+)	DMF	$TBAPF_6$ (0.3 M)	2.0 equiv.	7 h	36%
**3**	0.1 M	3.2	C foam(−)‖Zn(+)	DMF	$TBAPF_6$ (0.3 M)	2.0 equiv.	7 h	41%
**4**	0.2 M	3.2	C foam(−)‖Zn(+)	DMF	$TBAPF_6$ (0.3 M)	2.0 equiv.	7 h	47%
**5**	0.3 M	3.2	C foam(−)‖Zn(+)	DMF	$TBAPF_6$ (0.3 M)	2.0 equiv.	7 h	48%
**6**	0.4 M	3.2	C foam(−)‖Zn(+)	DMF	$TBAPF_6$ (0.3 M)	2.0 equiv.	7 h	42%
**7**	0.2 M	3.2	graphite(−)‖Zn(+)	DMF	$TBAPF_6$ (0.3 M)	2.0 equiv.	7 h	12%
**8**	0.2 M	3.2	Pt(−)‖Zn(+)	DMF	$TBAPF_6$ (0.3 M)	2.0 equiv.	7 h	<5%
**9**	0.2 M	3.2	Ag(−)‖Zn(+)	DMF	$TBAPF_6$ (0.3 M)	2.0 equiv.	7 h	<5%
**10**	0.2 M	3.2	C foam(−)‖Mg(+)	DMF	$TBAPF_6$ (0.3 M)	2.0 equiv.	7 h	44%
**11**	0.2 M	3.2	C foam(−)‖Fe(+)	DMF	$TBAPF_6$ (0.3 M)	2.0 equiv.	7 h	31%
**12**	0.2 M	3.2	C foam(−)‖Zn(+)	MeCN	$TBAPF_6$ (0.3 M)	2.0 equiv.	7 h	<5%
**13**	0.2 M	3.2	C foam(−)‖Zn(+)	DMF:MeCN = 1:1 ‖ DMF	$TBAPF_6$ (0.3 M)	2.0 equiv.	7 h	35%
**14**	0.2 M	3.2	C foam(−)‖Zn(+)	THF	$TBAPF_6$ (0.3 M)	2.0 equiv.	7 h	22%
**15**	0.2 M	3.2	C foam(−)‖Zn(+)	DMF:THF = 1:1 ‖ DMF	$TBAPF_6$ (0.3 M)	2.0 equiv.	7 h	68%
**16**	0.2 M	3.2	C foam(−)‖Zn(+)	DMF:1,4-dioxane = 1:1 ‖ DMF	$TBAPF_6$ (0.3 M)	2.0 equiv.	7 h	71% **(Standard)**

**17**	0.2 M	3.2	C foam(−)‖Zn(+)	DMF:1,4-dioxane = 1:1 ‖ DMF	$TBAPF_6$ (0.2 M)	2.0 equiv.	7 h	55%
**18**	0.2 M	3.2	C foam(−)‖Zn(+)	DMF:1,4-dioxane = 1:1 ‖ DMF	$TBAPF_6$ (0.4 M)	2.0 equiv.	7 h	41%
**19**	0.2 M	3.2	C foam(−)‖Zn(+)	DMF:1,4-dioxane = 1:1 ‖ DMF	$NaClO_4$ (0.3 M)	2.0 equiv.	7 h	29%
**20**	0.2 M	3.2	C foam(−)‖Zn(+)	DMF:1,4-dioxane = 1:1 ‖ DMF	KPF_6 (0.3 M)	2.0 equiv.	7 h	42%
**21**	0.2 M	3.2	C foam(−)‖Zn(+)	DMF:1,4-dioxane = 1:1 ‖ DMF	$TBAPF_4$ (0.3 M)	2.0 equiv.	7 h	68%
**22**	0.2 M	3.2	C foam(−)‖Zn(+)	DMF:1,4-dioxane = 1:1 ‖ DMF	$TBAClO_4$ (0.3 M)	2.0 equiv.	7 h	49%
**23**	0.2 M	3.2	C foam(−)‖Zn(+)	1,4-dioxane	$TBAPF_6$ (0.3 M)	2.0 equiv.	7 h	<5%
**24**	0.2 M	3.2	C foam(−)‖Zn(+)	DMF:1,4-dioxane = 1:1 ‖ DMF	$TBAPF_6$ (0.3 M)	2.0 equiv.	8 h	72%
**25**	0.2 M	3.2	C foam(−)‖Zn(+)	DMF:1,4-dioxane = 1:1 ‖ DMF	$TBAPF_6$ (0.3 M)	2.0 equiv.	10 h	70%
**26**	0.2 M	3.2	C foam(−)‖Zn(+)	DMF:1,4-dioxane = 1:1 ‖ DMF	$TBAPF_6$ (0.3 M)	3.0 equiv.	7 h	64%

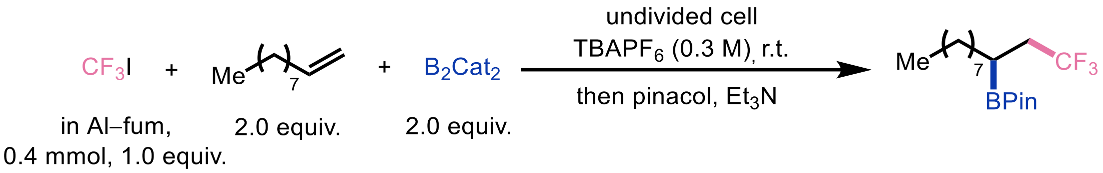

Table S4. Optimization of the electroreductive CF_3-borylation of 1-decene in an undivided cell.

Entry	Electricity	Electrodes	Solvent	Electrolyte	Time	Additive	NMR yield
1	I_{cell}=5 mA	C foam(−)| C foam(+)	DMF	$TBAPF_6$ (0.3 M)	4 h	DIPEA (3.0 equiv.)	51%
2	I_{cell}=5 mA	C foam(−)| Mg(+)	DMF	$TBAPF_6$ (0.3 M)	4 h	DIPEA (3.0 equiv.)	14%
3	I_{cell}=5 mA	C foam(−)| Zn(+)	DMF	$TBAPF_6$ (0.3 M)	4 h	DIPEA (3.0 equiv.)	<5%
4	I_{cell}=5 mA	C foam(−)| C foam(+)	DMF	$TBAPF_6$ (0.3 M)	4 h	NMe_3BH_3 (3.0 equiv.)	42%
5	I_{cell}=5 mA	C foam(−)| C foam(+)	DMF	$TBAPF_6$ (0.3 M)	4 h	NEt_3 (3.0 equiv.)	23%
6	U_{cell}=3.2 V	C foam(−)| C foam(+)	DMF	$TBAPF_6$ (0.3 M)	4 h	DIPEA (3.0 equiv.)	33%
7	U_{cell}=2.5 V	C foam(−)| C foam(+)	DMF	$TBAPF_6$ (0.3 M)	4 h	DIPEA (3.0 equiv.)	<5%
8	I_{cell}=5 mA	C foam(−)| C foam(+)	DMF	$TBAPF_6$ (0.3 M)	8 h	DIPEA (3.0 equiv.)	47%
9	U_{cell}=3.2 V	C foam(−)| C foam(+)	DMF	$TBAPF_6$ (0.3 M)	8 h	DIPEA (3.0 equiv.)	27%
10	I_{cell}=5 mA	C foam(−)| C foam(+)	1, 4 dioxane	$TBAPF_6$ (0.3 M)	4 h	DIPEA (3.0 equiv.)	<5%
11	I_{cell}=5 mA	C foam(−)| C foam(+)	DMF:1,4 dioxan = 1:1	$TBAPF_6$ (0.3 M)	4 h	DIPEA (3.0 equiv.)	8%
12	I_{cell}=5 mA	C foam(−)| Pt(+)	DMF	$TBAPF_6$ (0.3 M)	4 h	DIPEA (3.0 equiv.)	<5%

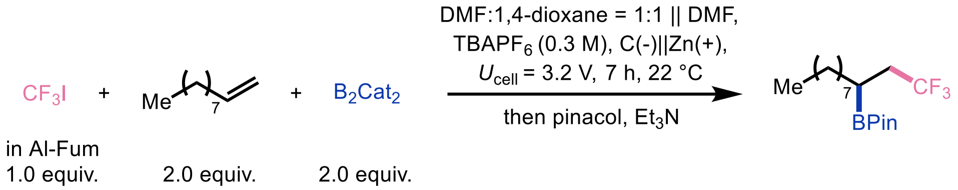

To evaluate the reproducibility of fluoroalkyl iodide loading into Al–fum and its influence on reaction performance, three independent batches of CF_3I–Al–fum were prepared using the standard gas-loading procedure, which revealed consistent gas uptake across batches (Table S5). Each independently prepared batch was subsequently subjected to the standard electroreductive difunctionalization conditions. In all cases, the desired product was obtained in comparable yields, indicating that variations in gas uptake do not measurably affect reaction efficiency when stoichiometry is adjusted based on the measured loading capacity.

Table S5. Reproducibility of gas loading and reaction performance.

**Trials**	**CF_3I Capacity (in Al–fum)**	**^{19}F NMR Yield**
**1**	42 wt%	71%
**2**	40 wt%	73%
**3**	43 wt%	70%
**4**	33 wt% (trial 1 after three months in benchtop desiccator)	75%

These results demonstrate that gas loading into Al–fum is reproducible and that reaction outcomes are governed by the molar quantity of delivered fluoroalkyl iodide rather than the absolute loading percentage, underscoring the robustness and practical reliability of the gas–MOF delivery strategy.

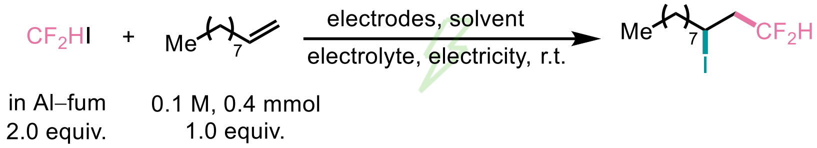

Table S6. Optimization of the CF_2H-iodination of 1-decene.

Entry	Electricity	Electrodes	Solvent	Electrolyte	Additive	NMR Yield
**1**	divided cell, U_{cell} = 2.5 V	C foam(−) ‖Zn(+)	DMF	$TBAPF_6$ (0.3 M)	/	<5%
**2**	divided cell, U_{cell} = 3.2 V	C foam(−) ‖Zn(+)	DMF	$TBAPF_6$ (0.3 M)	/	<5%
**3**	divided cell, U_{cell} = 3.8 V	C foam(−) ‖Zn(+)	DMF	$TBAPF_6$ (0.3 M)	/	<5%
**4**	undivided cell, I_{cell} = 5 mA	C foam(−) ‖ C foam(+)	DMF	$TBAPF_6$ (0.3 M)	/	31%
**5**	undivided cell, I_{cell} = 5 mA	C foam(−) ‖ Mg(+)	DMF	$TBAPF_6$ (0.3 M)	/	11%
**6**	undivided cell, I_{cell} = 5 mA	C foam(−) ‖ Fe(+)	DMF	$TBAPF_6$ (0.3 M)	/	8%
**7**	undivided cell, I_{cell} = 5 mA	C foam(−) ‖ C foam(+)	DMF	$TBAPF_6$ (0.3 M)	Et_3N (5.0 equiv.)	13%
**8**	undivided cell, I_{cell} = 5 mA	C foam(−) ‖ C foam(+)	DMF	$TBAPF_6$ (0.3 M)	DIPEA (5.0 equiv.)	33%
**9**	undivided cell, I_{cell} = 5 mA	C foam(−) ‖ C foam(+)	DMF	$TBAPF_6$ (0.3 M)	Me_3NBH_3 (5.0 equiv.)	52% **(Standard)**
**10**	undivided cell, I_{cell} = 5 mA	C foam(−) ‖ C foam(+)	DMF	$TBAPF_6$ (0.3 M)	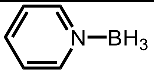 (5.0 equiv.)	<5%
**11**	undivided cell, I_{cell} = 5 mA	C foam(−) ‖ C foam(+)	DMF	$TBAPF_6$ (0.3 M)	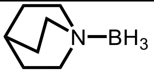 (5.0 equiv.)	29%
**12**	undivided cell, rAC, +50 mA, -50 mA, 50 ms	RVC(+), RVC (−)	DMF	$TBAPF_6$ (0.3 M)	Me_3NBH_3 (5.0 equiv.)	<5%
**13**	undivided cell, I_{cell} = 5 mA	C foam(−) ‖ C foam(+)	DMF	$TBAPF_6$ (0.3 M)	Me_3NBH_3 (3.0 equiv.)	37%
**14**	undivided cell, I_{cell} = 5 mA	C foam(−) ‖ C foam(+)	DMF	$TBAPF_6$ (0.3 M)	Me_3NBH_3 (10 equiv.)	30%
**15**	undivided cell, U_{cell} = 3.2 V	C foam(−) ‖ C foam(+)	DMF	$TBAPF_6$ (0.3 M)	Me_3NBH_3 (5.0 equiv.)	27%
**16**	undivided cell, U_{cell} = 2.5 V	C foam(−) ‖ C foam(+)	DMF	$TBAPF_6$ (0.3 M)	Me_3NBH_3 (5.0 equiv.)	17%

**17**	undivided cell, I_{cell} = 5 mA	C foam(−) || C foam(+)	MeCN	$TBAPF_6$ (0.3 M)	Me_3NBH_3 (5.0 equiv.)	<5%
**18**	undivided cell, I_{cell} = 5 mA	C foam(−) || C foam(+)	THF	$TBAPF_6$ (0.3 M)	Me_3NBH_3 (5.0 equiv.)	<5%
**19**	undivided cell, I_{cell} = 5 mA	C foam(−) || C foam(+)	DMF	KPF_6 (0.3 M)	Me_3NBH_3 (5.0 equiv.)	26%
**20**	undivided cell, I_{cell} = 5 mA	C foam(−) || C foam(+)	DMF	$TBAPF_4$ (0.3 M)	Me_3NBH_3 (5.0 equiv.)	49%
**21**	undivided cell, I_{cell} = 5 mA	C foam(−) || C foam(+)	DMF	$TBAClO_4$ (0.3 M)	Me_3NBH_3 (5.0 equiv.)	32%
**22**	undivided cell, I_{cell} = 5 mA	C foam(−) || C foam(+)	DMF	$NaClO_4$ (0.3 M)	Me_3NBH_3 (5.0 equiv.)	16%
**23**	undivided cell, I_{cell} = 10 mA	C foam(−) || C foam(+)	DMF	$TBAPF_6$ (0.3 M)	Me_3NBH_3 (5.0 equiv.)	51%
**24**	undivided cell, I_{cell} = 2 mA	C foam(−) || C foam(+)	DMF	$TBAPF_6$ (0.3 M)	Me_3NBH_3 (5.0 equiv.)	48%

10. CV Studies.

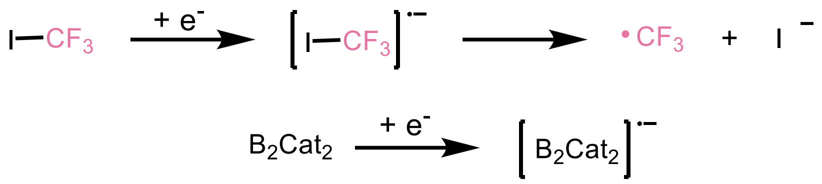

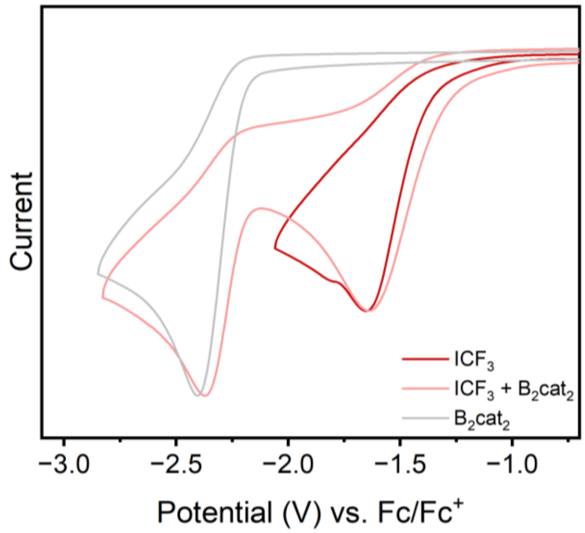

Figure S28. CVs of CF_3I alone (red), CF_3I mixed with B_2Cat_2 (pink), and B_2Cat_2 alone (gray), all 5 mM solutions dissolved in an electrolyte solution of $TBAPF_6$ (0.1 M) in DMF in a N_2-filled glovebox with Fc as an internal reference. Working electrode: glassy carbon; counter electrode: platinum wire, reference electrode: Ag/Ag^+ (0.01 M $AgNO_3$ in 0.1 M $TBAPF_6$/DMF). Scan rate: 50 mV/s.

CV studies indicate that B_2Cat_2 is much harder to reduce ($E_{p/2} = -2.27$ V vs. Fc/Fc^+) than CF_3I ($E_{p/2} = -1.36$ V vs Fc/Fc^+) and its presence does not significantly affect the reduction of CF_3I.

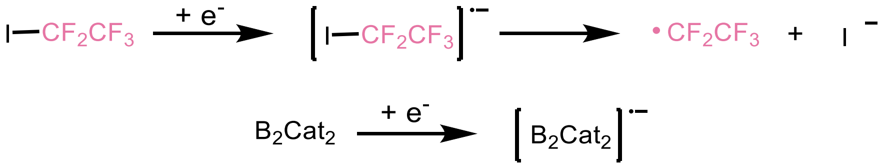

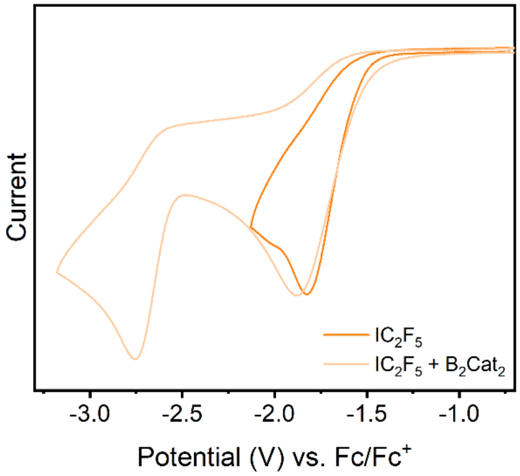

Figure S29. CVs of 1,1,1,2,2-pentafluoro-2-iodoethane (C_2F_5I) alone (dark orange) and C_2F_5I mixed with B_2Cat_2 (light orange), all 5 mM solutions dissolved in an electrolyte solution of $TBAPF_6$ (0.1 M) in DMF in a N_2-filled glovebox with Fc as an internal reference. Working electrode: glassy carbon; counter electrode: platinum wire, reference electrode: Ag/Ag^+ (0.01 M $AgNO_3$ in 0.1 M $TBAPF_6$/DMF). Scan rate: 50 mV/s.

CV studies indicate that B_2Cat_2 does not significantly affect the reduction of CF_3CF_2I ($E_{p/2}$ = −1.61 V vs Fc/Fc^+).

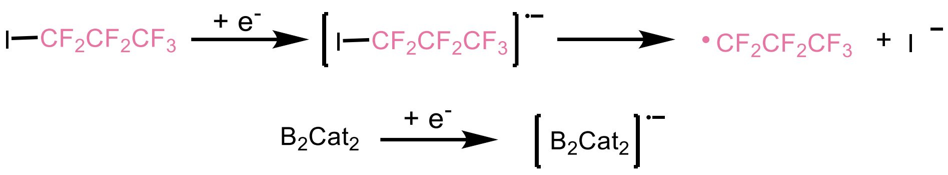

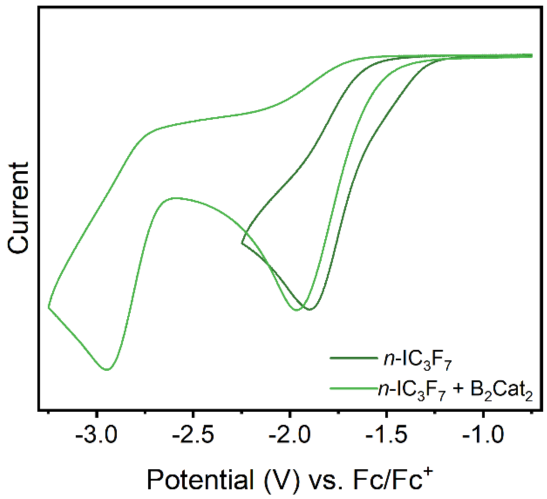

Figure S30. CVs of 1,1,1,2,2,3,3-heptafluoro-3-iodopropane (n-C_3F_7I) alone (dark green) and n-C_3F_7I mixed with B_2Cat_2 (light green), all 5 mM solutions dissolved in an electrolyte solution of $TBAPF_6$ (0.1 M) in DMF in a N_2-filled glovebox with Fc as an internal reference. Working electrode: glassy carbon; counter electrode: platinum wire, reference electrode: Ag/Ag^+ (0.01 M $AgNO_3$ in 0.1 M $TBAPF_6$/DMF). Scan rate: 50 mV/s.

CV studies indicate that B_2Cat_2 does not significantly affect the reduction of n-C_3F_7I ($E_{p/2}$ = −1.59 V vs Fc/Fc^+).

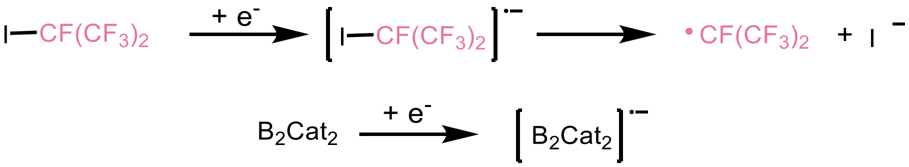

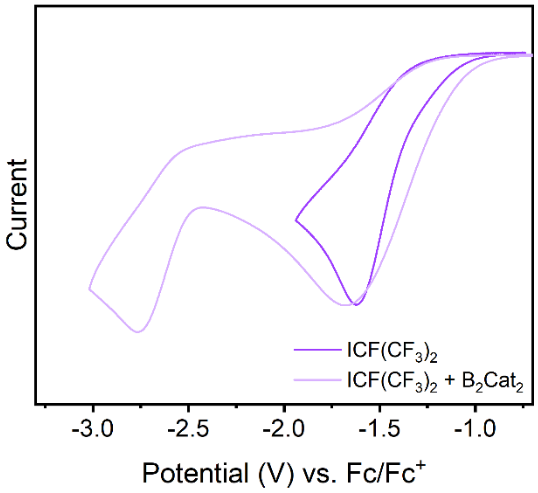

Figure S31. CVs of 1,1,1,2,3,3,3-heptafluoro-2-iodopropane ($ICF(CF_3)_2$) alone (dark purple) and $ICF(CF_3)_2$ mixed with B_2Cat_2 (light purple), all 5 mM solutions dissolved in an electrolyte solution of $TBAPF_6$ (0.1 M) in DMF in a N_2-filled glovebox with Fc as an internal reference. Working electrode: glassy carbon; counter electrode: platinum wire, reference electrode: Ag/Ag^+ (0.01 M $AgNO_3$ in 0.1 M $TBAPF_6$/DMF). Scan rate: 50 mV/s.

CV studies indicate that B_2Cat_2 does not significantly affect the reduction of $ICF(CF_3)_2$ ($E_{p/2}$ = −1.29 V vs Fc/Fc^+).

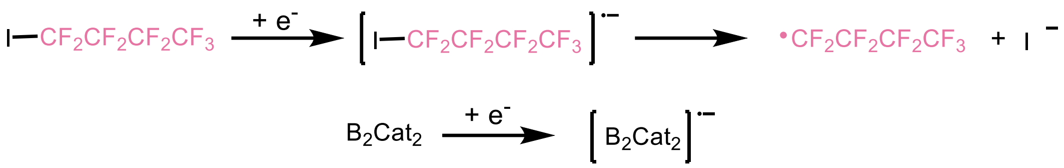

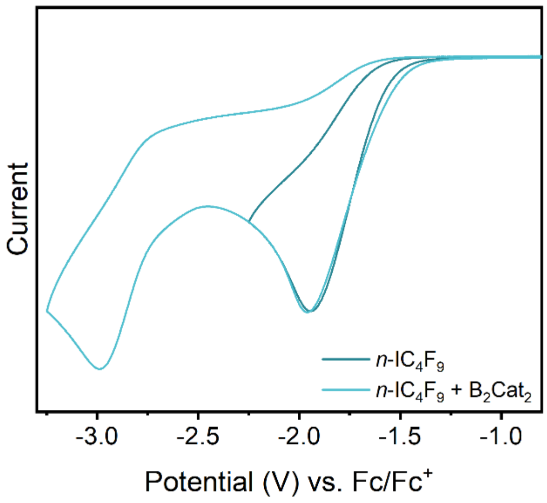

Figure S32. CVs of 1,1,1,2,2,3,3,4,4-nonafluoro-4-iodobutane (*n*-C_4F_9I) alone (dark blue) and C_4F_9I mixed with B_2Cat_2 (light blue), all 5 mM solutions dissolved in an electrolyte solution of $TBAPF_6$ (0.1 M) in DMF in a N_2-filled glovebox with Fc as an internal reference. Working electrode: glassy carbon; counter electrode: platinum wire, reference electrode: Ag/Ag^+ (0.01 M $AgNO_3$ in 0.1 M $TBAPF_6$/DMF). Scan rate: 50 mV/s.

CV studies indicate that B_2Cat_2 does not significantly affect the reduction of *n*-C_4F_9I ($E_{p/2} = -1.67$ V vs Fc/Fc^+).

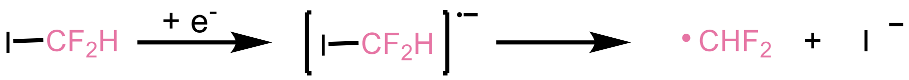

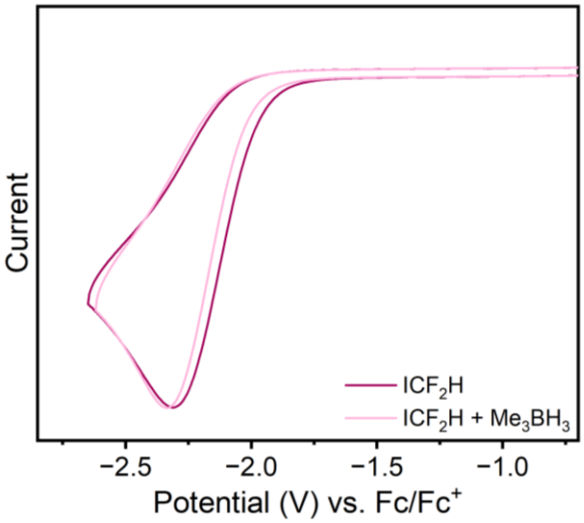

Figure S33. CVs of CF_2HI alone (purple) and CF_2HI mixed with $Me_3N{\cdot}BH_3$ (pink), all 5 mM solutions dissolved in an electrolyte solution of $TBAPF_6$ (0.1 M) in DMF in a N_2-filled glovebox with Fc as an internal reference. Working electrode: glassy carbon; counter electrode: platinum wire, reference electrode: Ag/Ag^+ (0.01 M $AgNO_3$ in 0.1 M $TBAPF_6$/DMF). Scan rate: 50 mV/s.

CV studies indicate that $Me_3N{\cdot}BH_3$ does not significantly affect the reduction of CF_2HI ($E_{p/2}$ = −2.05 V vs Fc/Fc^+).

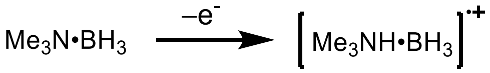

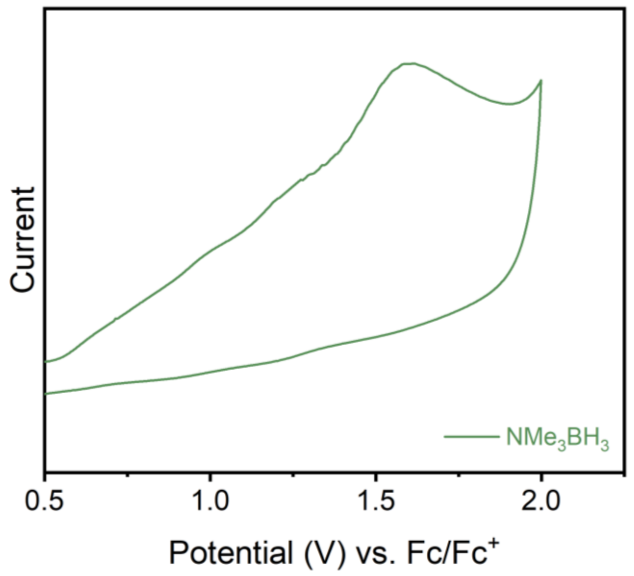

Figure S34. CV of $Me_3N{\cdot}BH_3$ dissolved in an electrolyte solution of $TBAPF_6$ (0.1 M) in DMF in a N_2-filled glovebox with Fc as an internal reference. Working electrode: glassy carbon; counter electrode: platinum wire, reference electrode: Ag/Ag^+ (0.01 M $AgNO_3$ in 0.1 M $TBAPF_6$/DMF). Scan rate: 50 mV/s. $E_{p/2}$ = +1.18 V vs. Fc/Fc^+.

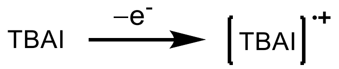

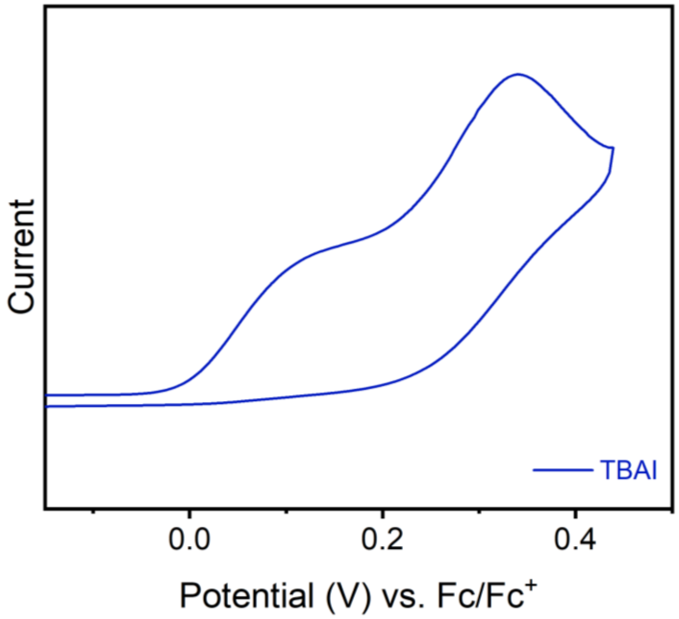

Figure S35. CV of tetrabutylammonium iodide (TBAI) dissolved in an electrolyte solution of $TBAPF_6$ (0.1 M) in DMF in a N_2-filled glovebox with Fc as an internal reference. Working electrode: glassy carbon; counter electrode: platinum wire, reference electrode: Ag/Ag^+ (0.01 M $AgNO_3$ in 0.1 M $TBAPF_6$/DMF). Scan rate: 50 mV/s. $E_{p/2}$ = +0.16 V vs. Fc/Fc^+.

Comparison of the redox potentials of TBAI (+0.16 V) and $Me_3N \cdot BH_3$ (+1.18 V) reveals that I^- is more readily oxidized than $Me_3N \cdot BH_3$. This suggests that anodic oxidation of I^- to I_2 can compete effectively when $Me_3N \cdot BH_3$ is present.

Al–fum:

Procedure adapted from the literature.[27] Preparation of the working electrode: carbon black (1.0 mg), PVDF (1.0 mg), and Al–fum (2.0 mg) were dispersed in 2.0 mL *N*-methyl-2-pyrrolidone (NMP) and sonicated for 2 h. A 20 μL aliquot of the suspension was drop-cast onto a glassy carbon electrode and dried under a heat lamp for 6 h before use.

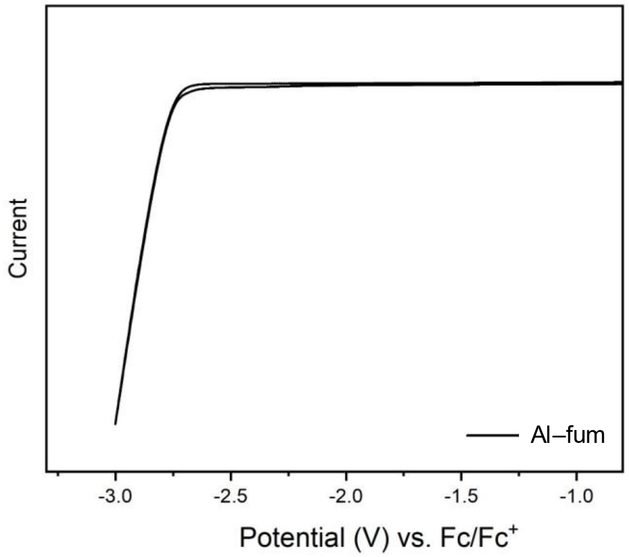

Figure S36. CV of Al–fum in electrolyte solution of $TBAPF_6$ (0.1 M) in DMF in a N_2-filled glovebox with Fc as an internal reference. Working electrode: glassy carbon; counter electrode: platinum wire, reference electrode: Ag/Ag^+ (0.01 M $AgNO_3$ in 0.1 M $TBAPF_6$/DMF). Scan rate: 50 mV/s. No detectable signal within the solvent window was observed, highlighting the lack of redox activity of this material.

11. Kinetic Studies.

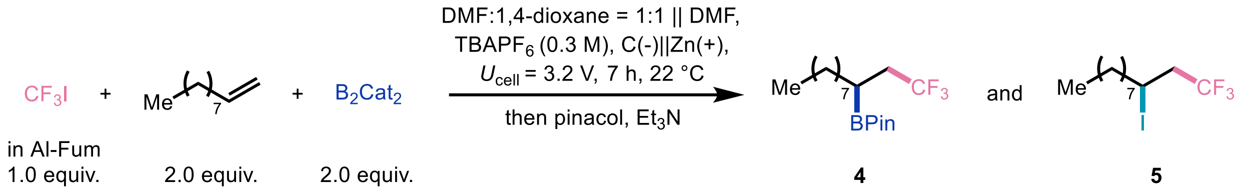

The reaction was set up as described in **General Procedure A** and quenched at various times (1, 2, 3, 4, 5, 6, and 7 h). The NMR yield of the reaction was determined by ^{19}F NMR ($PhCF_3$ as internal reference). Signals corresponding to products **4** and **5** were identified by comparison with authentic samples.

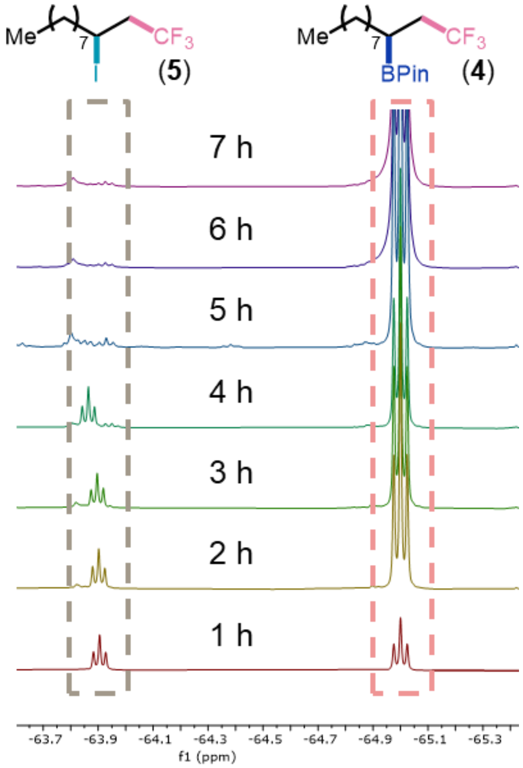

Figure S37. ^{19}F NMR spectra of the reaction mixture with different reaction times.

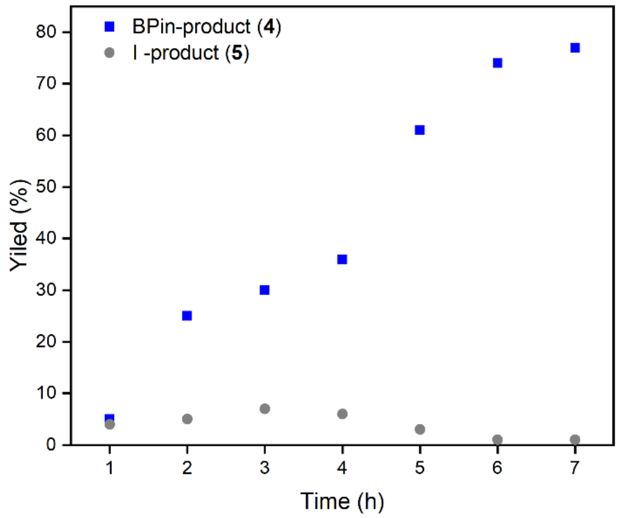

Figure S38. Yield vs. reaction time determined by ^{19}F NMR for representative RXN 1. Yield of BPin product **4** (blue); yield of iodinated product **5** (grey).

The iodinated product **5** was observed during the first 4 h and then gradually decreased, while the Bpin product **4** formed more rapidly between 4–7 h. These results suggest **5** forms under the reaction conditions, likely via an atom transfer radical addition (ATRA) pathway, and subsequently converts to the Bpin product **4**.

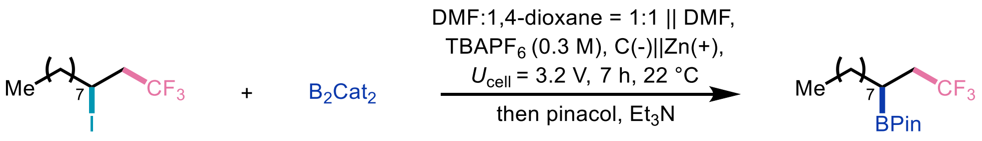

To further confirm this hypothesis, isolated **5** was subjected to the standard reaction conditions in place of CF_3I, which afforded 27% of the borylated product **4** after 14 h. Together, these data support that, while **5** can form under the reaction conditions via an ATRA process, any that forms is consumed by subsequent borylation to produce the desired product **4**.

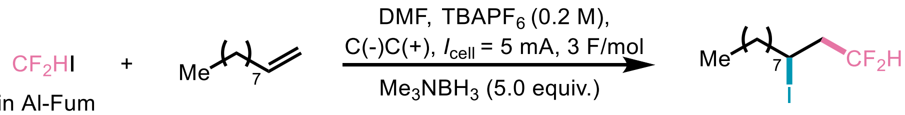

The reaction was set up as described in **General Procedure D** and quenched at specified charges (0.5, 1, 1.5, 2, 2.5, 3, and 3.5 F/mol). The NMR yield of the reaction was determined by ^{19}F NMR ($PhCF_3$ as internal reference).

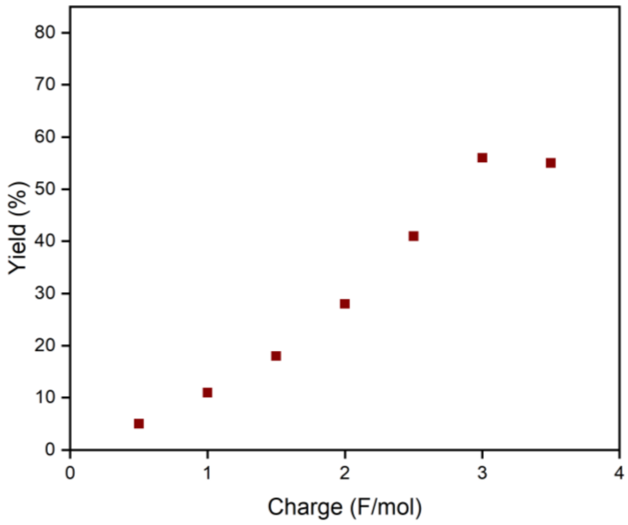

Figure S39. Yield vs. reaction time determined by ^{19}F NMR for representative RXN 2.

12. Three-Electrode Measurement to Determine Working Electrode Potentials.

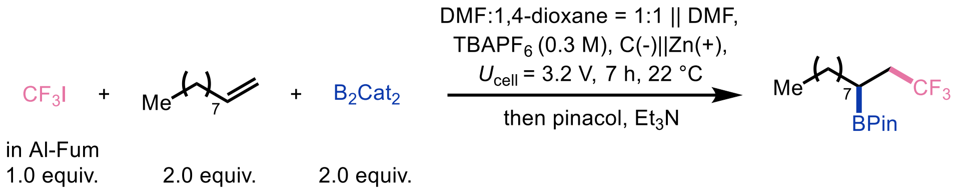

Following **General Procedure A**, in a N_2-filled glovebox, an oven-dried 10 mL H-cell was equipped with a Zn plate anode, a carbon felt cathode, and two additional reference electrodes (Ag) for both the cathodic and anodic chamber. B_2Cat_2 (190.2 mg, 0.80 mmol, 2.00 equiv.) and $TBAPF_6$ (232.5 mg, 0.60 mmol, 1.50 equiv.) were combined in the cathodic chamber, while the anodic chamber was charged with $TBAPF_6$ (232.5 mg, 0.60 mmol, 1.50 equiv.). Then, anhydrous degassed 1,4-dioxane (1.0 mL) and DMF (1.0 mL) were added to the cathodic chamber, while the anodic chamber was charged with DMF (2.0 mL). Subsequently, 1-decene (151 µL, 0.80 mmol, 2.00 equiv.) and CF_3I–Al–fum (188.0 mg, 0.40 mmol, 1.00 equiv.) were added to the cathodic chamber. Then, the reaction mixture was stirred at a constant cell potential of 3.2 V at room temperature (Figure S40). The potential difference between the cathode or the anode and the reference electrode was measured continuously using a voltmeter.

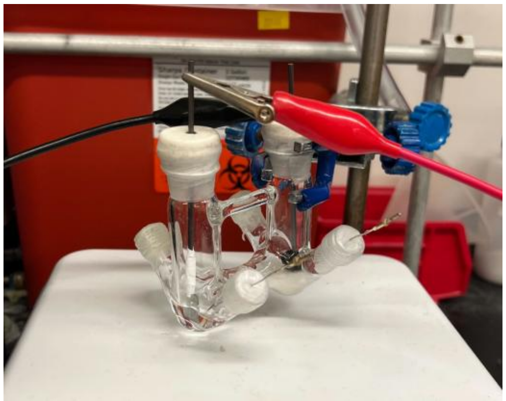

Figure S40. Working electrode potential measuring experiment.

Table S7. Electrode potentials (vs Fc/Fc^+) over time.

Time	Anode potential (V)	Cathode potential (V)
5 min	−0.34	−1.61
15 min	−0.10	−1.48
30 min	−0.15	−1.50
1 h	−0.17	−1.48
2 h	−0.25	−1.47
3 h	−0.29	−1.51
4 h	−0.23	−1.52
5 h	−0.27	−1.50
6 h	−0.27	−1.49
7 h	−0.28	−1.47
7.5 h	−0.26	−1.53
8 h	−0.15	−1.76

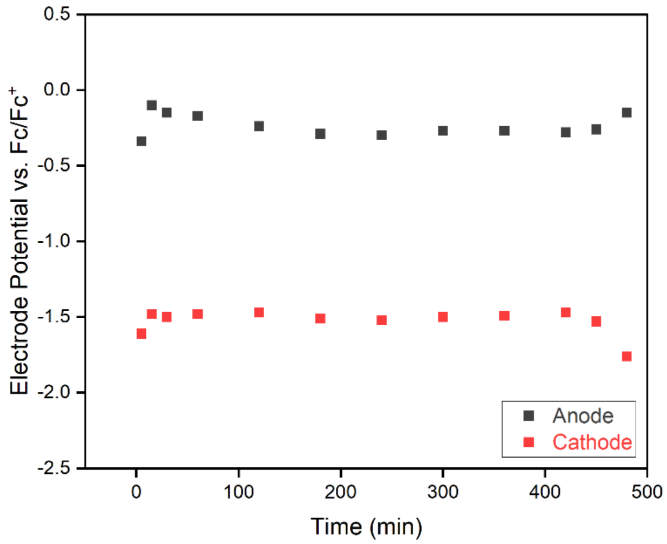

Figure S41. Cathode (red) and anode (black) potentials measured over time during a reaction in progress.

The cathode potential stabilized around −1.50 V vs Fc/Fc^+, and the anode potential stabilized around −0.30 V vs Fc/Fc^+ (Table S7, Figure S41). After 13 h, the yield of the reaction was determined to be 67% by ^{19}F NMR ($PhCF_3$ as internal reference), which is comparable to the yield obtained under the standard reaction conditions (71%).

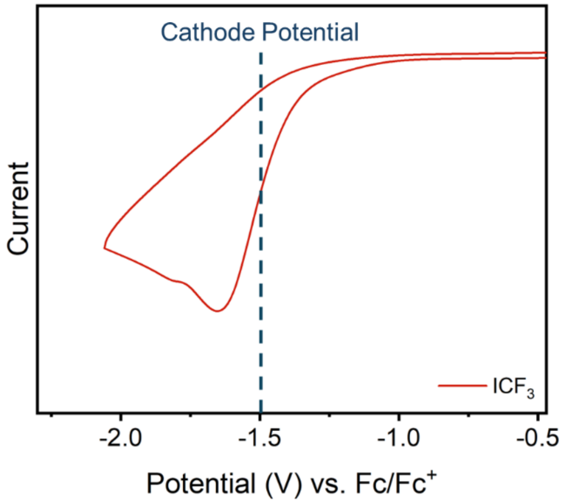

Figure S42. Average cathode potential of a reaction in progress vs. CV of CF_3I.

By comparing the average measured electrode potentials of a reaction in progress with the CVs, we hypothesize that the cathodic reaction is the reduction of CF_3I ($E_{p/2} = -1.36$ V vs Fc/Fc^+), which then generates $CF_3\cdot$ to further react with the alkene substrate, while the anodic reaction is likely the oxidation of Zn to Zn^{2+} ($E_{p/2} = -0.21$ V vs Fc/Fc^+).

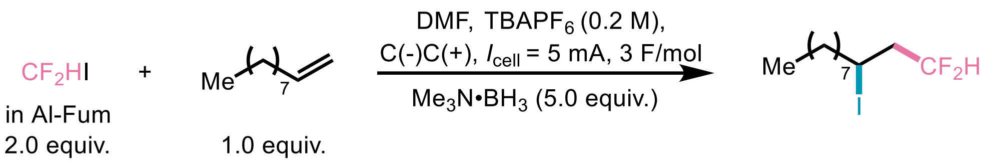

Following **General Procedure D**, in a N_2-filled glovebox, an oven-dried 10 mL 3-neck round-bottom flask was equipped with a stir bar, a Zn plate anode, a carbon felt cathode, and an additional reference electrode (Ag). The flask was charged with $TBAPF_6$ (309.8 mg, 0.80 mmol, 2.00 equiv.), $Me_3N{\cdot}BH_3$ (145.9 mg, 2.00 mmol, 5.00 equiv.) and anhydrous degassed DMF (4.0 mL). Then, 1-decene (151 µL, 0.80 mmol, 2.00 equiv.) and CF_2HI–Al–fum (134.8 mg, 0.80 mmol, 2.00 equiv.) were added sequentially. The vial was sealed, transferred out of the glovebox, and stirred at a constant cell current of 5 mA at 22 ºC for 13 h. The potential difference between the cathode or the anode and the reference electrode was measured continuously using a voltmeter (Figure S43).

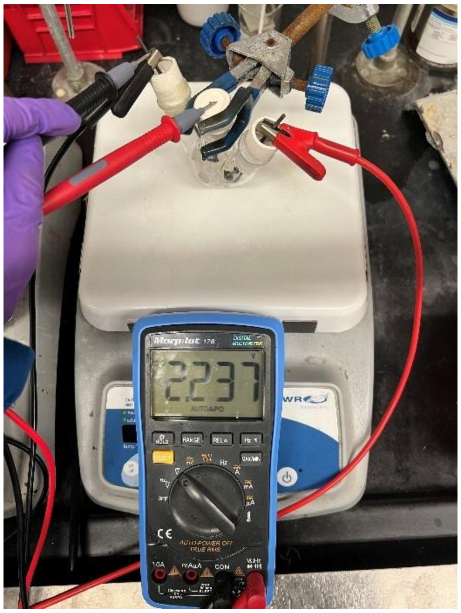

Figure S43. Working electrode potential measuring experiment.

Table S8. Electrode potentials (vs Fc/Fc^+) over time.

Time	Anode potential (V)	Cathode potential (V)
2 min	+2.56	−2.16
5 min	+2.61	−2.29
20 min	+1.59	−2.60
40 min	+1.46	−2.63
1 h	+1.33	−2.71
2 h	+1.29	−2.80
3 h	+1.39	−2.79
5 h	+1.16	−2.91
7 h	+1.28	−2.84
9 h	+1.40	−2.89
11 h	+1.12	−2.90
13 h	+1.15	−2.88

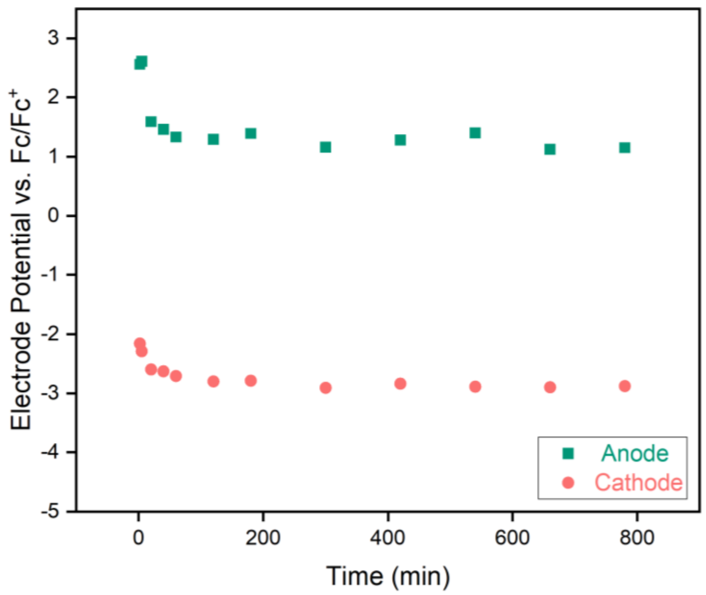

Figure S44. Cathode (red) and anode (green) potential measured over time during a reaction in progress.

The cathode potential stabilized around −2.80 V vs Fc/Fc^+, and the anode potential stabilized around +1.32 V vs Fc/Fc^+ (Table S8, Figure S44). After 13 h, the yield of the reaction was determined to be 45% by ^{19}F NMR ($PhCF_3$ as internal reference), which is comparable to the yield obtained under the standard reaction conditions (54%).

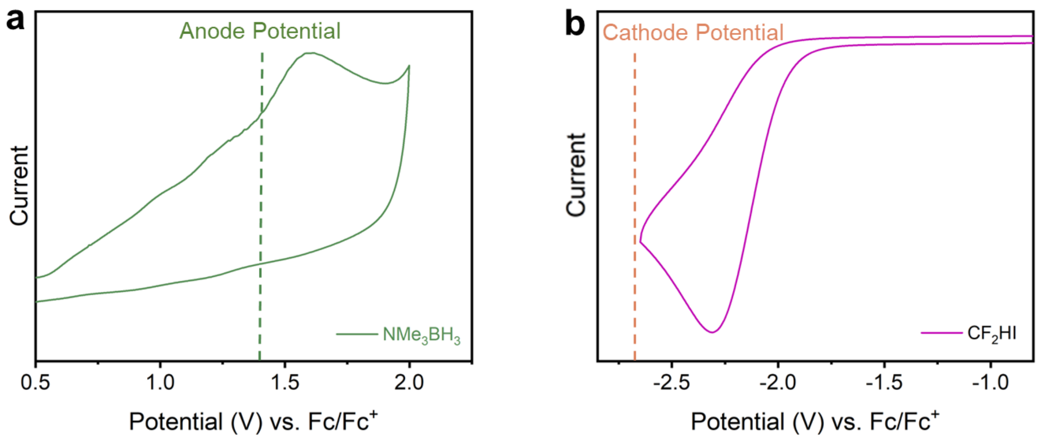

Figure S45. a) Average anode potential of reaction in progress vs. CV of $Me_3N{\cdot}BH_3$. b) Average cathode potential of reaction in progress vs. CV of CF_2HI.

By comparing the average measured electrode potentials of a reaction in progress with the CVs, we hypothesize that the anodic reaction is likely the oxidation of $Me_3N{\cdot}BH_3$ ($E_{p/2}$ = +1.18 V vs Fc/Fc^+), while the cathodic reaction is the reduction of CF_2HI ($E_{p/2}$ = −2.05 V vs Fc/Fc^+), which then generates $CF_2H{\cdot}$ to further react with the alkene substrate.

13. UV–Vis Analysis of Anodic Iodide Oxidation.

To probe the proposed anodic oxidation of I^- under the CF_2H-iodination reaction conditions in an undivided cell, we performed UV–Vis monitoring experiments under modified electrolysis conditions. To avoid spectral interference from $Me_3N{\cdot}BH_3$ and its oxidative byproducts, the experiment was conducted in its absence.

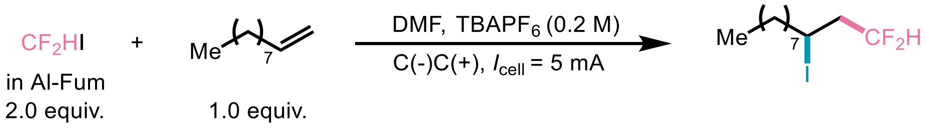

After passage of current, an aliquot from the reaction mixture was analyzed by UV–Vis spectroscopy. Characteristic absorption bands consistent with the triiodide anion (I_3^-) were observed (λ_{max} ≈ 290 nm and 360 nm). Importantly, no I_3^- absorption features were detected in a control experiment conducted under identical conditions without applied potential. These results indicate that I_3^- is generated only under electrolysis conditions, consistent with anodic oxidation of I^- during the reaction. It is likely that I_3^- is the source of I in the reaction product.

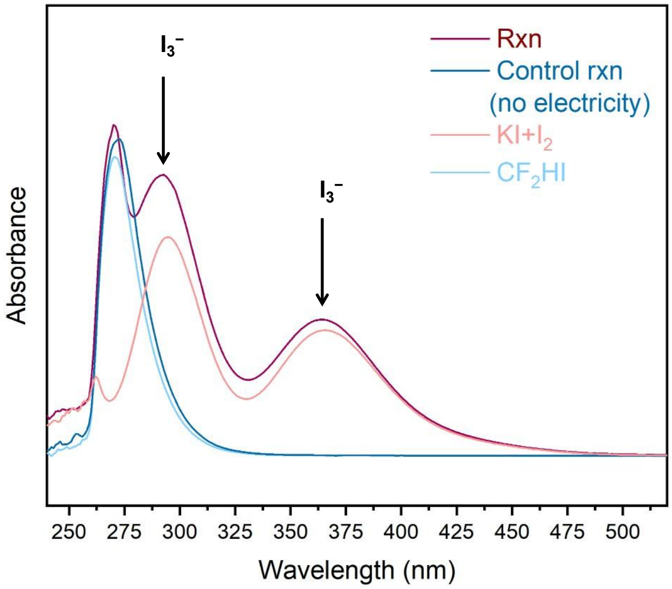

Figure S46. UV–vis spectra of a reaction aliquot (dark red), control reaction mixture without applied potential (dark blue), I_3^- solution prepared by mixing 2.0 equiv. KI and 1.0 equiv. I_2 in DMF (light red), and CF_2HI in DMF (light blue).

14. Proposed Mechanisms.

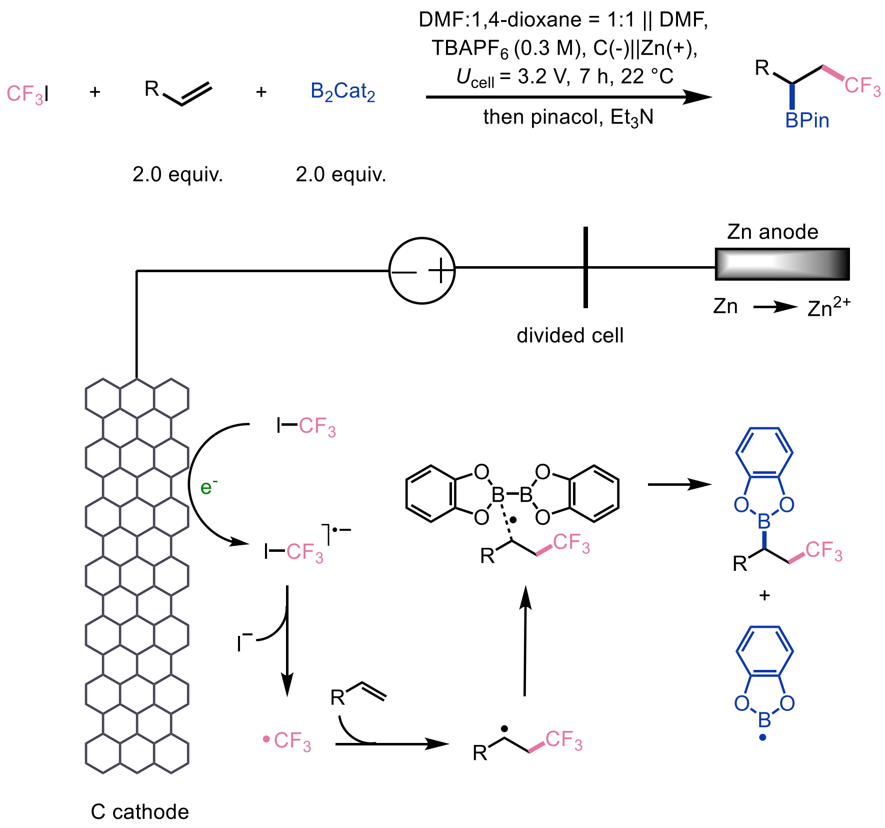

Figure S47. Proposed mechanism for RXN 1.

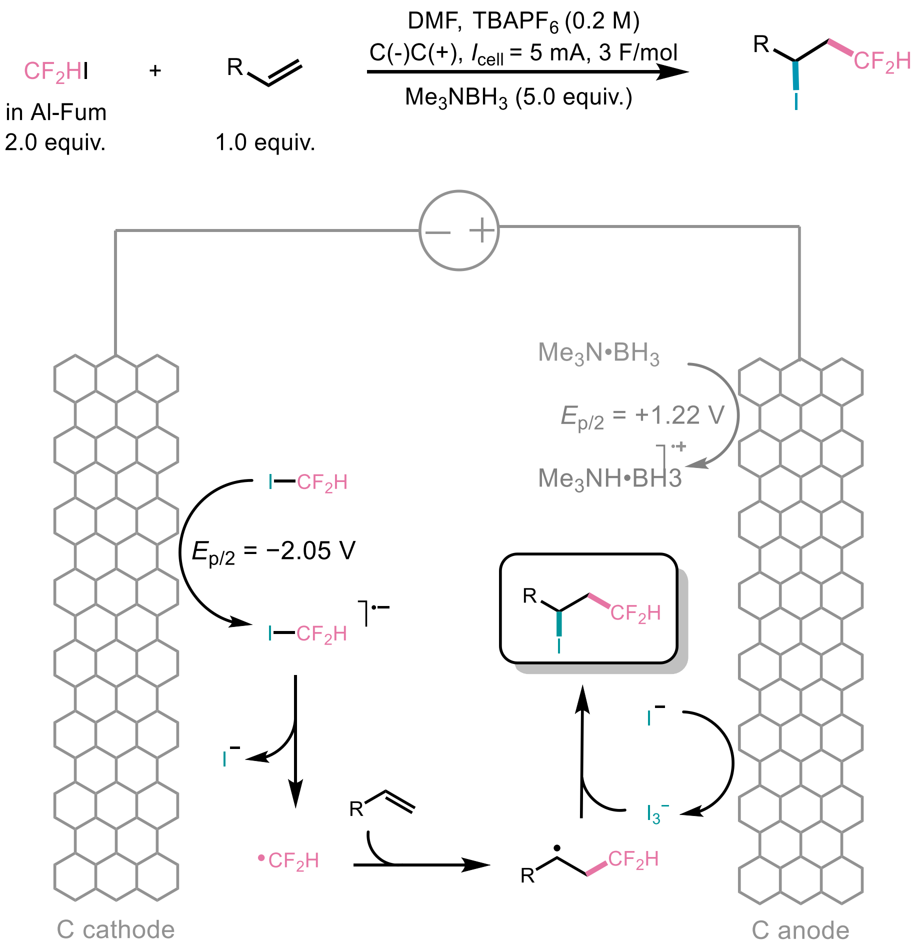

Figure S48. Proposed mechanism for RXN 2.

15. Substrates with Diminished Reactivity in CF2H–Iodination.

Table S9. Unsuccessful substrates in CF_2H–iodination.

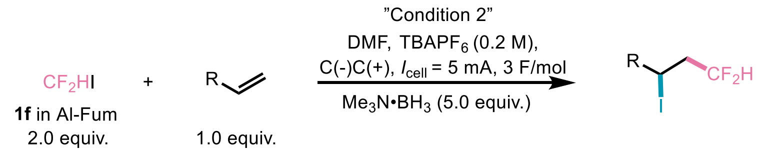

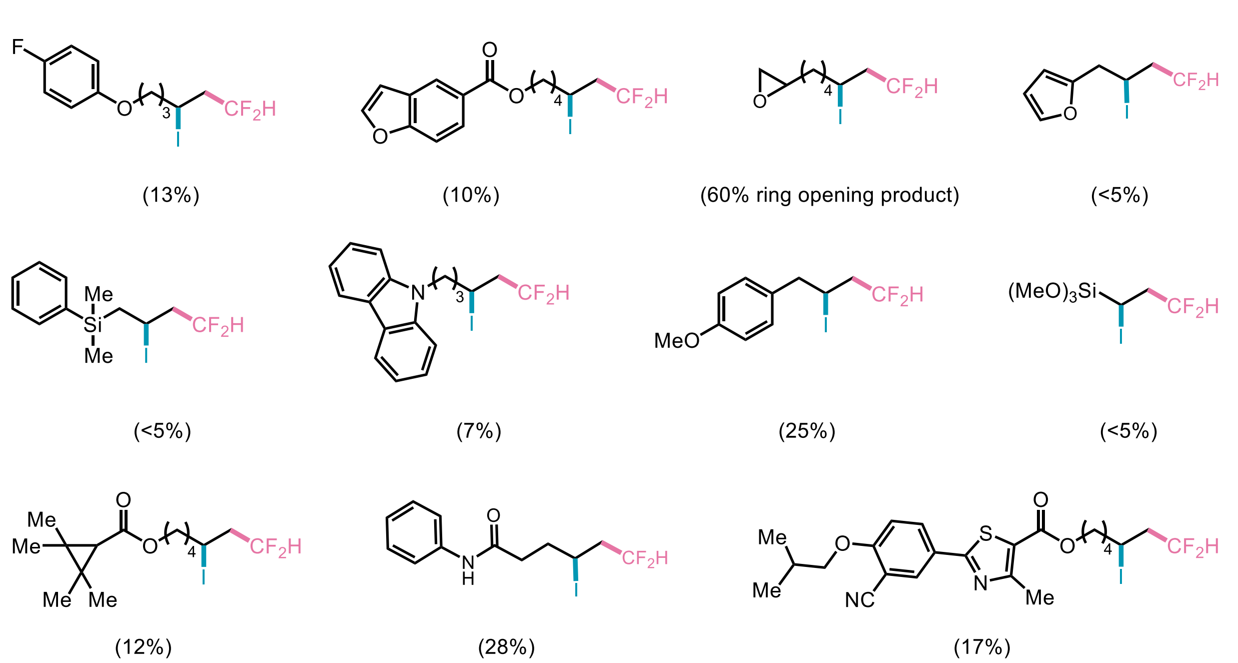

[a] "Condition 2": **1f** (in Al–fum, 0.8 mmol, 2.0 equiv.), alkene (1.0 equiv.), $Me_3N{\cdot}BH_3$ (5.0 equiv.) $TBAPF_6$ (0.3 M) and DMF (4.0 mL). [b] ^{19}F NMR yields are shown.

In contrast to the broad scope observed for CF_3–borylation, several substrates afforded diminished yields under the standard electroreductive conditions for the CF_2H-iodination reaction. In particular, alkenes containing heteroaromatic motifs such as furan, benzofuran, thiazole, and carbazole exhibited low conversion. Substrates bearing epoxide, cyclopropane, or amide functionalities likewise showed reduced efficiency. Additionally, silicon-containing substrates delivered only trace amounts of the desired products. The method is currently most effective for terminal, unactivated alkenes lacking strongly coordinating or easily reducible functionalities. Further development aimed at expanding the substrate scope for CF_2H incorporation is ongoing.

16. Porous Materials as Additives in Reactions.

Following **General Procedure C** (RXN 1) or **General Procedure E** (RXN 2), different porous materials were incorporated as additives in electrochemical reactions to examine their compatibility. By comparing the reaction yields obtained with and without the addition of porous materials (Figure S49, S50), we assessed whether specific materials interfered with the reaction. After each experiment, the porous materials were filtered off and characterized by PXRD to evaluate the retention of their crystallinity (if any solid was recovered).

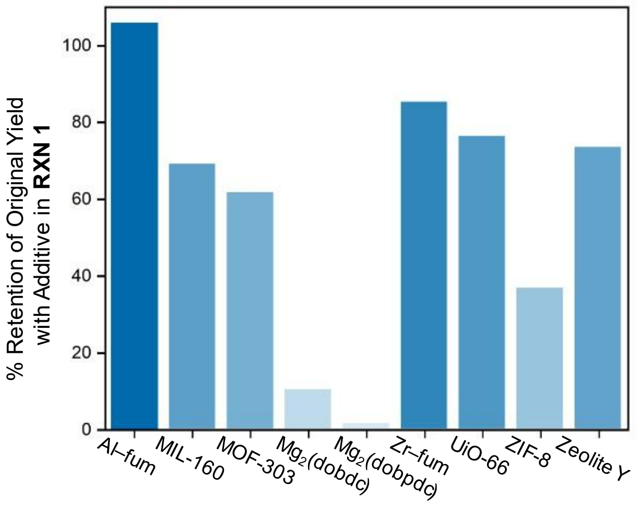

Figure S49. Yields of RXN 1 with different porous materials as additives.

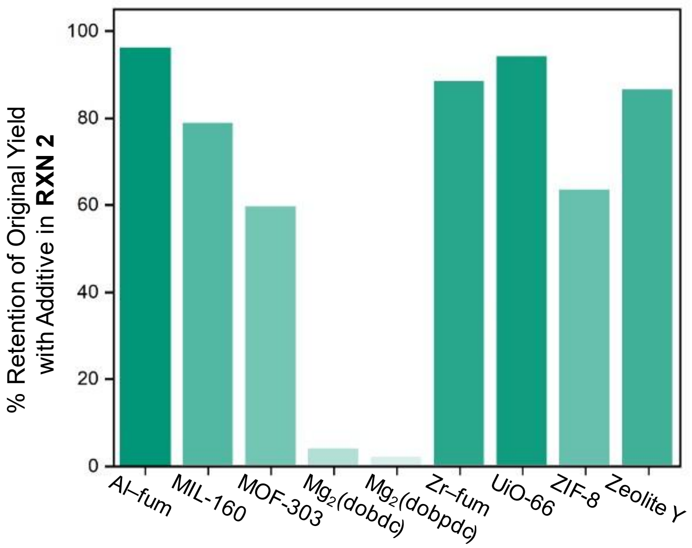

Figure S50. Yields of RXN 2 with different porous materials as additives.

Al–Fum: The ^{19}F NMR yields of RXN 1 and RXN 2 in the presence of Al–fum were 72% and 50%, respectively. Compared to the reactions without any additive, the corresponding retention of yields were 106% and 96%. These results indicate that Al–fum is compatible with both reactions. PXRD analysis of the recovered materials confirmed that the crystallinity was retained after both reactions, highlighting the potential of Al–fum as a robust platform for the delivery of fluorinated gases under electrochemical conditions.

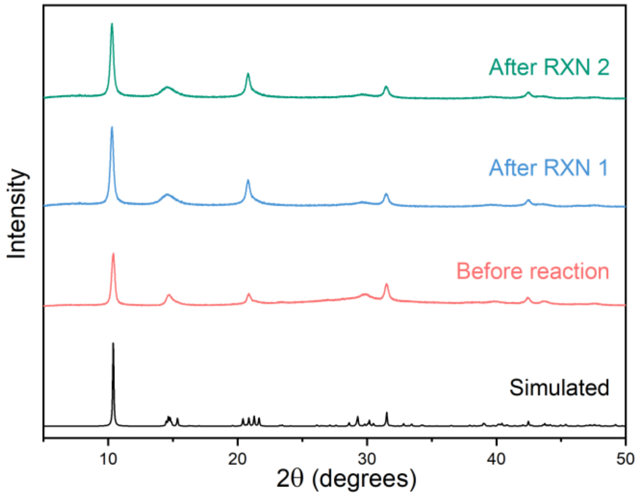

Figure S51. PXRD patterns (Cu Kα radiation, λ = 1.5406 Å) of Al–fum before and after **General Procedure C** (RXN 1) or **General Procedure E** (RXN 2).

MIL-160: The ^{19}F NMR yields of RXN 1 and RXN 2 in the presence of MIL-160 were 47% and 41%, respectively. Compared to the reactions without any additive, the corresponding retention of yields were 69% and 79%. PXRD analysis of the recovered materials revealed that crystallinity was retained after RXN 1, whereas significant loss of crystallinity was observed after RXN 2.

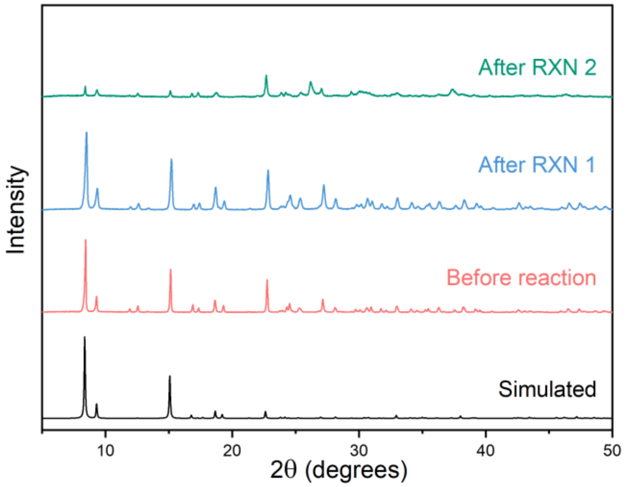

Figure S52. PXRD patterns (Cu Kα radiation, λ = 1.5406 Å) of MIL-160 before and after **General Procedure C** (RXN 1) or **General Procedure E** (RXN 2).

MOF-303: The ^{19}F NMR yields of RXN 1 and RXN 2 in the presence of MOF-303 were 42% and 31%, respectively. Compared to the reactions without any additive, the corresponding retention of yields were 62% and 60%. PXRD analysis of the recovered materials confirmed that crystallinity was retained after both reactions.

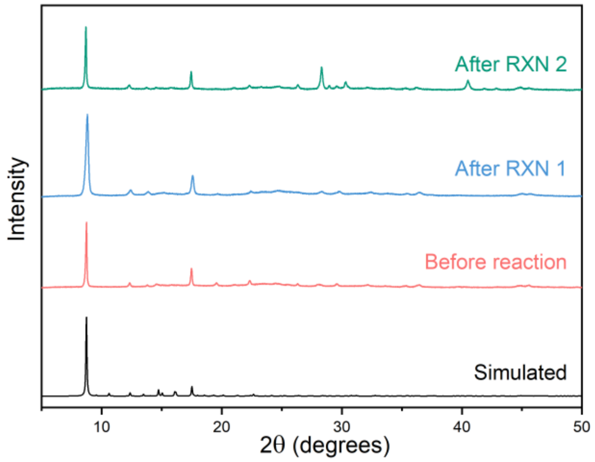

Figure S53. PXRD patterns (Cu Kα radiation, λ = 1.5406 Å) of MOF-303 before and after **General Procedure C** (RXN 1) or **General Procedure E** (RXN 2).

Mg_2(dobdc): The ^{19}F NMR yields of RXN 1 and RXN 2 in the presence of Mg_2(dobdc) were 7% and 2%, respectively. Compared to the reactions without any additive, the corresponding retention of yields were 10% and 4%. PXRD analysis of the recovered materials revealed that crystallinity was retained after RXN 1, whereas the material was not recovered after RXN 2. This material significantly inhibits electrochemical reactions, likely due to its redox-active linker.[28]

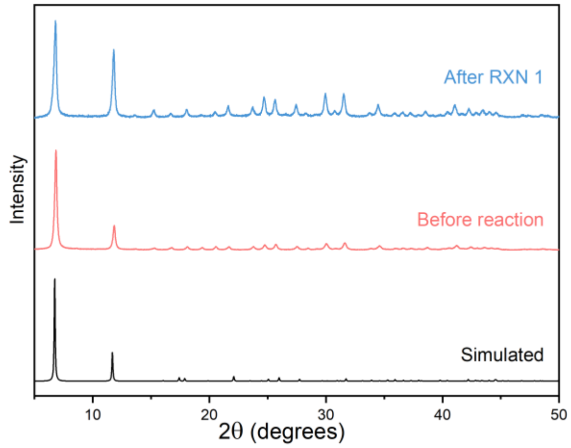

Figure S54. PXRD patterns (Cu Kα radiation, λ = 1.5406 Å) of Mg_2(dobdc) before and after **General Procedure C** (RXN 1). No solid was recovered after **General Procedure E** (RXN 2).

Mg_2(dobpdc): The ^{19}F NMR yields of RXN 1 and RXN 2 in the presence of Mg_2(dobpdc) were 1% and 1%, respectively. Compared to the reactions without any additive, the corresponding retention of yields were 1% and 2%. No material was recovered after either reaction. This material significantly inhibits electrochemical reactions, likely due to its redox-active linker.[28]

Zr–fum: The ^{19}F NMR yields of RXN 1 and RXN 2 in the presence of Zr–fum were 58% and 46%, respectively. Compared to the reactions without any additive, the corresponding retention of yields were 85% and 88%. PXRD analysis of the recovered materials confirmed that crystallinity was retained after both reactions.

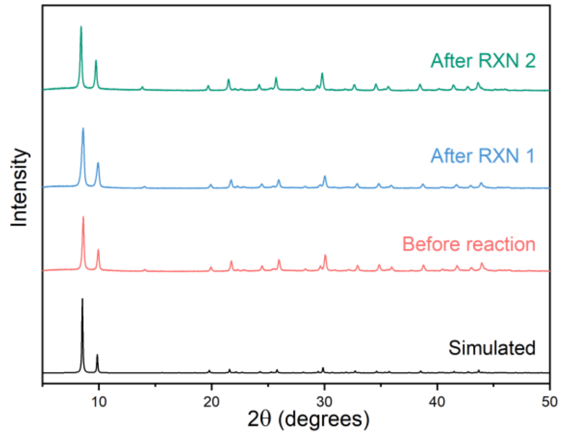

Figure S55. PXRD patterns (Cu Kα radiation, λ = 1.5406 Å) of Zr–fum before and after **General Procedure C** (RXN 1) or **General Procedure E** (RXN 2).

UiO-66: The ^{19}F NMR yields of RXN 1 and RXN 2 in the presence of UiO-66 were 52% and 49%, respectively. Compared to the reactions without any additive, the corresponding retention of yields

were 76% and 94%. PXRD analysis of the recovered materials confirmed that crystallinity was retained after both reactions.

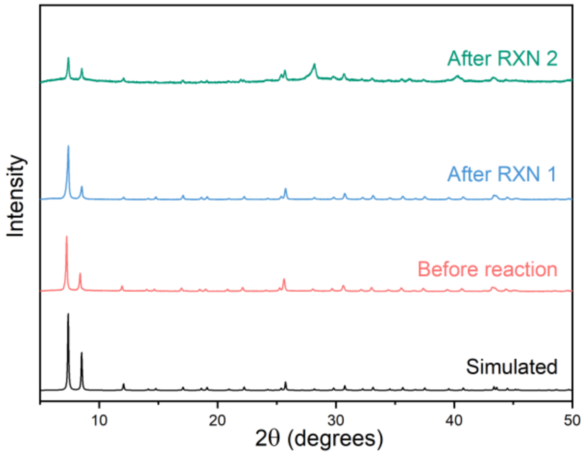

Figure S56. PXRD patterns (Cu Kα radiation, λ = 1.5406 Å) of UiO-66 before and after **General Procedure C** (RXN 1) or **General Procedure E** (RXN 2).

ZIF-8: The ^{19}F NMR yields of RXN 1 and RXN 2 in the presence of ZIF-8 were 25% and 33%, respectively. Compared to the reactions without any additive, the corresponding retention of yields were 37% and 63%. PXRD analysis of the recovered materials confirmed that crystallinity was retained after both reactions.

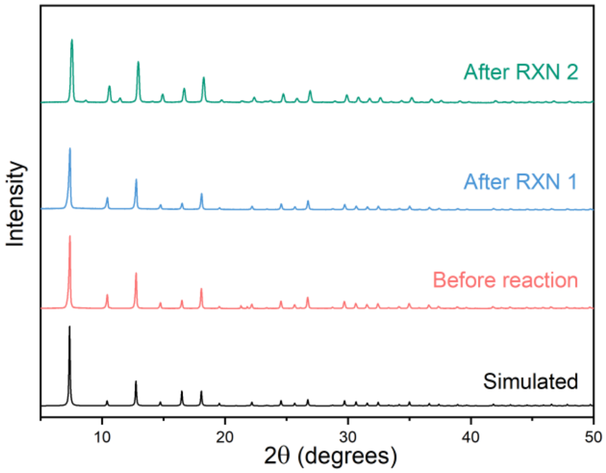

Figure S57. PXRD patterns (Cu Kα radiation, λ = 1.5406 Å) of ZIF-8 before and after **General Procedure C** (RXN 1) or **General Procedure E** (RXN 2).

Zeolite Y, Sodium: The ^{19}F NMR yields of RXN 1 and RXN 2 in the presence of Zeolite Y were 50% and 45%, respectively. Compared to the reactions without any additive, the corresponding

retention of yields were 74% and 87%. PXRD analysis of the recovered materials revealed that crystallinity was retained after RXN 1, whereas the material was not recovered after RXN 2.

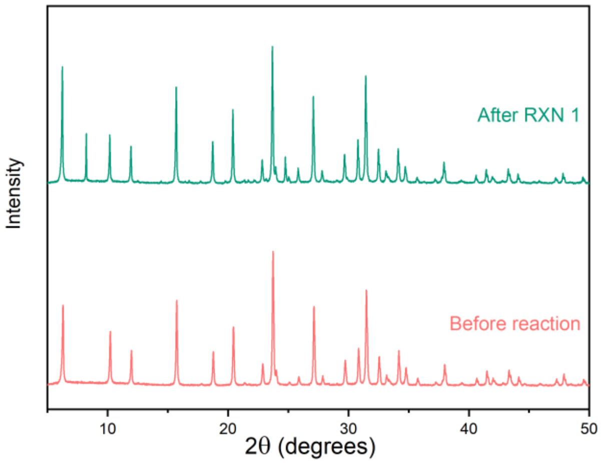

Figure S58. PXRD patterns (Cu Kα radiation, λ = 1.5406 Å) of Zeolite Y before and after **General Procedure C** (RXN 1). No solid was recovered after **General Procedure E** (RXN 2).

17. Procedures for Derivation Reactions.

1,1,1-Trifluoro-5-phenylpentan-3-ol (51):

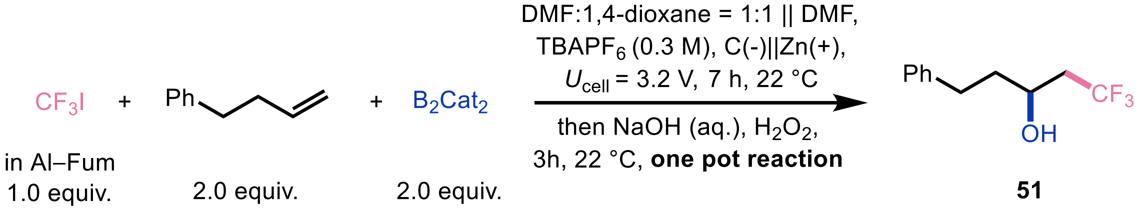

Following General Procedure A, B_2Cat_2 (190.2 mg, 0.80 mmol, 2.00 equiv.) and $TBAPF_6$ (232.5 mg, 0.60 mmol, 1.50 equiv.) were combined in the cathodic chamber of the H-cell, while the anodic chamber was charged with $TBAPF_6$ (232.5 mg, 0.60 mmol, 1.50 equiv.). Then, anhydrous degassed 1,4-dioxane (1.0 mL) and DMF (1.0 mL) were added to the cathodic chamber, while the anodic chamber was charged with DMF (2.0 mL). Subsequently, but-3-en-1-ylbenzene (117 µL, 0.80 mmol, 2.00 equiv.) and CF_3I–Al–fum (188.0 mg, 0.40 mmol, 1.00 equiv.) were added to the cathodic chamber. Then, the reaction mixture was stirred at a constant cell potential of 3.2 V at room temperature for 7 h. After completion of the reaction, a 2:1 (v:v) mixture of 3 M aq. NaOH (4 mL) and H_2O_2 (30% aqueous solution, 2 mL) was prepared at 0 °C and added to the cathodic chamber. The mixture was stirred for an additional 3 h. The reaction mixture was extracted with EtOAc (3 × 10 mL), and the combined organic phases were dried over Na_2SO_4 and concentrated under reduced pressure. The crude product was purified by flash chromatography (SiO_2, gradient of 0% → 30% EtOAc in hexane) to afford **51** (53.2 mg, 61%) as a colorless oil.

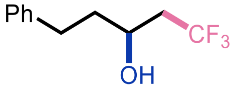

1,1,1-trifluoro-5-phenylpentan-3-ol (51)

^{1}H NMR (500 MHz, $CDCl_3$): δ 7.30 (dd, J = 8.5, 6.8 Hz, 2H), 7.22–7.19 (m, 3H), 4.04 (tq, J = 8.1, 3.7 Hz, 1H), 2.85–2.79 (m, 1H), 2.72 (ddd, J = 13.8, 9.0, 7.2 Hz, 1H), 2.38–2.21 (m, 2H), 1.91–1.80 (m, 3H) ppm.

^{13}C NMR (126 MHz, $CDCl_3$): δ 141.91, 128.27, 128.24, 127.13 (q, J = 276.9 Hz), 126.19, 123.98, 66.58, 34.98 (q, J = 28.0 Hz), 34.88, 32.83, 24.68, 24.60 ppm.

^{19}F NMR (470 MHz, $CDCl_3$): δ −63.41 (t, J = 10.9 Hz) ppm.

The NMR data are consistent with the reported data.[29]

MS (DART) m/z: $[M+H]^+$ Calcd. for $C_{11}H_{14}F_3O^+$: 219.0991; Found: 219.0976.

(5,5,5-Trifluoropentyl)benzene (52):

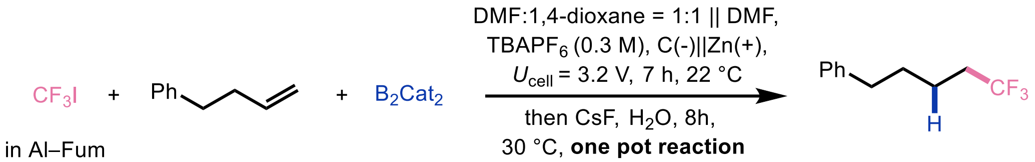

52

Following General Procedure A, B_2Cat_2 (190.2 mg, 0.80 mmol, 2.00 equiv.) and $TBAPF_6$ (232.5 mg, 0.60 mmol, 1.50 equiv.) were combined in the cathodic chamber of the H-cell, while the anodic chamber was charged with $TBAPF_6$ (232.5 mg, 0.60 mmol, 1.5 equiv.). Then, anhydrous degassed 1,4-dioxane (1.0 mL) and DMF (1.0 mL) were added to the cathodic chamber, while the anodic chamber was charged with DMF (2.0 mL). Subsequently, but-3-en-1-ylbenzene (117 µL, 0.80 mmol, 2.00 equiv.) and CF_3I–Al–fum (188.0 mg, 0.40 mmol, 1.00 equiv.) were added to the cathodic chamber. Then, the reaction mixture was stirred at a constant cell potential of 3.2 V at room temperature for 7 h. After completion of the reaction, CsF (91.1 mg, 0.60 mmol, 1.50 equiv.) and H_2O (10.8 µL, 0.60 mmol, 1.50 equiv.) were added to the cathodic chamber, and the mixture was stirred for another 8 h. The reaction mixture was concentrated under reduced pressure, and the crude product was purified by flash chromatography (SiO_2, gradient of 0% → 10% Et_2O in pentane) to afford **52** (42.8 mg, 53%) as a colorless oil.

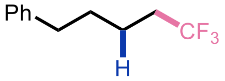

(5,5,5-Trifluoropentyl)benzene (52)

^{1}H NMR (500 MHz, $CDCl_3$): δ 7.35–7.30 (m, 2H), 7.25–7.15 (m, 3H), 2.89–2.58 (m, 2H), 2.45–2.23 (m, 2H), 1.97–1.82 (m, 2H), 1.43–1.26 (m, 2H) ppm.

^{13}C NMR (126 MHz, $CDCl_3$): δ 142.16, 128.54, 128.53, 128.49, 127.58 (q, J = 276.9 Hz), 126.03, 35.24 (q, J = 27.9 Hz), 35.13, 33.08, 24.93, 24.86 ppm.

^{19}F NMR (470 MHz, $CDCl_3$): δ −66.21 (t, J = 11.2 Hz) ppm.

The NMR data are consistent with the reported data.[30]

MS (DART) m/z: $[M+H]^+$ Calcd. for $C_{11}H_{14}F_3^+$: 203.1042; Found: 203.1066.

Trifluoro(1,1,1-trifluoro-5-phenylpentan-3-yl)-l4-borane, potassium salt (53):

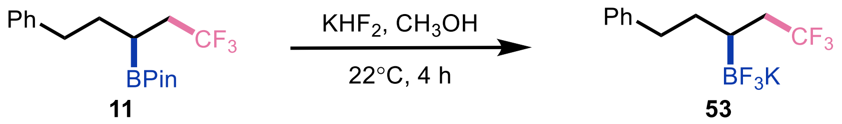

Compound **53** was prepared from isolated **11** following a modified literature procedure.[31] In an oven-dried 50 mL round-bottom flask, **11** (131.3 mg, 0.40 mmol, 1.00 equiv.) was dissolved in CH_3CN (5 mL), and saturated aq. potassium hydrogen fluoride solution (4.5 M, 0.5 mL, 2.4 mmol, 6.0 equiv.) was added. After stirring for 3 h at room temperature, the solvent was removed under reduced pressure. The residue was dissolved in acetone (5.0 mL), and the insoluble solid was removed by filtration. The filtrate was concentrated under reduced pressure. The resulting solid was rinsed with Et_2O (2 × 5 mL) to yield pure product **53** (109.7 mg, 89%) as a white solid.

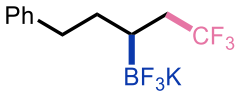

Trifluoro(1,1,1-trifluoro-5-phenylpentan-3-yl)-l4-borane, potassium salt (53)

^{1}H NMR (500 MHz, DMSO): δ 7.11 (dd, J = 8.3, 6.9 Hz, 2H), 7.01–6.99 (m, 3H), 2.50 (ddd, J = 13.4, 10.6, 6.0 Hz, 1H), 2.43–2.37 (m, 1H), 2.09–1.95 (m, 1H), 1.70 (ddt, J = 22.6, 15.4, 12.0 Hz, 1H), 1.43 (dp, J = 12.2, 6.3 Hz, 2H), 0.39 (s, 1H) ppm.

^{13}C NMR (126 MHz, $CDCl_3$): δ 144.85, 131.32, 129.10, 128.75, 128.64, 128.58, 128.55, 128.49, 128.41, 125.48, 40.46, 40.38, 40.29, 40.22, 40.13, 40.05, 39.96, 39.79, 39.71, 39.63, 39.46, 35.01, 34.78, 33.60, 25.41 ppm.

^{19}F NMR (470 MHz, $CDCl_3$): δ −62.11 (t, J = 12.9 Hz, 3F), −142.97 (s, 3F) ppm.

^{11}B NMR (160 MHz, $CDCl_3$): δ 4.46 ppm.

MS (DART) m/z: $[M+H]^+$ Calcd. for $C_{11}H_{13}BF_6K^+$: 309.0646; Found: 309.0606.

4,4,5,5-Tetramethyl-2-(4,4,4-trifluoro-2-phenethylbutyl)-1,3,2-dioxaborolane (54):

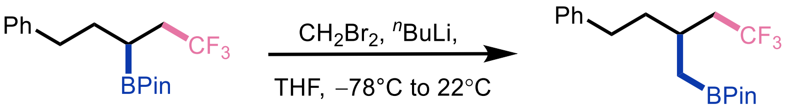

11 **54**

Compound **54** was prepared from isolated **11** following a modified literature procedure.[31] In an oven-dried 25 mL round-bottom flask, **11** (131.3 mg, 0.40 mmol, 1.00 equiv.) was dissolved in THF (3.0 mL). Dibromomethane (70.2 µL, 1.00 mmol, 2.50 equiv.) was added, and the mixture was cooled to −78 °C using a dry ice/acetone bath. *ⁿ*BuLi (2M in hexane, 0.30 mL, 0.60 mmol, 1.50 equiv.) was added to the solution dropwise, and the reaction mixture was stirred at −78 °C for 30 min before being allowed to warm to room temperature. After stirring for an additional 2 h, the reaction was quenched with saturated aqueous NH_4Cl (10.0 mL). The aqueous phase was extracted with Et_2O (3 × 15.0 mL), and the combined organic extracts were dried over Na_2SO_4. The solution was concentrated under reduced pressure, and the crude product was purified by flash chromatography (SiO_2, gradient of 0% → 5% Et_2O in pentane) to afford **54** (80.7 mg, 59%) as a colorless oil.

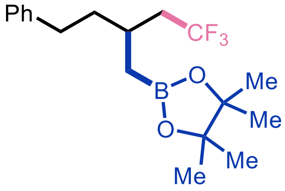

4,4,5,5-Tetramethyl-2-(4,4,4-trifluoro-2-phenethylbutyl)-1,3,2-dioxaborolane (54)

^{1}H NMR (500 MHz, $CDCl_3$): δ 7.31–7.28 (m, 2H), 7.22–7.18 (m, 3H), 2.85–2.69 (m, 2H), 2.36–2.26 (m, 1H), 1.88–1.83 (m, 2H), 1.56 (s, 12H), 1.33–1.24 (m, 2H), 0.95–0.84 (m, 2H) ppm.

^{13}C NMR (126 MHz, $CDCl_3$): δ 142.16, 128.65, 128.53, 128.49, 128.43, 127.49 (q, J = 276.8 Hz), 126.44, 83.79, 35.21 (q, J = 27.8 Hz), 35.13, 33.08 ppm.

^{19}F NMR (470 MHz, $CDCl_3$): δ −62.62 (t, J = 10.9 Hz) ppm.

^{11}B NMR (160 MHz, $CDCl_3$): δ 34.23 ppm.

MS (DART) m/z: $[M+H]^+$ Calcd. for $C_{18}H_{27}BF_3O_2^+$: 343.2051; Found: 343.2033.

2-(1,1-Difluoro-5-phenylpentan-3-yl)-4,4,5,5-tetramethyl-1,3,2-dioxaborolane (55):

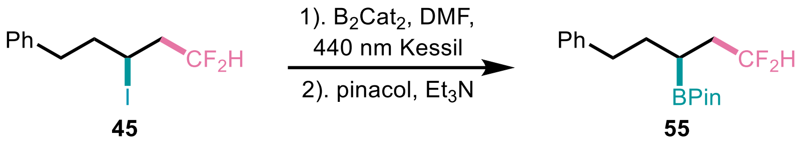

Compound **55** was prepared from isolated **45** following a modified literature procedure.[32] An oven-dried 8 mL screw-cap septum vial equipped with a stir bar was charged with B_2Cat_2 (190.2 mg, 0.80 mmol, 4.00 equiv.). The vial was transferred into an N_2-filled glovebox, where **45** (62.0 mg, 0.20 mmol, 1.00 equiv.) and anhydrous DMF (0.60 mL) were added. The vial was sealed with PTFE tape and transferred out of the glovebox. The reaction mixture was stirred under blue LED irradiation (40 W, 440 nm, 100% intensity) with a fan for 24 h. A solution of pinacol (95.0 mg, 0.80 mmol, 4.00 equiv.) in triethylamine (0.70 mL) was added to the mixture, and it was stirred for 1 h. After the reaction was complete, the reaction mixture was diluted with CH_2Cl_2 (15.0 mL) and concentrated under reduced pressure. The crude product was purified by flash chromatography (SiO_2, gradient of 2% → 20% Et_2O in pentane) to afford the desired compound **55** (47.7 mg, 77%) as a colorless oil.

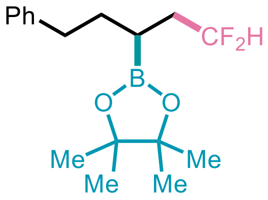

2-(1,1-Difluoro-5-phenylpentan-3-yl)-4,4,5,5-tetramethyl-1,3,2-dioxaborolane (55)

^{1}H NMR (500 MHz, $CDCl_3$): δ 7.29 (d, J = 7.7 Hz, 2H), 7.20–7.16 (m, 3H), 5.87 (tt, J = 57.2, 4.7 Hz, 1H), 2.70–2.57 (m, 2H), 2.11–1.64 (m, 4H), 1.27 (s, 13H) ppm.

^{13}C NMR (126 MHz, $CDCl_3$): δ 142.43, 128.52, 128.49, 125.95, 117.63 (t, J = 238.5 Hz), 83.61, 53.56, 35.68 (t, J = 20.7 Hz), 35.29, 33.35, 24.97, 24.90 ppm.

^{19}F NMR (470 MHz, $CDCl_3$): δ −113.13–−115.67 (m, 2F) ppm.

^{11}B NMR (160 MHz, $CDCl_3$): δ 32.06 ppm.

The NMR data are consistent with the reported data.[33]

MS (DART) m/z: $[M+H]^+$ Calcd. for $C_{17}H_{26}BF_2O_2^+$: 311.1994; Found: 311.2008.

Methyl-4-(1,1-difluoro-5-phenylpentan-3-yl)benzoate (56):

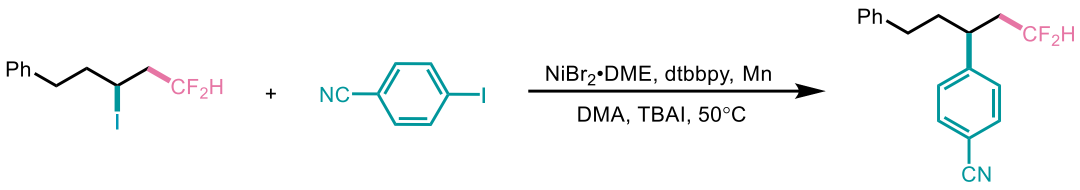

45 **56**

Compound **56** was prepared from isolated **45** following a modified literature procedure.[34] An oven-dried 8 mL screw-cap vial equipped with a magnetic stir bar was charged with 4-iodobenzonitrile (45.8 mg, 0.20 mmol, 1.00 equiv.), $NiBr_2$·DME (6.2 mg, 0.012 mmol, 0.1 equiv.), 4,4'-di-tert-butyl-2,2'-dipyridyl (dtbbpy, 5.3 mg, 0.016 mmol, 0.1 equiv.), Mn (22.0 mg, 0.40 mmol, 2.00 equiv.), and TBAI (73.8 mg, 0.20 mmol, 1.00 equiv.). The vial was transferred into an N_2-filled glovebox, where **45** (62.0 mg, 0.20 mmol, 1.00 equiv.) and anhydrous DMA (2.0 mL) were added. The reaction mixture was allowed to stir for 12 h under N_2 atmosphere at 50 ˚C. After the reaction was complete, the reaction mixture was diluted in CH_2Cl_2 (15.0 mL) and concentrated under reduced pressure. The crude product was purified by flash chromatography (SiO_2, gradient of 2% → 20% Et_2O in pentane) to afford the desired compound **56** (49.0 mg, 86%) as a white solid.

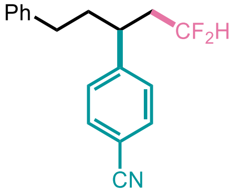

Methyl-4-(1,1-difluoro-5-phenylpentan-3-yl)benzoate (56)

^{1}H NMR (500 MHz, $CDCl_3$): δ 7.63 (d, J = 7.9 Hz, 2H), 7.29 (d, J = 8.0 Hz, 2H), 7.24–7.22 (m, 2H), 7.20–7.12 (m, 1H), 7.04 (d, J = 7.3 Hz, 2H), 5.49 (tdd, J = 56.4, 6.3, 3.4 Hz, 1H), 2.89 (tt, J = 10.0, 5.3 Hz, 1H), 2.42 (t, J = 7.9 Hz, 2H), 2.29–1.87 (m, 4H) ppm.

^{13}C NMR (126 MHz, $CDCl_3$): δ 148.87, 141.05, 132.84, 128.65, 128.57, 128.37, 126.30, 118.81, 116.06 (t, J = 239.5 Hz), 111.11, 40.78 (t, J = 21.0 Hz), 40.05 (dd, J = 7.2, 3.4 Hz), 38.00, 33.37 ppm.

^{19}F NMR (470 MHz, $CDCl_3$): δ −114.65–−117.48 (m, 2F) ppm.

MS (DART) m/z: $[M+H]^+$ Calcd. for $C_{18}H_{18}F_2N^+$: 286.1407; Found: 286.1416.

(E)-(3-(2,2-difluoroethyl)pent-1-ene-1,5-diyl)dibenzene (57):

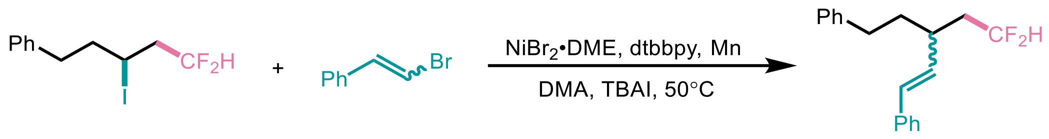

45 57

Compound **57** was prepared from isolated **45** following a modified literature procedure.[34] An oven-dried 8 mL screw-cap vial equipped with a magnetic stir bar was charged with (2-bromovinyl)benzene (*E* : *Z* = 9 : 1, 36.6 mg, 0.20 mmol, 1.0 equiv.), $NiBr_2$·DME (6.2 mg, 0.012 mmol, 0.1 equiv.), dtbbpy (5.3 mg, 0.016 mmol, 0.1 equiv.), Mn (22.0 mg, 0.4 mmol, 2.0 equiv.), and TBAI (73.8 mg, 0.2 mmol, 1.0 equiv.). The vial was transferred into an N_2-filled glovebox, where **45** (62.0 mg, 0.2 mmol, 1.0 equiv.) and anhydrous DMA (2.0 mL) were added. The reaction mixture was allowed to stir for 12 h under N_2 atmosphere at 50 ˚C. After the reaction was complete, the reaction mixture was diluted in CH_2Cl_2 (15.0 mL) and concentrated under reduced pressure. The crude product was purified by flash chromatography (SiO_2, gradient of 0% → 10% Et_2O in pentane) to afford the desired compound **57** (*E* : *Z* = 9 : 1, 37.8 mg, 66%) as a colorless oil.

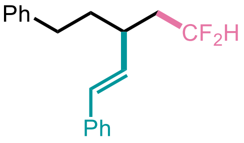

(E)-(3-(2,2-difluoroethyl)pent-1-ene-1,5-diyl)dibenzene (57)

^{1}H NMR (500 MHz, $CDCl_3$): δ 7.32–7.29 (m, 2H), 7.26–7.14 (m, 6H), 7.06 (d, *J* = 6.9 Hz, 2H), 6.60 (d, *J* = 11.7 Hz, 1H), 5.79 (tdd, *J* = 56.7, 6.4, 3.5 Hz, 1H), 5.44 (t, *J* = 11.2 Hz, 1H), 2.99 (ddq, *J* = 14.5, 9.6, 5.0 Hz, 1H), 2.66 (ddd, *J* = 13.8, 10.1, 5.7 Hz, 1H), 2.49 (ddd, *J* = 13.7, 10.1, 6.2 Hz, 1H), 2.07–1.96 (m, 1H), 1.91–1.75 (m, 2H), 1.68–1.61 (m, 1H) ppm.

^{13}C NMR (126 MHz, $CDCl_3$): δ 141.93, 137.11, 134.70, 131.12, 128.55, 128.49, 127.06, 125.98, 116.88 (t, *J* = 238.9 Hz), 39.92 (t, *J* = 20.7 Hz), 37.77, 33.30, 32.10 (dd, *J* = 6.9, 3.8 Hz) ppm.

^{19}F NMR (470 MHz, $CDCl_3$): δ −114.52–−116.50 (m, 2F) ppm.

MS (DART) m/z: $[M+H]^+$ Calcd. for $C_{19}H_{21}F_2^+$: 287.1611; Found: 287.1676.

(5,5-difluoro-3-methylpentyl)benzene (58):

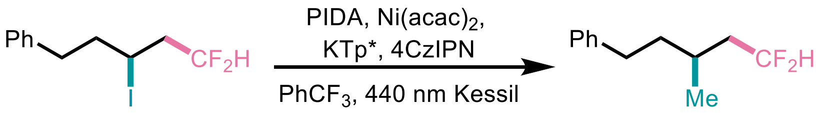

45 **58**

Compound **58** was prepared from isolated **45** following a modified literature procedure.[1] An oven-dried 8 mL screw-cap septum vial equipped with a stir bar was charged with diacetoxyiodobenzene (PIDA, 128.0 mg, 0.40 mmol, 2.0 equiv.) and **45** (62.0 mg, 0.2 mmol, 1.0 equiv.). The vial was transferred into an N_2-filled glovebox, where 4CzIPN (8.0 mg, 10.0 µmol, 5 mol%), $Ni(acac)_2$ (2.8 mg, 10.0 µmol, 5 mol%), and potassium tris(3,5-dimethyl-1-pyrazolyl)borate (KTp*, 3.4 mg, 10.0 µmol, 5 mol%) were sequentially added, followed by anhydrous $PhCF_3$ (2.0 mL, 0.1 M). The tube was sealed with PTFE tape and transferred out of the glovebox. The reaction mixture was stirred under blue LED irradiation (30 W, 440 nm, 75% intensity) with a fan for 16 h. After the reaction was complete, the reaction mixture was diluted in CH_2Cl_2 (15.0 mL) and concentrated under reduced pressure. The crude product was purified by flash chromatography (SiO_2, gradient of 0% → 10% Et_2O in pentane) to afford the desired compound **58** as a colorless oil.

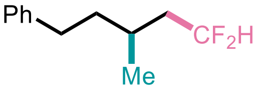

(5,5-difluoro-3-methylpentyl)benzene (58)

^{1}H NMR (500 MHz, $CDCl_3$): δ 7.29 (t, *J* = 7.6 Hz, 2H), 7.22–7.16 (m, 3H), 5.87 (tdd, *J* = 56.9, 5.4, 4.3 Hz, 1H), 2.64 (dddd, *J* = 40.0, 13.6, 10.3, 5.9 Hz, 2H), 1.97–1.86 (m, 1H), 1.82–1.64 (m, 3H), 1.58–1.51 (m, 1H), 1.05 (d, *J* = 6.5 Hz, 3H) ppm.

^{13}C NMR (126 MHz, $CDCl_3$): δ 142.31, 128.55, 128.51, 128.45, 125.98, 117.17 (t, *J* = 238.6 Hz), 40.98 (t, *J* = 19.9 Hz), 38.95, 33.24, 27.81, 27.77, 27.72, 19.74 ppm.

^{19}F NMR (470 MHz, $CDCl_3$): δ −114.33–−114.62 (m, 2F) ppm.

The NMR data are consistent with the reported data.[35]

MS (DART) m/z: $[M+H]^+$ Calcd. for $C_{12}H_{17}F_2^+$: 199.1298; Found: 199.1330.

18. Preparation and Characterization of Products.

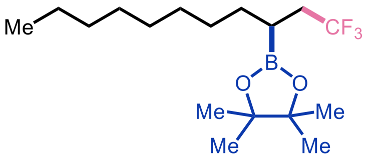

4,4,5,5-tetramethyl-2-(1,1,1-trifluoroundecan-3-yl)-1,3,2-dioxaborolane (6)

Following **General Procedure A**, B_2Cat_2 (190.2 mg, 0.80 mmol, 2.00 equiv.) and $TBAPF_6$ (232.5 mg, 0.60 mmol, 1.50 equiv.) were combined in the cathodic chamber of the H-cell, while the anodic chamber was charged with $TBAPF_6$ (232.5 mg, 0.60 mmol, 1.50 equiv.). Then, anhydrous degassed 1,4-dioxane (1.0 mL) and DMF (1.0 mL) were added to the cathodic chamber, while the anodic chamber was charged with DMF (2.0 mL). Subsequently, 1-decene (151 µL, 0.80 mmol, 2.00 equiv.) and CF_3I–Al–fum (188.0 mg, 0.40 mmol, 1.00 equiv.) were added to the cathodic chamber. Then, the reaction mixture was stirred at a constant cell potential of 3.2 V at room temperature for 7 h. After completion of the reaction, Et_3N (223 µL, 1.60 mmol, 4.00 equiv.) and pinacol (189.0 mg, 1.60 mmol, 4.00 equiv.) were added to the cathodic chamber, and the mixture was stirred for another 1 h. The crude product was purified by flash chromatography (SiO_2, gradient of 0% → 5% Et_2O in pentane) to afford compound **6** (92.8 mg, 69%) as a colorless oil.

^{1}H NMR (500 MHz, $CDCl_3$): δ 2.33–2.21 (m, 1H), 2.13–2.01 (m, 1H), 1.50–1.43 (m, 1H), 1.40–1.35 (m, 1H), 1.26–1.31 (m, 13H), 1.24 (s, 12H), 0.88 (t, J = 6.9 Hz, 3H) ppm.
^{13}C NMR (126 MHz, $CDCl_3$): δ 127.63 (q, J = 277.0 Hz), 83.61, 35.37 (q, J = 27.8 Hz), 32.00, 31.08, 29.57, 29.37, 28.72, 24.91, 24.86, 24.79, 22.83, 14.25 ppm.
^{19}F NMR (470 MHz, $CDCl_3$): δ −65.07 (t, J = 11.3 Hz) ppm.
^{11}B NMR (160 MHz, $CDCl_3$): δ 34.07 ppm.
MS (DART) m/z: $[M+H]^+$ Calcd. for $C_{17}H_{33}BF_3O_2^+$: 337.2520; Found: 337.2518.

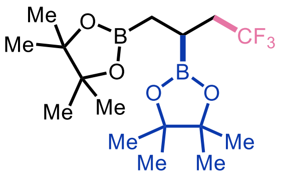

2,2'-(4,4,4-trifluorobutane-1,2-diyl)bis(4,4,5,5-tetramethyl-1,3,2-dioxaborolane) (7)

Following **General Procedure A**, B_2Cat_2 (190.2 mg, 0.80 mmol, 2.00 equiv.) and $TBAPF_6$ (232.5 mg, 0.60 mmol, 1.50 equiv.) were combined in the cathodic chamber of the H-cell, while the anodic chamber was charged with $TBAPF_6$ (232.5 mg, 0.60 mmol, 1.50 equiv.). Then, anhydrous degassed 1,4-dioxane (1.0 mL) and DMF (1.0 mL) were added to the cathodic chamber, while the anodic chamber was charged with DMF (2.0 mL). Subsequently, 2-allyl-4,4,5,5-tetramethyl-1,3,2-dioxaborolane (150 µL, 0.80 mmol, 2.00 equiv.) and CF_3I–Al–fum (188.0 mg, 0.40 mmol, 1.00 equiv.) were added to the cathodic chamber. Then, the reaction mixture was stirred at a constant cell potential of 3.2 V at room temperature for 7 h. After completion of the reaction, Et_3N (223 µL, 1.60 mmol, 4.00 equiv.) and pinacol (189.0 mg, 1.60 mmol, 4.00 equiv.) were added to the cathodic chamber, and the mixture was stirred for another 1 h. The crude product was purified by flash chromatography (SiO_2, gradient of 0% → 5% Et_2O in pentane) to afford compound **7** (83.0 mg, 57%) as a colorless oil.

^{1}H NMR (500 MHz, $CDCl_3$): δ 2.38–2.27 (m, 1H), 2.20–2.09 (m, 1H), 1.45 (ddd, J = 14.4, 7.8, 5.6 Hz, 1H), 1.23 (s, 24H), 1.02–0.88 (m, 2H) ppm.
^{13}C NMR (126 MHz, $CDCl_3$): δ 127.79 (q, J = 277.5 Hz), 83.60, 83.29, 36.64 (q, J = 27.4 Hz), 24.96, 24.95, 24.86, 24.82 ppm.
^{19}F NMR (470 MHz, $CDCl_3$): δ −64.50 (t, J = 11.4 Hz) ppm.
^{11}B NMR (160 MHz, $CDCl_3$): δ 34.25 ppm.
MS (DART) m/z: $[M+H]^+$ Calcd. for $C_{16}H_{30}B_2F_3O_2^+$: 365.2277; Found: 365.2281.

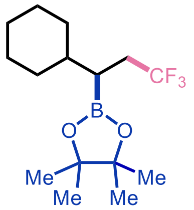

2-(1-cyclohexyl-3,3,3-trifluoropropyl)-4,4,5,5-tetramethyl-1,3,2-dioxaborolane (8)

Following **General Procedure A**, B_2Cat_2 (190.2 mg, 0.80 mmol, 2.00 equiv.) and $TBAPF_6$ (232.5 mg, 0.60 mmol, 1.50 equiv.) were combined in the cathodic chamber of the H-cell, while the anodic chamber was charged with $TBAPF_6$ (232.5 mg, 0.60 mmol, 1.50 equiv.). Then, anhydrous degassed 1,4-dioxane (1.0 mL) and DMF (1.0 mL) were added to the cathodic chamber, while the anodic chamber was charged with DMF (2.0 mL). Subsequently, vinylcyclohexane (109 μL, 0.80 mmol, 2.00 equiv.) and CF_3I–Al–fum (188.0 mg, 0.40 mmol, 1.00 equiv.) were added to the cathodic chamber. Then, the reaction mixture was stirred at a constant cell potential of 3.2 V at room temperature for 7 h. After completion of the reaction, Et_3N (223 μL, 1.60 mmol, 4.00 equiv.) and pinacol (189.0 mg, 1.60 mmol, 4.00 equiv.) were added to the cathodic chamber, and the mixture was stirred for another 1 h. The crude product was purified by flash chromatography (SiO_2, gradient of 0% → 5% Et_2O in pentane) to afford compound **8** (67.4 mg, 55%) as a colorless oil.

^{1}H NMR (500 MHz, $CDCl_3$): δ 2.36–2.25 (m, 1H), 2.13–2.03 (m, 1H), 1.75–1.70 (m, 3H), 1.67–1.62 (m, 2H), 1.45–1.37 (m, 1H), 1.24 (d, J = 2.7 Hz, 12H), 1.20–0.99 (m, 6H) ppm.
^{13}C NMR (126 MHz, $CDCl_3$): δ 127.96 (q, J = 276.7 Hz), 83.63, 39.81, 33.41 (q, J = 27.7 Hz), 32.59, 31.84, 26.75, 26.68, 26.61, 25.06, 24.87 ppm.
^{19}F NMR (470 MHz, $CDCl_3$): δ −65.29 (t, J = 11.3 Hz) ppm.
^{11}B NMR (160 MHz, $CDCl_3$): δ 33.74 ppm.
The NMR data are consistent with the reported data.[31]
MS (DART) m/z: $[M+H]^+$ Calcd. for $C_{15}H_{27}BF_3O_2^+$: 307.2051; Found: 307.2060.

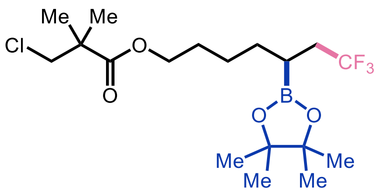

7,7,7-trifluoro-5-(4,4,5,5-tetramethyl-1,3,2-dioxaborolan-2-yl)heptyl 3-chloro-2,2-dimethylpropanoate (9)

Following **General Procedure A**, B_2Cat_2 (190.2 mg, 0.80 mmol, 2.00 equiv.) and $TBAPF_6$ (232.5 mg, 0.60 mmol, 1.50 equiv.) were combined in the cathodic chamber of the H-cell, while the anodic chamber was charged with $TBAPF_6$ (232.5 mg, 0.60 mmol, 1.50 equiv.). Then, anhydrous degassed 1,4-dioxane (1.0 mL) and DMF (1.0 mL) were added to the cathodic chamber, while the anodic chamber was charged with DMF (2.0 mL). Subsequently, hex-5-en-1-yl 3-chloro-2,2-dimethylpropanoate (174.4 mg, 0.80 mmol, 2.00 equiv.) and CF_3I–Al–fum (188.0 mg, 0.40 mmol, 1.00 equiv.) were added to the cathodic chamber. Then, the reaction mixture was stirred at a constant cell potential of 3.2 V at room temperature for 7 h. After completion of the reaction, Et_3N (223 µL, 1.60 mmol, 4.00 equiv.) and pinacol (189.0 mg, 1.60 mmol, 4.00 equiv.) were added to the cathodic chamber, and the mixture was stirred for another 1 h. The crude product was purified by flash chromatography (SiO_2, gradient of 0% → 5% Et_2O in pentane) to afford compound **9** (77.9 mg, 47%) as a colorless oil.

^{1}H NMR (500 MHz, $CDCl_3$): δ 4.09 (t, J = 6.6 Hz, 2H), 3.06 (s, 2H), 2.34–2.22 (m, 1H), 2.13–2.02 (m, 1H), 1.68–1.62 (m, 2H), 1.56–1.49 (m, 1H), 1.46–1.36 (m, 3H), 1.31–1.22 (m, 19H) ppm.
^{13}C NMR (126 MHz, $CDCl_3$): δ 175.21, 127.54 (q, J = 276.9 Hz), 83.72, 65.01, 52.21, 44.73, 35.17 (q, J = 27.9 Hz), 30.54, 28.78, 25.14, 24.90, 24.79, 23.37 ppm.
^{19}F NMR (470 MHz, $CDCl_3$): δ −64.97 (t, J = 11.2 Hz) ppm.
^{11}B NMR (160 MHz, $CDCl_3$): δ 32.93 ppm.
MS (DART) m/z: $[M+H]^+$ Calcd. for $C_{18}H_{32}BClF_3O_4^+$: 415.2029; Found: 415.2035.

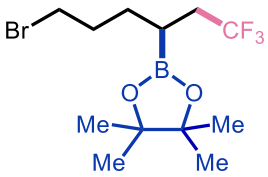

2-(6-bromo-1,1,1-trifluorohexan-3-yl)-4,4,5,5-tetramethyl-1,3,2-dioxaborolane (10)

Following **General Procedure A**, B_2Cat_2 (190.2 mg, 0.80 mmol, 2.00 equiv.) and $TBAPF_6$ (232.5 mg, 0.60 mmol, 1.50 equiv.) were combined in the cathodic chamber of the H-cell, while the anodic chamber was charged with $TBAPF_6$ (232.5 mg, 0.60 mmol, 1.50 equiv.). Then, anhydrous degassed 1,4-dioxane (1.0 mL) and DMF (1.0 mL) were added to the cathodic chamber, while the anodic chamber was charged with DMF (2.0 mL). Subsequently, 5-bromopent-1-ene (94.1 µL, 0.80 mmol, 2.00 equiv.) and CF_3I–Al–fum (188.0 mg, 0.40 mmol, 1.00 equiv.) were added to the cathodic chamber. Then, the reaction mixture was stirred at a constant cell potential of 3.2 V at room temperature for 7 h. After completion of the reaction, Et_3N (223 µL, 1.60 mmol, 4.00 equiv.) and pinacol (189.0 mg, 1.60 mmol, 4.00 equiv.) were added to the cathodic chamber, and the mixture was stirred for another 1 h. The crude product was purified by flash chromatography (SiO_2, gradient of 0% → 5% Et_2O in pentane) to afford compound **10** (92.2 mg, 67%) as a colorless oil.

^{1}H NMR (500 MHz, $CDCl_3$): δ 3.39 (td, J = 6.7, 1.4 Hz, 2H), 2.34–2.25 (m, 1H), 2.14–2.02 (m, 1H), 1.96–1.78 (m, 3H), 1.66–1.56 (m, 2H), 1.24 (s, 12H) ppm.
^{13}C NMR (126 MHz, $CDCl_3$): δ 127.44 (q, J = 277.0 Hz), 83.87, 35.25 (q, J = 27.9 Hz), 33.50, 31.87, 29.54, 24.92, 24.82 ppm.
^{19}F NMR (470 MHz, $CDCl_3$): δ −64.98 (t, J = 11.1 Hz) ppm.
^{11}B NMR (160 MHz, $CDCl_3$): δ 33.94 ppm.
MS (DART) m/z: $[M+H]^+$ Calcd. for $C_{12}H_{22}BBrF_3O_2^+$: 345.0843; Found: 345.0860.

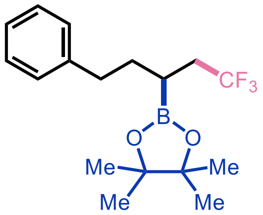

4,4,5,5-tetramethyl-2-(1,1,1-trifluoro-5-phenylpentan-3-yl)-1,3,2-dioxaborolane (11)

Following **General Procedure A**, B_2Cat_2 (190.2 mg, 0.80 mmol, 2.00 equiv.) and $TBAPF_6$ (232.5 mg, 0.60 mmol, 1.50 equiv.) were combined in the cathodic chamber of the H-cell, while the anodic chamber was charged with $TBAPF_6$ (232.5 mg, 0.60 mmol, 1.50 equiv.). Then, anhydrous degassed 1,4-dioxane (1.0 mL) and DMF (1.0 mL) were added to the cathodic chamber, while the anodic chamber was charged with DMF (2.0 mL). Subsequently, but-3-en-1-ylbenzene (116.5 µL, 0.80 mmol, 2.00 equiv.) and CF_3I–Al–fum (188.0 mg, 0.40 mmol, 1.00 equiv.) were added to the cathodic chamber. Then, the reaction mixture was stirred at a constant cell potential of 3.2 V at room temperature for 7 h. After completion of the reaction, Et_3N (223 µL, 1.60 mmol, 4.00 equiv.) and pinacol (189.0 mg, 1.60 mmol, 4.00 equiv.) were added to the cathodic chamber, and the mixture was stirred for another 1 h. The crude product was purified by flash chromatography (SiO_2, gradient of 0% → 10% Et_2O in pentane) to afford compound **11** (84.0 mg, 64%) as a colorless oil.

^{1}H NMR (500 MHz, $CDCl_3$): δ 7.31–7.28 (m, 2H), 7.21–7.18 (m, 3H), 2.70–2.59 (m, 2H), 2.41–2.29 (m, 1H), 2.21–2.10 (m, 1H), 1.87–1.80 (m, 1H), 1.77–1.69 (m, 1H), 1.41–1.35 (m, 1H), 1.28 (s, 12H) ppm.
^{13}C NMR (126 MHz, $CDCl_3$): δ 142.16, 128.53, 128.49, 127.46 (q, J = 277.0 Hz), 126.03, 83.79, 35.22 (q, J = 27.9 Hz), 35.13, 33.08, 24.93, 24.86 ppm.
^{19}F NMR (470 MHz, $CDCl_3$): δ −64.86 (t, J = 11.2 Hz) ppm.
^{11}B NMR (160 MHz, $CDCl_3$): δ 34.34 ppm.
MS (DART) m/z: $[M+H]^+$ Calcd. for $C_{17}H_{25}BF_3O_2^+$: 329.1894; Found: 329.1902.

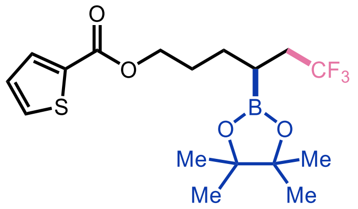

6,6,6-trifluoro-4-(4,4,5,5-tetramethyl-1,3,2-dioxaborolan-2-yl)hexyl thiophene-2-carboxylate (12)

Following **General Procedure A**, B_2Cat_2 (190.2 mg, 0.80 mmol, 2.00 equiv.) and $TBAPF_6$ (232.5 mg, 0.60 mmol, 1.50 equiv.) were combined in the cathodic chamber of the H-cell, while the anodic chamber was charged with $TBAPF_6$ (232.5 mg, 0.60 mmol, 1.50 equiv.). Then, anhydrous degassed 1,4-dioxane (1.0 mL) and DMF (1.0 mL) were added to the cathodic chamber, while the anodic chamber was charged with DMF (2.0 mL). Subsequently, pent-4-en-1-yl thiophene-2-carboxylate (156.8 mg, 0.80 mmol, 2.00 equiv.) and CF_3I–Al–fum (188.0 mg, 0.40 mmol, 1.00 equiv.) were added to the cathodic chamber. Then, the reaction mixture was stirred at a constant cell potential of 3.2 V at room temperature for 7 h. After completion of the reaction, Et_3N (223 µL, 1.60 mmol, 4.00 equiv.) and pinacol (189.0 mg, 1.60 mmol, 4.00 equiv.) were added to the cathodic chamber, and the mixture was stirred for another 1 h. The crude product was purified by flash chromatography (SiO_2, gradient of 0% → 15% Et_2O in pentane) to afford compound **12** (83.1 mg, 64%) as a colorless oil.

^{1}H NMR (500 MHz, $CDCl_3$): δ 7.79 (dd, J = 3.7, 1.3 Hz, 1H), 7.55 (dd, J = 4.9, 1.3 Hz, 1H), 7.10 (dd, J = 5.0, 3.8 Hz, 1H), 4.28 (t, J = 6.4 Hz, 2H), 2.38–2.27 (m,1H), 2.16–2.06 (m, 1H), 1.81–1.73 (m, 2H), 1.67–1.53 (m, 2H), 1.37–1.31 (m, 1H), 1.24 (s, 12H) ppm.
^{13}C NMR (126 MHz, $CDCl_3$): δ 162.38, 134.10, 133.46, 132.40, 127.85, 127.34 (q, J = 277.0 Hz), 83.83, 65.10, 35.19 (q, J = 28.0 Hz), 27.93, 27.34, 24.89, 24.80 ppm.
^{19}F NMR (470 MHz, $CDCl_3$): δ −64.92 (t, J = 11.2 Hz) ppm.
^{11}B NMR (160 MHz, $CDCl_3$): δ 32.25 ppm.
MS (DART) m/z: $[M+H]^+$ Calcd. for $C_{17}H_{25}BF_3O_4S^+$: 393.1513; Found: 393.1530.

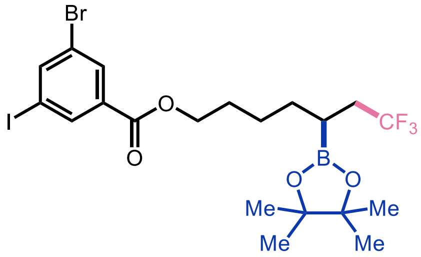

6,6,6-trifluoro-4-(4,4,5,5-tetramethyl-1,3,2-dioxaborolan-2-yl)hexyl 3-bromo-5-iodobenzoate (13)

Following **General Procedure A**, B_2Cat_2 (190.2 mg, 0.80 mmol, 2.00 equiv.) and $TBAPF_6$ (232.5 mg, 0.60 mmol, 1.50 equiv.) were combined in the cathodic chamber of the H-cell, while the anodic chamber was charged with $TBAPF_6$ (232.5 mg, 0.60 mmol, 1.50 equiv.). Then, anhydrous degassed 1,4-dioxane (1.0 mL) and DMF (1.0 mL) were added to the cathodic chamber, while the anodic chamber was charged with DMF (2.0 mL). Subsequently, hex-5-en-1-yl 3-bromo-5-iodobenzoate (326.3 mg, 0.80 mmol, 2.00 equiv.) and CF_3I–Al–fum (188.0 mg, 0.40 mmol, 1.00 equiv.) were added to the cathodic chamber. Then, the reaction mixture was stirred at a constant cell potential of 3.2 V at room temperature for 7 h. After completion of the reaction, Et_3N (223 μL, 1.60 mmol, 4.00 equiv.) and pinacol (189.0 mg, 1.60 mmol, 4.00 equiv.) were added to the cathodic chamber, and the mixture was stirred for another 1 h. The crude product was purified by flash chromatography (SiO_2, gradient of 0% → 10% Et_2O in pentane) to afford compound **13** (120.8 mg, 50%) as a white solid.

^{1}H NMR (500 MHz, $CDCl_3$): δ 8.27 (s, 1H), 8.10 (s, 1H), 8.03 (s, 1H), 4.31 (t, J = 6.5 Hz, 2H), 2.37–2.23 (m, 1H), 2.17–2.02 (m, 1H), 1.83–1.71 (m, 2H), 1.55–1.40 (m, 4H), 1.35–1.28 (m, 1H), 1.22 (s, 12H) ppm.
^{13}C NMR (126 MHz, $CDCl_3$): δ 164.01, 143.87, 137.23, 133.71, 132.03, 127.51 (q, J = 277.0 Hz), 123.13, 94.15, 83.76, 65.84, 35.31 (q, J = 28.0 Hz), 30.69, 28.84, 25.23, 24.92, 24.81 ppm.
^{19}F NMR (470 MHz, $CDCl_3$): δ −64.94 (t, J = 11.2 Hz) ppm.
^{11}B NMR (160 MHz, $CDCl_3$): δ 32.07 ppm.
MS (DART) m/z: $[M+H]^+$ Calcd. for $C_{20}H_{27}BBrF_3IO_4^+$: 605.0177; Found: 605.0152.
Melting Point: 272.8−273.6 °C.

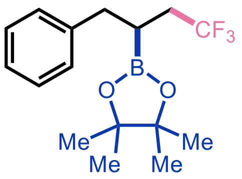

4,4,5,5-tetramethyl-2-(4,4,4-trifluoro-1-phenylbutan-2-yl)-1,3,2-dioxaborolane (14)

Following **General Procedure A**, B_2Cat_2 (190.2 mg, 0.80 mmol, 2.00 equiv.) and $TBAPF_6$ (232.5 mg, 0.60 mmol, 1.50 equiv.) were combined in the cathodic chamber of the H-cell, while the anodic chamber was charged with $TBAPF_6$ (232.5 mg, 0.60 mmol, 1.50 equiv.). Then, anhydrous degassed 1,4-dioxane (1.0 mL) and DMF (1.0 mL) were added to the cathodic chamber, while the anodic chamber was charged with DMF (2.0 mL). Subsequently, allylbenzene (106.2 µL, 0.80 mmol, 2.00 equiv.) and CF_3I–Al–fum (188.0 mg, 0.40 mmol, 1.00 equiv.) were added to the cathodic chamber. Then, the reaction mixture was stirred at a constant cell potential of 3.2 V at room temperature for 7 h. After completion of the reaction, Et_3N (223 µL, 1.60 mmol, 4.00 equiv.) and pinacol (189.0 mg, 1.60 mmol, 4.00 equiv.) were added to the cathodic chamber, and the mixture was stirred for another 1 h. The crude product was purified by flash chromatography (SiO_2, gradient of 0% → 5% Et_2O in pentane) to afford compound **14** (59.1 mg, 47%) as a colorless oil.

^{1}H NMR (500 MHz, $CDCl_3$): δ 7.29–7.26 (m, 2H), 7.20–7.17 (m, 3H), 2.80 (dd, J = 13.8, 8.0 Hz, 1H), 2.69 (dd, J = 13.8, 8.4 Hz, 1H), 2.31–2.19 (m, 1H), 2.15–2.04 (m, 1H), 1.71–1.65 (m, 1H), 1.16 (d, J = 22.1 Hz, 12H) ppm.
^{13}C NMR (126 MHz, $CDCl_3$): δ 140.29, 129.04, 128.50, 127.73 (q, J = 277.0 Hz), 126.42, 83.78, 36.47, 34.66 (q, J = 28.1 Hz), 24.87, 24.71 ppm.
^{19}F NMR (470 MHz, $CDCl_3$): δ −64.76 (t, J = 11.1 Hz) ppm.
^{11}B NMR (160 MHz, $CDCl_3$): δ 33.80 ppm.
The NMR data are consistent with the reported data.[31]
MS (DART) m/z: $[M+H]^+$ Calcd. for $C_{16}H_{23}BF_3O_2^+$: 315.1738; Found: 315.1729.

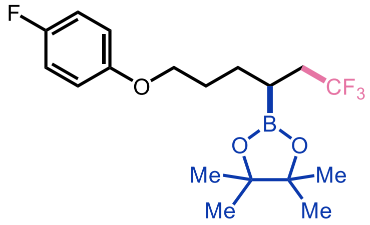

4,4,5,5-tetramethyl-2-(1,1,1-trifluoro-6-(4-fluorophenoxy)hexan-3-yl)-1,3,2-dioxaborolane (15)

Following **General Procedure A**, B_2Cat_2 (190.2 mg, 0.80 mmol, 2.00 equiv.) and $TBAPF_6$ (232.5 mg, 0.60 mmol, 1.50 equiv.) were combined in the cathodic chamber of the H-cell, while the anodic chamber was charged with $TBAPF_6$ (232.5 mg, 0.60 mmol, 1.50 equiv.). Then, anhydrous degassed 1,4-dioxane (1.0 mL) and DMF (1.0 mL) were added to the cathodic chamber, while the anodic chamber was charged with DMF (2.0 mL). Subsequently, 1-fluoro-4-(pent-4-en-1-yloxy)benzene (144.1 mg, 0.80 mmol, 2.00 equiv.) and CF_3I–Al–fum (188.0 mg, 0.40 mmol, 1.00 equiv.) were added to the cathodic chamber. Then, the reaction mixture was stirred at a constant cell potential of 3.2 V at room temperature for 7 h. After completion of the reaction, Et_3N (223 μL, 1.60 mmol, 4.00 equiv.) and pinacol (189.0 mg, 1.60 mmol, 4.00 equiv.) were added to the cathodic chamber, and the mixture was stirred for another 1 h. The crude product was purified by flash chromatography (SiO_2, gradient of 0% → 10% Et_2O in pentane) to afford compound **15** (100.8 mg, 67%) as a colorless oil.

^{1}H NMR (500 MHz, $CDCl_3$): δ 6.97–6.93 (m, 2H), 6.83–6.79 (m, 2H), 3.91 (t, J = 6.4 Hz, 2H), 2.38–2.27 (m, 1H), 2.17–2.07 (m, 1H), 1.84–1.75 (m, 2H), 1.69–1.56 (m, 2H), 1.37–1.31 (m, 1H), 1.24 (s, 12H) ppm.
^{13}C NMR (126 MHz, $CDCl_3$): δ 158.25, 156.36, 155.22, 155.21, 127.53 (q, J = 277.0 Hz), 115.95, 115.76, 115.60, 115.60, 115.60, 115.53, 83.80, 68.42, 35.25 (q, J = 28.0 Hz), 28.39, 27.41, 24.88, 24.80 ppm.
^{19}F NMR (470 MHz, $CDCl_3$): δ −64.93 (t, J = 11.2 Hz, 3F), −124.34 (tt, J = 7.9, 4.1 Hz, 1F) ppm.
^{11}B NMR (160 MHz, $CDCl_3$): δ 33.98 ppm.
MS (DART) m/z: $[M+H]^+$ Calcd. for $C_{18}H_{26}BF_4O_3^+$: 377.1906; Found: 377.1919.

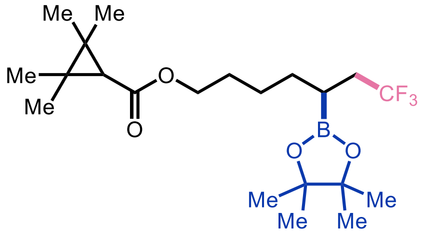

7,7,7-trifluoro-5-(4,4,5,5-tetramethyl-1,3,2-dioxaborolan-2-yl)heptyl 2,2,3,3-tetramethylcyclopropane-1-carboxylate (16)

Following **General Procedure A**, B_2Cat_2 (190.2 mg, 0.80 mmol, 2.00 equiv.) and $TBAPF_6$ (232.5 mg, 0.60 mmol, 1.50 equiv.) were combined in the cathodic chamber of the H-cell, while the anodic chamber was charged with $TBAPF_6$ (232.5 mg, 0.60 mmol, 1.50 equiv.). Then, anhydrous degassed 1,4-dioxane (1.0 mL) and DMF (1.0 mL) were added to the cathodic chamber, while the anodic chamber was charged with DMF (2.0 mL). Subsequently, hex-5-en-1-yl 2,2,3,3-tetramethylcyclopropane-1-carboxylate (144.1 mg, 0.80 mmol, 2.00 equiv.) and CF_3I–Al–fum (179.4 mg, 0.40 mmol, 1.00 equiv.) were added to the cathodic chamber. Then, the reaction mixture was stirred at a constant cell potential of 3.2 V at room temperature for 7 h. After completion of the reaction, Et_3N (223 µL, 1.60 mmol, 4.00 equiv.) and pinacol (189.0 mg, 1.60 mmol, 4.00 equiv.) were added to the cathodic chamber, and the mixture was stirred for another 1 h. The crude product was purified by flash chromatography (SiO_2, gradient of 0% → 10% Et_2O in pentane) to afford compound **16** (109.3 mg, 65%) as a colorless oil.

^{1}H NMR (500 MHz, $CDCl_3$): δ 3.99 (t, J = 6.6 Hz, 2H), 2.33–2.21 (m, 1H), 2.12–2.01 (m, 1H), 1.64–1.56 (m, 2H), 1.54–1.47 (m, 1H), 1.46–1.34 (m, 3H), 1.31–1.26 (m, 1H), 1.22 (s, 18H), 1.16 (s, 6H), 1.14 (s, 1H) ppm.
^{13}C NMR (126 MHz, $CDCl_3$): δ 172.37, 127.54 (q, J = 276.9 Hz), 83.67, 63.59, 35.89, 35.25 (q, J = 27.9 Hz), 30.67, 30.05, 28.94, 24.89, 24.77, 23.66, 16.69 ppm.
^{19}F NMR (470 MHz, $CDCl_3$): δ −65.05 (t, J = 11.2 Hz) ppm.
^{11}B NMR (160 MHz, $CDCl_3$): δ 34.56 ppm.
MS (DART) m/z: $[M+H]^+$ Calcd. for $C_{21}H_{37}BF_3O_4^+$: 421.2732; Found: 421.2750.

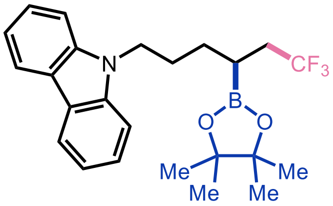

9-(6,6,6-trifluoro-4-(4,4,5,5-tetramethyl-1,3,2-dioxaborolan-2-yl)hexyl)-9H-carbazole (17)

Following **General Procedure A**, B_2Cat_2 (190.2 mg, 0.80 mmol, 2.00 equiv.) and $TBAPF_6$ (232.5 mg, 0.60 mmol, 1.50 equiv.) were combined in the cathodic chamber of the H-cell, while the anodic chamber was charged with $TBAPF_6$ (232.5 mg, 0.60 mmol, 1.50 equiv.). Then, anhydrous degassed 1,4-dioxane (1.0 mL) and DMF (1.0 mL) were added to the cathodic chamber, while the anodic chamber was charged with DMF (2.0 mL). Subsequently, 9-(pent-4-en-1-yl)-9H-carbazole (188.1 mg, 0.80 mmol, 2.00 equiv.) and CF_3I–Al–fum (179.4 mg, 0.40 mmol, 1.00 equiv.) were added to the cathodic chamber. Then, the reaction mixture was stirred at a constant cell potential of 3.2 V at room temperature for 7 h. After completion of the reaction, Et_3N (223 μL, 1.60 mmol, 4.00 equiv.) and pinacol (189.0 mg, 1.60 mmol, 4.00 equiv.) were added to the cathodic chamber, and the mixture was stirred for another 1 h. The crude product was purified by flash chromatography (SiO_2, gradient of 10% → 40% Et_2O in pentane) to afford compound **17** (77.6 mg, 45%) as a white solid.

^{1}H NMR (500 MHz, $CDCl_3$): δ 8.10 (d, J = 7.8 Hz, 2H), 7.47–7.39 (m, 4H), 7.22 (ddd, J = 7.9, 6.9, 1.2 Hz, 2H), 4.38–4.25 (m, 2H), 2.34–1.83 (m, 4H), 1.66–1.49 (m, 2H), 1.38–1.32 (m, 1H), 1.16 (d, J = 2.9 Hz, 12H) ppm.
^{13}C NMR (126 MHz, $CDCl_3$): δ 140.47, 125.73, 122.99, 121.65 (q, J = 277.0 Hz), 120.49, 118.93, 108.73, 83.83, 35.31 (q, J = 28.2 Hz), 29.85, 28.62, 28.05, 24.79, 1.17 ppm.
^{19}F NMR (470 MHz, $CDCl_3$): δ −64.98 (t, J = 11.2 Hz) ppm.
^{11}B NMR (160 MHz, $CDCl_3$): δ 33.88 ppm.
The NMR data are consistent with the reported data.[31]
MS (DART) m/z: $[M+H]^+$ Calcd. for $C_{24}H_{30}BF_3NO_2^+$: 432.2316; Found: 432.2332.

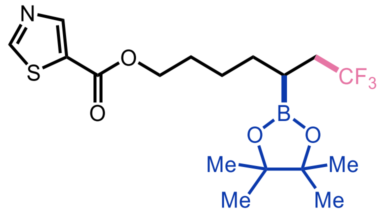

6,6,6-trifluoro-4-(4,4,5,5-tetramethyl-1,3,2-dioxaborolan-2-yl)hexyl thiazole-5-carboxylate (18)

Following **General Procedure A**, B_2Cat_2 (190.2 mg, 0.80 mmol, 2.00 equiv.) and $TBAPF_6$ (232.5 mg, 0.60 mmol, 1.50 equiv.) were combined in the cathodic chamber of the H-cell, while the anodic chamber was charged with $TBAPF_6$ (232.5 mg, 0.60 mmol, 1.50 equiv.). Then, anhydrous degassed 1,4-dioxane (1.0 mL) and DMF (1.0 mL) were added to the cathodic chamber, while the anodic chamber was charged with DMF (2.0 mL). Subsequently, hex-5-en-1-yl thiazole-5-carboxylate (168.9 mg, 0.80 mmol, 2.00 equiv.) and CF_3I–Al–fum (179.4 mg, 0.40 mmol, 1.00 equiv.) were added to the cathodic chamber. Then, the reaction mixture was stirred at a constant cell potential of 3.2 V at room temperature for 7 h. After completion of the reaction, Et_3N (223 μL, 1.60 mmol, 4.00 equiv.) and pinacol (189.0 mg, 1.60 mmol, 4.00 equiv.) were added to the cathodic chamber, and the mixture was stirred for another 1 h. The crude product was purified by flash chromatography (SiO_2, gradient of 0% → 30% Et_2O in pentane) to afford compound **18** (99.3 mg, 61%) as a colorless oil.

^{1}H NMR (500 MHz, $CDCl_3$): δ 8.84 (d, J = 2.1 Hz, 1H), 8.22 (d, J = 2.2 Hz, 1H), 4.35 (t, J = 6.7 Hz, 2H), 2.33–2.21 (m, 1H), 2.12–2.02 (m, 1H), 1.84–1.72 (m, 2H), 1.58–1.50 (m, 1H), 1.47–1.41 (m, 3H), 1.33–1.26 (m, 1H), 1.20 (s, 12H) ppm.
^{13}C NMR (126 MHz, $CDCl_3$): δ 161.42, 153.50, 148.27, 127.51 (q, J = 277.0 Hz), 127.25, 83.69, 65.46, 35.21 (q, J = 27.9 Hz), 30.59, 28.82, 25.13, 24.85, 24.74, 1.13 ppm.
^{19}F NMR (470 MHz, $CDCl_3$): δ −64.98 (t, J = 11.2 Hz) ppm.
^{11}B NMR (160 MHz, $CDCl_3$): δ 31.25 ppm.
MS (DART) m/z: $[M+NH_4]^+$ Calcd. for $C_{17}H_{26}BF_3NO_4S^+$: 408.1628; Found: 408.1611.

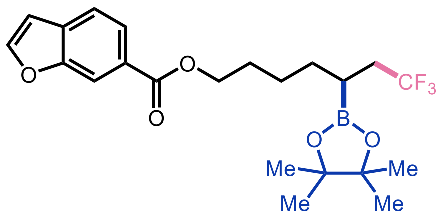

7,7,7-trifluoro-5-(4,4,5,5-tetramethyl-1,3,2-dioxaborolan-2-yl)heptyl benzofuran-6-carboxylate (19)

Following **General Procedure A**, B_2Cat_2 (190.2 mg, 0.80 mmol, 2.00 equiv.) and $TBAPF_6$ (232.5 mg, 0.60 mmol, 1.50 equiv.) were combined in the cathodic chamber of the H-cell, while the anodic chamber was charged with $TBAPF_6$ (232.5 mg, 0.60 mmol, 1.50 equiv.). Then, anhydrous degassed 1,4-dioxane (1.0 mL) and DMF (1.0 mL) were added to the cathodic chamber, while the anodic chamber was charged with DMF (2.0 mL). Subsequently, hex-5-en-1-yl benzofuran-6-carboxylate (195.3 mg, 0.80 mmol, 2.00 equiv.) and CF_3I–Al–fum (179.4 mg, 0.40 mmol, 1.00 equiv.) were added to the cathodic chamber. Then, the reaction mixture was stirred at a constant cell potential of 3.2 V at room temperature for 7 h. After completion of the reaction, Et_3N (223 µL, 1.60 mmol, 4.00 equiv.) and pinacol (189.0 mg, 1.60 mmol, 4.00 equiv.) were added to the cathodic chamber, and the mixture was stirred for another 1 h. The crude product was purified by flash chromatography (SiO_2, gradient of 0% → 15% Et_2O in pentane) to afford compound **19** (77.5 mg, 44%) as a colorless oil.

^{1}H NMR (500 MHz, $CDCl_3$): δ 8.36–8.26 (m, 1H), 8.01 (dd, J = 8.6, 1.7 Hz, 1H), 7.67 (d, J = 2.2 Hz, 1H), 7.51 (dt, J = 8.7, 0.80 Hz, 1H), 6.82 (dd, J = 2.2, 1.0 Hz, 1H), 4.32 (t, J = 6.5 Hz, 2H), 2.35–2.23 (m, 1H), 2.14–2.03 (m, 1H), 1.84–1.74 (m, 2H), 1.61–1.44 (m, 4H), 1.32 (dt, J = 8.9, 4.5 Hz, 1H), 1.22 (d, J = 23.2 Hz, 12H) ppm.
^{13}C NMR (126 MHz, $CDCl_3$): δ 166.87, 157.56, 146.33, 127.50, 127.24 (q, J = 277.0 Hz), 126.11, 125.54, 123.76, 111.35, 111.31, 107.23, 107.20, 83.67, 83.60, 64.89, 35.28 (q, J = 28.0 Hz), 30.71, 28.97, 25.32, 25.13, 24.84, 24.74 ppm.
^{19}F NMR (470 MHz, $CDCl_3$): δ −64.97 (t, J = 11.2 Hz) ppm.
^{11}B NMR (160 MHz, $CDCl_3$): δ 33.97 ppm.
MS (DART) m/z: $[M+H]^+$ Calcd. for $C_{22}H_{29}BF_3O_5^+$: 441.2055; Found: 441.2061.

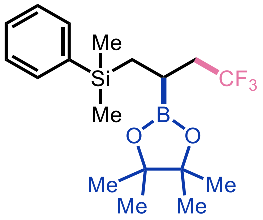

dimethyl(phenyl)(4,4,4-trifluoro-2-(4,4,5,5-tetramethyl-1,3,2-dioxaborolan-2-yl)butyl)silane (20)

Following **General Procedure A**, B_2Cat_2 (190.2 mg, 0.80 mmol, 2.00 equiv.) and $TBAPF_6$ (232.5 mg, 0.60 mmol, 1.50 equiv.) were combined in the cathodic chamber of the H-cell, while the anodic chamber was charged with $TBAPF_6$ (232.5 mg, 0.60 mmol, 1.50 equiv.). Then, anhydrous degassed 1,4-dioxane (1.0 mL) and DMF (1.0 mL) were added to the cathodic chamber, while the anodic chamber was charged with DMF (2.0 mL). Subsequently, allyldimethyl(phenyl)silane (140.9 mg, 0.80 mmol, 2.00 equiv.) and CF_3I–Al–fum (179.4 mg, 0.40 mmol, 1.00 equiv.) were added to the cathodic chamber. Then, the reaction mixture was stirred at a constant cell potential of 3.2 V at room temperature for 7 h. After completion of the reaction, Et_3N (223 µL, 1.60 mmol, 4.00 equiv.) and pinacol (189.0 mg, 1.60 mmol, 4.00 equiv.) were added to the cathodic chamber, and the mixture was stirred for another 1 h. The crude product was purified by flash chromatography (SiO_2, gradient of 0% → 10% Et_2O in pentane) to afford compound **20** (83.4 mg, 56%) as a colorless oil.

^{1}H NMR (500 MHz, $CDCl_3$): δ 7.52–7.50 (m, 2H), 7.36–7.34 (m, 3H), 2.33–2.20 (m, 1H), 2.07–1.97 (m, 1H), 1.41–1.35 (m, 1H), 1.20 (s, 12H), 1.02 (dd, J = 14.9, 7.5 Hz, 1H), 0.80 (dd, J = 14.9, 7.0 Hz, 1H), 0.32 (d, J = 4.5 Hz, 6H) ppm.
^{13}C NMR (126 MHz, $CDCl_3$): δ 139.23, 133.72, 129.13, 128.13, 127.96, 127.17 (q, J = 277.0 Hz), 83.61, 37.93 (q, J = 27.2 Hz), 24.85, 24.83, 16.45, −2.25, −2.52 ppm.
^{19}F NMR (470 MHz, $CDCl_3$): δ −64.88 (t, J = 11.1 Hz) ppm.
^{11}B NMR (160 MHz, $CDCl_3$): δ 33.92 ppm.
MS (DART) m/z: $[M+H]^+$ Calcd. for $C_{18}H_{29}BF_3O_2Si^+$: 373.1976; Found: 373.1988.

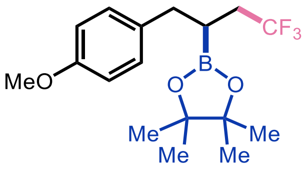

4,4,5,5-tetramethyl-2-(4,4,4-trifluoro-1-(4-methoxyphenyl)butan-2-yl)-1,3,2-dioxaborolane (21)

Following **General Procedure A**, B_2Cat_2 (190.2 mg, 0.80 mmol, 2.00 equiv.) and $TBAPF_6$ (232.5 mg, 0.60 mmol, 1.50 equiv.) were combined in the cathodic chamber of the H-cell, while the anodic chamber was charged with $TBAPF_6$ (232.5 mg, 0.60 mmol, 1.50 equiv.). Then, anhydrous degassed 1,4-dioxane (1.0 mL) and DMF (1.0 mL) were added to the cathodic chamber, while the anodic chamber was charged with DMF (2.0 mL). Subsequently, 1-allyl-4-methoxybenzene (125 µL, 0.80 mmol, 2.00 equiv.) and CF_3I–Al–fum (179.4 mg, 0.40 mmol, 1.00 equiv.) were added to the cathodic chamber. Then, the reaction mixture was stirred at a constant cell potential of 3.2 V at room temperature for 7 h. After completion of the reaction, Et_3N (223 µL, 1.60 mmol, 4.00 equiv.) and pinacol (189.0 mg, 1.60 mmol, 4.00 equiv.) were added to the cathodic chamber, and the mixture was stirred for another 1 h. The crude product was purified by flash chromatography (SiO_2, gradient of 0% → 10% Et_2O in pentane) to afford compound **21** (70.2 mg, 51%) as a colorless oil.

^{1}H NMR (500 MHz, $CDCl_3$): δ 7.11 (d, J = 8.6 Hz, 2H), 6.82 (d, J = 8.6 Hz, 2H), 3.78 (s, 3H), 2.75 (dd, J = 13.9, 7.9 Hz, 1H), 2.62 (dd, J = 13.9, 8.5 Hz, 1H), 2.29–2.17 (m, 1H), 2.14–2.03 (m, 1H), 1.66–1.60 (m, 1H), 1.17 (d, J = 19.8 Hz, 12H) ppm.
^{13}C NMR (126 MHz, $CDCl_3$): δ 158.24, 132.34, 130.84, 127.52 (q, J = 277.0 Hz), 113.89, 83.74, 55.40, 35.57, 34.57 (q, J = 28.0 Hz), 24.88, 24.73 ppm.
^{19}F NMR (470 MHz, $CDCl_3$): δ −64.75 (t, J = 11.1 Hz) ppm.
^{11}B NMR (160 MHz, $CDCl_3$): δ 31.86 ppm.
The NMR data are consistent with the reported data.[31]
MS (DART) m/z: $[M+H]^+$ Calcd. for $C_{17}H_{25}BF_3O_3^+$: 345.1843; Found: 345.1856.

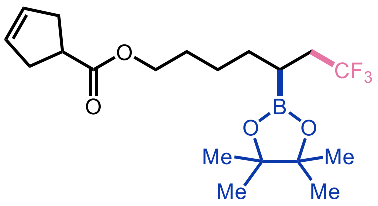

7,7,7-trifluoro-5-(4,4,5,5-tetramethyl-1,3,2-dioxaborolan-2-yl)heptyl cyclopent-3-ene-1-carboxylate (22)

Following **General Procedure A**, B_2Cat_2 (190.2 mg, 0.80 mmol, 2.00 equiv.) and $TBAPF_6$ (232.5 mg, 0.60 mmol, 1.50 equiv.) were combined in the cathodic chamber of the H-cell, while the anodic chamber was charged with $TBAPF_6$ (232.5 mg, 0.60 mmol, 1.50 equiv.). Then, anhydrous degassed 1,4-dioxane (1.0 mL) and DMF (1.0 mL) were added to the cathodic chamber, while the anodic chamber was charged with DMF (2.0 mL). Subsequently, hex-5-en-1-yl cyclopent-3-ene-1-carboxylate (155.3 mg, 0.80 mmol, 2.00 equiv.) and CF_3I–Al–fum (179.4 mg, 0.40 mmol, 1.00 equiv.) were added to the cathodic chamber. Then, the reaction mixture was stirred at a constant cell potential of 3.2 V at room temperature for 7 h. After completion of the reaction, Et_3N (223 μL, 1.60 mmol, 4.00 equiv.) and pinacol (189.0 mg, 1.60 mmol, 4.00 equiv.) were added to the cathodic chamber, and the mixture was stirred for another 1 h. The crude product was purified by flash chromatography (SiO_2, gradient of 0% → 5% Et_2O in pentane) to afford compound **22** (101.5 mg, 65%) as a colorless oil.

^{1}H NMR (500 MHz, $CDCl_3$): δ 5.65 (s, 2H), 4.07 (t, J = 6.6 Hz, 2H), 3.12–3.06 (m, 1H), 2.65–2.61 (m, 4H), 2.34–2.22 (m, 1H), 2.12–2.02 (m, 1H), 1.68–1.59 (m, 2H), 1.56–1.48 (m, 1H), 1.47–1.33 (m, 3H), 1.32–1.26 (m, 1H), 1.23 (s, 12H) ppm.
^{13}C NMR (126 MHz, $CDCl_3$): δ 176.38, 129.11, 127.67 (q, J = 277.0Hz), 83.71, 64.53, 41.73, 36.45, 35.23 (q, J = 27.8 Hz), 30.63, 28.85, 25.18, 24.89, 24.78 ppm.
^{19}F NMR (470 MHz, $CDCl_3$): δ −65.01 (t, J = 11.2 Hz) ppm.
^{11}B NMR (160 MHz, $CDCl_3$): δ 32.01 ppm.
MS (DART) m/z: $[M+H]^+$ Calcd. for $C_{19}H_{31}BF_3O_4^+$: 391.2262; Found: 391.2251.

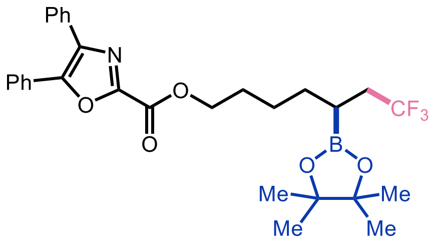

7,7,7-trifluoro-5-(4,4,5,5-tetramethyl-1,3,2-dioxaborolan-2-yl)heptyl 4,5-diphenyloxazole-2-carboxylate (23)

Following **General Procedure A**, B_2Cat_2 (190.2 mg, 0.80 mmol, 2.00 equiv.) and $TBAPF_6$ (232.5 mg, 0.60 mmol, 1.50 equiv.) were combined in the cathodic chamber of the H-cell, while the anodic chamber was charged with $TBAPF_6$ (232.5 mg, 0.60 mmol, 1.50 equiv.). Then, anhydrous degassed 1,4-dioxane (1.0 mL) and DMF (1.0 mL) were added to the cathodic chamber, while the anodic chamber was charged with DMF (2.0 mL). Subsequently, hex-5-en-1-yl 4,5-diphenyloxazole-2-carboxylate (277.7 mg, 0.80 mmol, 2.00 equiv.) and CF_3I–Al–fum (179.4 mg, 0.40 mmol, 1.00 equiv.) were added to the cathodic chamber. Then, the reaction mixture was stirred at a constant cell potential of 3.2 V at room temperature for 7 h. After completion of the reaction, Et_3N (223 µL, 1.60 mmol, 4.00 equiv.) and pinacol (189.0 mg, 1.60 mmol, 4.00 equiv.) were added to the cathodic chamber, and the mixture was stirred for another 1 h. The crude product was purified by flash chromatography (SiO_2, gradient of 0% → 40% Et_2O in pentane) to afford compound **23** (91.3 mg, 42%) as a colorless oil.

^{1}H NMR (500 MHz, $CDCl_3$): δ 7.63 (d, J = 8.4 Hz, 2H), 7.57 (d, J = 7.4 Hz, 2H), 7.38–7.29 (m, 6H), 4.11 (t, J = 8.0 Hz, 2H), 3.18 (t, J = 7.5 Hz, 2H), 2.90 (t, J = 8.0 Hz, 2H), 1.70–1.62 (m, 2H), 1.50–1.36 (m, 3H), 1.23 (s, 12H) ppm.
^{13}C NMR (126 MHz, $CDCl_3$): δ 172.18, 172.15, 161.88, 145.54, 135.28, 132.61, 129.14, 128.78, 128.68, 128.59, 128.19, 128.03, 126.61, 83.71, 64.83, 64.44, 35.22 (q, J = 28.0 Hz), 31.30, 30.56, 28.75, 27.71, 26.17, 25.17, 25.10, 24.90, 24.79, 23.70, 21.21 ppm.
^{19}F NMR (470 MHz, $CDCl_3$): δ −65.76 (t, J = 11.1 Hz) ppm.
^{11}B NMR (160 MHz, $CDCl_3$): δ 32.65 ppm.
MS (DART) m/z: $[M+NH_4]^+$ Calcd. for $C_{29}H_{34}BF_3N_2O_5^+$: 544.2482; Found: 544.2466.

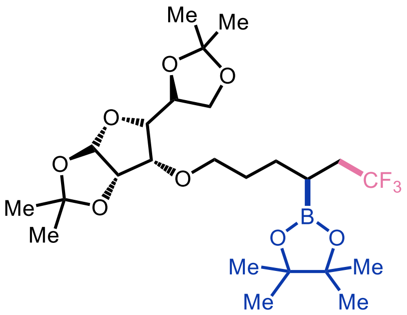

2-((S)-6-(((3aS,5R,6S,6aS)-5-((R)-2,2-dimethyl-1,3-dioxolan-4-yl)-2,2-dimethyltetrahydrofuro[2,3-d][1,3]dioxol-6-yl)oxy)-1,1,1-trifluorohexan-3-yl)-4,4,5,5-tetramethyl-1,3,2-dioxaborolane (24)

Following **General Procedure A**, B_2Cat_2 (190.2 mg, 0.80 mmol, 2.00 equiv.) and $TBAPF_6$ (232.5 mg, 0.60 mmol, 1.50 equiv.) were combined in the cathodic chamber of the H-cell, while the anodic chamber was charged with $TBAPF_6$ (232.5 mg, 0.60 mmol, 1.50 equiv.). Then, anhydrous degassed 1,4-dioxane (1.0 mL) and DMF (1.0 mL) were added to the cathodic chamber, while the anodic chamber was charged with DMF (2.0 mL). Subsequently, (3aS,5R,6S,6aS)-5-((R)-2,2-dimethyl-1,3-dioxolan-4-yl)-2,2-dimethyl-6-(pent-4-en-1-yloxy)tetrahydrofuro[2,3-d][1,3]dioxole (262.6 mg, 0.80 mmol, 2.00 equiv.) and CF_3I–Al–fum (179.4 mg, 0.40 mmol, 1.00 equiv.) were added to the cathodic chamber. Then, the reaction mixture was stirred at a constant cell potential of 3.2 V at room temperature for 7 h. After completion of the reaction, Et_3N (223 µL, 1.60 mmol, 4.00 equiv.) and pinacol (189.0 mg, 1.60 mmol, 4.00 equiv.) were added to the cathodic chamber, and the mixture was stirred for another 1 h. The crude product was purified by flash chromatography (SiO_2, gradient of 0% → 20% Et_2O in pentane) to afford compound **24** (167.8 mg, 80%) as a white solid.

^{1}H NMR (500 MHz, $CDCl_3$): δ 4.94 (d, J = 1.4 Hz, 1H), 4.76 (dd, J = 5.9, 3.6 Hz, 1H), 4.56 (d, J = 5.9 Hz, 1H), 4.38 (ddd, J = 7.7, 6.3, 4.4 Hz, 1H), 4.10 (dd, J = 8.6, 6.3 Hz, 1H), 4.01 (ddd, J = 8.7, 4.4, 0.9 Hz, 1H), 3.89 (dd, J = 7.8, 3.6 Hz, 1H), 3.60 (dtd, J = 9.8, 6.2, 3.3 Hz, 1H), 3.35 (dtd, J = 9.8, 6.3, 1.6 Hz, 1H), 2.34–2.22 (m, 1H), 2.13–2.02 (m, 1H), 1.59–1.54 (m, 2H), 1.51–1.41 (m, 8H), 1.37 (s, 3H), 1.31 (s, 3H), 1.25 (s, 1H), 1.23 (s, 12H) ppm.
^{13}C NMR (126 MHz, $CDCl_3$): δ 127.52 (q, J = 277.1 Hz), 112.68, 109.35, 106.36, 85.20, 83.72, 80.41, 79.68, 73.29, 67.30, 67.12, 35.24 (qd, J = 27.9, 11.0 Hz), 28.52 (d, J = 9.4 Hz), 27.52 (d, J = 8.7 Hz), 26.03, 25.15, 24.89, 24.79, 24.67 ppm.
^{19}F NMR (470 MHz, $CDCl_3$): δ −64.97 (td, J = 11.1, 2.9 Hz) ppm.
^{11}B NMR (160 MHz, $CDCl_3$): δ 32.37 ppm.
MS (DART) m/z: $[M+H]^+$ Calcd. for $C_{24}H_{41}BF_3O_8^+$: 525.2841; Found: 525.2822.
Melting Point: 218.8−220.1 °C.

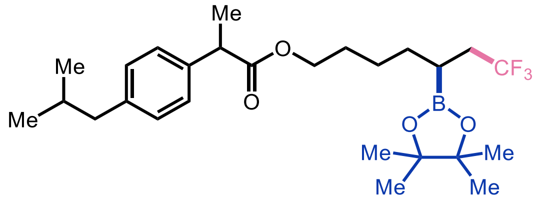

7,7,7-trifluoro-5-(4,4,5,5-tetramethyl-1,3,2-dioxaborolan-2-yl)heptyl 2-(4-isobutylphenyl)propanoate (25)

Following **General Procedure A**, B_2Cat_2 (190.2 mg, 0.80 mmol, 2.00 equiv.) and $TBAPF_6$ (232.5 mg, 0.60 mmol, 1.50 equiv.) were combined in the cathodic chamber of the H-cell, while the anodic chamber was charged with $TBAPF_6$ (232.5 mg, 0.60 mmol, 1.50 equiv.). Then, anhydrous degassed 1,4-dioxane (1.0 mL) and DMF (1.0 mL) were added to the cathodic chamber, while the anodic chamber was charged with DMF (2.0 mL). Subsequently, hex-5-en-1-yl 2-(4-isobutylphenyl)propanoate (230.6 mg, 0.80 mmol, 2.00 equiv.) and CF_3I–Al–fum (179.4 mg, 0.40 mmol, 1.00 equiv.) were added to the cathodic chamber. Then, the reaction mixture was stirred at a constant cell potential of 3.2 V at room temperature for 7 h. After completion of the reaction, Et_3N (223 µL, 1.60 mmol, 4.00 equiv.) and pinacol (189.0 mg, 1.60 mmol, 4.00 equiv.) were added to the cathodic chamber, and the mixture was stirred for another 1 h. The crude product was purified by flash chromatography (SiO_2, gradient of 0% → 5% Et_2O in pentane) to afford compound **25** (139.5 mg, 72%) as a white solid.

^{1}H NMR (500 MHz, $CDCl_3$): δ 7.19 (d, J = 8.2 Hz, 2H), 7.08 (d, J = 7.8 Hz, 2H), 4.08–4.00 (m, 2H), 3.67 (q, J = 7.1 Hz, 1H), 2.44 (d, J = 7.2 Hz, 2H), 2.30–2.19 (m, 1H), 2.04 (dddd, J = 22.2, 11.1, 4.9, 2.5 Hz, 1H), 1.84 (dp, J = 13.5, 6.7 Hz, 1H), 1.61–1.55 (m, 2H), 1.48 (d, J = 7.2 Hz, 3H), 1.41–1.26 (m, 5H), 1.23 (s, 12H), 0.89 (d, J = 6.6 Hz, 6H) ppm.
^{13}C NMR (126 MHz, $CDCl_3$): δ 174.88, 174.87, 140.59, 137.94, 129.40, 127.82 (q, J = 277.1 Hz), 127.23, 83.65, 64.61, 64.58, 45.28, 45.13, 35.12 (q, J = 27.8 Hz), 30.48, 30.46, 30.28, 28.69, 28.67, 25.00, 24.98, 24.86, 24.74, 22.49, 18.63, 18.62 ppm.
^{19}F NMR (470 MHz, $CDCl_3$): δ −64.99 (t, J = 11.2 Hz) ppm.
^{11}B NMR (160 MHz, $CDCl_3$): δ 30.77 ppm.
The NMR data are consistent with the reported data.[31]
MS (DART) m/z: $[M+H]^+$ Calcd. for $C_{26}H_{41}BF_3O_4^+$: 485.3045; Found: 485.3063.

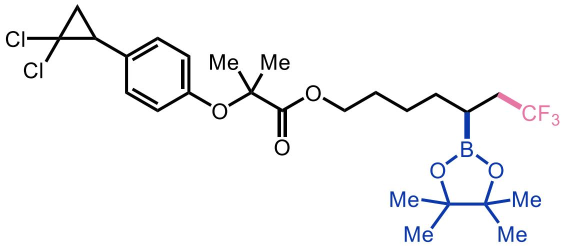

7,7,7-trifluoro-5-(4,4,5,5-tetramethyl-1,3,2-dioxaborolan-2-yl)heptyl 2-(4-(2,2-dichlorocyclopropyl)phenoxy)-2-methylpropanoate (26)

Following **General Procedure A**, B_2Cat_2 (190.2 mg, 0.80 mmol, 2.00 equiv.) and $TBAPF_6$ (232.5 mg, 0.60 mmol, 1.50 equiv.) were combined in the cathodic chamber of the H-cell, while the anodic chamber was charged with $TBAPF_6$ (232.5 mg, 0.60 mmol, 1.50 equiv.). Then, anhydrous degassed 1,4-dioxane (1.0 mL) and DMF (1.0 mL) were added to the cathodic chamber, while the anodic chamber was charged with DMF (2.0 mL). Subsequently, hex-5-en-1-yl 2-(4-(2,2-dichlorocyclopropyl)phenoxy)-2-methylpropanoate (296.1 mg, 0.80 mmol, 2.00 equiv.) and CF_3I–Al–fum (179.4 mg, 0.40 mmol, 1.00 equiv.) were added to the cathodic chamber. Then, the reaction mixture was stirred at a constant cell potential of 3.2 V at room temperature for 7 h. After completion of the reaction, Et_3N (223 µL, 1.60 mmol, 4.00 equiv.) and pinacol (189.0 mg, 1.60 mmol, 4.00 equiv.) were added to the cathodic chamber, and the mixture was stirred for another 1 h. The crude product was purified by flash chromatography (SiO_2, gradient of 0% → 10% Et_2O in pentane) to afford compound **26** (149.5 mg, 66%) as a colorless oil.

^{1}H NMR (500 MHz, $CDCl_3$): δ 7.10 (d, J = 8.6 Hz, 2H), 6.79 (d, J = 8.7 Hz, 2H), 4.13 (t, J = 6.6 Hz, 2H), 2.82 (ddd, J = 10.6, 8.3, 4.1 Hz, 1H), 2.29–2.17 (m, 1H), 2.06–1.96 (m, 1H), 1.92 (dd, J = 10.7, 7.4 Hz, 1H), 1.77 (ddd, J = 8.5, 7.4, 1.3 Hz, 1H), 1.62–1.56 (m, 8H), 1.50–1.42 (m, 1H), 1.40–1.34 (m, 1H), 1.32–1.25 (m, 2H), 1.22 (s, 13H) ppm.
^{13}C NMR (126 MHz, $CDCl_3$): δ 174.37, 155.10, 129.75, 128.22, 127.35 (q, J = 277.0 Hz), 118.67, 118.66, 83.70, 79.26, 77.37, 65.41, 61.00, 35.06 (qd, J = 27.9, 11.0 Hz), 34.93, 30.43, 30.42, 28.61, 25.92, 25.58, 25.56, 25.52, 25.50, 24.99, 24.98, 24.88, 24.77 ppm.
^{19}F NMR (470 MHz, $CDCl_3$): δ −64.94 (t, J = 11.2 Hz) ppm.
^{11}B NMR (160 MHz, $CDCl_3$): δ 31.84 ppm.
MS (DART) m/z: $[M+H]^+$ Calcd. for $C_{26}H_{37}BCl_2F_3O_5^+$: 567.2058; Found: 567.2046.

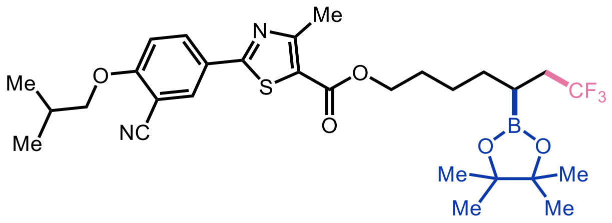

7,7,7-trifluoro-5-(4,4,5,5-tetramethyl-1,3,2-dioxaborolan-2-yl)heptyl 2-(3-cyano-4-isobutoxyphenyl)-4-methylthiazole-5-carboxylate (27)

Following **General Procedure A**, B_2Cat_2 (190.2 mg, 0.80 mmol, 2.00 equiv.) and $TBAPF_6$ (232.5 mg, 0.60 mmol, 1.50 equiv.) were combined in the cathodic chamber of the H-cell, while the anodic chamber was charged with $TBAPF_6$ (232.5 mg, 0.60 mmol, 1.50 equiv.). Then, anhydrous degassed 1,4-dioxane (1.0 mL) and DMF (1.0 mL) were added to the cathodic chamber, while the anodic chamber was charged with DMF (2.0 mL). Subsequently, hex-5-en-1-yl 2-(3-cyano-4-isobutoxyphenyl)-4-methylthiazole-5-carboxylate (318.6 mg, 0.80 mmol, 2.00 equiv.) and CF_3I–Al–fum (179.4 mg, 0.40 mmol, 1.00 equiv.) were added to the cathodic chamber. Then, the reaction mixture was stirred at a constant cell potential of 3.2 V at room temperature for 7 h. After completion of the reaction, Et_3N (223 μL, 1.60 mmol, 4.00 equiv.) and pinacol (189.0 mg, 1.60 mmol, 4.00 equiv.) were added to the cathodic chamber, and the mixture was stirred for another 1 h. The crude product was purified by flash chromatography (SiO_2, gradient of 0% → 30% Et_2O in pentane) to afford compound **27** (130.7 mg, 55%) as a colorless oil.

^{1}H NMR (500 MHz, $CDCl_3$): δ 8.17 (d, J = 2.3 Hz, 1H), 8.08 (dd, J = 8.8, 2.2 Hz, 1H), 7.01 (d, J = 8.9 Hz, 1H), 4.28 (t, J = 6.5 Hz, 2H), 3.90 (d, J = 6.5 Hz, 2H), 2.76 (s, 3H), 2.41–2.03 (m, 3H), 1.95–1.68 (m, 3H), 1.64–1.39 (m, 4H), 1.23 (s, 12H), 1.09 (d, J = 6.7 Hz, 6H) ppm.
^{13}C NMR (126 MHz, $CDCl_3$): δ 167.27, 162.61, 162.16, 161.27, 132.67, 132.63, 132.20, 132.16, 127.47 (q, J = 277.0 Hz), 126.12, 126.09, 121.91, 121.80, 115.48, 112.74, 103.10, 83.72, 77.36, 75.81, 65.30, 65.13, 64.91, 39.18, 35.20 (q, J = 27.9 Hz), 30.56, 29.81, 28.84, 28.27, 27.77, 26.27, 25.19, 24.87, 24.80, 24.77, 24.70, 19.16, 17.65, 17.59 ppm.
^{19}F NMR (470 MHz, $CDCl_3$): δ −64.97 (td, J = 11.1, 2.8 Hz) ppm.
^{11}B NMR (160 MHz, $CDCl_3$): δ 32.62 ppm.
MS (DART) m/z: $[M+NH_4]^+$ Calcd. for $C_{29}H_{39}BF_3N_2O_5S^+$: 595.2625; Found: 595.2618.

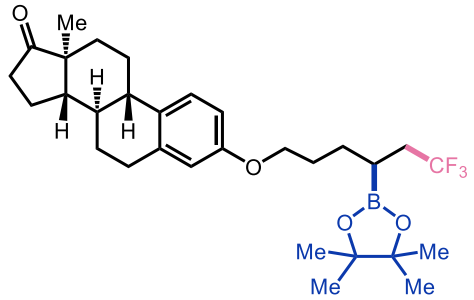

(8R,9S,13S,14S)-13-methyl-3-(((S)-6,6,6-trifluoro-4-(4,4,5,5-tetramethyl-1,3,2-dioxaborolan-2-yl)hexyl)oxy)-6,7,8,9,11,12,13,14,15,16-decahydro-17H-cyclopenta[a]phenanthren-17-one (28)

Following **General Procedure A**, B_2Cat_2 (190.2 mg, 0.80 mmol, 2.00 equiv.) and $TBAPF_6$ (232.5 mg, 0.60 mmol, 1.50 equiv.) were combined in the cathodic chamber of the H-cell, while the anodic chamber was charged with $TBAPF_6$ (232.5 mg, 0.60 mmol, 1.50 equiv.). Then, anhydrous degassed 1,4-dioxane (1.0 mL) and DMF (1.0 mL) were added to the cathodic chamber, while the anodic chamber was charged with DMF (2.0 mL). Subsequently, (8R,9S,13S,14S)-13-methyl-3-(pent-4-en-1-yloxy)-6,7,8,9,11,12,13,14,15,16-decahydro-17H-cyclopenta[a]phenanthren-17-one (270.6 mg, 0.80 mmol, 2.00 equiv.) and CF_3I–Al–fum (179.4 mg, 0.40 mmol, 1.00 equiv.) were added to the cathodic chamber. Then, the reaction mixture was stirred at a constant cell potential of 3.2 V at room temperature for 7 h. After completion of the reaction, Et_3N (223 µL, 1.60 mmol, 4.00 equiv.) and pinacol (189.0 mg, 1.60 mmol, 4.00 equiv.) were added to the cathodic chamber, and the mixture was stirred for another 1 h. The crude product was purified by flash chromatography (SiO_2, gradient of 0% → 10% Et_2O in pentane) to afford compound **28** (111.1 mg, 52%) as a colorless oil.

^{1}H NMR (500 MHz, $CDCl_3$): δ 7.18 (dd, J = 8.7, 1.0 Hz, 1H), 6.70 (dd, J = 8.6, 2.7 Hz, 1H), 6.63 (d, J = 2.7 Hz, 1H), 3.93 (t, J = 6.4 Hz, 2H), 3.00–2.84 (m, 2H), 2.63–2.45 (m, 1H), 2.44–2.21 (m, 3H), 2.19–1.91 (m, 5H), 1.79 (ddt, J = 12.8, 8.4, 4.7 Hz, 2H), 1.71–1.55 (m, 4H), 1.51–1.31 (m, 4H), 1.24 (s, 13H), 0.91 (s, 3H) ppm.
^{13}C NMR (126 MHz, $CDCl_3$): δ 221.05, 157.11, 137.81, 132.05, 127.42(q, J = 277.0 Hz), 126.41, 114.65, 114.64, 112.26, 83.74, 77.36, 67.70, 50.53, 48.13, 44.10, 38.51, 35.99, 35.24 (q, J = 28.0 Hz), 31.71, 29.77, 28.44, 27.43, 26.68, 26.05, 25.14, 24.88, 24.79, 21.70, 13.97 ppm.
^{19}F NMR (470 MHz, $CDCl_3$): δ −64.92 (t, J = 11.2 Hz) ppm.
^{11}B NMR (160 MHz, $CDCl_3$): δ 31.48 ppm.
MS (DART) m/z: $[M+H]^+$ Calcd. for $C_{30}H_{43}BF_3O_4^+$: 535.3201; Found: 535.3238.

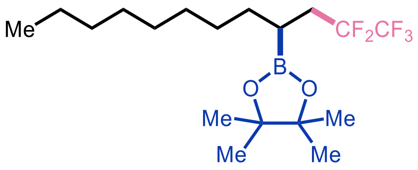

4,4,5,5-tetramethyl-2-(1,1,1,2,2-pentafluorododecan-4-yl)-1,3,2-dioxaborolane (29)

Following **General Procedure A**, B_2Cat_2 (190.2 mg, 0.80 mmol, 2.00 equiv.) and $TBAPF_6$ (232.5 mg, 0.60 mmol, 1.50 equiv.) were combined in the cathodic chamber of the H-cell, while the anodic chamber was charged with $TBAPF_6$ (232.5 mg, 0.60 mmol, 1.50 equiv.). Then, anhydrous degassed 1,4-dioxane (1.0 mL) and DMF (1.0 mL) were added to the cathodic chamber, while the anodic chamber was charged with DMF (2.0 mL). Subsequently, 1-decene (151 µL, 0.80 mmol, 2.00 equiv.) and CF_3CF_2I–Al–fum (220.0 mg, 0.40 mmol, 1.00 equiv.) were added to the cathodic chamber. Then, the reaction mixture was stirred at a constant cell potential of 3.2 V at room temperature for 7 h. After completion of the reaction, Et_3N (223 µL, 1.60 mmol, 4.00 equiv.) and pinacol (189.0 mg, 1.60 mmol, 4.00 equiv.) were added to the cathodic chamber, and the mixture was stirred for another 1 h. The crude product was purified by flash chromatography (SiO_2, gradient of 0% → 5% Et_2O in pentane) to afford compound **29** (111.2 mg, 72%) as a colorless oil.

^{1}H NMR (500 MHz, $CDCl_3$): δ 2.37–2.16 (m, 1H), 1.99 (dddd, *J* = 29.4, 14.7, 10.4, 3.9 Hz, 1H), 1.49 (dq, *J* = 14.4, 7.0 Hz, 1H), 1.44–1.20 (m, 26H), 0.87 (t, *J* = 6.9 Hz, 3H) ppm.
^{13}C NMR (126 MHz, $CDCl_3$): δ 134.16–110.31 (m), 83.62, 32.16 (t, *J* = 21.6 Hz), 31.98, 31.56, 29.79, 29.58, 29.38, 28.74, 24.90, 24.74, 22.83, 14.23 ppm.
^{19}F NMR (470 MHz, $CDCl_3$): δ −85.74 (s, 3F), −115.92–−117.85 (m, 2F) ppm.
^{11}B NMR (160 MHz, $CDCl_3$): δ 33.91 ppm.
MS (DART) m/z: $[M+H]^+$ Calcd. for $C_{18}H_{33}BF_5O_2^+$: 387.2488; Found: 387.2497.

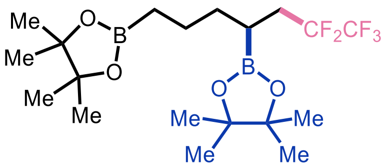

2,2'-(6,6,7,7,7-pentafluoroheptane-1,4-diyl)bis(4,4,5,5-tetramethyl-1,3,2-dioxaborolane) (30)

Following **General Procedure A**, B_2Cat_2 (190.2 mg, 0.80 mmol, 2.00 equiv.) and $TBAPF_6$ (232.5 mg, 0.60 mmol, 1.50 equiv.) were combined in the cathodic chamber of the H-cell, while the anodic chamber was charged with $TBAPF_6$ (232.5 mg, 0.60 mmol, 1.50 equiv.). Then, anhydrous degassed 1,4-dioxane (1.0 mL) and DMF (1.0 mL) were added to the cathodic chamber, while the anodic chamber was charged with DMF (2.0 mL). Subsequently, 4,4,5,5-tetramethyl-2-(pent-4-en-1-yl)-1,3,2-dioxaborolane (157.0 mg, 0.80 mmol, 2.00 equiv.) and CF_3CF_2I–Al–fum (220.0 mg, 0.40 mmol, 1.00 equiv.) were added to the cathodic chamber. Then, the reaction mixture was stirred at a constant cell potential of 3.2 V at room temperature for 7 h. After completion of the reaction, Et_3N (223 µL, 1.60 mmol, 4.00 equiv.) and pinacol (189.0 mg, 1.60 mmol, 4.00 equiv.) were added to the cathodic chamber, and the mixture was stirred for another 1 h. The crude product was purified by flash chromatography (SiO_2, gradient of 0% → 5% Et_2O in pentane) to afford compound **30** (123.8 mg, 70%) as a colorless oil.

^{1}H NMR (500 MHz, $CDCl_3$): δ 2.32–2.13 (m, 1H), 2.00 (dddd, J = 29.4, 14.5, 10.4, 3.6 Hz, 1H), 1.55–1.32 (m, 5H), 1.23 (d, J = 3.4 Hz, 24H), 0.77 (t, J = 6.9 Hz, 2H) ppm.
^{13}C NMR (126 MHz, $CDCl_3$): δ 120.51–114.46 (m), 83.57, 83.06, 77.37, 34.05, 31.96 (t, J = 21.6 Hz), 24.94, 24.90, 24.71, 23.20 ppm.
^{19}F NMR (470 MHz, $CDCl_3$): δ −85.68 (s, 3F), −115.93 –−117.83 (m, 2F) ppm.
^{11}B NMR (160 MHz, $CDCl_3$): δ 34.23 ppm.
MS (DART) m/z: $[M+H]^+$ Calcd. for $C_{19}H_{34}B_2F_5O_4^+$: 443.2558; Found: 443.2577.

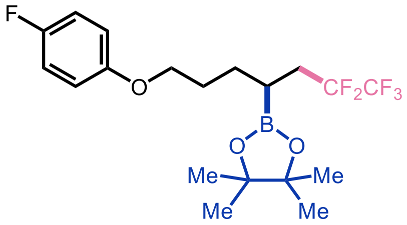

4,4,5,5-tetramethyl-2-(1,1,1,2,2-pentafluoro-7-(4-fluorophenoxy)heptan-4-yl)-1,3,2-dioxaborolane (31)

Following **General Procedure A**, B_2Cat_2 (190.2 mg, 0.80 mmol, 2.00 equiv.) and $TBAPF_6$ (232.5 mg, 0.60 mmol, 1.50 equiv.) were combined in the cathodic chamber of the H-cell, while the anodic chamber was charged with $TBAPF_6$ (232.5 mg, 0.60 mmol, 1.50 equiv.). Then, anhydrous degassed 1,4-dioxane (1.0 mL) and DMF (1.0 mL) were added to the cathodic chamber, while the anodic chamber was charged with DMF (2.0 mL). Subsequently, 1-fluoro-4-(pent-4-en-1-yloxy)benzene (144.1 mg, 0.80 mmol, 2.00 equiv.) and CF_3CF_2I–Al–fum (220.0 mg, 0.40 mmol, 1.00 equiv.) were added to the cathodic chamber. Then, the reaction mixture was stirred at a constant cell potential of 3.2 V at room temperature for 7 h. After completion of the reaction, Et_3N (223 µL, 1.60 mmol, 4.00 equiv.) and pinacol (189.0 mg, 1.60 mmol, 4.00 equiv.) were added to the cathodic chamber, and the mixture was stirred for another 1 h. The crude product was purified by flash chromatography (SiO_2, gradient of 0% → 5% Et_2O in pentane) to afford compound **31** (109.1 mg, 64%) as a colorless oil.

^{1}H NMR (500 MHz, $CDCl_3$): δ 6.95 (t, J = 8.7 Hz, 2H), 6.81 (dd, J = 9.1, 4.3 Hz, 2H), 3.91 (t, J = 6.3 Hz, 2H), 2.29 (ddt, J = 29.7, 15.2, 9.2 Hz, 1H), 2.04 (dddd, J = 29.6, 15.1, 10.5, 4.5 Hz, 1H), 1.87–1.76 (m, 2H), 1.72–1.60 (m, 2H), 1.50–1.38 (m, 1H), 1.24 (s, 12H) ppm.
^{13}C NMR (126 MHz, $CDCl_3$): δ 158.26, 156.37, 155.21, 155.20, 120.46–114.10 (m), 115.96, 115.78, 115.60, 115.54, 83.82, 68.39, 32.00 (t, J = 21.7 Hz), 28.42, 27.88, 24.89, 24.78 ppm.
^{19}F NMR (470 MHz, $CDCl_3$): δ −85.70 (s, 3F), −115.96–−117.70 (m, 2F), −124.33 (tt, J = 8.4, 4.3 Hz, 1F) ppm.
^{11}B NMR (160 MHz, $CDCl_3$): δ 32.88 ppm.
MS (DART) m/z: $[M+H]^+$ Calcd. for $C_{19}H_{26}BF_6O_3^+$: 427.1874; Found: 427.1857.

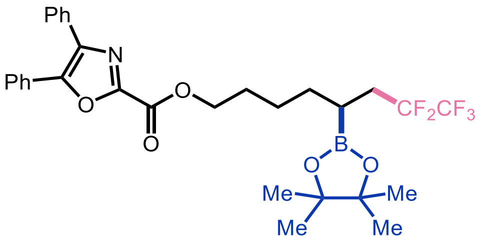

7,7,8,8,8-pentafluoro-5-(4,4,5,5-tetramethyl-1,3,2-dioxaborolan-2-yl)octyl 4,5-diphenyloxazole-2-carboxylate (32)

Following **General Procedure A**, B_2Cat_2 (190.2 mg, 0.80 mmol, 2.00 equiv.) and $TBAPF_6$ (232.5 mg, 0.60 mmol, 1.50 equiv.) were combined in the cathodic chamber of the H-cell, while the anodic chamber was charged with $TBAPF_6$ (232.5 mg, 0.60 mmol, 1.50 equiv.). Then, anhydrous degassed 1,4-dioxane (1.0 mL) and DMF (1.0 mL) were added to the cathodic chamber, while the anodic chamber was charged with DMF (2.0 mL). Subsequently, hex-5-en-1-yl 4,5-diphenyloxazole-2-carboxylate (277.7 mg, 0.80 mmol, 2.00 equiv.) and CF_3CF_2I–Al–fum (220.0 mg, 0.40 mmol, 1.00 equiv.) were added to the cathodic chamber. Then, the reaction mixture was stirred at a constant cell potential of 3.2 V at room temperature for 7 h. After completion of the reaction, Et_3N (223 μL, 1.60 mmol, 4.00 equiv.) and pinacol (189.0 mg, 1.60 mmol, 4.00 equiv.) were added to the cathodic chamber, and the mixture was stirred for another 1 h. The crude product was purified by flash chromatography (SiO_2, gradient of 0% → 20% Et_2O in pentane) to afford compound **32** (116.3 mg, 49%) as a colorless oil.

^{1}H NMR (500 MHz, $CDCl_3$): δ 7.63 (d, J = 6.9 Hz, 2H), 7.57 (d, J = 6.8 Hz, 2H), 7.40–7.29 (m, 6H), 4.11 (t, J = 6.7 Hz, 2H), 3.18 (t, J = 7.6 Hz, 2H), 2.90 (t, J = 7.6 Hz, 2H), 1.65 (q, J = 6.8 Hz, 2H), 1.413–1.32 (m, 3H), 1.23 (s, 12H) ppm.
^{13}C NMR (126 MHz, $CDCl_3$): δ 172.19, 161.92, 145.56, 135.24, 132.54, 129.09, 128.77, 128.69, 128.60, 128.21, 128.04, 126.60, 122.76–114.61 (m), 83.73, 64.82, 31.99 (t, J = 21.7 Hz), 31.28, 31.04, 28.73, 25.16, 25.11, 24.89, 24.75, 24.72, 23.68 ppm.
^{19}F NMR (470 MHz, $CDCl_3$): δ −85.67 (s, 3F), −116.47–−117.71 (m, 2F) ppm.
^{11}B NMR (160 MHz, $CDCl_3$): δ 31.91 ppm.
MS (DART) m/z: $[M+H]^+$ Calcd. for $C_{30}H_{34}BF_5NO_5^+$: 594.2445; Found: 594.2471.

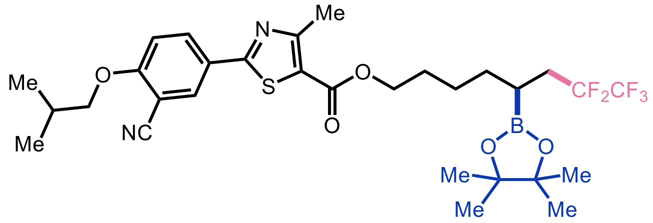

7-((difluoromethylene)-l3-fluoraneyl)-7,7-difluoro-5-(4,4,5,5-tetramethyl-1,3,2-dioxaborolan-2-yl)heptyl 2-(3-cyano-4-isobutoxyphenyl)-4-methylthiazole-5-carboxylate (33)

Following **General Procedure A**, B_2Cat_2 (190.2 mg, 0.80 mmol, 2.00 equiv.) and $TBAPF_6$ (232.5 mg, 0.60 mmol, 1.50 equiv.) were combined in the cathodic chamber of the H-cell, while the anodic chamber was charged with $TBAPF_6$ (232.5 mg, 0.60 mmol, 1.50 equiv.). Then, anhydrous degassed 1,4-dioxane (1.0 mL) and DMF (1.0 mL) were added to the cathodic chamber, while the anodic chamber was charged with DMF (2.0 mL). Subsequently, hex-5-en-1-yl 2-(3-cyano-4-isobutoxyphenyl)-4-methylthiazole-5-carboxylate (318.6 mg, 0.80 mmol, 2.00 equiv.) and CF_3CF_2I–Al–fum (220.0 mg, 0.40 mmol, 1.00 equiv.) were added to the cathodic chamber. Then, the reaction mixture was stirred at a constant cell potential of 3.2 V at room temperature for 7 h. After completion of the reaction, Et_3N (223 µL, 1.60 mmol, 4.00 equiv.) and pinacol (189.0 mg, 1.60 mmol, 4.00 equiv.) were added to the cathodic chamber, and the mixture was stirred for another 1 h. The crude product was purified by flash chromatography (SiO_2, gradient of 0% → 25% Et_2O in pentane) to afford compound **33** (144.3 mg, 56%) as a white solid.

^{1}H NMR (500 MHz, $CDCl_3$): δ 8.16 (d, J = 2.3 Hz, 1H), 8.08 (dd, J = 8.8, 2.3 Hz, 1H), 7.00 (d, J = 8.9 Hz, 1H), 4.28 (t, J = 6.5 Hz, 2H), 3.89 (d, J = 6.5 Hz, 2H), 2.75 (s, 3H), 2.33–2.16 (m, 2H), 2.01 (dddd, J = 27.7, 12.9, 8.5, 3.3 Hz, 1H), 1.76 (pt, J = 10.0, 5.0 Hz, 2H), 1.58 (dt, J = 10.0, 5.3 Hz, 1H), 1.53–1.43 (m, 3H), 1.38 (dtd, J = 11.2, 7.6, 3.3 Hz, 1H), 1.23 (d, J = 1.6 Hz, 12H), 1.09 (d, J = 6.7 Hz, 6H) ppm.
^{13}C NMR (126 MHz, $CDCl_3$): δ 167.31, 162.63, 162.18, 161.30, 132.64, 132.19, 126.14, 121.92, 115.50, 112.74, 103.13, 83.75, 75.83, 65.28, 31.95, 31.04, 28.85, 28.29, 25.22, 25.15, 24.88, 24.75, 19.18, 17.60 ppm.
^{19}F NMR (470 MHz, $CDCl_3$): δ −85.67 (s, 3F), −115.98–−117.67 (m, 2F) ppm.
^{11}B NMR (160 MHz, $CDCl_3$): δ 33.06 ppm.
MS (DART) m/z: $[M+H]^+$ Calcd. for $C_{30}H_{39}BF_5N_2O_5S^+$: 645.2587; Found: 645.2529.
Melting Point: 312.1−314.4 °C.

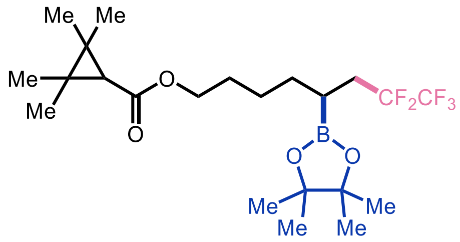

7,7,8,8,8-pentafluoro-5-(4,4,5,5-tetramethyl-1,3,2-dioxaborolan-2-yl)octyl 2,2,3,3-tetramethylcyclopropane-1-carboxylate (34)

Following **General Procedure A**, B_2Cat_2 (190.2 mg, 0.80 mmol, 2.00 equiv.) and $TBAPF_6$ (232.5 mg, 0.60 mmol, 1.50 equiv.) were combined in the cathodic chamber of the H-cell, while the anodic chamber was charged with $TBAPF_6$ (232.5 mg, 0.60 mmol, 1.50 equiv.). Then, anhydrous degassed 1,4-dioxane (1.0 mL) and DMF (1.0 mL) were added to the cathodic chamber, while the anodic chamber was charged with DMF (2.0 mL). Subsequently, hex-5-en-1-yl 2,2,3,3-tetramethylcyclopropane-1-carboxylate (144.1 mg, 0.80 mmol, 2.00 equiv.) and CF_3CF_2I–Al–fum (220.0 mg, 0.40 mmol, 1.00 equiv.) were added to the cathodic chamber. Then, the reaction mixture was stirred at a constant cell potential of 3.2 V at room temperature for 7 h. After completion of the reaction, Et_3N (223 µL, 1.60 mmol, 4.00 equiv.) and pinacol (189.0 mg, 1.60 mmol, 4.00 equiv.) were added to the cathodic chamber, and the mixture was stirred for another 1 h. The crude product was purified by flash chromatography (SiO_2, gradient of 0% → 15% Et_2O in pentane) to afford compound **34** (126.0 mg, 67%) as a colorless oil.

^{1}H NMR (500 MHz, $CDCl_3$): δ 4.00 (t, J = 6.6 Hz, 2H), 2.24 (ddt, J = 29.7, 15.1, 9.0 Hz, 1H), 1.99 (dddd, J = 29.4, 14.8, 10.4, 4.2 Hz, 1H), 1.65–1.57 (m, 2H), 1.52 (ddd, J = 11.2, 8.1, 4.7 Hz, 1H), 1.47–1.33 (m, 4H), 1.22 (s, 18H), 1.16 (s, 6H), 1.14 (s, 1H) ppm.
^{13}C NMR (126 MHz, $CDCl_3$): δ 172.39, 122.74–114.39 (m), 114.10, 83.70, 63.57, 35.90, 32.02 (t, J = 21.6 Hz), 31.14, 30.07, 28.95, 25.26, 23.66, 16.69 ppm.
^{19}F NMR (470 MHz, $CDCl_3$): δ −85.72 (s, 3F), −115.98–−117.78 (m, 2F) ppm.
^{11}B NMR (160 MHz, $CDCl_3$): δ 33.49 ppm.
MS (DART) m/z: $[M+H]^+$ Calcd. for $C_{22}H_{37}BF_5O_4^+$: 471.2700; Found: 471.2731.

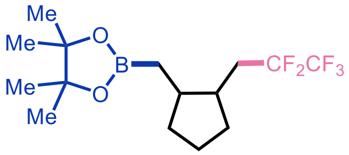

4,4,5,5-tetramethyl-2-((2-(2,2,3,3,3-pentafluoropropyl)cyclopentyl)methyl)-1,3,2-dioxaborolane (36)

Following **General Procedure A**, B_2Cat_2 (190.2 mg, 0.80 mmol, 2.00 equiv.) and $TBAPF_6$ (232.5 mg, 0.60 mmol, 1.50 equiv.) were combined in the cathodic chamber of the H-cell, while the anodic chamber was charged with $TBAPF_6$ (232.5 mg, 0.60 mmol, 1.50 equiv.). Then, anhydrous degassed 1,4-dioxane (1.0 mL) and DMF (1.0 mL) were added to the cathodic chamber, while the anodic chamber was charged with DMF (2.0 mL). Subsequently, hepta-1,6-diene (108 µL, 0.80 mmol, 2.00 equiv.) and CF_3CF_2I–Al–fum (220.0 mg, 0.40 mmol, 1.00 equiv.) were added to the cathodic chamber. Then, the reaction mixture was stirred at a constant cell potential of 3.2 V at room temperature for 7 h. After completion of the reaction, Et_3N (223 µL, 1.60 mmol, 4.00 equiv.) and pinacol (189.0 mg, 1.60 mmol, 4.00 equiv.) were added to the cathodic chamber, and the mixture was stirred for another 1 h. The crude product was purified by flash chromatography (SiO_2, gradient of 0% → 5% Et_2O in pentane) to afford compound **36** (93.1 mg, 68%) as a colorless oil.

^{1}H NMR (500 MHz, $CDCl_3$): δ 2.26–2.09 (m, 3H), 1.94–1.73 (m, 3H), 1.69 (dddd, J = 15.6, 9.0, 4.5, 1.8 Hz, 1H), 1.57 (ddtd, J = 14.7, 12.0, 8.7, 4.2 Hz, 1H), 1.44–1.38 (m, 1H), 1.30 (ddt, J = 12.6, 8.7, 4.8 Hz, 1H), 1.23 (d, J = 2.7 Hz, 12H), 0.71 (dd, J = 15.3, 5.4 Hz, 1H), 0.60 (dd, J = 15.3, 9.4 Hz, 1H) ppm.
^{13}C NMR (126 MHz, $CDCl_3$): δ 123.14–114.44 (m), 83.26, 42.50, 40.91, 38.66, 36.10, 36.08, 34.73 (t, J = 21.4 Hz), 33.61, 32.91, 32.87, 32.85, 31.00 (t, J = 21.2 Hz), 30.18, 30.16, 25.01, 24.99, 24.82, 24.80, 22.39 ppm.
^{19}F NMR (470 MHz, $CDCl_3$): δ −85.88 (s, 3F), −116.19–−118.92 (m, 2F) ppm.
^{11}B NMR (160 MHz, $CDCl_3$): δ 33.96 ppm.
MS (DART) m/z: $[M+H]^+$ Calcd. for $C_{15}H_{25}BF_5O_2^+$: 343.1862; Found: 343.1889.

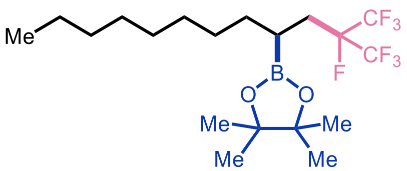

4,4,5,5-tetramethyl-2-(1,1,1,2-tetrafluoro-2-(trifluoromethyl)dodecan-4-yl)-1,3,2-dioxaborolane (37)

Following **General Procedure B**, B_2Cat_2 (190.2 mg, 0.80 mmol, 2.00 equiv.) and $TBAPF_6$ (232.5 mg, 0.60 mmol, 1.50 equiv.) were combined in the cathodic chamber of the H-cell, while the anodic chamber was charged with $TBAPF_6$ (232.5 mg, 0.60 mmol, 1.50 equiv.). Then, anhydrous degassed 1,4-dioxane (1.0 mL) and DMF (1.0 mL) were added to the cathodic chamber, while the anodic chamber was charged with DMF (2.0 mL). Subsequently, 1-decene (152 μL, 0.80 mmol, 2.00 equiv.) and heptafluoro-2-iodopropane (56.9 μL, 0.40 mmol, 1.00 equiv.) were added to the cathodic chamber. Then, the reaction mixture was stirred at a constant cell potential of 3.2 V at room temperature for 7 h. After completion of the reaction, Et_3N (223 μL, 1.60 mmol, 4.00 equiv.) and pinacol (189.0 mg, 1.60 mmol, 4.00 equiv.) were added to the cathodic chamber, and the mixture was stirred for another 1 h. The crude product was purified by flash chromatography (SiO_2, gradient of 0% → 5% Et_2O in pentane) to afford compound **37** (104.7 mg, 60%) as a colorless oil.

^{1}H NMR (500 MHz, $CDCl_3$): δ 2.36 (q, J = 12.6 Hz, 1H), 1.98 (dd, J = 27.9, 15.3 Hz, 1H), 1.49–1.42 (m, 1H), 1.38–1.23 (m, 26H), 0.87 (t, J = 6.9 Hz, 3H) ppm.
^{13}C NMR (126 MHz, $CDCl_3$): δ 124.94–117.80 (m), 93.57–90.96 (m), 83.61, 32.24, 32.00, 30.29, 30.13, 29.78, 29.56, 29.37, 28.62, 24.91, 24.71, 22.82, 14.22 ppm.
^{19}F NMR (470 MHz, $CDCl_3$): δ −76.22 (p, J = 9.0 Hz, 3F), −77.45 (P, J = 9.0 Hz, 3F), −183.74 (ddp, J = 26.3, 13.2, 6.7 Hz, 1F) ppm.
^{11}B NMR (160 MHz, $CDCl_3$): δ 34.58 ppm.
MS (DART) m/z: $[M+H]^+$ Calcd. for $C_{19}H_{33}BF_7O_2^+$: 437.2456; Found: 437.2473.

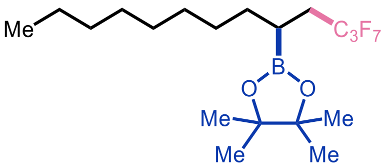

2-(1,1,1,1,1,1,1-heptafluoro-1l8-tridec-2-yn-5-yl)-4,4,5,5-tetramethyl-1,3,2-dioxaborolane (38)

Following **General Procedure B**, B_2Cat_2 (190.2 mg, 0.80 mmol, 2.00 equiv.) and $TBAPF_6$ (232.5 mg, 0.60 mmol, 1.50 equiv.) were combined in the cathodic chamber of the H-cell, while the anodic chamber was charged with $TBAPF_6$ (232.5 mg, 0.60 mmol, 1.50 equiv.). Then, anhydrous degassed 1,4-dioxane (1.0 mL) and DMF (1.0 mL) were added to the cathodic chamber, while the anodic chamber was charged with DMF (2.0 mL). Subsequently, 1-decene (152 µL, 0.80 mmol, 2.00 equiv.) and 1,1,1,2,2,3,3-heptafluoro-3-iodopropane (57.4 µL, 0.40 mmol, 1.00 equiv.) were added to the cathodic chamber. Then, the reaction mixture was stirred at a constant cell potential of 3.2 V at room temperature for 7 h. After completion of the reaction, Et_3N (223 µL, 1.60 mmol, 4.00 equiv.) and pinacol (189.0 mg, 1.60 mmol, 4.00 equiv.) were added to the cathodic chamber, and the mixture was stirred for another 1 h. The crude product was purified by flash chromatography (SiO_2, gradient of 0% → 5% Et_2O in pentane) to afford compound **38** (116.9 mg, 67%) as a colorless oil.

^{1}H NMR (500 MHz, $CDCl_3$): δ 2.34–2.21 (m, 1H), 2.08–1.95 (m, 1H), 1.49 (tdd, J = 10.1, 6.9, 2.8 Hz, 1H), 1.43–1.35 (m, 2H), 1.32–1.21 (m, 24H), 0.87 (t, J = 6.9 Hz, 3H) ppm.
^{13}C NMR (126 MHz, $CDCl_3$): δ 120.52–116.01 (m), 111.25–106.77 (m), 83.62, 32.22 (t, J = 21.7 Hz), 32.02, 31.58, 29.79, 29.58, 29.38, 28.78, 24.90, 24.72, 22.83, 14.22 ppm.
^{19}F NMR (470 MHz, $CDCl_3$): δ −80.61 (t, J = 9.6 Hz, 3F), −113.78 (qdt, J = 269.7, 31.3, 9.6 Hz, 2F), −128.04 (s, 2F) ppm.
^{11}B NMR (160 MHz, $CDCl_3$): δ 34.72 ppm.
MS (DART) m/z: $[M+H]^+$ Calcd. for $C_{19}H_{33}BF_7O_2^+$: 437.2456; Found: 437.2432.

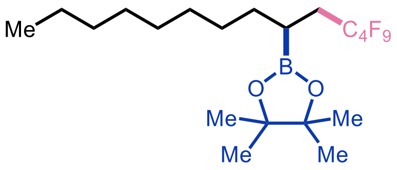

4,4,5,5-tetramethyl-2-(1,1,1,1,1,1,1,1-nonafluoro-1l12-tetradeca-1,3-diyn-6-yl)-1,3,2-dioxaborolane (39)

Following **General Procedure B**, B_2Cat_2 (190.2 mg, 0.80 mmol, 2.00 equiv.) and $TBAPF_6$ (232.5 mg, 0.60 mmol, 1.50 equiv.) were combined in the cathodic chamber of the H-cell, while the anodic chamber was charged with $TBAPF_6$ (232.5 mg, 0.60 mmol, 1.50 equiv.). Then, anhydrous degassed 1,4-dioxane (1.0 mL) and DMF (1.0 mL) were added to the cathodic chamber, while the anodic chamber was charged with DMF (2.0 mL). Subsequently, 1-decene (152 μL, 0.80 mmol, 2.00 equiv.) and 1,1,1,2,2,3,3,4,4-nonafluoro-4-iodobutane (68.8 μL, 0.40 mmol, 1.00 equiv.) were added to the cathodic chamber. Then, the reaction mixture was stirred at a constant cell potential of 3.2 V at room temperature for 7 h. After completion of the reaction, Et_3N (223 μL, 1.60 mmol, 4.00 equiv.) and pinacol (189.0 mg, 1.60 mmol, 4.00 equiv.) were added to the cathodic chamber, and the mixture was stirred for another 1 h. The crude product was purified by flash chromatography (SiO_2, gradient of 0% → 5% Et_2O in pentane) to afford compound **39** (159.5 mg, 82%) as a colorless oil.

^{1}H NMR (500 MHz, $CDCl_3$): δ 2.36–2.23 (m, 1H), 2.09–1.96 (m, 1H), 1.49 (qd, J = 9.3, 6.9 Hz, 1H), 1.43–1.34 (m, 2H), 1.30–1.23 (m, 24H), 0.87 (t, J = 6.9 Hz, 3H) ppm.
^{13}C NMR (126 MHz, $CDCl_3$): δ 120.96 (q, J = 31.4 Hz), 119.05–116.22 (m), 114.46–112.48 (m), 111.27–106.42 (m), 83.63, 32.42 (t, J = 21.9 Hz), 32.02, 31.60, 29.80, 29.59, 29.39, 28.79, 24.89, 24.71, 22.84, 14.21 ppm.
^{19}F NMR (470 MHz, $CDCl_3$): δ −81.17 (td, J = 9.6, 5.0 Hz, 3F), −107.54 –−116.08 (m, 2F), −124.72 (q, J = 9.2 Hz, 2F), −126.05 (td, J = 12.4, 4.4 Hz, 2F) ppm.
^{11}B NMR (160 MHz, $CDCl_3$): δ 34.89 ppm.
MS (DART) m/z: $[M+H]^+$ Calcd. for $C_{20}H_{33}BF_9O_2^+$: 487.2424; Found: 487.2448.

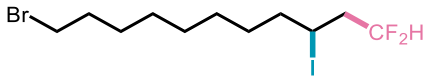

11-bromo-1,1-difluoro-3-iodoundecane (40)

Following **General Procedure D**, $TBAPF_6$ (309.8 mg, 0.80 mmol, 2.00 equiv.), $Me_3N{\cdot}BH_3$ (145.9 mg, 2.00 mmol, 5.00 equiv.) and anhydrous degassed DMF (4.0 mL) were combined in an oven-dried 20 mL glass vial. Then, 10-bromodec-1-ene (79.9 µL, 0.40 mmol, 1.00 equiv.) and CF_2HI–Al–fum (134.8 mg, 0.80 mmol, 2.00 equiv.) were added sequentially. The vial was sealed, transferred out of the glovebox, and stirred at a constant cell current of 5 mA at 22 °C for 13 h. The crude product was purified by flash chromatography (SiO_2, gradient of 0% → 5% Et_2O in pentane) to afford compound **40** (102.9 mg, 65%) as a colorless oil.

^{1}H NMR (500 MHz, $CDCl_3$): δ 6.19–5.90 (m, 1H), 4.05 (ddt, *J* = 10.0, 8.7, 5.3 Hz, 1H), 3.41 (t, *J* = 6.8 Hz, 1H), 3.19 (t, *J* = 7.0 Hz, 1H), 2.43–2.27 (m, 2H), 1.90–1.69 (m, 4H), 1.53–1.28 (m, 10H) ppm.
^{13}C NMR (126 MHz, $CDCl_3$): δ 118.53, 118.08 (t, *J* = 239.9 Hz), 116.11, 114.73, 48.93 (dd, *J* = 9.3, 3.7 Hz), 44.43 (t, *J* = 22.5 Hz), 43.47, 43.29, 43.11, 40.69, 39.24, 34.11, 33.60, 32.89, 30.54, 29.32, 29.30, 29.26, 28.83, 28.76, 28.75, 28.62, 28.22, 27.23, 27.04 (dd, *J* = 8.6, 3.9 Hz) ppm.
^{19}F NMR (470 MHz, $CDCl_3$): δ −116.55–−118.56 (m, 2F) ppm.
MS (DART) m/z: $[M+H]^+$ Calcd. for $C_{11}H_{21}BrF_2I^+$: 396.9834; Found: 396.9847.

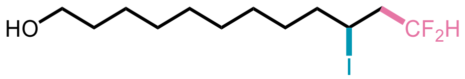

12,12-difluoro-10-iodododecan-1-ol (41)

Following **General Procedure D**, $TBAPF_6$ (309.8 mg, 0.80 mmol, 2.00 equiv.), $Me_3N{\cdot}BH_3$ (145.9 mg, 2.00 mmol, 5.00 equiv.) and anhydrous degassed DMF (4.0 mL) were combined in an oven-dried 20 mL glass vial. Then, undec-10-en-1-ol (80.1 µL, 0.40 mmol, 1.00 equiv.) and CF_2HI–Al–fum (134.8 mg, 0.80 mmol, 2.00 equiv.) were added sequentially. The vial was sealed, transferred out of the glovebox, and stirred at a constant cell current of 5 mA at 22 °C for 13 h. The crude product was purified by flash chromatography (SiO_2, gradient of 5% → 40% Et_2O in pentane) to afford compound **41** (69.6 mg, 50%) as a colorless oil.

^{1}H NMR (500 MHz, $CDCl_3$): δ 6.02 (dddd, *J* = 57.2, 56.0, 6.6, 3.0 Hz, 1H), 4.07–4.02 (m, 1H), 3.63 (t, *J* = 6.6 Hz, 2H), 2.44–2.26 (m, 2H), 1.91–1.79 (m, 1H), 1.72 (ddt, *J* = 14.8, 10.2, 5.3 Hz, 1H), 1.59–1.48 (m, 3H), 1.45–1.25 (m, 12H) ppm.
^{13}C NMR (126 MHz, $CDCl_3$): δ 118.03 (t, *J* = 239.8 Hz), 63.14, 44.41 (t, *J* = 22.5 Hz), 40.71, 32.88, 29.57, 29.53, 29.52, 29.47, 29.43, 29.28, 28.70, 27.11 (dd, *J* = 8.6, 3.9 Hz), 25.83, 1.14 ppm.
^{19}F NMR (470 MHz, $CDCl_3$): δ −116.69–−118.60 (m, 2F) ppm.
MS (DART) m/z: $[M+H]^+$ Calcd. for $C_{12}H_{24}F_2IO^+$: 349.0834; Found: 349.0816.

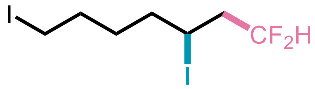

1,1-difluoro-3,7-diiodoheptane (42)

Following **General Procedure D**, TBAPF$_6$ (309.8 mg, 0.80 mmol, 2.00 equiv.), $Me_3N·BH_3$ (145.9 mg, 2.00 mmol, 5.00 equiv.) and anhydrous degassed DMF (4.0 mL) were combined in an oven-dried 20 mL glass vial. Then, 6-iodohex-1-ene (57.9 µL, 0.40 mmol, 1.00 equiv.) and CF_2HI–Al–fum (134.8 mg, 0.80 mmol, 2.00 equiv.) were added sequentially. The vial was sealed, transferred out of the glovebox, and stirred at a constant cell current of 5 mA at 22 °C for 13 h. The crude product was purified by flash chromatography (SiO_2, gradient of 0% → 10% Et_2O in pentane) to afford compound **42** (108.6 mg, 70%) as a colorless oil.

^{1}H NMR (500 MHz, $CDCl_3$): δ 6.02 (dddd, *J* = 56.9, 55.8, 6.5, 2.9 Hz, 1H), 4.07–4.01 (m, 1H), 3.20 (t, *J* = 6.9 Hz, 2H), 2.45–2.28 (m, 2H), 1.94–1.73 (m, 4H), 1.67 (ddp, *J* = 19.6, 10.0, 5.0 Hz, 1H), 1.59–1.50 (m, 1H) ppm.
^{13}C NMR (126 MHz, $CDCl_3$): δ 117.88 (t, *J* = 239.9 Hz), 44.35 (t, *J* = 22.6 Hz), 39.50, 32.44, 30.29, 25.95 (dd, *J* = 8.4, 3.9 Hz), 6.10 ppm.
^{19}F NMR (470 MHz, $CDCl_3$): δ −116.53–−118.56 (m, 2F) ppm.
The NMR data are consistent with the reported data.[36]
MS (DART) m/z: $[M+H]^+$ Calcd. for $C_7H_{13}F_2I_2^+$: 388.9069; Found: 388.9088.

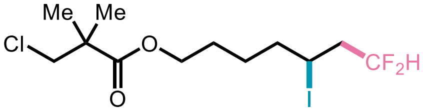

7,7-difluoro-5-iodoheptyl 3-chloro-2,2-dimethylpropanoate (43)

Following **General Procedure D**, TBAPF$_6$ (309.8 mg, 0.80 mmol, 2.00 equiv.), $Me_3N·BH_3$ (145.9 mg, 2.00 mmol, 5.00 equiv.) and anhydrous degassed DMF (4.0 mL) were combined in an oven-dried 20 mL glass vial. Then, hex-5-en-1-yl 3-chloro-2,2-dimethylpropanoate (87.2 mg, 0.40 mmol, 1.00 equiv.) and CF_2HI–Al–fum (134.8 mg, 0.80 mmol, 2.00 equiv.) were added sequentially. The vial was sealed, transferred out of the glovebox, and stirred at a constant cell current of 5 mA at 22 °C for 13 h. The crude product was purified by flash chromatography (SiO_2, gradient of 0% → 10% Et_2O in pentane) to afford compound **43** (112.5 mg, 71%) as a colorless oil.

^{1}H NMR (500 MHz, $CDCl_3$): δ 6.02 (dddd, *J* = 57.0, 55.8, 6.5, 2.9 Hz, 1H), 4.13 (td, *J* = 6.3, 1.1 Hz, 2H), 4.04 (dp, *J* = 8.8, 4.4 Hz, 1H), 3.61 (s, 2H), 2.43–2.26 (m, 2H), 1.90 (dtd, *J* = 18.8, 9.4, 4.1 Hz, 1H), 1.79–1.60 (m, 4H), 1.55–1.45 (m, 1H), 1.29 (s, 6H) ppm.
^{13}C NMR (126 MHz, $CDCl_3$): δ 175.11, 117.88 (t, *J* = 239.9 Hz), 64.52, 52.21, 44.78, 44.37 (t, *J* = 22.6 Hz), 40.06, 27.73, 26.32 (dd, *J* = 8.6, 4.0 Hz), 25.82, 23.41, 1.13 ppm.
^{19}F NMR (470 MHz, $CDCl_3$): δ −116.52–−118.50 (m, 2F) ppm.

MS (DART) m/z: $[M+H]^+$ Calcd. for $C_{12}H_{21}ClF_2IO_2^+$: 397.0237; Found: 397.0211.

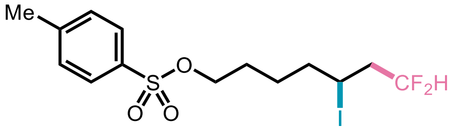

7,7-difluoro-5-iodoheptyl 4-methylbenzenesulfonate (44)

Following **General Procedure D**, $TBAPF_6$ (309.8 mg, 0.80 mmol, 2.00 equiv.), $Me_3N·BH_3$ (145.9 mg, 2.00 mmol, 5.00 equiv.) and anhydrous degassed DMF (4.0 mL) were combined in an oven-dried 20 mL glass vial. Then, hex-5-en-1-yl 4-methylbenzenesulfonate (101.6 mg, 0.40 mmol, 1.00 equiv.) and CF_2HI–Al–fum (134.8 mg, 0.80 mmol, 2.00 equiv.) were added sequentially. The vial was sealed, transferred out of the glovebox, and stirred at a constant cell current of 5 mA at 22 °C for 13 h. The crude product was purified by flash chromatography (SiO_2, gradient of 0% → 10% Et_2O in pentane) to afford compound **44** (115.9 mg, 67%) as a colorless oil.

^{1}H NMR (500 MHz, $CDCl_3$): δ 7.80 (d, J = 8.2 Hz, 2H), 7.36 (d, J = 7.9 Hz, 2H), 6.11–5.87 (m, 1H), 4.06–3.93 (m, 3H), 2.45 (s, 3H), 2.40–2.17 (m, 2H), 1.85–1.76 (m, 1H), 1.74–1.63 (m, 2H), 1.42 (dddd, J = 15.6, 10.1, 7.3, 4.8 Hz, 1H), 1.33–1.25 (m, 2H) ppm.
^{13}C NMR (126 MHz, $CDCl_3$): δ 145.01, 133.17, 130.05, 128.04, 117.82 (t, J = 240.0 Hz), 70.07, 44.30 (t, J = 22.6 Hz), 39.83, 29.83, 27.99, 25.80 (dd, J = 8.6, 4.1 Hz), 25.44, 21.79 ppm.
^{19}F NMR (470 MHz, $CDCl_3$): δ −115.97–−118.42 (m, 2F) ppm.
MS (DART) m/z: $[M+H]^+$ Calcd. for $C_{14}H_{20}F_2IO_3S^+$: 433.0140; Found: 433.0161.

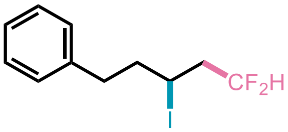

(5,5-difluoro-3-iodopentyl)benzene (45)

Following **General Procedure D**, $TBAPF_6$ (309.8 mg, 0.80 mmol, 2.00 equiv.), $Me_3N·BH_3$ (145.9 mg, 2.00 mmol, 5.00 equiv.) and anhydrous degassed DMF (4.0 mL) were combined in an oven-dried 20 mL glass vial. Then, but-3-en-1-ylbenzene (58.3 µL, 0.40 mmol, 1.00 equiv.) and CF_2HI–Al–fum (134.8 mg, 0.80 mmol, 2.00 equiv.) were added sequentially. The vial was sealed, transferred out of the glovebox, and stirred at a constant cell current of 5 mA at 22 °C for 13 h. The crude product was purified by flash chromatography (SiO_2, gradient of 0% → 5% Et_2O in pentane) to afford compound **45** (75.6 mg, 61%) as a colorless oil.

^{1}H NMR (500 MHz, $CDCl_3$): δ 7.31 (dd, J = 8.1, 6.8 Hz, 2H), 7.23–7.21 (m, 3H), 6.04 (dddd, J = 56.8, 55.8, 6.4, 3.0 Hz, 1H), 4.02 (dt, J = 9.5, 4.4 Hz, 1H), 2.91 (ddd, J = 14.0, 9.2, 5.1 Hz, 1H), 2.74 (ddd, J = 13.7, 9.2, 6.8 Hz, 1H), 2.52–2.28 (m, 2H), 2.20 (dtd, J = 14.4, 9.2, 5.1 Hz, 1H), 2.07 –2.00 (m, 1H) ppm.

^{13}**C NMR** (126 MHz, $CDCl_3$): δ 140.20, 128.76, 128.63, 126.51, 117.92 (t, *J* = 239.9 Hz), 44.45 (t, *J* = 22.6 Hz), 42.26, 35.40, 26.13 (dd, *J* = 8.4, 4.1 Hz) ppm.
^{19}**F NMR** (470 MHz, $CDCl_3$): δ −116.29–−118.26 (m, 2F) ppm.
The NMR data are consistent with the reported data.[36]
MS (DART) m/z: $[M+H]^+$ Calcd. for $C_{11}H_{14}F_2I^+$: 311.0103; Found: 311.0085.

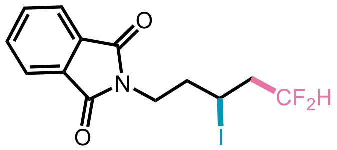

2-(5,5-difluoro-3-iodopentyl)isoindoline-1,3-dione (46)

Following **General Procedure D**, $TBAPF_6$ (309.8 mg, 0.80 mmol, 2.00 equiv.), $Me_3N{\cdot}BH_3$ (145.9 mg, 2.00 mmol, 5.00 equiv.) and anhydrous degassed DMF (4.0 mL) were combined in an oven-dried 20 mL glass vial. Then, 2-(but-3-en-1-yl)isoindoline-1,3-dione (80.4 mg, 0.40 mmol, 1.00 equiv.) and CF_2HI–Al–fum (134.8 mg, 0.80 mmol, 2.00 equiv.) were added sequentially. The vial was sealed, transferred out of the glovebox, and stirred at a constant cell current of 5 mA at 22 °C for 13 h. The crude product was purified by flash chromatography (SiO_2, gradient of 5% → 40% Et_2O in pentane) to afford compound **46** (62.2 mg, 41%) as a colorless oil.

^{1}**H NMR** (500 MHz, $CDCl_3$): δ 7.86 (dd, *J* = 5.5, 3.0 Hz, 2H), 7.73 (dd, *J* = 5.5, 3.0 Hz, 2H), 6.04 (tdd, *J* = 56.3, 6.1, 3.1 Hz, 1H), 4.06–3.69 (m, 3H), 2.52–2.17 (m, 4H) ppm.
^{13}**C NMR** (126 MHz, $CDCl_3$): δ 168.31, 134.30, 132.08, 123.57, 117.76 (t, *J* = 240.1 Hz), 44.06 (t, *J* = 22.9 Hz), 39.08, 37.84, 20.90 (dd, *J* = 8.4, 4.4 Hz) ppm.
^{19}**F NMR** (470 MHz, $CDCl_3$): δ −115.89–−118.35 (m, 2F) ppm.
MS (DART) m/z: $[M+H]^+$ Calcd. for $C_{13}H_{13}F_2INO_2^+$: 379.9954; Found: 379.9989.

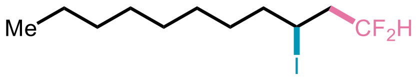

1,1-difluoro-3-iodoundecane (47)

Following **General Procedure D**, $TBAPF_6$ (309.8 mg, 0.80 mmol, 2.00 equiv.), $Me_3N{\cdot}BH_3$ (145.9 mg, 2.00 mmol, 5.00 equiv.) and anhydrous degassed DMF (4.0 mL) were combined in an oven-dried 20 mL glass vial. Then, 1-decene (75.5 µL, 0.40 mmol, 1.00 equiv.) and CF_2HI–Al–fum (134.8 mg, 0.80 mmol, 2.00 equiv.) were added sequentially. The vial was sealed, transferred out of the glovebox, and stirred at a constant cell current of 5 mA at 22 °C for 13 h. The crude product was purified by flash chromatography (SiO_2, gradient of 0% → 5% Et_2O in pentane) to afford compound **47** (68.7 mg, 61%) as a colorless oil.

^{1}H NMR (500 MHz, $CDCl_3$): δ 6.18–5.87 (m, 1H), 4.06 (tt, *J* = 9.3, 4.6 Hz, 1H), 2.46–2.26 (m, 2H), 1.80 (dddt, *J* = 60.0, 14.8, 10.2, 4.9 Hz, 2H), 1.58–1.47 (m, 1H), 1.45–1.38 (m, 1H), 1.28 (q, *J* = 7.0 Hz, 10H), 0.89 (t, *J* = 12.3 Hz, 3H) ppm.
^{13}C NMR (126 MHz, $CDCl_3$): δ 118.06 (t, *J* = 239.8 Hz), 44.44 (t, *J* = 22.5 Hz), 40.77, 31.98, 31.96, 29.86, 29.59, 29.51, 29.48, 29.34, 29.32, 28.77, 27.15 (dd, *J* = 8.6, 3.9 Hz), 22.80, 14.25, 1.16 ppm.
^{19}F NMR (470 MHz, $CDCl_3$): δ −116.57–−118.52 (m, 2F) ppm.
MS (DART) m/z: $[M+H]^+$ Calcd. for $C_{11}H_{22}F_2I^+$: 319.0729; Found: 319.0709.

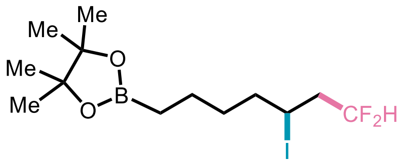

2-(7,7-difluoro-5-iodoheptyl)-4,4,5,5-tetramethyl-1,3,2-dioxaborolane (48)

Following **General Procedure D**, $TBAPF_6$ (309.8 mg, 0.80 mmol, 2.00 equiv.), $Me_3N{\cdot}BH_3$ (145.9 mg, 2.00 mmol, 5.00 equiv.) and anhydrous degassed DMF (4.0 mL) were combined in an oven-dried 20 mL glass vial. Then, 2-(hex-5-en-1-yl)-4,4,5,5-tetramethyl-1,3,2-dioxaborolane (84.1 mg, 0.40 mmol, 1.00 equiv.) and CF_2HI–Al–fum (134.8 mg, 0.80 mmol, 2.00 equiv.) were added sequentially. The vial was sealed, transferred out of the glovebox, and stirred at a constant cell current of 5 mA at 22 °C for 13 h. The crude product was purified by flash chromatography (SiO_2, gradient of 0% → 5% Et_2O in pentane) to afford compound **48** (82.3 mg, 53%) as a colorless oil.

^{1}H NMR (500 MHz, $CDCl_3$): δ 6.02 (dddd, *J* = 57.0, 55.9, 6.5, 3.0 Hz, 1H), 4.06 (tt, *J* = 8.5, 4.6 Hz, 1H), 2.42–2.28 (m, 2H), 1.95–1.87 (m, 1H), 1.76 (ddt, *J* = 14.8, 10.4, 5.3 Hz, 1H), 1.68–1.61 (m, 1H), 1.57–1.48 (m, 1H), 1.46–1.33 (m, 1H), 1.24 (s, 12H), 0.89–0.76 (m, 3H) ppm.
^{13}C NMR (126 MHz, $CDCl_3$): δ 118.06 (t, *J* = 239.7 Hz), 83.29, 83.09, 44.31 (t, *J* = 22.5 Hz), 43.14, 31.82, 29.85, 26.59 (dd, *J* = 8.5, 4.0 Hz), 24.97, 24.95, 23.71 ppm.
^{19}F NMR (470 MHz, $CDCl_3$): δ −116.66–−118.55 (m, 2F) ppm.
^{11}B NMR (160 MHz, $CDCl_3$): δ 34.06 ppm.
MS (DART) m/z: $[M+H]^+$ Calcd. for $C_{13}H_{25}BF_2IO_2^+$: 389.0955; Found: 389.0978.

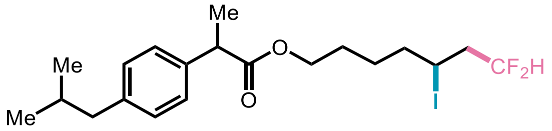

7,7-difluoro-5-iodoheptyl 2-(4-isobutylphenyl)propanoate (49)

Following **General Procedure D**, $TBAPF_6$ (309.8 mg, 0.80 mmol, 2.00 equiv.), $Me_3N·BH_3$ (145.9 mg, 2.00 mmol, 5.00 equiv.) and anhydrous degassed DMF (4.0 mL) were combined in an oven-dried 20 mL glass vial. Then, hex-5-en-1-yl 2-(4-isobutylphenyl)propanoate (115.3 mg, 0.40 mmol, 1.00 equiv.) and CF_2HI–Al–fum (134.8 mg, 0.80 mmol, 2.00 equiv.) were added sequentially. The vial was sealed, transferred out of the glovebox, and stirred at a constant cell current of 5 mA at 22 °C for 13 h. The crude product was purified by flash chromatography (SiO_2, gradient of 0% → 15% Et_2O in pentane) to afford compound **49** (108.1 mg, 58%) as a colorless oil.

^{1}H NMR (500 MHz, $CDCl_3$): δ 7.20 (d, J = 8.0 Hz, 2H), 7.09 (d, J = 8.1 Hz, 2H), 6.00 (dddd, J = 56.9, 55.8, 6.5, 2.8 Hz, 1H), 4.08 (dqt, J = 12.8, 4.7, 1.9 Hz, 2H), 3.99–3.94 (m, 1H), 3.69 (q, J = 7.1 Hz, 1H), 2.44 (d, J = 7.2 Hz, 2H), 2.40–2.18 (m, 2H), 1.89–1.77 (m, 2H), 1.71–1.56 (m, 3H), 1.49 (d, J = 7.2 Hz, 4H), 1.41–1.31 (m, 1H), 0.90 (d, J = 6.6 Hz, 6H) ppm.
^{13}C NMR (126 MHz, $CDCl_3$): δ 174.87, 140.69, 137.91, 137.90, 129.47, 129.44, 127.28, 117.93 (t, J = 239.9 Hz), 64.20, 45.31, 45.14, 44.49–44.12, 40.10 (m), 40.08, 30.32, 29.84, 27.70, 27.68, 26.32 (dt, J = 8.7, 3.3 Hz), 25.74, 22.53, 22.50 ppm.
^{19}F NMR (470 MHz, $CDCl_3$): δ −116.58–−118.59 (m, 2F) ppm.
MS (DART) m/z: $[M+H]^+$ Calcd. for $C_{20}H_{30}F_2IO_2^+$: 467.1253; Found: 467.1219.

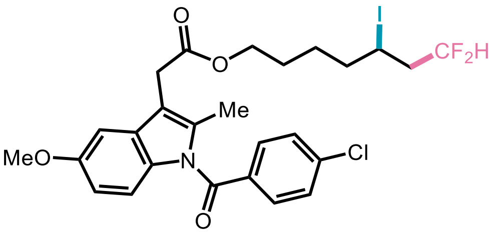

7,7-difluoro-5-iodoheptyl 2-(1-(4-chlorobenzoyl)-5-methoxy-2-methyl-1H-indol-3-yl)acetate (50)

Following **General Procedure D**, $TBAPF_6$ (309.8 mg, 0.80 mmol, 2.00 equiv.), $Me_3N·BH_3$ (145.9 mg, 2.00 mmol, 5.00 equiv.) and anhydrous degassed DMF (4.0 mL) were combined in an oven-dried 20 mL glass vial. Then, hex-5-en-1-yl 2-(1-(4-chlorobenzoyl)-5-methoxy-2-methyl-1H-indol-3-yl)acetate (175.7 mg, 0.40 mmol, 1.00 equiv.) and CF_2HI–Al–fum (134.8 mg, 0.80 mmol, 2.00 equiv.) were added sequentially. The vial was sealed, transferred out of the glovebox, and stirred at a constant cell current of 5 mA at 22 °C for 13 h. The crude product was purified by flash

chromatography (SiO_2, gradient of 10% → 50% Et_2O in pentane) to afford compound **50** (116.0 mg, 47%) as a colorless oil.

^{1}H NMR (500 MHz, $CDCl_3$): δ 7.66 (d, *J* = 8.5 Hz, 2H), 7.47 (d, *J* = 8.5 Hz, 2H), 6.97 (d, *J* = 2.5 Hz, 1H), 6.85 (d, *J* = 9.0 Hz, 1H), 6.67 (dd, *J* = 9.0, 2.5 Hz, 1H), 5.99 (dddd, *J* = 57.0, 55.8, 6.5, 2.9 Hz, 1H), 4.12 (t, *J* = 6.3 Hz, 2H), 3.95 (td, *J* = 8.7, 3.7 Hz, 1H), 3.83 (s, 3H), 3.67 (s, 2H), 2.40 (s, 3H), 2.35–2.15 (m, 2H), 1.86–1.78 (m, 1H), 1.66 (tdd, *J* = 15.9, 9.7, 5.5 Hz, 3H), 1.55 (dtd, *J* = 18.1, 9.7, 5.0 Hz, 1H), 1.43–1.35 (m, 1H) ppm.
^{13}C NMR (126 MHz, $CDCl_3$): δ 170.99, 168.41, 156.15, 139.41, 136.11, 134.00, 131.31, 131.30, 130.94, 130.77, 129.26, 117.89 (t, *J* = 239.9 Hz), 115.10, 112.70, 111.61, 101.60, 64.64, 55.86, 44.26 (t, *J* = 22.6 Hz), 40.07, 30.57, 27.78, 26.24 (dd, *J* = 8.5, 3.9 Hz), 25.86, 13.52 ppm.
^{19}F NMR (470 MHz, $CDCl_3$): δ −116.49–−118.55 (m, 2F) ppm.
MS (DART) m/z: $[M+H]^+$ Calcd. for $C_{26}H_{28}ClF_2INO_4^+$: 618.0714; Found: 618.0689.

19. Copies of NMR Spectra.

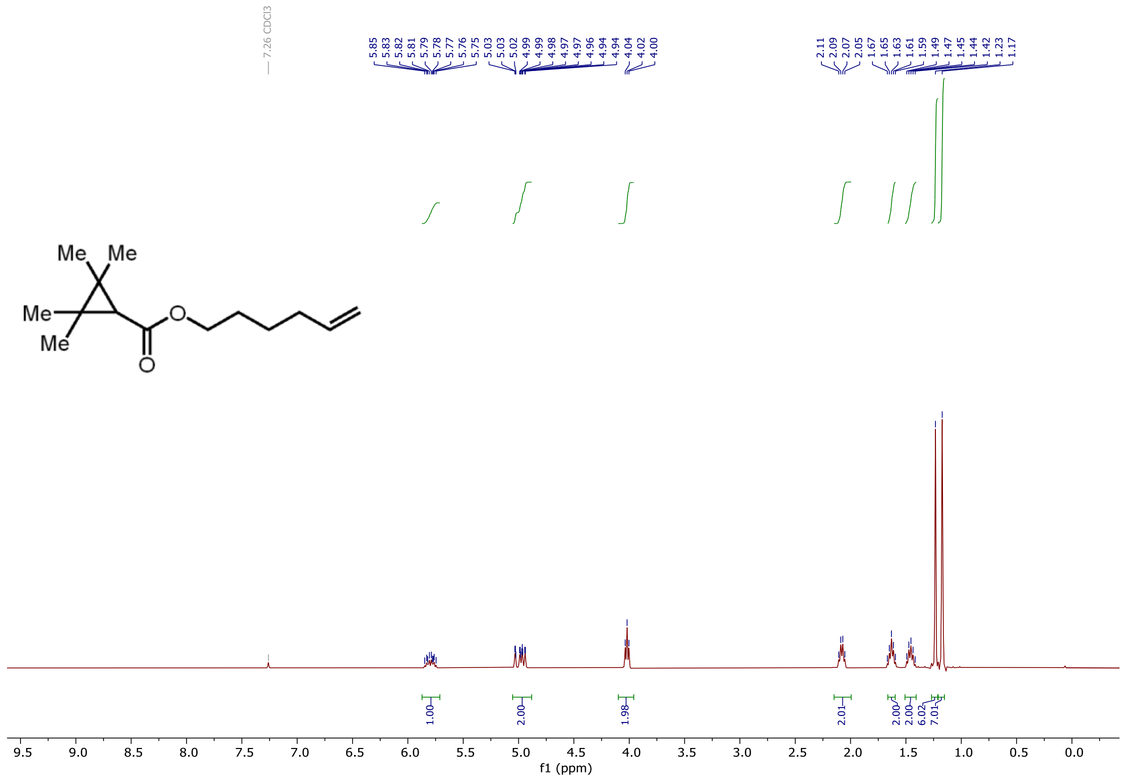

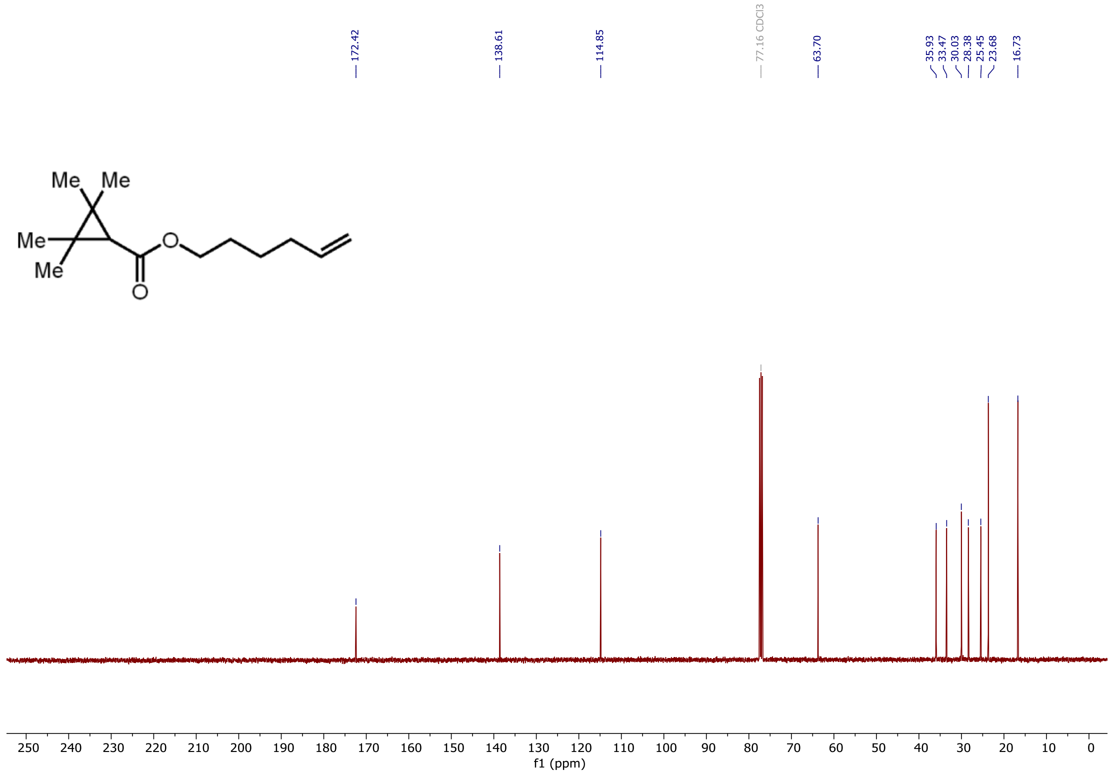

Figure 59. ^{1}H NMR (500 MHz, $CDCl_3$) and ^{13}C NMR (101 MHz, $CDCl_3$) spectra of **S1**.

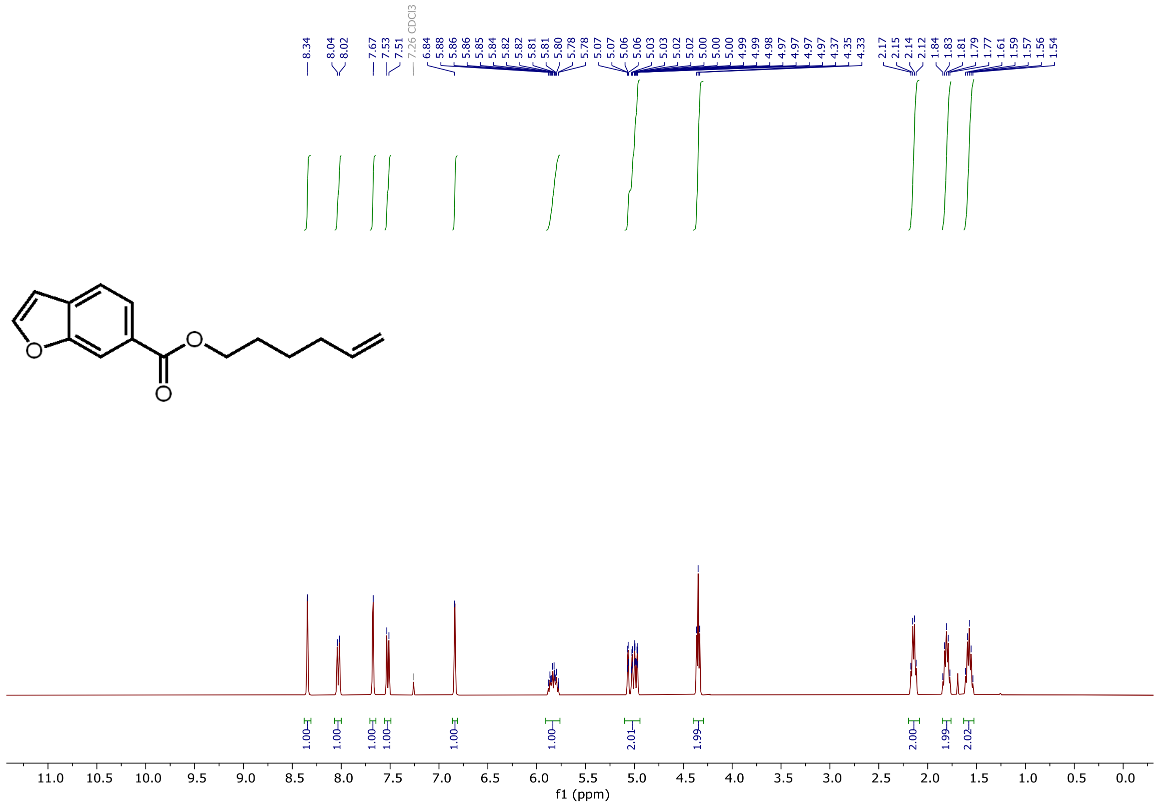

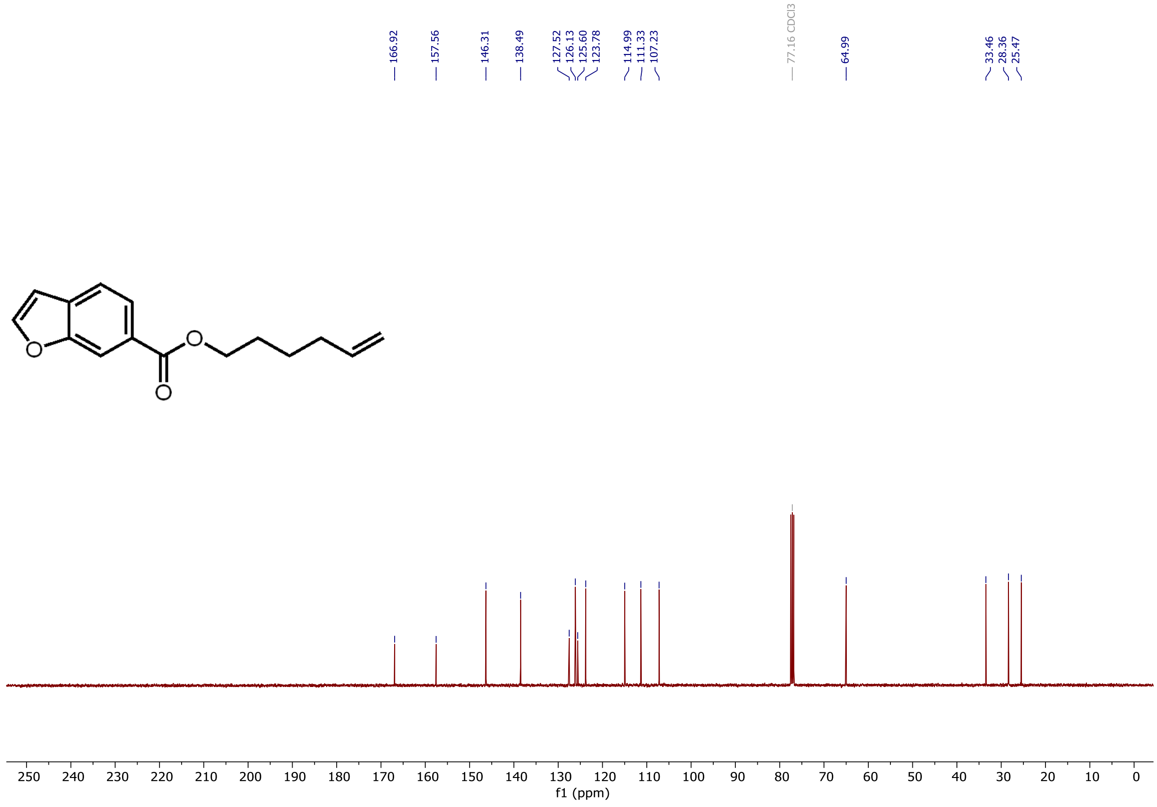

Figure S60. ^{1}H NMR (500 MHz, $CDCl_3$) and ^{13}C NMR (101 MHz, $CDCl_3$) spectra of **S2**.

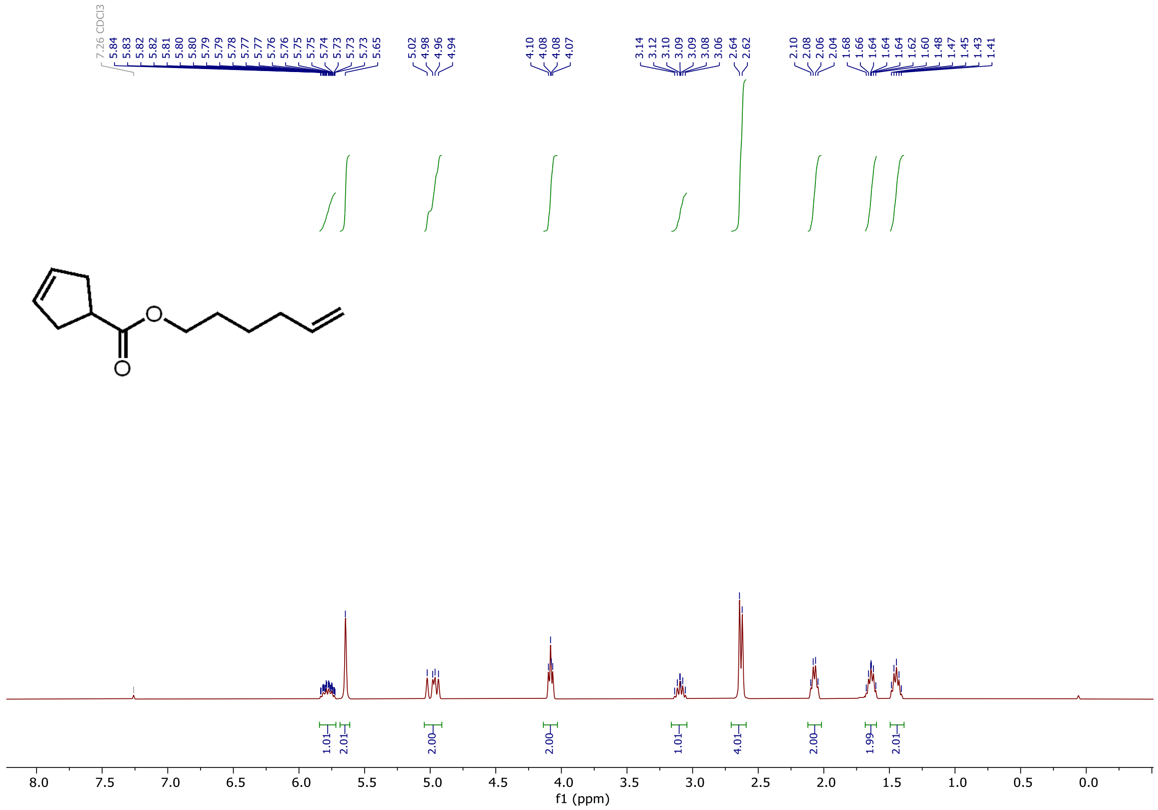

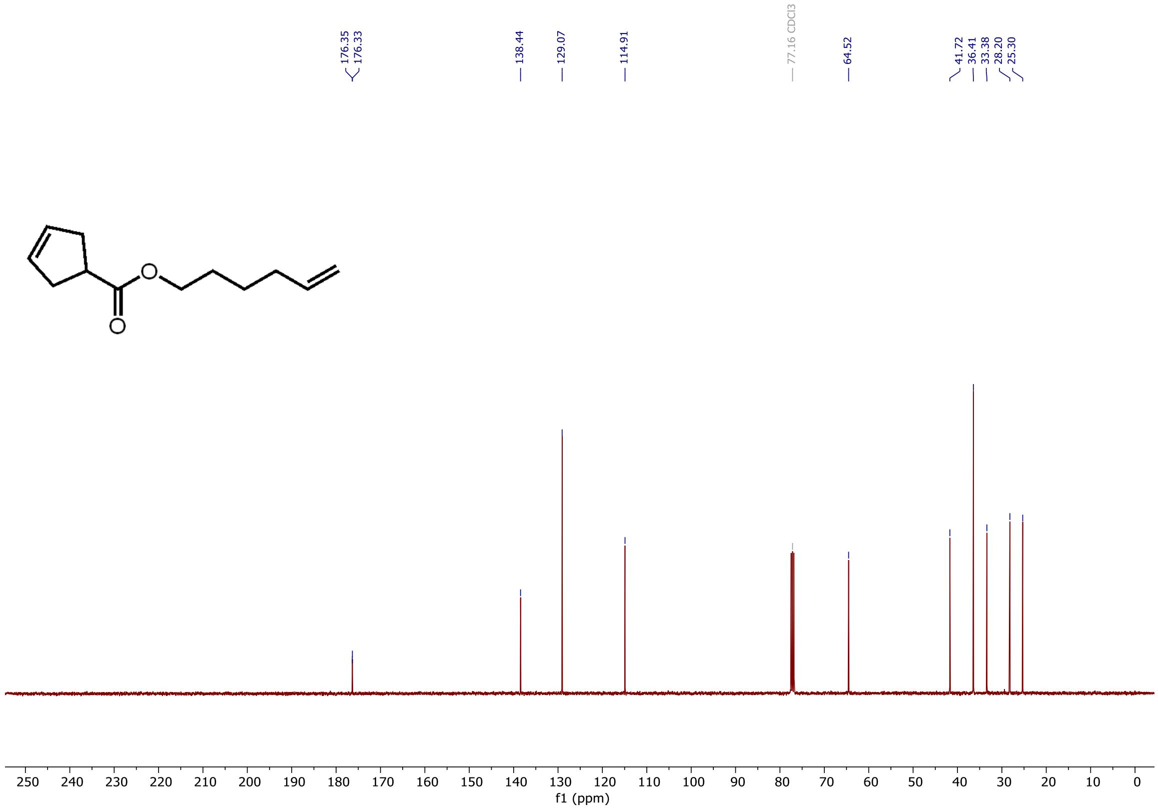

Figure S61. ^{1}H NMR (500 MHz, $CDCl_3$) and ^{13}C NMR (101 MHz, $CDCl_3$) spectra of **S3**.

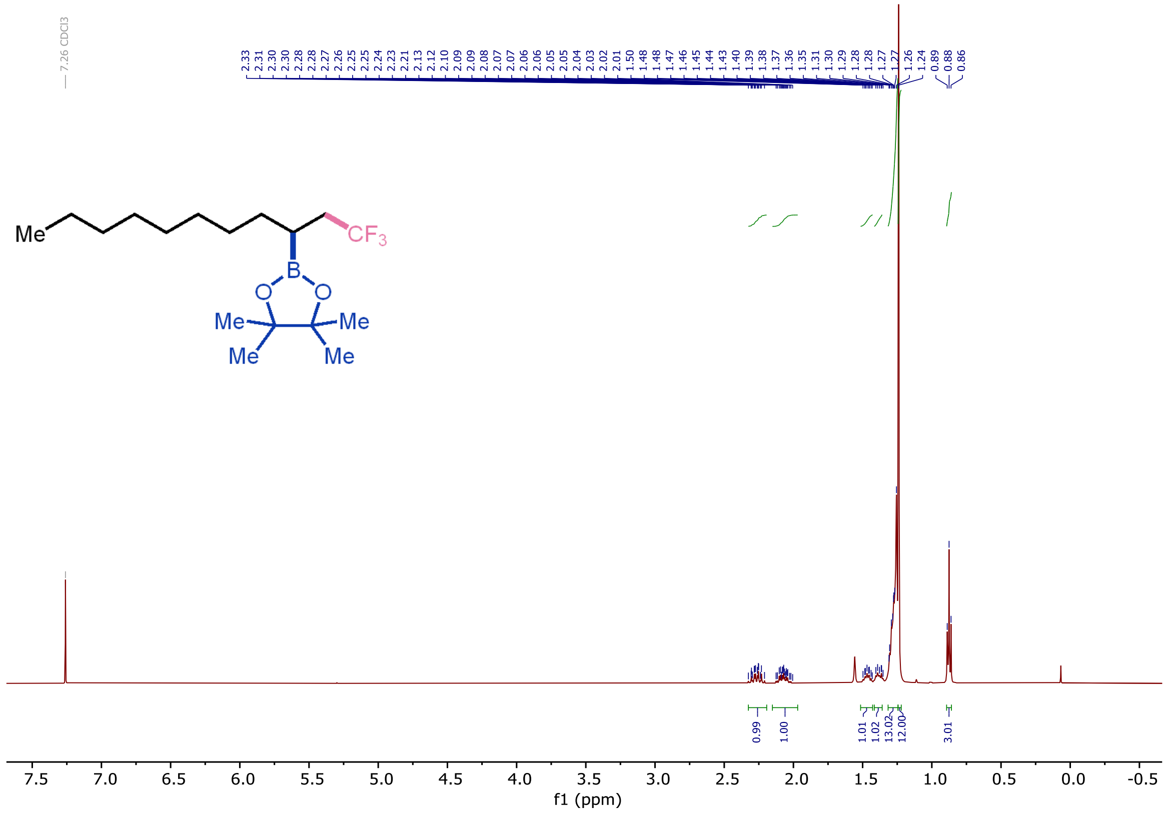
7.26 CDCl3
2.33 2.31 2.30 2.30 2.28 2.28 2.27 2.26 2.25 2.25 2.24 2.23 2.21 2.13 2.12 2.10 2.09 2.09 2.08 2.07 2.07 2.06 2.06 2.05 2.05 2.04 2.03 2.02 2.01 1.50 1.48 1.48 1.47 1.46 1.45 1.44 1.43 1.40 1.39 1.38 1.37 1.36 1.35 1.31 1.30 1.29 1.28 1.28 1.27 1.27 1.26 1.24 0.89 0.88 0.86
Me
CF3
B
O
O
Me
Me
Me
Me
0.99
1.00
1.01
1.02
13.02
12.00
3.01
7.5 7.0 6.5 6.0 5.5 5.0 4.5 4.0 3.5 3.0 2.5 2.0 1.5 1.0 0.5 0.0 -0.5
f1 (ppm)

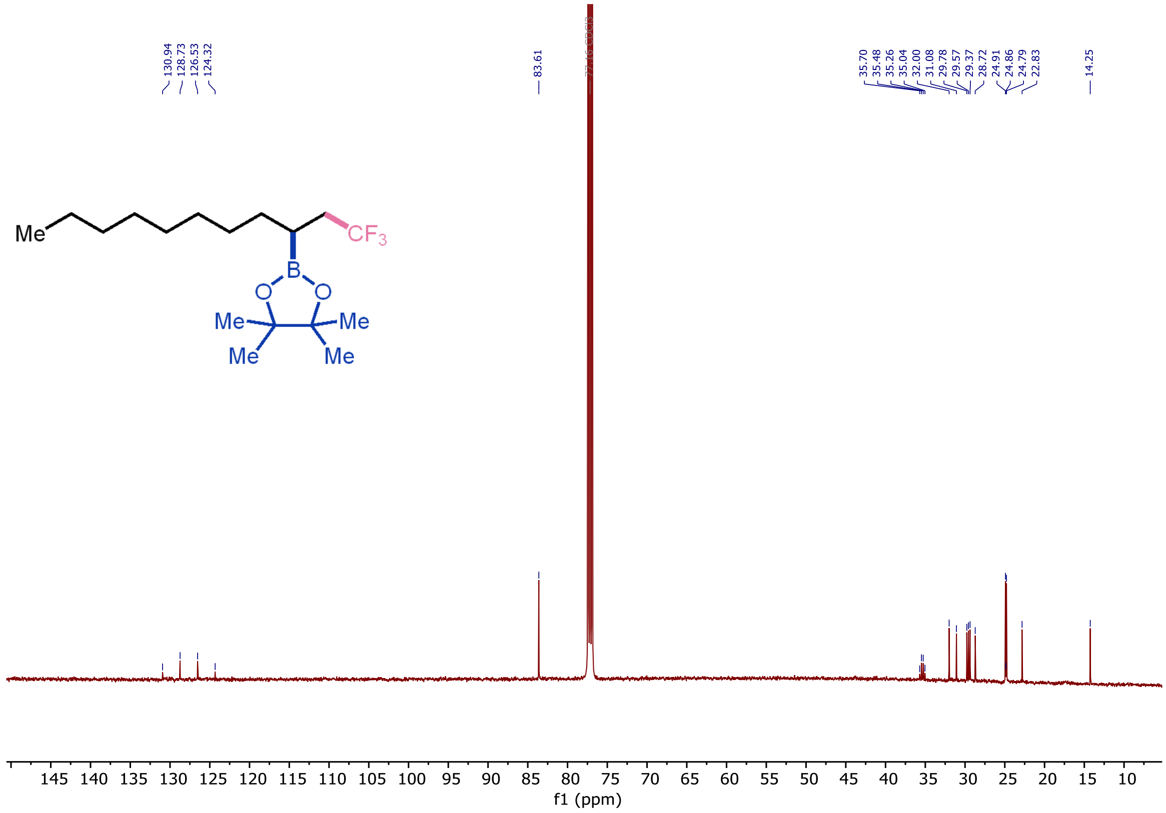
130.94 128.73 126.53 124.32
83.61
35.70 35.48 35.26 35.04 32.00 31.08 29.78 29.57 29.37 28.72 24.91 24.86 24.79 22.83
14.25
Me
CF3
B
O
O
Me
Me
Me
Me
145 140 135 130 125 120 115 110 105 100 95 90 85 80 75 70 65 60 55 50 45 40 35 30 25 20 15 10
f1 (ppm)

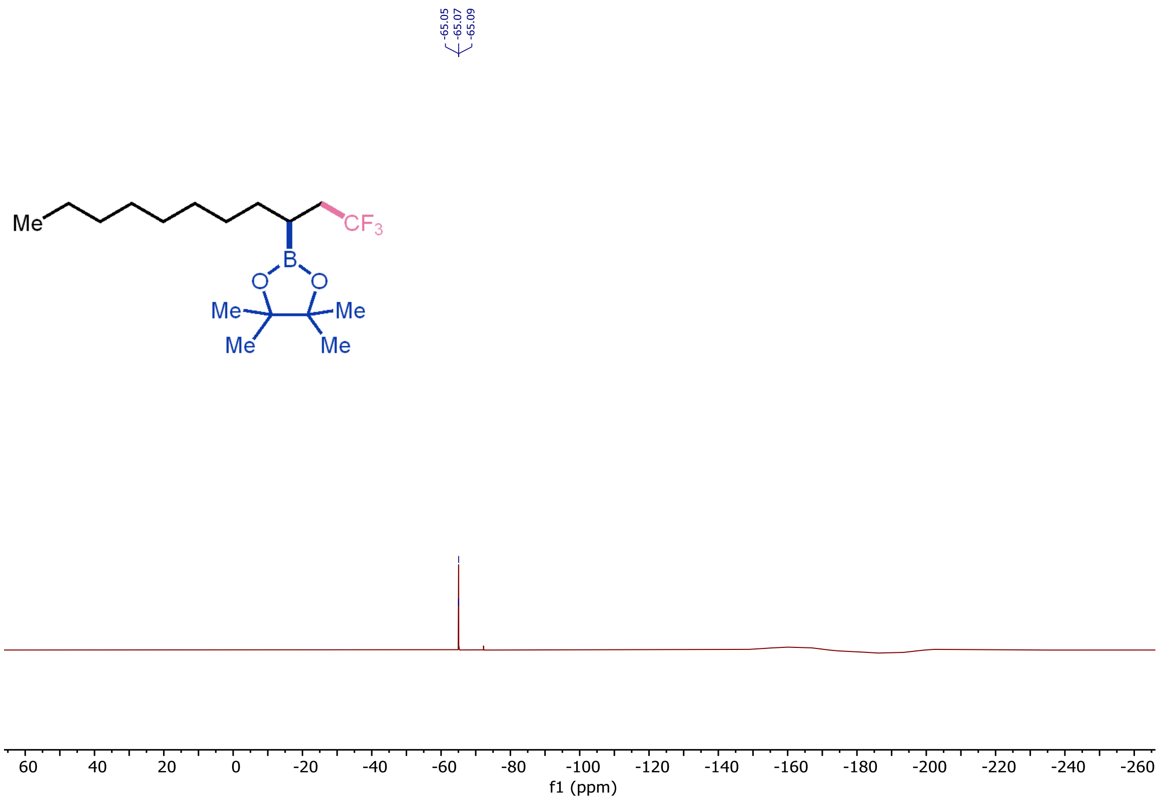

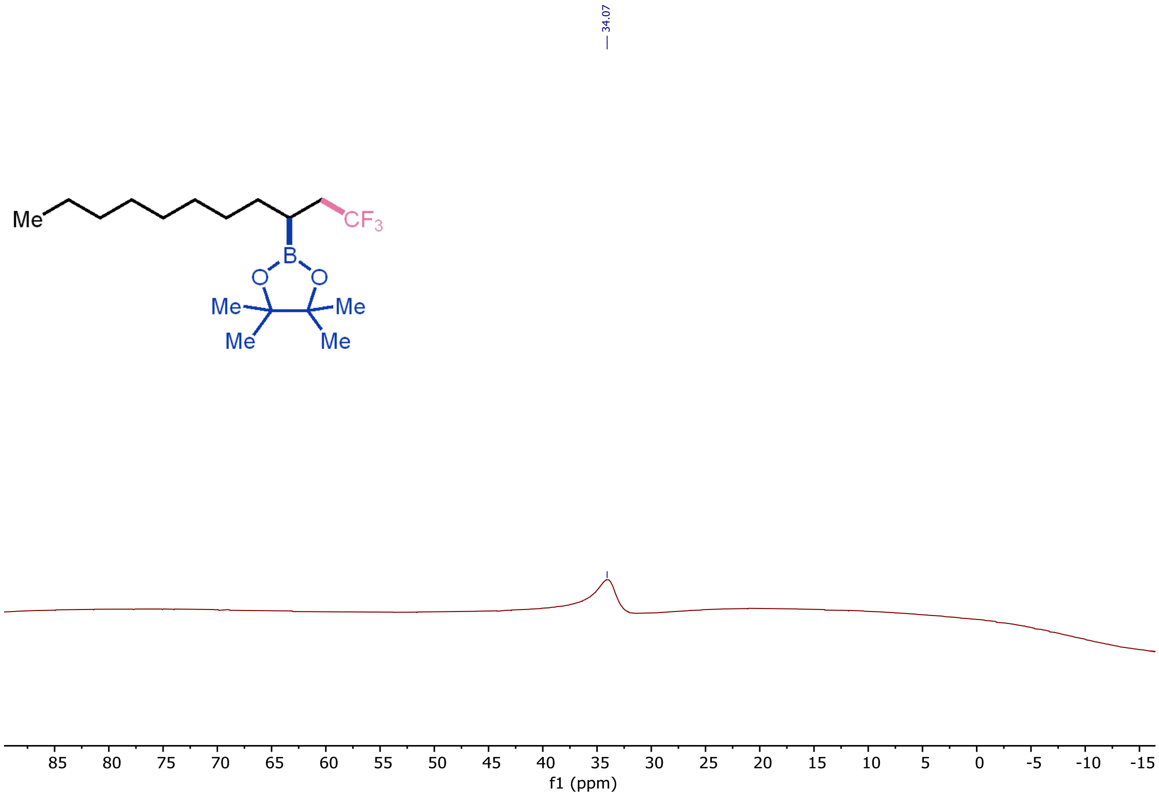

Figure S62. ^{1}H NMR (500 MHz, $CDCl_3$), ^{13}C NMR (101 MHz, $CDCl_3$), ^{19}F NMR (376 MHz, $CDCl_3$) and ^{11}B NMR (160 MHz, $CDCl_3$) spectra of **6**.

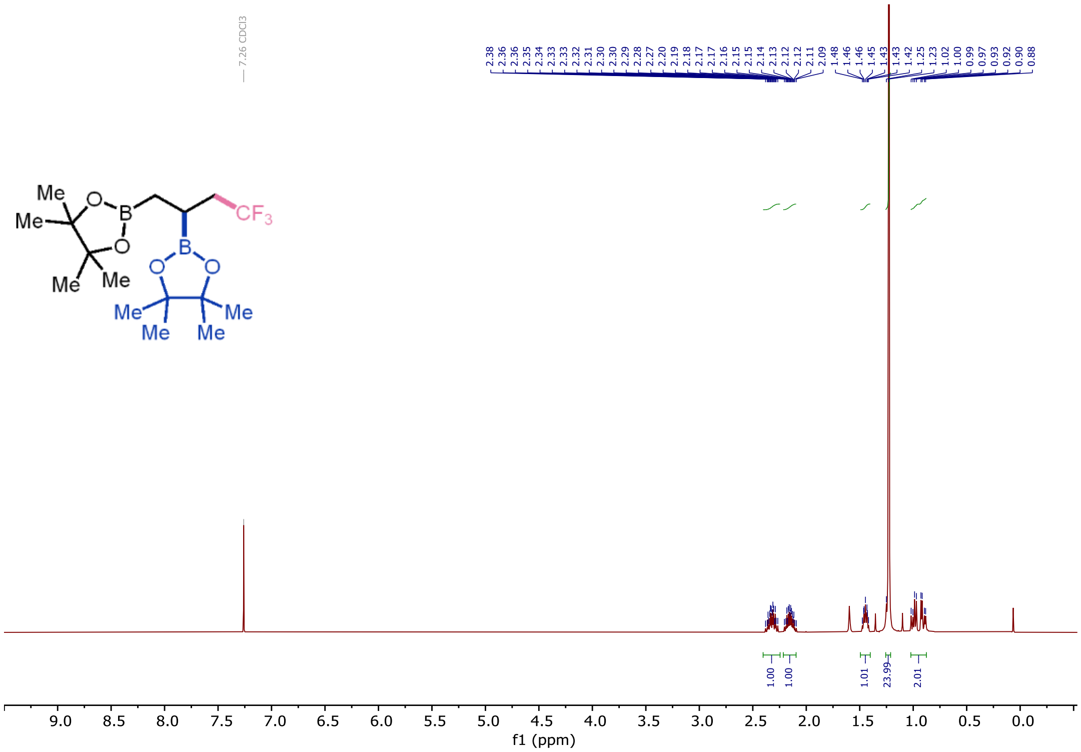
7.26 CDCl3
2.38 2.36 2.36 2.35 2.34 2.33 2.33 2.32 2.31 2.30 2.30 2.29 2.28 2.27 2.20 2.19 2.18 2.17 2.17 2.16 2.15 2.15 2.14 2.13 2.12 2.12 2.11 2.09 1.48 1.46 1.46 1.45 1.43 1.43 1.42 1.25 1.23 1.02 1.00 1.00 0.99 0.97 0.93 0.92 0.90 0.88
Me
Me
Me
Me
O
B
O
CF3
B
O
O
Me
Me
Me
Me
1.00
1.00
1.01
23.99
2.01
9.0 8.5 8.0 7.5 7.0 6.5 6.0 5.5 5.0 4.5 4.0 3.5 3.0 2.5 2.0 1.5 1.0 0.5 0.0
f1 (ppm)

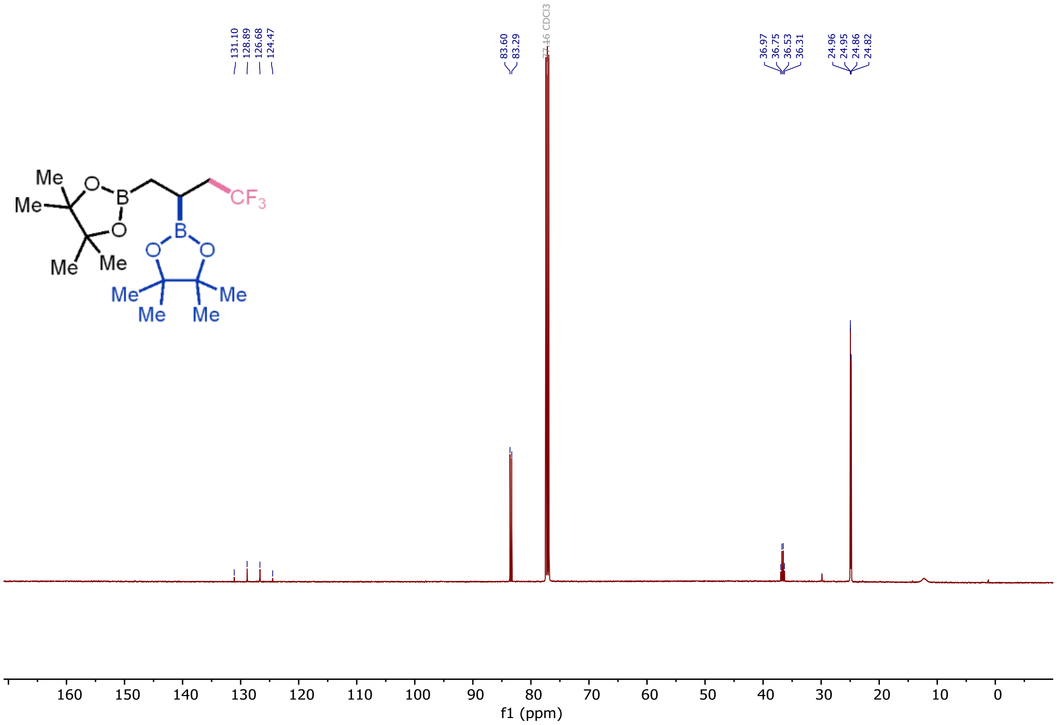
131.10
128.89
126.68
124.47
83.60
83.29
77.16 CDCl3
36.97
36.75
36.53
36.31
24.96
24.95
24.86
24.82
Me
Me
Me
Me
O
B
O
CF3
B
O
O
Me
Me
Me
Me
160 150 140 130 120 110 100 90 80 70 60 50 40 30 20 10 0
f1 (ppm)

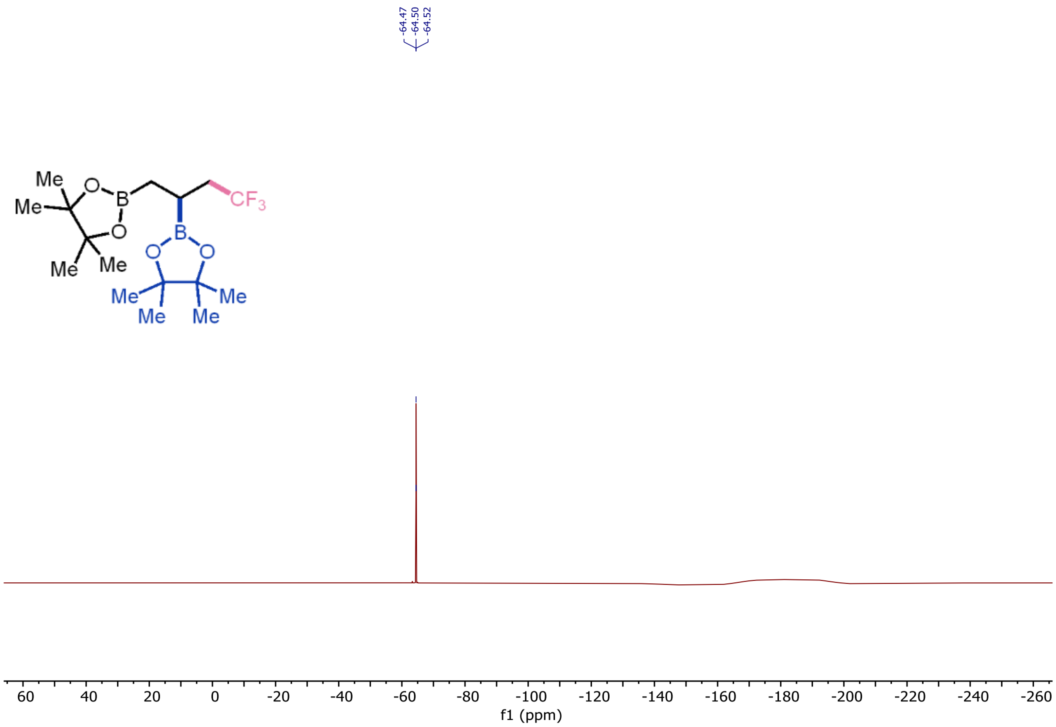

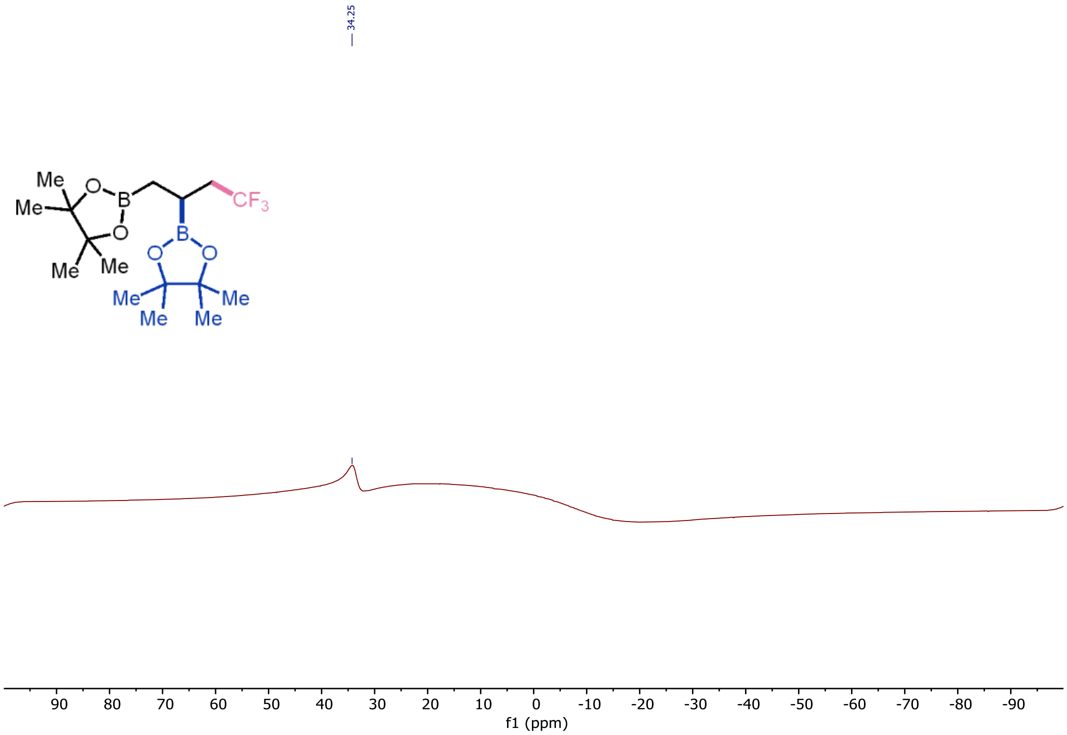

Figure S63. ^{1}H NMR (500 MHz, $CDCl_3$), ^{13}C NMR (101 MHz, $CDCl_3$), ^{19}F NMR (376 MHz, $CDCl_3$) and ^{11}B NMR (160 MHz, $CDCl_3$) spectra of **7**.

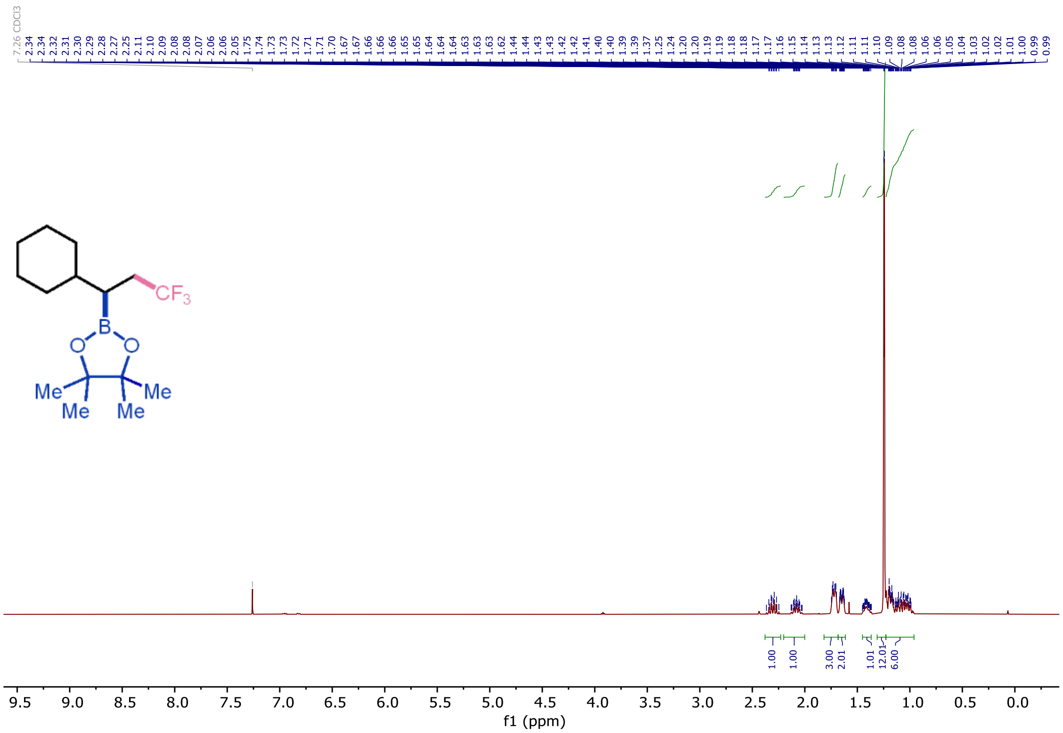
7.26 CDCl3
2.34 2.34 2.32 2.31 2.30 2.29 2.28 2.27 2.25 2.11 2.10 2.09 2.08 2.08 2.07 2.06 2.06 2.05 1.75 1.74 1.73 1.73 1.72 1.71 1.71 1.70 1.67 1.66 1.66 1.66 1.65 1.64 1.64 1.64 1.63 1.63 1.63 1.62 1.44 1.43 1.43 1.42 1.41 1.40 1.40 1.39 1.39 1.37 1.25 1.24 1.20 1.20 1.19 1.19 1.18 1.18 1.17 1.17 1.16 1.15 1.14 1.13 1.13 1.12 1.11 1.11 1.10 1.09 1.08 1.08 1.06 1.06 1.05 1.04 1.03 1.02 1.01 1.00 0.99 0.99
CF3
B
O
O
Me
Me
Me
Me
1.00
1.00
3.00
2.01
1.01
12.01
6.00
9.5 9.0 8.5 8.0 7.5 7.0 6.5 6.0 5.5 5.0 4.5 4.0 3.5 3.0 2.5 2.0 1.5 1.0 0.5 0.0
f1 (ppm)

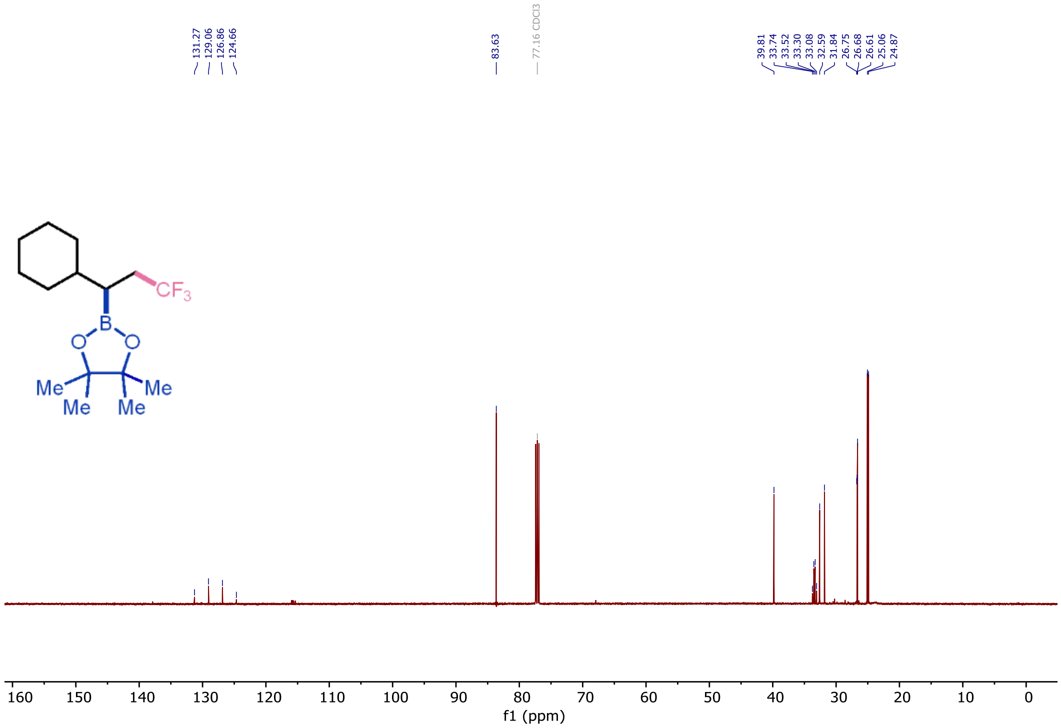
131.27 129.06 126.86 124.66
83.63
77.16 CDCl3
39.81 33.74 33.52 33.30 33.08 32.59 31.84 26.75 26.68 26.61 25.06 24.87
CF3
B
O
O
Me
Me
Me
Me
160 150 140 130 120 110 100 90 80 70 60 50 40 30 20 10 0
f1 (ppm)

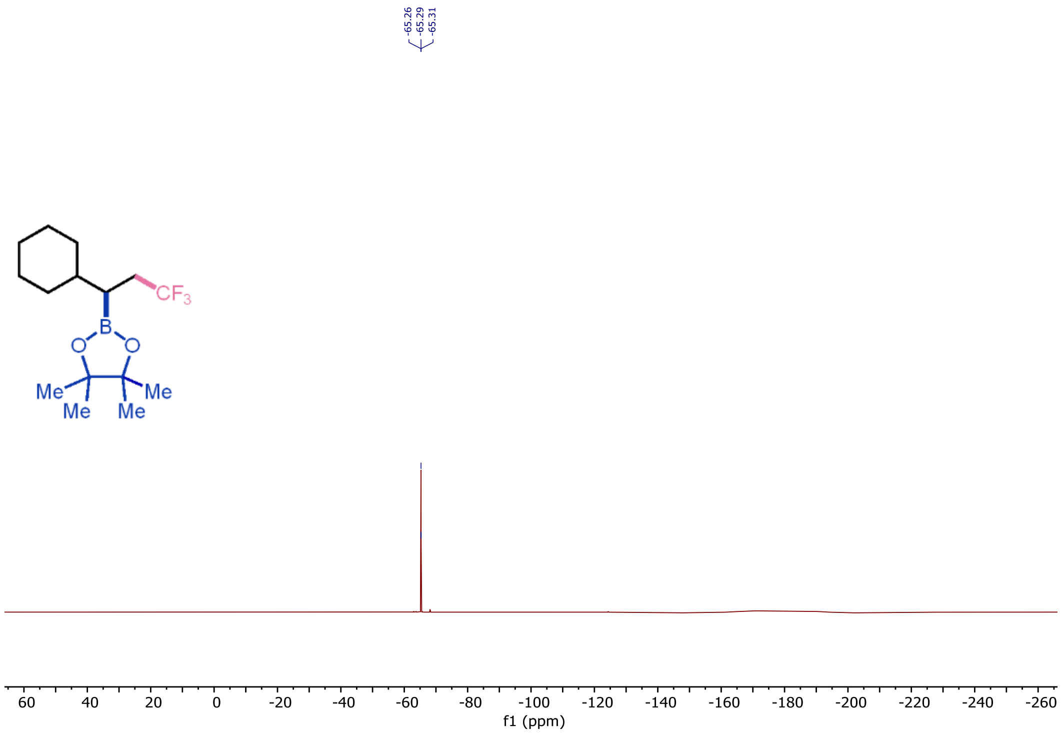

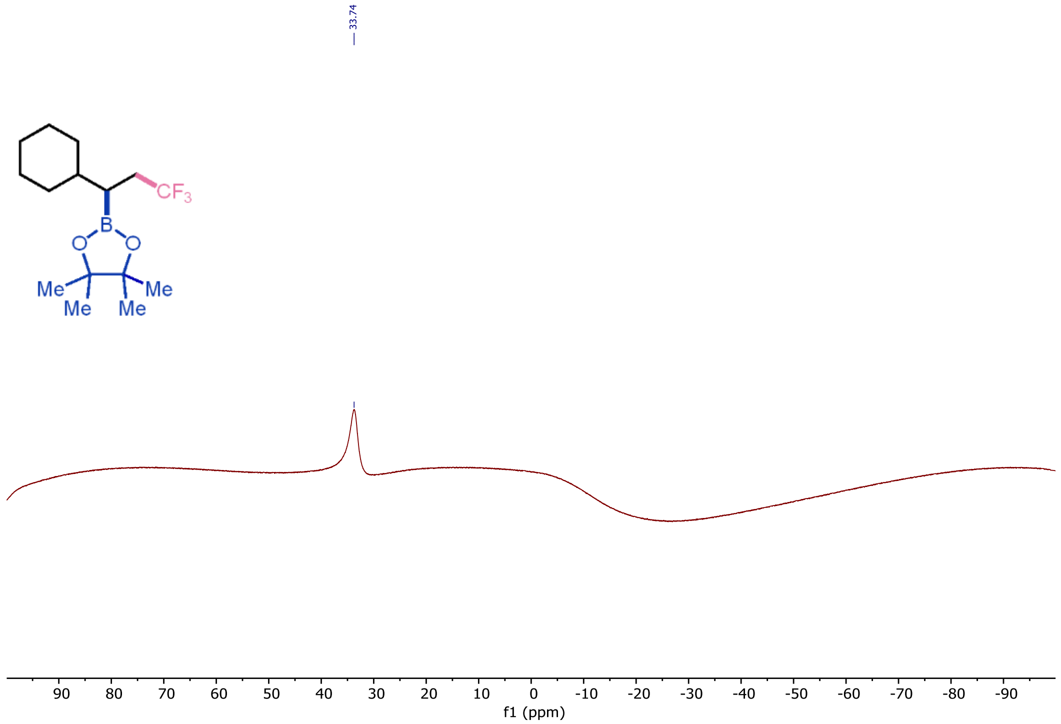

Figure S64. ^{1}H NMR (500 MHz, $CDCl_3$), ^{13}C NMR (101 MHz, $CDCl_3$), ^{19}F NMR (376 MHz, $CDCl_3$) and ^{11}B NMR (160 MHz, $CDCl_3$) spectra of **8**.

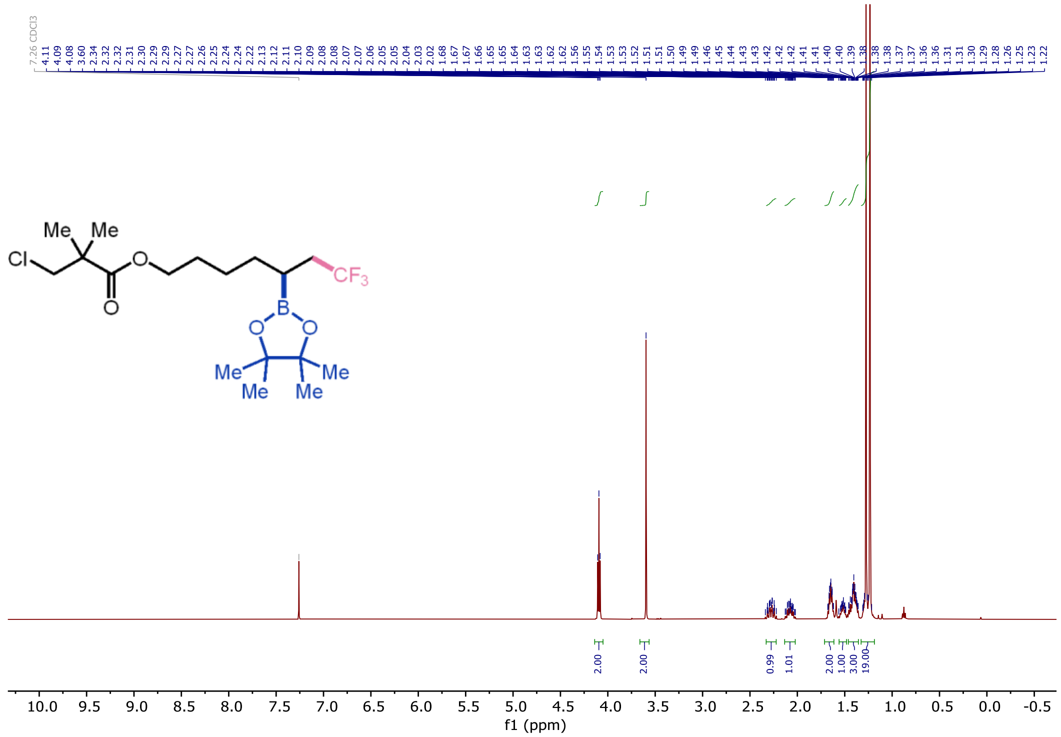

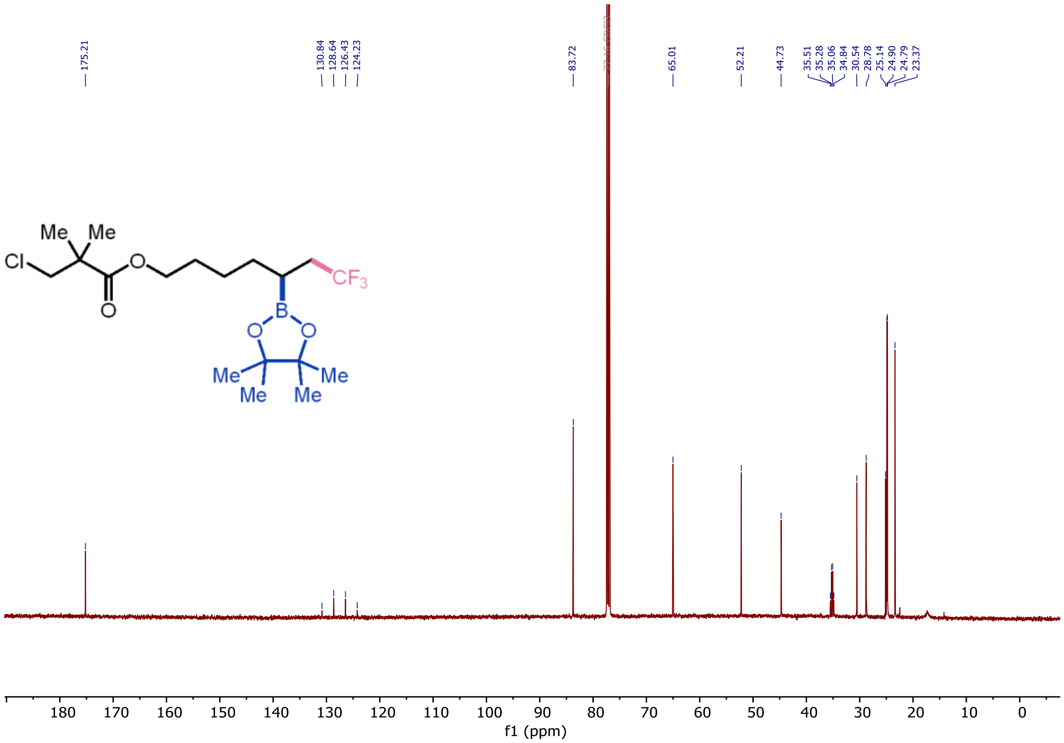

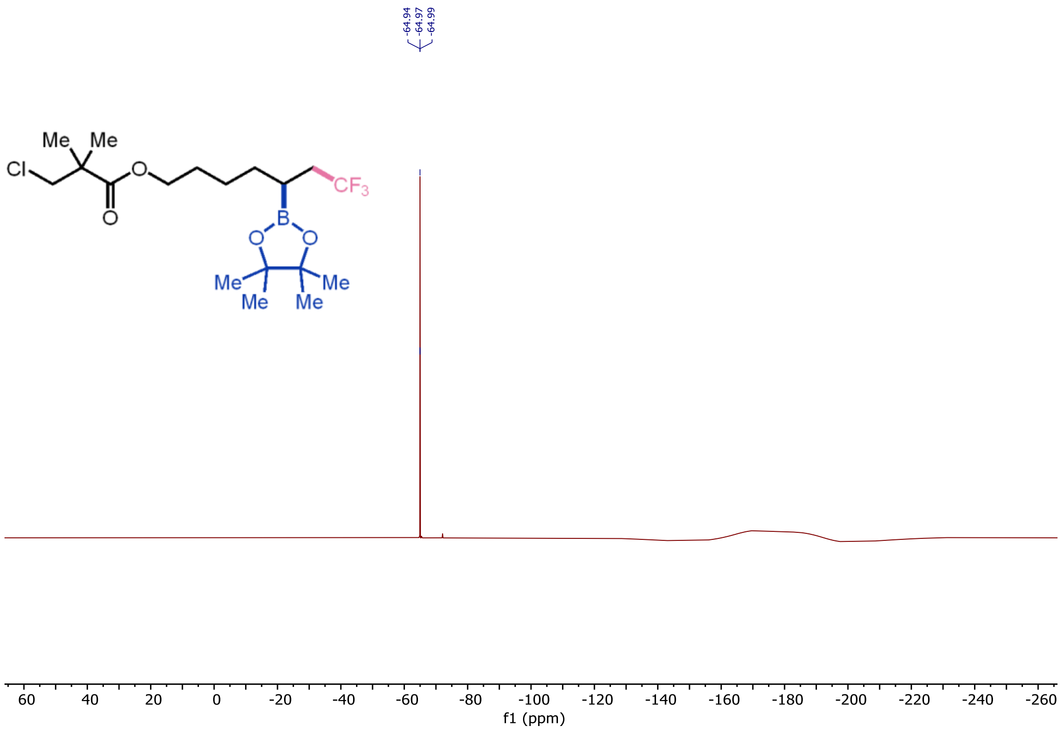

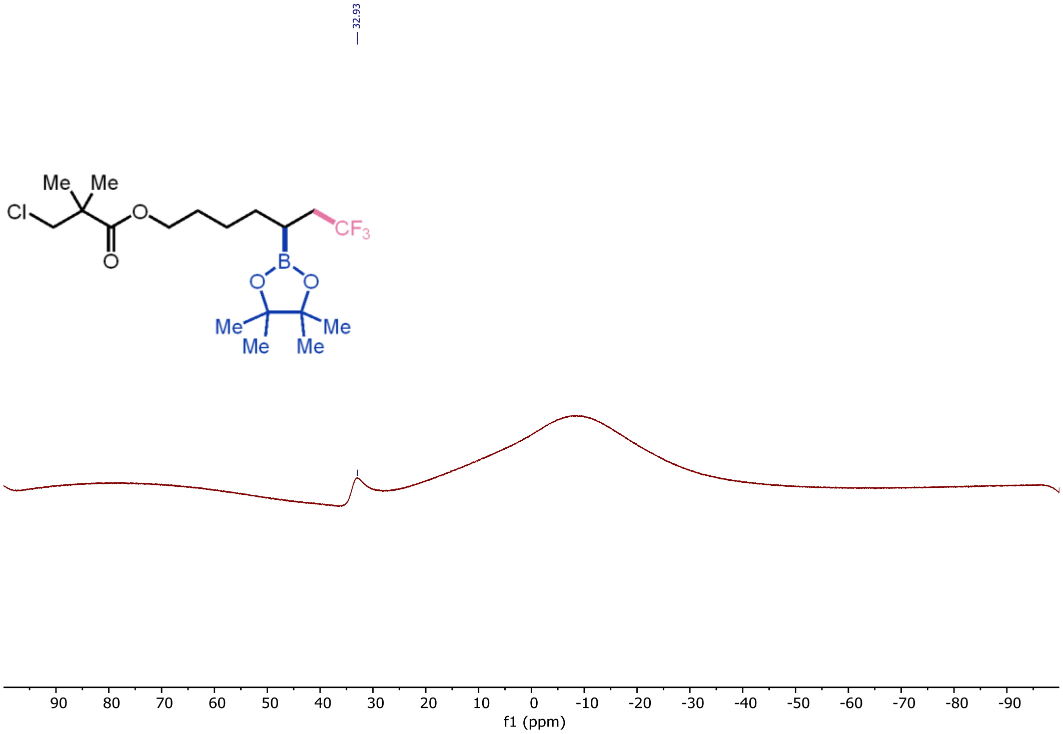

Figure S65. ^{1}H NMR (500 MHz, $CDCl_3$), ^{13}C NMR (101 MHz, $CDCl_3$), ^{19}F NMR (376 MHz, $CDCl_3$) and ^{11}B NMR (160 MHz, $CDCl_3$) spectra of **9**.

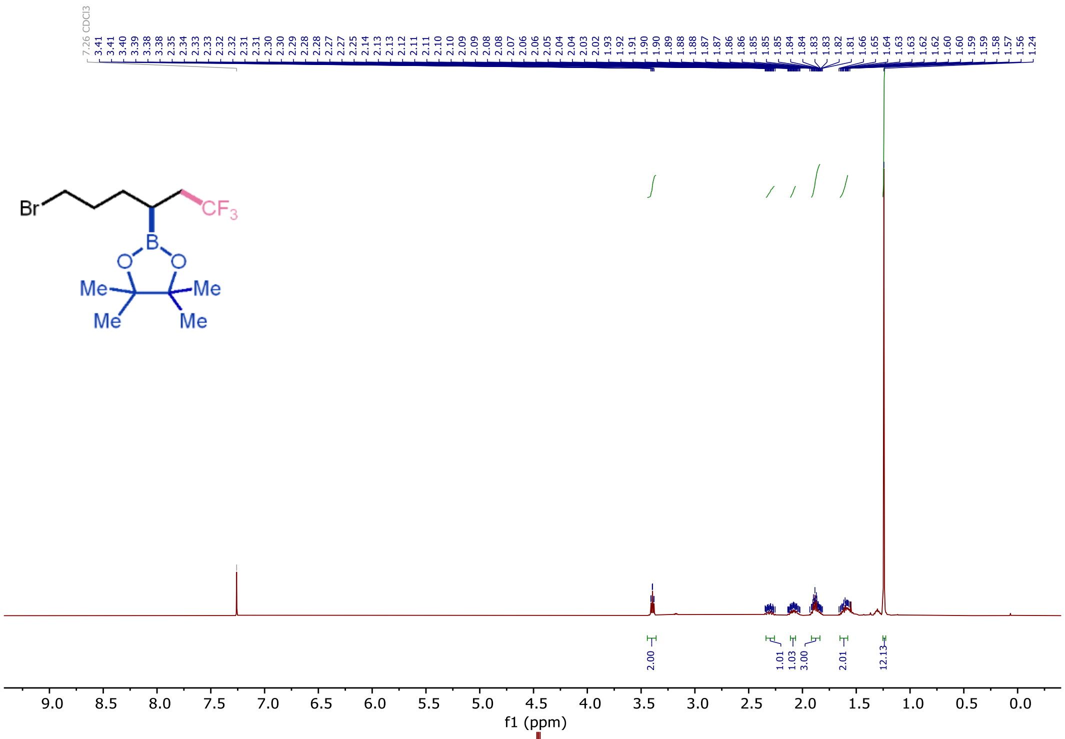

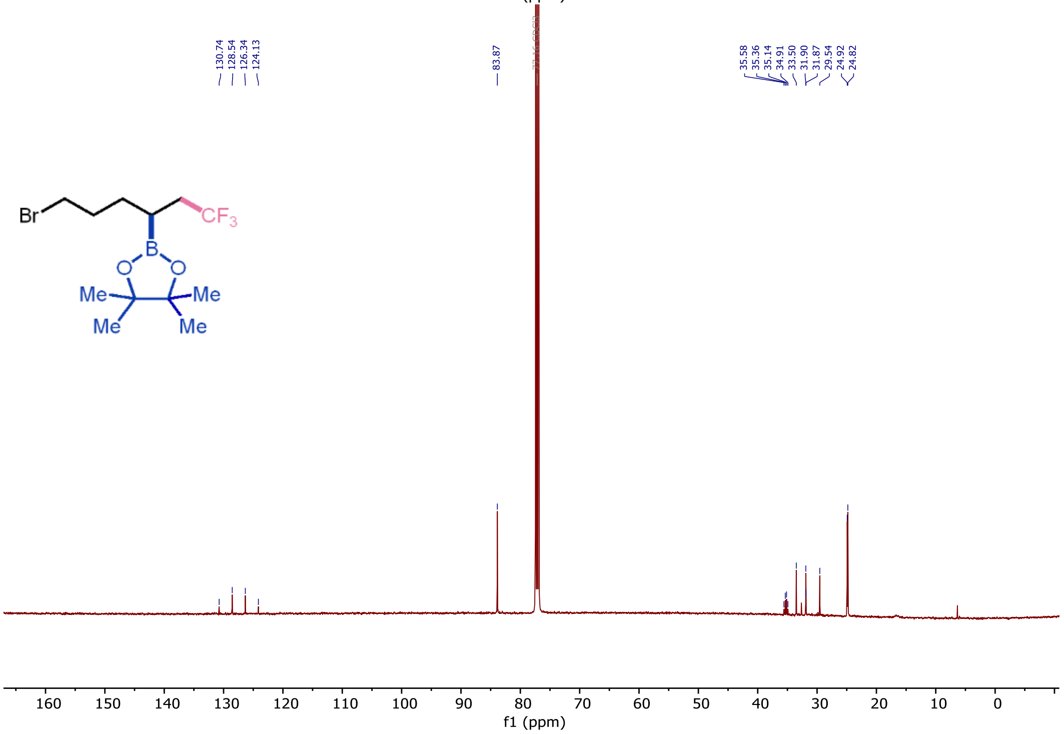

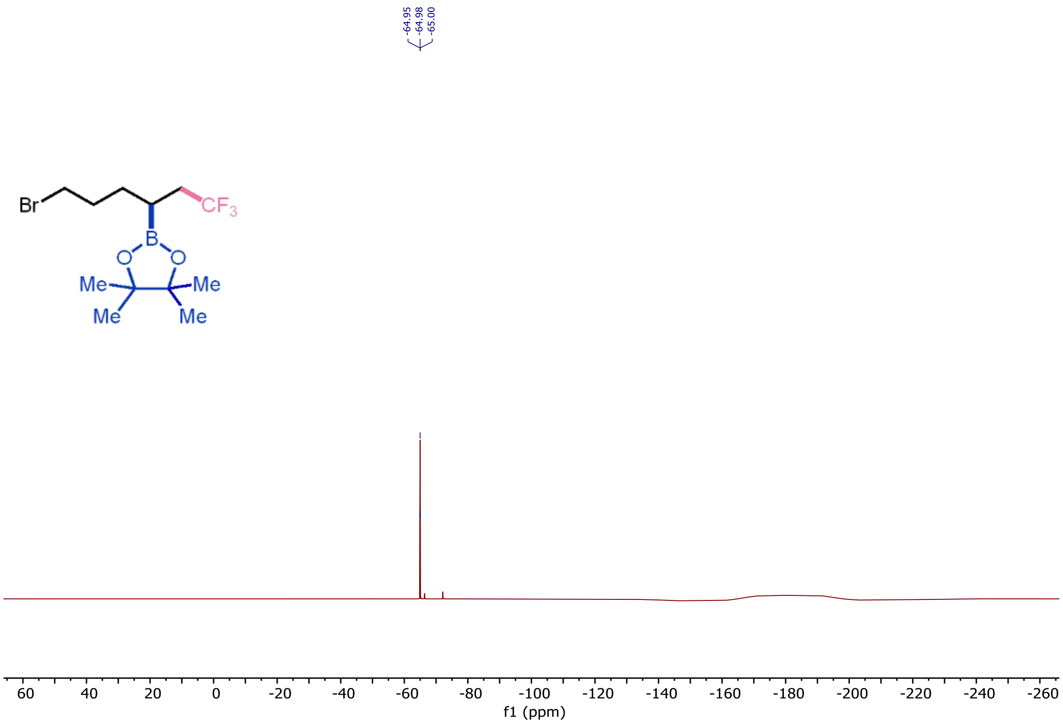

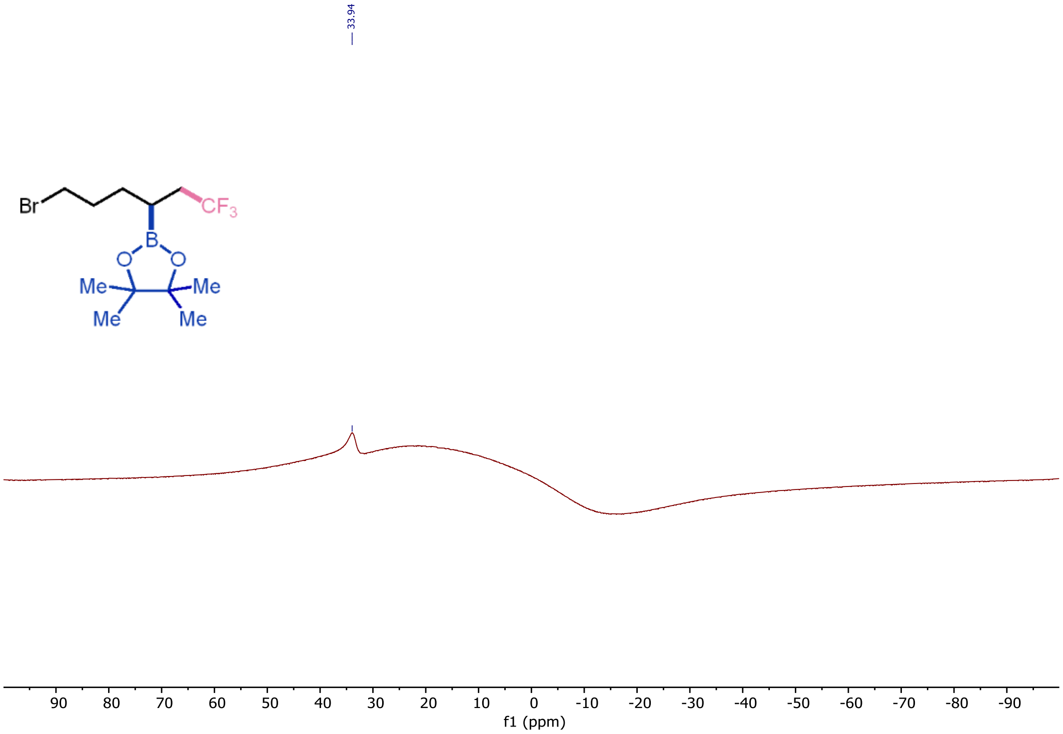

Figure S66. ^{1}H NMR (500 MHz, $CDCl_3$), ^{13}C NMR (101 MHz, $CDCl_3$), ^{19}F NMR (376 MHz, $CDCl_3$) and ^{11}B NMR (160 MHz, $CDCl_3$) spectra of **10**.

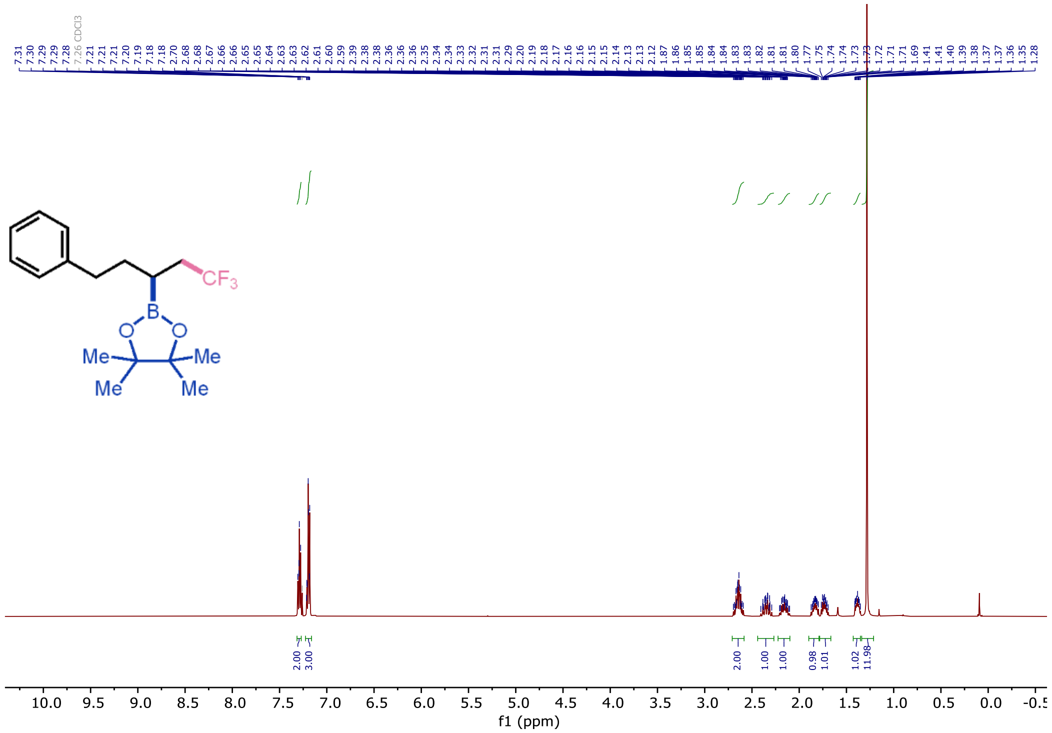
7.31 7.30 7.29 7.29 7.28 7.26 CDCl3 7.21 7.21 7.21 7.20 7.19 7.18 2.70 2.68 2.68 2.67 2.66 2.66 2.65 2.65 2.64 2.63 2.63 2.62 2.61 2.60 2.59 2.39 2.38 2.38 2.36 2.36 2.36 2.35 2.34 2.34 2.33 2.32 2.31 2.31 2.29 2.20 2.19 2.18 2.17 2.16 2.16 2.15 2.15 2.14 2.13 2.13 2.12 1.87 1.86 1.85 1.85 1.84 1.84 1.83 1.83 1.82 1.81 1.81 1.80 1.77 1.75 1.74 1.74 1.73 1.73 1.72 1.71 1.71 1.69 1.41 1.41 1.40 1.39 1.38 1.37 1.37 1.36 1.35 1.28
CF3
B
O
O
Me
Me
Me
Me
2.00
3.00
2.00
1.00
1.00
0.98
1.01
1.02
11.98
10.0 9.5 9.0 8.5 8.0 7.5 7.0 6.5 6.0 5.5 5.0 4.5 4.0 3.5 3.0 2.5 2.0 1.5 1.0 0.5 0.0 -0.5
f1 (ppm)

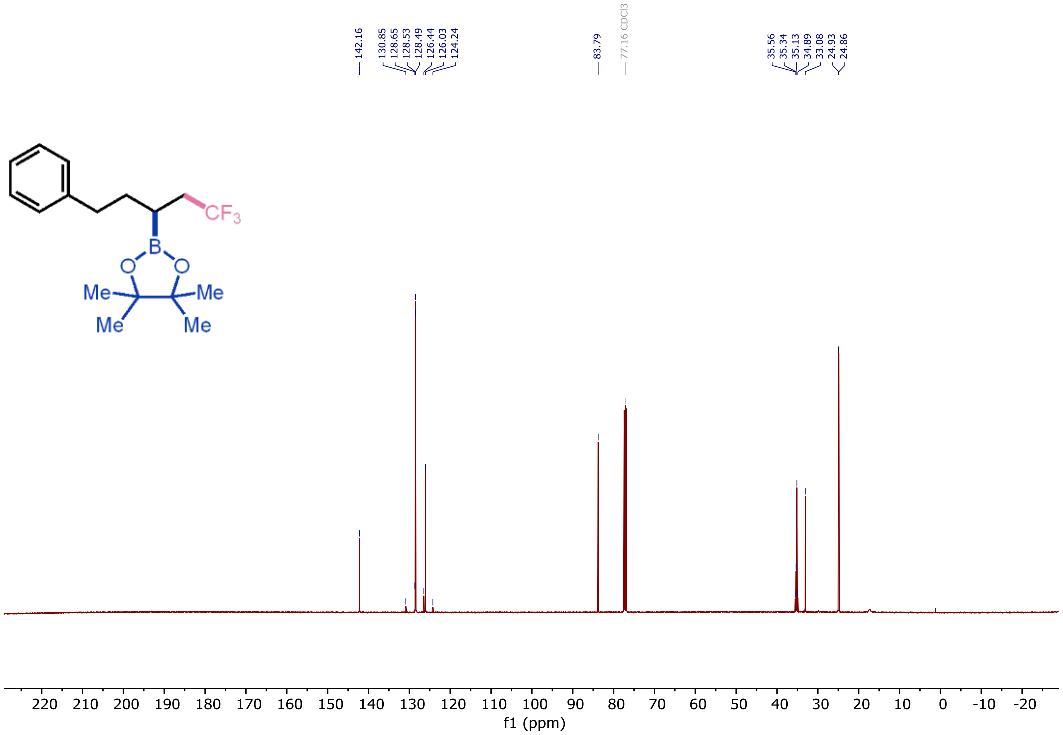
142.16
130.85 128.65 128.53 128.49 126.44 126.03 124.24
83.79
77.16 CDCl3
35.56 35.34 35.13 34.89 33.08 24.93 24.86
CF3
B
O
O
Me
Me
Me
Me
220 210 200 190 180 170 160 150 140 130 120 110 100 90 80 70 60 50 40 30 20 10 0 -10 -20
f1 (ppm)

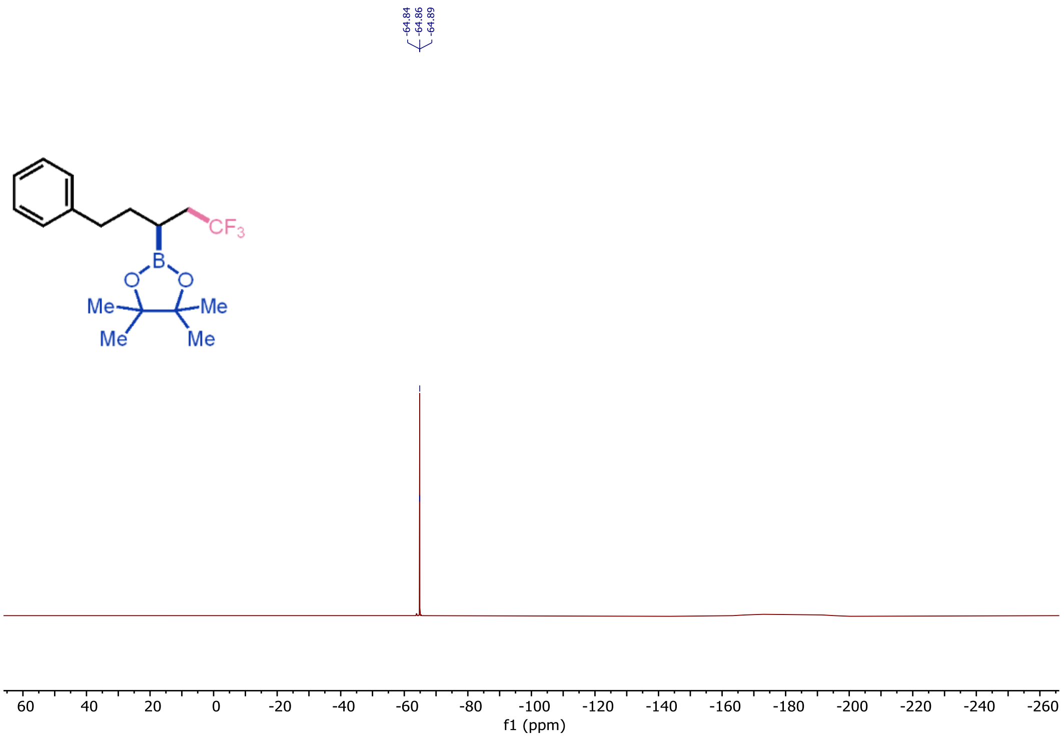

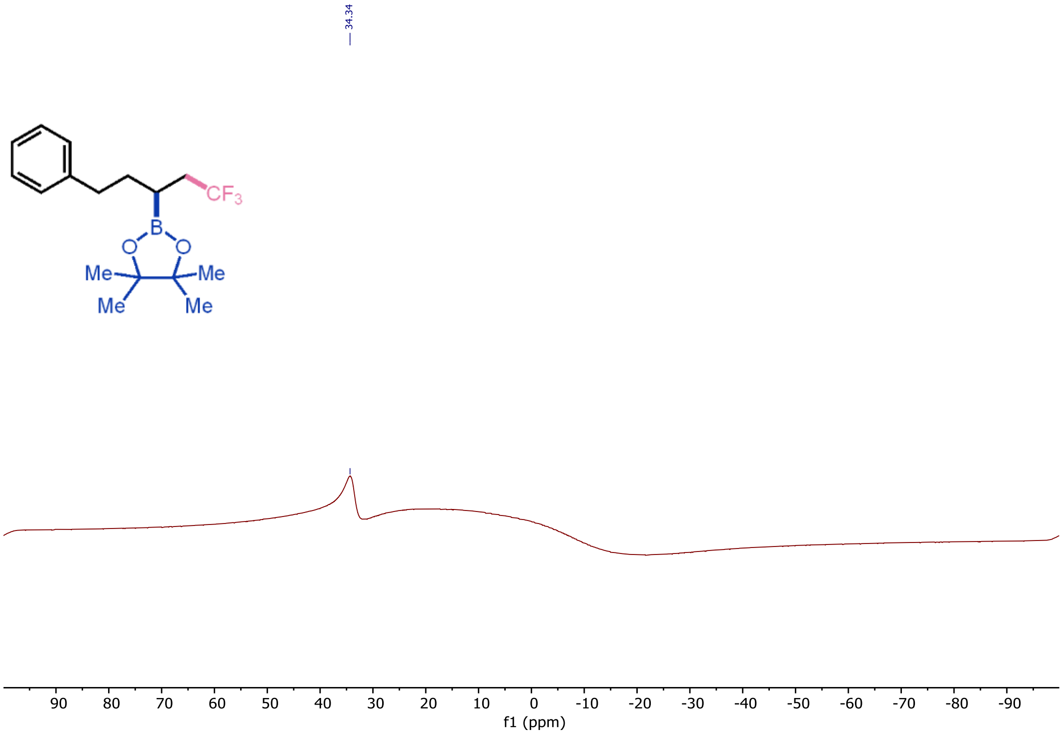

Figure S67. ^{1}H NMR (500 MHz, $CDCl_3$), ^{13}C NMR (101 MHz, $CDCl_3$), ^{19}F NMR (376 MHz, $CDCl_3$) and ^{11}B NMR (160 MHz, $CDCl_3$) spectra of **11**.

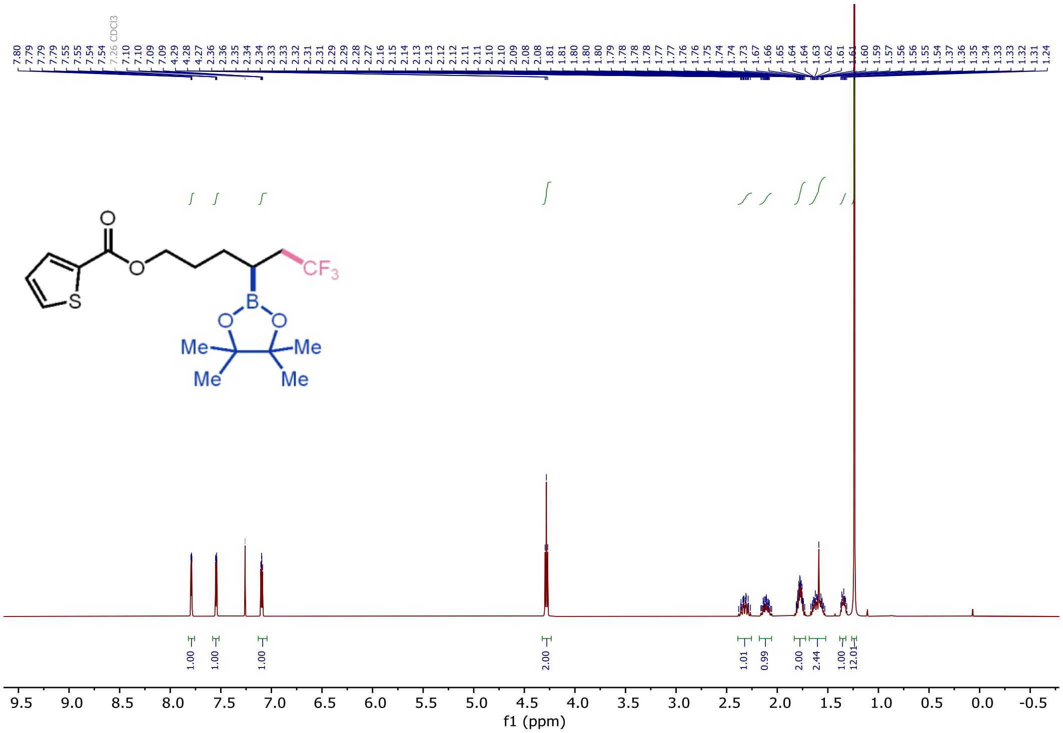
7.80 7.79 7.79 7.79 7.55 7.55 7.54 7.54 7.26 CDCl3 7.10 7.10 7.09 7.09 4.29 4.28 4.27 2.36 2.36 2.35 2.34 2.34 2.33 2.33 2.32 2.31 2.31 2.29 2.29 2.28 2.27 2.16 2.15 2.14 2.13 2.13 2.12 2.12 2.11 2.11 2.10 2.10 2.09 2.08 2.08 1.81 1.81 1.80 1.80 1.79 1.78 1.78 1.78 1.77 1.77 1.76 1.76 1.75 1.74 1.74 1.73 1.67 1.66 1.65 1.64 1.64 1.63 1.62 1.61 1.61 1.60 1.59 1.57 1.56 1.56 1.55 1.54 1.37 1.36 1.35 1.34 1.33 1.33 1.32 1.31 1.24
CF3
Me
1.00 1.00 1.00 2.00 1.01 0.99 2.00 2.44 1.00 12.01
9.5 9.0 8.5 8.0 7.5 7.0 6.5 6.0 5.5 5.0 4.5 4.0 3.5 3.0 2.5 2.0 1.5 1.0 0.5 0.0 -0.5
f1 (ppm)

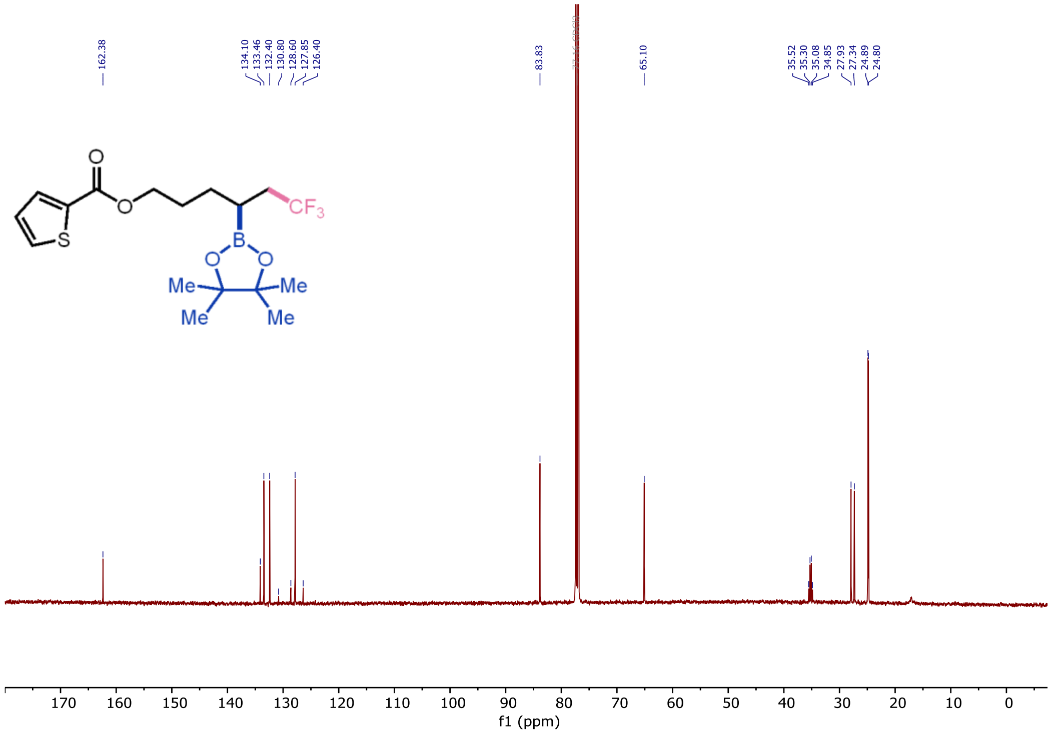
162.38
134.10 133.46 132.40 130.80 128.60 127.85 126.40
83.83
65.10
35.52 35.30 35.08 34.85 27.93 27.34 24.89 24.80
CF3
Me
170 160 150 140 130 120 110 100 90 80 70 60 50 40 30 20 10 0
f1 (ppm)

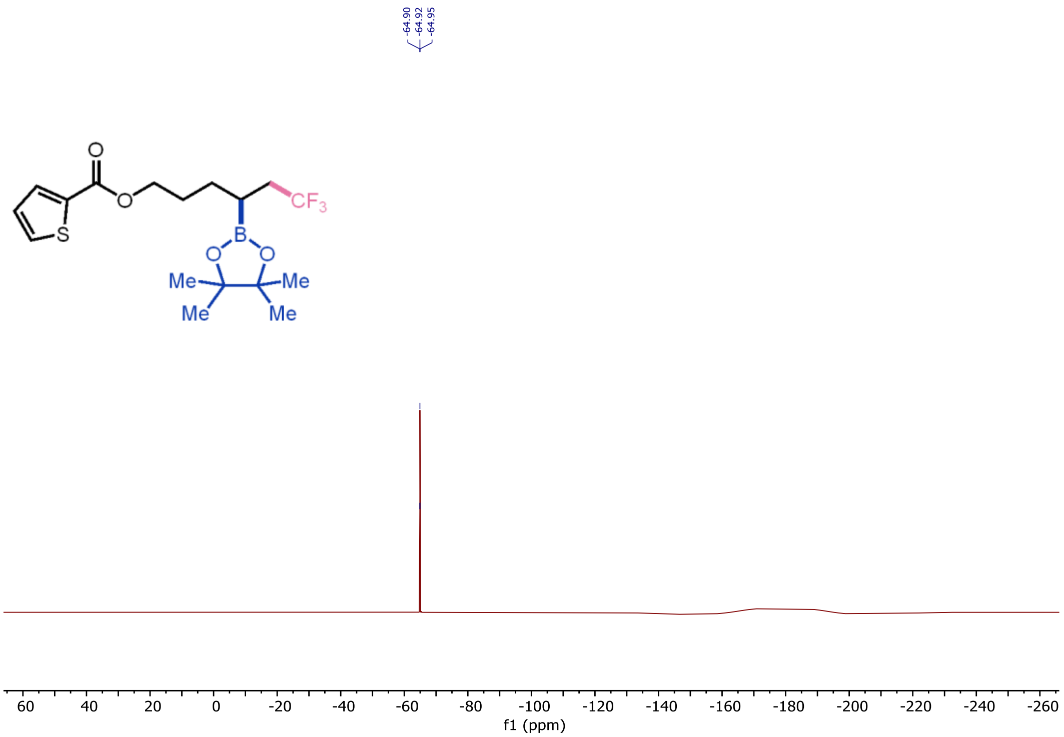

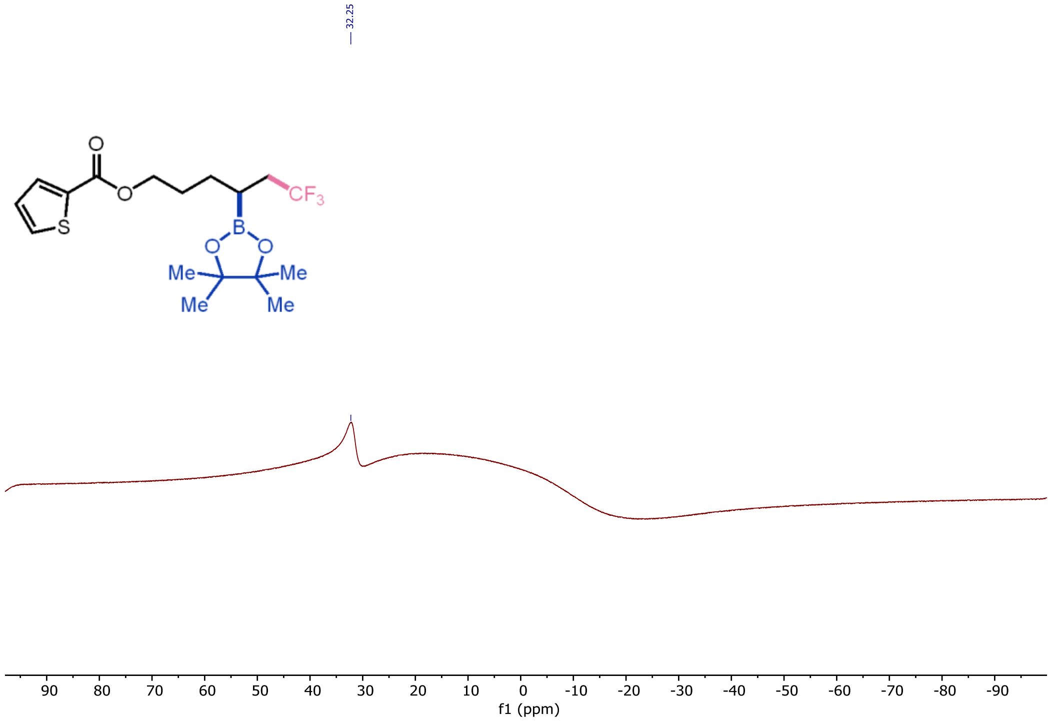

Figure S68. ^{1}H NMR (500 MHz, $CDCl_3$), ^{13}C NMR (101 MHz, $CDCl_3$), ^{19}F NMR (376 MHz, $CDCl_3$) and ^{11}B NMR (160 MHz, $CDCl_3$) spectra of **12**.

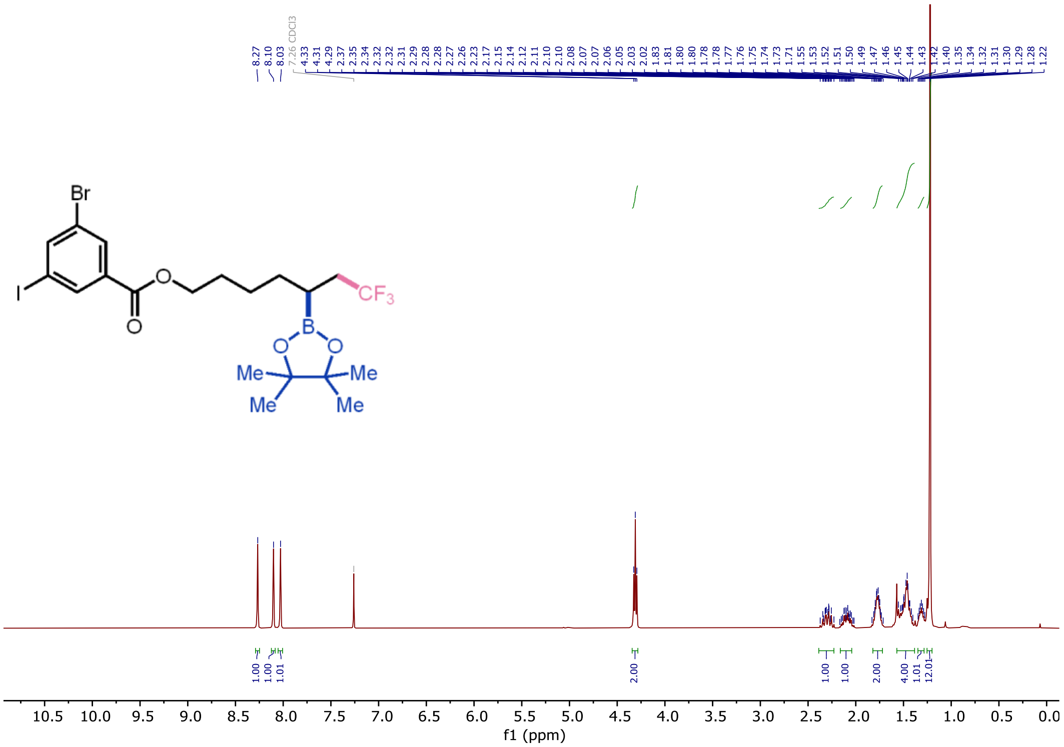
8.27
8.10
8.03
7.26 CDCl3
4.33
4.31
4.29
2.37
2.35
2.34
2.32
2.32
2.31
2.29
2.28
2.28
2.27
2.26
2.23
2.17
2.15
2.14
2.12
2.11
2.10
2.08
2.07
2.06
2.05
2.03
2.02
1.83
1.81
1.80
1.78
1.78
1.77
1.76
1.75
1.74
1.73
1.71
1.55
1.53
1.52
1.51
1.50
1.49
1.47
1.46
1.45
1.44
1.43
1.42
1.40
1.35
1.34
1.32
1.31
1.30
1.29
1.28
1.22
Br
I
O
O
CF3
B
O
O
Me
Me
Me
Me
1.00
1.00
1.01
2.00
1.00
1.00
2.00
4.00
1.01
12.01
f1 (ppm)

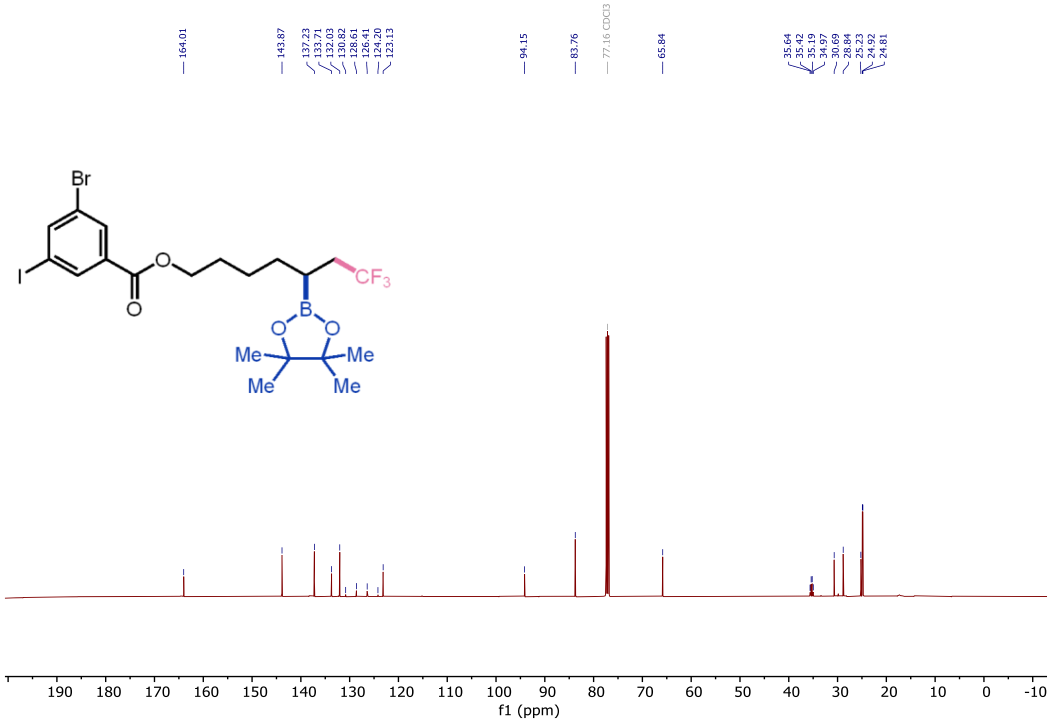
164.01
143.87
137.23
133.71
132.03
130.82
128.61
126.41
124.20
123.13
94.15
83.76
77.16 CDCl3
65.84
35.64
35.42
35.19
34.97
30.69
28.84
25.23
24.92
24.81
Br
I
O
O
CF3
B
O
O
Me
Me
Me
Me
f1 (ppm)

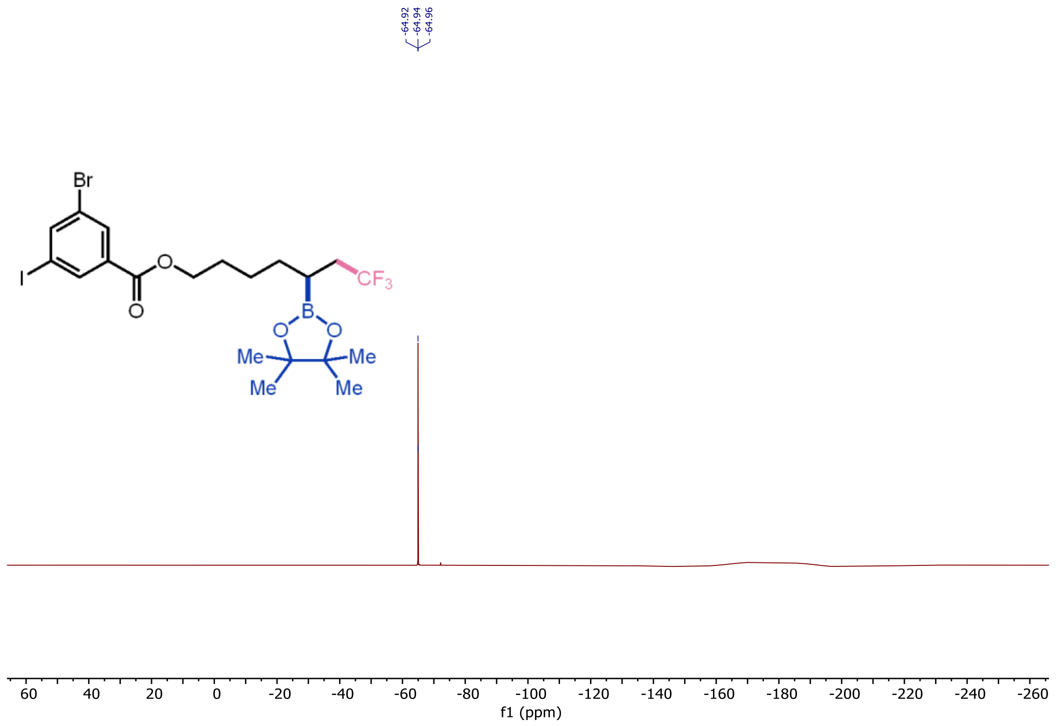

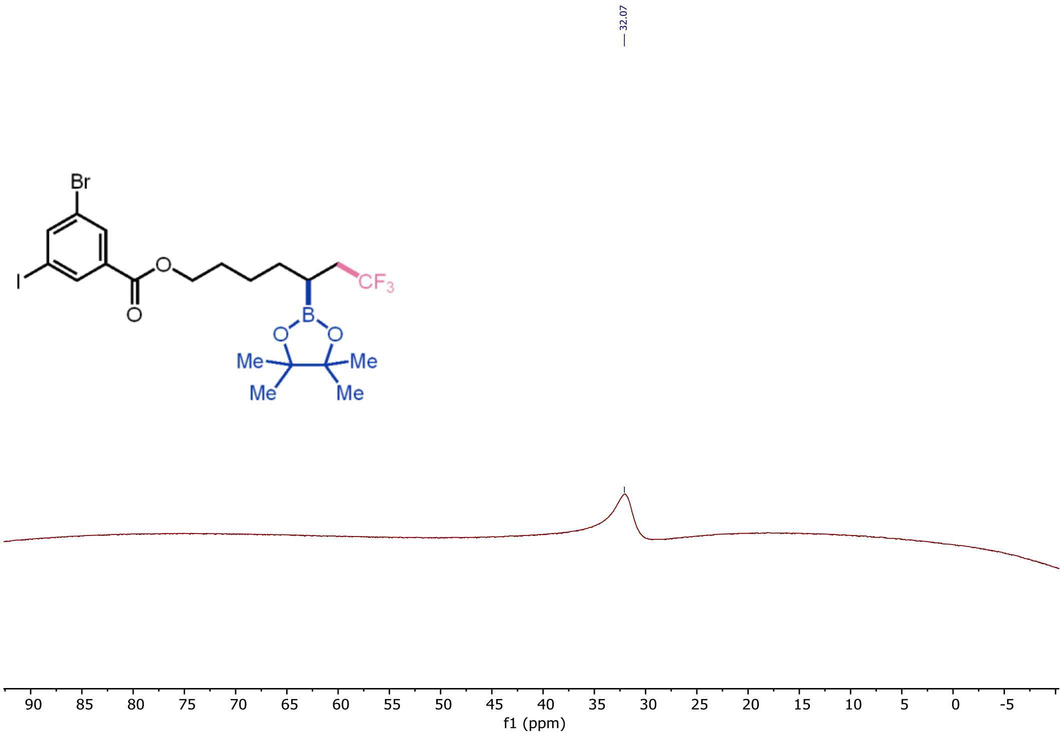

Figure S69. ^{1}H NMR (500 MHz, $CDCl_3$), ^{13}C NMR (101 MHz, $CDCl_3$), ^{19}F NMR (376 MHz, $CDCl_3$) and ^{11}B NMR (160 MHz, $CDCl_3$) spectra of **13**.

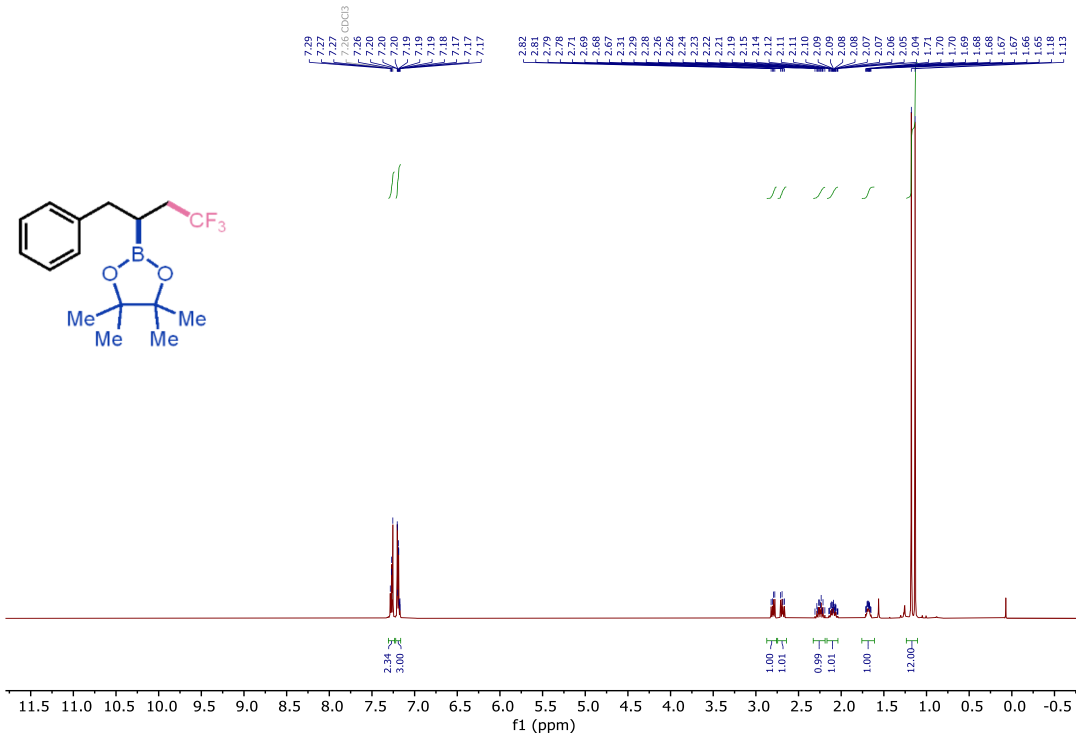
7.29 7.27 7.27 7.26 CDCl3 7.26 7.20 7.20 7.20 7.19 7.19 7.19 7.18 7.17 7.17 7.17
2.82 2.81 2.79 2.78 2.71 2.69 2.68 2.67 2.31 2.29 2.28 2.26 2.26 2.24 2.23 2.22 2.21 2.19 2.15 2.14 2.12 2.11 2.11 2.10 2.09 2.09 2.08 2.08 2.07 2.06 2.05 2.04 1.71 1.70 1.70 1.69 1.68 1.68 1.67 1.67 1.66 1.65 1.18 1.13
CF3
B
O
O
Me
Me
Me
Me
2.34
3.00
1.00
1.01
0.99
1.01
1.00
12.00
11.5 11.0 10.5 10.0 9.5 9.0 8.5 8.0 7.5 7.0 6.5 6.0 5.5 5.0 4.5 4.0 3.5 3.0 2.5 2.0 1.5 1.0 0.5 0.0 -0.5
f1 (ppm)

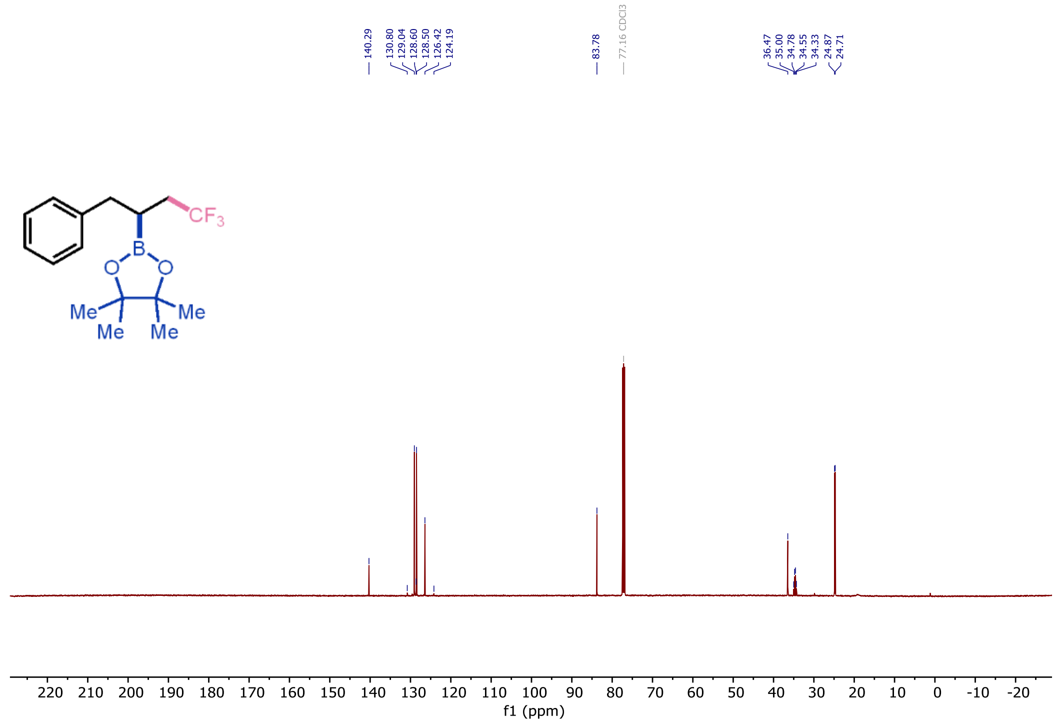
140.29
130.80 129.04 128.60 128.50 126.42 124.19
83.78
77.16 CDCl3
36.47 35.00 34.78 34.55 34.33 24.87 24.71
CF3
B
O
O
Me
Me
Me
Me
220 210 200 190 180 170 160 150 140 130 120 110 100 90 80 70 60 50 40 30 20 10 0 -10 -20
f1 (ppm)

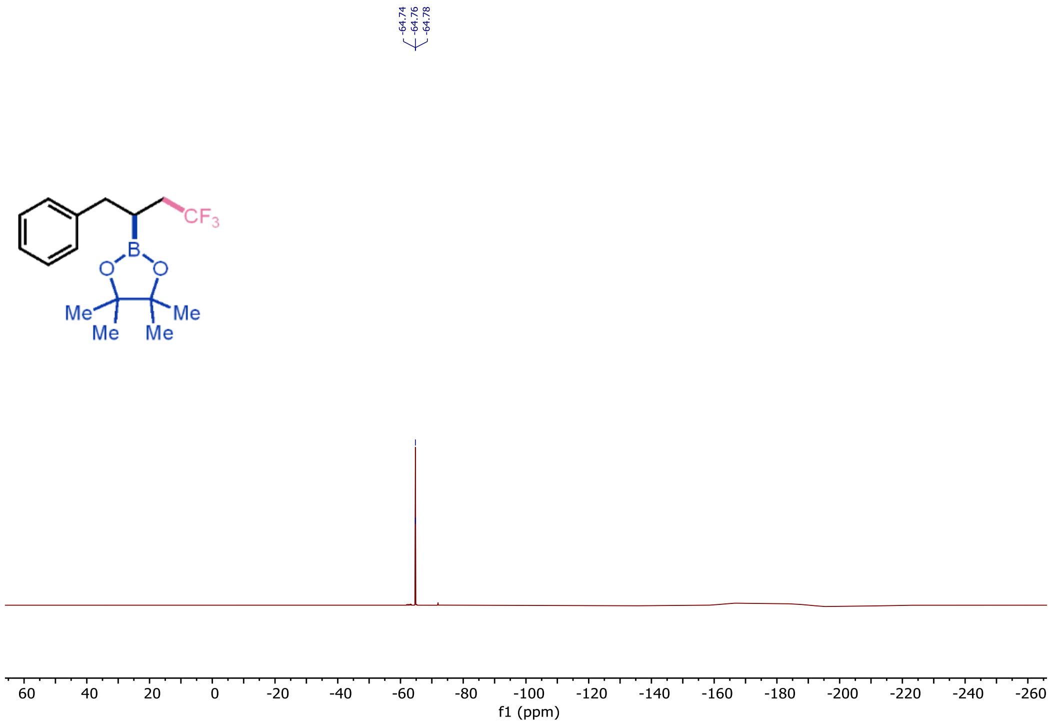

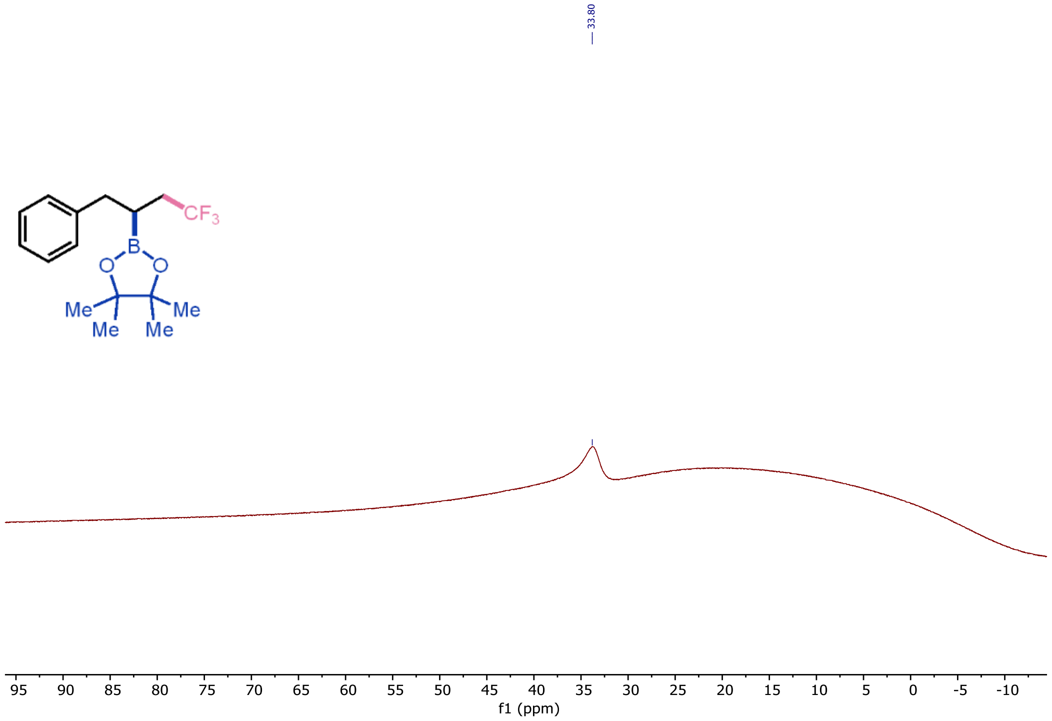

Figure S70. ^{1}H NMR (500 MHz, $CDCl_3$), ^{13}C NMR (101 MHz, $CDCl_3$), ^{19}F NMR (376 MHz, $CDCl_3$) and ^{11}B NMR (160 MHz, $CDCl_3$) spectra of **14**.

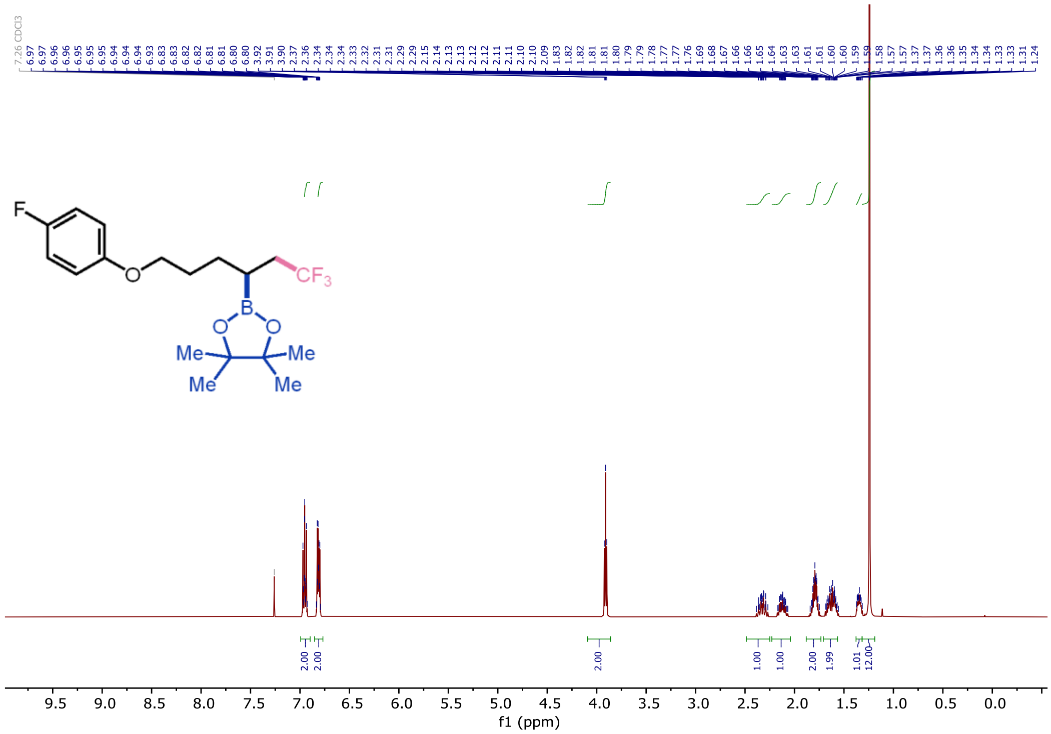
F
O
B
O
O
Me
Me
Me
Me
CF3
7.26 CDCl3
2.00
2.00
2.00
1.00
1.00
2.00
1.99
1.01
12.00
f1 (ppm)

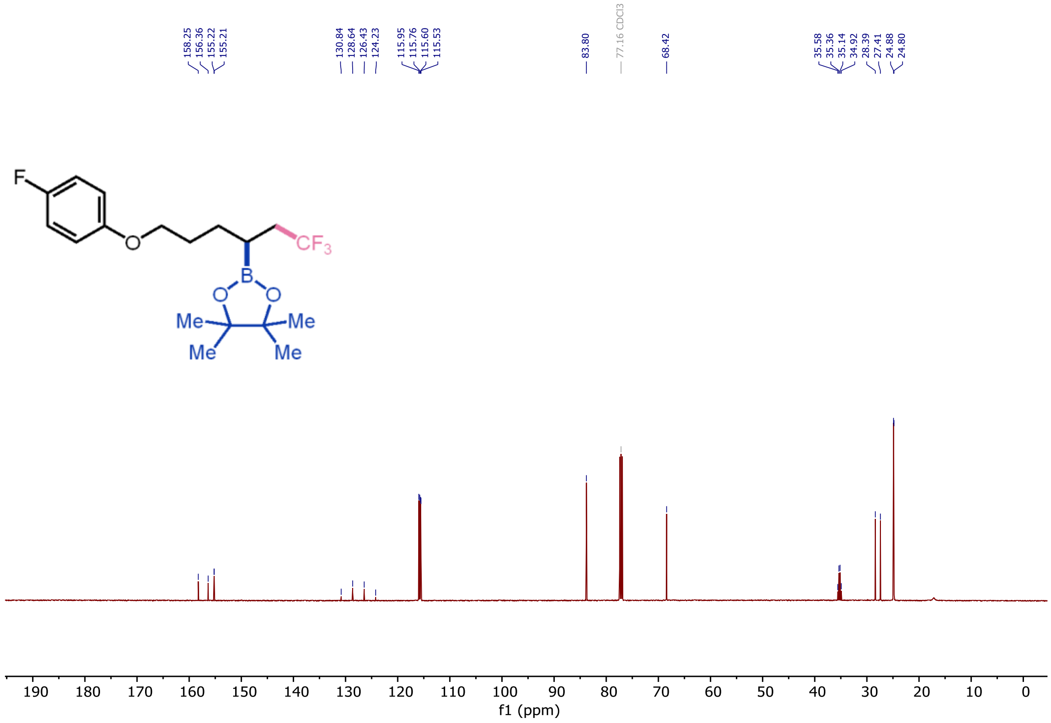
F
O
B
O
O
Me
Me
Me
Me
CF3
158.25
156.36
155.22
155.21
130.84
128.64
126.43
124.23
115.95
115.76
115.60
115.53
83.80
77.16 CDCl3
68.42
35.58
35.36
35.14
34.92
28.39
27.41
24.88
24.80
f1 (ppm)

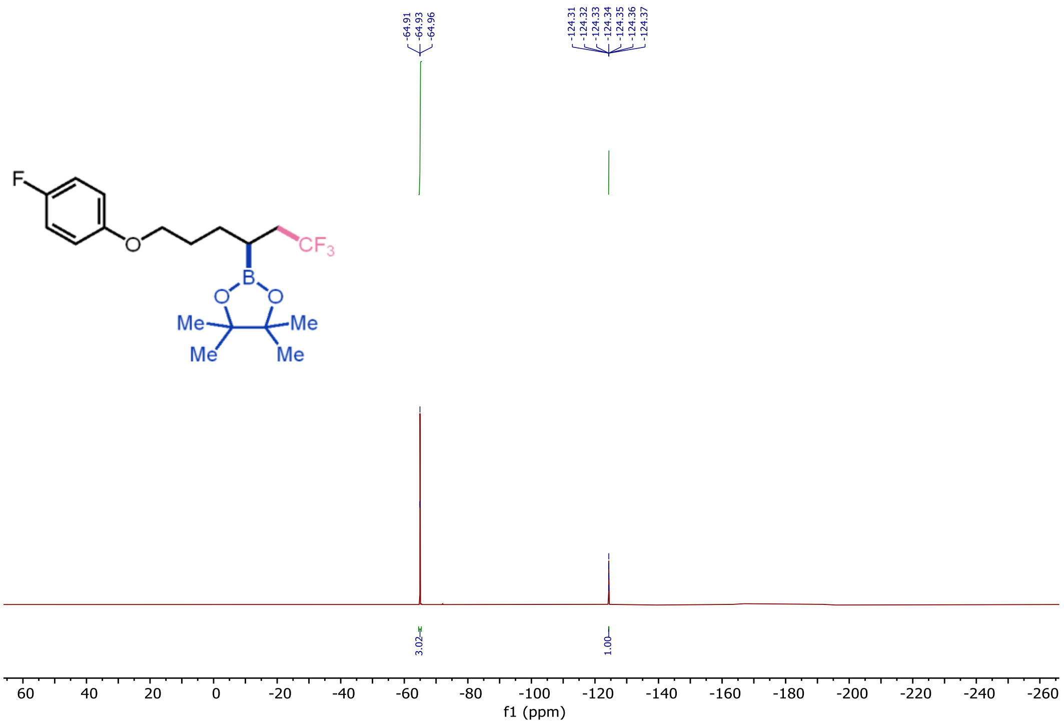

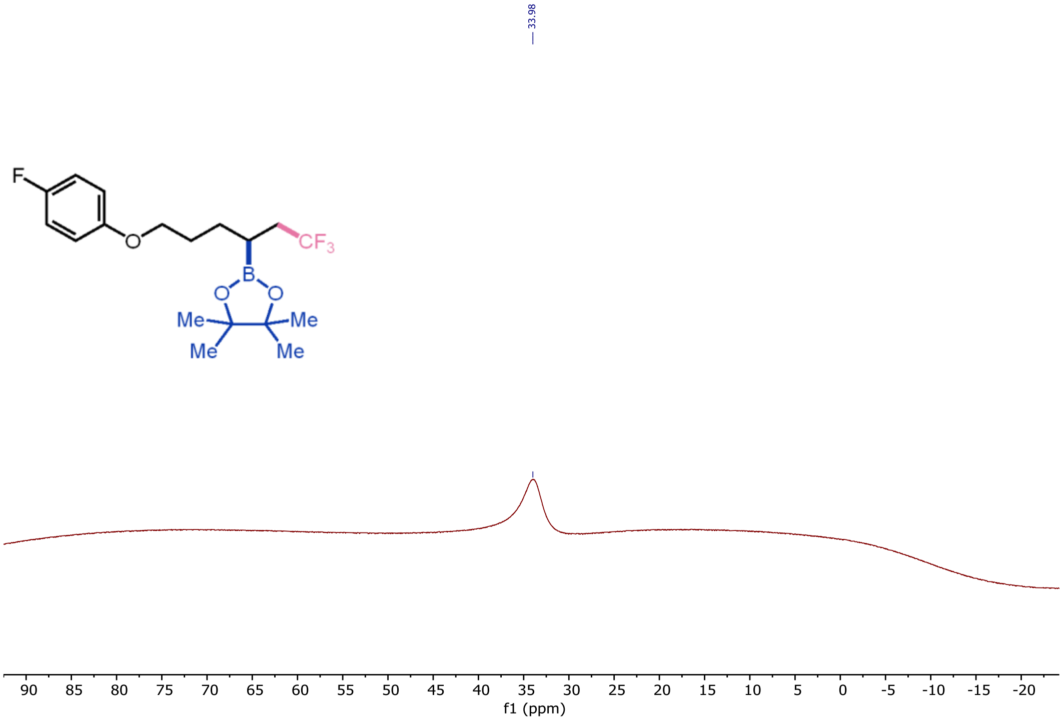

Figure S71. ^{1}H NMR (500 MHz, $CDCl_3$), ^{13}C NMR (101 MHz, $CDCl_3$), ^{19}F NMR (376 MHz, $CDCl_3$) and ^{11}B NMR (160 MHz, $CDCl_3$) spectra of **15**.

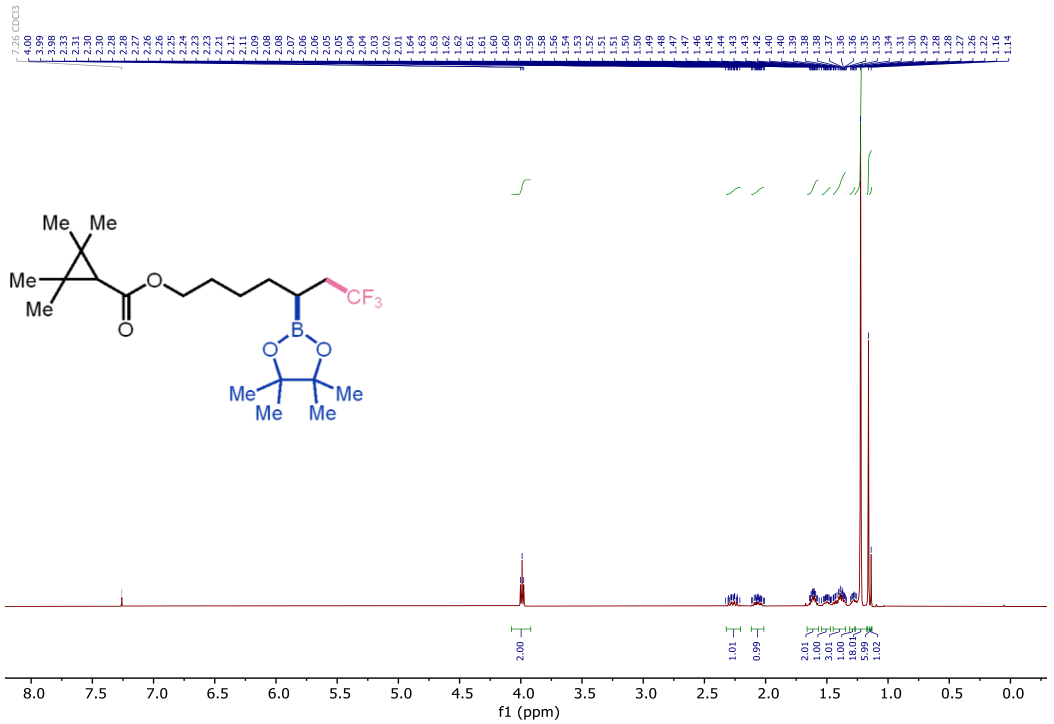

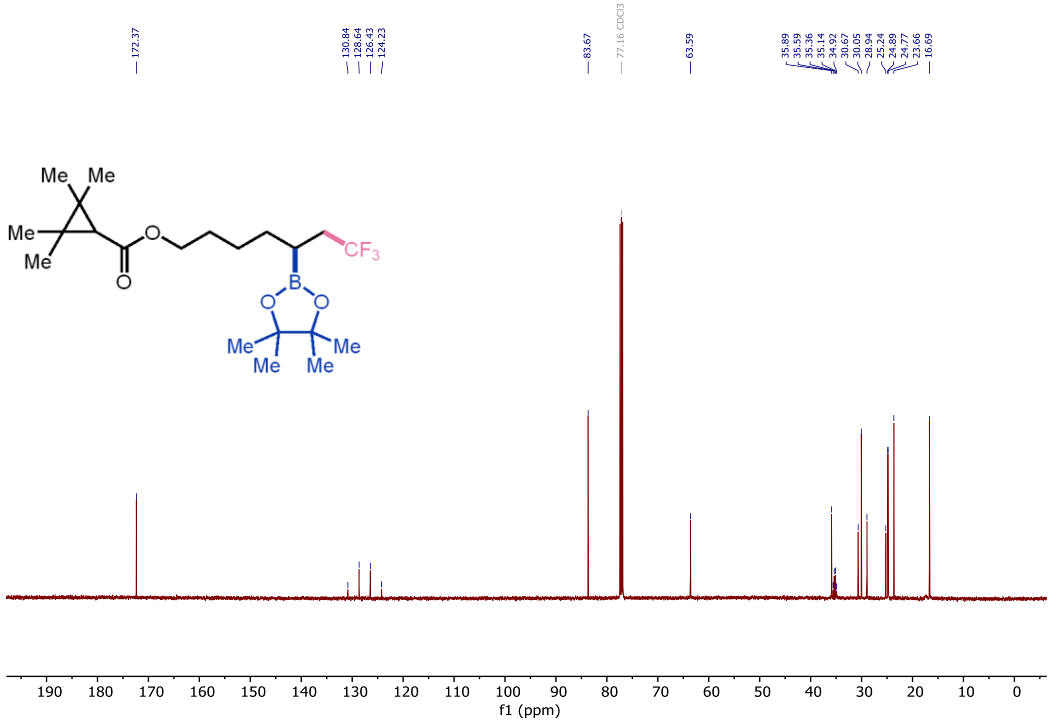

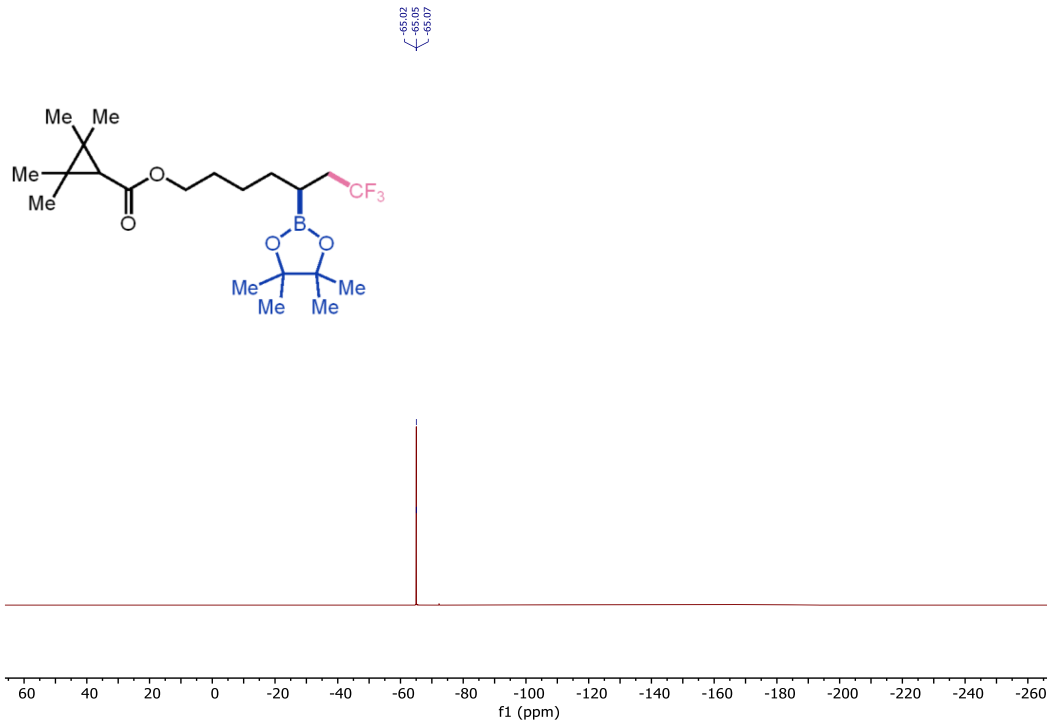

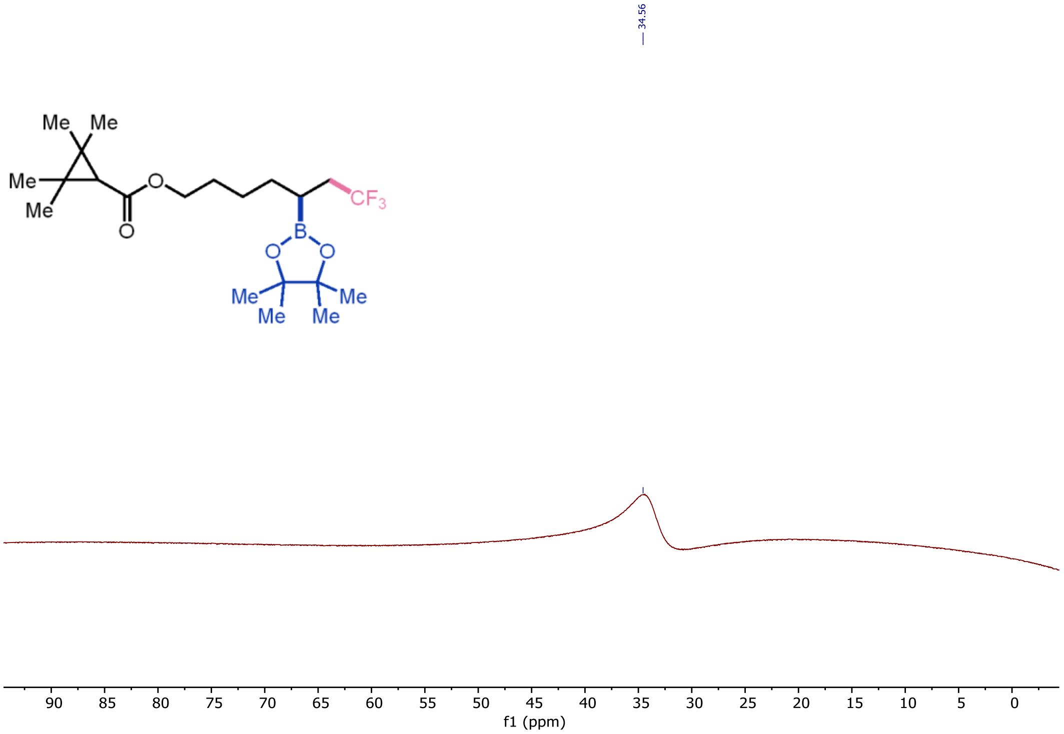

Figure S72. ^{1}H NMR (500 MHz, $CDCl_3$), ^{13}C NMR (101 MHz, $CDCl_3$), ^{19}F NMR (376 MHz, $CDCl_3$) and ^{11}B NMR (160 MHz, $CDCl_3$) spectra of **16**.

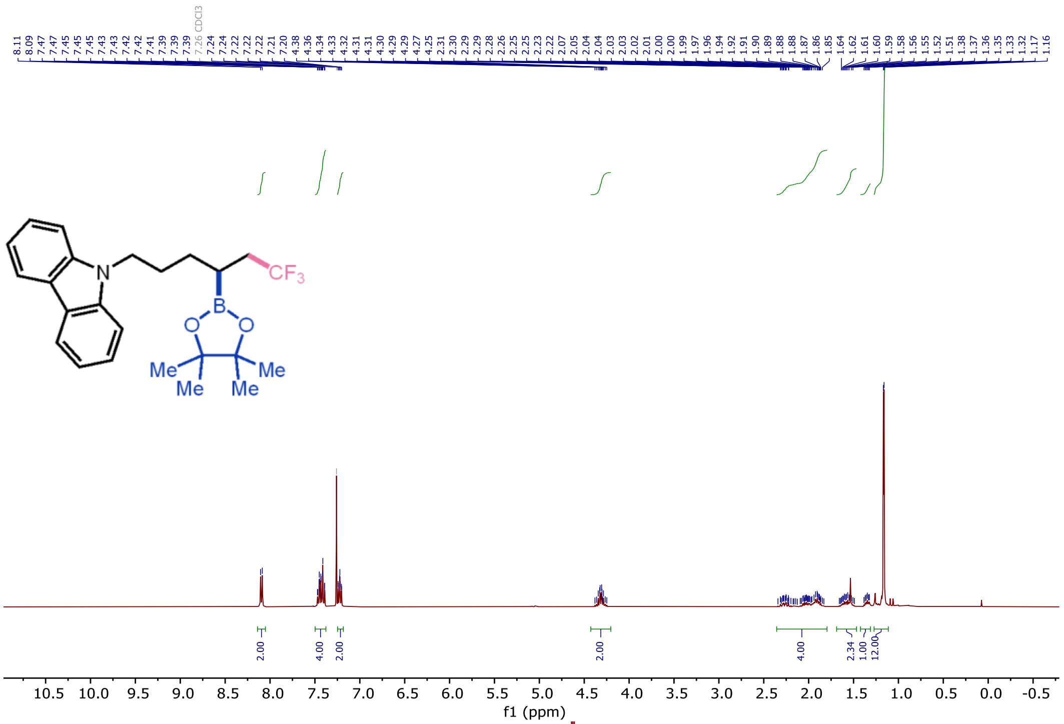
8.11 8.09 7.47 7.47 7.45 7.45 7.43 7.43 7.42 7.42 7.41 7.39 7.39 7.39 7.26 CDCl3 7.24 7.24 7.22 7.22 7.22 7.21 7.20 4.38 4.36 4.34 4.33 4.32 4.31 4.31 4.30 4.29 4.29 4.27 4.25 2.31 2.30 2.29 2.29 2.28 2.26 2.25 2.25 2.23 2.22 2.07 2.05 2.04 2.04 2.03 2.03 2.02 2.01 2.00 2.00 1.99 1.97 1.96 1.94 1.92 1.91 1.90 1.89 1.88 1.88 1.87 1.86 1.85 1.64 1.62 1.61 1.60 1.59 1.58 1.56 1.55 1.52 1.51 1.38 1.37 1.36 1.35 1.33 1.32 1.17 1.16
N
CF3
B
O O
Me Me Me Me
2.00 4.00 2.00 2.00 4.00 2.34 1.00 12.00
10.5 10.0 9.5 9.0 8.5 8.0 7.5 7.0 6.5 6.0 5.5 5.0 4.5 4.0 3.5 3.0 2.5 2.0 1.5 1.0 0.5 0.0 -0.5
f1 (ppm)

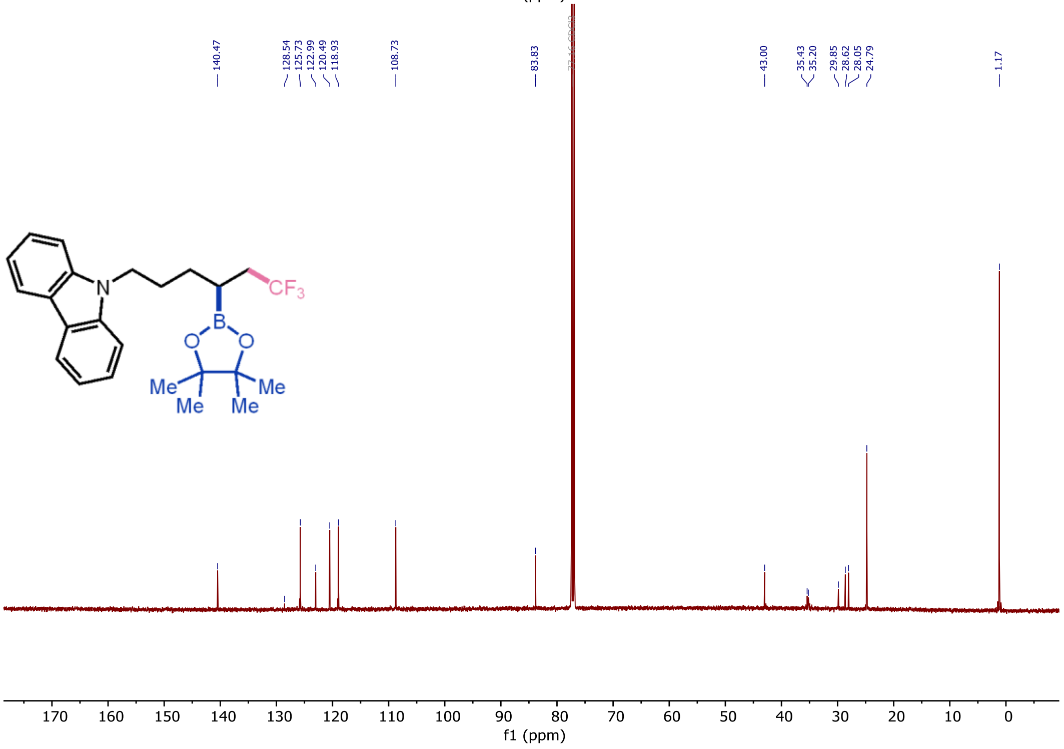
140.47 128.54 125.73 122.99 120.49 118.93 108.73 83.83 43.00 35.43 35.20 29.85 28.62 28.05 24.79 1.17
N
CF3
B
O O
Me Me Me Me
170 160 150 140 130 120 110 100 90 80 70 60 50 40 30 20 10 0
f1 (ppm)

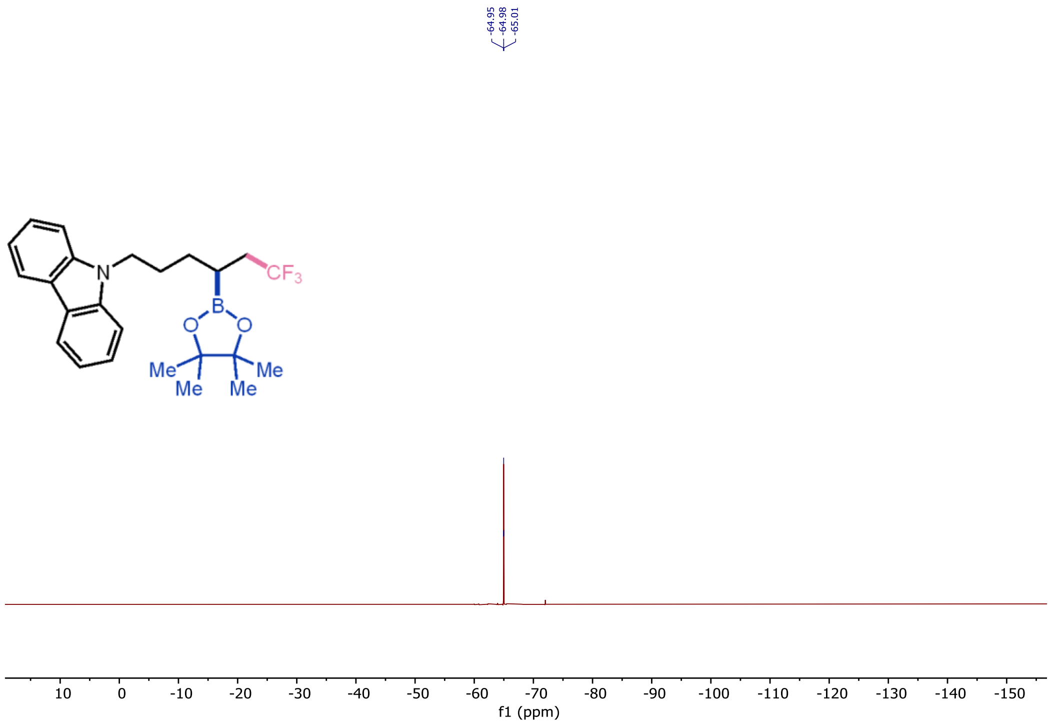

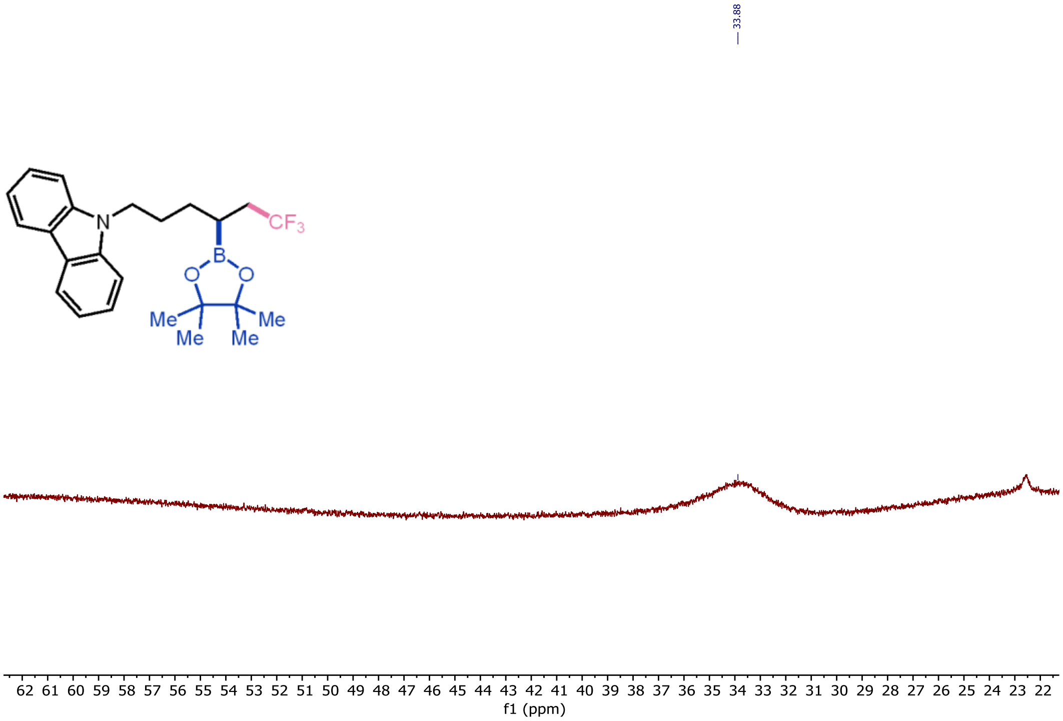

Figure S73. ^{1}H NMR (500 MHz, $CDCl_3$), ^{13}C NMR (101 MHz, $CDCl_3$), ^{19}F NMR (376 MHz, $CDCl_3$) and ^{11}B NMR (160 MHz, $CDCl_3$) spectra of **17**.

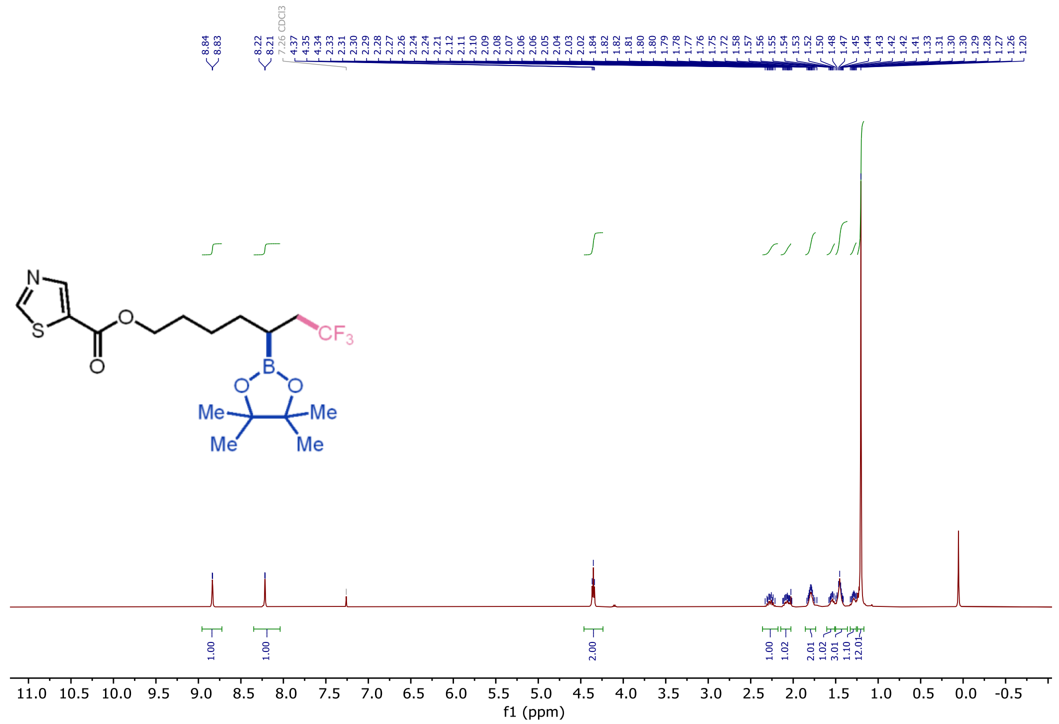

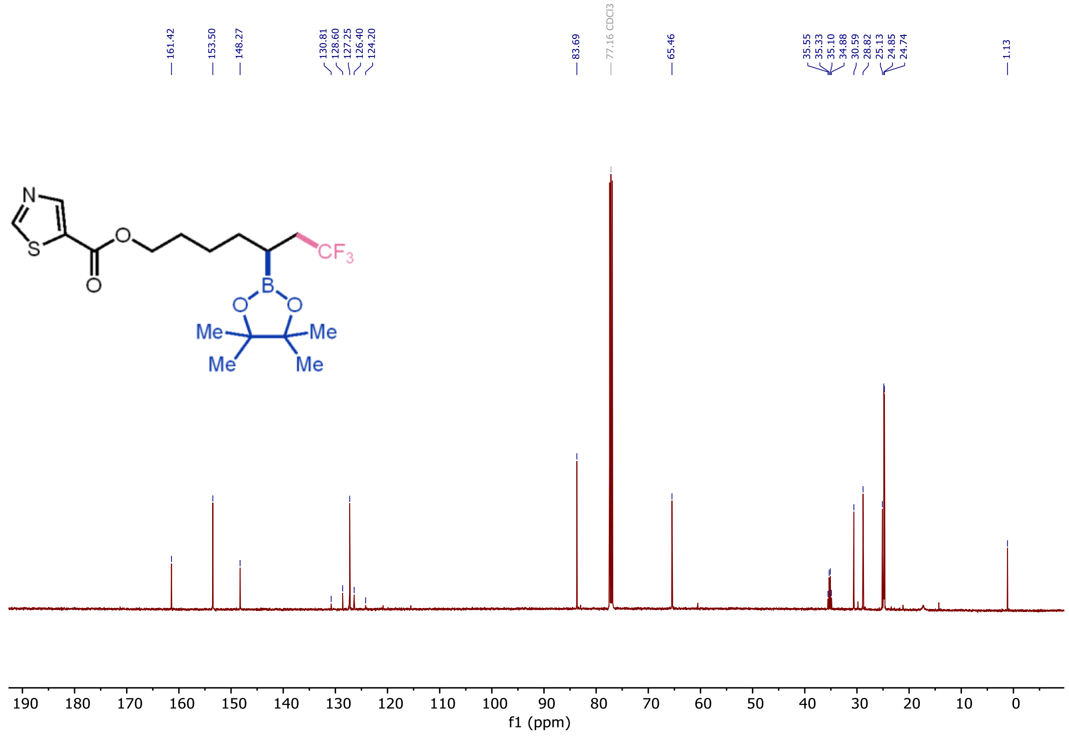

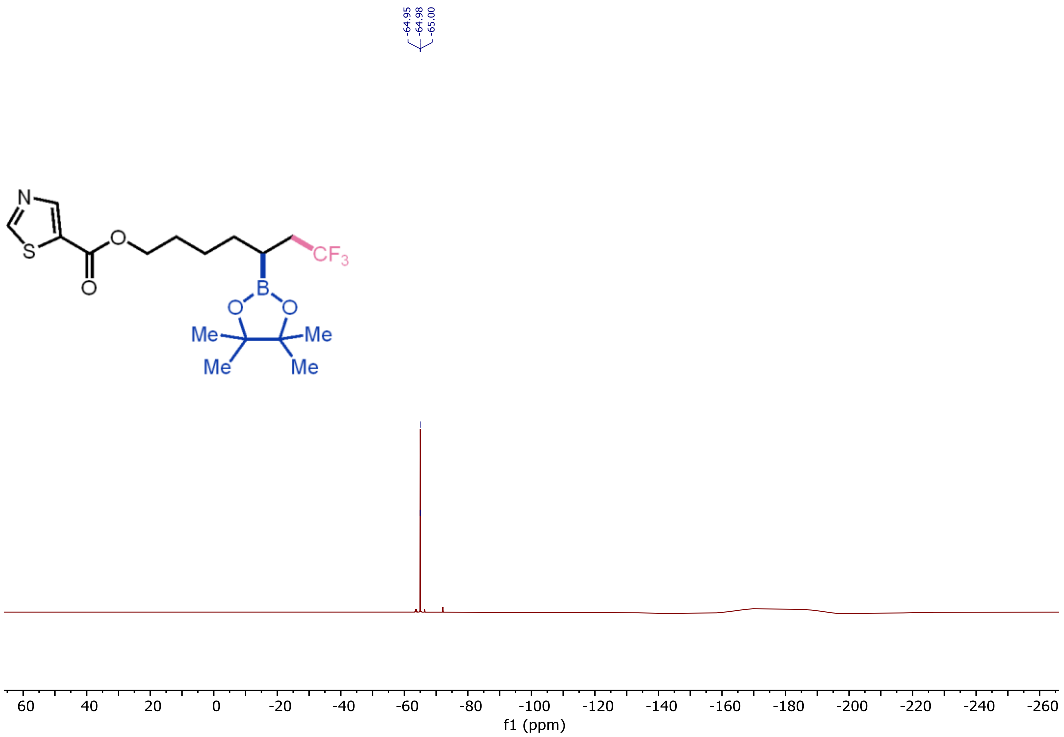

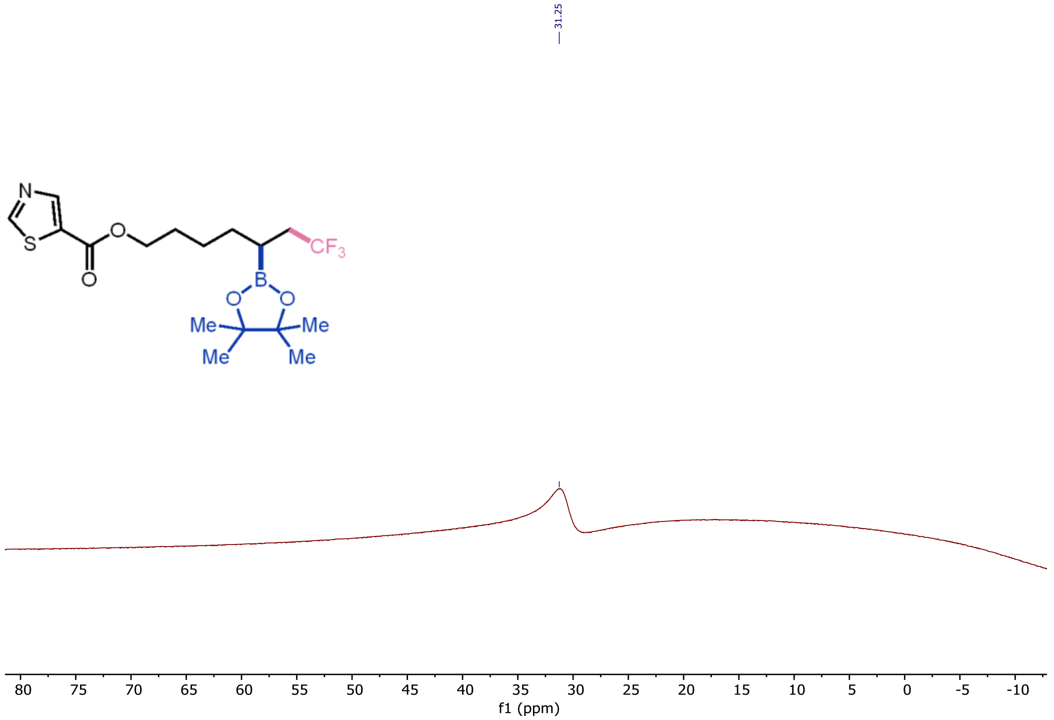

Figure S74. ^{1}H NMR (500 MHz, $CDCl_3$), ^{13}C NMR (101 MHz, $CDCl_3$), ^{19}F NMR (376 MHz, $CDCl_3$) and ^{11}B NMR (160 MHz, $CDCl_3$) spectra of **18**.

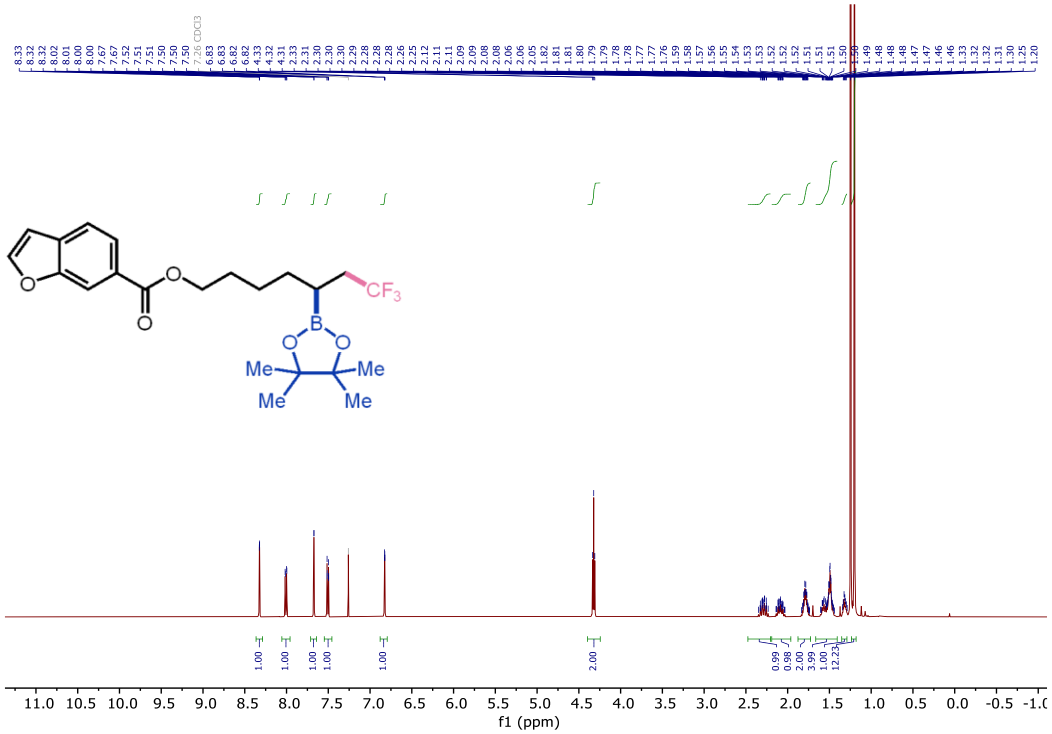

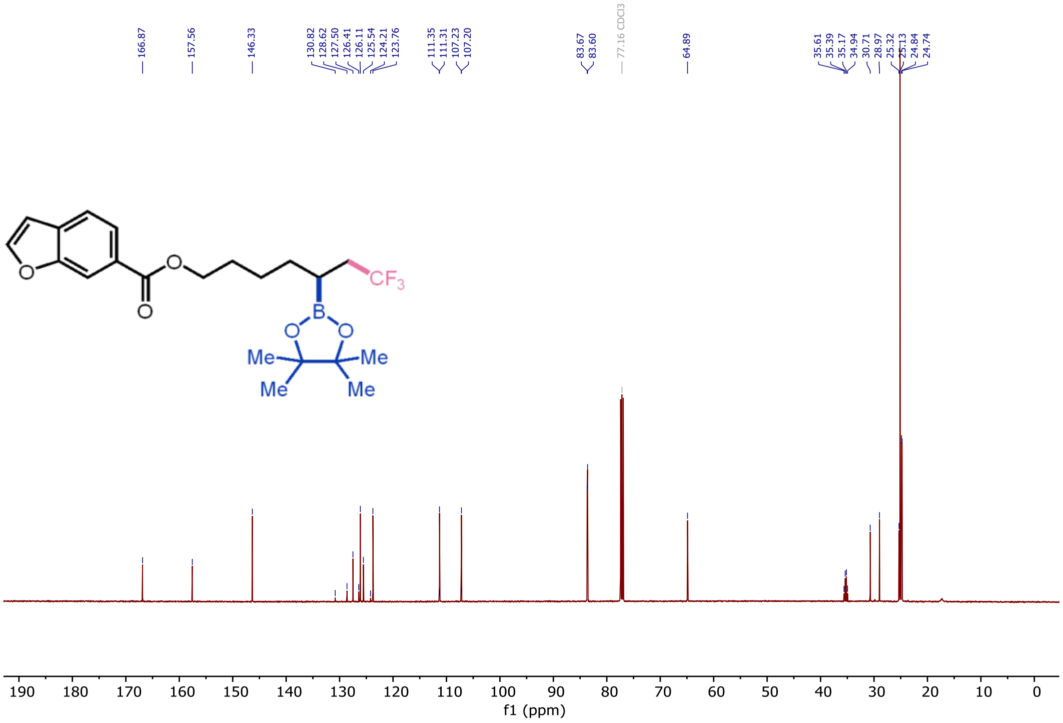

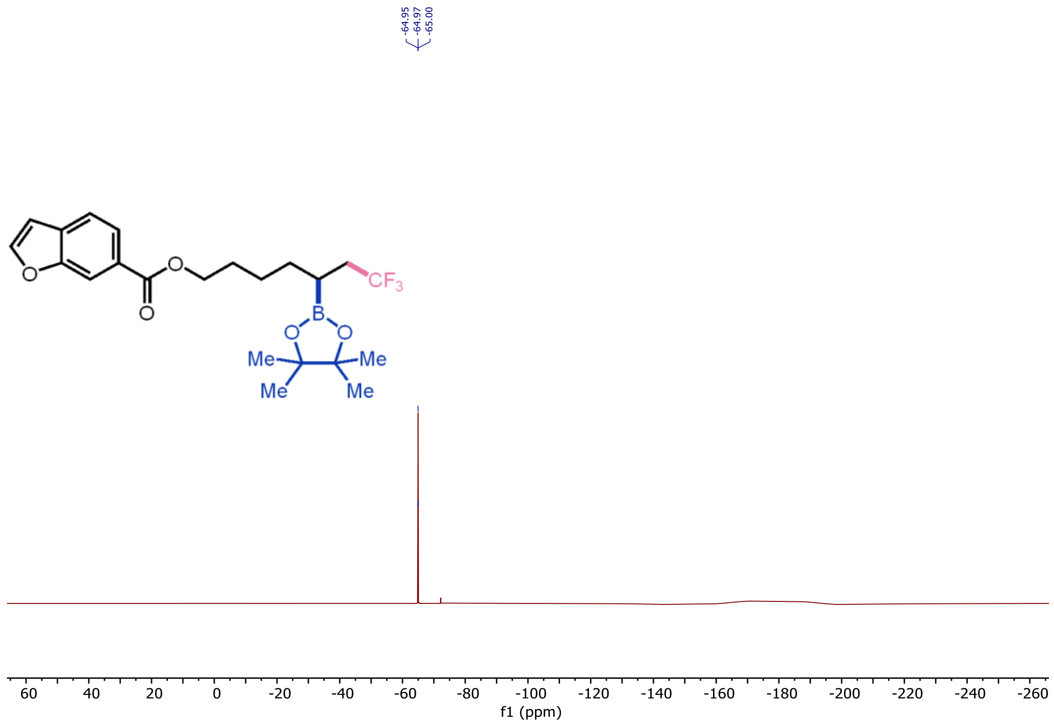

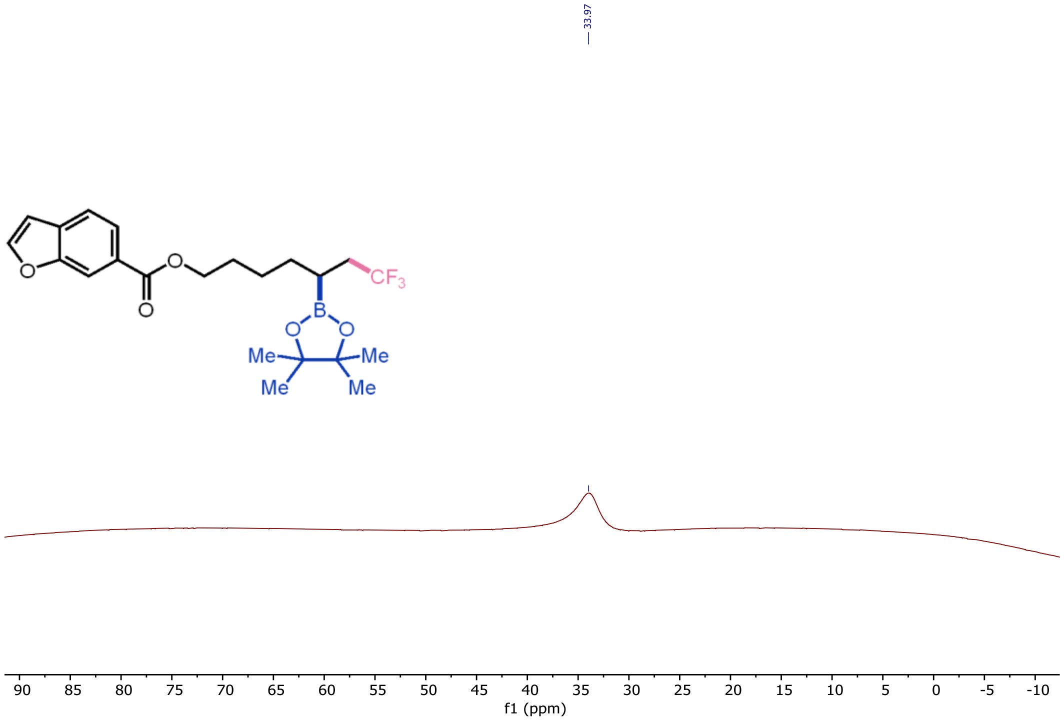

Figure S75. ^{1}H NMR (500 MHz, $CDCl_3$), ^{13}C NMR (101 MHz, $CDCl_3$), ^{19}F NMR (376 MHz, $CDCl_3$) and ^{11}B NMR (160 MHz, $CDCl_3$) spectra of **19**.

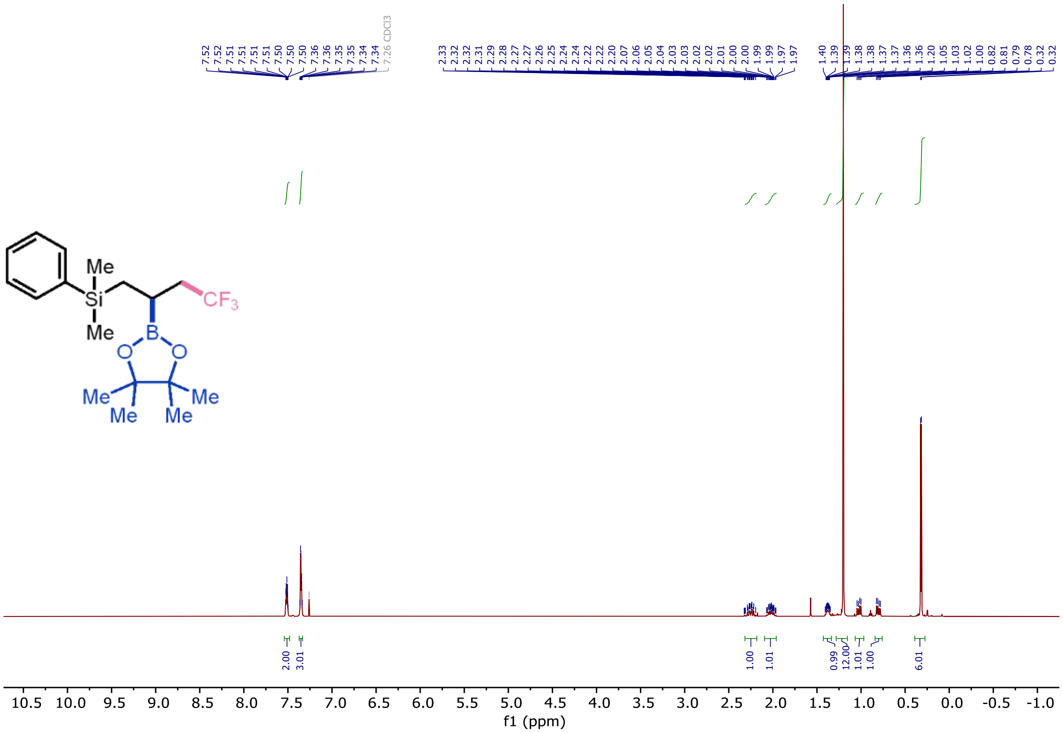

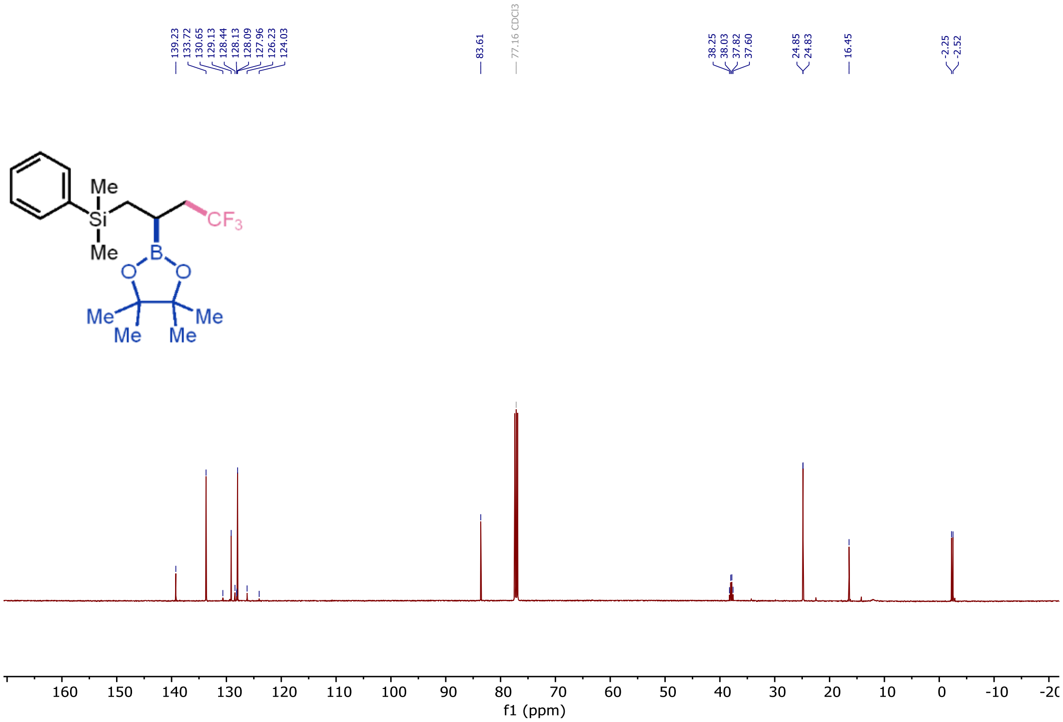

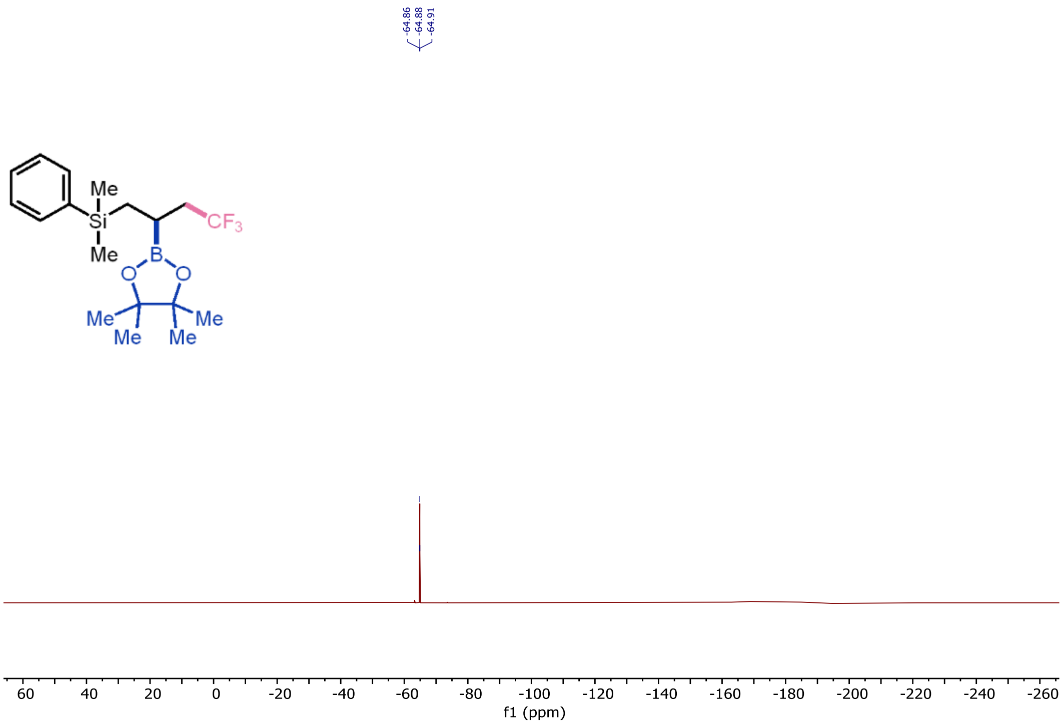

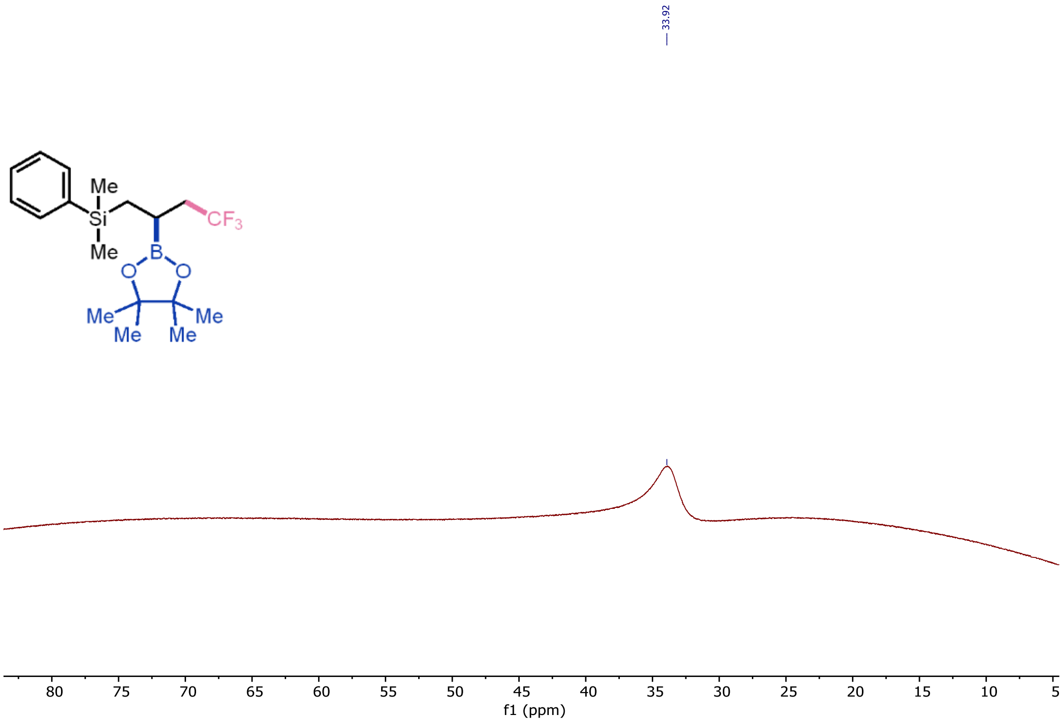

Figure S76. ^{1}H NMR (500 MHz, $CDCl_3$), ^{13}C NMR (101 MHz, $CDCl_3$), ^{19}F NMR (376 MHz, $CDCl_3$) and ^{11}B NMR (160 MHz, $CDCl_3$) spectra of **20**.

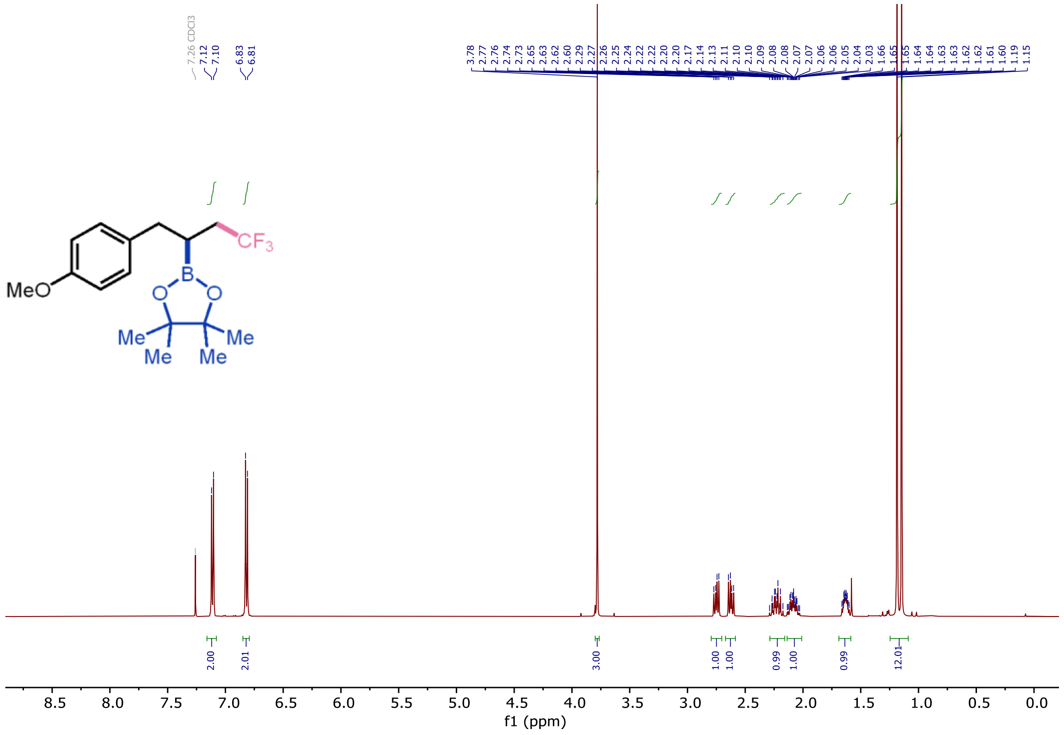

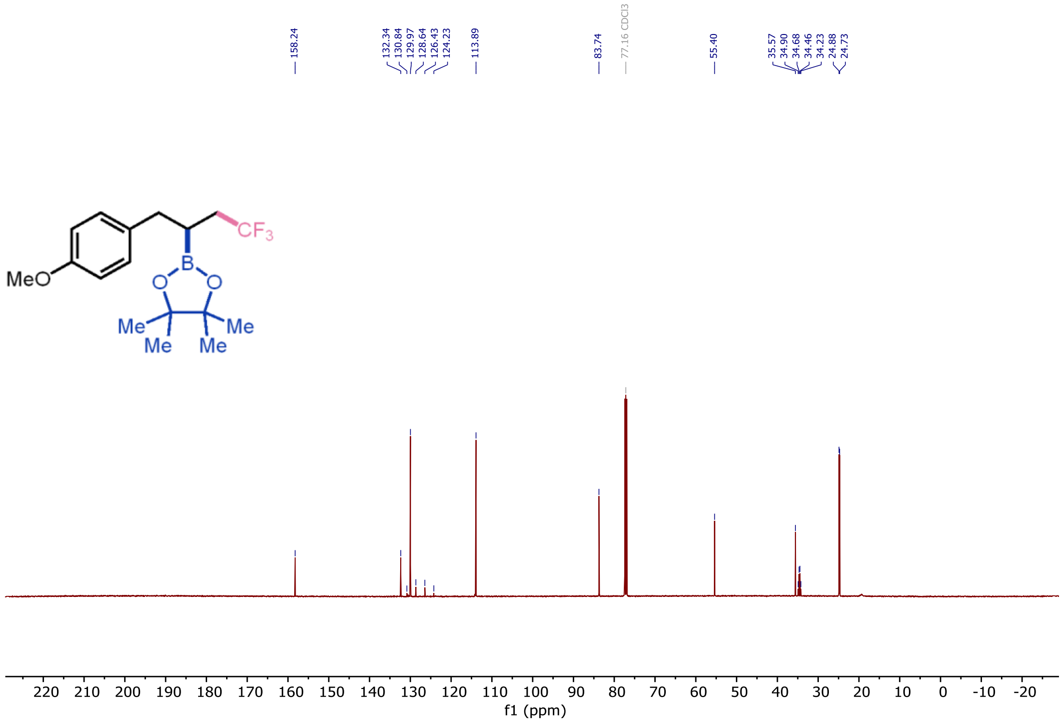

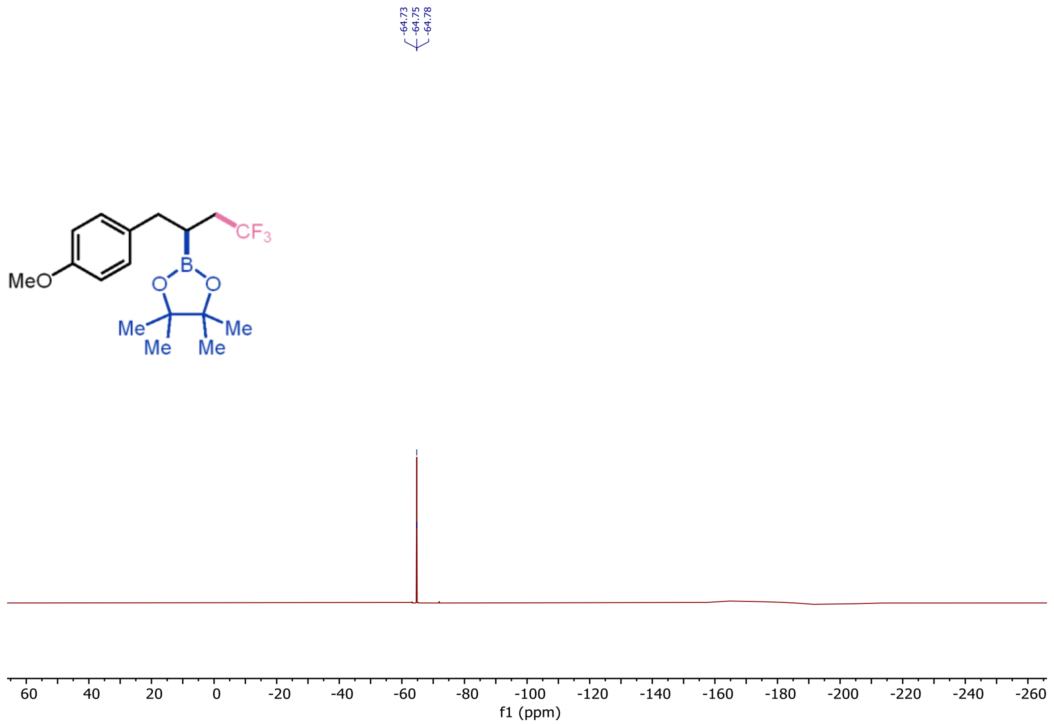

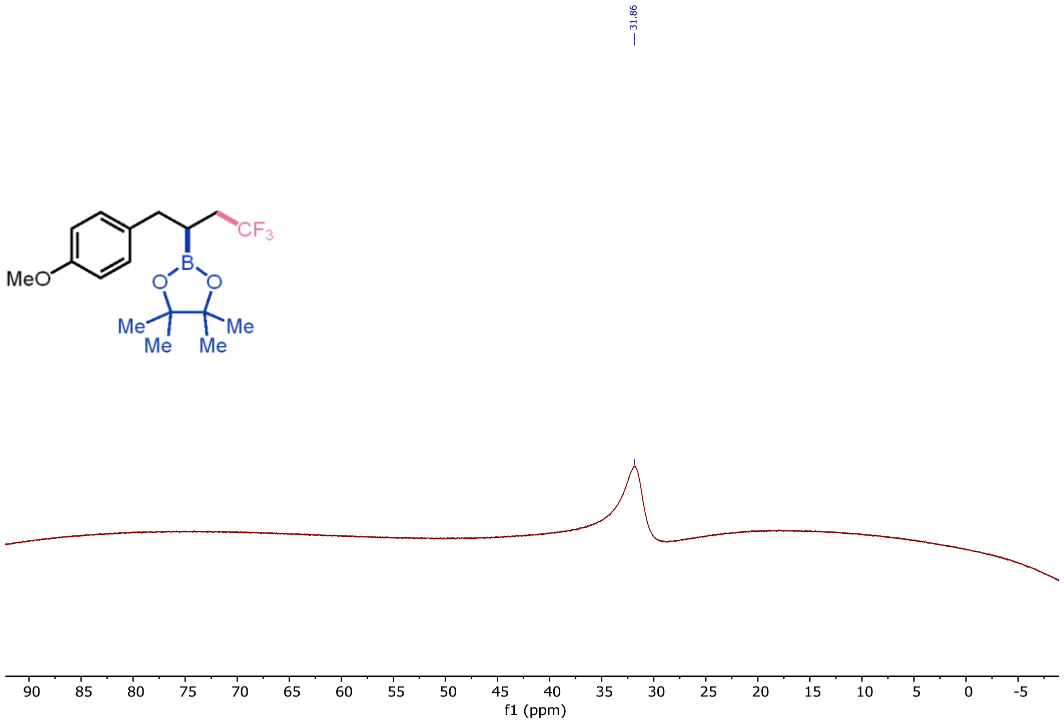

Figure S77. ^{1}H NMR (500 MHz, $CDCl_3$), ^{13}C NMR (101 MHz, $CDCl_3$), ^{19}F NMR (376 MHz, $CDCl_3$) and ^{11}B NMR (160 MHz, $CDCl_3$) spectra of **21**.

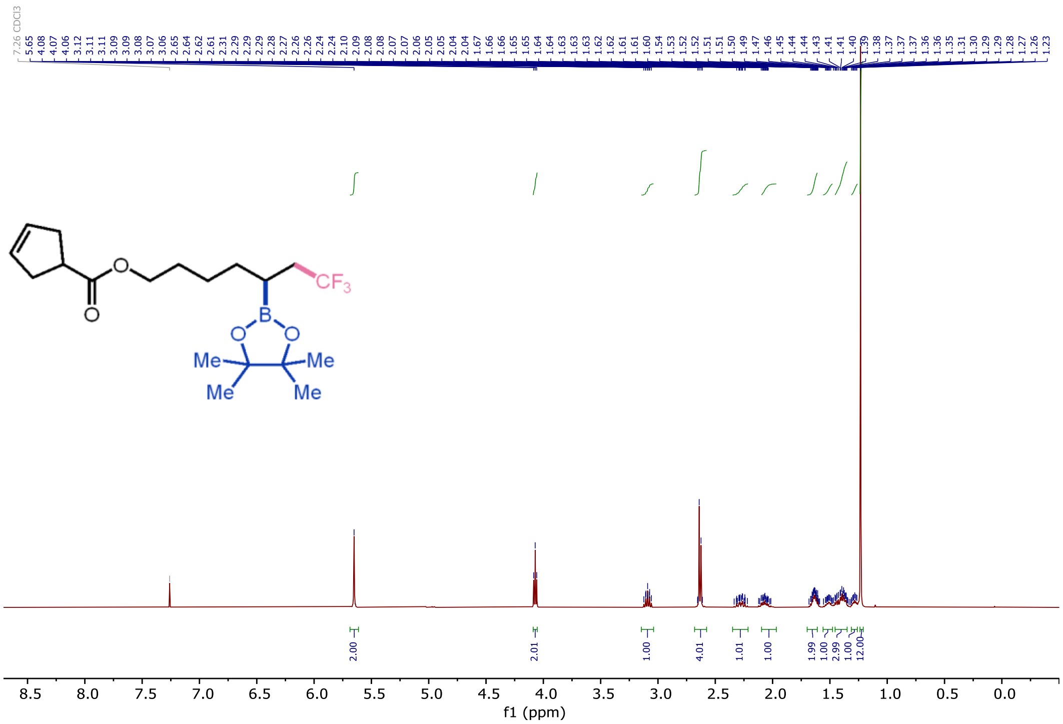

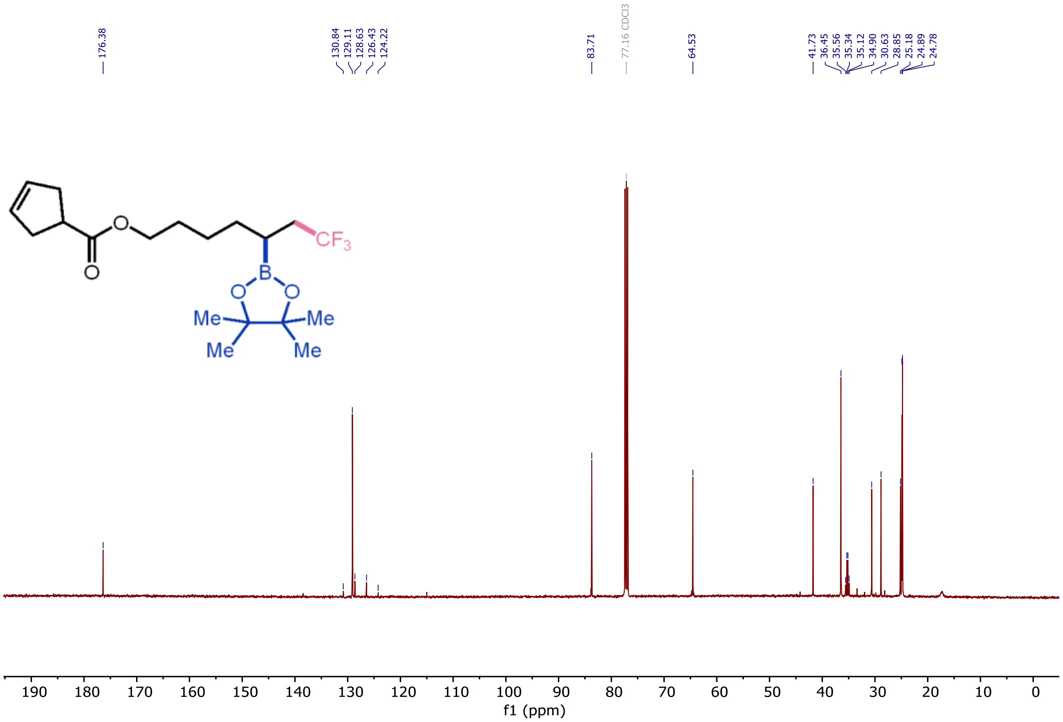

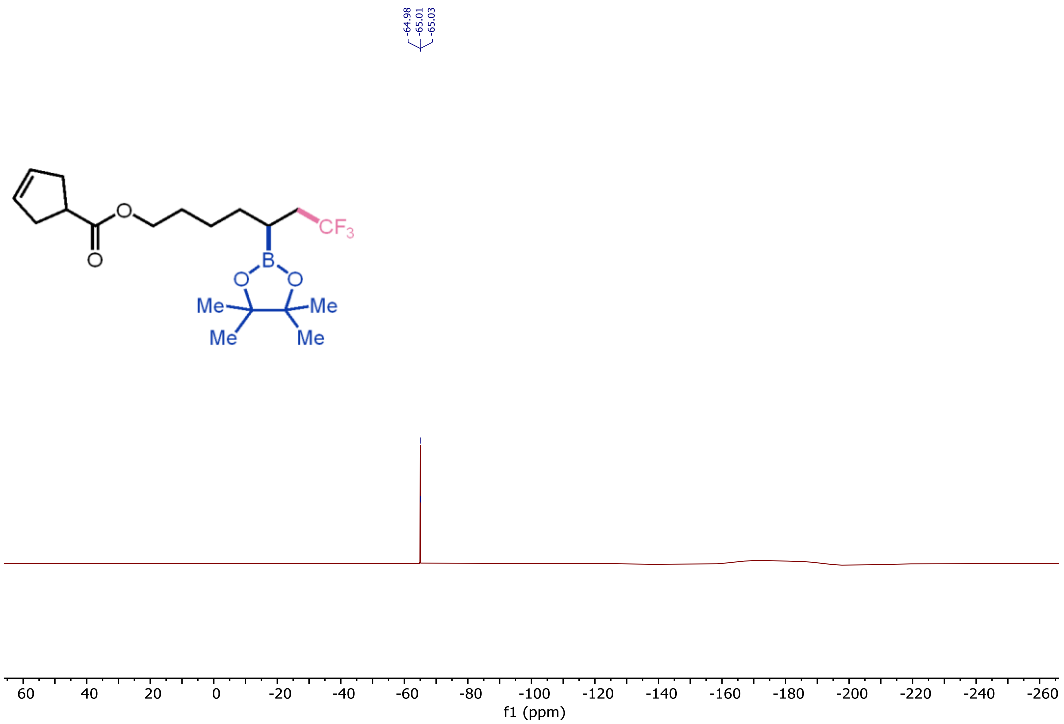

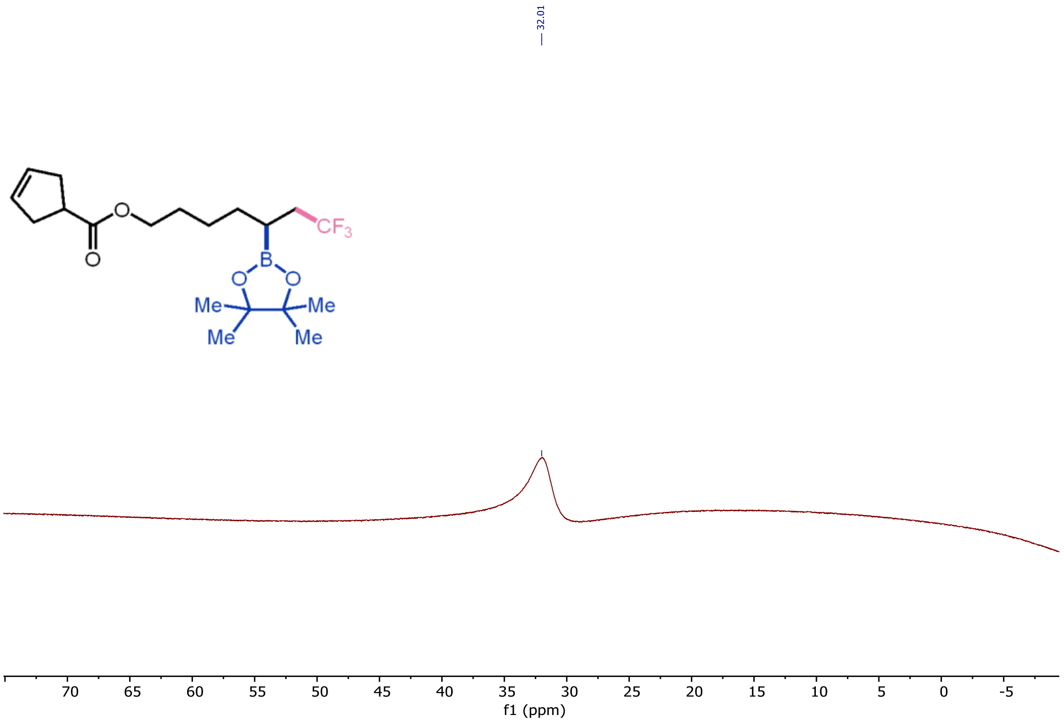

Figure S78. ^{1}H NMR (500 MHz, $CDCl_3$), ^{13}C NMR (101 MHz, $CDCl_3$), ^{19}F NMR (376 MHz, $CDCl_3$) and ^{11}B NMR (160 MHz, $CDCl_3$) spectra of **22**.

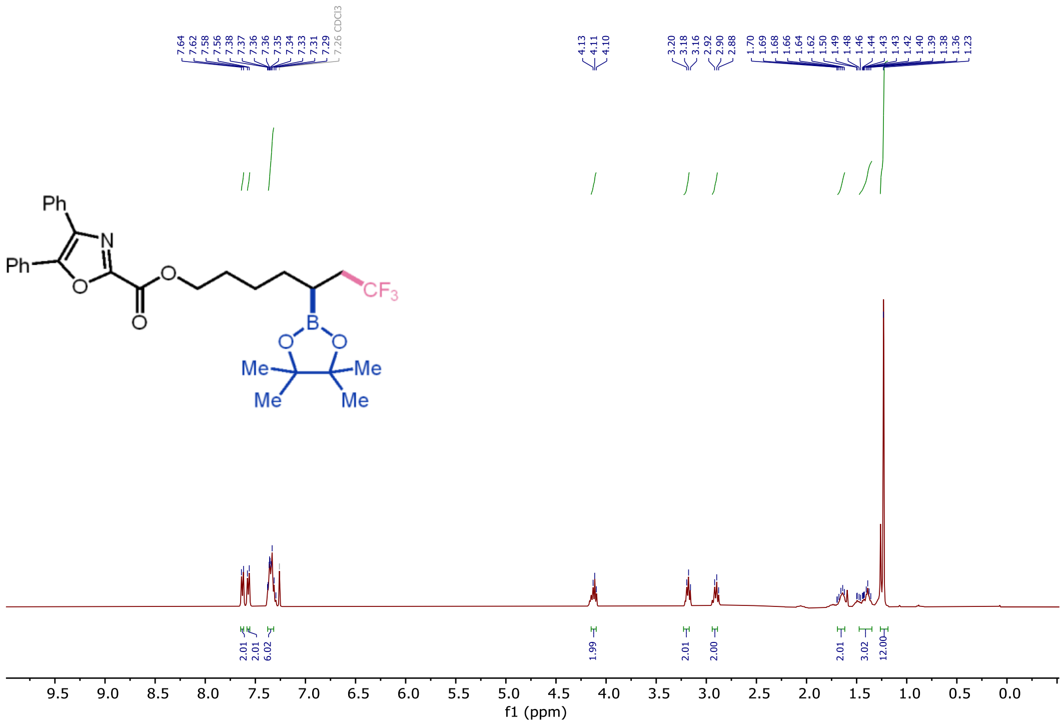
7.64
7.62
7.58
7.56
7.38
7.37
7.36
7.36
7.35
7.34
7.33
7.31
7.29
7.26 CDCl3
4.13
4.11
4.10
3.20
3.18
3.16
2.92
2.90
2.88
1.70
1.69
1.68
1.66
1.64
1.62
1.50
1.49
1.48
1.46
1.44
1.43
1.43
1.42
1.40
1.39
1.38
1.36
1.23
Ph
Ph
N
O
O
O
CF3
B
O
O
Me
Me
Me
Me
2.01
2.01
6.02
1.99
2.01
2.00
2.01
3.02
12.00
9.5
9.0
8.5
8.0
7.5
7.0
6.5
6.0
5.5
5.0
4.5
4.0
3.5
3.0
2.5
2.0
1.5
1.0
0.5
0.0
f1 (ppm)

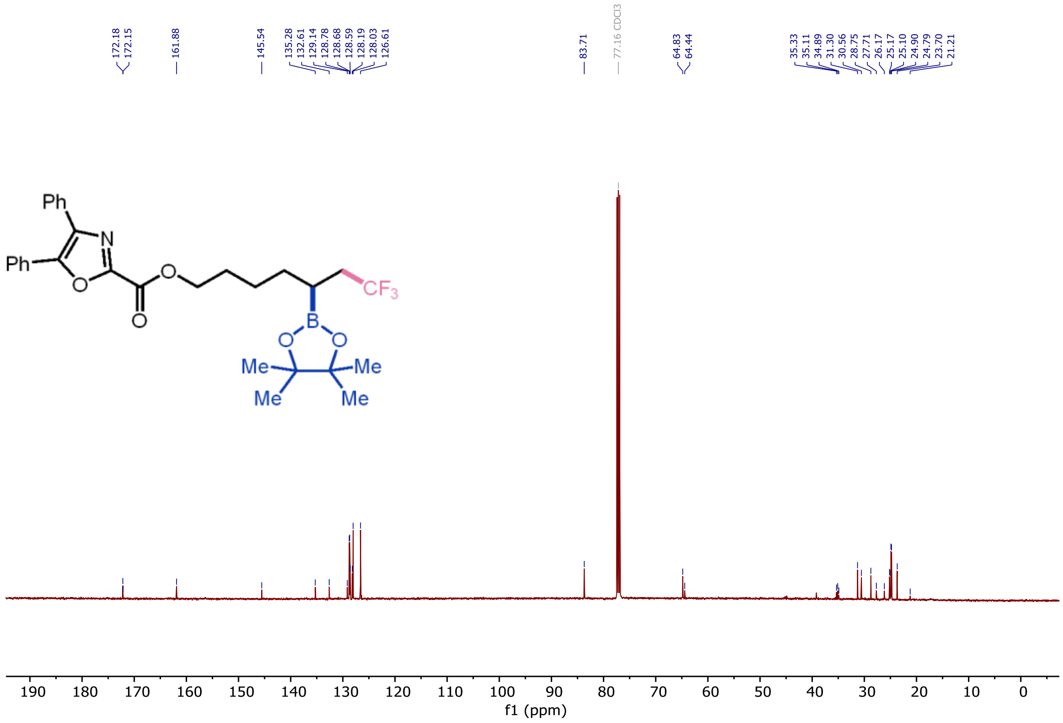
172.18
172.15
161.88
145.54
135.28
132.61
129.14
128.78
128.68
128.59
128.19
128.03
126.61
83.71
77.16 CDCl3
64.83
64.44
35.33
35.11
34.89
31.30
30.56
28.75
27.71
26.17
25.17
25.10
24.90
24.79
23.70
21.21
Ph
Ph
N
O
O
O
CF3
B
O
O
Me
Me
Me
Me
190
180
170
160
150
140
130
120
110
100
90
80
70
60
50
40
30
20
10
0
f1 (ppm)

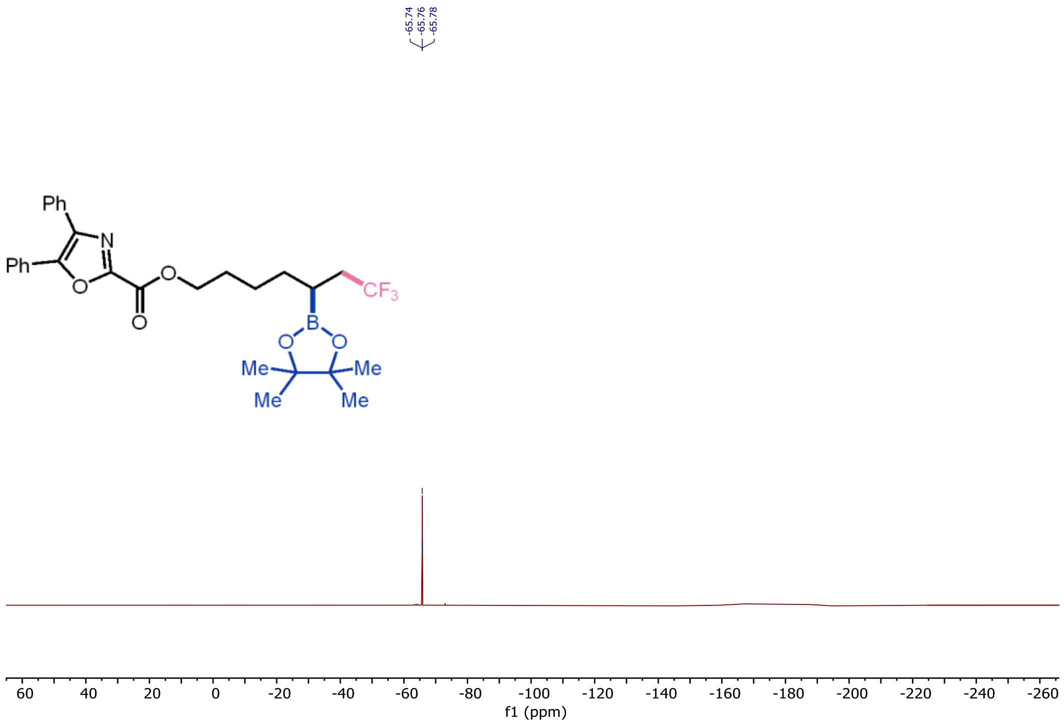

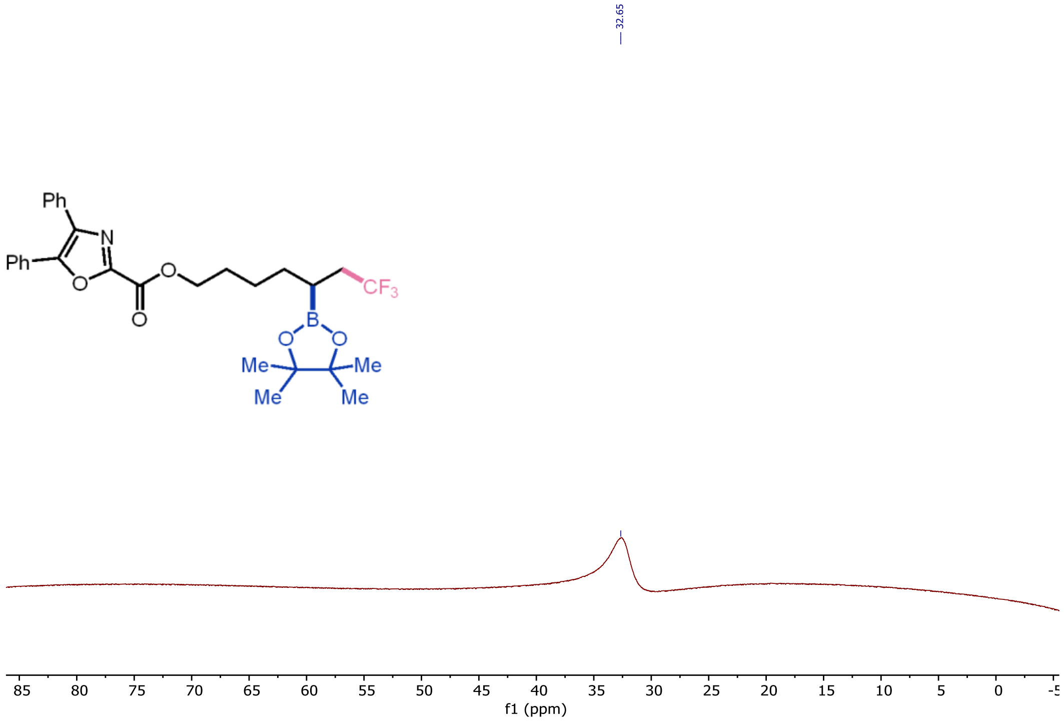

Figure S79. ^{1}H NMR (500 MHz, $CDCl_3$), ^{13}C NMR (101 MHz, $CDCl_3$), ^{19}F NMR (376 MHz, $CDCl_3$) and ^{11}B NMR (160 MHz, $CDCl_3$) spectra of **23**.

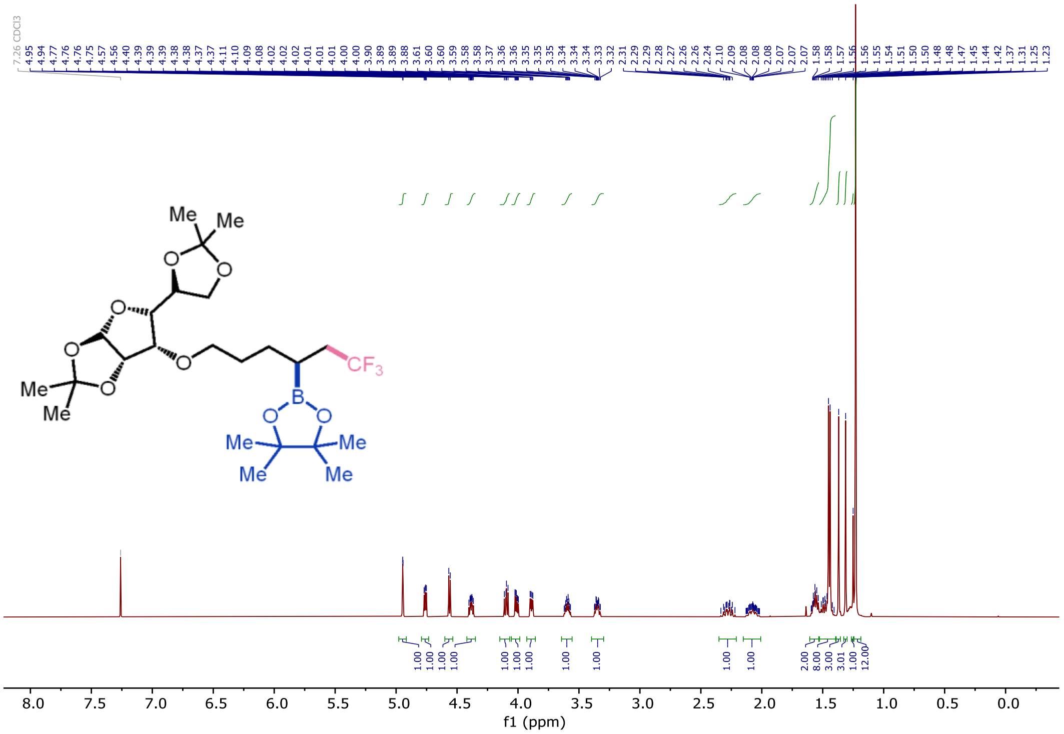

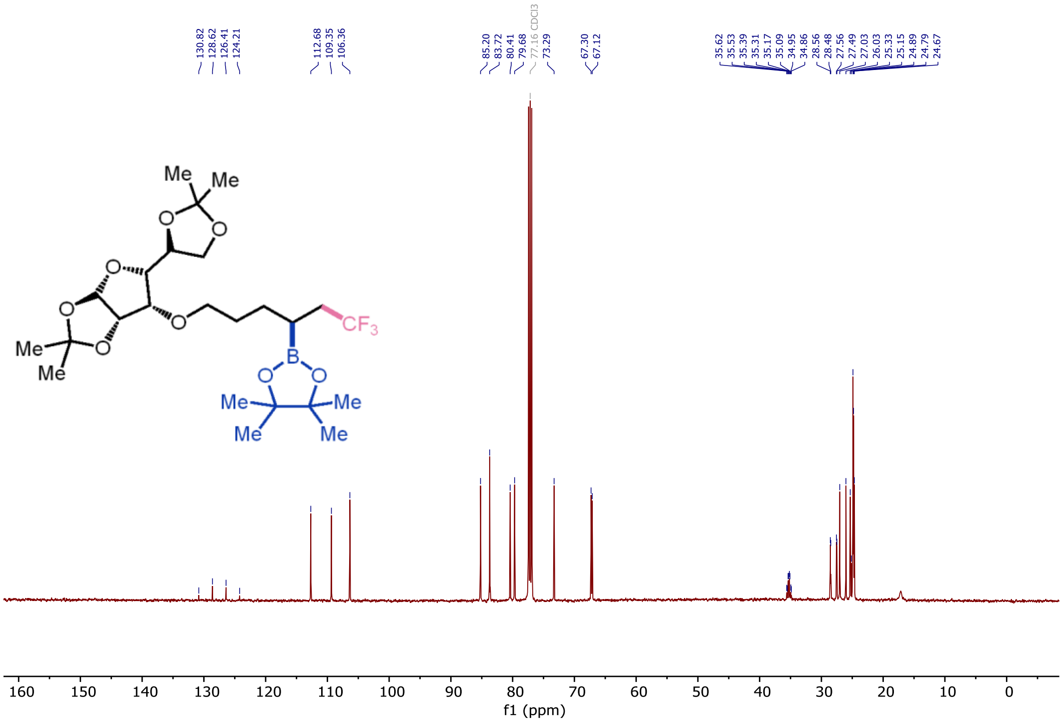

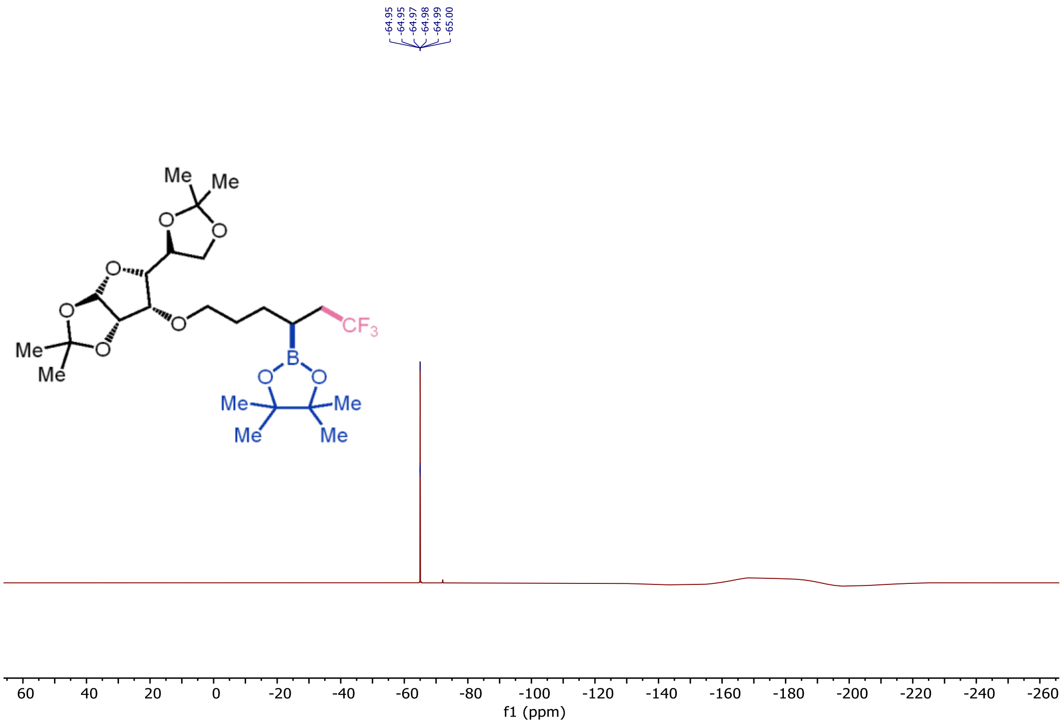

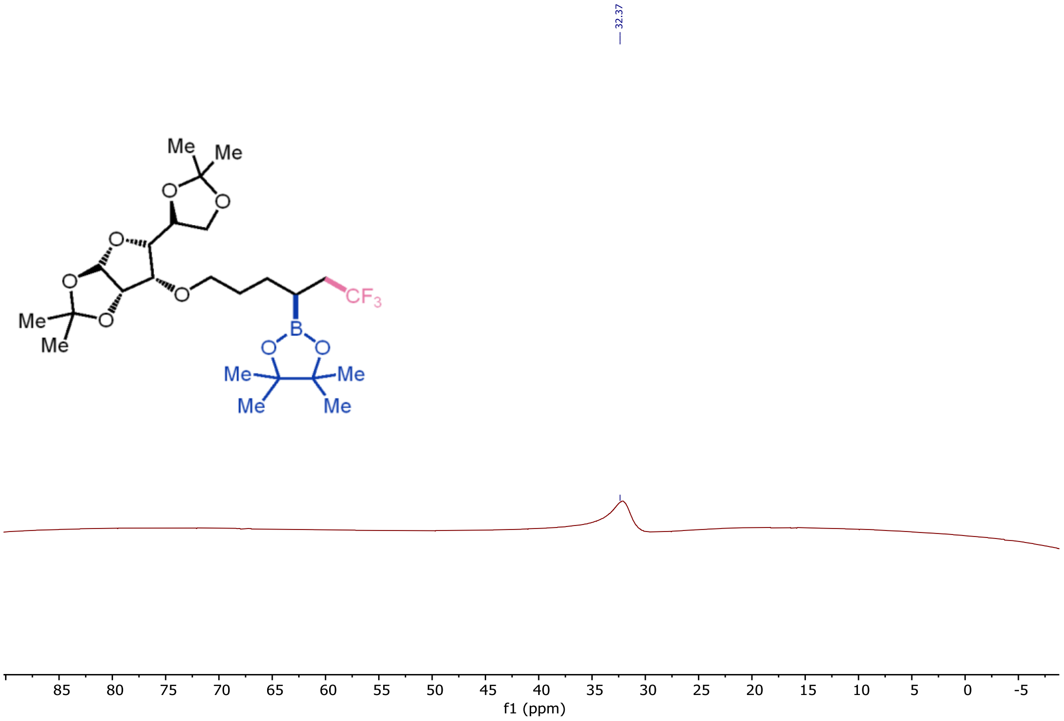

Figure S80. ^{1}H NMR (500 MHz, $CDCl_3$), ^{13}C NMR (101 MHz, $CDCl_3$), ^{19}F NMR (376 MHz, $CDCl_3$) and ^{11}B NMR (160 MHz, $CDCl_3$) spectra of **24**.

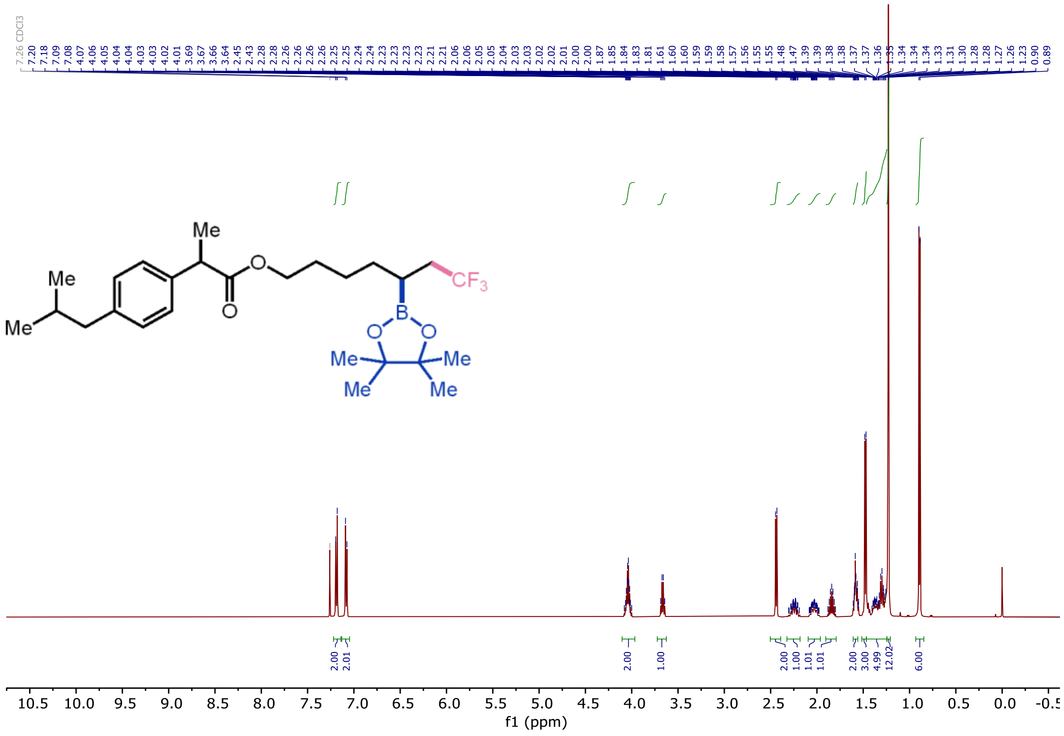

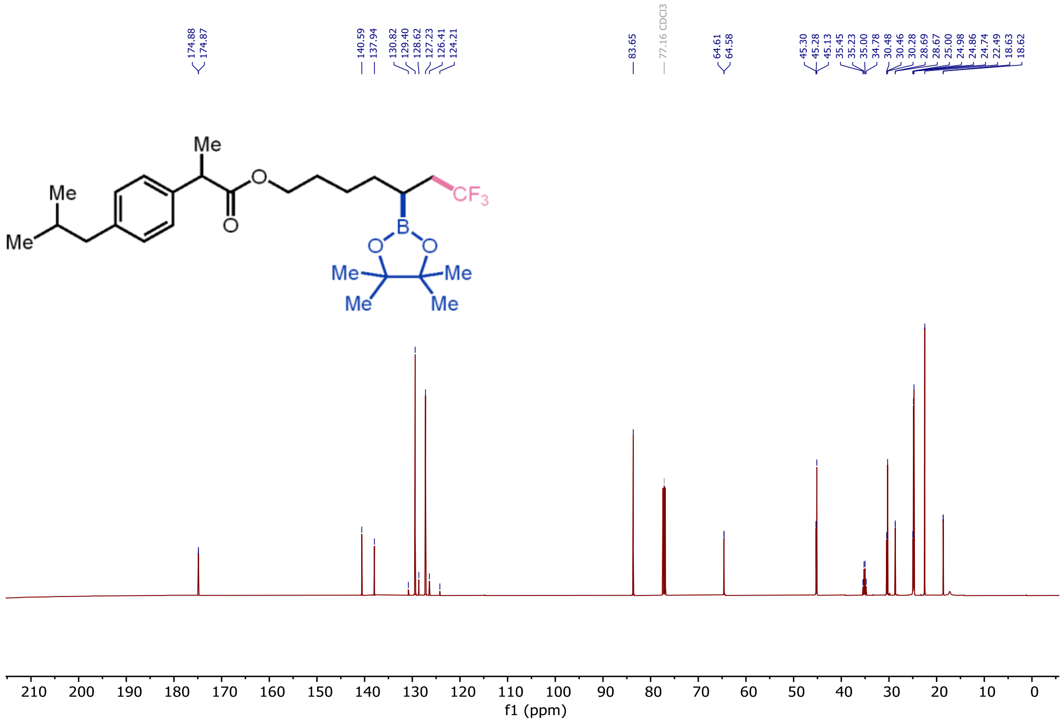

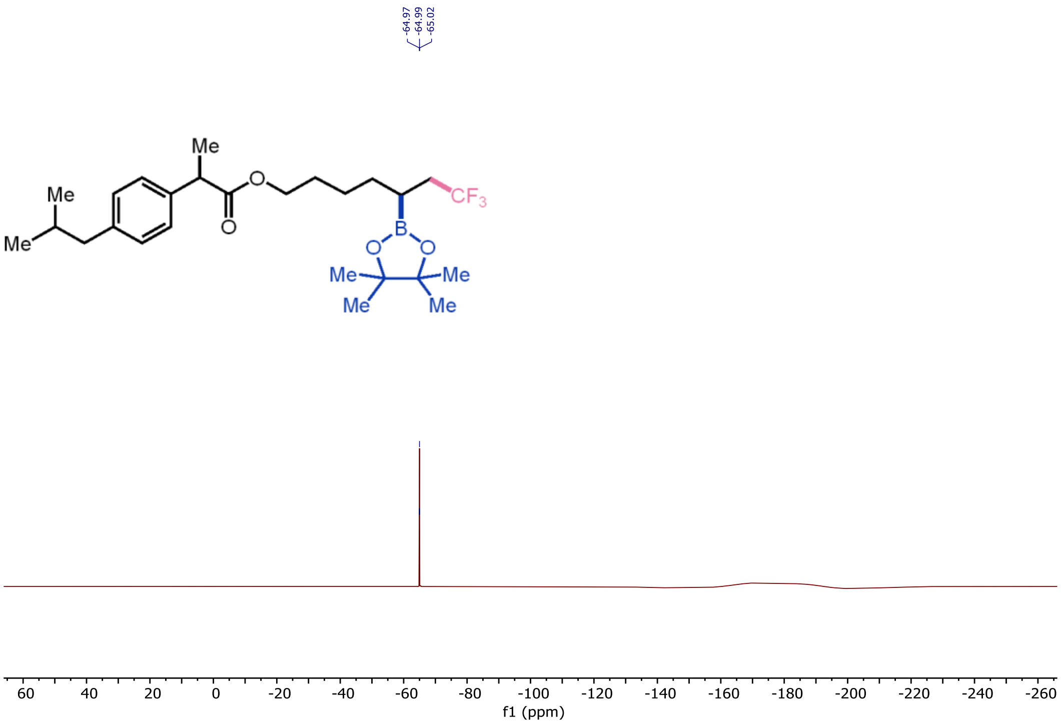

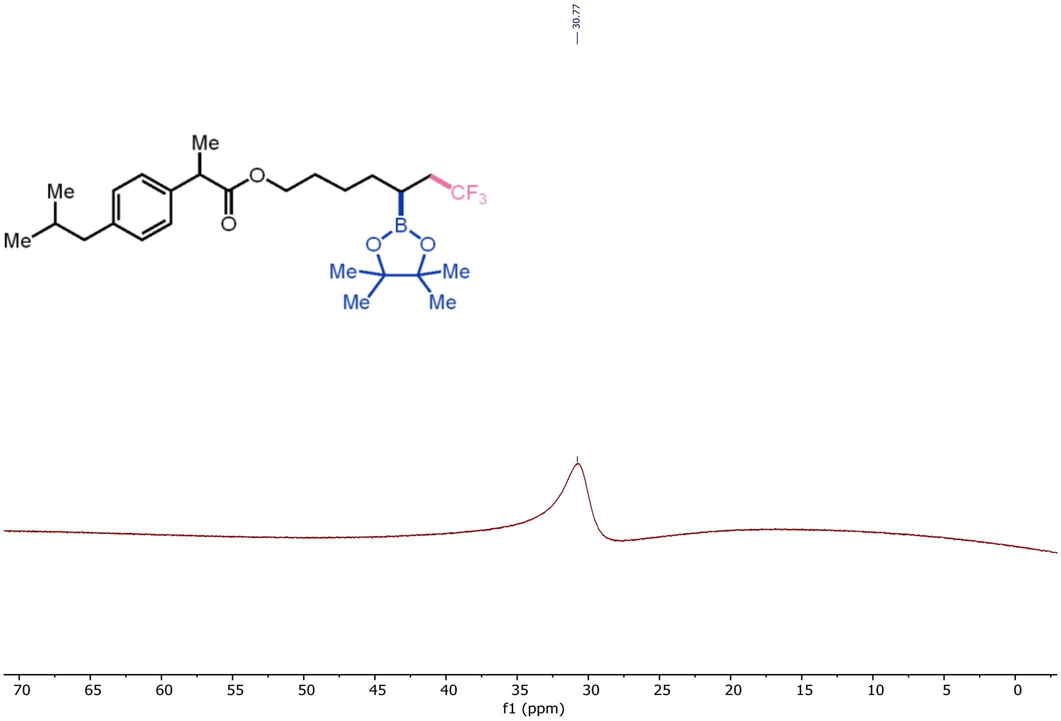

Figure S81. ^{1}H NMR (500 MHz, $CDCl_3$), ^{13}C NMR (101 MHz, $CDCl_3$), ^{19}F NMR (376 MHz, $CDCl_3$) and ^{11}B NMR (160 MHz, $CDCl_3$) spectra of **25**.

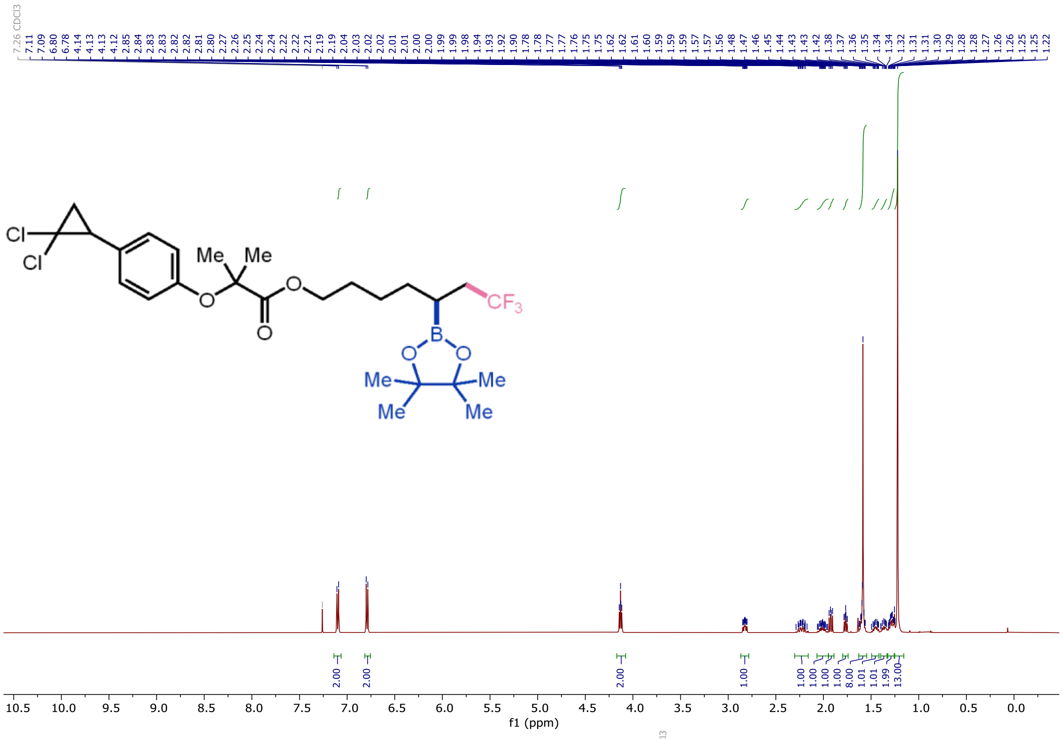

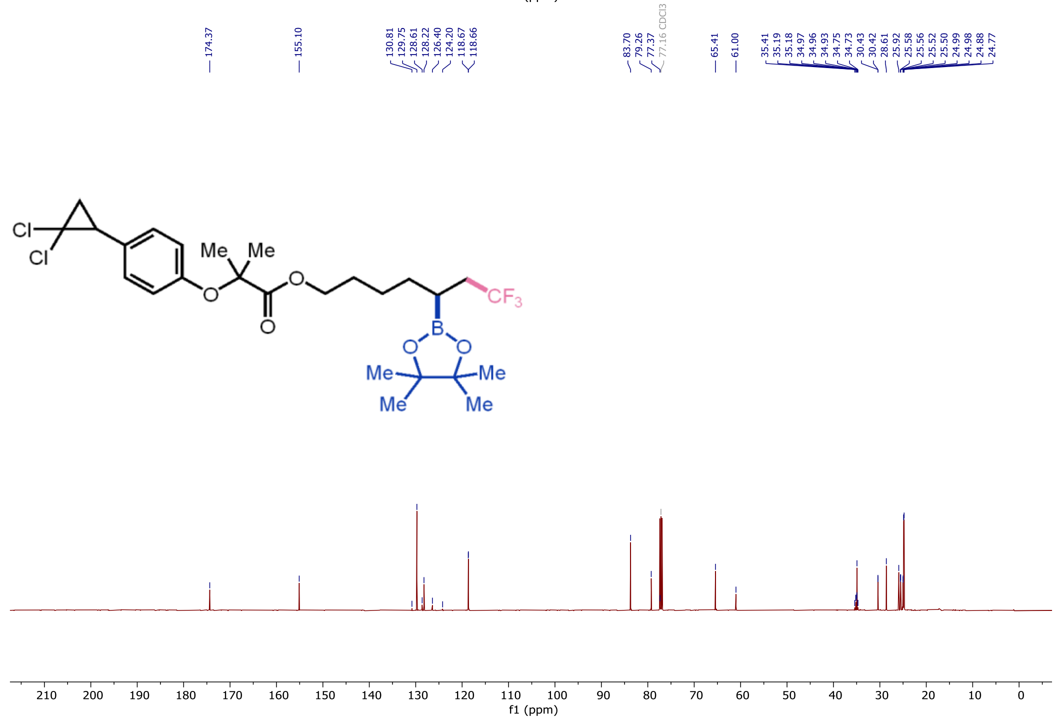

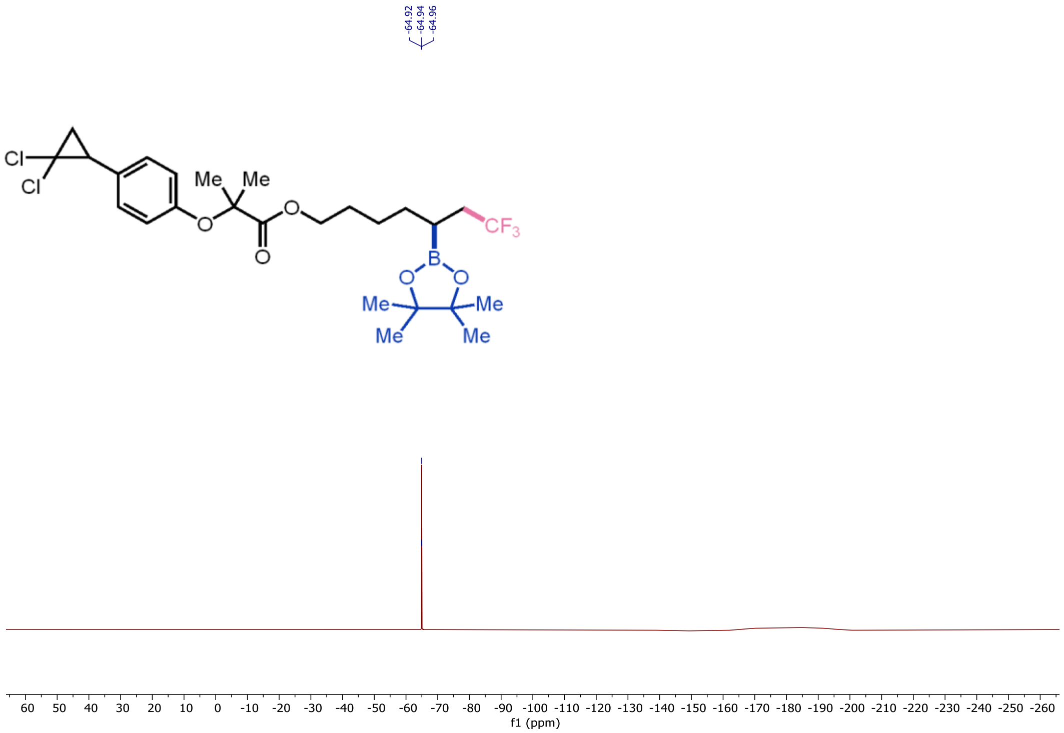

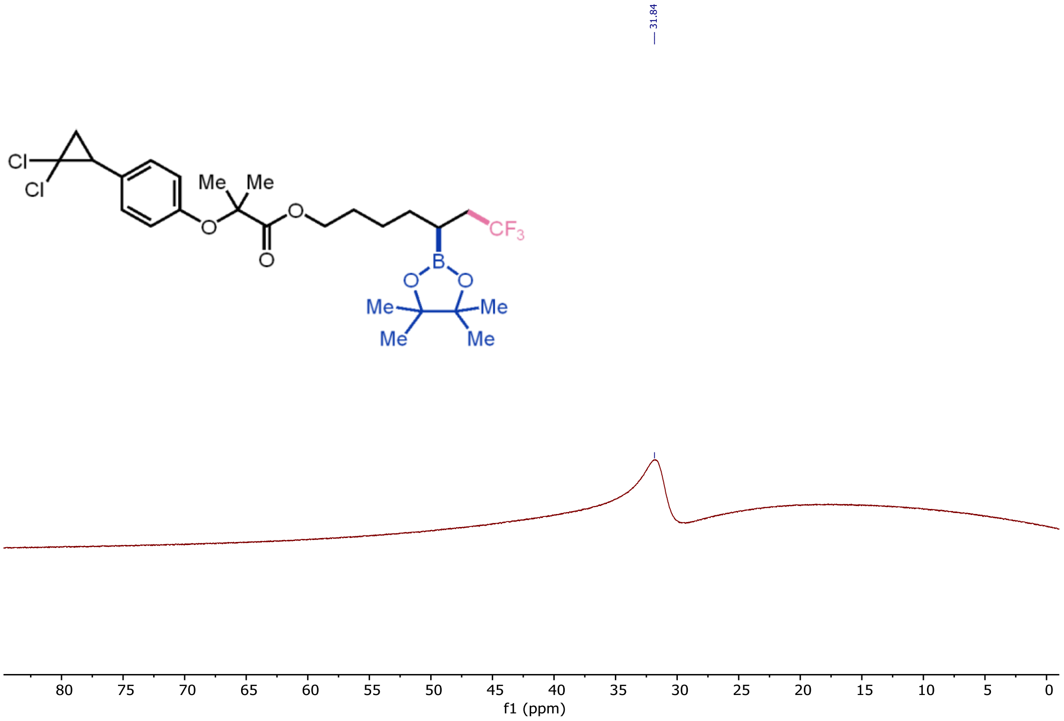

Figure S82. ^{1}H NMR (500 MHz, $CDCl_3$), ^{13}C NMR (101 MHz, $CDCl_3$), ^{19}F NMR (376 MHz, $CDCl_3$) and ^{11}B NMR (160 MHz, $CDCl_3$) spectra of **26**.

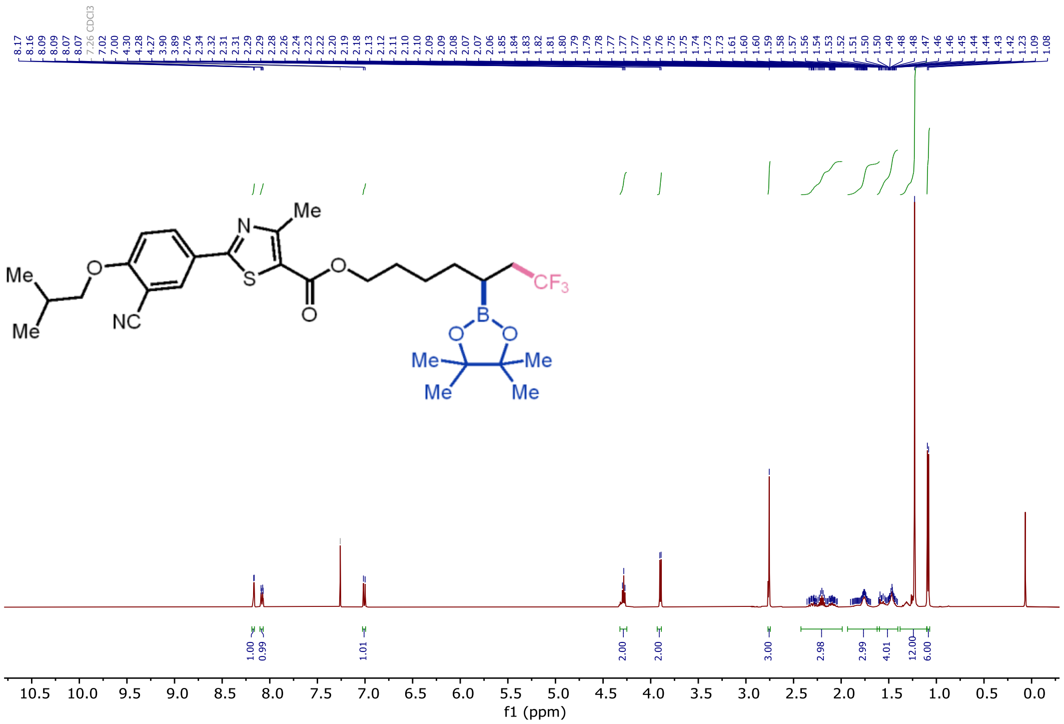

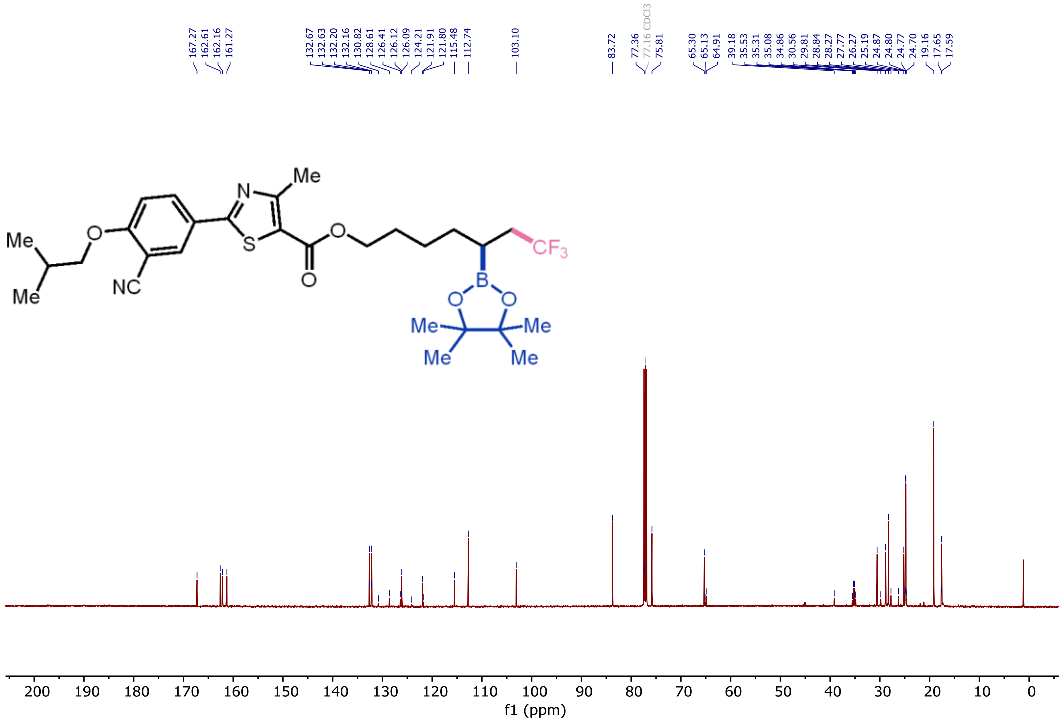

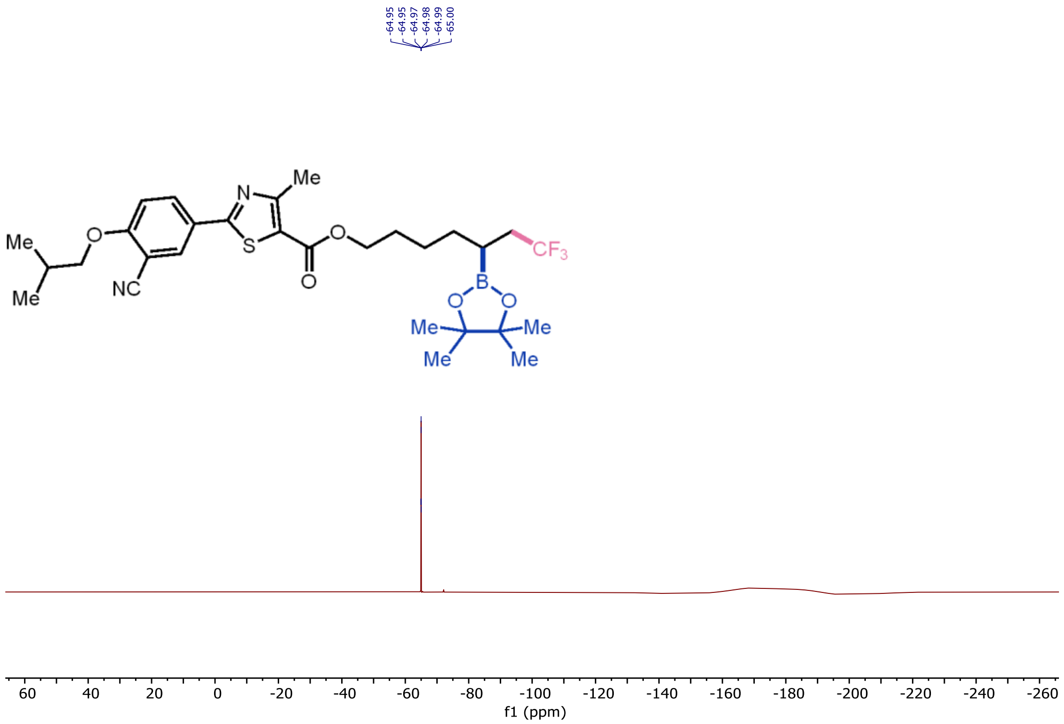

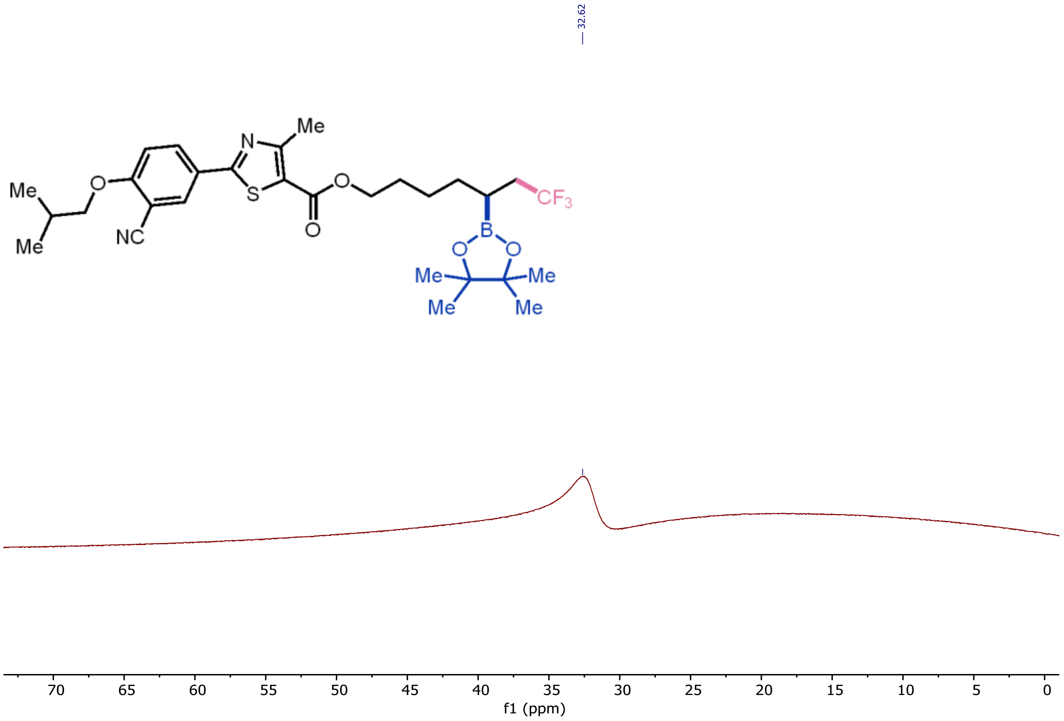

Figure S83. ^{1}H NMR (500 MHz, $CDCl_3$), ^{13}C NMR (101 MHz, $CDCl_3$), ^{19}F NMR (376 MHz, $CDCl_3$) and ^{11}B NMR (160 MHz, $CDCl_3$) spectra of **27**.

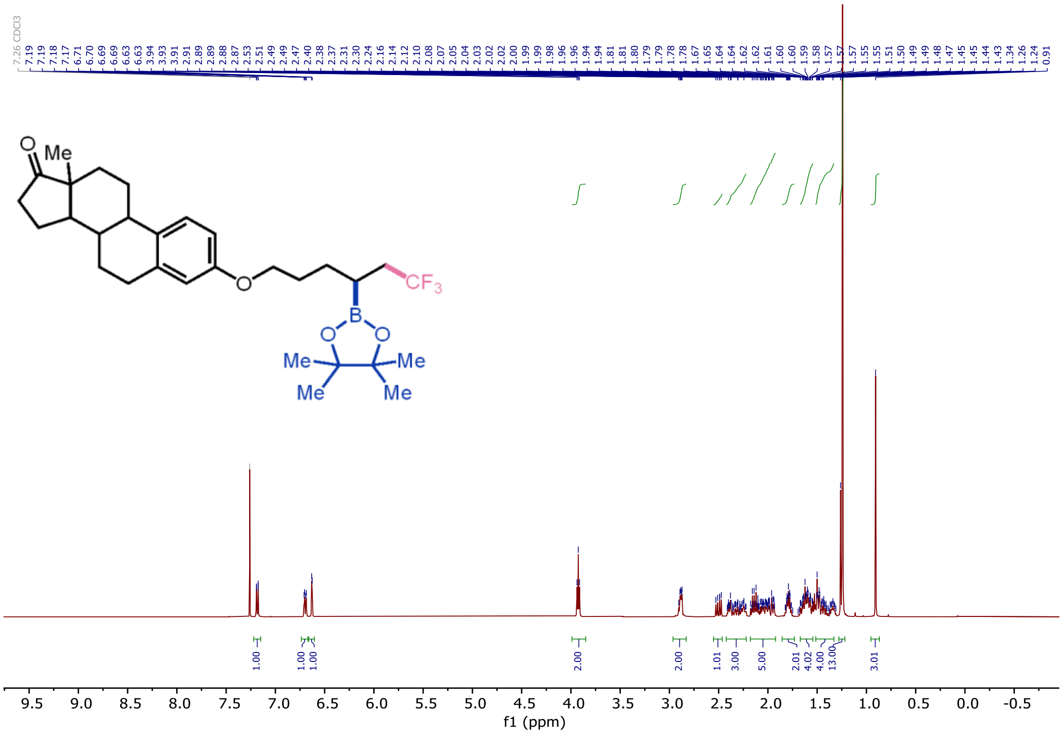

7.26 CDCl3
Me
O
CF3
B
Me
Me
Me
Me
1.00
1.00
1.00
2.00
2.00
1.01
3.00
5.00
2.01
4.02
4.00
13.00
3.01
f1 (ppm)

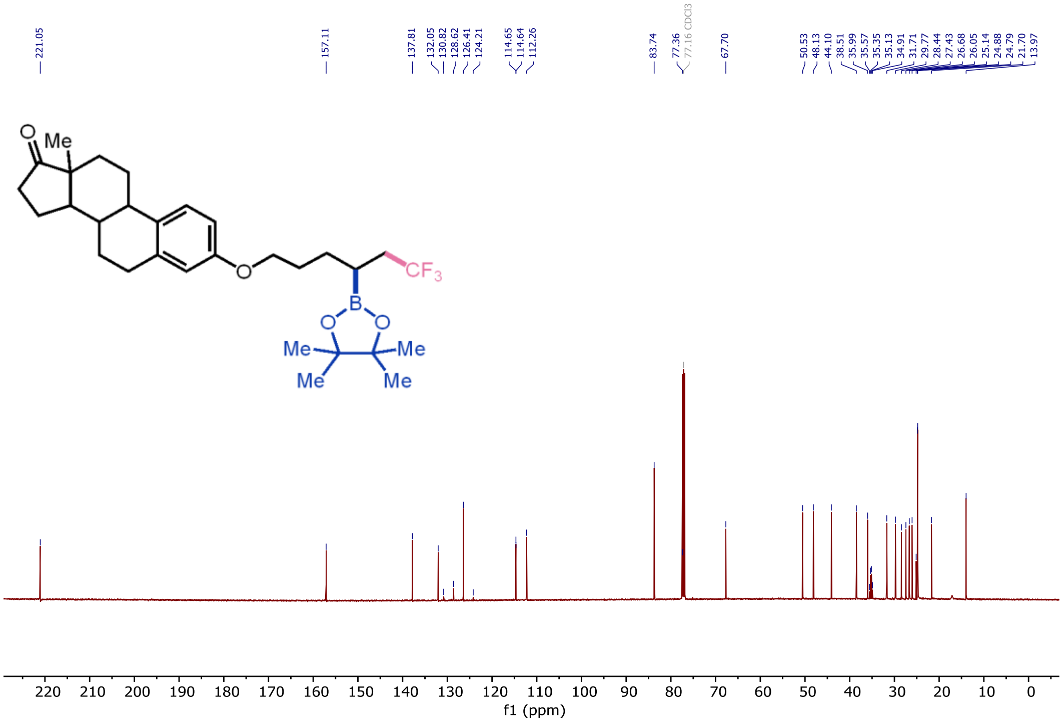

221.05
157.11
137.81
132.05
130.82
128.62
126.41
124.21
114.65
114.64
112.26
83.74
77.36
77.16 CDCl3
67.70
50.53
48.13
44.10
38.51
35.99
35.57
35.35
35.13
34.91
31.71
29.77
28.44
27.43
26.68
26.05
25.14
24.88
24.79
21.70
13.97
Me
O
CF3
B
Me
Me
Me
Me
f1 (ppm)

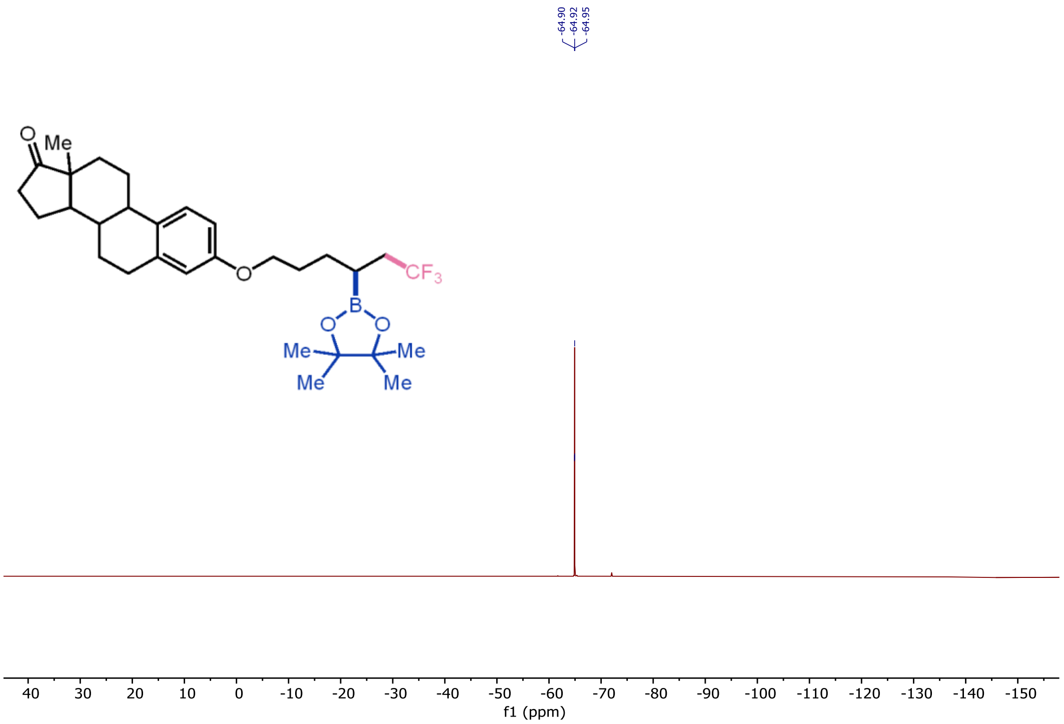

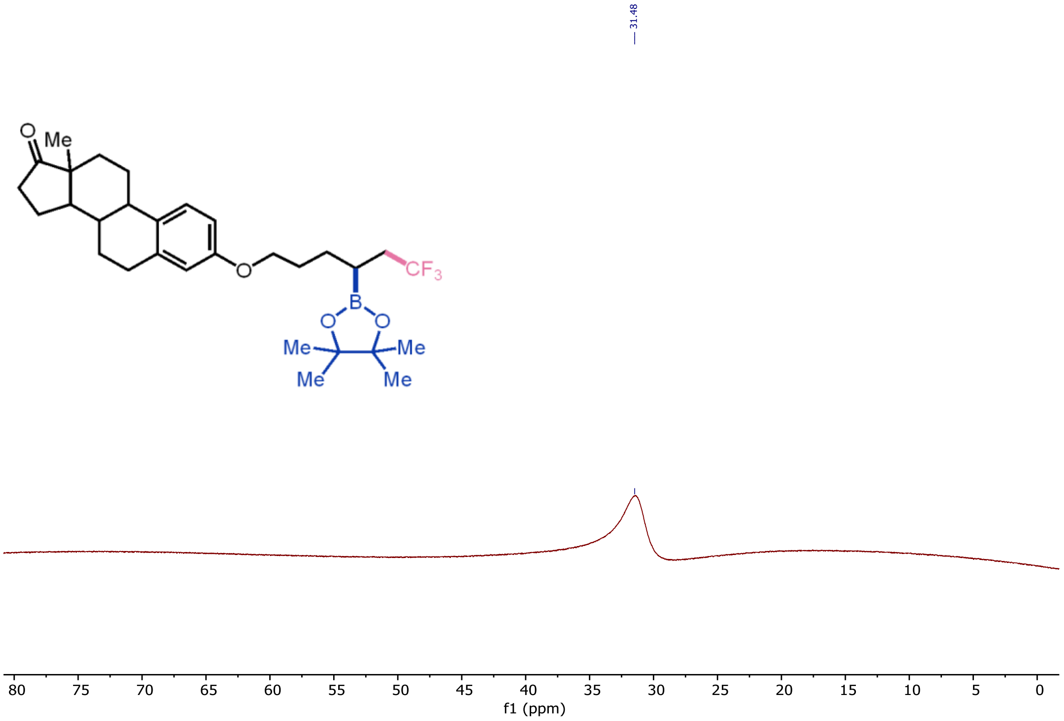

Figure S84. ^{1}H NMR (500 MHz, $CDCl_3$), ^{13}C NMR (101 MHz, $CDCl_3$), ^{19}F NMR (376 MHz, $CDCl_3$) and ^{11}B NMR (160 MHz, $CDCl_3$) spectra of **28**.

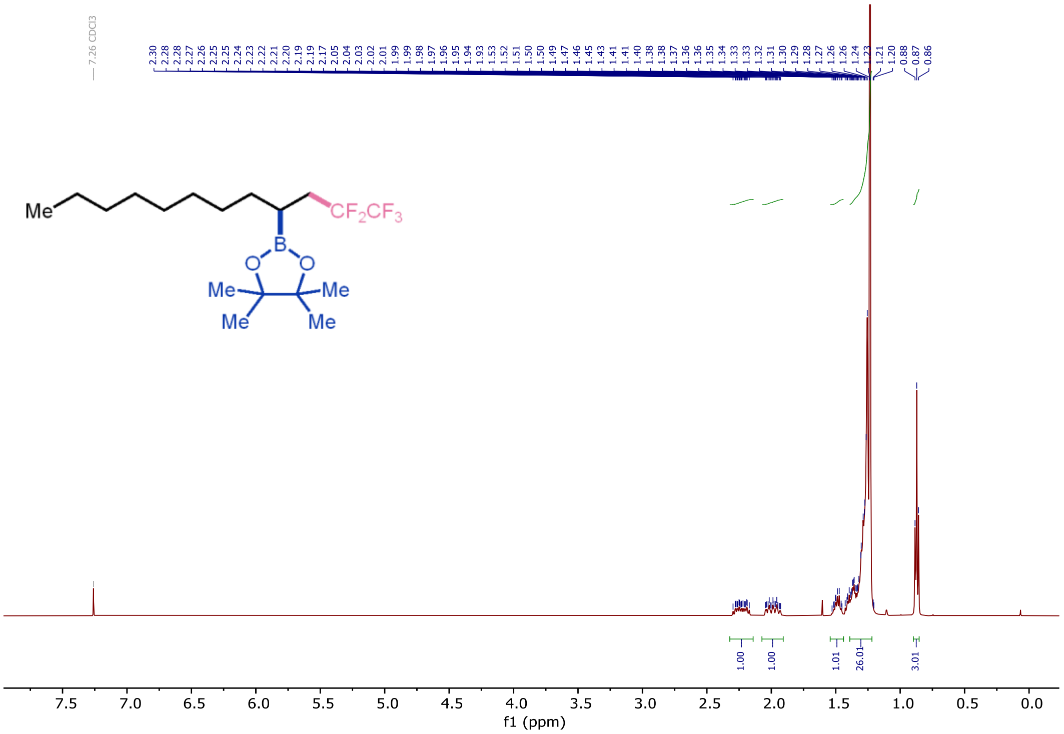

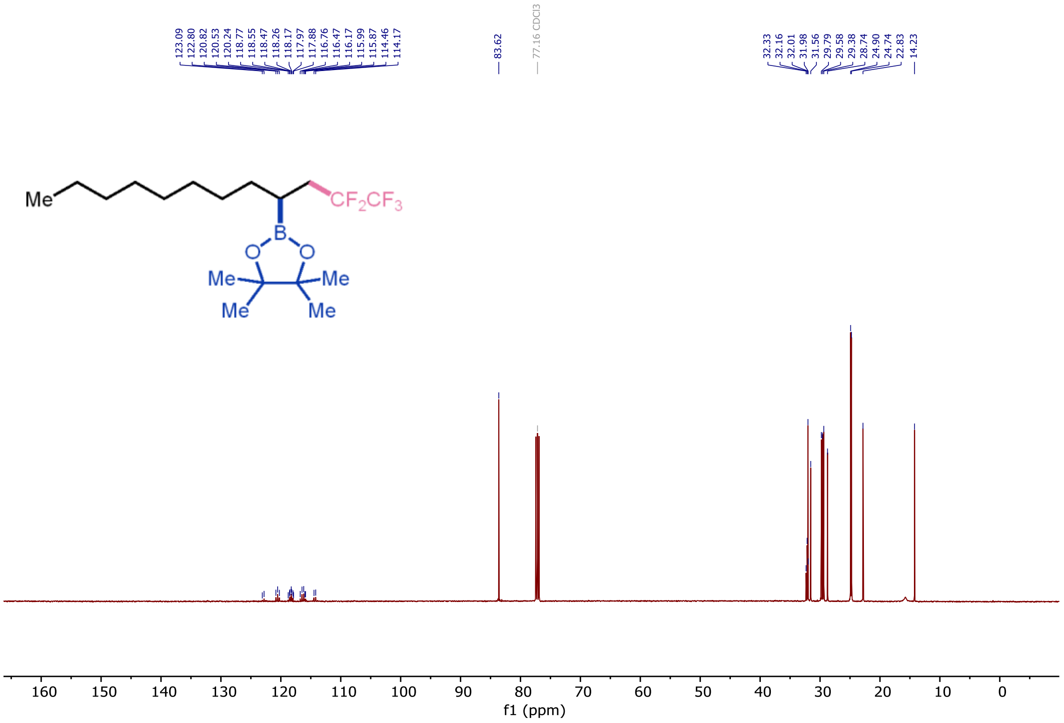

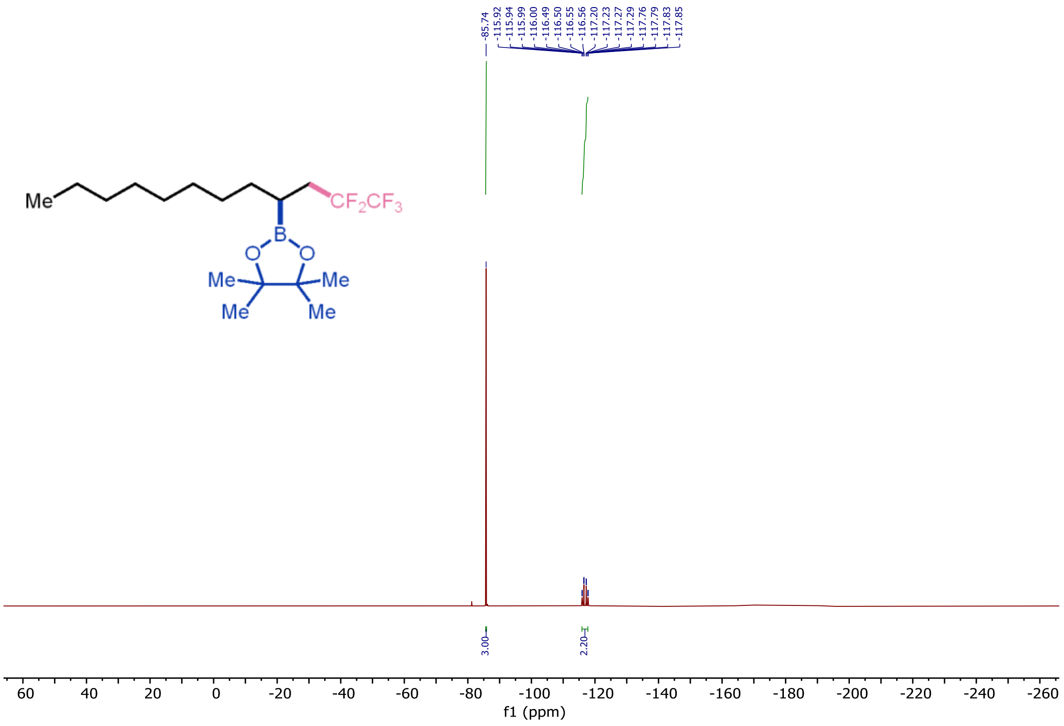

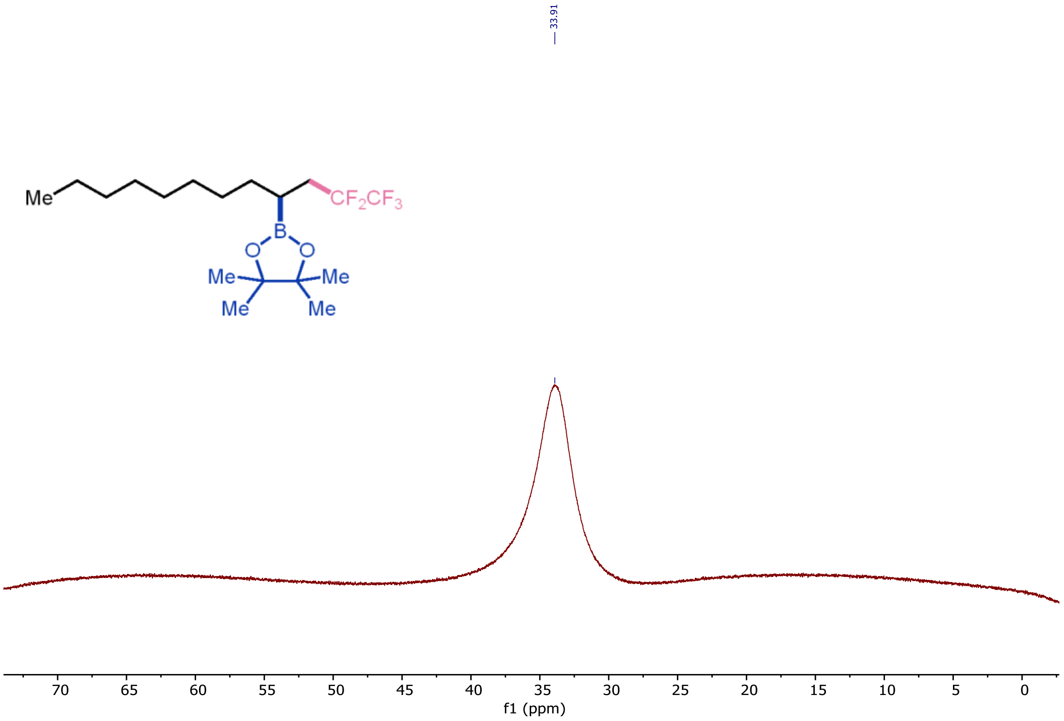

Figure S85. ^{1}H NMR (500 MHz, $CDCl_3$), ^{13}C NMR (101 MHz, $CDCl_3$), ^{19}F NMR (376 MHz, $CDCl_3$) and ^{11}B NMR (160 MHz, $CDCl_3$) spectra of **29**.

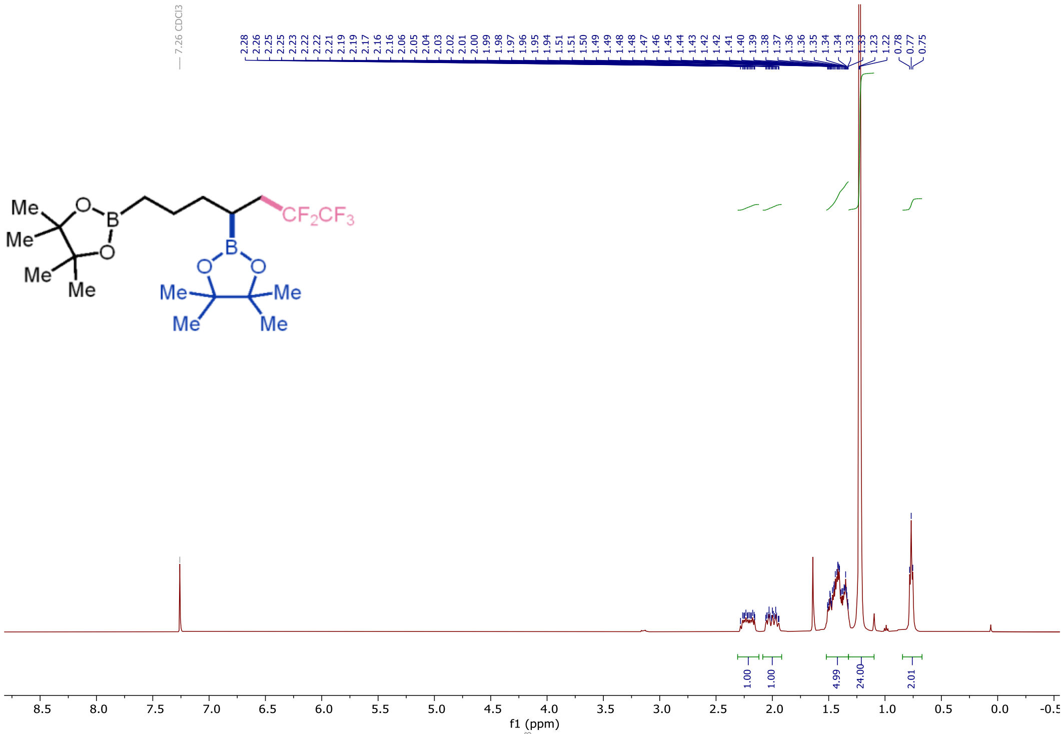
7.26 CDCl3
2.28 2.26 2.25 2.25 2.23 2.22 2.22 2.21 2.19 2.19 2.17 2.16 2.16 2.06 2.05 2.04 2.03 2.02 2.01 2.00 1.99 1.98 1.97 1.96 1.95 1.94 1.51 1.51 1.50 1.49 1.49 1.48 1.48 1.47 1.46 1.45 1.44 1.43 1.42 1.42 1.41 1.40 1.39 1.38 1.37 1.36 1.36 1.35 1.34 1.34 1.33 1.33 1.23 1.22 0.78 0.77 0.75
1.00 1.00 4.99 24.00 2.01
f1 (ppm)

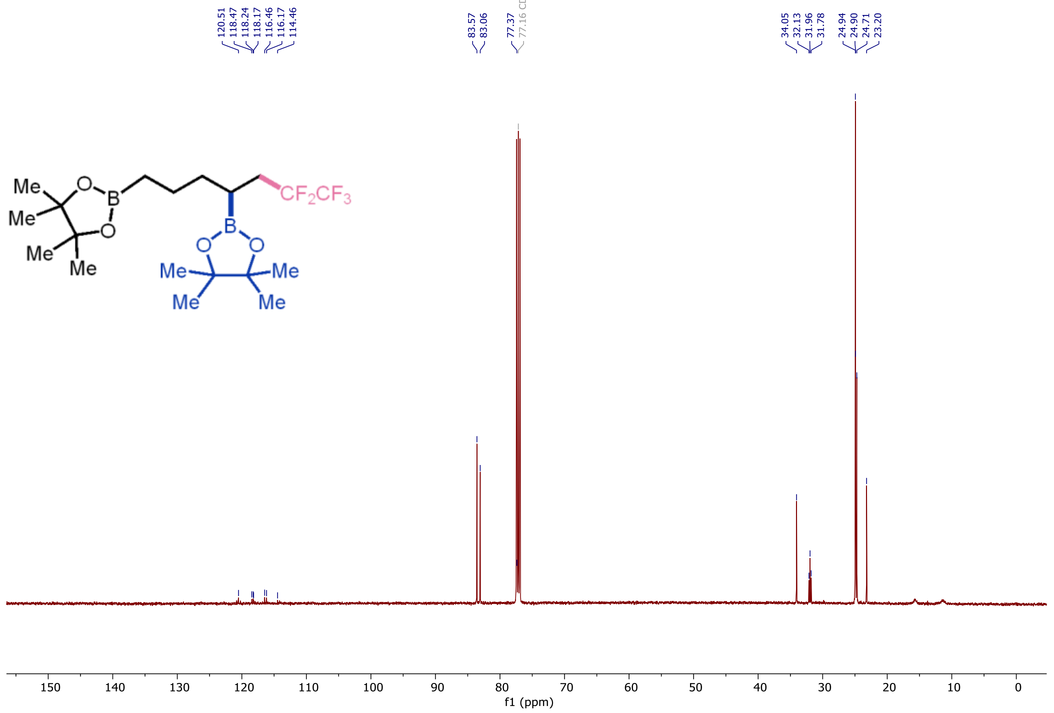
120.51 118.47 118.24 118.17 116.46 116.17 114.46
83.57 83.06
77.37 77.16 CDCl3
34.05 32.13 31.96 31.78
24.94 24.90 24.71 23.20
f1 (ppm)

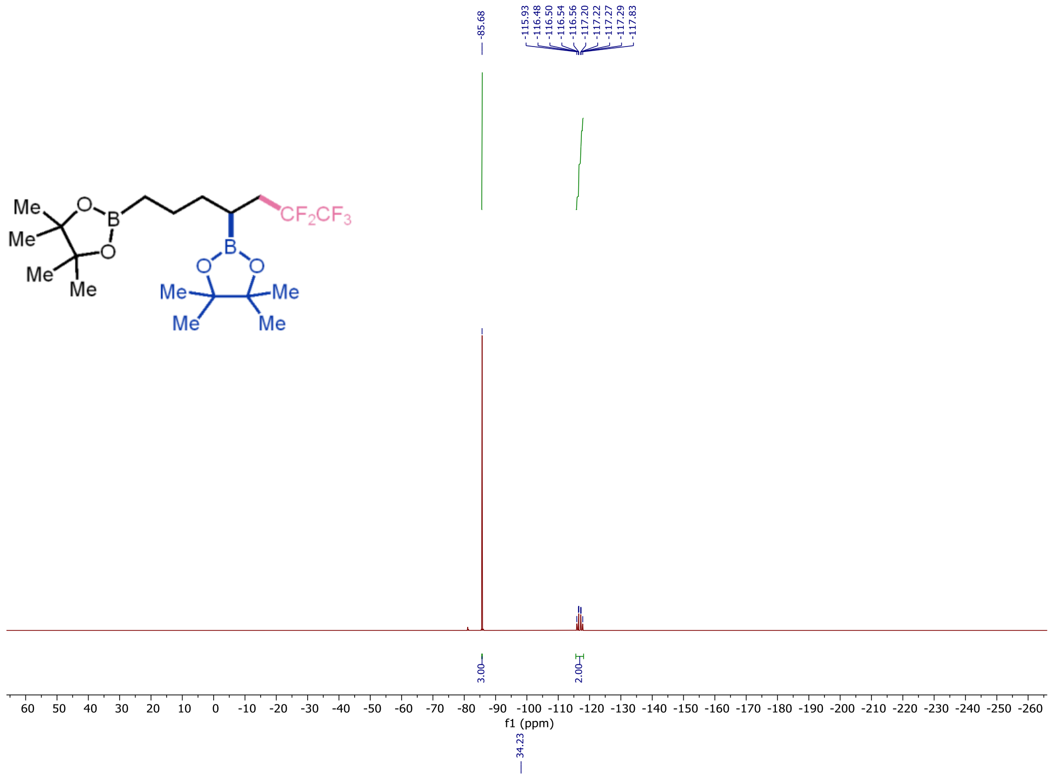

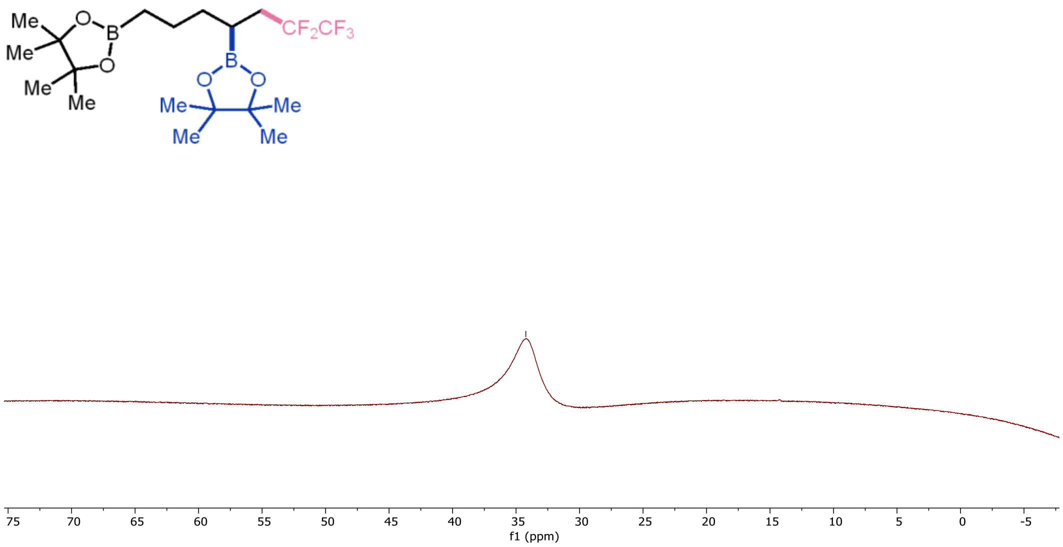

Figure S86. ^{1}H NMR (500 MHz, $CDCl_3$), ^{13}C NMR (101 MHz, $CDCl_3$), ^{19}F NMR (376 MHz, $CDCl_3$) and ^{11}B NMR (160 MHz, $CDCl_3$) spectra of **30**.

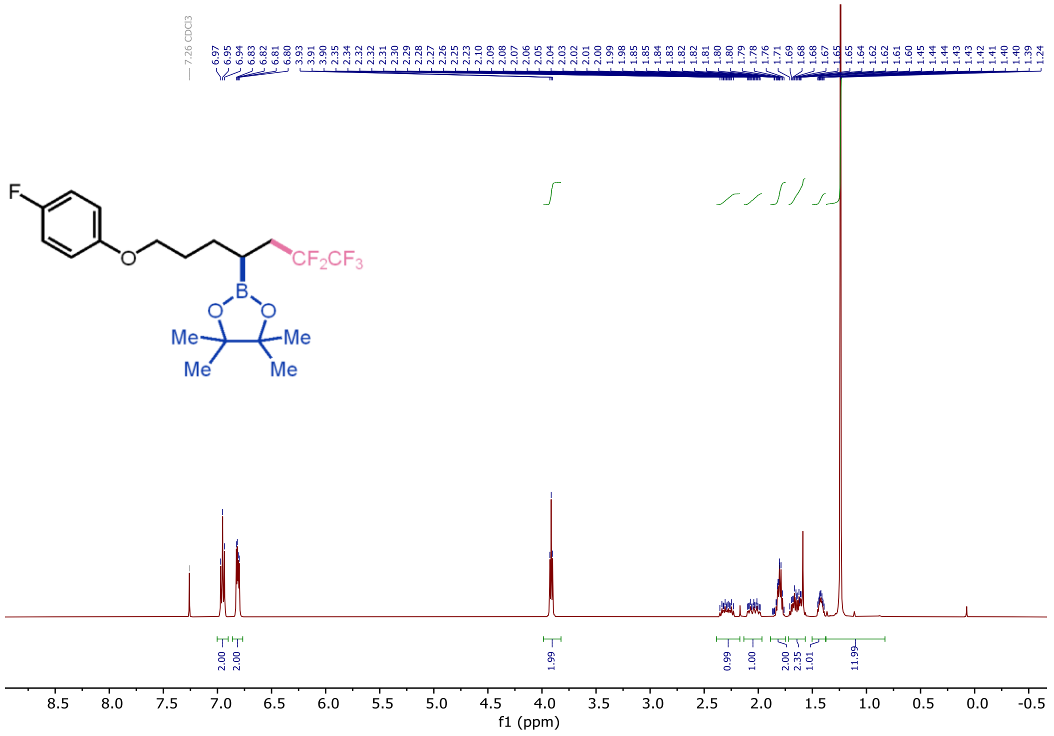

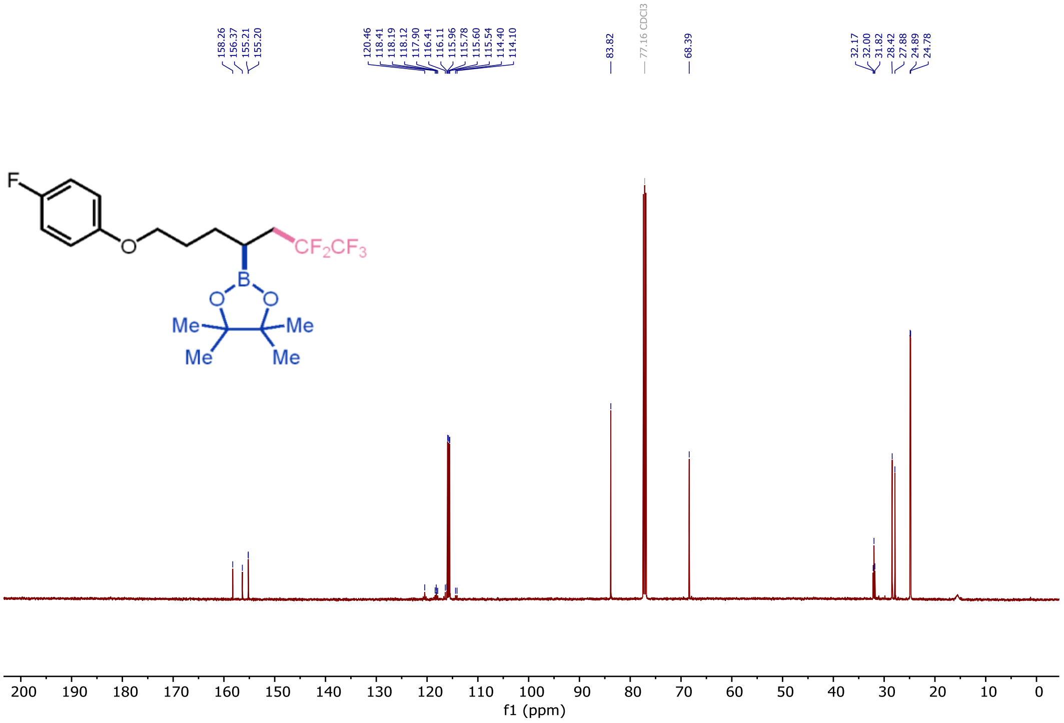

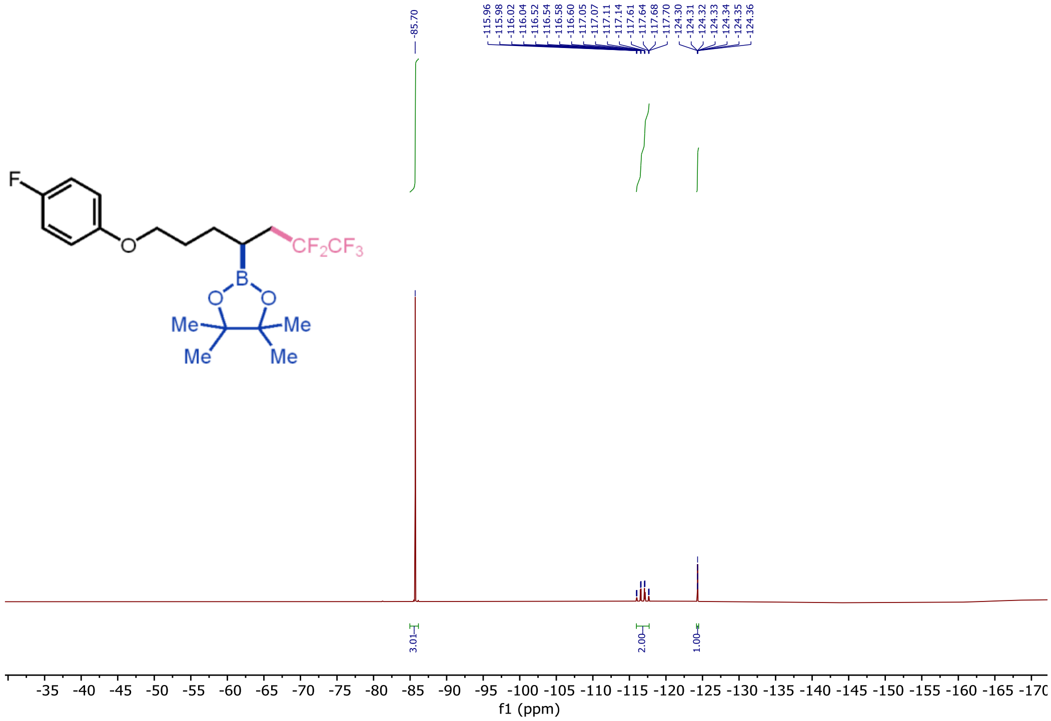

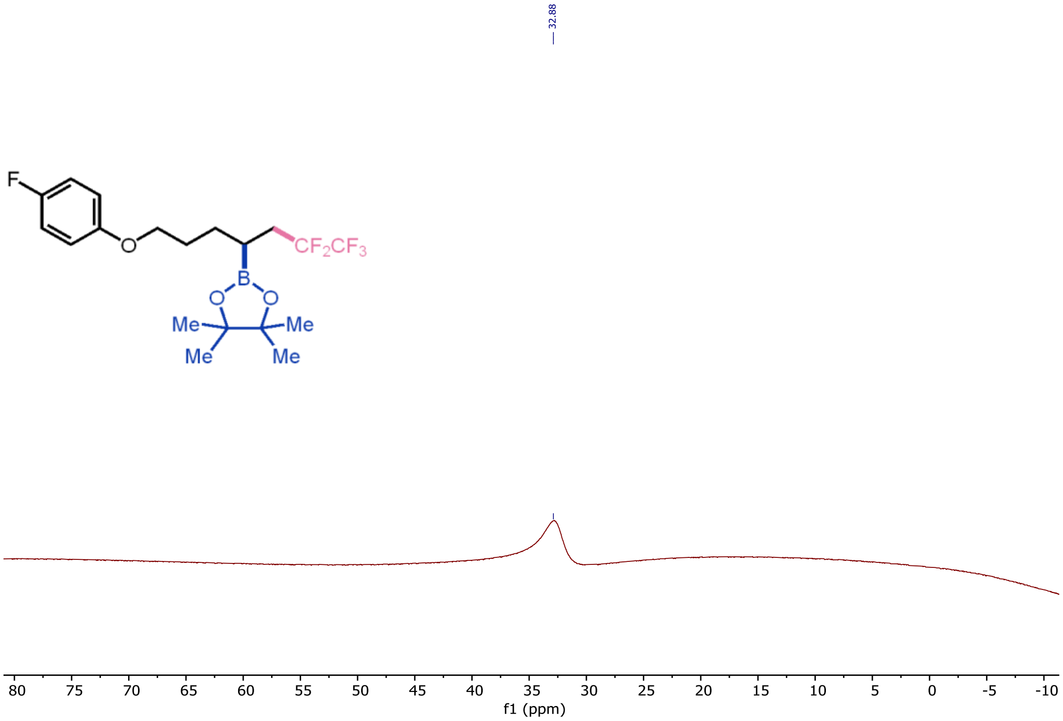

Figure S87. ^{1}H NMR (500 MHz, $CDCl_3$), ^{13}C NMR (101 MHz, $CDCl_3$), ^{19}F NMR (376 MHz, $CDCl_3$) and ^{11}B NMR (160 MHz, $CDCl_3$) spectra of **31**.

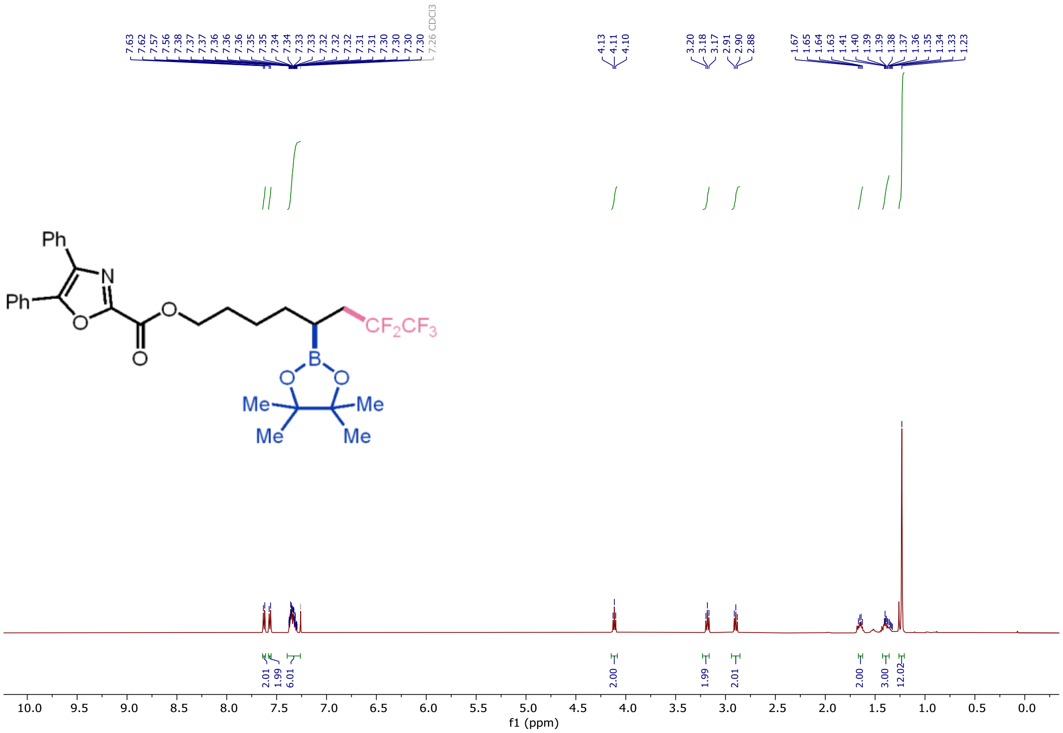

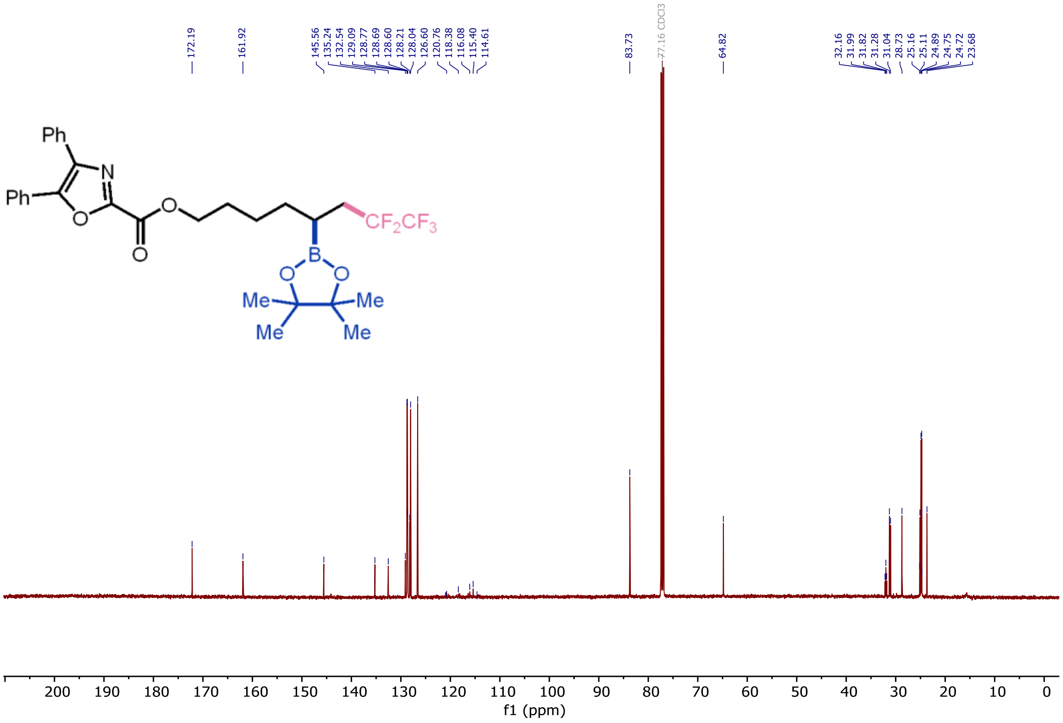

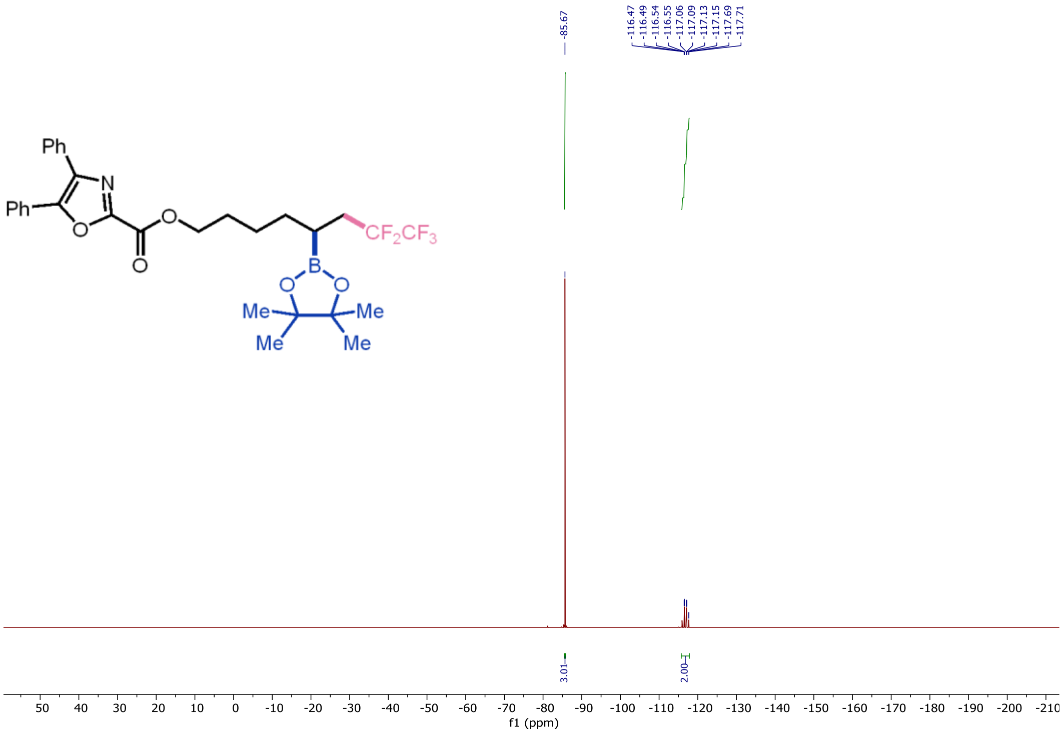

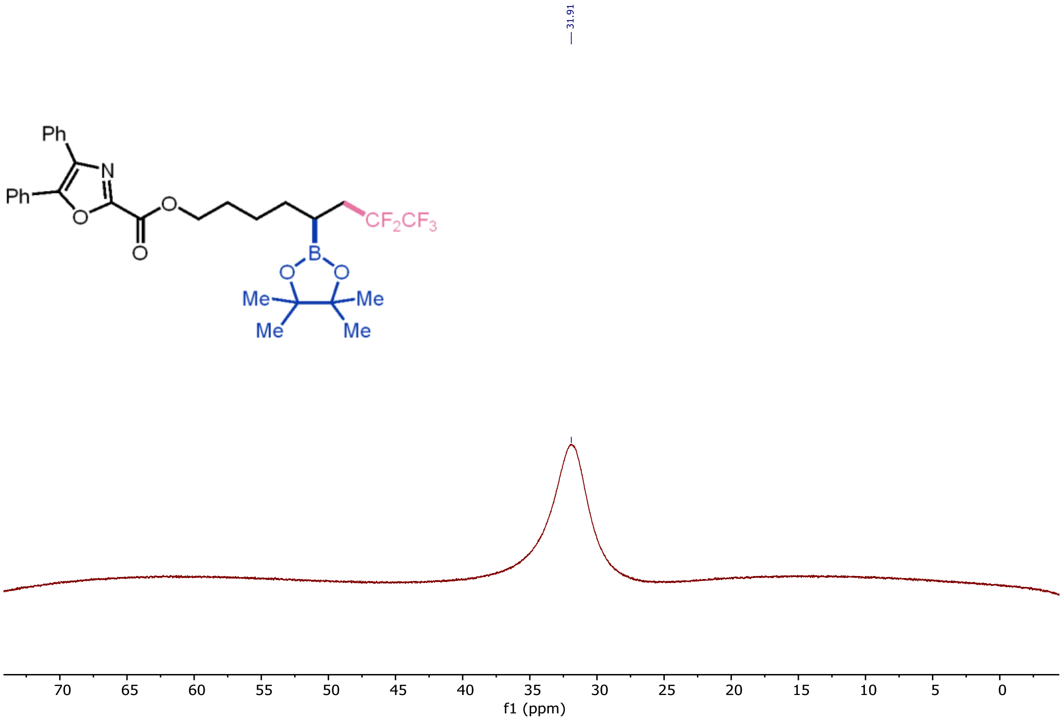

Figure S88. ^{1}H NMR (500 MHz, CDCl$_3$), ^{13}C NMR (101 MHz, CDCl$_3$), ^{19}F NMR (376 MHz, CDCl$_3$) and ^{11}B NMR (160 MHz, CDCl$_3$) spectra of **32**.

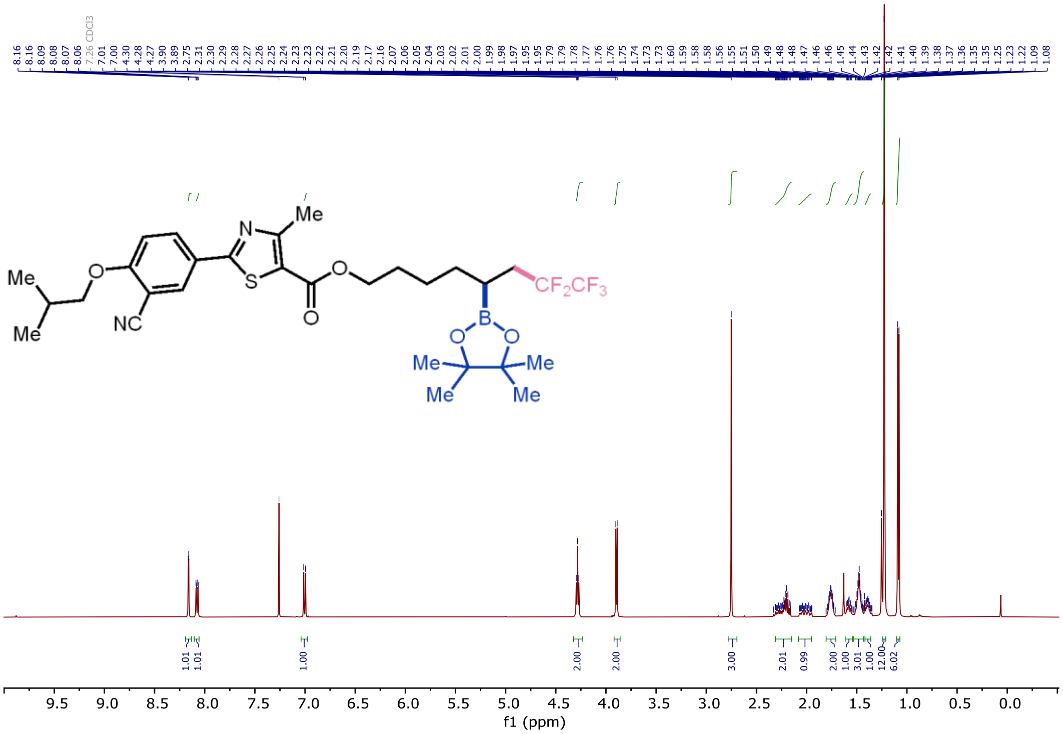

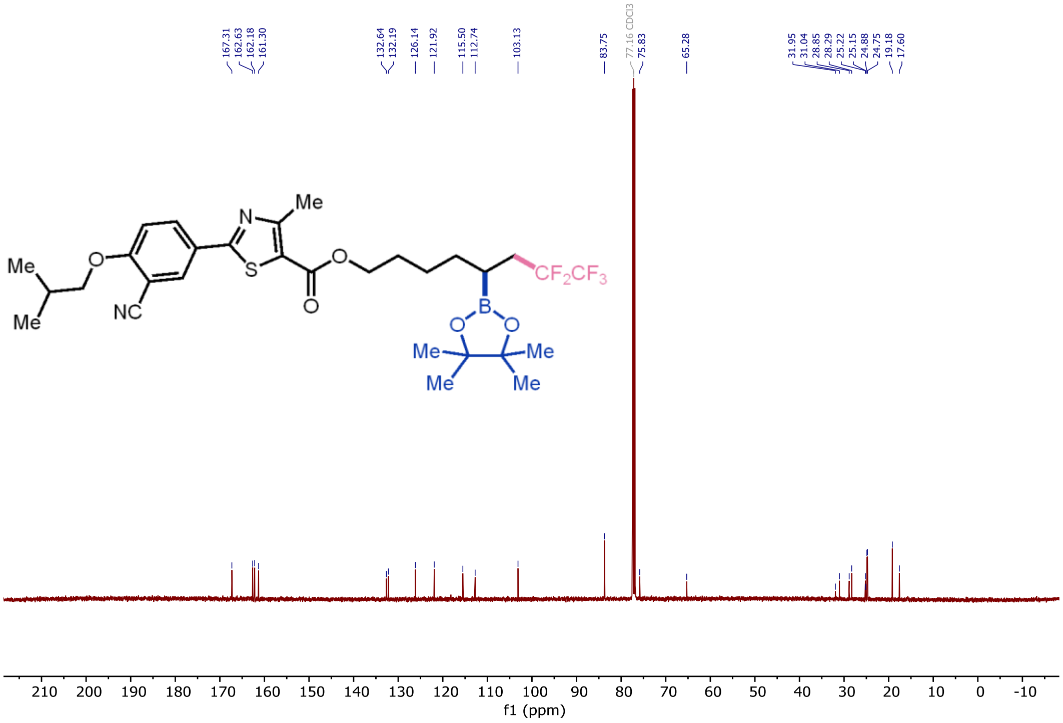

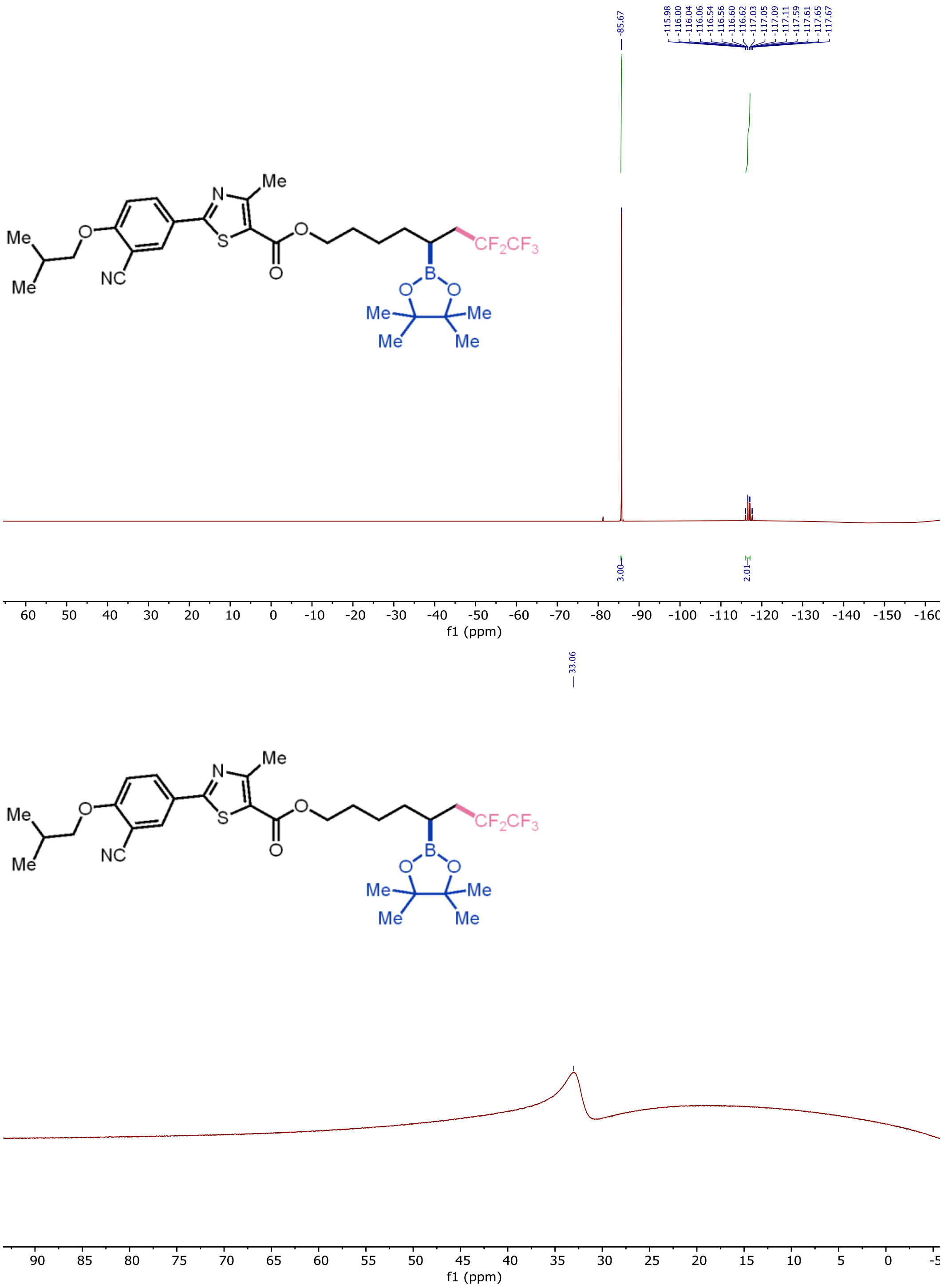

Figure S89. ^{1}H NMR (500 MHz, $CDCl_3$), ^{13}C NMR (101 MHz, $CDCl_3$), ^{19}F NMR (376 MHz, $CDCl_3$) and ^{11}B NMR (160 MHz, $CDCl_3$) spectra of **33**.

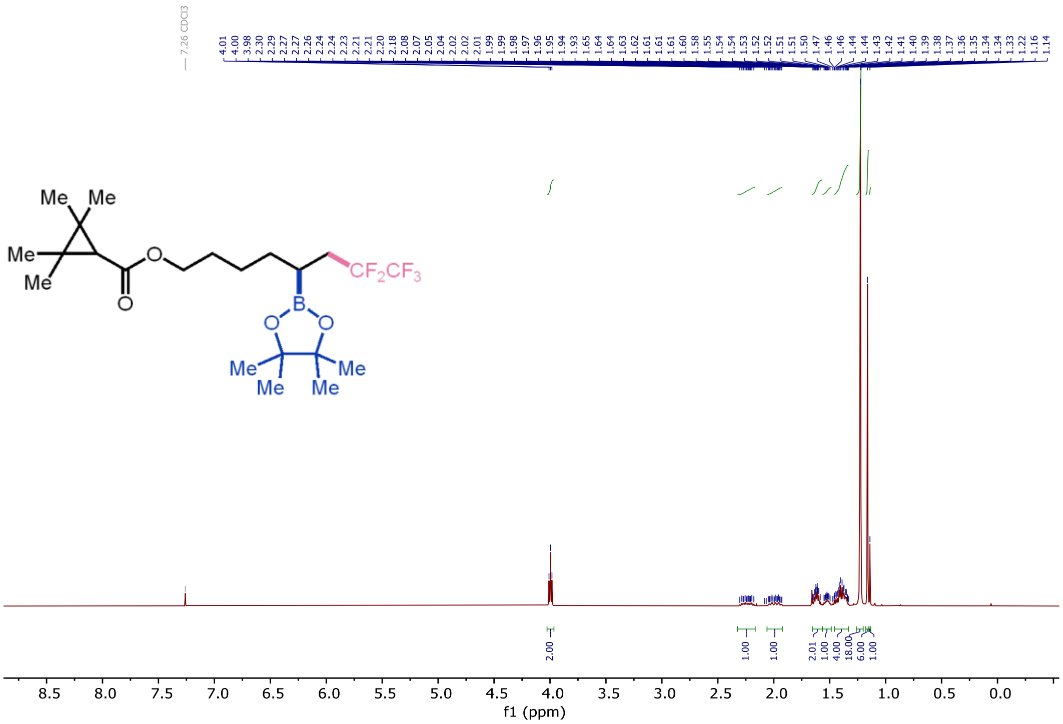

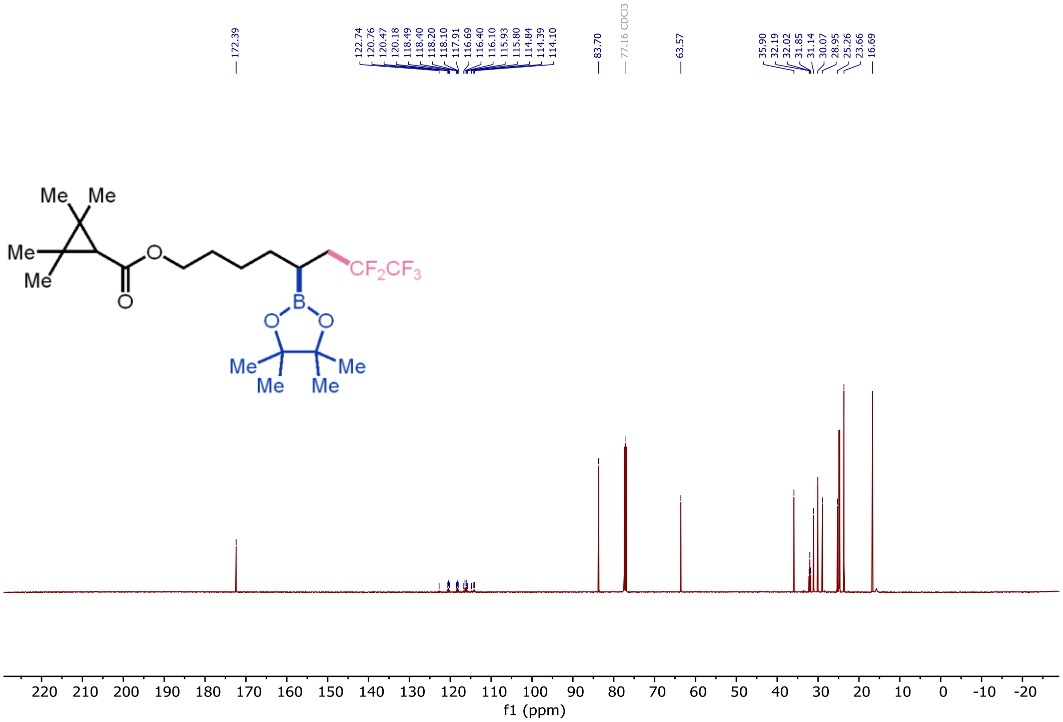

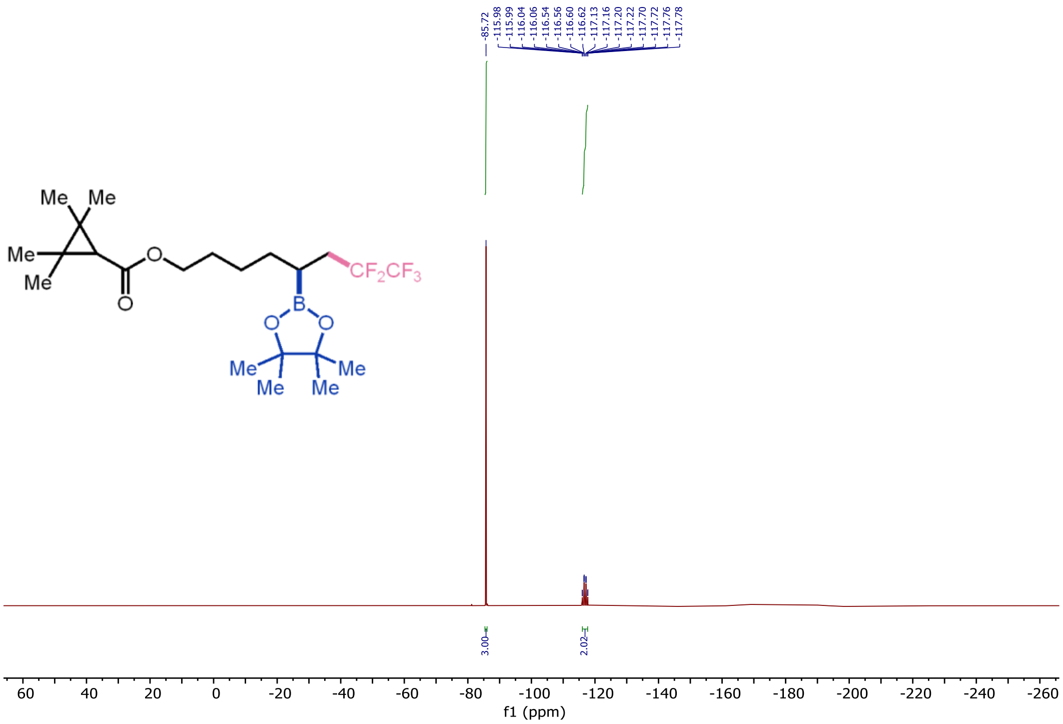

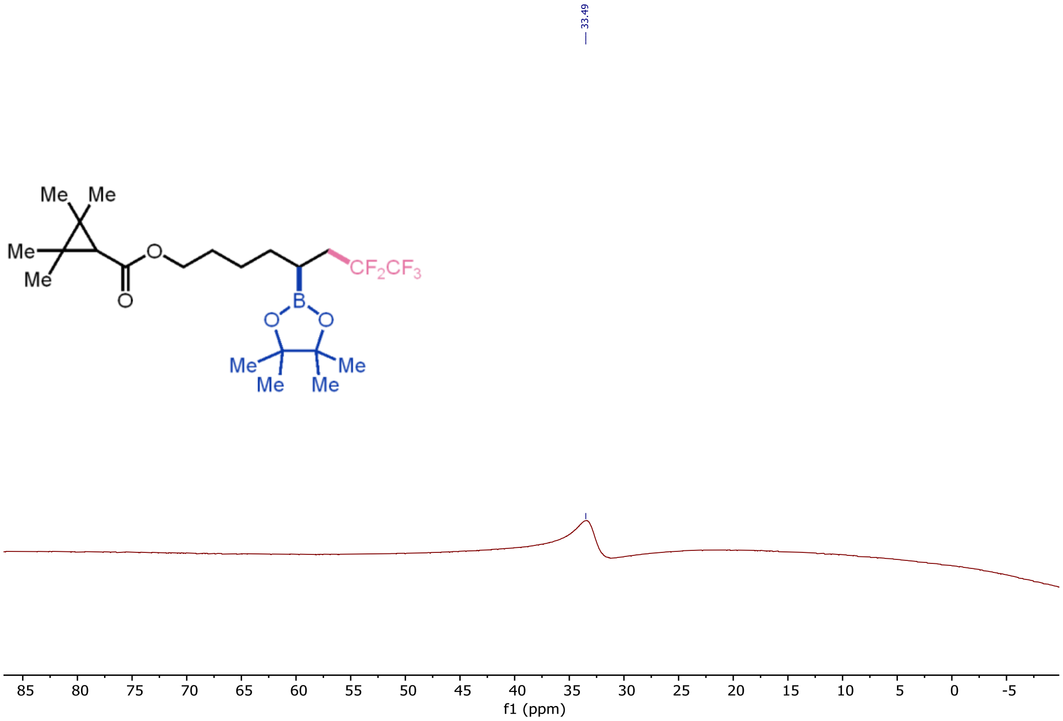

Figure S90. ^{1}H NMR (500 MHz, $CDCl_3$), ^{13}C NMR (101 MHz, $CDCl_3$), ^{19}F NMR (376 MHz, $CDCl_3$) and ^{11}B NMR (160 MHz, $CDCl_3$) spectra of **34**.

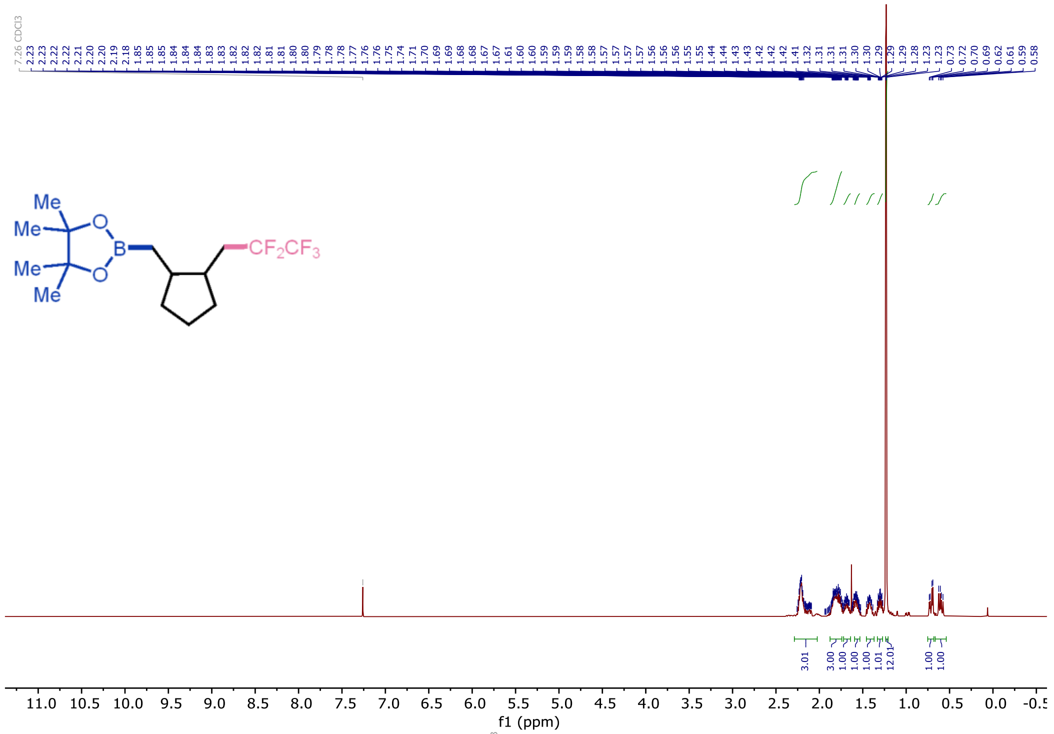
Me
Me
Me
Me
O
B
O
CF2CF3
f1 (ppm)

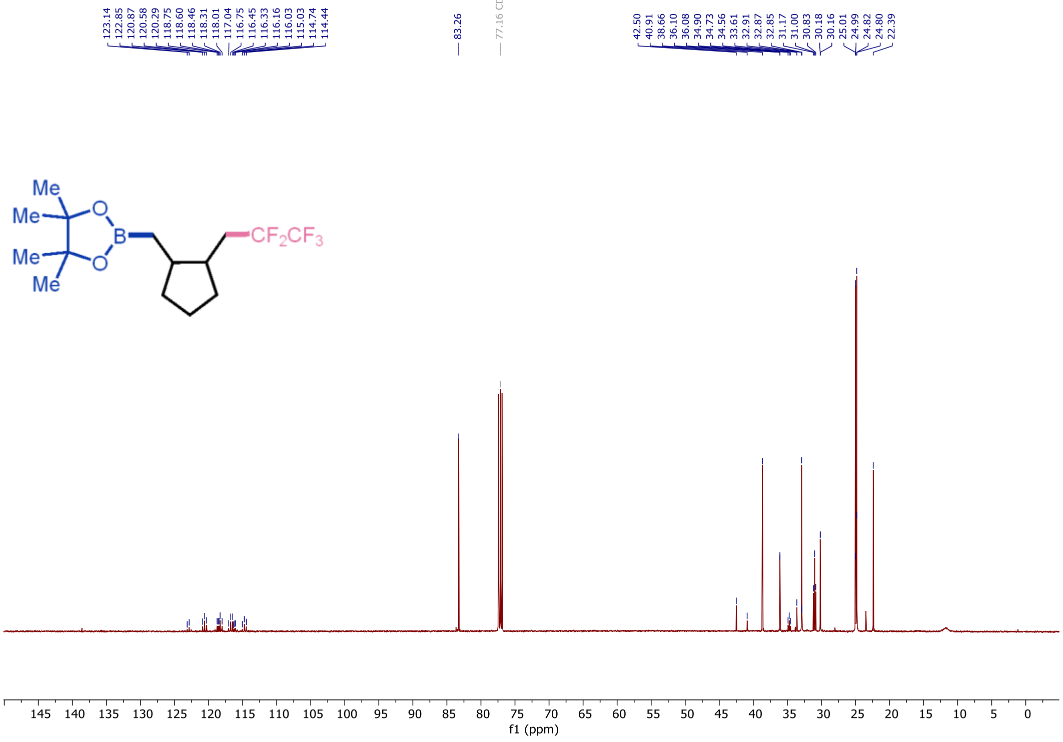
Me
Me
Me
Me
O
B
O
CF2CF3
f1 (ppm)

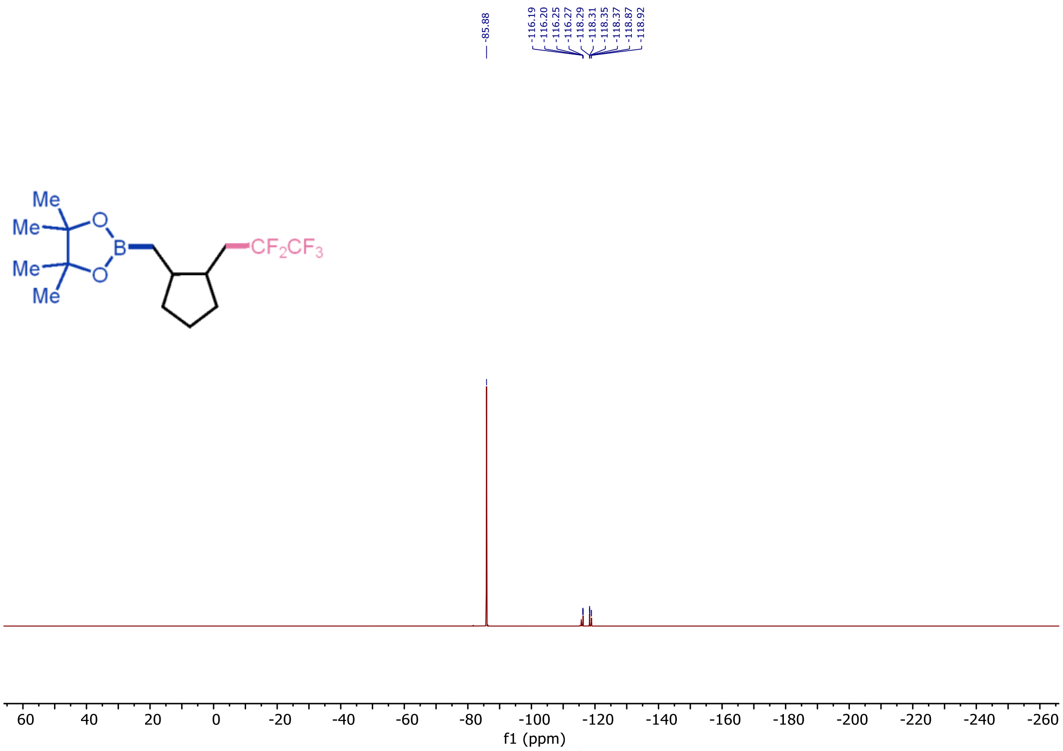

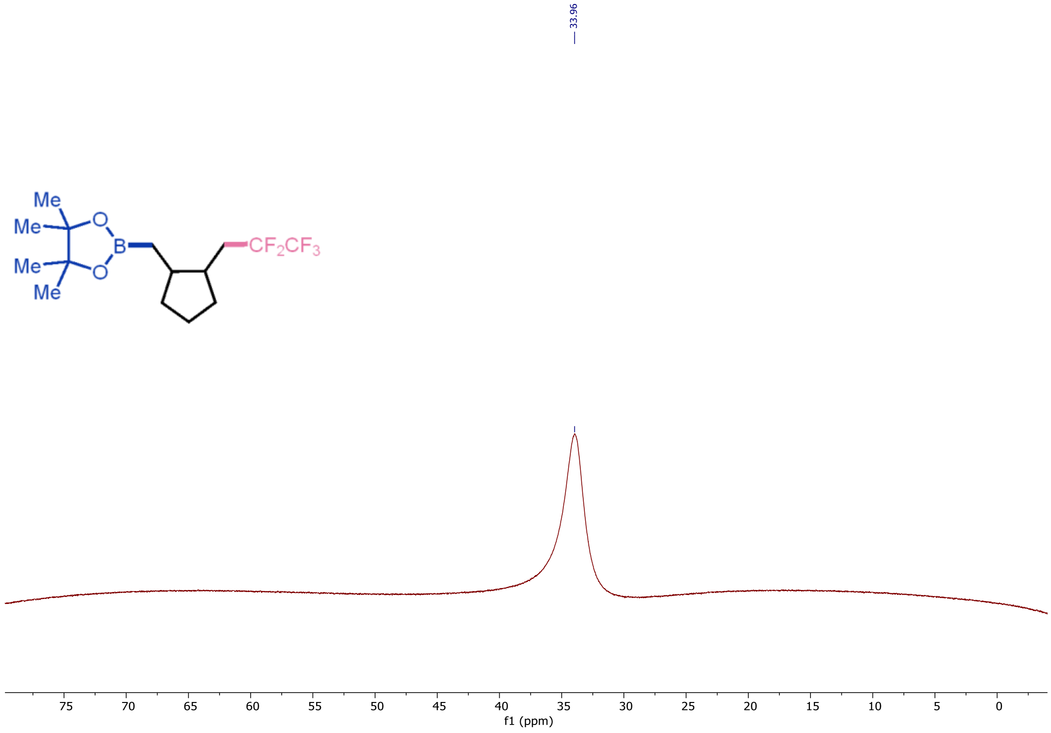

Figure S91. ^{1}H NMR (500 MHz, $CDCl_3$), ^{13}C NMR (101 MHz, $CDCl_3$), ^{19}F NMR (376 MHz, $CDCl_3$) and ^{11}B NMR (160 MHz, $CDCl_3$) spectra of **36**.

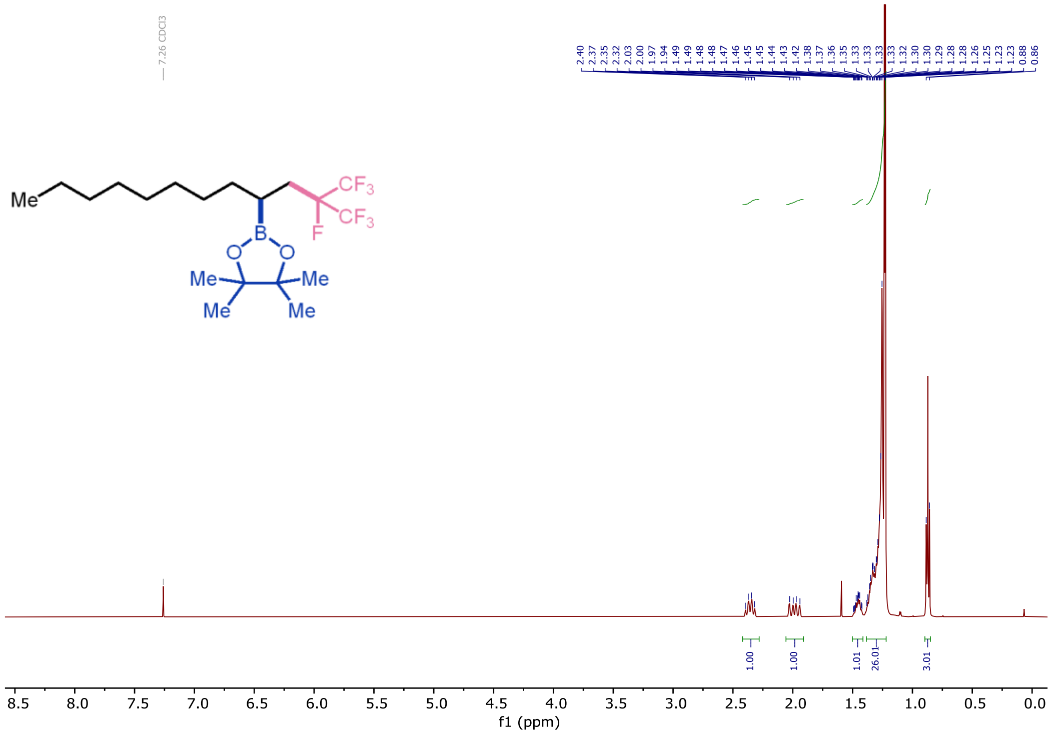

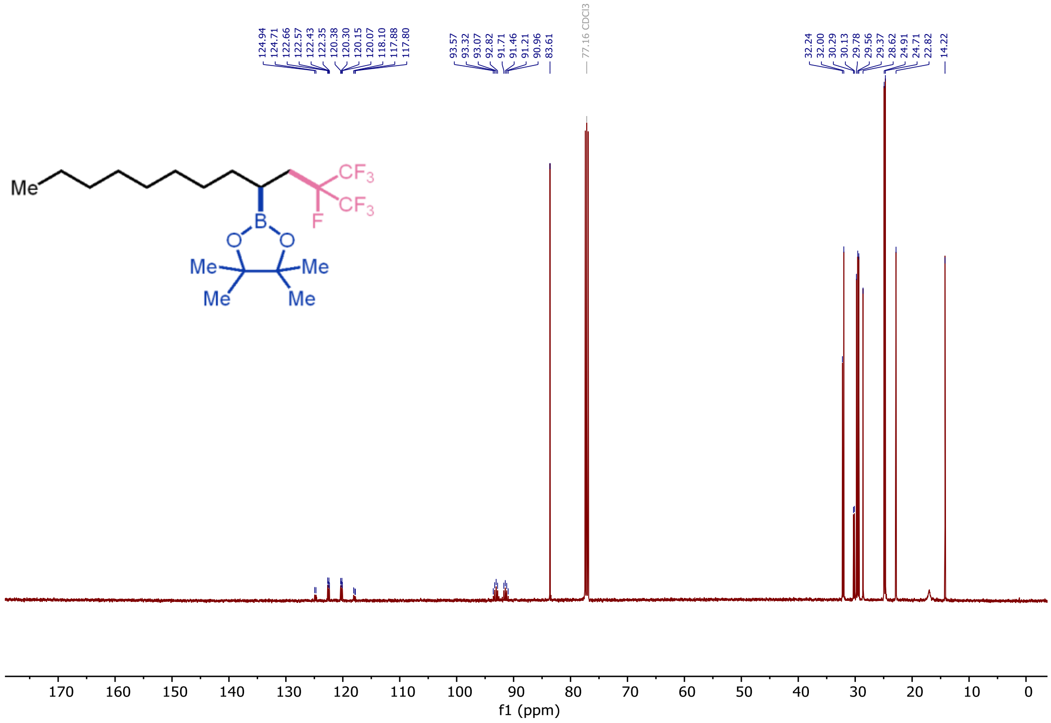

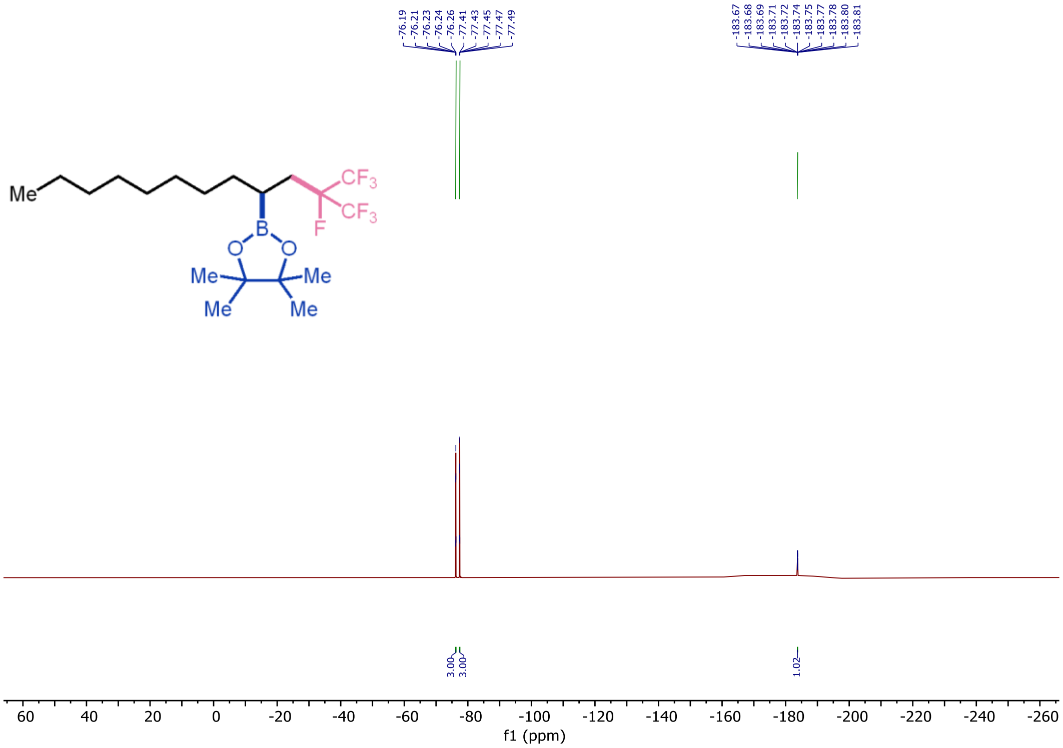

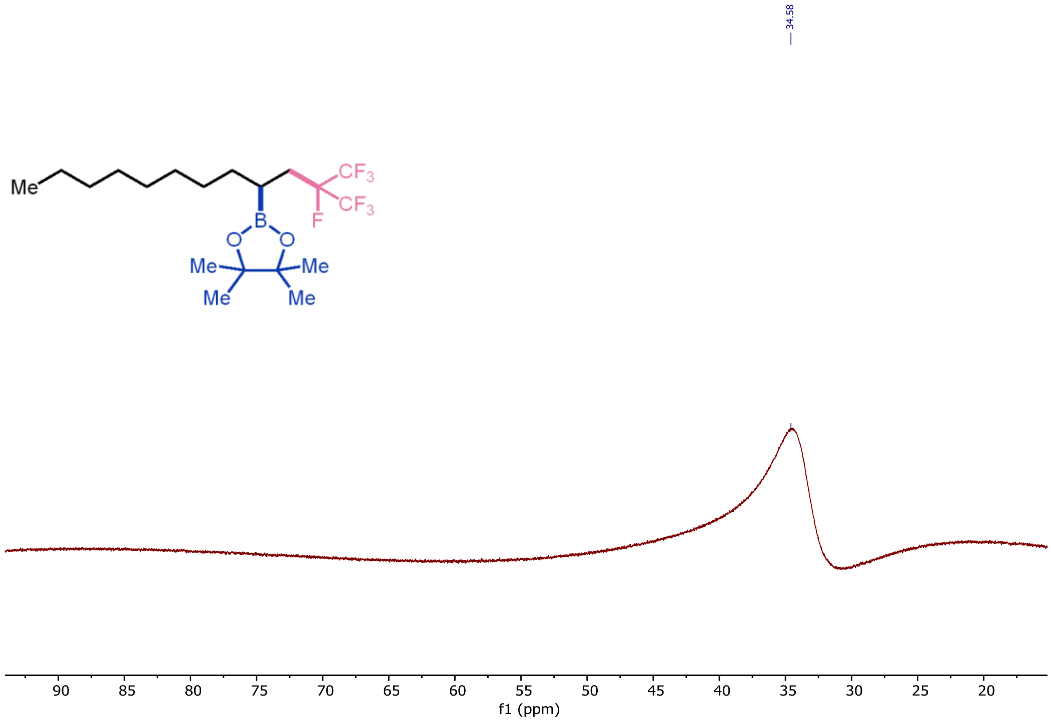

Figure S92. ^{1}H NMR (500 MHz, $CDCl_3$), ^{13}C NMR (101 MHz, $CDCl_3$), ^{19}F NMR (376 MHz, $CDCl_3$) and ^{11}B NMR (160 MHz, $CDCl_3$) spectra of **37**.

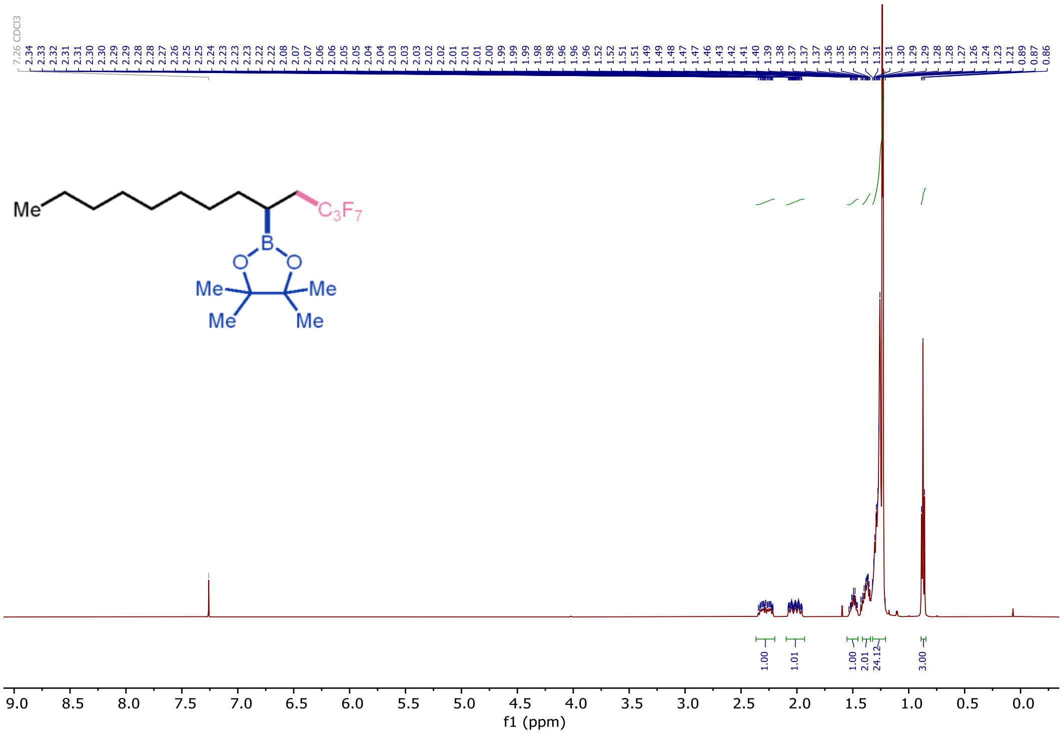
7.26 CDCl3
Me
C3F7
B
O
O
Me
Me
Me
Me
1.00
1.01
1.00
2.01
24.12
3.00
9.0 8.5 8.0 7.5 7.0 6.5 6.0 5.5 5.0 4.5 4.0 3.5 3.0 2.5 2.0 1.5 1.0 0.5 0.0
f1 (ppm)

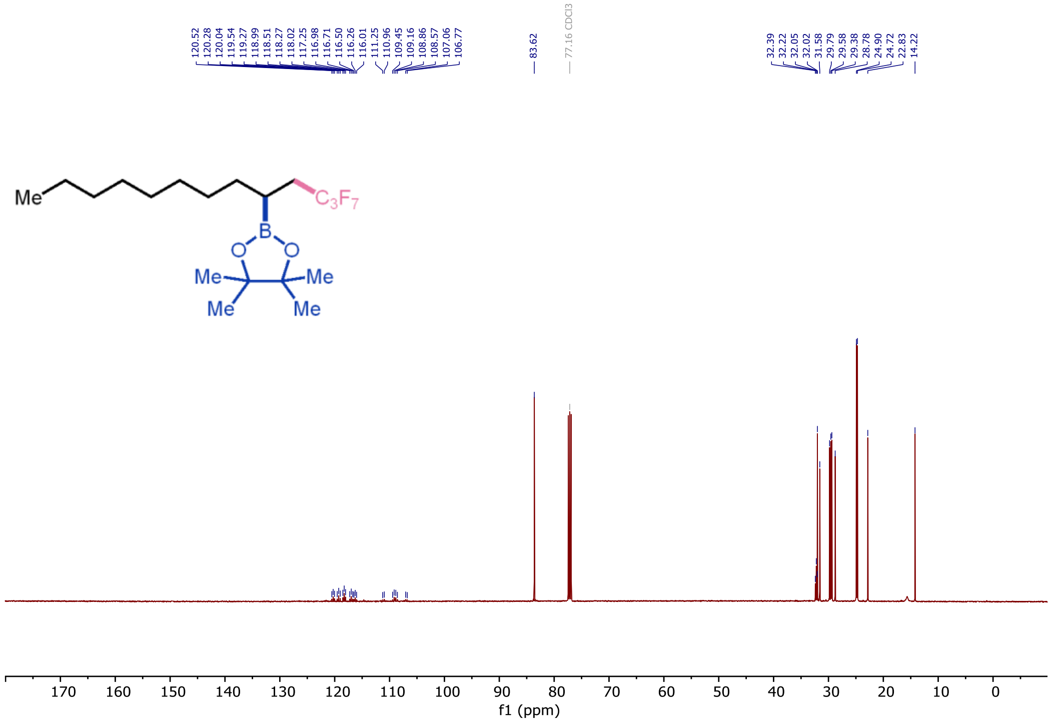
120.52 120.28 120.04 119.54 119.27 118.99 118.51 118.27 118.02 117.25 116.98 116.71 116.50 116.26 116.01 111.25 110.96 109.45 109.16 108.86 108.57 107.06 106.77
83.62
77.16 CDCl3
32.39 32.22 32.05 32.02 31.58 29.79 29.58 29.38 28.78 24.90 24.72 22.83 14.22
Me
C3F7
B
O
O
Me
Me
Me
Me
170 160 150 140 130 120 110 100 90 80 70 60 50 40 30 20 10 0
f1 (ppm)

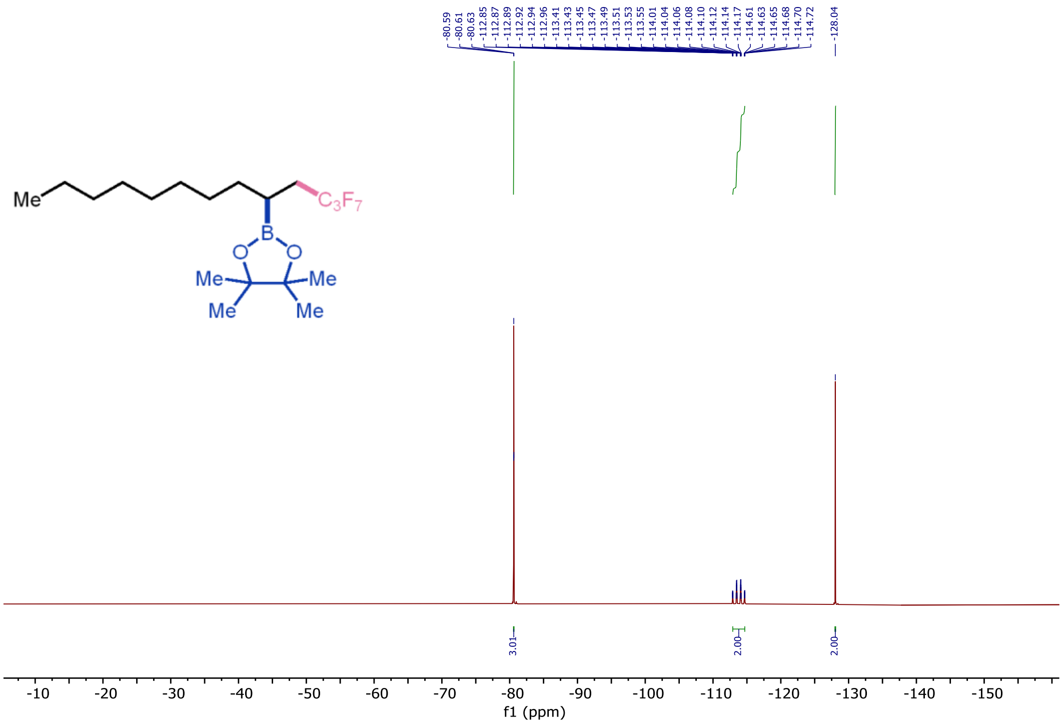

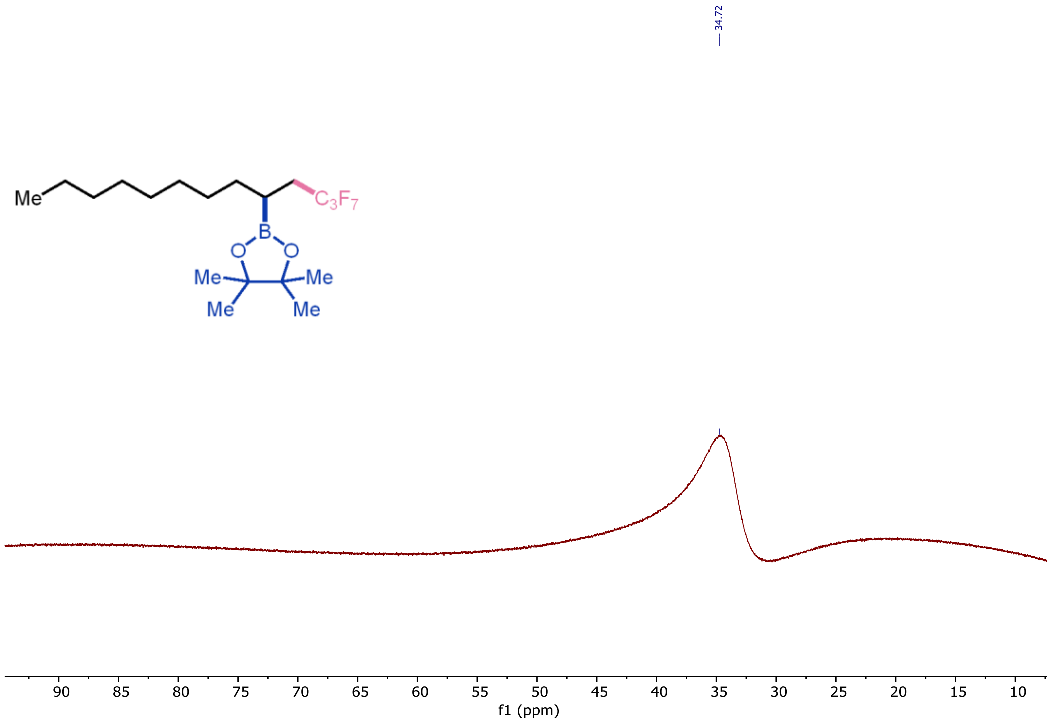

Figure S93. ^{1}H NMR (500 MHz, $CDCl_3$), ^{13}C NMR (101 MHz, $CDCl_3$), ^{19}F NMR (376 MHz, $CDCl_3$) and ^{11}B NMR (160 MHz, $CDCl_3$) spectra of **38**.

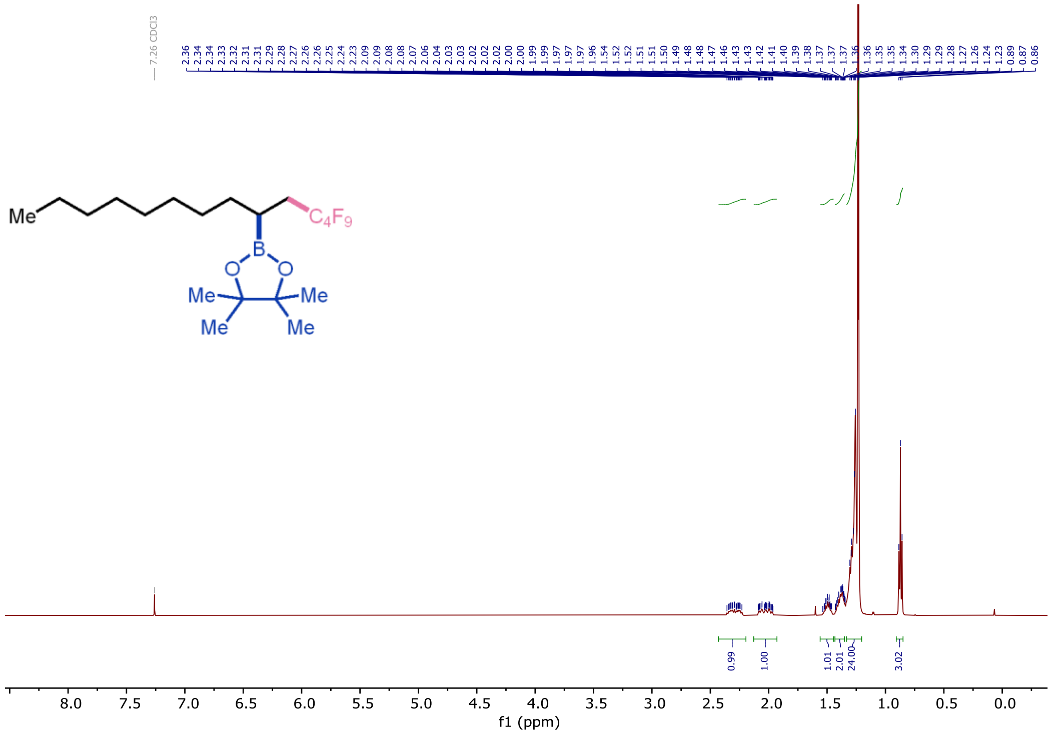
7.26 CDCl3
2.36 2.34 2.34 2.33 2.32 2.31 2.31 2.29 2.28 2.27 2.26 2.26 2.25 2.24 2.23 2.09 2.09 2.08 2.08 2.07 2.06 2.04 2.03 2.03 2.02 2.02 2.02 2.00 2.00 1.99 1.99 1.97 1.97 1.97 1.96 1.54 1.52 1.52 1.51 1.51 1.50 1.49 1.48 1.48 1.47 1.46 1.43 1.43 1.42 1.41 1.40 1.39 1.38 1.37 1.37 1.37 1.36 1.35 1.35 1.34 1.30 1.29 1.29 1.28 1.27 1.26 1.24 1.23 0.89 0.87 0.86
Me
C4F9
B
O
O
Me
Me
Me
Me
0.99
1.00
1.01
2.01
24.00
3.02
8.0 7.5 7.0 6.5 6.0 5.5 5.0 4.5 4.0 3.5 3.0 2.5 2.0 1.5 1.0 0.5 0.0
f1 (ppm)

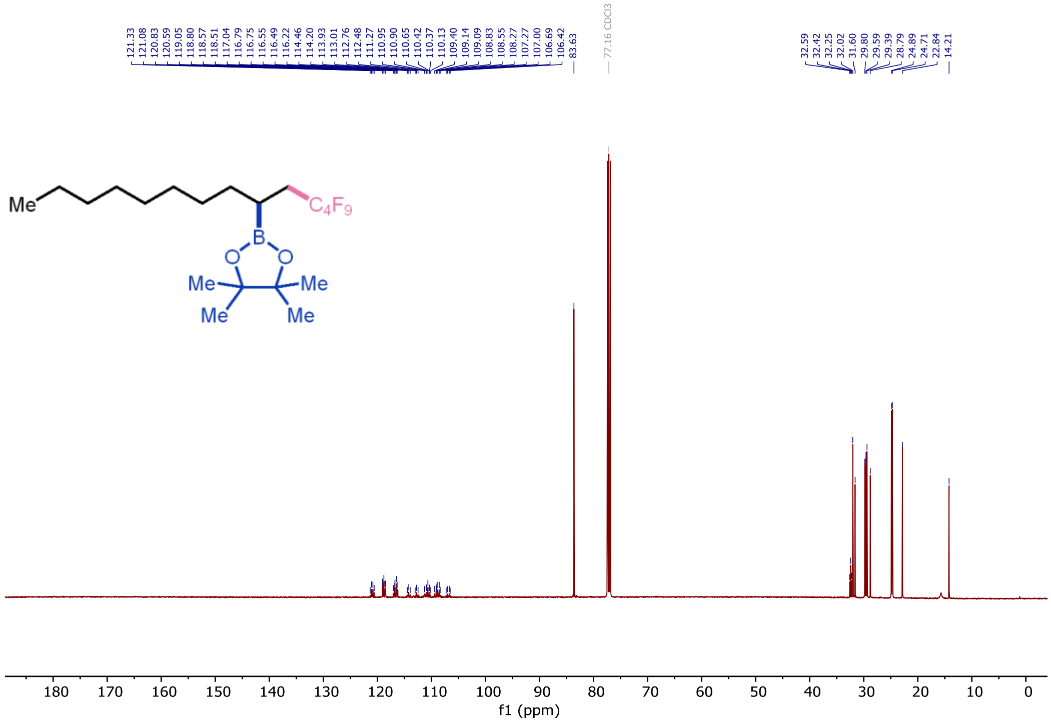
121.33 121.08 120.83 120.59 119.05 118.80 118.57 118.51 117.04 116.79 116.75 116.55 116.49 116.22 114.46 114.20 113.93 113.01 112.76 112.48 111.27 110.95 110.90 110.65 110.42 110.37 110.13 109.40 109.14 109.09 108.83 108.55 108.27 107.27 107.00 106.69 106.42
83.63
77.16 CDCl3
32.59 32.42 32.25 32.02 31.60 29.80 29.59 29.39 28.79 24.89 24.71 22.84
14.21
Me
C4F9
B
O
O
Me
Me
Me
Me
180 170 160 150 140 130 120 110 100 90 80 70 60 50 40 30 20 10 0
f1 (ppm)

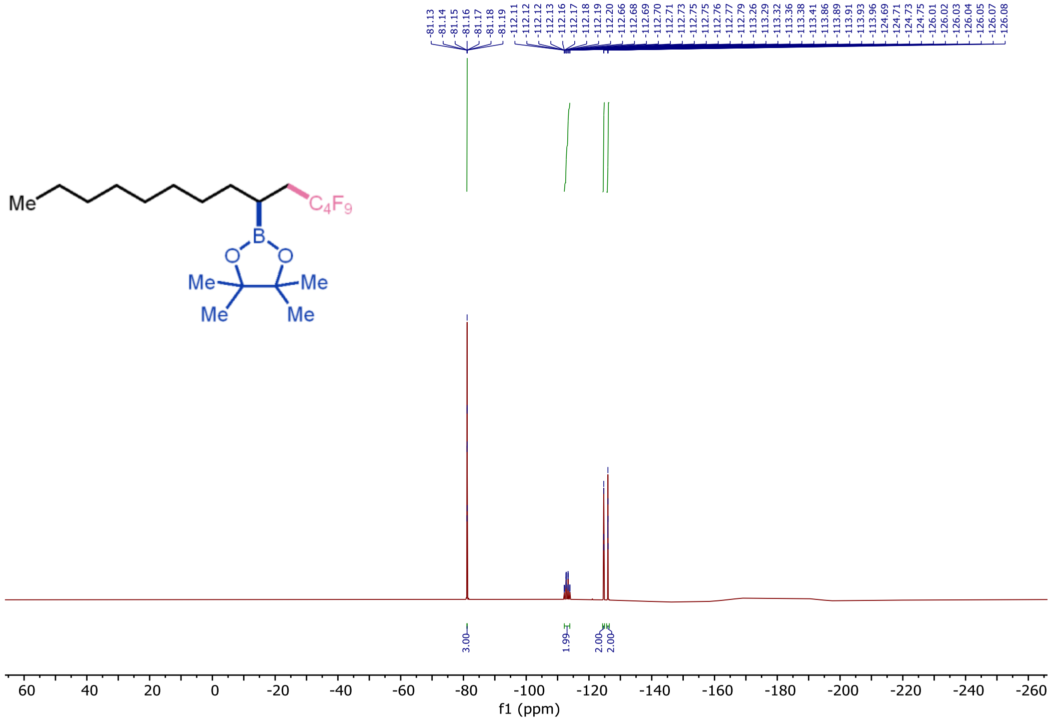

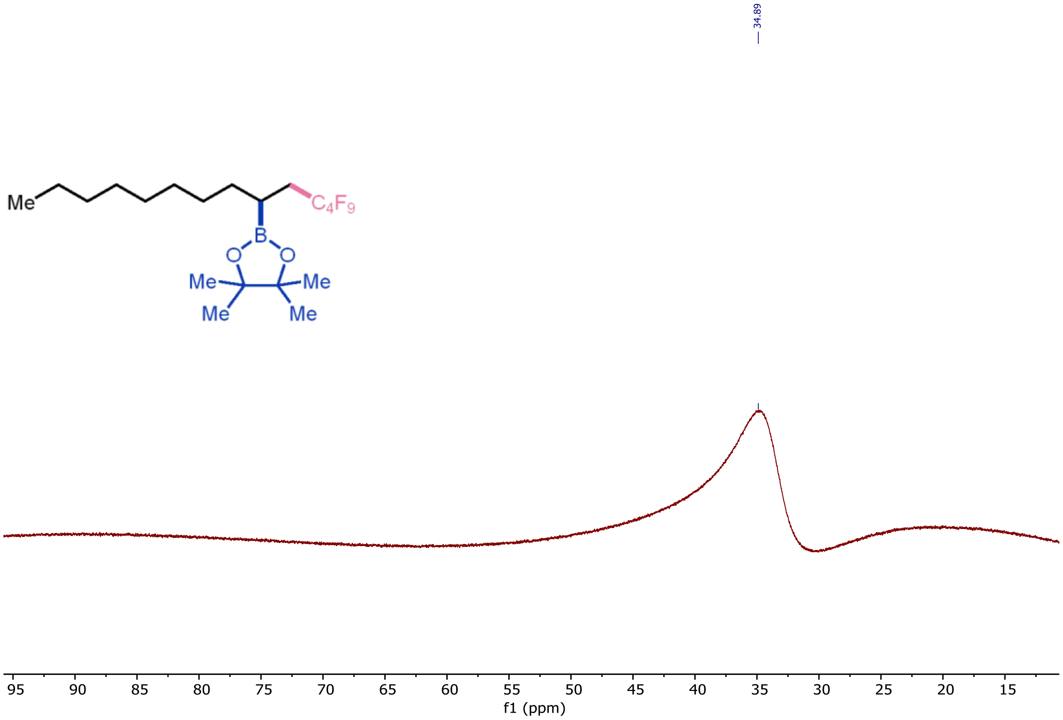

Figure S94. ^{1}H NMR (500 MHz, $CDCl_3$), ^{13}C NMR (101 MHz, $CDCl_3$), ^{19}F NMR (376 MHz, $CDCl_3$) and ^{11}B NMR (160 MHz, $CDCl_3$) spectra of **39**.

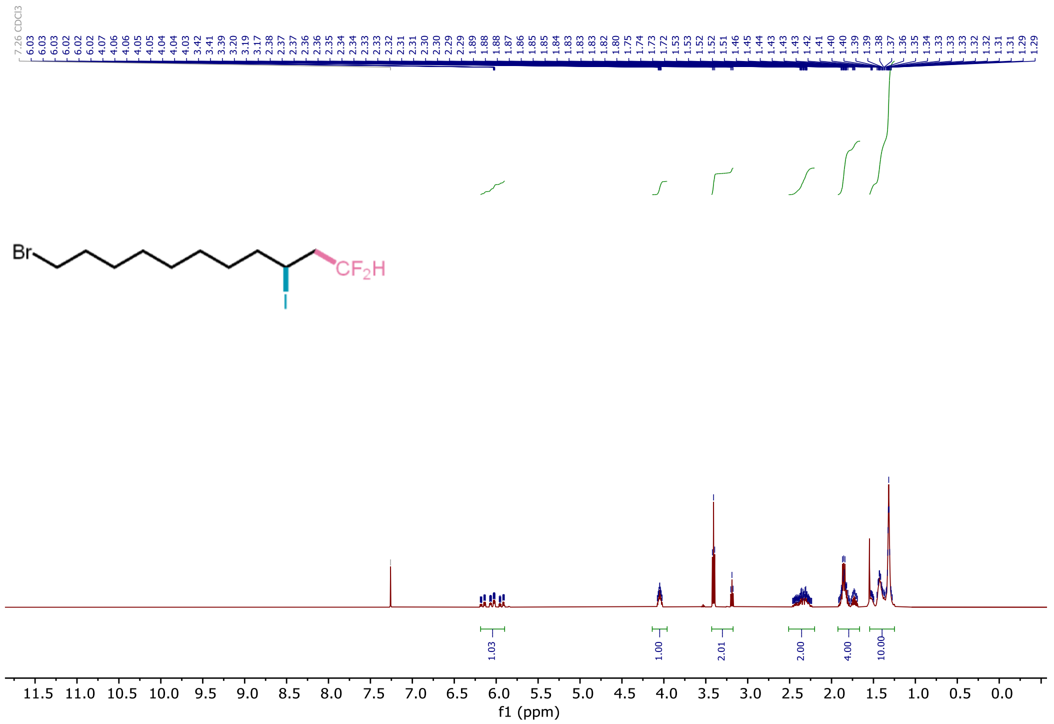
7.26 CDCl3
Br
CF2H
I
1.03
1.00
2.01
2.00
4.00
10.00
f1 (ppm)

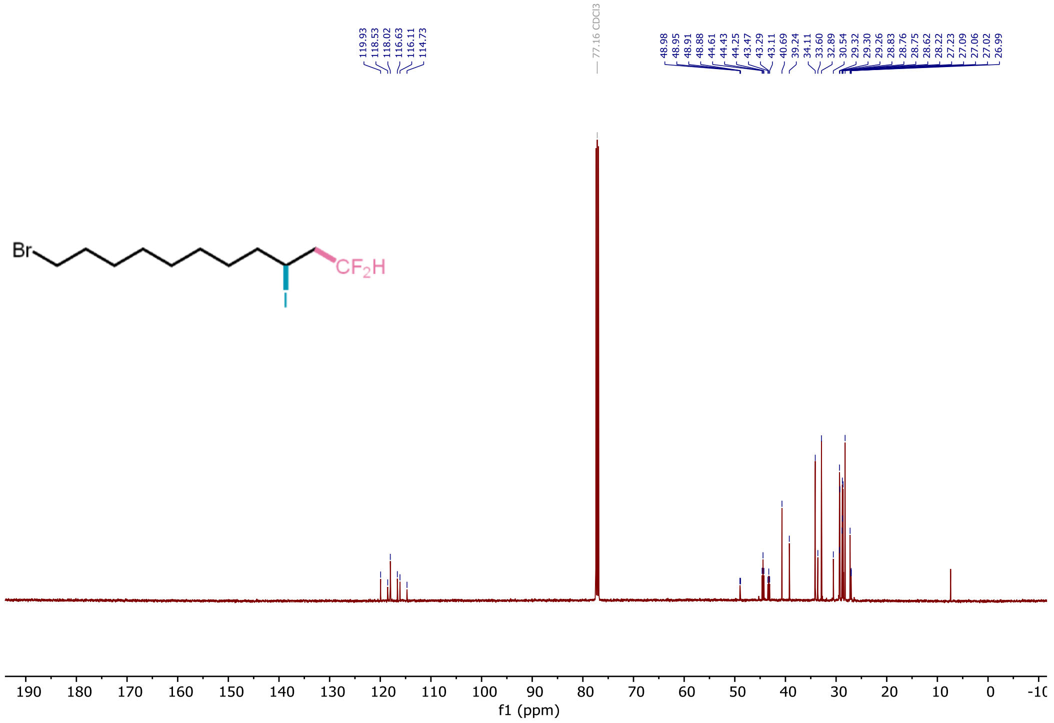
119.93
118.53
118.02
116.63
116.11
114.73
77.16 CDCl3
48.98
48.95
48.91
48.88
44.61
44.43
44.25
43.47
43.29
43.11
40.69
39.24
34.11
33.60
32.89
30.54
29.32
29.30
29.26
28.83
28.76
28.75
28.62
28.22
27.23
27.09
27.06
27.02
26.99
Br
CF2H
I
f1 (ppm)

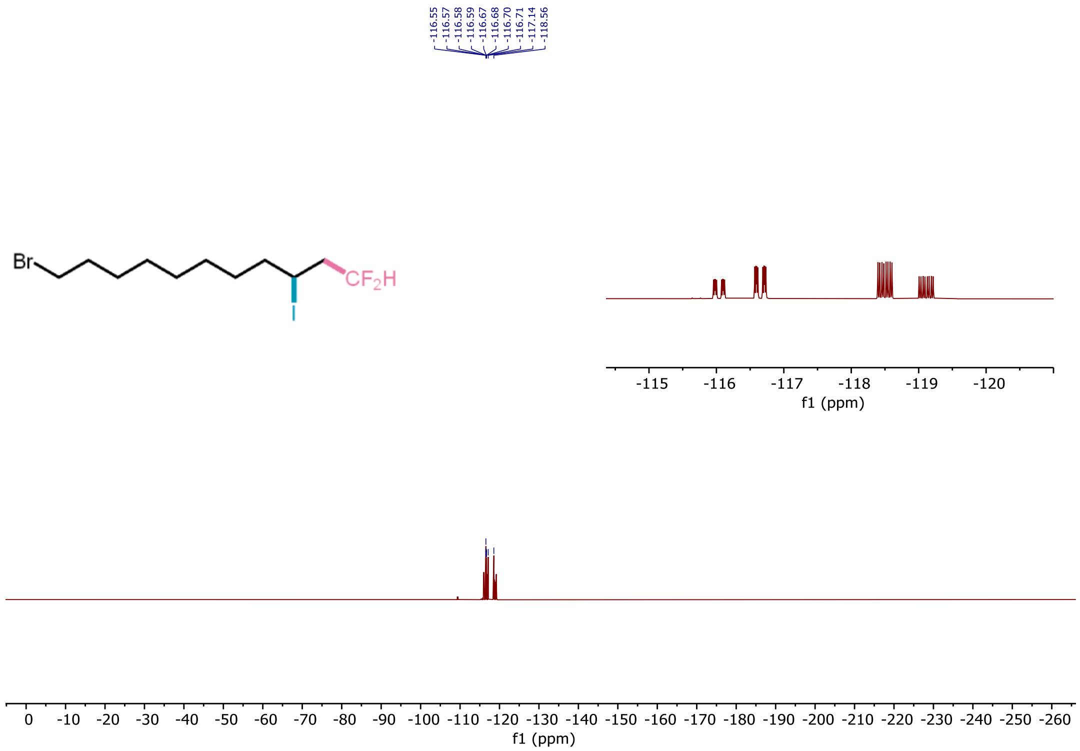

Figure S95. ^{1}H NMR (500 MHz, $CDCl_3$), ^{13}C NMR (101 MHz, $CDCl_3$) and ^{19}F NMR (376 MHz, $CDCl_3$) spectra of **40**.

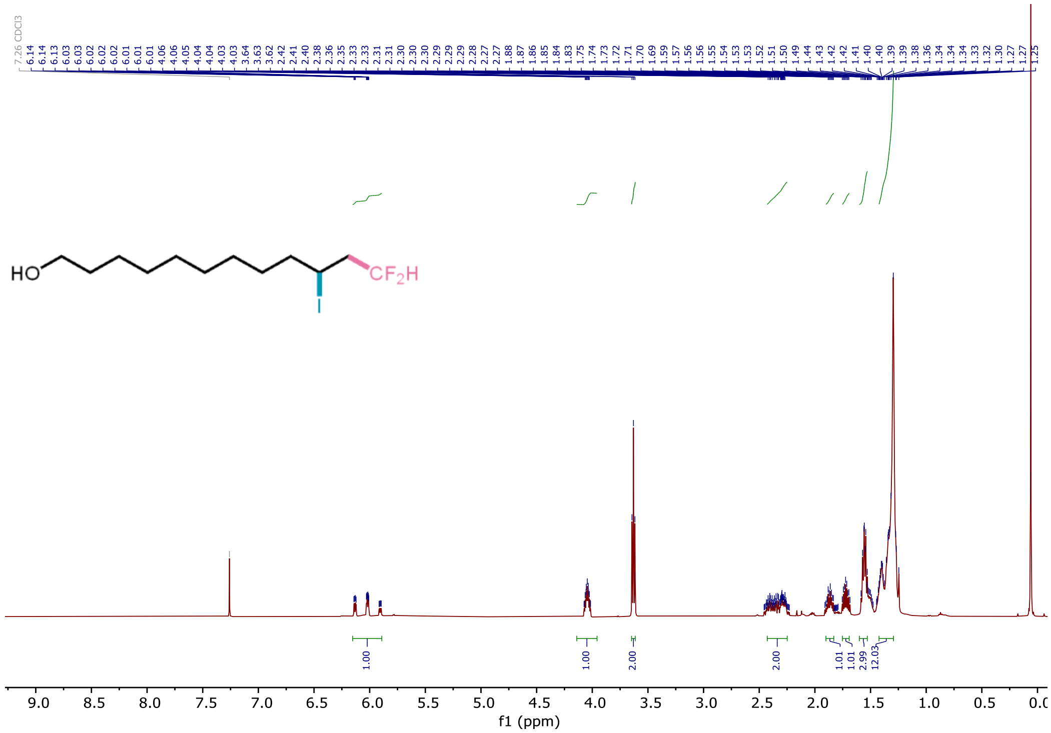
HO
CF2H
I
f1 (ppm)

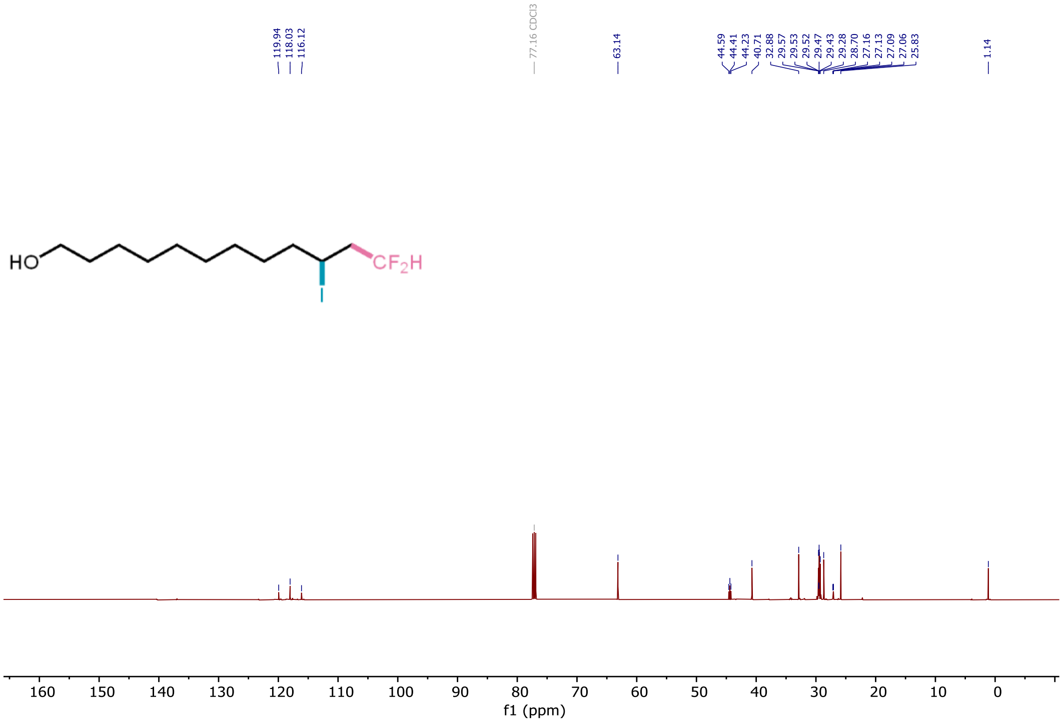
HO
CF2H
I
f1 (ppm)

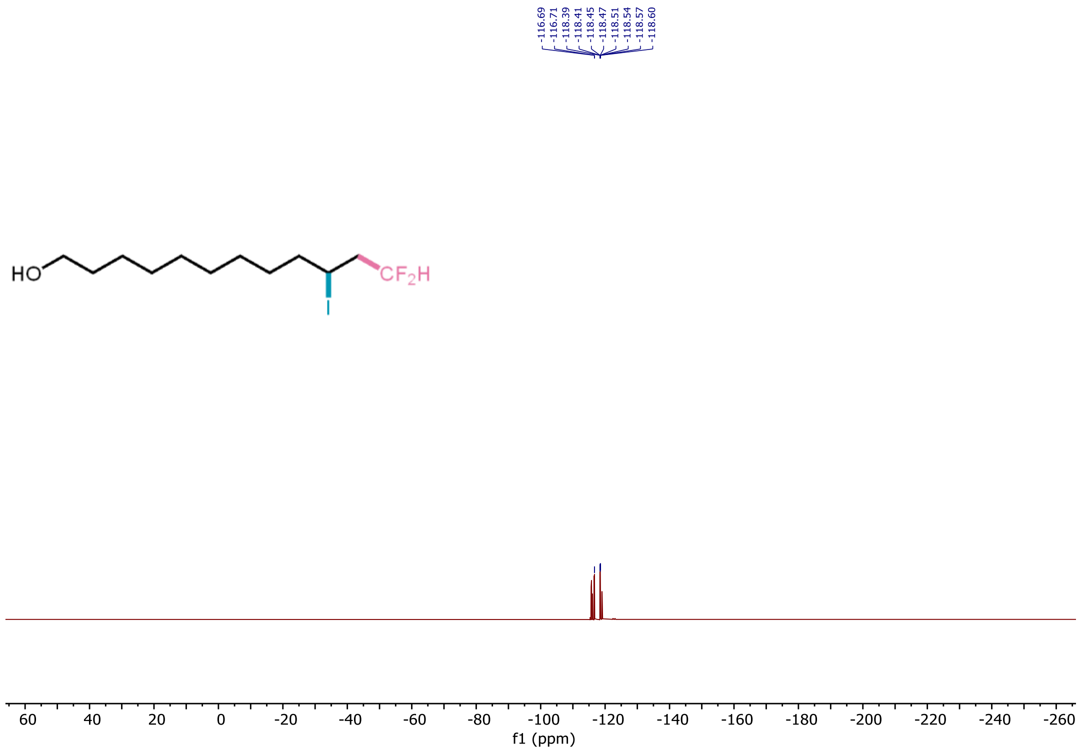

Figure S96. ^{1}H NMR (500 MHz, $CDCl_3$), ^{13}C NMR (101 MHz, $CDCl_3$) and ^{19}F NMR (376 MHz, $CDCl_3$) spectra of **41**.

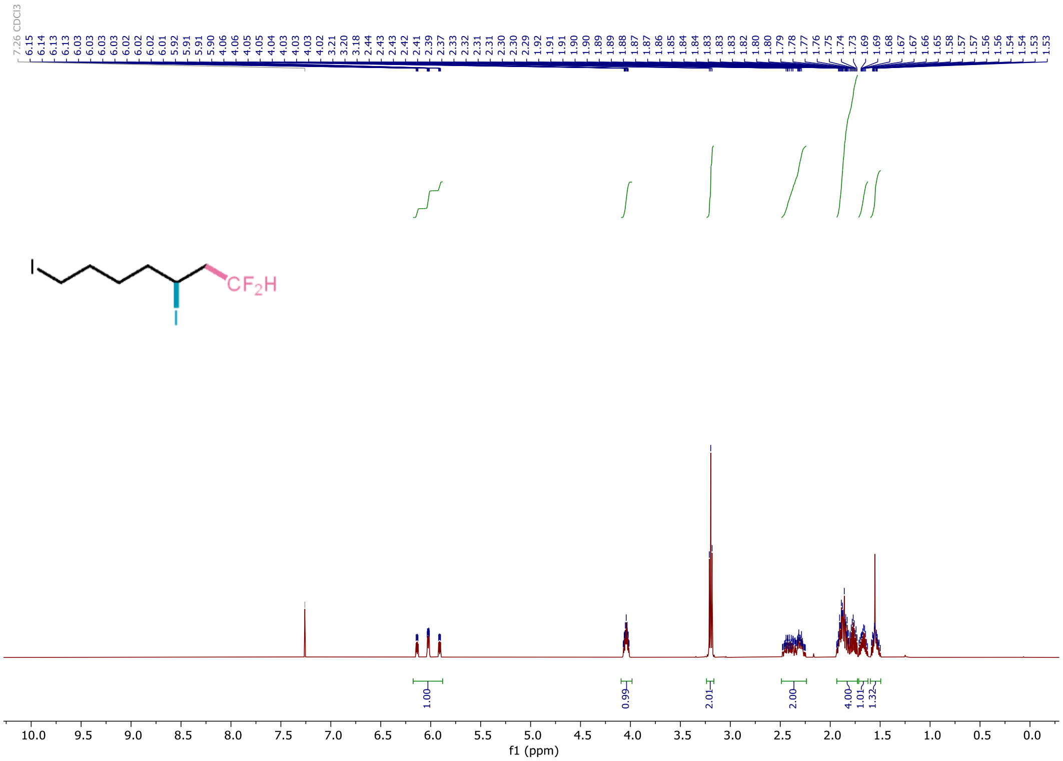
7.26 CDCl3
6.15 6.14 6.13 6.13 6.03 6.03 6.03 6.03 6.02 6.02 6.02 6.01 5.92 5.91 5.91 5.90 4.06 4.06 4.05 4.05 4.04 4.03 4.03 4.03 4.02 3.21 3.20 3.18 2.44 2.43 2.43 2.42 2.41 2.39 2.37 2.33 2.32 2.31 2.31 2.30 2.30 2.29 1.92 1.91 1.91 1.90 1.90 1.89 1.89 1.88 1.87 1.87 1.86 1.85 1.84 1.84 1.83 1.83 1.83 1.83 1.82 1.80 1.80 1.79 1.78 1.77 1.76 1.75 1.74 1.73 1.69 1.69 1.68 1.67 1.67 1.66 1.65 1.58 1.57 1.57 1.56 1.56 1.54 1.53 1.53
I
CF2H
I
1.00
0.99
2.01
2.00
4.00
1.01
1.32
10.0 9.5 9.0 8.5 8.0 7.5 7.0 6.5 6.0 5.5 5.0 4.5 4.0 3.5 3.0 2.5 2.0 1.5 1.0 0.5 0.0
f1 (ppm)

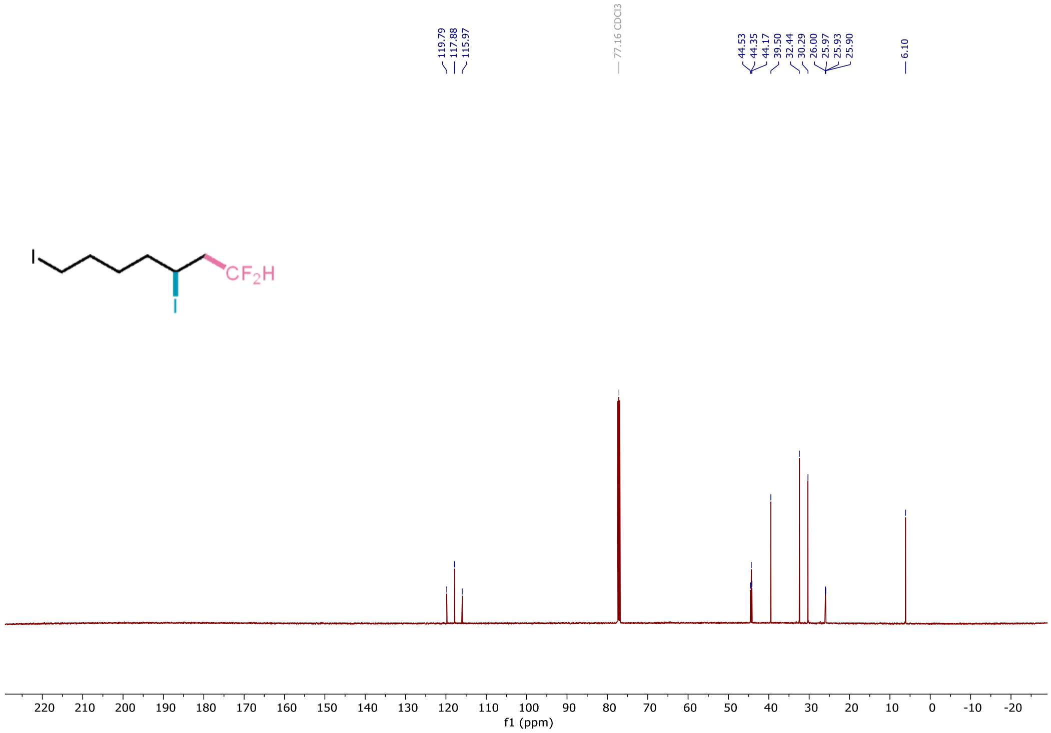
119.79
117.88
115.97
77.16 CDCl3
44.53 44.35 44.17 39.50 32.44 30.29 26.00 25.97 25.93 25.90
6.10
I
CF2H
I
220 210 200 190 180 170 160 150 140 130 120 110 100 90 80 70 60 50 40 30 20 10 0 -10 -20
f1 (ppm)

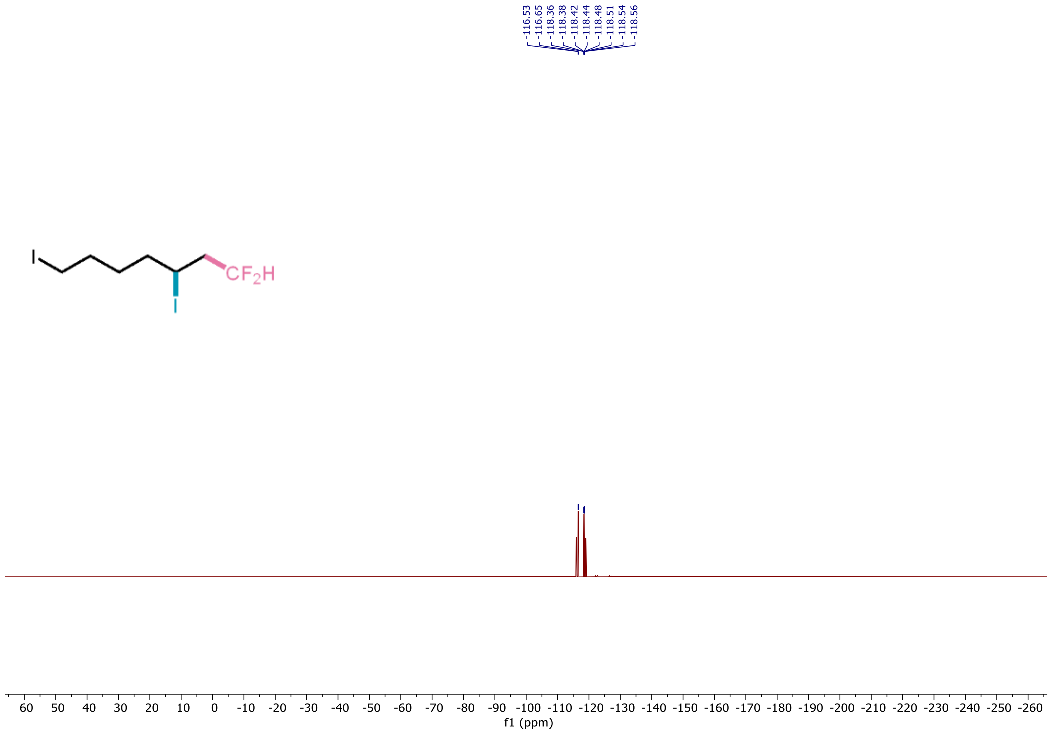

Figure S97. ^{1}H NMR (500 MHz, $CDCl_3$), ^{13}C NMR (101 MHz, $CDCl_3$) and ^{19}F NMR (376 MHz, $CDCl_3$) spectra of **42**.

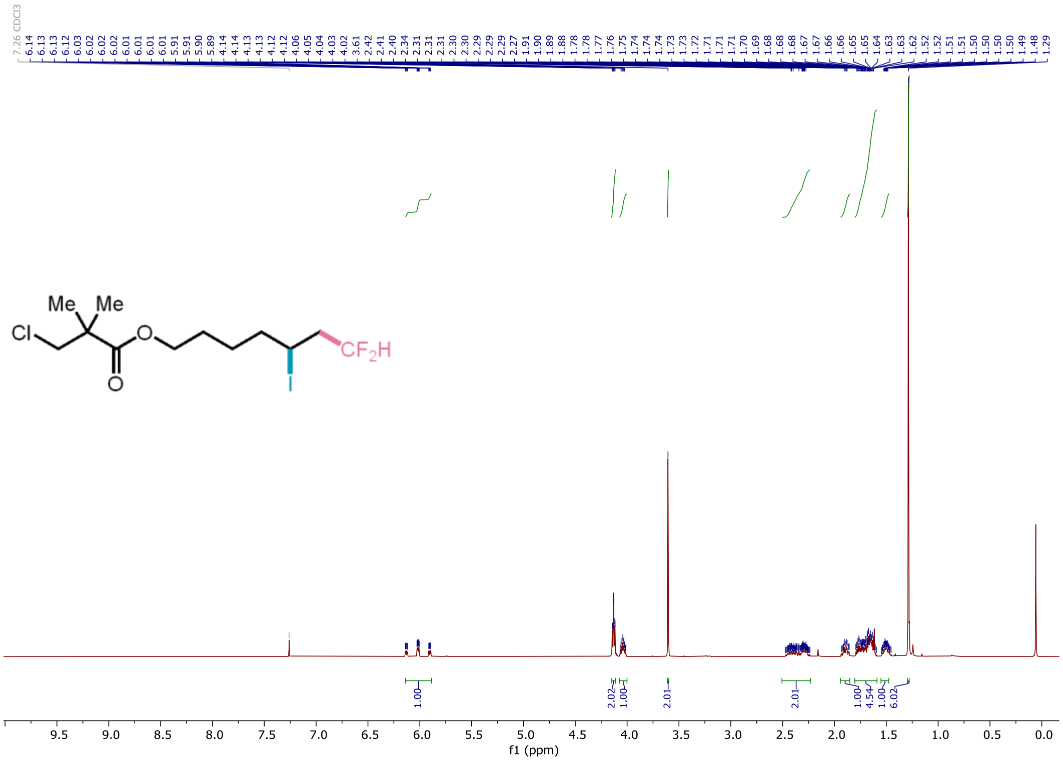
7.26 CDCl3
6.14 6.13 6.13 6.12 6.03 6.02 6.02 6.01 6.01 6.01 6.01 5.91 5.91 5.90 5.89 4.14 4.14 4.13 4.13 4.12 4.12 4.06 4.05 4.04 4.03 4.02 3.61 2.42 2.41 2.40 2.34 2.31 2.31 2.30 2.30 2.29 2.29 2.29 2.27 1.91 1.90 1.89 1.88 1.78 1.77 1.76 1.75 1.74 1.74 1.74 1.73 1.73 1.72 1.71 1.71 1.71 1.70 1.69 1.68 1.68 1.68 1.67 1.67 1.66 1.66 1.65 1.65 1.64 1.63 1.63 1.62 1.52 1.52 1.51 1.51 1.50 1.50 1.50 1.50 1.49 1.48 1.29
Me
Me
Cl
O
O
CF2H
I
1.00
2.02
1.00
2.01
2.01
1.00
4.54
1.00
6.02
9.5 9.0 8.5 8.0 7.5 7.0 6.5 6.0 5.5 5.0 4.5 4.0 3.5 3.0 2.5 2.0 1.5 1.0 0.5 0.0
f1 (ppm)

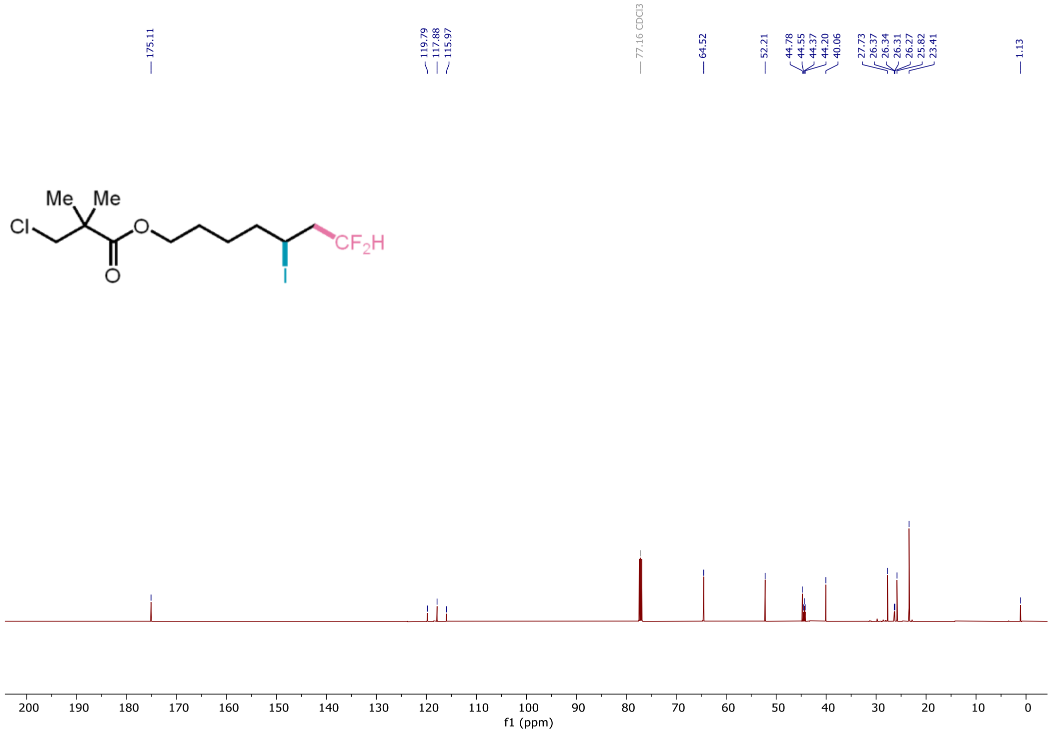
175.11
119.79 117.88 115.97
77.16 CDCl3
64.52
52.21
44.78 44.55 44.37 44.20 40.06
27.73 26.37 26.34 26.31 26.27 25.82 23.41
1.13
Me
Me
Cl
O
O
CF2H
I
200 190 180 170 160 150 140 130 120 110 100 90 80 70 60 50 40 30 20 10 0
f1 (ppm)

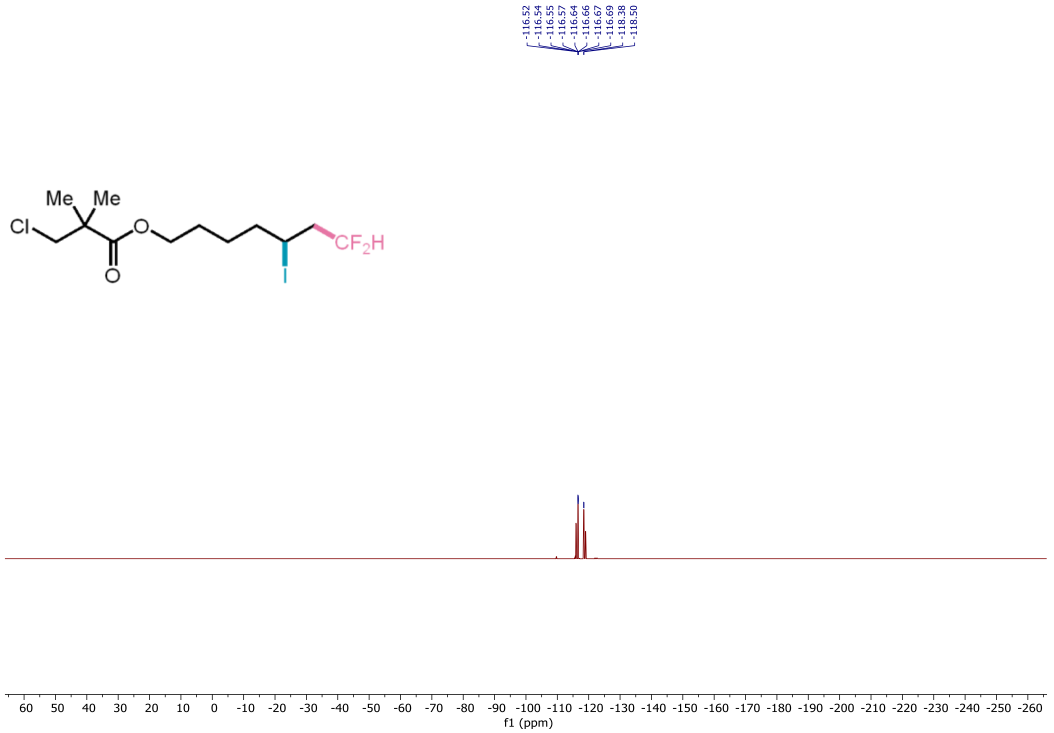

Figure S98. ^{1}H NMR (500 MHz, $CDCl_3$), ^{13}C NMR (101 MHz, $CDCl_3$) and ^{19}F NMR (376 MHz, $CDCl_3$) spectra of **43**.

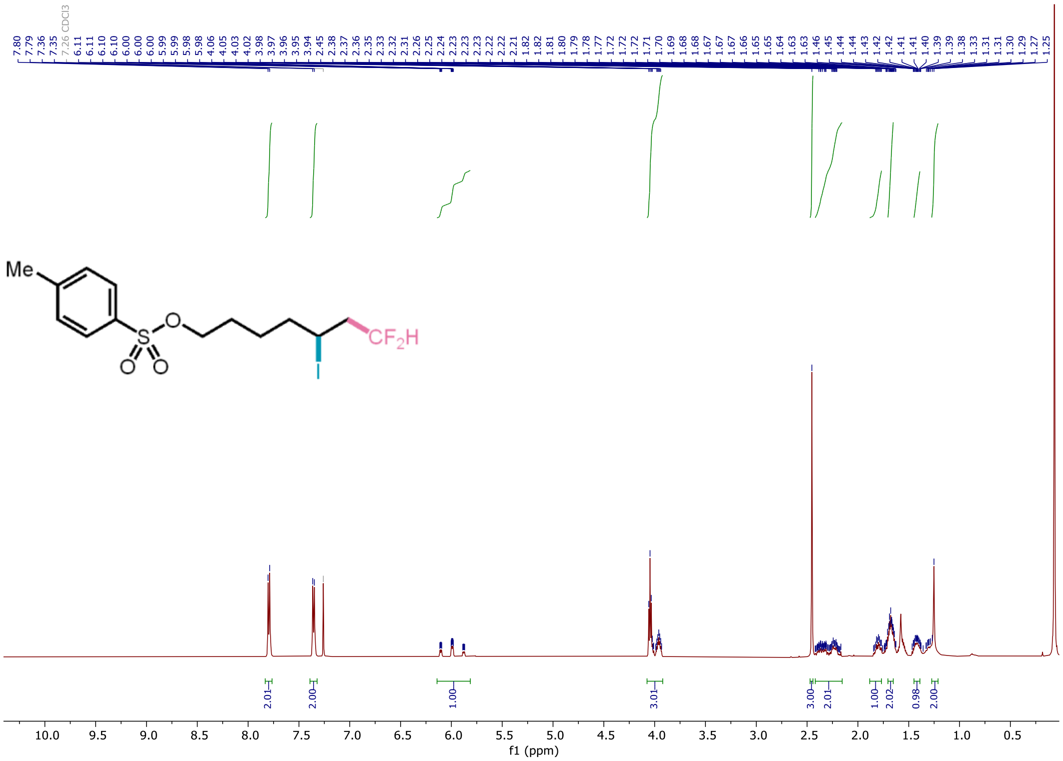

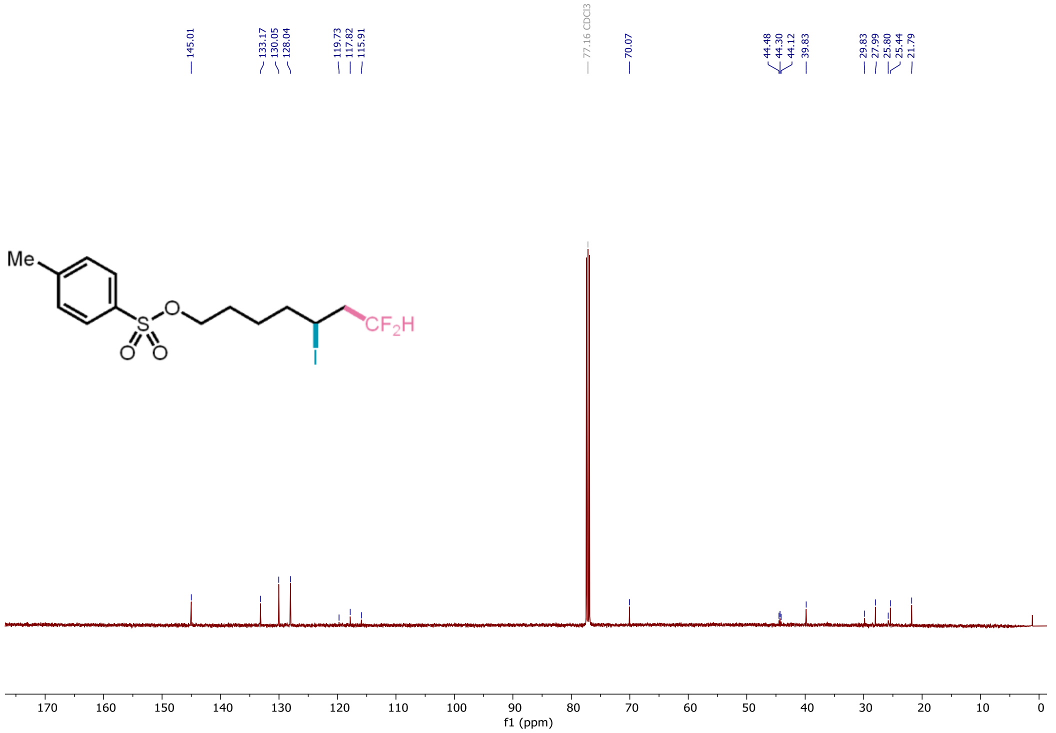

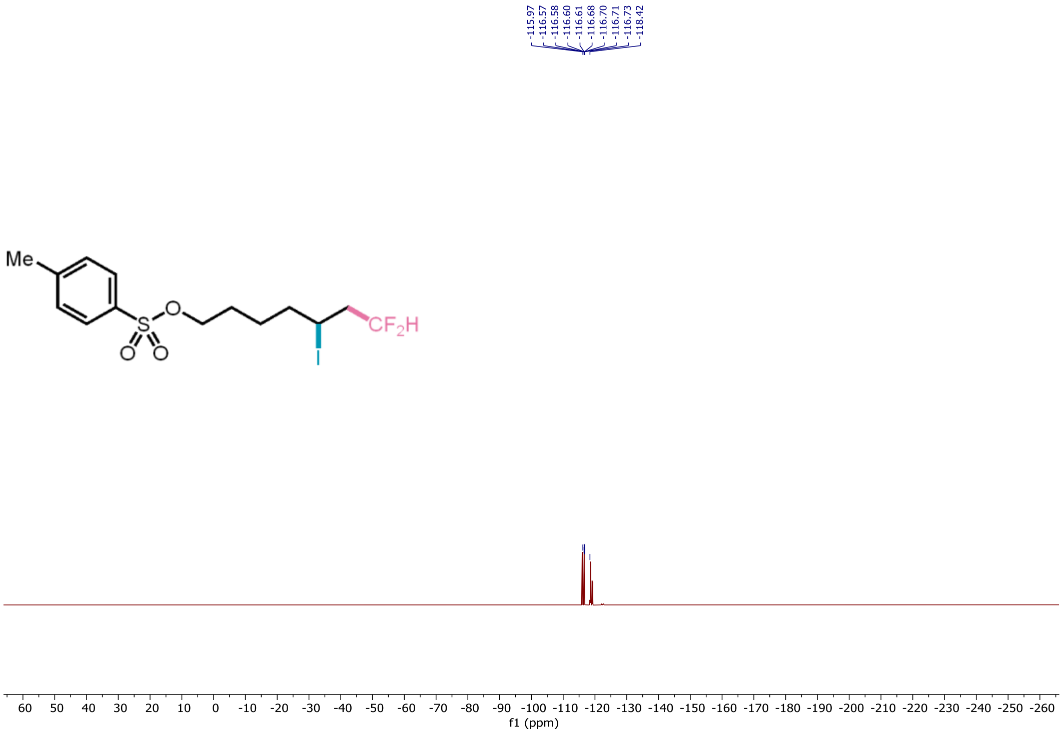

Figure S99. ^{1}H NMR (500 MHz, $CDCl_3$), ^{13}C NMR (101 MHz, $CDCl_3$) and ^{19}F NMR (376 MHz, $CDCl_3$) spectra of **44**.

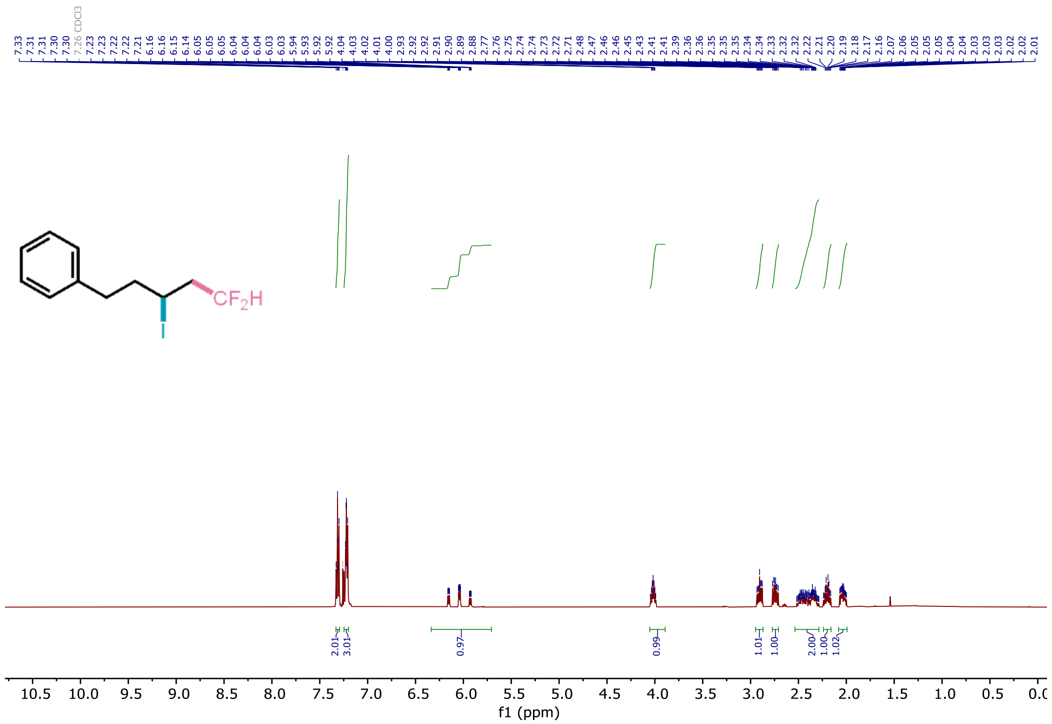

7.33 7.31 7.31 7.30 7.26 CDCl3 7.23 7.23 7.22 7.22 7.21 6.16 6.15 6.14 6.05 6.05 6.04 6.04 6.04 6.03 6.03 5.94 5.93 5.92 5.92 4.04 4.03 4.03 4.02 4.01 4.00 2.93 2.92 2.92 2.91 2.90 2.89 2.88 2.77 2.76 2.75 2.74 2.74 2.73 2.72 2.71 2.48 2.47 2.46 2.46 2.45 2.43 2.41 2.41 2.39 2.36 2.36 2.35 2.35 2.35 2.34 2.34 2.33 2.32 2.32 2.22 2.21 2.20 2.19 2.18 2.17 2.16 2.07 2.06 2.05 2.05 2.05 2.04 2.04 2.03 2.03 2.03 2.02 2.01
CF2H
I
2.01 3.01 0.97 0.99 1.01 1.00 2.00 1.00 1.02
10.5 10.0 9.5 9.0 8.5 8.0 7.5 7.0 6.5 6.0 5.5 5.0 4.5 4.0 3.5 3.0 2.5 2.0 1.5 1.0 0.5 0.0
f1 (ppm)

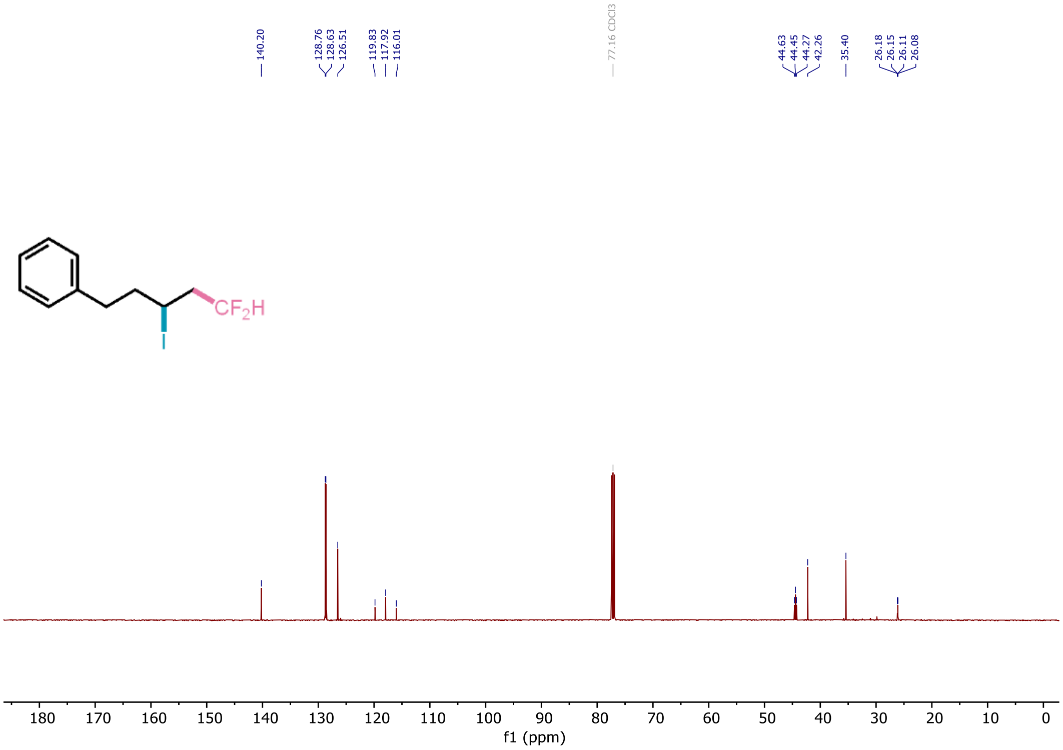

140.20 128.76 128.63 126.51 119.83 117.92 116.01 77.16 CDCl3 44.63 44.45 44.27 42.26 35.40 26.18 26.15 26.11 26.08
CF2H
I
180 170 160 150 140 130 120 110 100 90 80 70 60 50 40 30 20 10 0
f1 (ppm)

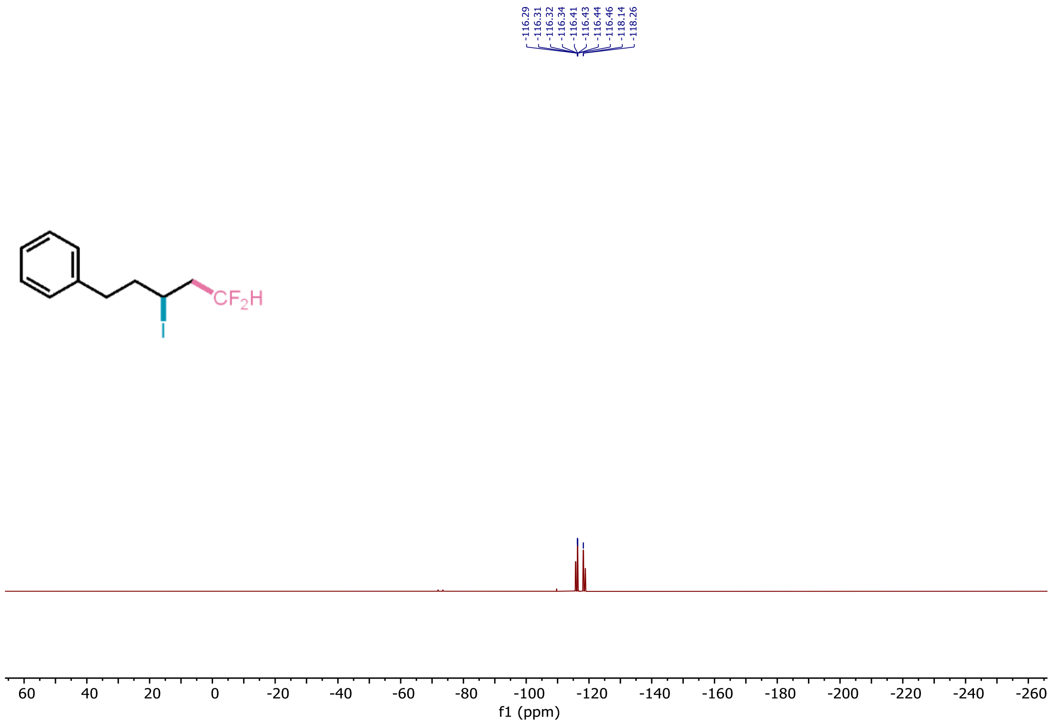

Figure S100. ^{1}H NMR (500 MHz, $CDCl_3$), ^{13}C NMR (101 MHz, $CDCl_3$) and ^{19}F NMR (376 MHz, $CDCl_3$) spectra of **45**.

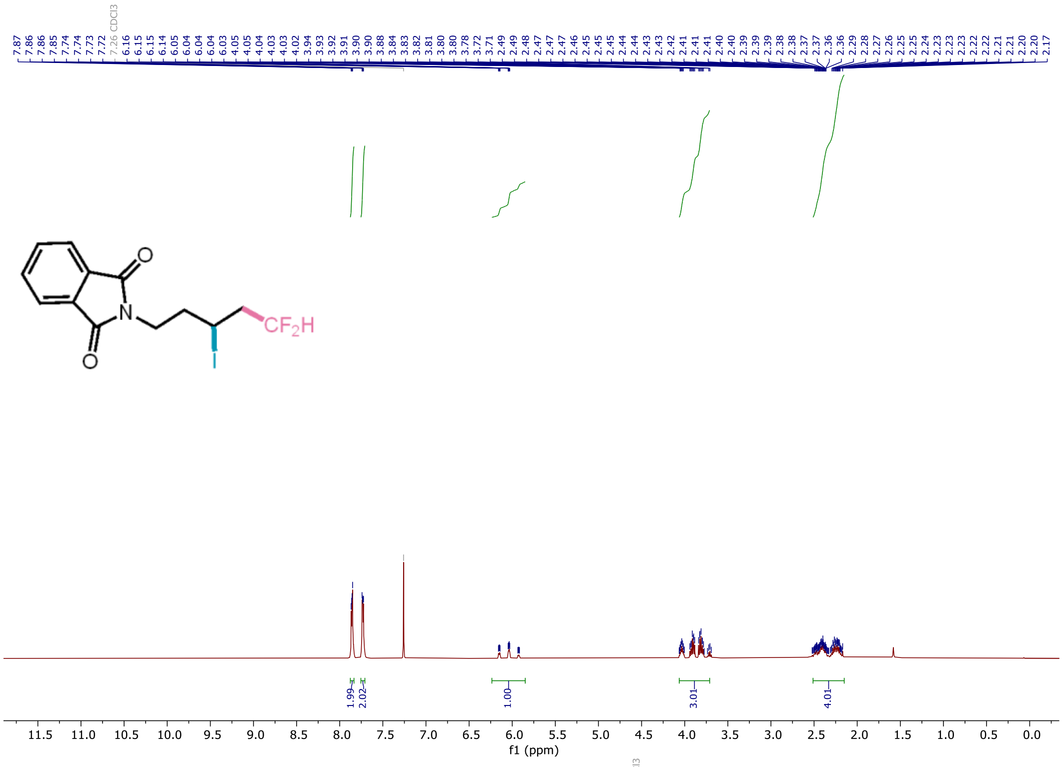

7.87 7.86 7.85 7.74 7.74 7.73 7.72 7.26 CDCl3 6.16 6.15 6.15 6.14 6.05 6.04 6.04 6.04 6.03 4.05 4.05 4.04 4.03 4.03 4.02 3.94 3.93 3.92 3.91 3.90 3.90 3.88 3.84 3.83 3.82 3.81 3.80 3.80 3.78 3.72 3.71 2.49 2.49 2.48 2.47 2.47 2.46 2.45 2.45 2.45 2.44 2.43 2.43 2.42 2.41 2.41 2.41 2.40 2.40 2.39 2.39 2.39 2.38 2.37 2.37 2.36 2.36 2.29 2.28 2.27 2.26 2.25 2.24 2.23 2.23 2.23 2.22 2.22 2.21 2.21 2.20 2.17
O
N
O
I
CF2H
1.99
2.02
1.00
3.01
4.01
11.5 11.0 10.5 10.0 9.5 9.0 8.5 8.0 7.5 7.0 6.5 6.0 5.5 5.0 4.5 4.0 3.5 3.0 2.5 2.0 1.5 1.0 0.5 0.0
f1 (ppm)

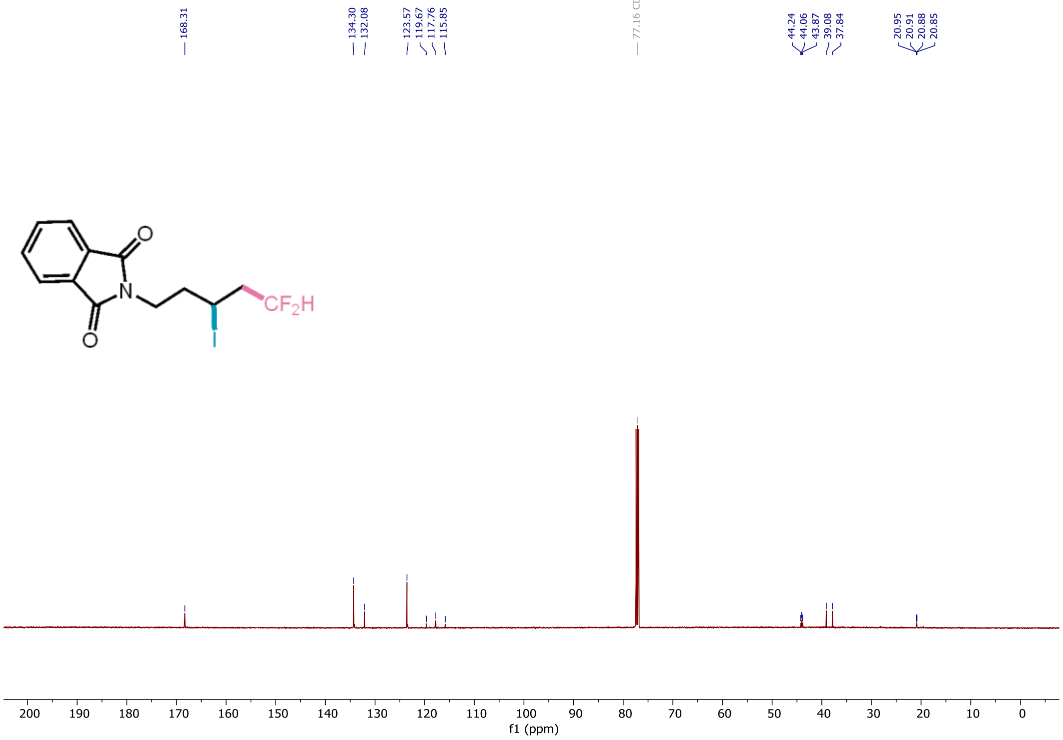

168.31
134.30 132.08
123.57 119.67 117.76 115.85
77.16 CDCl3
44.24 44.06 43.87 39.08 37.84
20.95 20.91 20.88 20.85
O
N
O
I
CF2H
200 190 180 170 160 150 140 130 120 110 100 90 80 70 60 50 40 30 20 10 0
f1 (ppm)

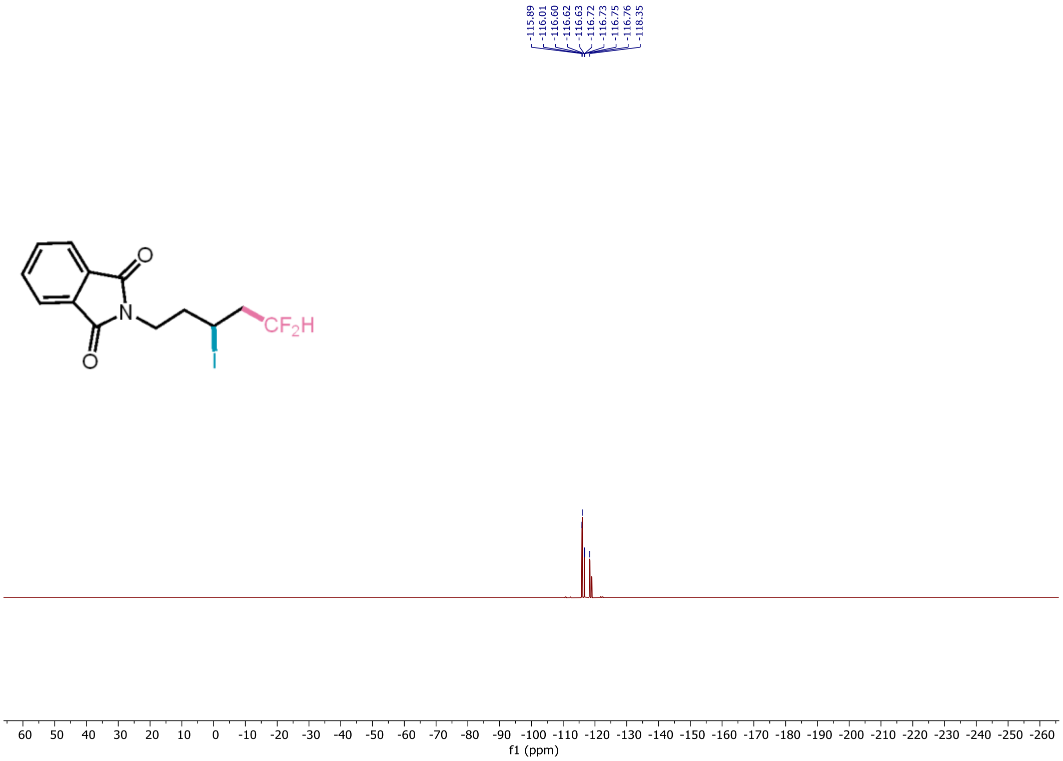

Figure S101. ^{1}H NMR (500 MHz, $CDCl_3$), ^{13}C NMR (101 MHz, $CDCl_3$) and ^{19}F NMR (376 MHz, $CDCl_3$) spectra of **46**.

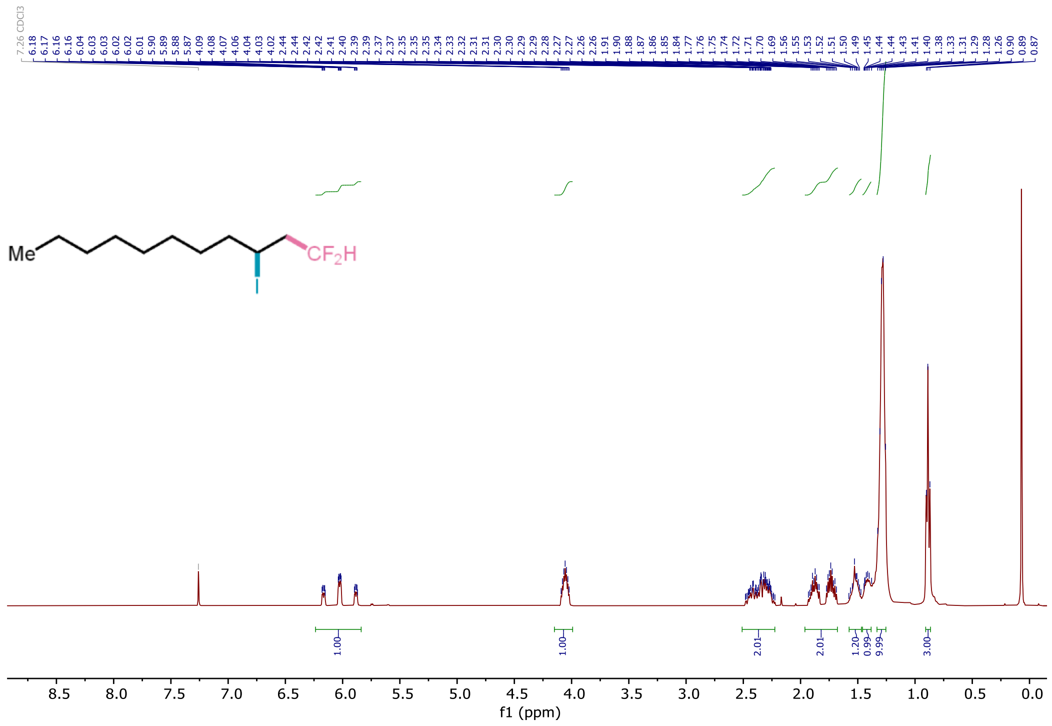

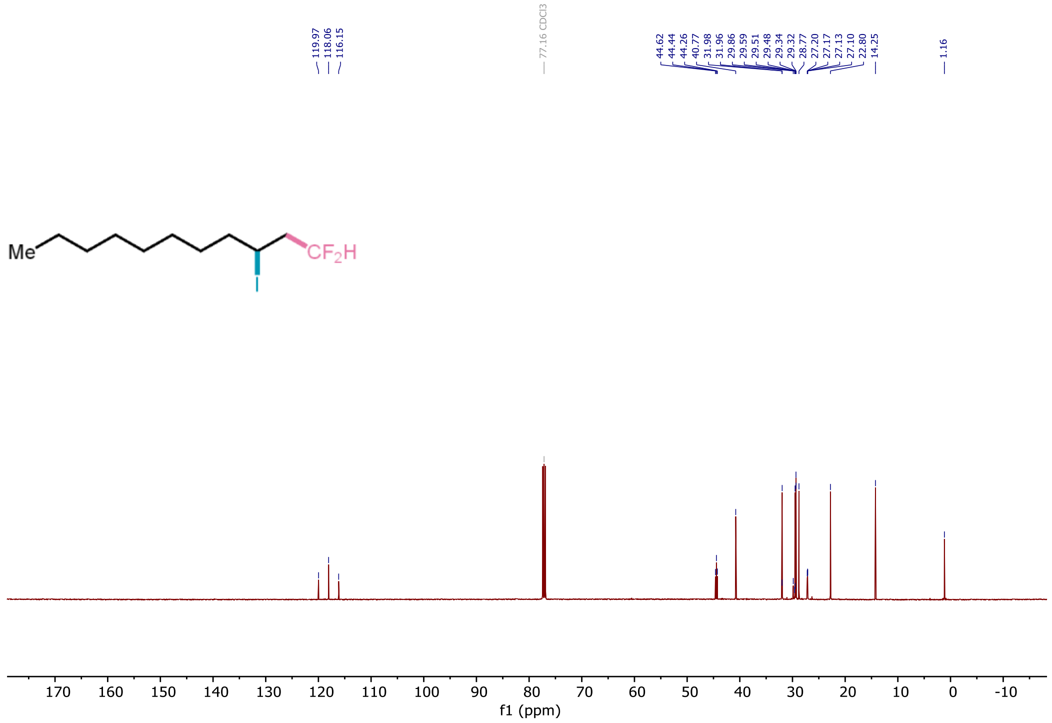

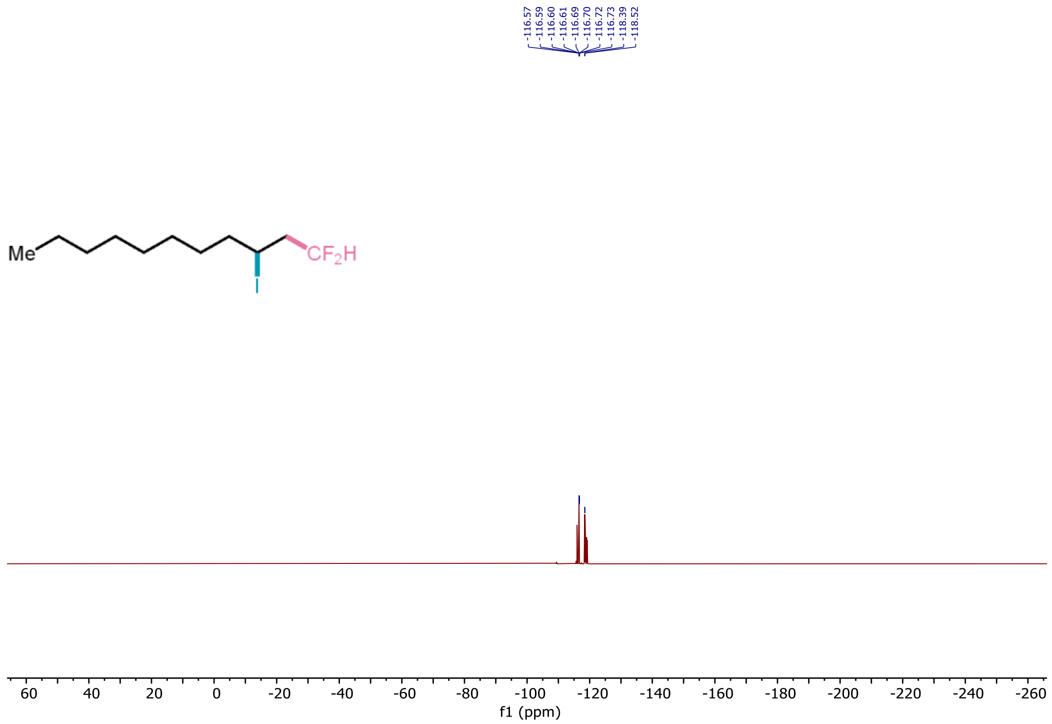

Figure S102. ^{1}H NMR (500 MHz, $CDCl_3$), ^{13}C NMR (101 MHz, $CDCl_3$) and ^{19}F NMR (376 MHz, $CDCl_3$) spectra of **47**.

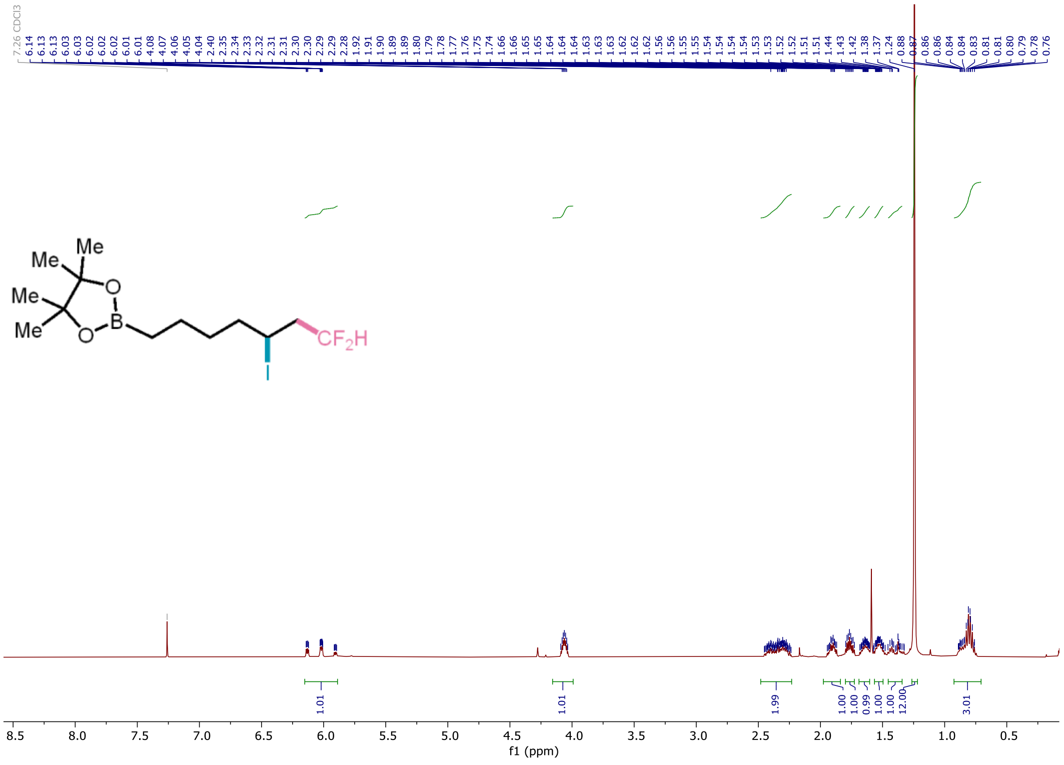

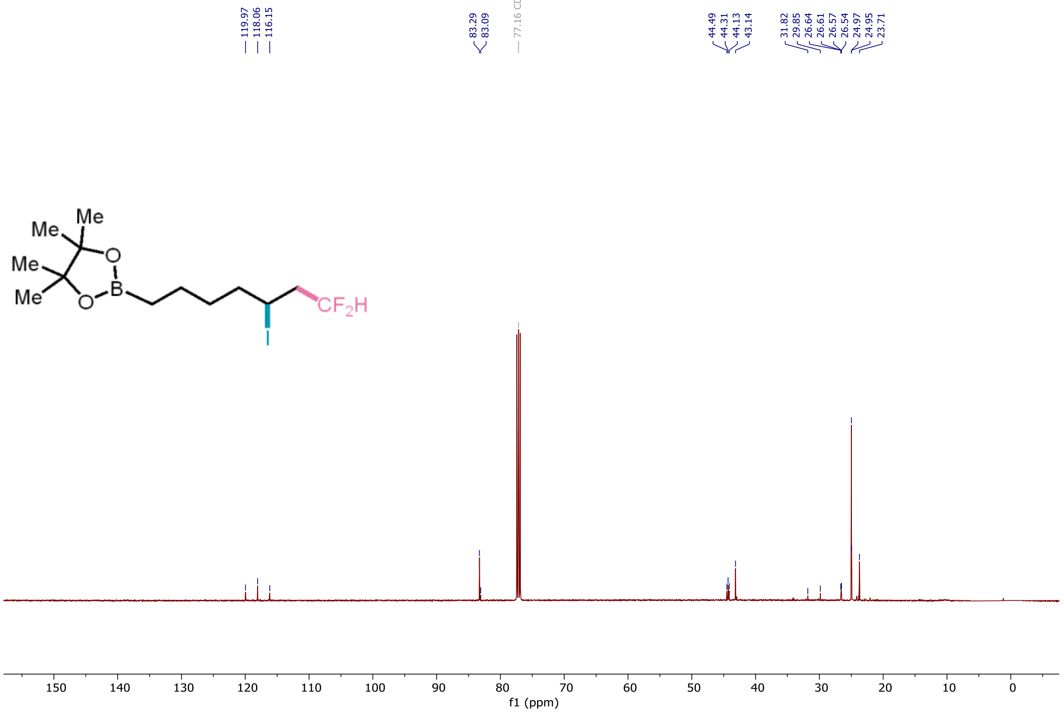

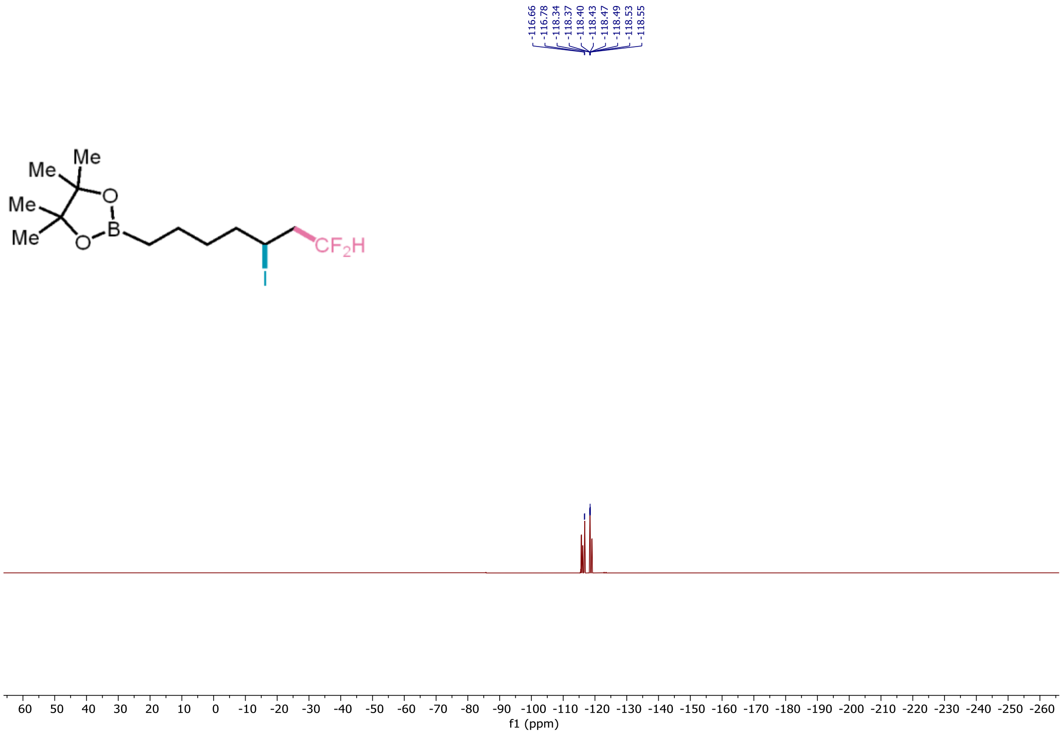

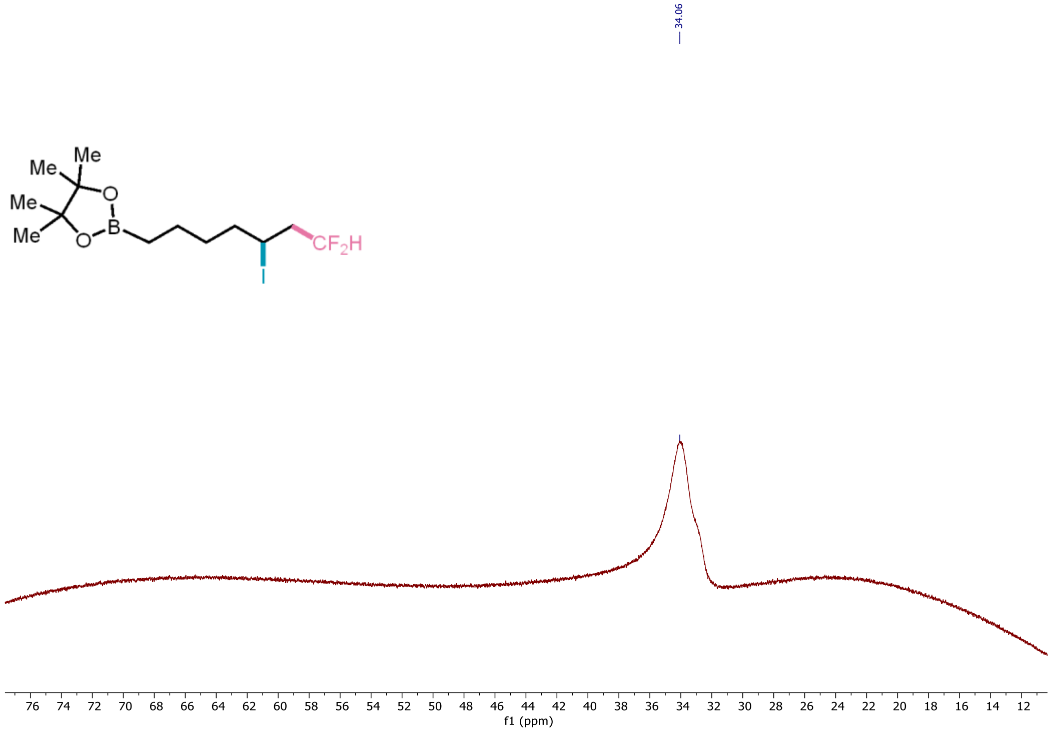

Figure S103. ^{1}H NMR (500 MHz, $CDCl_3$), ^{13}C NMR (101 MHz, $CDCl_3$), ^{19}F NMR (376 MHz, $CDCl_3$) and ^{11}B NMR (160 MHz, $CDCl_3$) spectra of **48**.

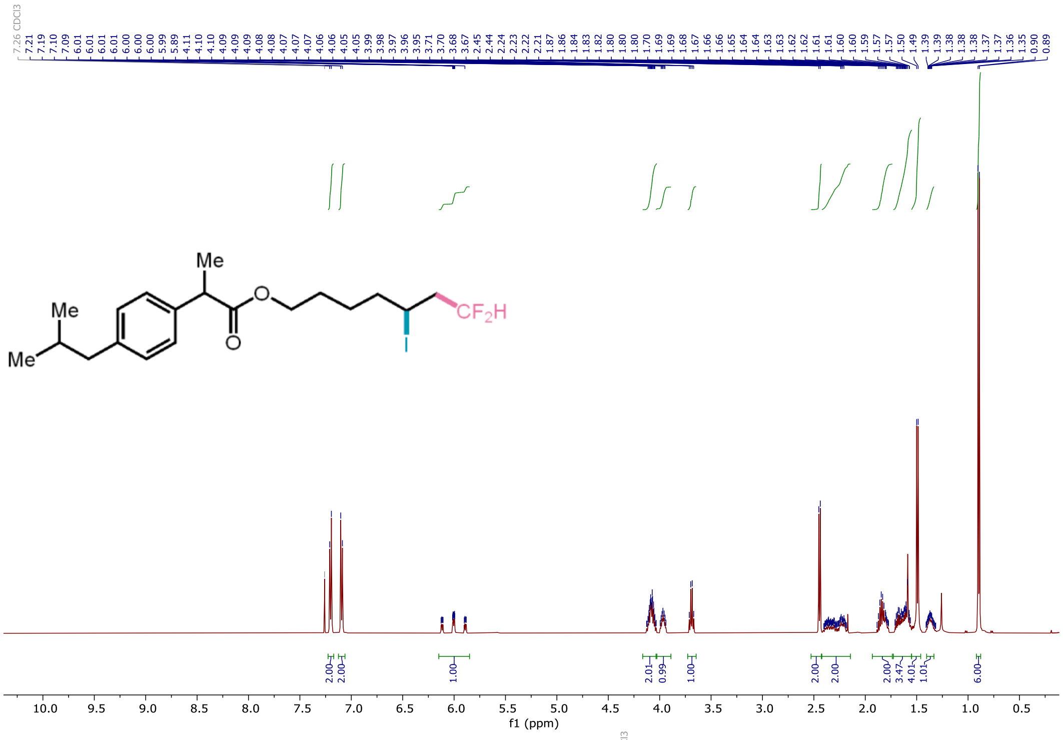

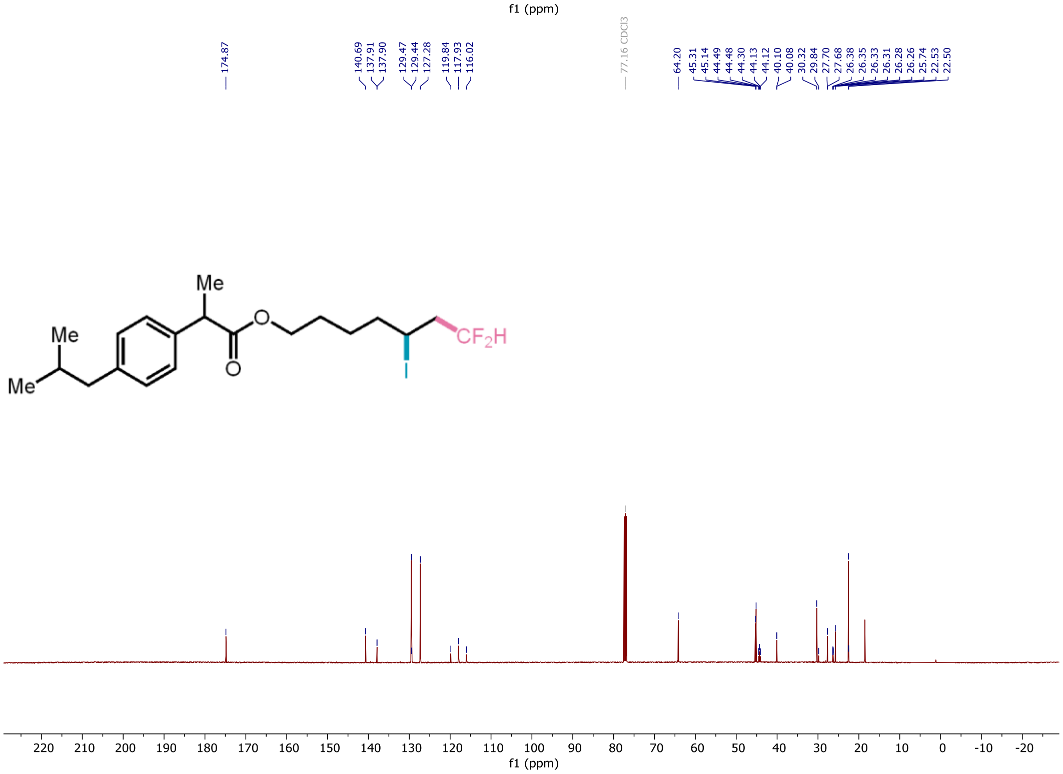

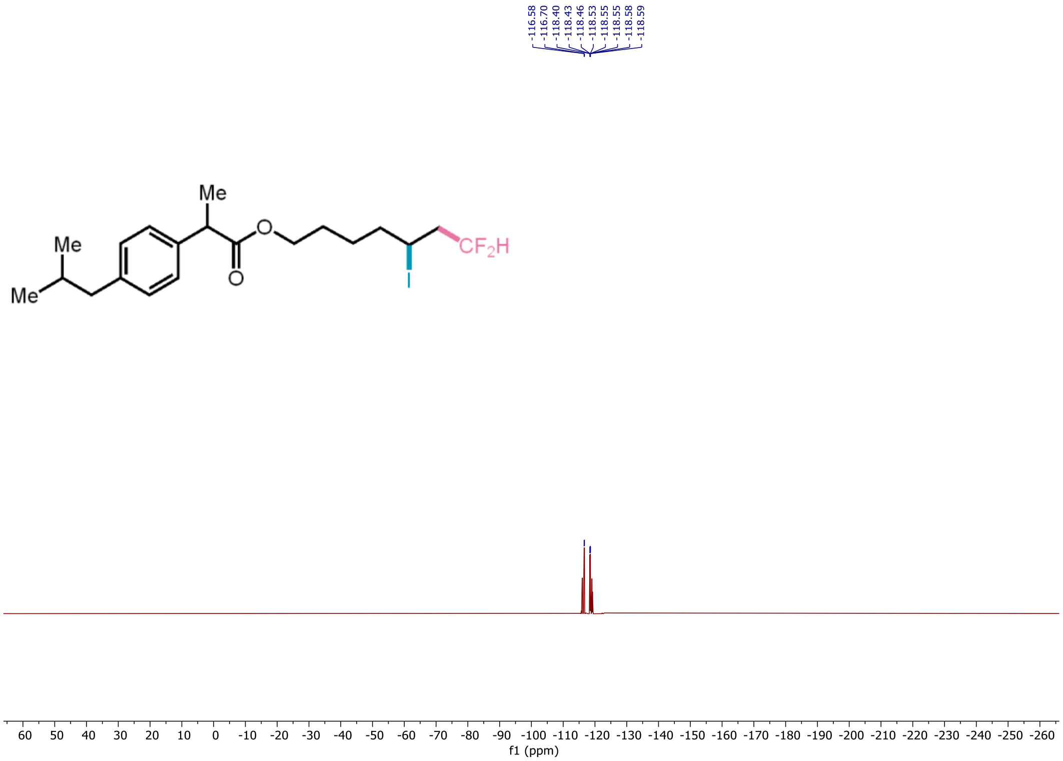

Figure S104. ^{1}H NMR (500 MHz, $CDCl_3$), ^{13}C NMR (101 MHz, $CDCl_3$) and ^{19}F NMR (376 MHz, $CDCl_3$) spectra of **49**.

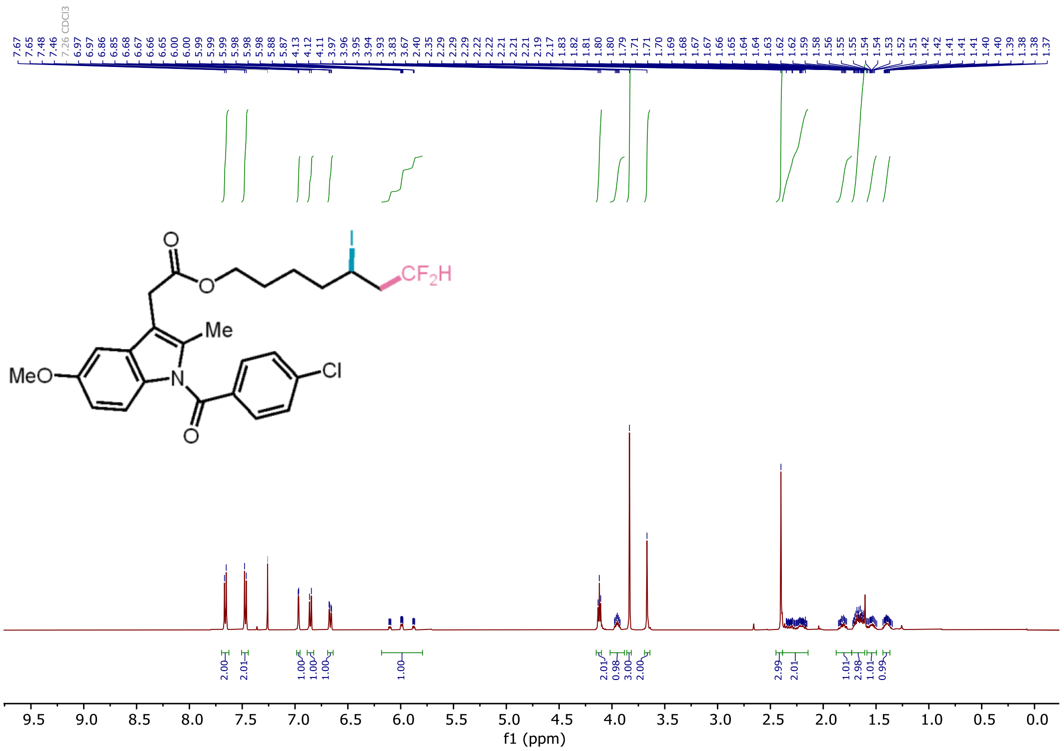
7.67 7.65 7.48 7.46 7.26 CDCl3 6.97 6.97 6.86 6.85 6.68 6.67 6.66 6.65 6.00 6.00 5.99 5.99 5.99 5.98 5.98 5.98 5.88 5.87 4.13 4.12 4.11 3.97 3.96 3.95 3.94 3.93 3.83 3.67 2.40 2.35 2.29 2.29 2.29 2.22 2.21 2.21 2.21 2.19 2.17 1.83 1.81 1.80 1.79 1.71 1.70 1.69 1.68 1.67 1.66 1.65 1.64 1.64 1.63 1.62 1.62 1.59 1.58 1.56 1.55 1.55 1.54 1.54 1.53 1.52 1.51 1.42 1.41 1.41 1.40 1.40 1.39 1.38 1.37
O
O
CF2H
Me
MeO
N
Cl
O
2.00 2.01 1.00 1.00 1.00 1.00 2.01 0.98 3.00 2.00 2.99 2.01 1.01 2.98 1.01 0.99
9.5 9.0 8.5 8.0 7.5 7.0 6.5 6.0 5.5 5.0 4.5 4.0 3.5 3.0 2.5 2.0 1.5 1.0 0.5 0.0
f1 (ppm)

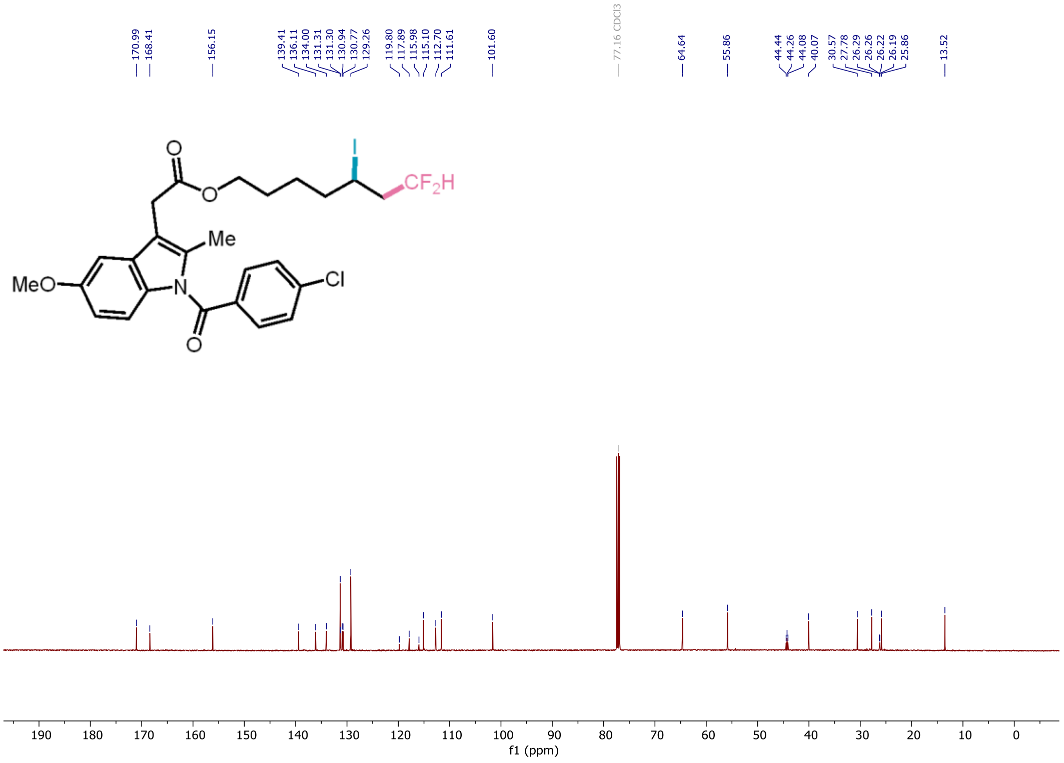
170.99 168.41 156.15 139.41 136.11 134.00 131.31 131.30 130.94 130.77 129.26 119.80 117.89 115.98 115.10 112.70 111.61 101.60 77.16 CDCl3 64.64 55.86 44.44 44.26 44.08 40.07 30.57 27.78 26.29 26.26 26.22 26.19 25.86 13.52
O
O
CF2H
Me
MeO
N
Cl
O
190 180 170 160 150 140 130 120 110 100 90 80 70 60 50 40 30 20 10 0
f1 (ppm)

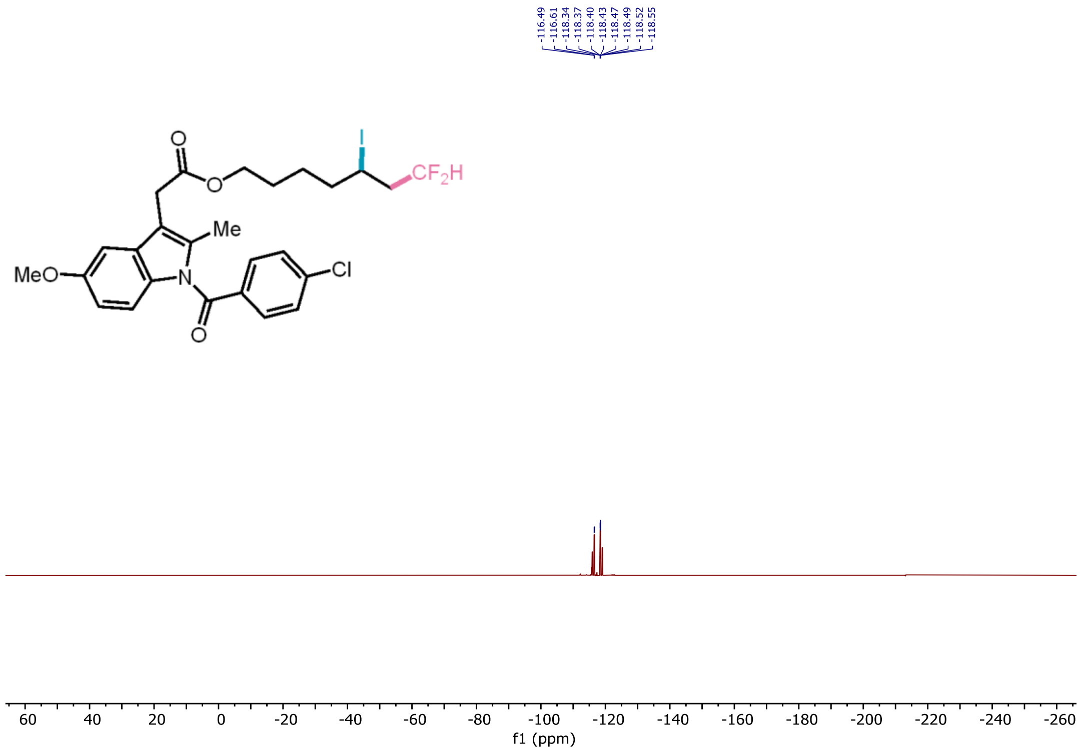

Figure S105. ^{1}H NMR (500 MHz, $CDCl_3$), ^{13}C NMR (101 MHz, $CDCl_3$) and ^{19}F NMR (376 MHz, $CDCl_3$) spectra of **50**.

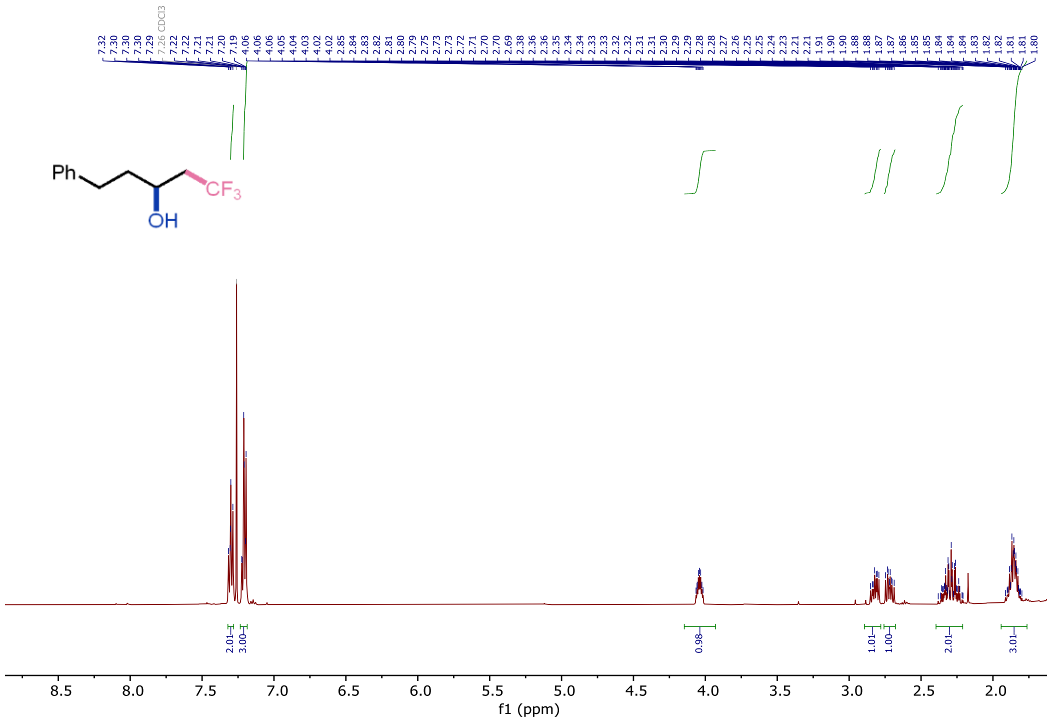
Ph
OH
CF3
2.01
3.00
0.98
1.01
1.00
2.01
3.01
f1 (ppm)

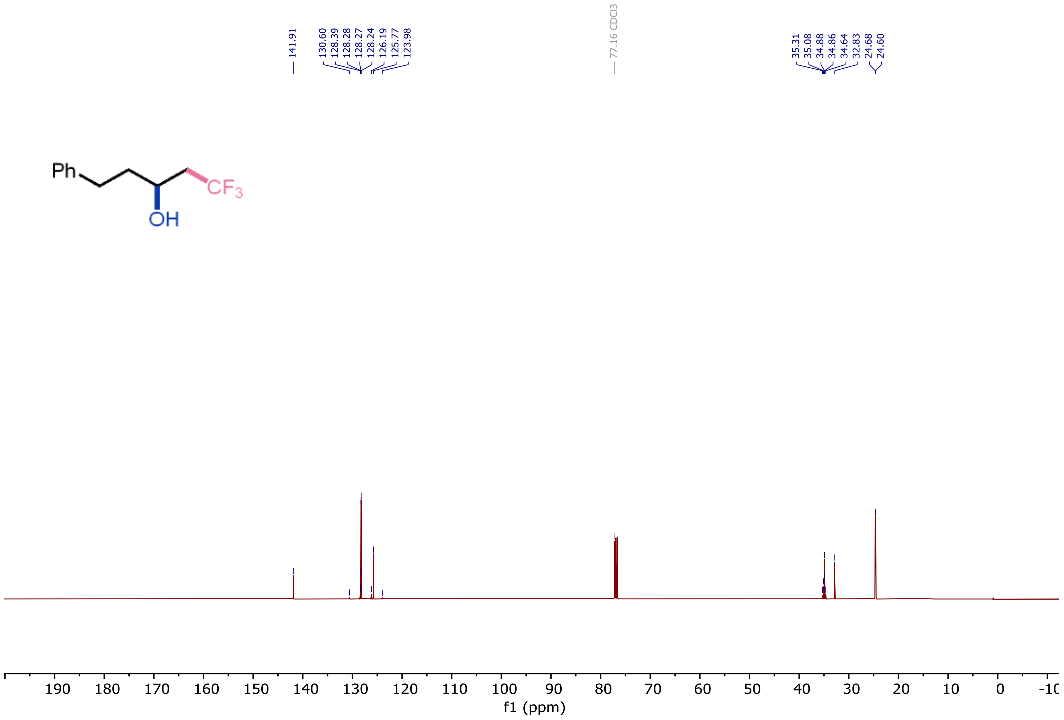
141.91
130.60
128.39
128.28
128.27
128.24
126.19
125.77
123.98
77.16 CDCl3
35.31
35.08
34.88
34.86
34.64
32.83
24.68
24.60
Ph
OH
CF3
f1 (ppm)

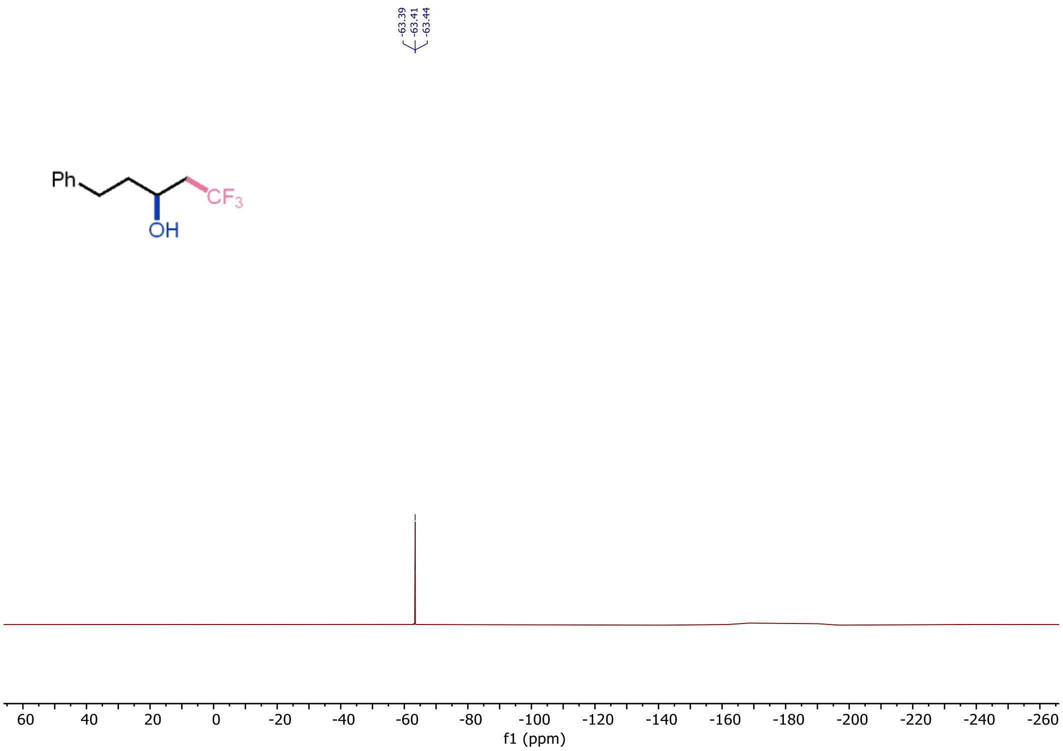

Figure S106. ^{1}H NMR (500 MHz, $CDCl_3$), ^{13}C NMR (101 MHz, $CDCl_3$) and ^{19}F NMR (376 MHz, $CDCl_3$) spectra of **51**.

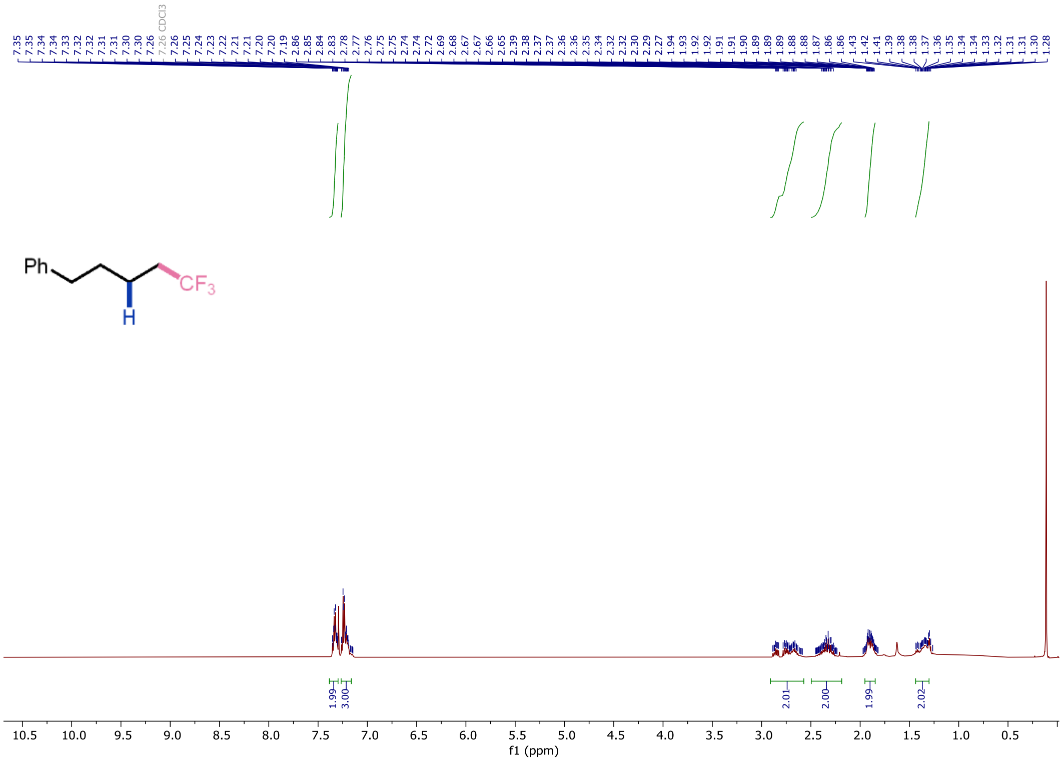
Ph
H
CF3
7.26 CDCl3
1.99
3.00
2.01
2.00
1.99
2.02
f1 (ppm)

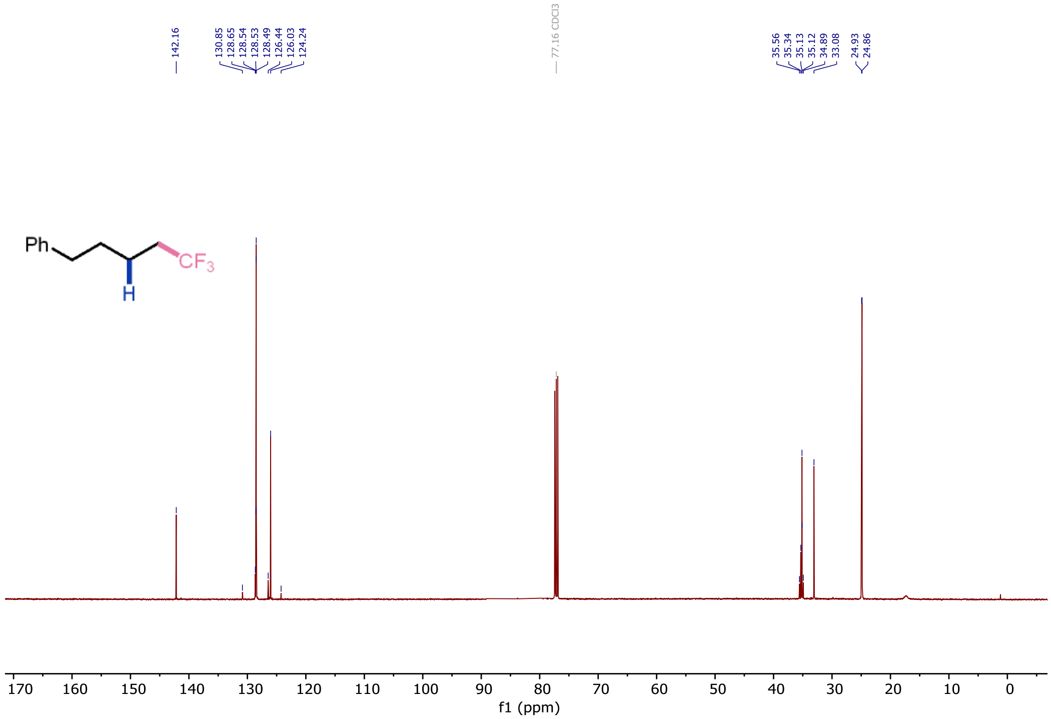
142.16
130.85
128.65
128.54
128.53
128.49
126.44
126.03
124.24
77.16 CDCl3
35.56
35.34
35.13
35.12
34.89
33.08
24.93
24.86
Ph
H
CF3
f1 (ppm)

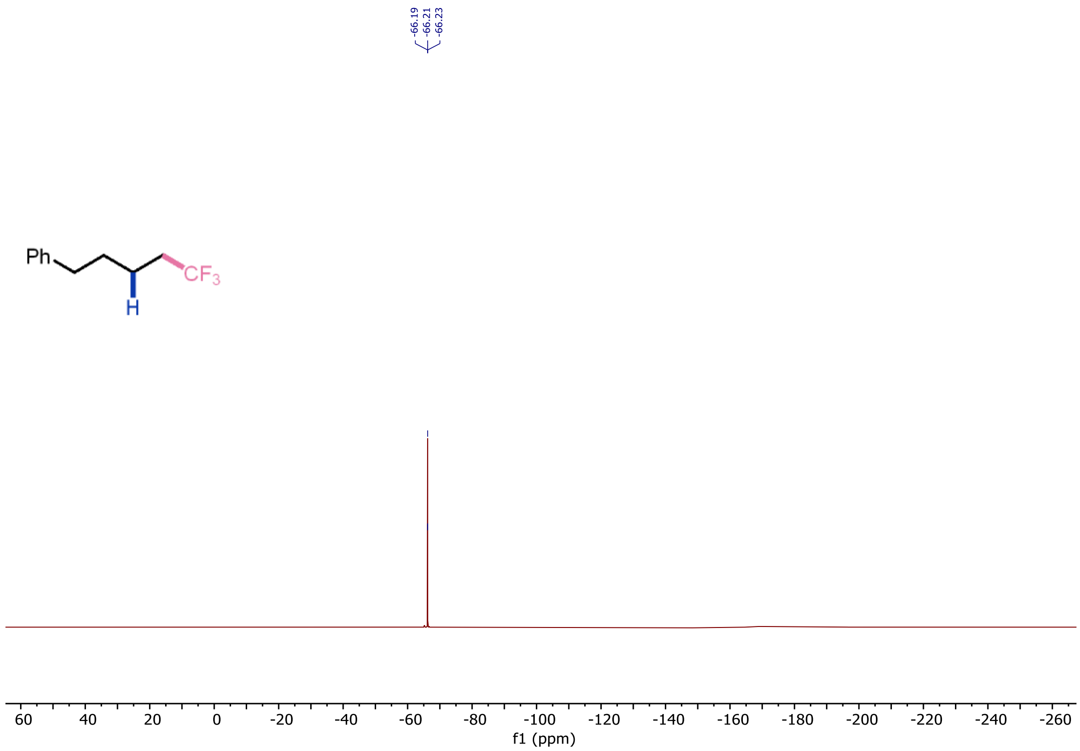

Figure S107. ^{1}H NMR (500 MHz, $CDCl_3$), ^{13}C NMR (101 MHz, $CDCl_3$) and ^{19}F NMR (376 MHz, $CDCl_3$) spectra of **52**.

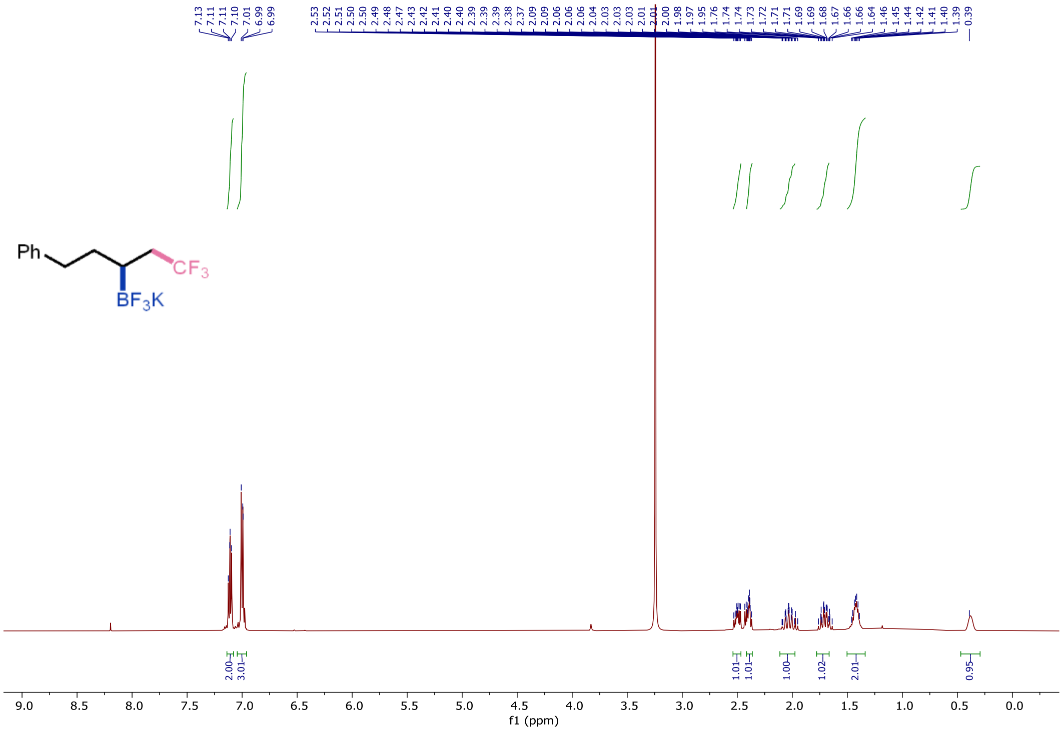

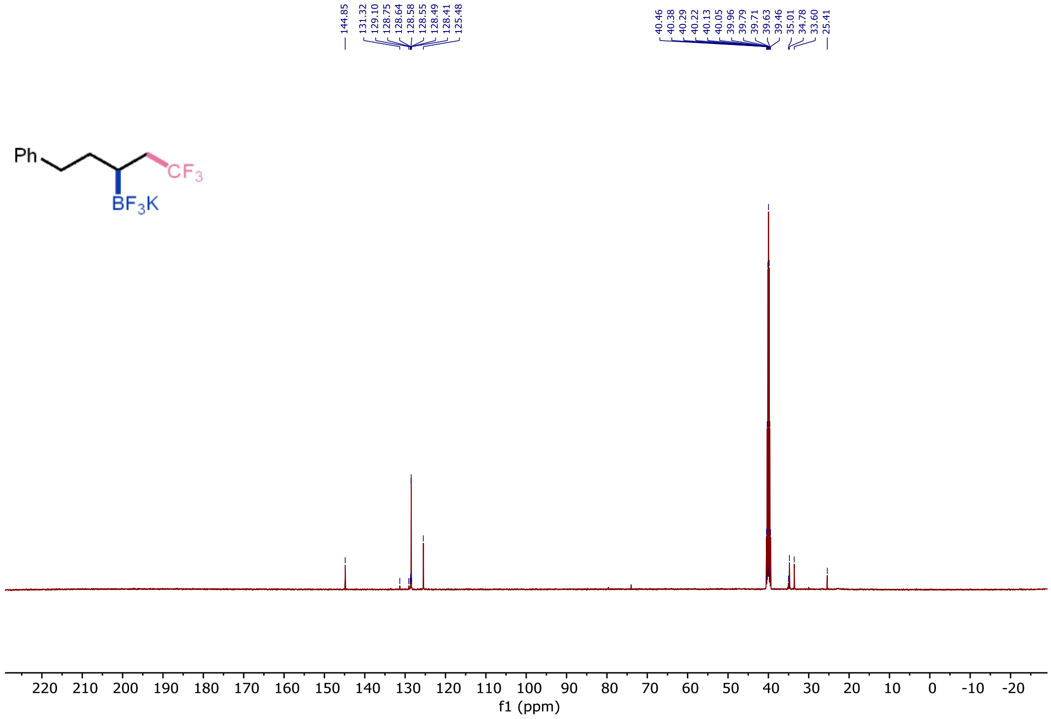

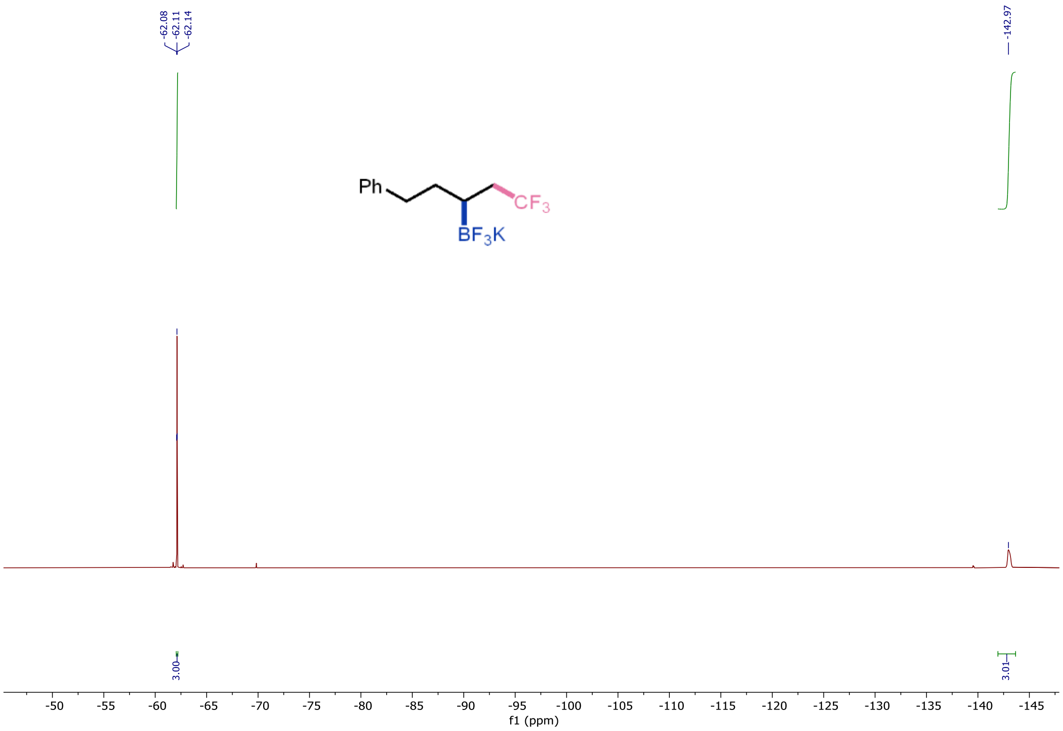

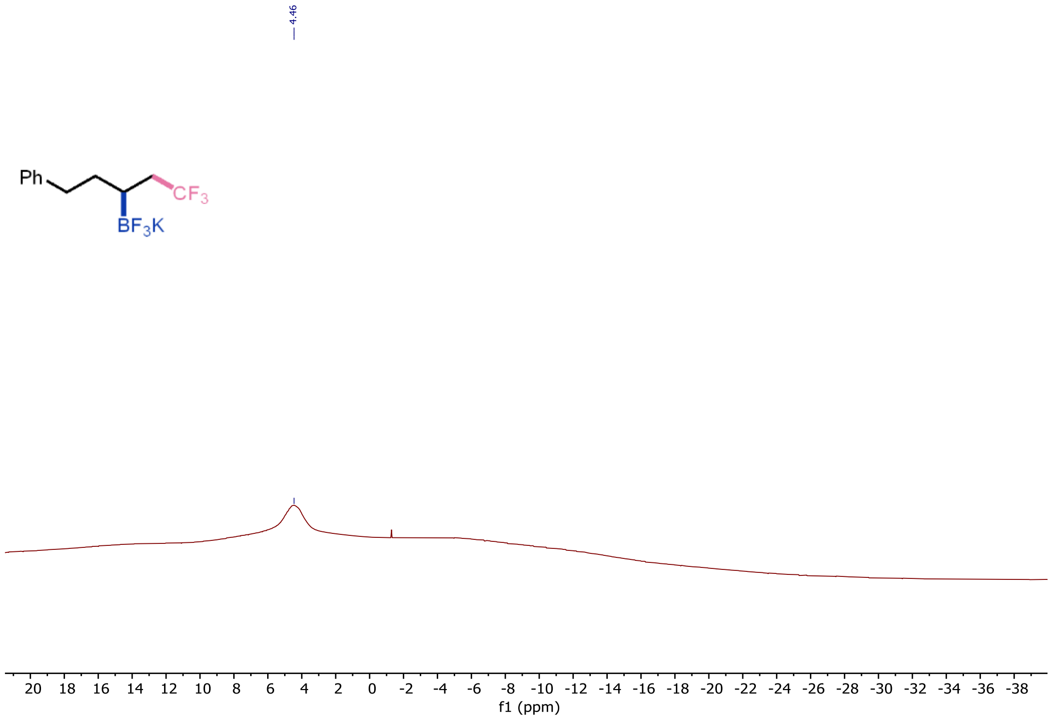

Figure S108. ^{1}H NMR (500 MHz, $CDCl_3$), ^{13}C NMR (101 MHz, $CDCl_3$), ^{19}F NMR (376 MHz, $CDCl_3$) and ^{11}B NMR (160 MHz, $CDCl_3$) spectra of **53**.

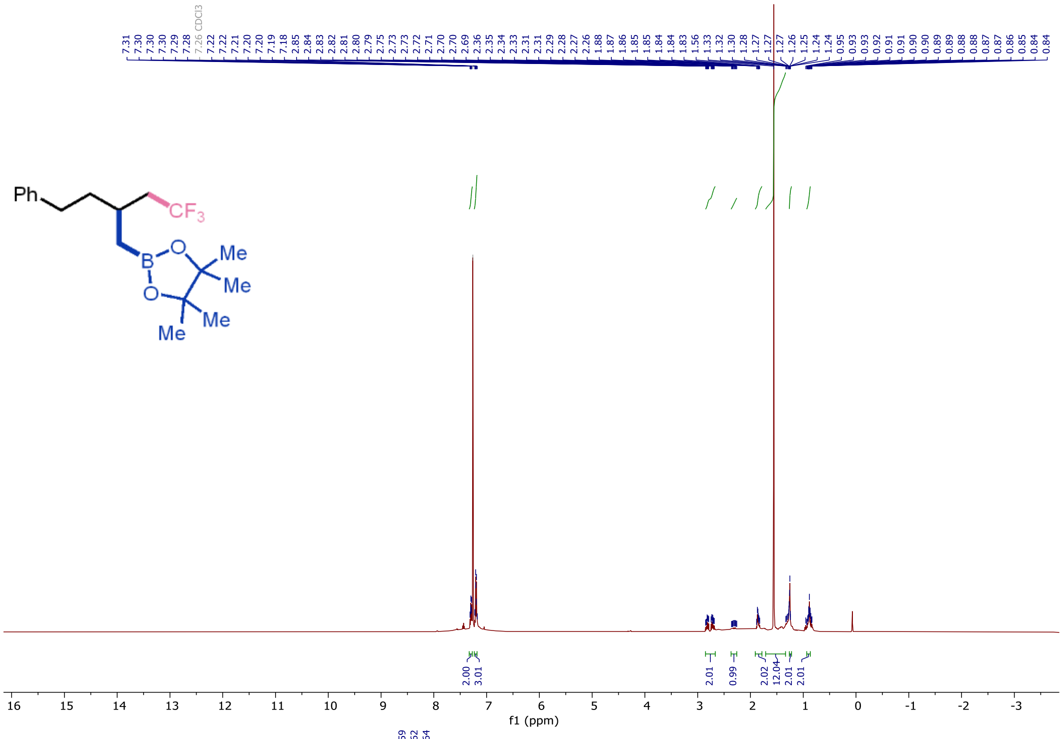

7.31 7.30 7.30 7.29 7.28 7.26 CDCl3 7.22 7.22 7.21 7.20 7.20 7.19 7.18 2.85 2.84 2.83 2.82 2.81 2.80 2.79 2.75 2.75 2.73 2.73 2.72 2.71 2.70 2.69 2.36 2.35 2.34 2.33 2.31 2.31 2.29 2.28 2.27 2.26 1.88 1.87 1.86 1.85 1.85 1.84 1.84 1.83 1.56 1.33 1.32 1.30 1.28 1.27 1.27 1.26 1.25 1.24 1.24 0.95 0.93 0.93 0.92 0.91 0.90 0.90 0.89 0.89 0.88 0.88 0.87 0.86 0.85 0.84 0.84
Ph
CF3
B
O
O
Me
Me
Me
Me
2.00
3.01
2.01
0.99
2.02
12.04
2.01
2.01
16 15 14 13 12 11 10 9 8 7 6 5 4 3 2 1 0 -1 -2 -3
f1 (ppm)

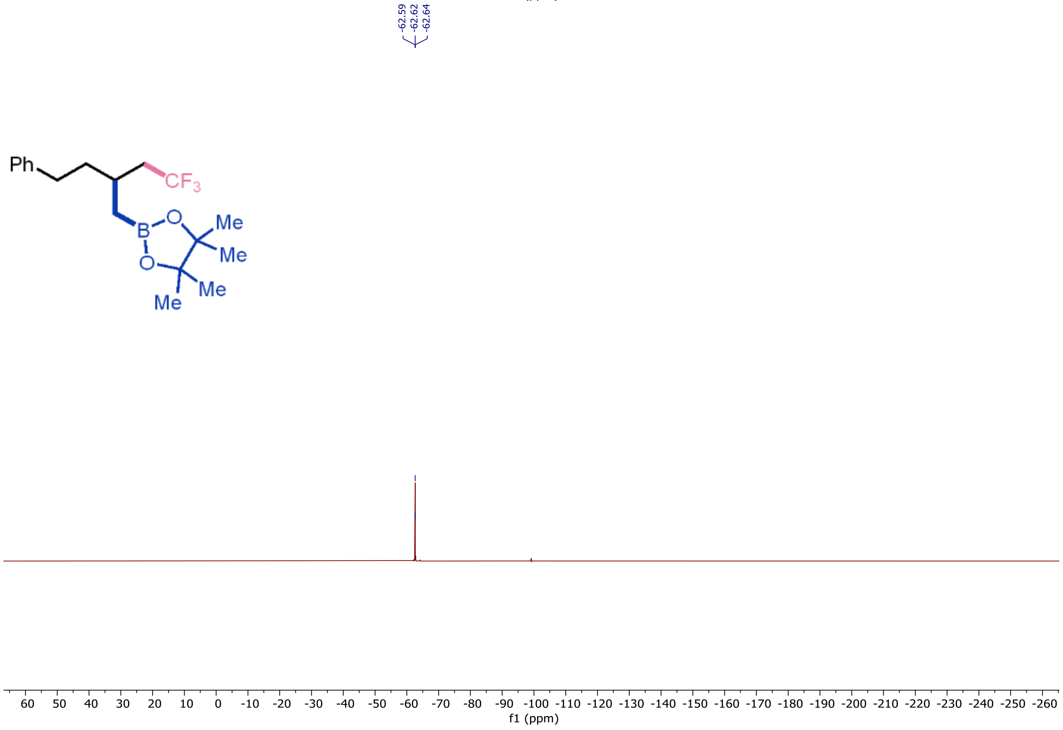

-62.59
-62.62
-62.64
Ph
CF3
B
O
O
Me
Me
Me
Me
60 50 40 30 20 10 0 -10 -20 -30 -40 -50 -60 -70 -80 -90 -100 -110 -120 -130 -140 -150 -160 -170 -180 -190 -200 -210 -220 -230 -240 -250 -260
f1 (ppm)

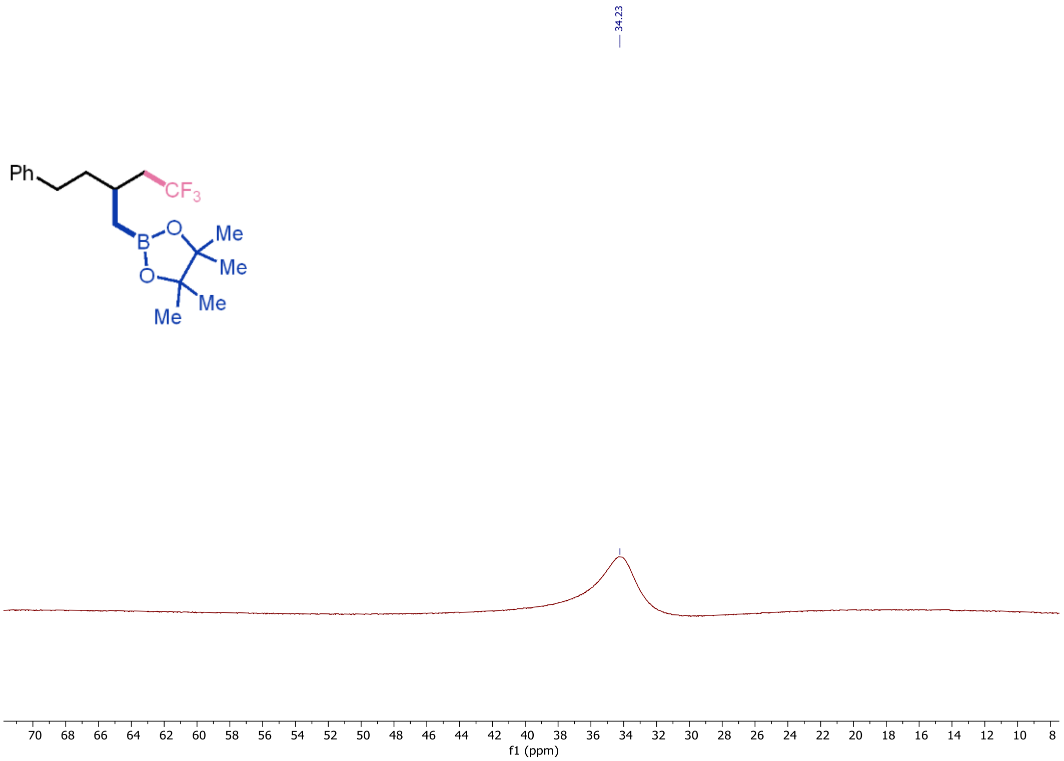

Figure S109. ^{1}H NMR (500 MHz, $CDCl_3$), ^{13}C NMR (101 MHz, $CDCl_3$), ^{19}F NMR (376 MHz, $CDCl_3$) and ^{11}B NMR (160 MHz, $CDCl_3$) spectra of **54**.

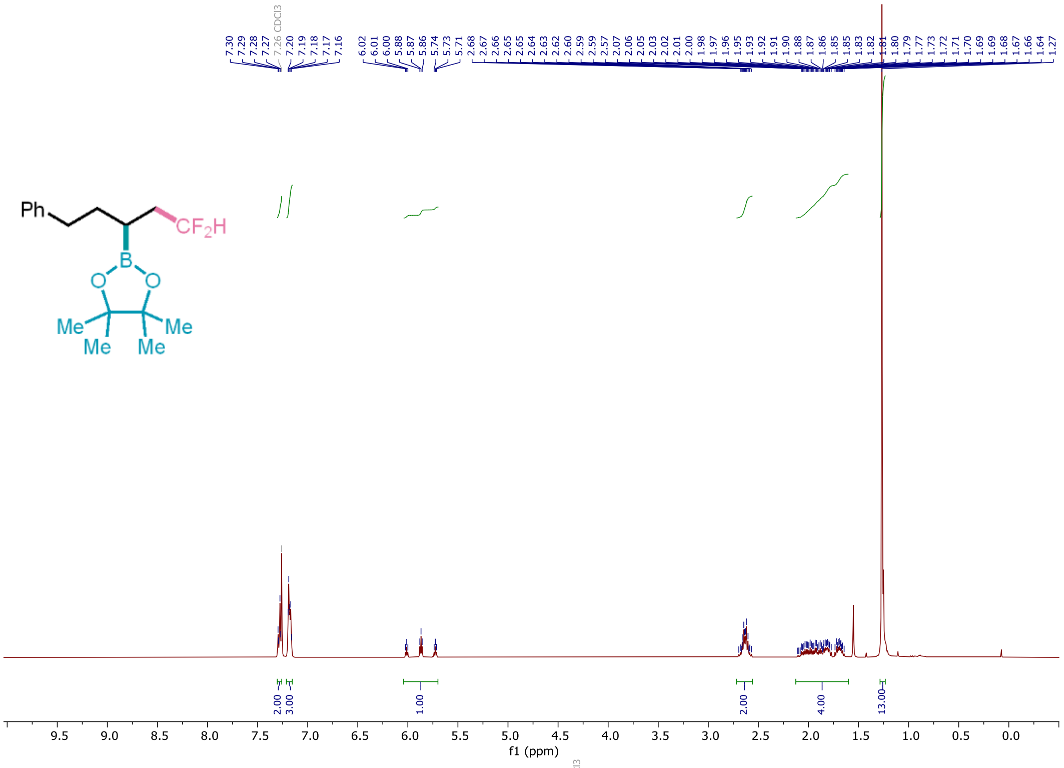

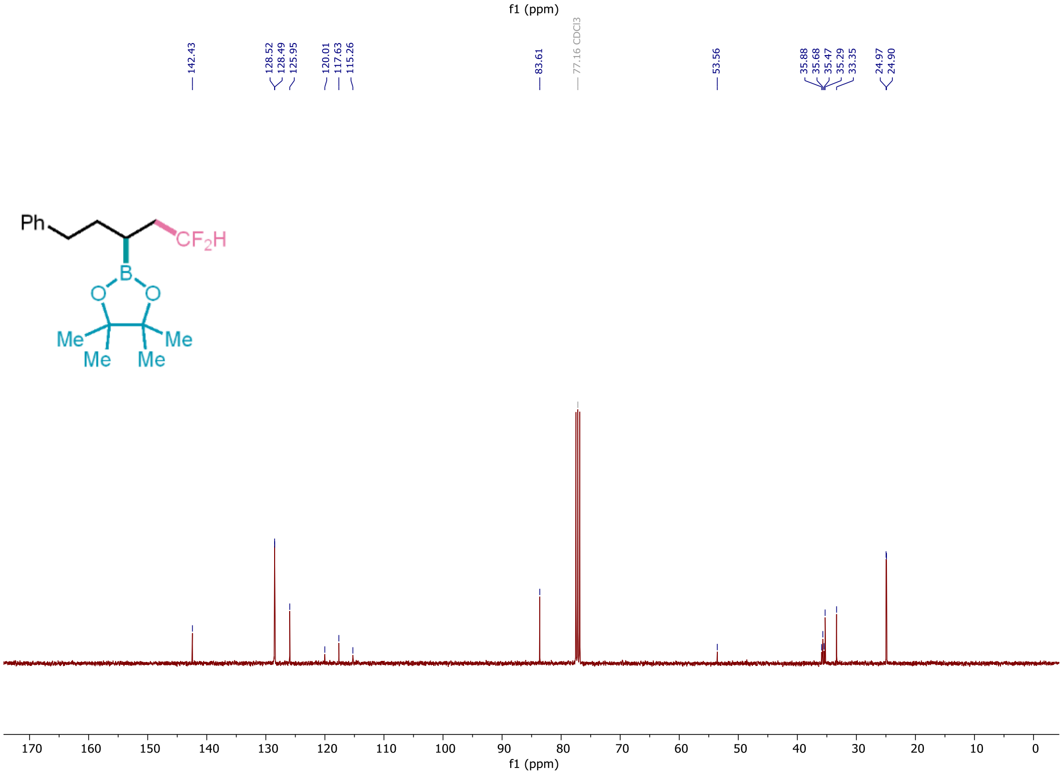

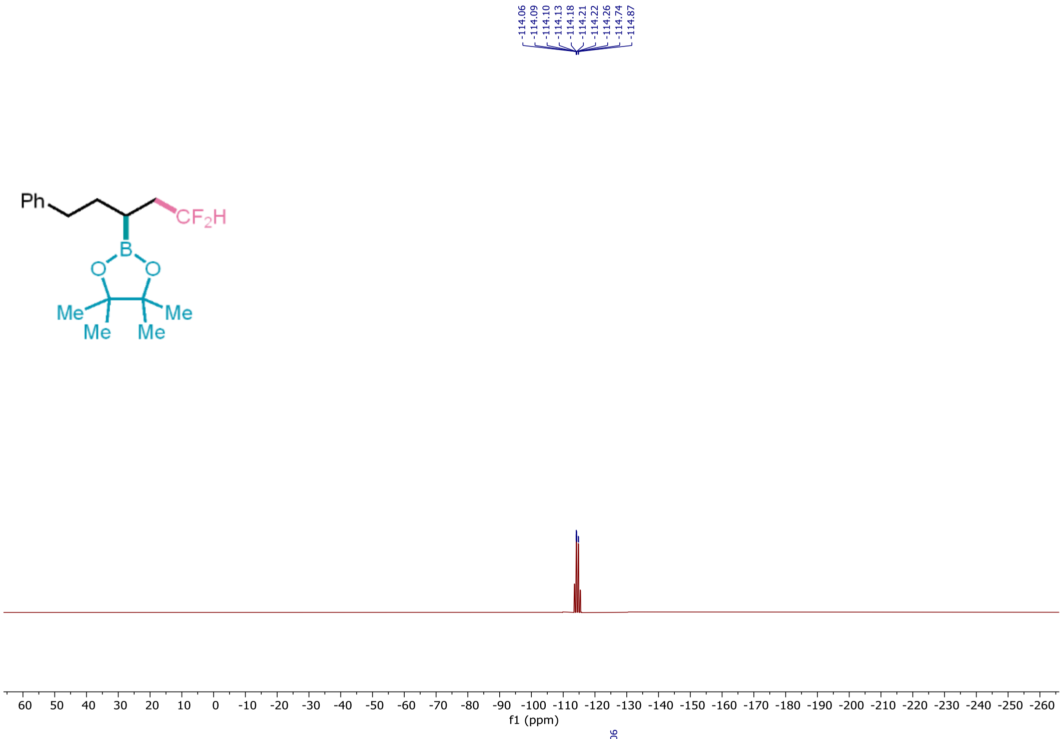

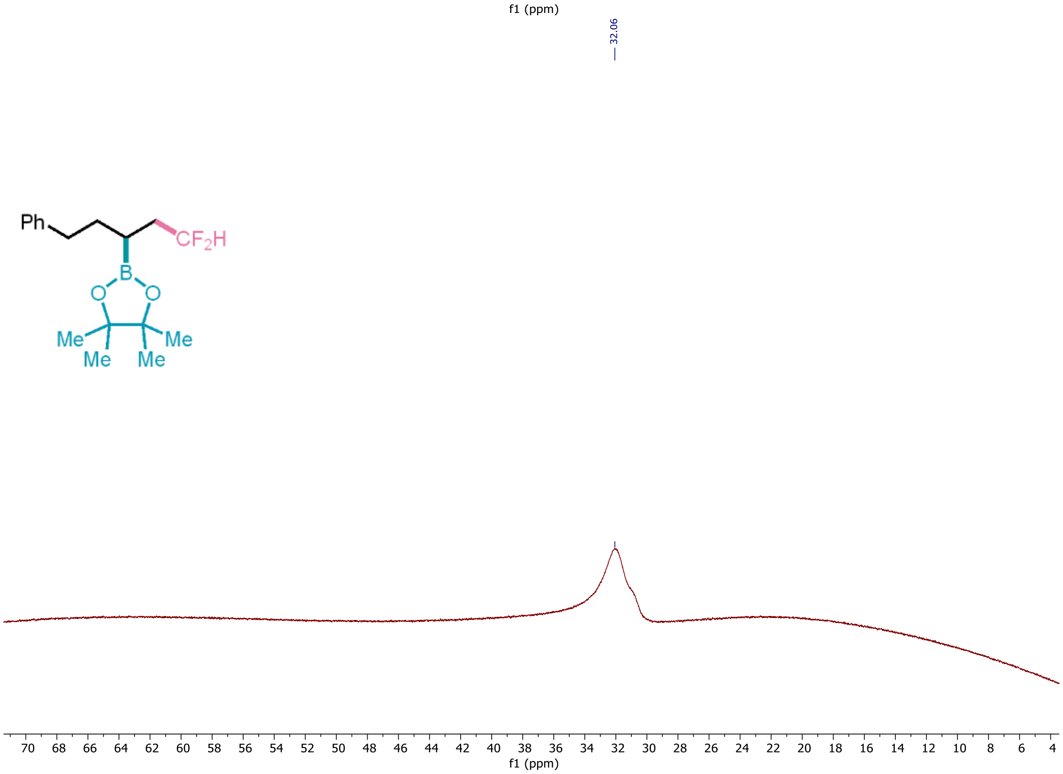

Figure S110. ^{1}H NMR (500 MHz, $CDCl_3$), ^{13}C NMR (101 MHz, $CDCl_3$), ^{19}F NMR (376 MHz, $CDCl_3$) and ^{11}B NMR (160 MHz, $CDCl_3$) spectra of **55**.

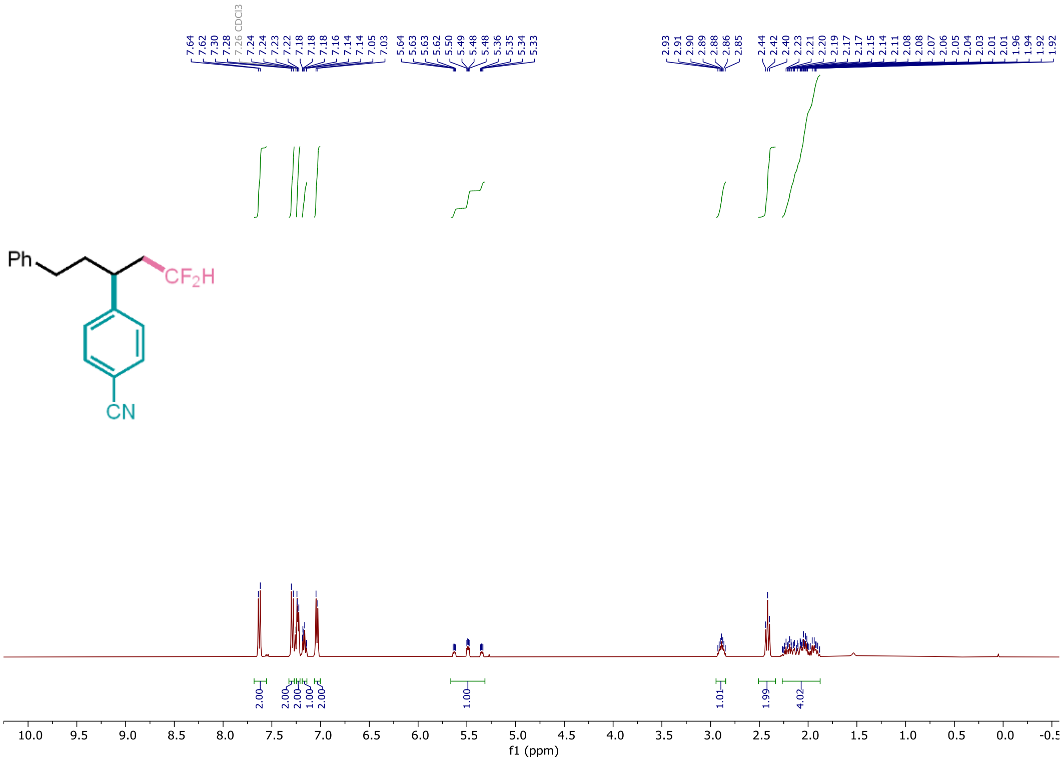
7.64 7.62 7.30 7.28 7.26 CDCl3 7.24 7.24 7.23 7.22 7.18 7.18 7.18 7.16 7.14 7.14 7.05 7.03
5.64 5.63 5.63 5.62 5.50 5.49 5.49 5.48 5.48 5.36 5.35 5.34 5.33
2.93 2.91 2.90 2.89 2.88 2.86 2.85
2.44 2.42 2.40 2.23 2.21 2.20 2.19 2.17 2.17 2.15 2.14 2.11 2.08 2.08 2.07 2.06 2.05 2.04 2.03 2.01 2.01 1.96 1.94 1.92 1.92
Ph
CF2H
CN
2.00 2.00 2.00 1.00 2.00 1.00 1.01 1.99 4.02
10.0 9.5 9.0 8.5 8.0 7.5 7.0 6.5 6.0 5.5 5.0 4.5 4.0 3.5 3.0 2.5 2.0 1.5 1.0 0.5 0.0 -0.5
f1 (ppm)

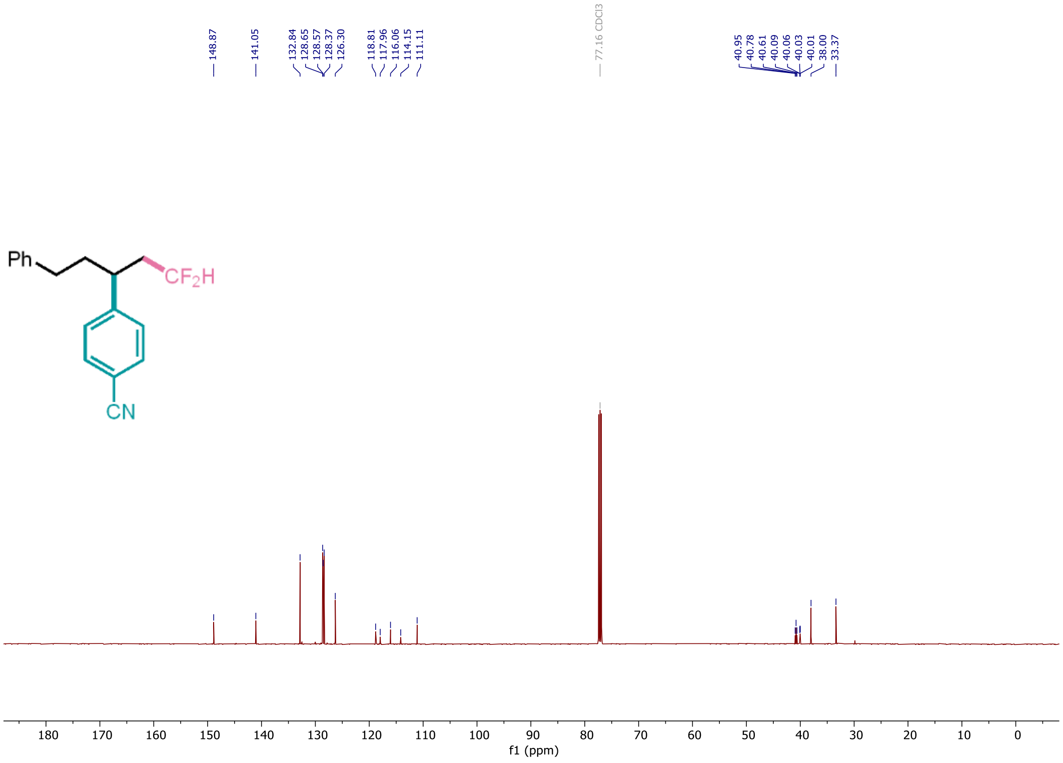
148.87
141.05
132.84 128.65 128.57 128.37 126.30
118.81 117.96 116.06 114.15 111.11
77.16 CDCl3
40.95 40.78 40.61 40.09 40.06 40.03 40.01 38.00 33.37
Ph
CF2H
CN
180 170 160 150 140 130 120 110 100 90 80 70 60 50 40 30 20 10 0
f1 (ppm)

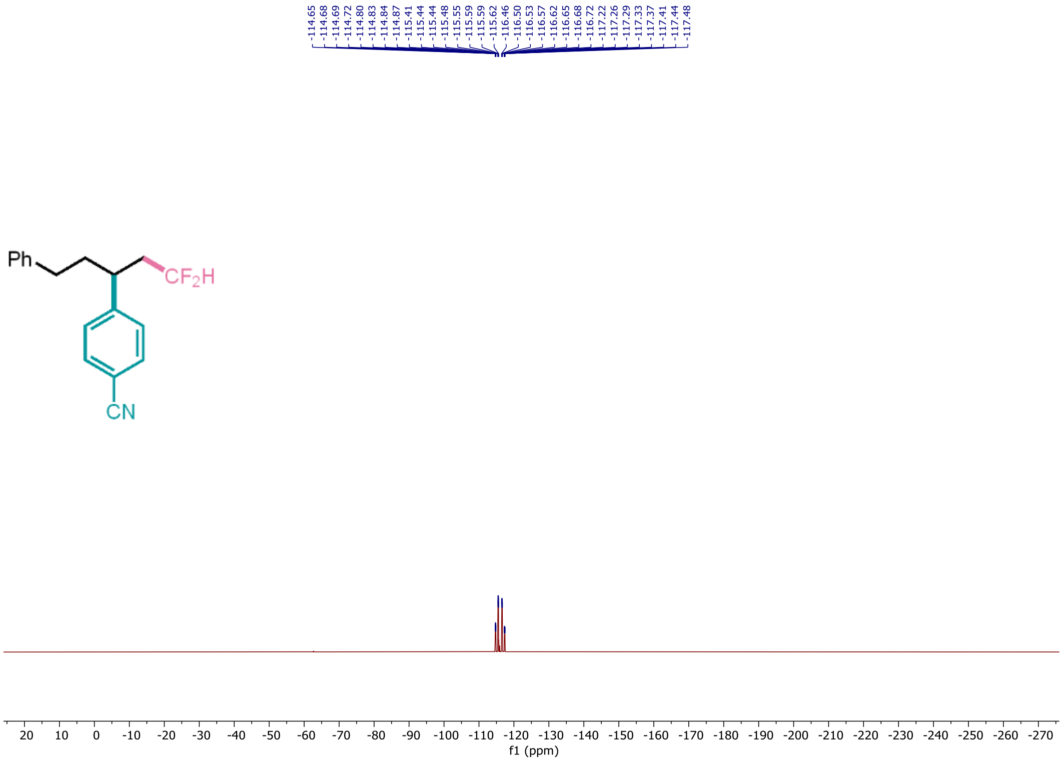

Figure S111. ^{1}H NMR (500 MHz, $CDCl_3$), ^{13}C NMR (101 MHz, $CDCl_3$) and ^{19}F NMR (376 MHz, $CDCl_3$) spectra of **56**.

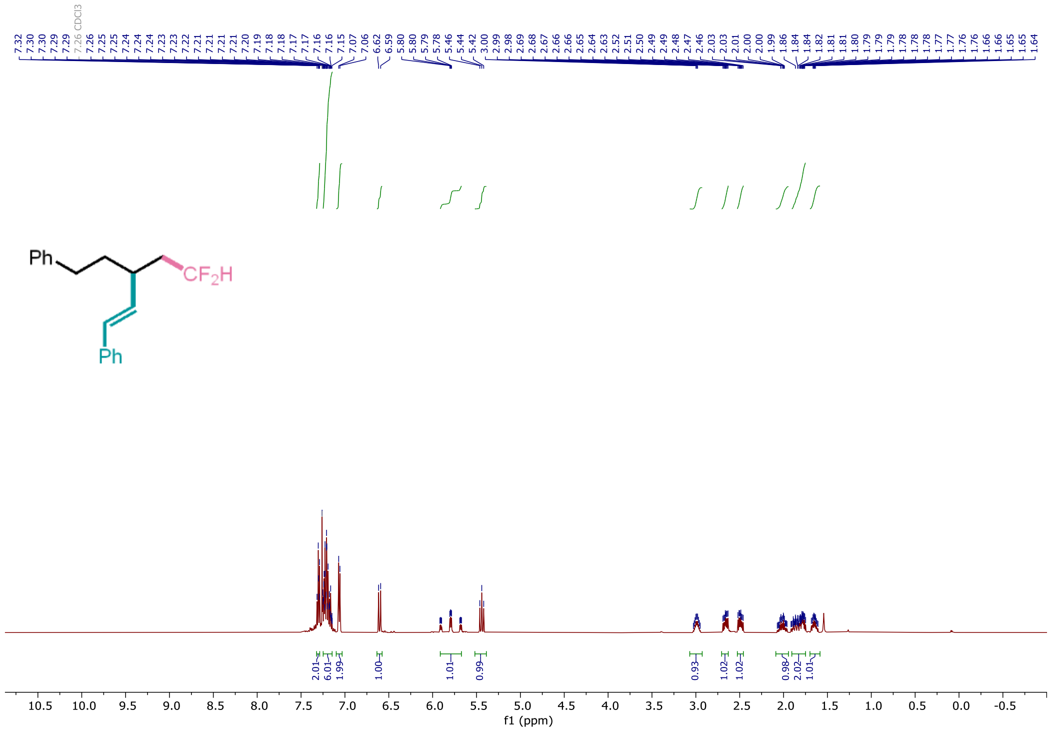
7.32 7.30 7.30 7.29 7.29 7.26 CDCl3 7.26 7.25 7.25 7.24 7.24 7.24 7.23 7.23 7.22 7.22 7.21 7.21 7.21 7.20 7.19 7.18 7.18 7.17 7.17 7.16 7.16 7.15 7.07 7.06 6.62 6.59 5.80 5.80 5.79 5.78 5.46 5.44 5.42 3.00 2.99 2.98 2.69 2.68 2.67 2.66 2.66 2.65 2.64 2.63 2.52 2.51 2.50 2.49 2.49 2.48 2.47 2.46 2.03 2.03 2.01 2.00 2.00 1.99 1.86 1.84 1.84 1.82 1.81 1.80 1.79 1.79 1.79 1.78 1.78 1.78 1.77 1.77 1.76 1.76 1.66 1.66 1.65 1.64
Ph
CF2H
Ph
2.01 6.01 1.99 1.00 1.01 0.99 0.93 1.02 1.02 0.98 2.02 1.01
10.5 10.0 9.5 9.0 8.5 8.0 7.5 7.0 6.5 6.0 5.5 5.0 4.5 4.0 3.5 3.0 2.5 2.0 1.5 1.0 0.5 0.0 -0.5
f1 (ppm)

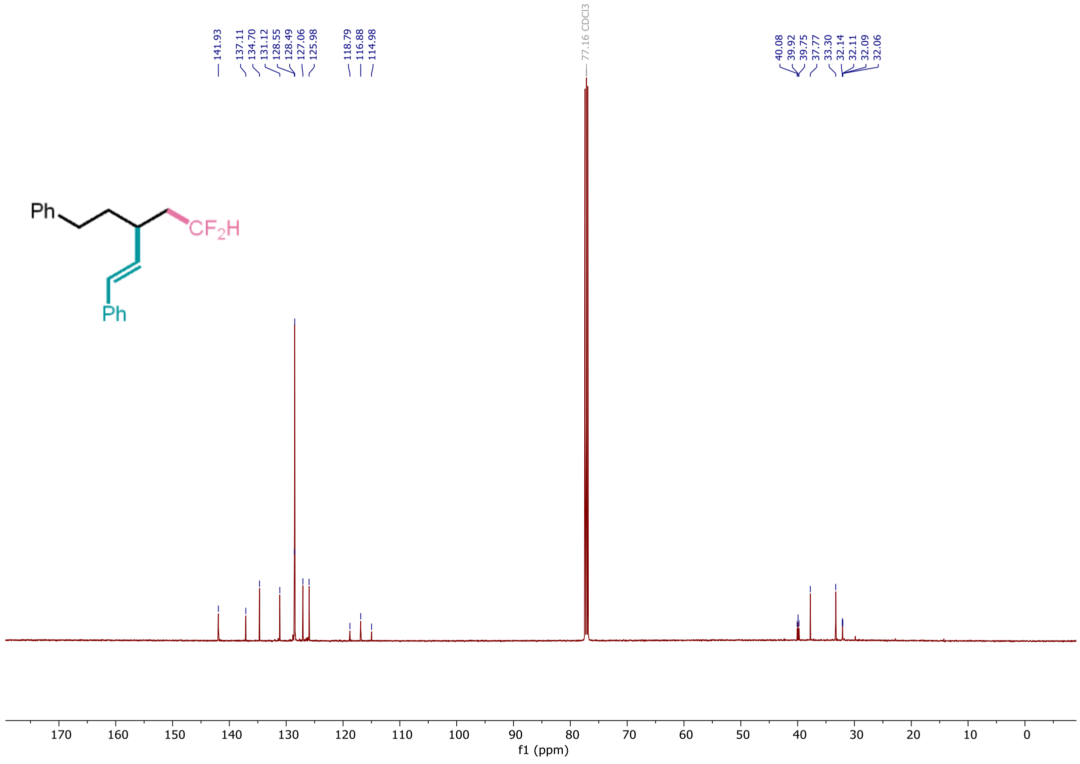
141.93 137.11 134.70 131.12 128.55 128.49 127.06 125.98 118.79 116.88 114.98 77.16 CDCl3 40.08 39.92 39.75 37.77 33.30 32.14 32.11 32.09 32.06
Ph
CF2H
Ph
170 160 150 140 130 120 110 100 90 80 70 60 50 40 30 20 10 0
f1 (ppm)

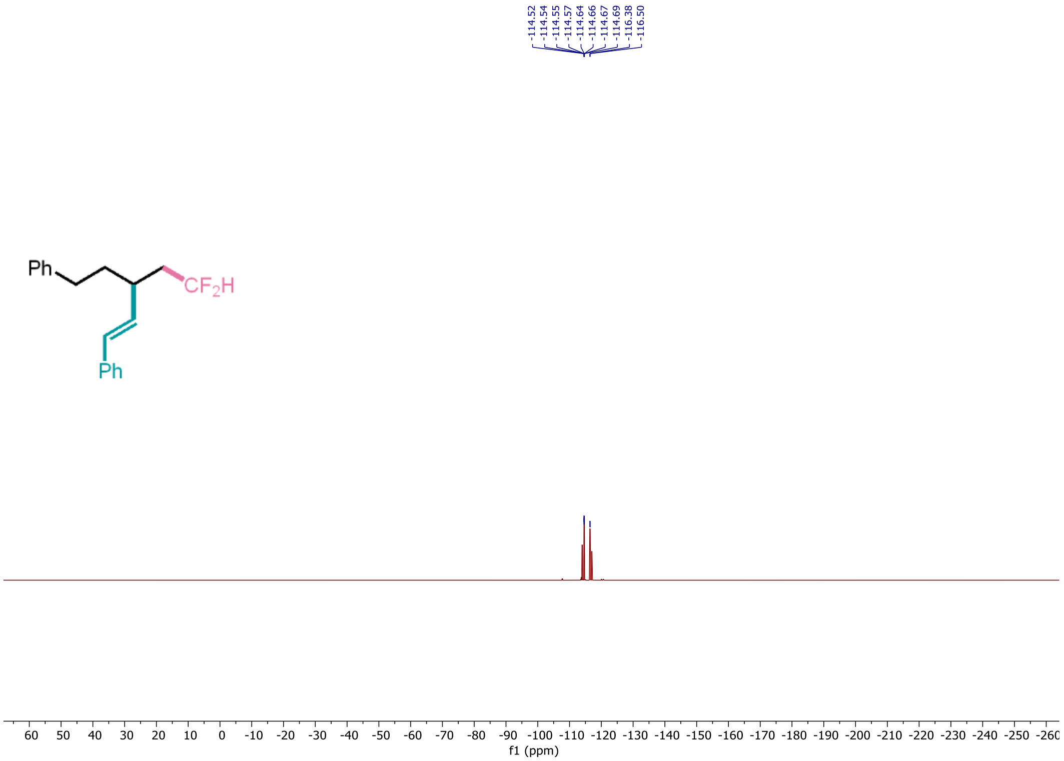

Figure S112. ^{1}H NMR (500 MHz, $CDCl_3$), ^{13}C NMR (101 MHz, $CDCl_3$) and ^{19}F NMR (376 MHz, $CDCl_3$) spectra of **57**.

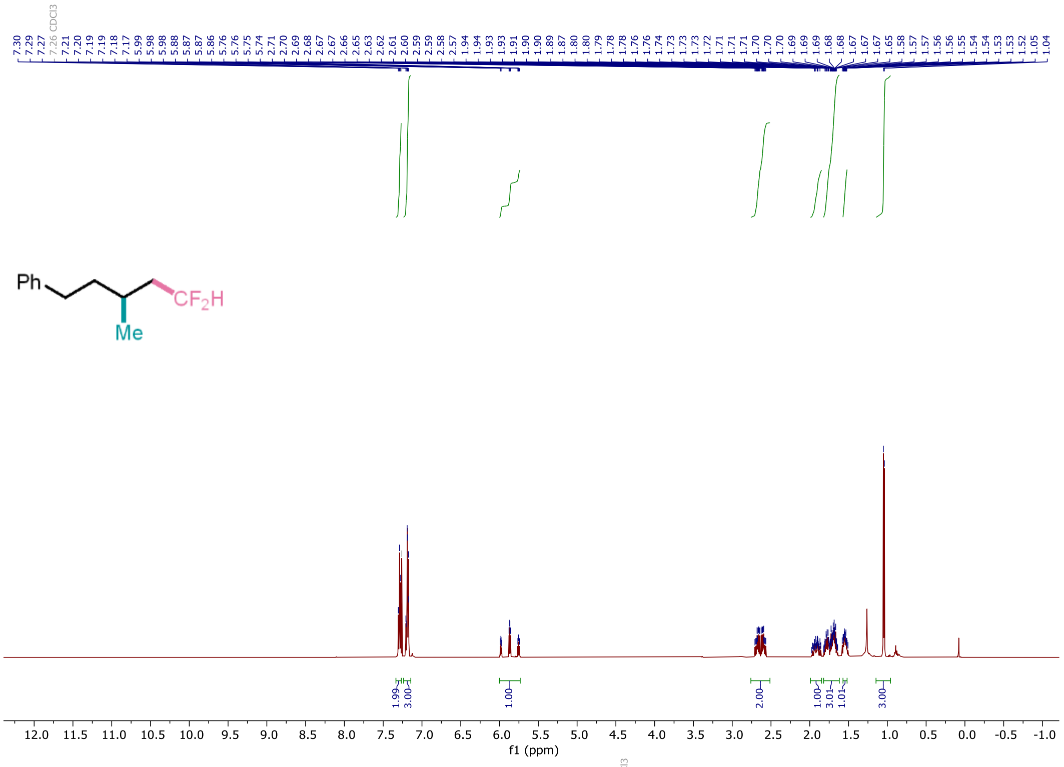
7.30 7.29 7.27 7.26 CDCl3 7.21 7.20 7.19 7.19 7.18 7.17 5.99 5.98 5.98 5.88 5.87 5.86 5.76 5.75 5.74 2.71 2.70 2.69 2.68 2.67 2.67 2.66 2.65 2.63 2.62 2.61 2.60 2.59 2.59 2.58 2.57 1.94 1.94 1.93 1.93 1.91 1.90 1.90 1.89 1.87 1.80 1.80 1.79 1.78 1.78 1.76 1.76 1.74 1.73 1.73 1.73 1.72 1.72 1.71 1.71 1.71 1.70 1.70 1.70 1.69 1.69 1.69 1.68 1.68 1.67 1.67 1.67 1.65 1.58 1.57 1.57 1.56 1.56 1.55 1.54 1.53 1.53 1.52 1.05 1.04
Ph
CF2H
Me
1.99 3.00 1.00 2.00 1.00 3.01 1.01 3.00
12.0 11.5 11.0 10.5 10.0 9.5 9.0 8.5 8.0 7.5 7.0 6.5 6.0 5.5 5.0 4.5 4.0 3.5 3.0 2.5 2.0 1.5 1.0 0.5 0.0 -0.5 -1.0
f1 (ppm)

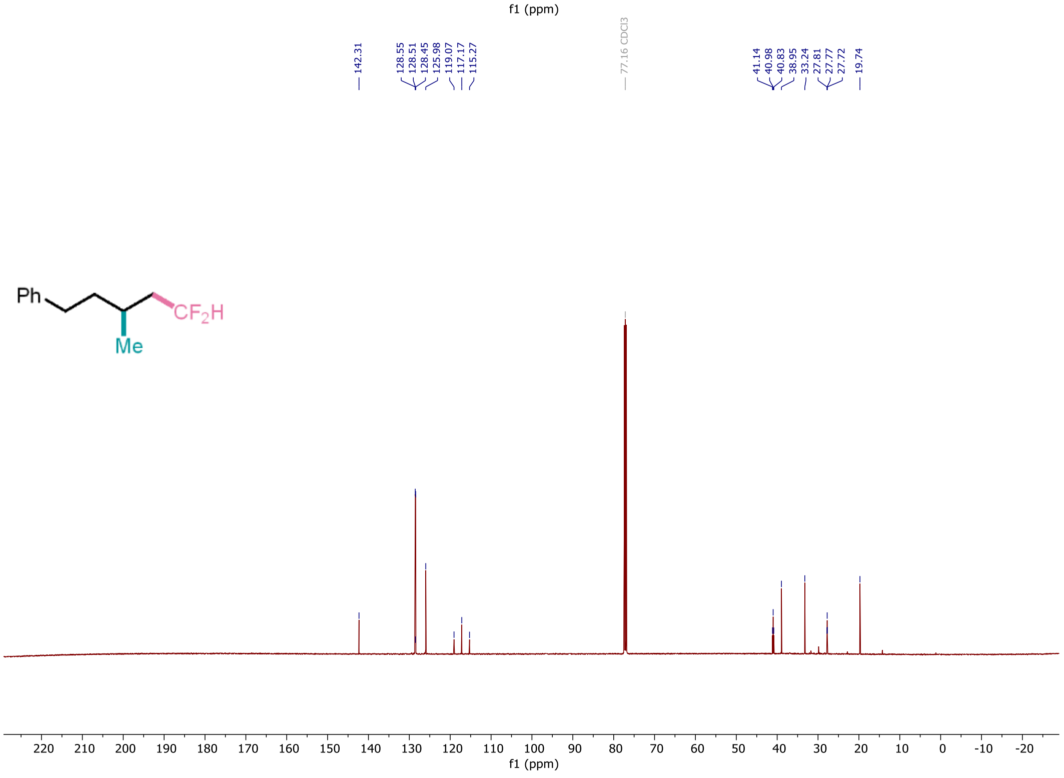
142.31 128.55 128.51 128.45 125.98 119.07 117.17 115.27
77.16 CDCl3
41.14 40.98 40.83 38.95 33.24 27.81 27.77 27.72 19.74
Ph
CF2H
Me
220 210 200 190 180 170 160 150 140 130 120 110 100 90 80 70 60 50 40 30 20 10 0 -10 -20
f1 (ppm)

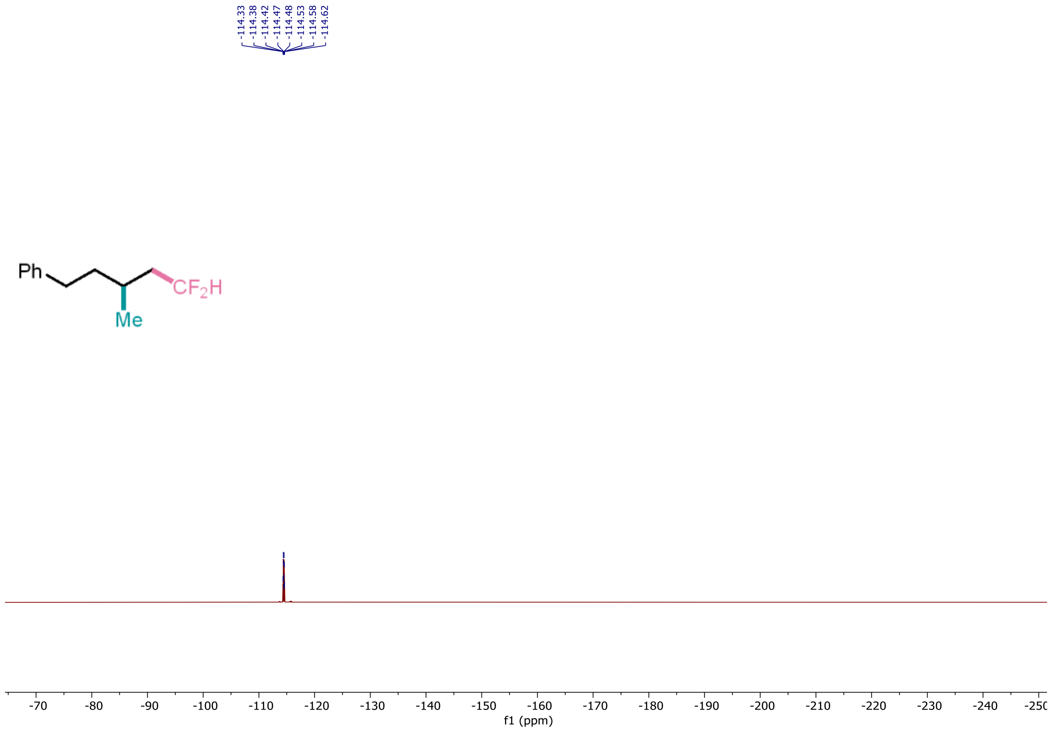

Figure S113. ^{1}H NMR (500 MHz, $CDCl_3$), ^{13}C NMR (101 MHz, $CDCl_3$) and ^{19}F NMR (376 MHz, $CDCl_3$) spectra of **58**.

20. References.

(1) He, J.; Randrianandraina, J.; Adamji, H.; Chang, V.; Lai, Y.; Nguyen, T. N.; Román-Leshkov, Y.; Kulik, H. J.; Lee, J.-H.; Milner, P. J. Photochemical Fluoroalkylations with Fluorinated Gases Facilitated by a Robust Metal–Organic Framework. *J. Am. Chem. Soc.* **2026**, *148* (1), 1369–1380.

(2) Alvarez, E.; Guillou, N.; Martineau, C.; Bueken, B.; Van de Voorde, B.; Le Guillouzer, C.; Fabry, P.; Nouar, F.; Taulelle, F.; de Vos, D.; Chang, J.; Cho, K. H.; Ramsahye, N.; Devic, T.; Daturi, M.; Maurin, G.; Serre, C. The Structure of the Aluminum Fumarate Metal–Organic Framework A520. *Angew. Chem. Int. Ed.* **2015**, *54* (12), 3664–3668.

(3) Queen, W. L.; Hudson, M. R.; Bloch, E. D.; Mason, J. A.; Gonzalez, M. I.; Lee, J. S.; Gygi, D.; Howe, J. D.; Lee, K.; Darwish, T. A.; James, M.; Peterson, V. K.; Teat, S. J.; Smit, B.; Neaton, J. B.; Long, J. R.; Brown, C. M. Comprehensive Study of Carbon Dioxide Adsorption in the Metal–Organic Frameworks M_2(dobdc) (M = Mg, Mn, Fe, Co, Ni, Cu, Zn). *Chem. Sci.* **2014**, *5* (12), 4569–4581.

(4) Dietzel, P. D. C.; Johnsen, R. E.; Blom, R.; Fjellvåg, H. Structural Changes and Coordinatively Unsaturated Metal Atoms on Dehydration of Honeycomb Analogous Microporous Metal–Organic Frameworks. *Chem. Eur. J.* **2008**, *14* (8), 2389–2397.

(5) Wang, Z.; Bilegsaikhan, A.; Jerozal, R. T.; Pitt, T. A.; Milner, P. J. Evaluating the Robustness of Metal–Organic Frameworks for Synthetic Chemistry. *ACS Appl. Mater. Interfaces* **2021**, *13* (15), 17517–17531.

(6) Siegelman, R. L.; McDonald, T. M.; Gonzalez, M. I.; Martell, J. D.; Milner, P. J.; Mason, J. A.; Berger, A. H.; Bhown, A. S.; Long, J. R. Controlling Cooperative CO_2 Adsorption in Diamine-Appended Mg_2(Dobpdc) Metal–Organic Frameworks. *J. Am. Chem. Soc.* **2017**, *139* (30), 10526–10538. https://doi.org/10.1021/jacs.7b05858.

(7) Edzards, J.; Saßnick, H.-D.; Buzanich, A. G.; Valencia, A. M.; Emmerling, F.; Beyer, S.; Cocchi, C. Effects of Ligand Substituents on the Character of Zn-Coordination in Zeolitic Imidazolate Frameworks. *J. Phys. Chem. C* **2023**, *127* (43), 21456–21464.

(8) Furukawa, H.; Gándara, F.; Zhang, Y.-B.; Jiang, J.; Queen, W. L.; Hudson, M. R.; Yaghi, O. M. Water Adsorption in Porous Metal–Organic Frameworks and Related Materials. *J. Am. Chem. Soc.* **2014**, *136* (11), 4369–4381.

(9) Øien, S.; Wragg, D.; Reinsch, H.; Svelle, S.; Bordiga, S.; Lamberti, C.; Lillerud, K. P. Detailed Structure Analysis of Atomic Positions and Defects in Zirconium Metal–Organic Frameworks. *Cryst. Growth Des.* **2014**, *14* (11), 5370–5372.

(10) Guo, L.; Hurd, J.; He, M.; Lu, W.; Li, J.; Crawshaw, D.; Fan, M.; Sapchenko, S.; Chen, Y.; Zeng, X.; Kippax-Jones, M.; Huang, W.; Zhu, Z.; Manuel, P.; Frogley, M. D.; Lee, D.; Schröder, M.; Yang, S. Efficient Capture and Storage of Ammonia in Robust Aluminium-Based Metal–Organic Frameworks. *Commun. Chem.* **2023**, *6* (1), 55.

(11) Zheng, Z.; Nguyen, H. L.; Hanikel, N.; Li, K. K.-Y.; Zhou, Z.; Ma, T.; Yaghi, O. M. High-Yield, Green and Scalable Methods for Producing MOF-303 for Water Harvesting from Desert Air. *Nat. Protoc.* **2023**, *18* (1), 136–156.

(12) Sheldon, D. J.; Parr, J. M.; Crimmin, M. R. Defluorination of HFCs by a Magnesium Reagent. *Dalton Trans.* **2024**, *53* (15), 6524–6528.

(13) Fokin, A. V.; Studnev, Yu. N.; Rapkin, A. I.; Krotovich, I. N.; Tamarinov, A. S.; Verenikin, O. V. Reactions of Fluoroolefins with Halogens in Strong Acid Medium. *Bull. Acad. Sci. USSR Div. Chem. Sci.* **1985**, *34* (10), 2128–2131.

(14) Kresse, G.; Hafner, J. *Ab Initio* Molecular Dynamics for Liquid Metals. *Phys. Rev. B* **1993**, *47* (1), 558–561.

(15) Kresse, G.; Hafner, J. *Ab Initio* Molecular-Dynamics Simulation of the Liquid-Metal–Amorphous-Semiconductor Transition in Germanium. *Phys. Rev. B* **1994**, *49* (20), 14251–14269.

(16) Kresse, G.; Furthmüller, J. Efficiency of Ab-Initio Total Energy Calculations for Metals and Semiconductors Using a Plane-Wave Basis Set. *Comput. Mater. Sci.* **1996**, *6* (1), 15–50.

(17) Kresse, G.; Furthmüller, J. Efficient Iterative Schemes for *Ab Initio* Total-Energy Calculations Using a Plane-Wave Basis Set. *Phys. Rev. B* **1996**, *54* (16), 11169–11186.

(18) Perdew, J. P.; Burke, K.; Wang, Y. Generalized Gradient Approximation for the Exchange-Correlation Hole of a Many-Electron System. *Phys. Rev. B* **1996**, *54* (23), 16533–16539.

(19) Perdew, J. P.; Burke, K.; Ernzerhof, M. Generalized Gradient Approximation Made Simple. *Phys. Rev. Lett.* **1996**, *77* (18), 3865–3868.

(20) Grimme, S.; Antony, J.; Ehrlich, S.; Krieg, H. A Consistent and Accurate *Ab Initio* Parametrization of Density Functional Dispersion Correction (DFT-D) for the 94 Elements H-Pu. *J. Chem. Phys.* **2010**, *132* (15), 154104.

(21) Blöchl, P. E. Projector Augmented-Wave Method. *Phys. Rev. B* **1994**, *50* (24), 17953–17979.

(22) Kresse, G.; Joubert, D. From Ultrasoft Pseudopotentials to the Projector Augmented-Wave Method. *Phys. Rev. B* **1999**, *59* (3), 1758–1775.

(23) Vanpoucke, D. E. P.; Lejaeghere, K.; Van Speybroeck, V.; Waroquier, M.; Ghysels, A. Mechanical Properties from Periodic Plane Wave Quantum Mechanical Codes: The Challenge of the Flexible Nanoporous MIL-47(V) Framework. *J. Phys. Chem. C* **2015**, *119* (41), 23752–23766.

(24) Wieme, J.; Vanduyfhuys, L.; Rogge, S. M. J.; Waroquier, M.; Van Speybroeck, V. Exploring the Flexibility of MIL-47(V)-Type Materials Using Force Field Molecular Dynamics Simulations. *J. Phys. Chem. C* **2016**, *120* (27), 14934–14947.

(25) Ramaswamy, P.; Wieme, J.; Alvarez, E.; Vanduyfhuys, L.; Itié, J.-P.; Fabry, P.; Van Speybroeck, V.; Serre, C.; Yot, Pascal. G.; Maurin, G. Mechanical Properties of a Gallium Fumarate Metal–Organic Framework: A Joint Experimental-Modelling Exploration. *J. Mater. Chem. A* **2017**, *5* (22), 11047–11054.

(26) Qi, X.-K.; Yao, L.-J.; Zheng, M.-J.; Zhao, L.; Yang, C.; Guo, L.; Xia, W. Photoinduced Hydrodifluoromethylation and Hydromethylation of Alkenes Enabled by Ligand-to-Iron Charge Transfer Mediated Decarboxylation. *ACS Catal.* **2024**, *14* (3), 1300–1310.

(27) Ling, J.; Haar, A. V.; Colley, K.; Kim, J.; A. Musser; Milner, J. P. Polymer Connectivity Governs Electrophotocatalytic Activity in the Solid State. *Nat. Chem.* **2025**, 17, 1853–1861.

(28) Aubrey, M. L.; Ameloot, R.; Wiers, B. M.; Long, J. R. Metal–Organic Frameworks as Solid Magnesium Electrolytes. *Energy Environ. Sci.* **2014**, *7* (2), 667.

(29) Su, Z.; Guo, Y.; Chen, Q.; Zhao, Z.; Nian, B. Catalyst-Free Hydroxytrifluoromethylation of Alkenes Using Iodotrifluoromethane. *Chin. J. Chem.* **2019**, *37* (6), 597–604.

(30) Alvarez, E. M.; Li, J.; Malapit, C. A. A General Hydrotrifluoromethylation of Unactivated Olefins Enabled by Voltage-Gated Electrosynthesis. *Angew. Chem. Int. Ed.* **2025**, *64* (4), e202415218.

(31) Keerthika, K.; Muhammed S, B.; Geetharani, K. A Metal-Free and Operationally Simple Radical Trifluoromethylative Borylation of Unactivated Alkenes. *Chem. Eur. J.* **2024**, *30* (6), e202303468.

(32) Cheng, Y.; Mück-Lichtenfeld, C.; Studer, A. Transition Metal-Free 1,2-Carboboration of Unactivated Alkenes. *J. Am. Chem. Soc.* **2018**, *140* (20), 6221–6225.

(33) Panferova, L. I.; Dilman, A. D. Light-Mediated Sulfur–Boron Exchange. *Org. Lett.* **2021**, *23* (10), 3919–3922.

(34) Liu, B.; Dong, J.; Wang, H.; Chen, J.; Liu, S.; Xiong, X.; Yuan, Y.; Zeng, X. Nickel-Catalyzed Reductive Cross-Coupling of Difluoromethylated Secondary Alkyl Bromides with Organohalides. *Chem. Commun.* **2025**, *61* (11), 2357–2360.

(35) Sorokin, A. O.; Levin, V. V.; Dilman, A. D. Visible Light Mediated Difluoroalkylation of Alkenes Using Mercaptobenzothiazole-Derived Sulfide Reagent. *Adv. Synth. Catal.* **2024**, *366* (24), 5171–5175.

(36) Trifonov, A. L.; Panferova, L. I.; Levin, V. V.; Kokorekin, V. A.; Dilman, A. D. Visible-Light-Promoted Iododifluoromethylation of Alkenes via (Phosphonio)Difluoromethyl Radical Cation. *Org. Lett.* **2020**, *22* (6), 2409–2413.
